# Enabling Fluoroalkyl-Sulfonylalkylation and Fluoroalkyl-Halogenation of Alkenes and Alkynes via Photoredox Catalysis

**DOI:** 10.1021/jacs.6c00567

**Published:** 2026-04-17

**Authors:** Supuni I. N. Hewa Inaththappulige, Ayush Acharya, Nipuna D. D. Z. Agampodi, Harshvardhan Singh, Ramesh Giri

**Affiliations:** Department of Chemistry, 8082 The Pennsylvania State University, University Park, Pennsylvania 16802, United States

## Abstract

We describe a versatile photocatalytic approach for inter- and intramolecular fluoroalkyl-sulfonylalkylation of carbon–carbon bonds in activated and unactivated alkenes and alkynes. This protocol employs fluoroalkyl sulfinate salts as bifunctional reagents to introduce fluoroalkyl and SO_2_ groups and alkyl bromides to intercept the ensuing sulfonyl intermediates in a process that creates one C­(sp^3^)-C­(sp^3^) and two C­(sp^3^)-S bonds in one step. This method is applicable for the fluoroalkyl-sulfonylalkylation of both alkenes and alkynes bearing a diverse set of functional groups and unsaturated gaseous hydrocarbons. The robustness of the method is also demonstrated by the late-stage diversification of steroids, alkaloids, and pharmaceuticals. Mechanistic insights reveal a photoredox-mediated sequential process involving fluoroalkyl radical addition, SO_2_ incorporation, and subsequent S_N_2-type displacement. Notably, the same reaction condition can be extended to achieve iodo- and bromo-trifluoromethylation via a halogen atom transfer mechanism by utilizing alkyl iodides and bromoacetyl bromides, respectively, in place of general alkyl bromides.

## Graphical abstract

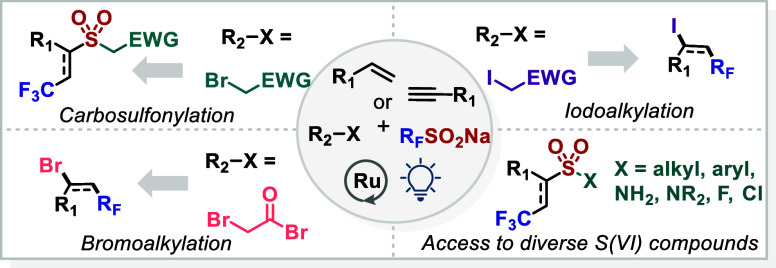

## Introduction

Fluorine and sulfur, which are ranked as the fifth and seventh most abundant elements in medicines, play a crucial role in regulating various biological processes.^1–3^ They affect metabolic pathways, therapeutic efficacy, and lipophilicity, and function as bioisosteres to modify the pharmacological properties of drugs.^4–6^ Additionally, organosulfur compounds modified with fluorine exhibit remarkable stability and resistance to oxidation.^7^ Therefore, fragments bearing these atoms are prevalent in a number of drugs,^8–10^ agrochemicals,^11,12^ and organic materials^13,14^ (Scheme 1). Traditionally, the F- and S-containing fragments are introduced into molecules independently, requiring different sets of methods in multistep processes with a variety of reagents.^15^ For example, the fluoroalkyl motifs are introduced from precursors such as CF_3_I,^16,17^ CF_3_SO_2_Cl,^18–20^ Togni’s reagent,^21,22^ Umemoto’s reagent,^23^ Hu’s reagent,^24,25^ and CF_3_SO_2_Na^26^ by utilizing redox agents, thermal, photoirradiation, or electrochemical methods.^27–30^ Sulfonylation frequently requires synthetic procedures such as the alkylation of sulfinate salts,^31,32^ the oxidation of thiol groups,^33,34^ the Friedel–Crafts-type sulfonation of arenes,^35,36^ and radical SO_2_ insertion strategies.^37–41^

**Scheme 1:**
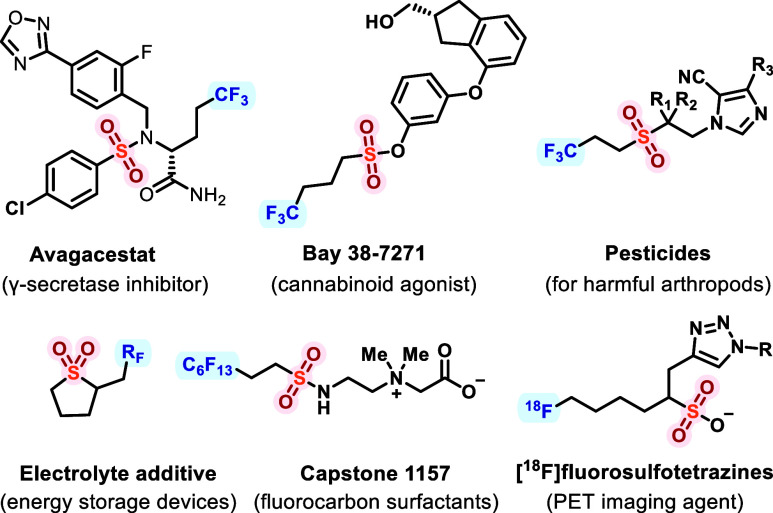
Some Important Molecules Containing C­(sp^3^)-SO_2_ and C­(sp^3^)-Fluoroalkyl Moieties

Difunctionalization of carbon–carbon unsaturated bonds is an important strategy to engineer complex molecules rapidly using simple organic feedstock.^42–48^ In this regard, simultaneous introduction of fluoroalkyl and sulfonyl groups across carbon–carbon multiple bonds would offer an expeditious route to access complex F- and S-containing molecules from readily accessible starting materials.^49,50^ Nevertheless, achieving such a reaction remains challenging.^51–57^ The difficulty arises because the conditions, catalysts, or reagents required for introducing one functional group are generally not compatible with those needed for the other (Scheme 2a). For example, generating fluoroalkyl radicals (Scheme 2a.1.i) from their sources typically requires extreme conditions such as high temperatures or strong oxidizing agents like TBPB (*tert*-butyl peroxybenzoate), *tert*-butyl hydroperoxide (*t*BuOOH), and potassium persulfate (K_2_S_2_O_8_).^58–60^ In addition, under these oxidizing conditions, the subsequent alkyl sulfinate anions (Scheme 2a.1.ii), which have a very low one-electron oxidation potential (+0.5 V vs SCE),^61–64^ can undergo radical decomposition to the original alkyl radicals with the extrusion of SO_2_ ^65–68^ in a reverse process that is counterproductive to capturing SO_2_ crucial for sulfone formation.^56,69^ The reversal activity could also increase the concentration of secondary alkyl radicals and promote the formation of undesired side products.^70,71^

**Scheme 2:**
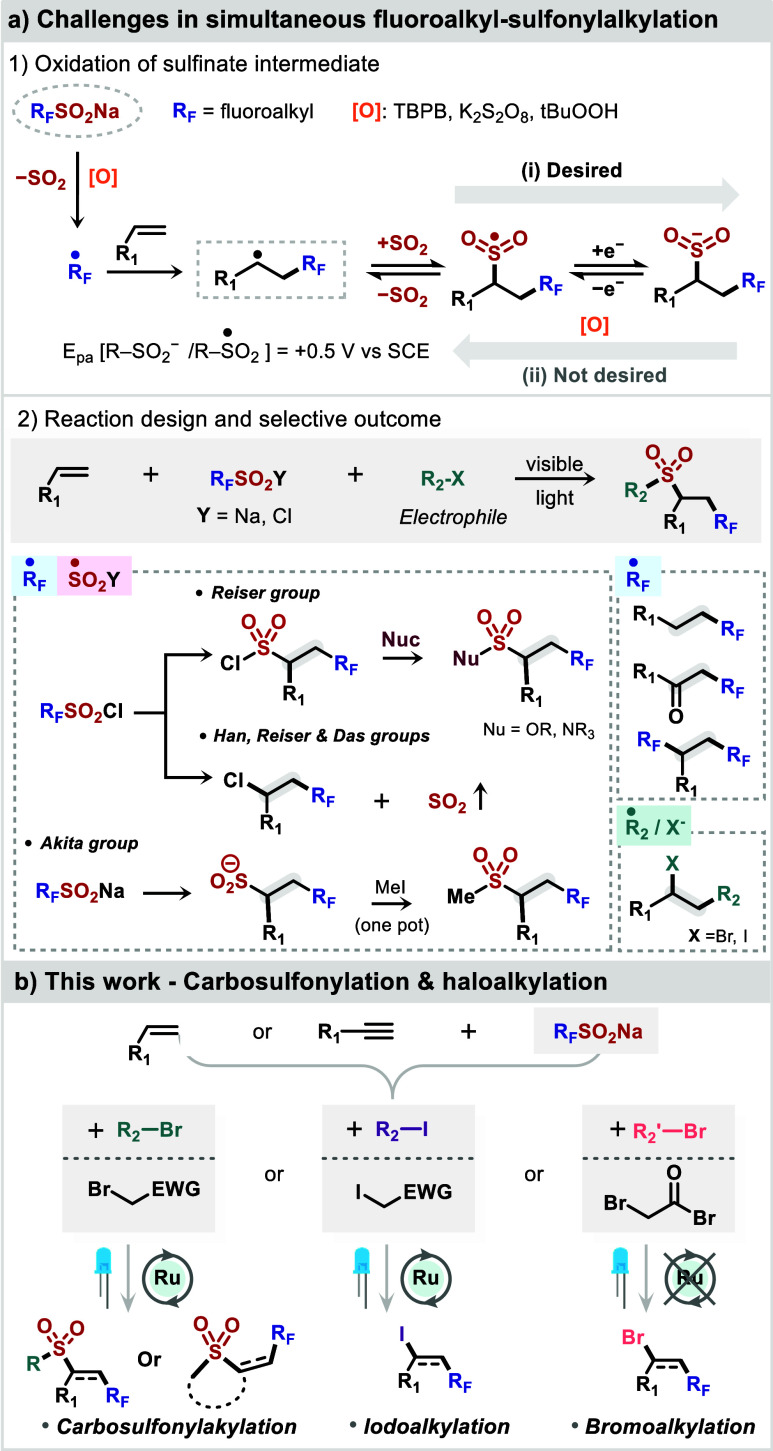
Challenges for Introducing C­(sp^3^)-SO_2_ and C­(sp^3^)-Fluoroalkyl Moieties and Current Work

This transformation involves formation of three bonds in a single operation via photooxidation of CF_3_SO_2_Na, radical addition to the alkene, and trapping with electrophiles (Scheme 2a.2).^52^ But attempting to achieve this can lead to numerous byproducts along with the desired product (Scheme 2a.2). In particular, bifunctional reagents such as CF_3_SO_2_Cl can lead to alternative reaction pathways that diverge from intended transformation.^72^ For instance, Reiser and co-workers reported a visible light-mediated copper-catalyzed carbosulfonylation of alkenes that proceeds via photoreduction of CF_3_SO_2_Cl, followed by radical addition to the alkene, and subsequent trapping with nucleophiles such as alcohols or amines.^20,50,54^ In contrast, CF_3_SO_2_Cl can also undergo SO_2_ extrusion to furnish CF_3_ radicals, thereby promoting chloroalkylation pathways instead as demonstrated by Han, Reiser, and Das groups.^19,54,73^ Additionally, the Akita group developed fluoroalkyl-sulfonylation of olefins using CF_3_SO_2_Na where the of RSO_2_^–^ anions were intercepted and trapped by alkyl iodides in a one-pot fashion.^52^

Furthermore, previous studies have shown fluoroalkyl radicals may engage in competing reaction pathways,^74–78^ generating side products through hydrogen atom transfer (HAT),^79^ HAT followed by oxidation,^80^ and bis-fluoroalkylation.^81^ A further challenge emerges when α-bromoesters are used as the alkyl sources in the reaction. In such cases, existing methods are not suitable, mainly due to catalysts, particularly Ir photocatalysts and organophotocatalysts like 4CzIPN, tend to undergo oxidative quenching in the presence of α-haloesters.^82,83^ This pathway favors carbobromination of alkenes, yielding predominantly γ-halogenated carbonyl compounds over the desired reaction. Despite these challenges, we report a mild and selective approach for the simultaneous introduction of fluoroalkyl, sulfonyl, and alkyl groups across the vicinal carbons on unactivated alkenes and alkynes in one step. This method enables access to a series of acyclic sulfones, cyclic γ-sultines, and cyclic sulfones with fluoroalkyl groups that hold promise in various application fields. Interestingly, this protocol can be tuned to enable iodo and bromo trifluoromethylation by utilizing iodoesters and bromoacetyl bromides, respectively, under similar reaction conditions (Scheme 2b).

## Results and Discussion

In our studies, we examined the reaction of 4-phenylbutene (**1**) with methyl α-bromoacetate (2) and sodium trifluoromethanesulfinate (CF_3_SO_2_Na, Langlois reagent) using different photocatalysts (PCs) in MeCN under 440 nm blue LED (Table 1, entries 1–4). Among the PCs evaluated, 2 mol % Ru­(bpz)_3_(PF_6_)_2_ furnished the difunctionalized product **3** in 75% yield under nitrogen (entry 1). Product **3** was formed as a single regioisomer with the incorporation of CF_3_ to the terminal carbon and the sulfonylalkyl group to the internal carbon of the alkene. The reaction can be conducted in air, but the product yield decreased slightly (entry 2). [Ir­{dF­(CF_3_)­ppy}_2_(bpy)]­(PF_6_), Ir­(dtbbpy)­(ppy)_2_PF_6_, eosin Y–Na, fac-Ir­(ppy)_3_, or 4-CzIPN did not afford the desired product except trace amounts of hydroalkylation **4**. When *fac*-Ir­(ppy)_3_ was used, carbobrominated product **5** was formed in 52% yield without desired product **3**. Moderate yields of product **3** were observed when 9-mesityl-10-phenylacridinium tetrafluoroborate (MPAT) and Ru­(bpy)_3_(PF_6_)_2_ were used as catalysts (entry 5). Unlike the Langlois reagent, (CF_3_SO_2_)_2_Zn was ineffective as a donor of CF_3_ and SO_2_ (entry 6). The product yield was also considerably diminished when MeCN was replaced with DMF, DMA, DMSO, DCM, or DCE (entry 7). Two mol % Ru­(bpz)_3_(PF_6_)_2_ loading at 1.5 equiv of α-bromoacetate (2) and 2 equiv of CF_3_SO_2_Na remained optimal since reducing their concentrations to 1.0 mol % or 1 equiv each also reduced the product yield (entries 8–10). Similarly, the product was formed in best yield at 440 nm since no light, ambient light, or the use of lights with wavelengths higher or lower than 440 nm also decreased the product yield (entries 11–13).

**Table 1:**
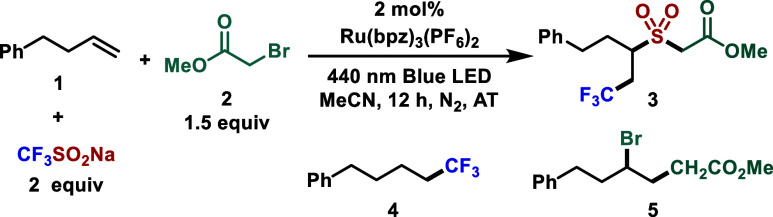
Reaction Parameter Optimization^a^

entry	variation from the standard condition	yield of 3 (%)
1	None	75 (70)
2	under open air	63
3	Ir(dtbbpy)(ppy)_2_PF_6_ or Ir(dFCF_3_ppy)(bpy)_2_PF_6_ instead of Ru(bpz)_3_(PF_6_)_2_	0
4	Eosin Y–Na, *fac*-Ir(ppy)_3_ or 4CzIPN instead of Ru(bpz)_3_(PF_6_)_2_	0
5	MPAT or Ru(bpy)_3_(PF_6_)_2_ instead of Ru(bpz)_3_(PF_6_)_2_	55, 51
6	(CF_3_SO_2_)_2_Zn instead of CF_3_SO_2_Na	0
7	DMF, DMA, DMSO, DCM or DCE instead of MeCN	trace
8	1 equiv of **2**	46
9	1 equiv of CF_3_SO_2_Na	32
10	1.0 mol % of Ru(bpz)_3_(PF_6_)_2_	44
11	390 nm LED	38
12	467 nm LED	57
13	ambient light or dark instead of blue LED	0

a Reaction conditions: 1 (1.0 equiv), 2 (1.5 equiv), CF_3_SO_2_Na (2.0 equiv), photocatalyst (1–5 mol %), solvent (0.1 M), ambient temperature (AT), 12 h, 440 nm blue LED, N_2_. Yields were determined by ^1^H NMR using the crude mixture and tetrachloroethane as the internal standard. See the SI for details.

With the optimized conditions established, we proceeded to explore the scope of the reaction with regard to alkenes, fluoroalkyl salts, and alkyl bromides (Table 2). First, we examined the scope of unactivated alkenes using CF_3_SO_2_Na and alkyl α-bromoacetate or α-bromo-*N*,*N*-dimethylacetamide as coupling reagents. As shown in Table 2A, terminal unactivated alkenes containing ether, ketone, and ester were compatible with the reaction condition affording their products in good yields (**6**–**8**). Esters containing heterocycles, such as the one derived from 2-furoate (**9**), and functional groups that are sensitive to radical conditions, like alkyl bromide (10) and trialkylsilyl (**11**), that are present in alkenes were also well tolerated in the reaction. The reaction worked well with alkenes bearing tertiary amide derived from morpholine (**12**) and amine protected by phthalimide (**13**). An alkene bearing a *Boc*-protected amine with an active hydrogen (NH) (**14**) furnished the product in a good yield. In addition, alkenes containing active alcoholic hydrogens (OH), such as those in primary alcohols (**15**), and acidic OH in phenols (**16**) also served as substrates in the reaction. More importantly, the current reaction condition was applicable to internal alkenes, which are typically more challenging than terminal alkenes because of their low polarization and steric congestion for difunctionalization. For example, the internal acyclic alkene in 3-hexene (**17**) and the internal cyclic alkenes in cyclopentene, cyclooctene, and norbornene (**18**–20) were readily difunctionalized affording their products in good yields. Moreover, the reaction condition could be extended to difunctionalize the 1,1-disubstituted alkene in 3-methylene-1-cyanocyclobutane (**21**) and the internal alkene in cyclic skipped diene (**22**). In the skipped diene, the additional alkene was preserved during the reaction. Stereoselectivity of this reaction is solely substrate dependent. The linear internal alkenes predominantly resulted in anti-addition due to steric effects (**17**).^84^ Five- and six-membered rings favor anti-addition due to steric constraints and rigidity (**18**, **20**, and **22**),^85,86^ while cyclic octene being more flexible produced both anti- and syn-addition products resulting in diastereomers (**19**).^87^ 1,1-Disubstituted alkenes with an existing diastereotopic carbon center generate the product (**21**) with low diastereoselectivity owing to the balance of steric and electronic effects.^88^ Activated alkenes, such as styrenes, dienes, and α,β-unsaturated carbonyls, were unreactive (See the Supporting Information for details).

**Table 2:**
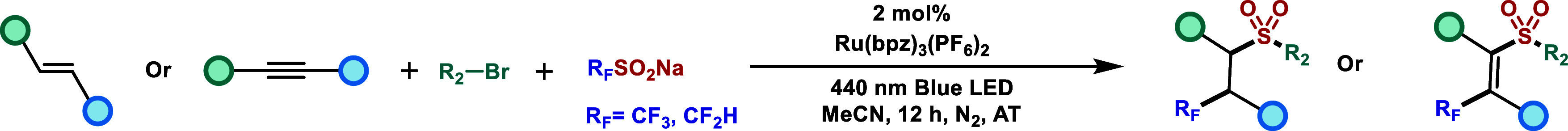

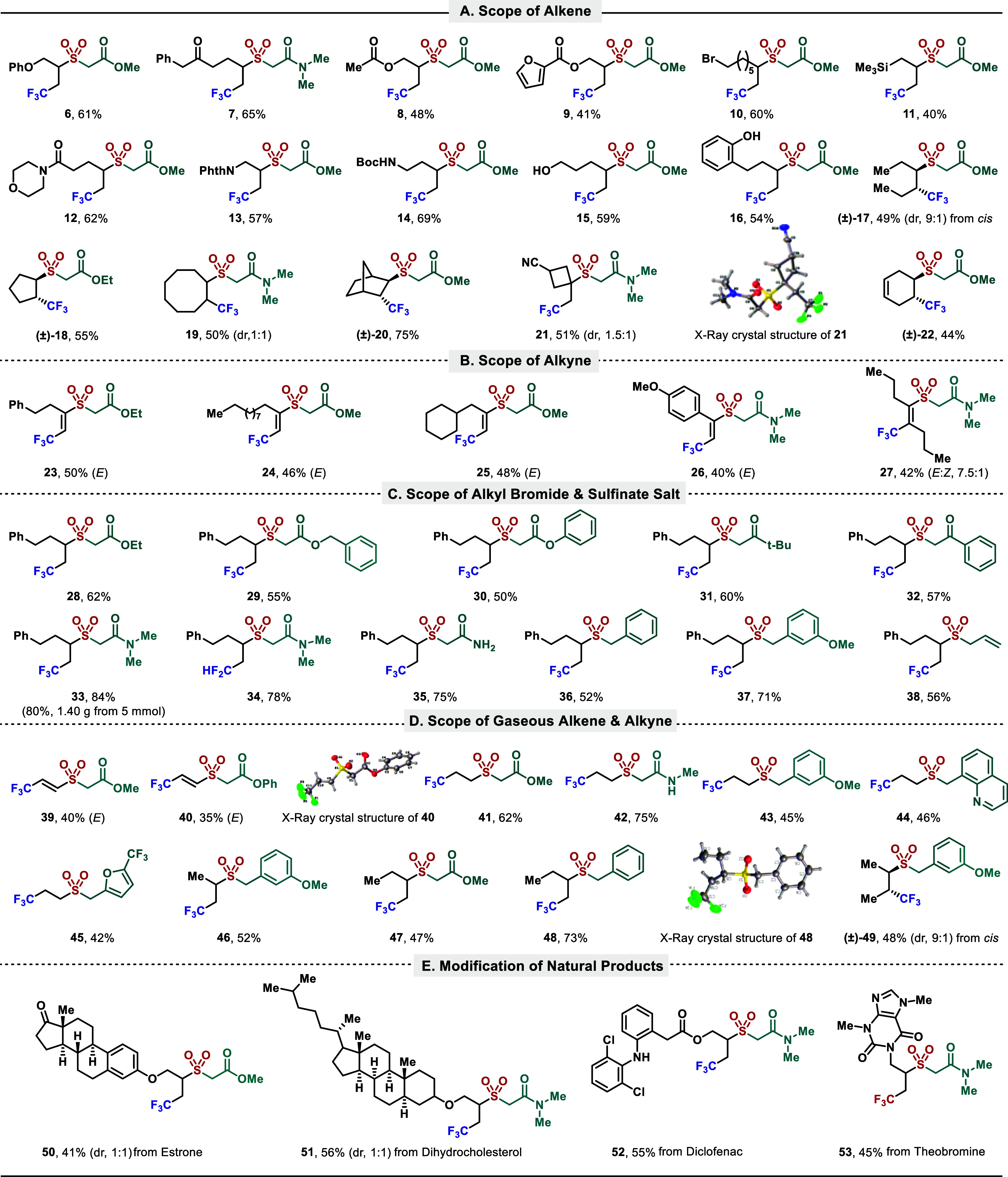
Scope of the Reaction in Alkene, Alkyne, Alkyl Halide, and Fluoroalkyl-SO_2_Na^a^

a Reaction conditions: alkene (1.0 equiv), alkyl bromide (1.5 equiv), CF_3_SO_2_Na (2.0 equiv), Ru­(bpz)_3_(PF_6_)_2_ (2 mol %), MeCN (0.1 M), 12 h, 440 nm blue LED, N_2_, AT, in vials; isolated yields are reported. The dr ratio was determined based on the ^1^H NMR analysis of the crude reaction mixture and GC. See the Supporting Information for details.

Second, we explored the reaction with alkynes (Table 2B). The difunctionalization reaction could be performed with aliphatic and aromatic alkynes, as well as internal alkynes. For example, the reaction of CF_3_SO_2_Na and alkyl α-bromoacetate or α-bromo-*N*,*N*-dimethylacetamide with 4-phenyl-1-butyne, 1-dodecyne, and 3-cyclohexyl-1-propyne generated their alkyne-difunctionalized products (**23**–**25**) in good yields. The regiochemistry was similar to that of the alkene with the addition of CF_3_ to the terminal carbon and the sulfonylalkyl group to the internal carbon of the alkyne. Similarly, the terminal alkyne in 4-ethynylanisole (**26**) and the internal alkyne in 4-octyne (**27**) were also difunctionalized to afford their products in good yields. The reaction of terminal alkynes yielded anti-addition products as a single configurational stereoisomer. The internal alkyne also generated the product (**27**) in high stereoselectivity.^89–91^ The high stereoselectivity is likely due to the steric and electronic effects, which stabilize intermediates or transition states favoring anti-addition.^92–94^ Thermodynamically more stable *E*-isomer was thus obtained as the major stereoisomer.

Next, we examined the scope of the reaction in alkyl halides and sulfinate salts (Table 2C). The reaction showed a broad scope in terms of alkyl halides and worked well with benzyl halides and allyl halides in addition to α-halocarbonyls. As shown in Table 2C, the alkene in 4-phenylbutene (**1**) could be readily difunctionalized with CF_3_SO_2_Na using the alkyl, benzyl, and phenyl esters of α-bromoacetic acid (**28**–30). α-Bromoketones, such as α-bromopinacolone and α-bromoacetophenone, could also serve as alkyl sources, enabling the formation of the difunctionalized products (**31**–**32**) in good yields. Likewise, the reaction furnished good to excellent yields of products (**33**–**34**) when α-bromo-*N*,*N*-dimethylacetamide was used. More importantly, the reaction was compatible with a primary α-bromoamide containing two active hydrogens (NH_2_) (**35**). Additionally, the α-bromocarbonyls could be readily replaced with benzyl bromide, 3-methoxybenzyl bromide, and allyl bromide as alkyl halides, and the corresponding trifluoromethyl-sulfonylalkylated products (**36**–**38**) could be obtained in good yields. During our studies, we also integrated HCF_2_SO_2_Na as a fluoroalkyl source. To our delight, this reagent was similarly effective and furnished the difluoromethylsulfonylalkylated product (**34**) in excellent yield. Finally, the reaction could be conducted in large scales (5 mmol) at a slightly longer reaction time (16 h) but without compromising yields, as demonstrated for the reaction of 4-phenylbutene **1** with CF_3_SO_2_Na and α-bromo-*N*,*N*-dimethylacetamide affording the product **33** in 80% yield (1.40 g).

Reactions of unsaturated gaseous hydrocarbons, particularly ethylene and acetylene, struggle for difunctionalization since they are completely unpolarized.^95–97^ In addition, the presence of reactive radicals also causes these unsaturated hydrocarbons to undergo polymerization.^98^ Gratifyingly, our difunctionalization method was applicable to ethylene, acetylene, and other terminal and internal gaseous alkenes (Table 2D). Acetylene gas generated trans-alkenes featuring CF_3_ and SO_2_CH_2_CO_2_R as two strongly electron-withdrawing groups upon reaction with CF_3_SO_2_Na and α-bromoesters (**39**–40). Similarly, ethylene could be reacted with CF_3_SO_2_Na along with α-bromoacetic acid ester and amide and benzyl bromides to afford the corresponding products (**41**–**45**) in good yields. In particular, ethylene could be difunctionalized with CF_3_SO_2_Na using heterocyclic benzyl bromides, such as 8-quinolinylmethyl bromide and 5-(trifluoromethyl)-2-furylmethyl bromide and introduce heterocycles to trifluoromethylsulfonylated products (**44**–**45**). Additional gaseous alkenes containing terminal and internal alkenes, such as 1-propene, 1-butene, and 2-butene, could be difunctionalized with CF_3_SO_2_Na and α-bromoesters or benzyl bromides (**46**–**49**). The structure of the trifluoromethyl-sulfonylalkylated product **48** was determined by single-crystal X-ray crystallography, further confirming the regiochemistry.

Furthermore, we evaluated alkenes attached to natural products and pharmaceuticals containing heterocyclic motifs as a way to showcase the method’s synthetic application to complex molecular architectures (Table 2E). Under the standard conditions, alkenes tethered to steroids, such as estrone and dehydrocholesterol, successfully reacted to yield the desired products (50–**51**) in good yields. Pleasingly, alkenes attached to the pharmaceutical diclofenac containing chlorinated diarylamine and the alkaloid theobromine bearing a complex heterocycle were also readily difunctionalized, affording the products (**52**–**53**) in good yields.

Cyclic sulfones and sultines are sulfur-containing heterocycles with application in medicine and materials, making their synthesis essential.^99,100^ This method could also employ alkene-tethered alkyl bromides or α-bromocarbonyls as starting materials for an intramolecular process to generate a variety of fluoroalkyl-decorated cyclic sulfones (Table 3). The reaction was applicable for the formation of five- and six-membered cyclic sulfones (**54**–**57**) by the interception of SO_2_ during the cyclization of 5-bromo-1-pentene, diethyl 2-allyl-2-(bromomethyl)­malonate, and 6-bromo-1-hexene, respectively. The structure of sulfone **56** was also confirmed by single-crystal X-ray crystallography. Both CF_3_SO_2_Na and HCF_2_SO_2_Na were amenable as a source of SO_2_ and fluoroalkyls. Similarly, *N*-allyl-α-bromoacetamide could be cyclized with the incorporation of SO_2_ to generate thiomorpholin-3-one 1,1-dioxide (**58**). However, 4-bromo-1-butene underwent intramolecular cyclization through O rather than S following SO_2_ insertion. As a result, 4-bromo-1-butene generated five-membered γ-sultines predominantly as trans-isomers (**59**–60) as the major product instead of four-membered sulfones. Likewise, 4-bromo-1-butyne could also be cyclized with CF_3_SO_2_Na to form a γ-sultine with an exocyclic alkene (**61**).^83,90,91^ The reaction of 5-bromo-2-methylpent-2-ene (**62**) furnished the sulfone product **65** in which CF_3_ was added to the alkenyl carbon proximal to the alkyl bromide in an apparent regioreversal process. This reversed addition arose from the preference to generate a 3° (**63**) over a 2° radical and the ultimate cyclization of the intermediate **64** to create a 5-membered (**65**) over a 4-membered heterocycle.

**Table 3:**
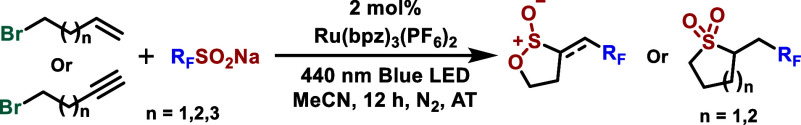

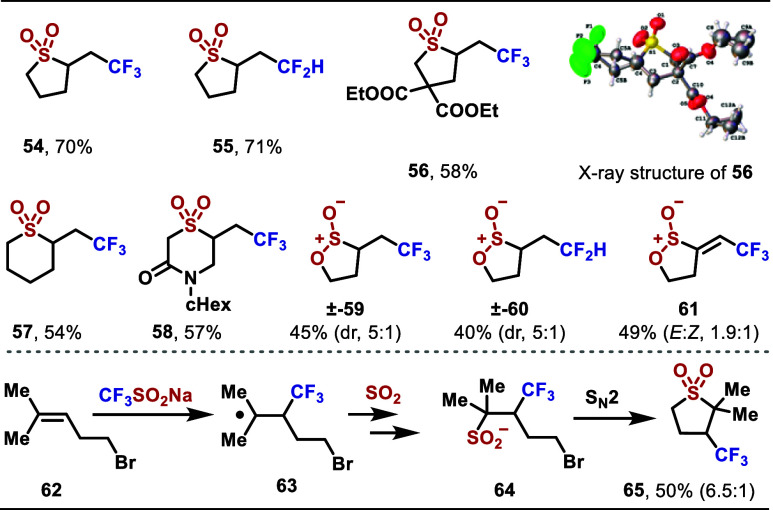
Scope of the Cyclization Reaction^a^ ^,^ ^b^

a Reaction conditions: Alkyl bromide (1.0 equiv), CF_3_SO_2_Na (2.0 equiv), Ru­(bpz)_3_(PF_6_)_2_ (2 mol %), MeCN (0.1 M), 12 h, 440 nm blue LED, N_2_, AT, in vials; isolated yields are reported.

b The dr ratio was determined based on the ^1^H NMR analysis of the crude reaction mixture and GC. See the Supporting Information for details.

The process of intercepting sulfonyl anions, which are generated in situ through photoredox catalysis, could be utilized to synthesize a range of sulfur­(VI)-containing molecules, such as alkyl and aryl sulfones, sulfonamides, and sulfonyl halides, by a two-step, one-pot protocol upon reaction with diverse electrophiles without having to purify reaction intermediates (Table 4). Treating the in situ-generated sulfonyl anion with a base and secondary alkyl iodide allowed for the generation of alkyl sulfones **66** in moderate yields. When diphenyliodonium trifluoromethanesulfonate and 2-bromothiazole were used, aryl sulfones **67–68** were obtained in good to moderate yields. This method could also be applied to the synthesis of sulfonamides for creating medicinally significant substituted sulfonamides (69–70).^101^ Sulfonyl fluorides, involved in sulfur­(VI)-fluoride exchange (SuFEx) click chemistry,^102,103^ are garnering increasing interests in drug discovery and materials research. This method also facilitated the concurrent integration of CF_3_ and sulfonyl fluoride (**71**), and CF_3_ and sulfonyl chloride (**72**) into unactivated alkenes in high yields, providing a versatile method essential for pharmacokinetic investigations.^104–107^

**Table 4:**
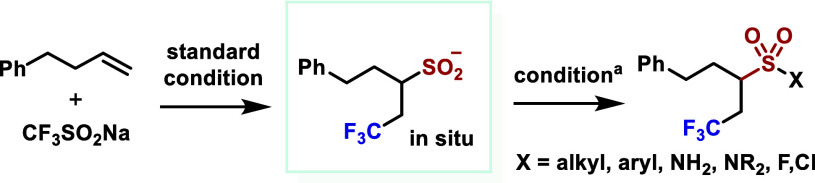

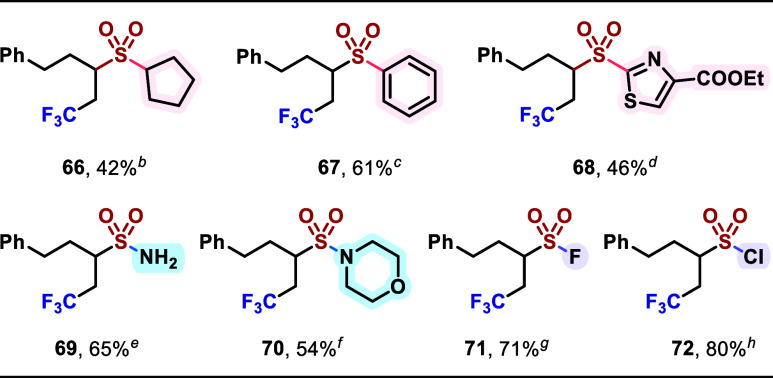
In Situ Interception of Sulfonyl Anion for Diverse Derivatization^a^

a Reactions were run at a 0.50 mmol scale. All yields are isolated. For the detailed reaction conditions, see the Supporting Information. The percentage numbers are the yields of isolated products.

b Alkyl iodide, K_2_CO_3_, MeCN, 60 °C.

c Diphenyliodonium trifluoromethanesulfonate, DMF, 110 °C.

d 2-bromothiazole, DMSO, 110 °C.

e HSOA, NaOAc,H_2_O.

f Morpholine, SO_2_Cl_2_, THF, 0 °C to rt.

g NFSI, DIPEA, DCM.

h SO_2_Cl_2_, THF, 0 °C to rt.

In addition to developing the method, we have also conducted preliminary mechanistic studies to deduce the working underpinnings of the fluoroalkyl-sulfonylalkylation reaction and proposed a possible mechanism, as illustrated in Scheme 3. Initially, the excited-state photocatalyst Ru­(bpz)_3_^2+^ (*E* ^II*/I^ = +1.45 V vs SCE in MeCN) accepts a single electron from CF_3_SO_2_Na and generates ^•^CF_3_ with the concomitant release of SO_2_.^108,109^ The resulting •CF_3_ then adds to an alkene, generating a stabilized *sec*-alkyl radical (**73**). The alkyl radical **73** subsequently captures SO_2_ to form a sulfonyl radical (**74**), which then accepts an electron from the reduced photocatalyst Ru­(bpz)_3_^+^ to produce a sulfonyl anion (**75**) and regenerate photocatalyst Ru­(bpz)_3_^2+^. Finally, the sulfonyl anion **75** reacts with alkyl bromides off-cycle by nucleophilic substitution to yield the final product **76**.

**Scheme 3:**
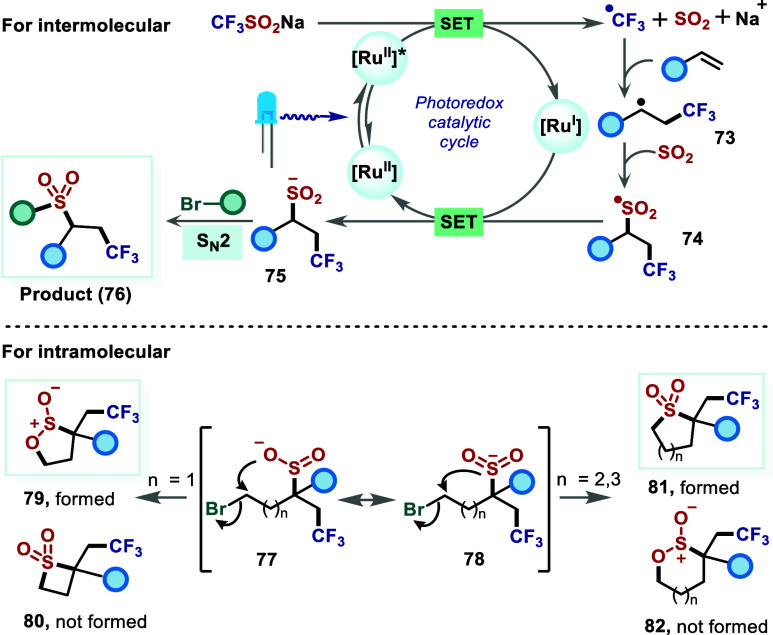
Proposed Catalytic Cycle

In the intramolecular setting, the reaction preferentially afforded five- and six-membered cyclic sultines and sulfones even with unactivated alkyl bromides whereas the intermolecular reactions failed under the same condition unless it was heated at 60 °C in the presence of a base (**66**).^110^ This discrepancy may arise from the intrinsic enthalpy and entropic advantages of intramolecular cyclization. This rationale also explains the preference for 4-bromo-1-butene and butyne to yield five-membered sultine (**79**) over four-membered sulfone (80). In the 5-bromo-1-pentene case, the reaction favored a five-membered sulfone (**81**) over the six-membered product (**82**). This specific kinetically favored selectivity for five-membered rings could be due to the added thermodynamic stability, resulting from an optimal balance between angle strain and entropy.^111^

We conducted a series of experiments to deduce the presence of radical and anionic intermediates (Scheme 4). The Stern–Volmer quenching studies revealed fluorescence quenching with fluoroalkyl sulfinates but not with α-bromoesters, indicating the formation of ^•^CF_3_ (Scheme 4D). The presence of ^•^CF_3_ and the subsequent *sec*-alkyl radical **73** was confirmed by HRMS/LCMS and ^19^F NMR as their 2,2,6,6-tetramethylpiperidine-1-oxyl (TEMPO)-adducts **83** and **84** from the standard reaction (Scheme 4A).^112,113^ The addition of TEMPO completely suppressed the difunctionalization reaction and led to the formation of these radical intermediates. In addition, the *sec*-alkyl radical **73** and the SO_2_-captured sulfonyl radical **74** also underwent dimerization to generate their dimers **87** and **88**, as confirmed by HRMS/GCMS, when *n*-PrBr was used as an unreactive alkyl halide (Scheme 4A). We have gathered further evidence for the presence of the *sec*-alkyl radical **73** in the reaction by radical clock experiments and a chiral racemic probe. The vinylcyclopropyl (**89**) and 1,6-heptadienyl (**91**) radical clocks yielded the ring-opened product 90 and the cyclized product **92** (Scheme 4B), respectively. Similarly, the reaction of chiral racemic allylic ether (**93**) generated two diastereomers with barely any stereocontrol (dr, 1.2:1) (Scheme 4C). These experiments support the proposed free-radical mechanism. Light on/off experiments and quantum yield measurements indicated that the reaction did not involve radical chain propagation but rather followed a closed photoredox catalytic cycle (see the Supporting Information for details).^114,115^

**Scheme 4:**
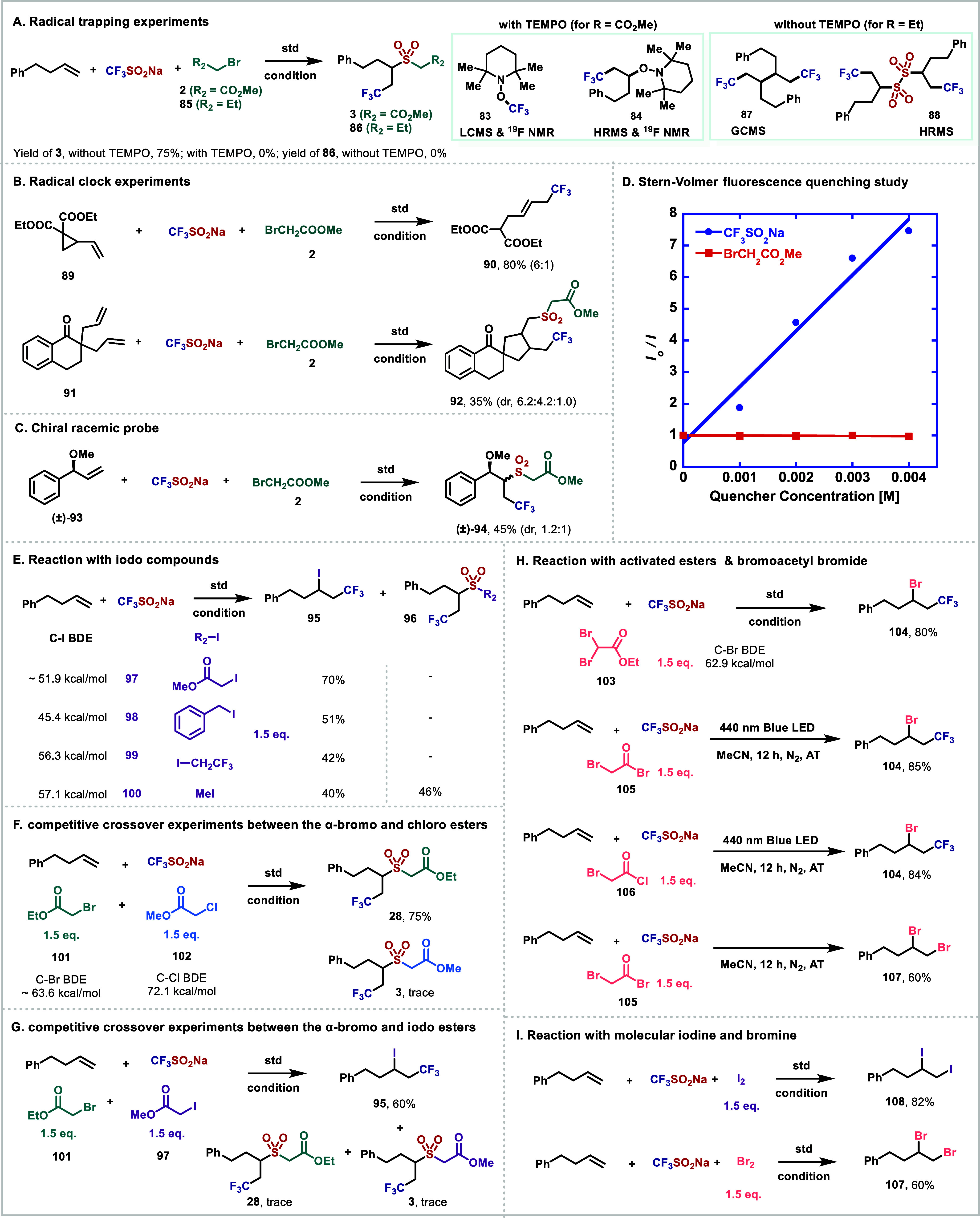
Mechanistic Studies: (A) Radical Trapping and Dimerization Experiments, (B) Radical Clock Experiment, (C) Chiral Racemic Probe, (D) Stern–Volmer Quenching Studies, (E) Reactivity of Iodo Compounds under Standard Conditions, (F) Three-Way Competition Experiment with α-Bromo and α-Chloro Substrates, (G) Three-Way Competition Experiment with α-Bromo and α-Iodo Substrates, (H) Reactivity Trend of Activated α-Bromo Compounds, and (I) Reactivity with Molecular Iodine and Bromine, See the Supporting Information for Details

During our mechanistic studies, we observed that when switching the α-bromoester with α-chloroester, it resulted in lower yield of product **3** to 31% (see the Supporting Information). In contrast, when bromo α-ester was replaced with iodo α-ester (**97**) rather than anticipated carbosulfonylation, it resulted in fluoroalkyl iodination of alkene (**95**) in 70% yield (Scheme 4E). Remarkably, this reactivity pattern was consistent when other iodo-compounds were used such as benzyl iodide (**98**), methyl iodide (MeI, 100), or trifluoroethyl iodide (ICH_2_CF_3_, **99**), albeit in varying yields. To better understand this preference for iodofluoroalkylation over carbosulfonylation, we investigated the relative reactivity and selectivity of radical intermediate **73** by conducting a series of crossover competition experiments. In three-way competitive experiments, between the α-bromo ethyl ester and α-chloro methyl ester, the reaction predominantly resulted as expected from α-bromo ester undergoing effective S_N_2 nucleophilic addition (Scheme 4F). The crossover between α-bromo ethyl ester and α-iodo methyl ester primarily yielded iodotrifluoromethylated products (**95**) as the major species (Scheme 4G).

Theoretically, iodo α-ester ought to be more S_N_2-active due to its superior leaving group ability and lower C–I bond dissociation energy (BDE).^116,117^ However, the divergence of the S_N_2 product formation with iodo α-ester can be rationalized to the preference of *sec*-alkyl radical **73** to undergo halogen atom transfer (XAT) over SO_2_ addition, furnishing the iodotrifluoromethylated product (95). In this scenario, α-Bromoesters exhibit a balanced reactivity supporting efficient S_N_2 but avoid XAT. In contrast, α-chloroesters display poor reactivity due to the strong C–Cl bond and sluggish leaving group ability, limiting both S_N_2 and XAT pathways.

Scheme 5a outlines the proposed catalytic cycle for the iodotrifluoromethylation of alkenes. Following initial radical formation, similar to the carbosulfonylation mechanism, the secondary alkyl radical (**73**) undergoes XAT with an iodinated species (**109**), forming an acyl radical (**111**). This intermediate regenerates the photocatalyst and subsequently undergoes protonation to yield ketone **112**, as verified by crude NMR and deuterium-labeling experiments. A control experiment with I_2_ and CF_3_SO_2_Na produced diiodinated product **108**, indicating no in situ I_2_ formation and supporting the XAT pathway via iodine radicals (Scheme 4I). The observed XAT preference is attributed to favorable C–I bond formation and polarity matching between the nucleophilic radical (**73**) and electrophilic iodine.^118^ Given the importance of iodides in downstream transformations, the development of single-step methods for incorporating both iodine and CF_3_ groups is well justified. The new optimized conditions for iodotrifluoromethylation of alkenes demonstrate compatibility with both alkene and alkyne substrates (Table 5).

**Scheme 5:**
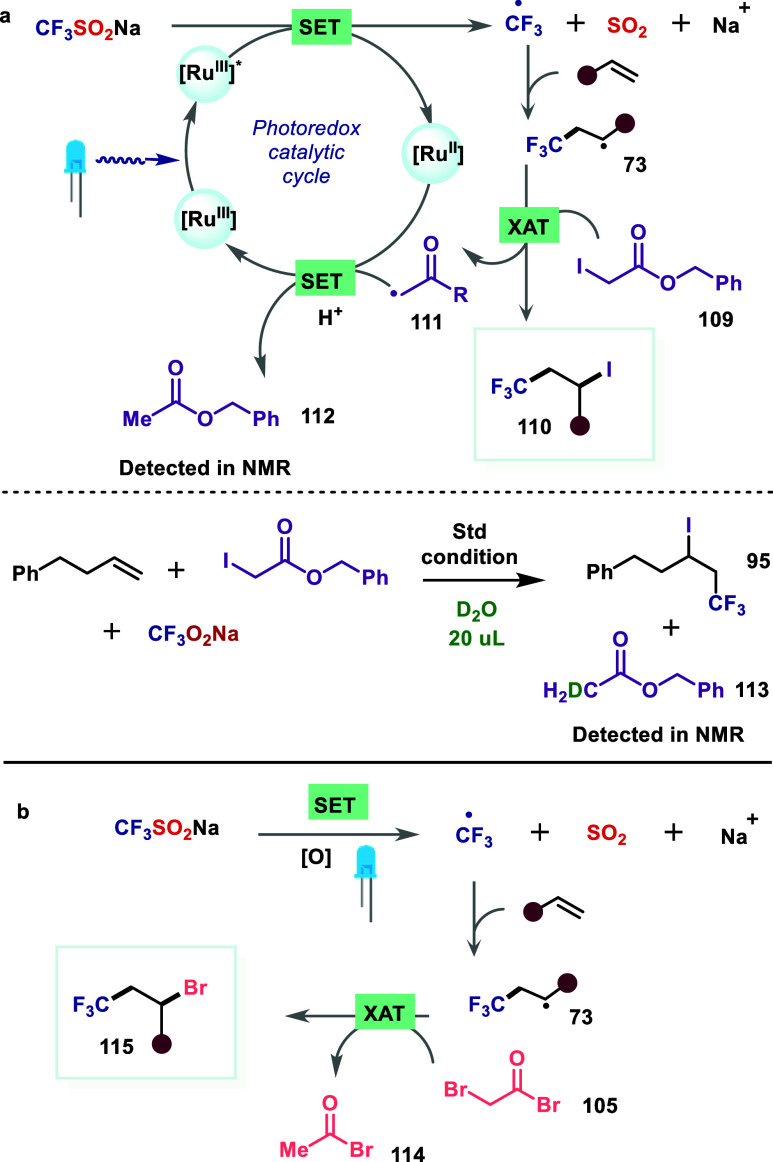
Proposed Catalytic Cycle (a) Iodotrifluoromethylation and (b) Bromotrifluoromethylation of Alkenes

**Table 5:**
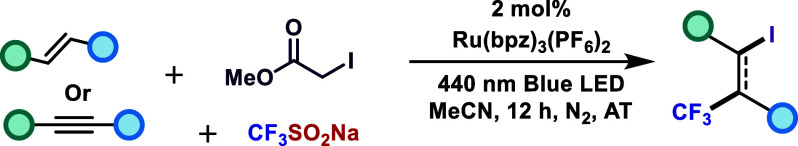

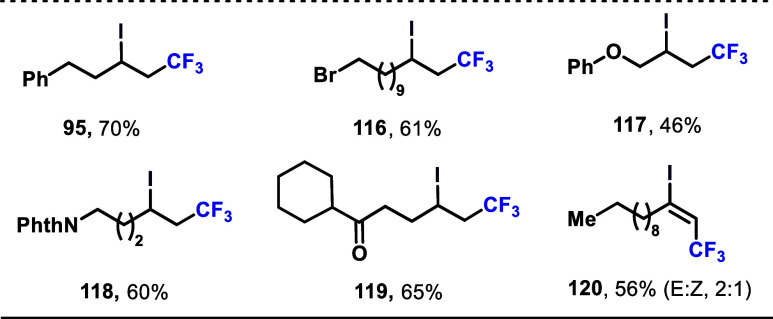
Scope of Iodotrifluoromethylation of Alkenes and Alkynes^a^

a Reaction conditions: Alkene or alkyne (1.0 equiv), **97** (1.5 equiv), CF_3_SO_2_Na (2.5 equiv), Ru­(bpz)_3_(PF_6_)_2_ (2 mol %), MeCN (0.1 M), AT, 12 h, 440 nm blue LED, in vials; isolated yields are reported. The dr ratio was determined based on the ^1^H NMR analysis of the crude reaction mixture and GC. See the Supporting Information for details.

As we progressed testing other activated α-bromoester analogues, when highly activated α,α-dibromo esters (**103**) with alkenes were used, the reaction selectively generated bromotrifluoromethylated alkenes (Scheme 4H). To our surprise, bromoacetyl bromide (**105**) and bromoacetyl chloride (**106**) enabled this outcome, even without the need for a photocatalyst. In addition, we also observed that under dark conditions, the same reagents led to the dibrominated product (**107**). This may be because the fluoroalkyl radical generation is suppressed in dark, and conventional electrophilic dibromination dominates. Similarly, external addition of Br_2_ led predominantly to dibromination, as seen with I_2_, underscoring the importance of the need for radical initiation to prevent unselective electrophilic halogenation. The proposed catalytic pathway involves generation of CF_3_^•^ radicals from CF_3_SO_2_Na upon irradiation which eventually added to the alkene to form the alkyl radical intermediate (**73**). The resulting alkyl radical undergoes halogen atom transfer with bromoacetyl bromide, completing the reaction (Scheme 5b). Overall, this condition shows that intermediate **73** exhibits distinct reactivity in the presence of different reagents, leading to variation in the product preference.

All other trifluoromethylation reactions reported here require a photocatalyst. However, the bromotrifluoromethylation reaction does not. This is likely because other haloesters (**103** and **97**) can undergo oxidative quenching and form carbohalogenation products without incorporating the CF_3_ group. Therefore, rapid CF_3_ radical generation via a photocatalyst is necessary to favor the desired product and suppress the side reactions. In contrast, bromoacetyl bromide is not known to undergo carbohalogenation, and thus, bromotrifluoromethylation is the major product formed under light. To support this, control experiments show that direct photolysis of CF_3_SO_2_Na can generate radicals in the absence of a catalyst, as seen by the 14% yield of the hydrotrifluoromethylated product (see the Supporting Information for details). This process may be facilitated by the presence of oxygen in air, which could serve as an oxidant to promote SET. This methodology was well extended to catalyst-free bromotrifluoromethylation across a range of internal and terminal alkenes and alkynes (Table 6).

**Table 6:**
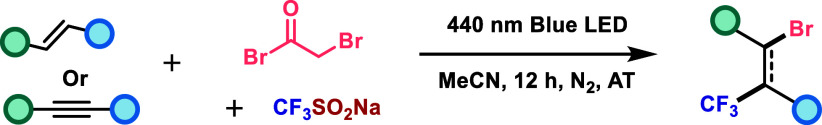

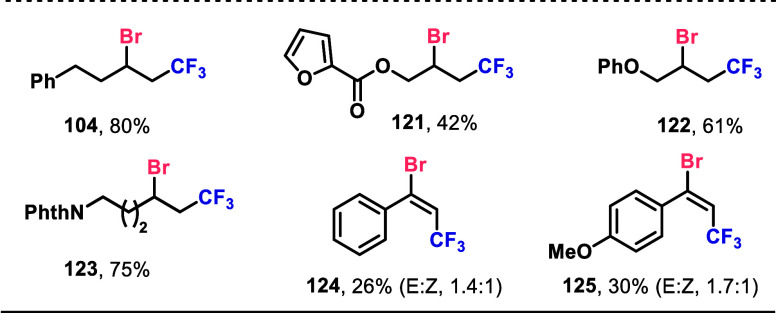
Scope of Bromotrifluoromethylation of Alkenes and Alkynes^a^

a Reaction conditions: Alkene or alkyne (1.0 equiv), **105** (1.5 equiv), CF_3_SO_2_Na (2.0 equiv), MeCN (0.1 M), AT, 12 h, 440 nm blue LED, in vials; isolated yields are reported. The dr ratio was determined based on the ^1^H NMR analysis of the crude reaction mixture and GC. See the Supporting Information for details.

## Conclusion

In summary, we have established a practical method for efficiently inducing visible-light-mediated intermolecular and intramolecular fluoroalkyl-sulfonylalkylation of alkenes and alkynes. Mechanistic studies reveal that the reaction proceeds through a sequential process involving the addition of fluoroalkyl radicals, the incorporation of SO_2_, and subsequent S_N_2-type reactions. This approach demonstrates a wide range of applicability, producing a variety of acyclic sulfones, cyclic γ-sultines, and cyclic sulfones with fluoroalkyl groups, predominantly with high stereoselectivity and regioselectivity. The generality of this protocol is further evident by its capacity to perform iodo- and bromo-trifluoromethylation under the same reaction condition using structurally distinct precursors, iodoesters, and bromoacetyl bromides.

## Supplementary Material



## Notes

Experimental procedures and characterization data for all compounds. CCDC numbers 2419235, 2419238, 2419234, and 2419232 for compounds **21**, **40**, **48**, and **56** contain the supplementary crystallographic data for this paper (PDF)
