# Supplementary material for: Enabling Fluoroalkyl-Sulfonylalkylation and Fluoroalkyl-Halogenation of Alkenes and Alkynes via Photoredox Catalysis
Source: J Am Chem Soc. 2026 Apr 17;148(16):16882–93. doi: 10.1021/jacs.6c00567 (PMC13135463; doi:10.1021/jacs.6c00567)

Enabling Fluoroalkyl-Sulfonylalkylation and Fluoroalkyl-Halogenation of Alkenes and Alkynes via Photoredox Catalysis

Supuni I. N. Hewa Inaththappulige, Ayush Acharya, Nipuna D. D. Z. Agampodi, Harshvardhan Singh, Ramesh Giri*

Department of Chemistry, The Pennsylvania State University, University Park, Pennsylvania 16802, United States.

*Corresponding author. Email: rkg5374@psu.edu

Contents

1.0 General Information 2

2.0 Experimental Section 3

2.1 Reaction setup – 0.1 mmol scale reactions 3

2.2 Reaction setup – 0.5 mmol scale gas reactions 3

2.3 Reaction optimization and control experiments 4

2.4 Starting material synthesis 11

2.5 General optimized method of fluoroalkyl sulfonylation alkenes and alkynes 12

2.6 General optimized method of fluoroalkyl sulfonylation gaseous alkenes and alkynes 12

2.7 General procedure for large scale reaction 14

2.8 Sequential two-pot preparation of fluoroalkyl sulfonylation 14

3.0 Mechanistic Studies 19

3.1 Radical clock experiments 19

3.2 Radical trap experiment[5] 21

3.3 Observation of radical dimer 23

3.4 Light- dark experiment 24

3.5 Quantum yield 25

3.6 Stern-Volmer quenching experiment 31

3.7 General optimized method of iodotriflouromethylation alkenes and alkynes 35

3.8 Competition study between α-halo esters 35

3.9 General optimized method of bromotriflouromethylation alkenes and alkynes 37

4.0 X-ray Crystal of Compounds 40

5.0 Experimental Data for Flouroalkyl Sulfonylation Compounds: 72

6.0 References 116
7.0 Spectra 117

1.0 General Information

All the reactions were set up in nitrogen environment, exposed to Kessil lamps, model PR160L 440 nm LED (maximum 45W) irradiation and cooled by a compact fan unless stated otherwise. In scale-up trials, 6-dram screw-top glass vials were positioned 1cm away from the light source without any spectral filtration. All the glassware including the 4-dram, 1-dram borosilicate (Kimble-Chase) vials were properly dried in an oven before use. Bulk solvents were obtained from Fisher, anhydrous solvents (DMF, DMSO, NMP, dioxane, toluene, dichloroethane, THF, MeCN) were obtained from Sigma-Aldrich and were used directly without further purification. Deuterated solvents were purchased from Cambridge Isotope. ^{1}H, ^{13}C, and ^{19}F spectra were recorded on a Bruker instrument (400, 101, and 376 MHz respectively) and internally referenced to the residual solvent signals of $(CD_3)_2CO$ at δ 2.05 ppm for ^{1}H, 206.26 ppm for ^{13}C NMR and $CDCl_3$ at δ 7.26 ppm for ^{1}H, 77.0 ppm for ^{13}C NMR. The chemical shifts of NMR and the coupling constants (*J*) for ^{1}H, ^{13}C and ^{19}F NMR are reported in δ parts per millions (ppm) and in Hertz, respectively. The following conventions are used for multiplicities: s, singlet; d, doublet; t, triplet; q, quartet; m, multiples. High resolution masses of new compounds were recorded at the Mass Spectrometry Facility at Department of Chemistry of Indiana University. HRMS analyses were conducted employing a time-of-flight (TOF) mass spectrometer equipped with an electrospray ionization (ESI) source FT-IR spectra were obtained using Bruker V70 spectrometer with ZnSe crystal. All NMR spectra were collected at Department of Chemistry, Pennsylvania State University, PSU. While UV spectra were recorded in Shimadzu UV-2600i spectrophotometer fluorescence was studied using Shimadzu RF-6000 Spectro fluorophotometer. X-ray data were measured on a

Bruker Kappa APEXII Duo system equippPennsylvaniaec Microfocus IμS (Cu Kα, λ = 1.54178 Å) and a multi-layer mirror monochromator at the X-ray facility of The Pennsylvania State University.

2.0 Experimental Section

2.1 Reaction setup – 0.1 mmol scale reactions

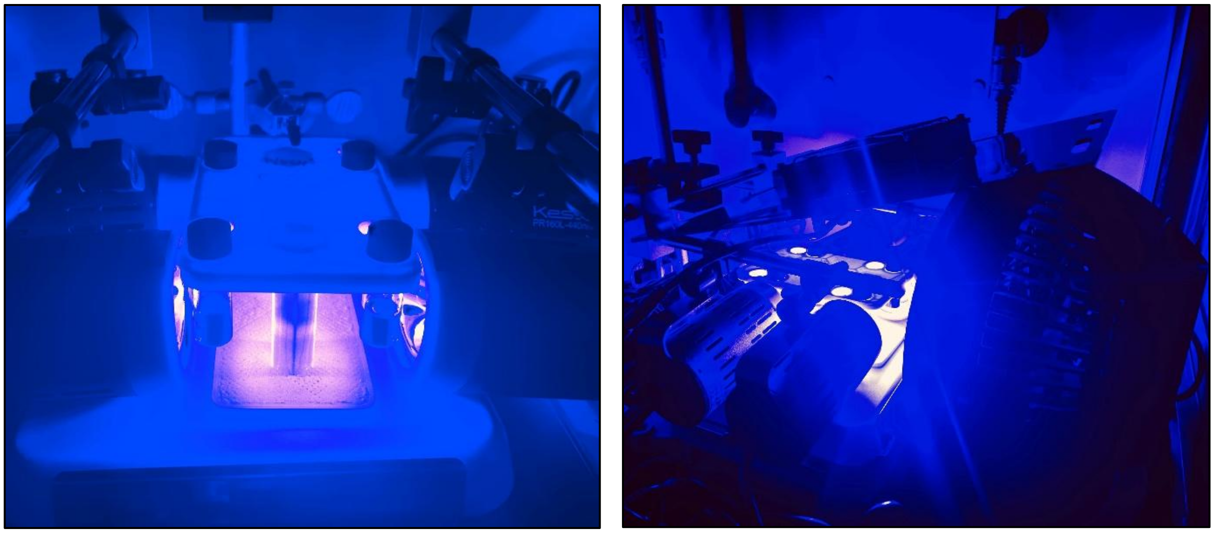

Supplementary Figure 01: 0.1 mmol scale reaction under irradiation with 440 nm blue LEDs

2.2 Reaction setup – 0.5 mmol scale gas reactions

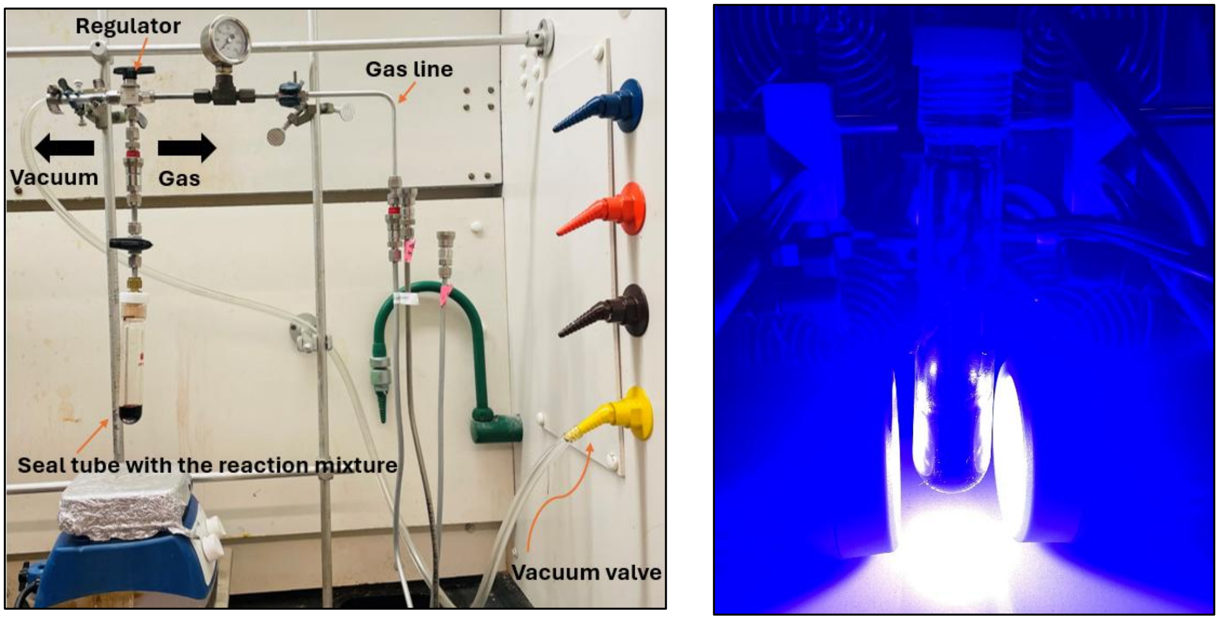

Supplementary Figure 02: 0.1 mmol scale reaction under irradiation with 440 nm blue LEDs

Two Kessil lamps were employed to irradiate gas reactions in seal tubes, based on the assumption that the significant thickness of the seal tubes would hinder the penetration of visible light at 440 nm.

2.3 Reaction optimization and control experiments

An oven-dried 1-dram vial was charged with a magnetic stir bar and photocatalyst inside the glove box. 4-Phenyl-1-butene (0.1 mmol, 1 eq), methyl 2-bromoacetate (0.15 mmol, 1.5 eq), CF_3SO_2Na salt (0.2 mmol, 2 eq) and anhydrous MeCN (0.5 mL) were added to the vial. The reaction tube was placed at a distance of ~1 cm away from 440 nm KESSIL blue LEDs (36 W operating at 100% intensity 440 nm blue LED lamps were then irradiated with stirring at ambient temperature. After 6 hours, the reaction mixture concentrated in vacuo and check the NMR yield by using the 1,1,2,2-tetrachloroethane as NMR standard.

Supplementary Table 1. Control experiments.

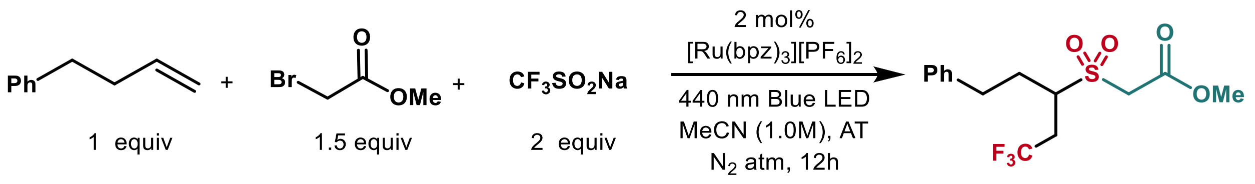

1 2 3

Deviation from the standard condition	Yield% (3)
Without 440 nm blue LEDs	0%
Without photocatalyst	0%
Reaction at ambient light	0%
Reaction at dark	0%

Supplementary Table 2. Evaluation of photocatalysts.

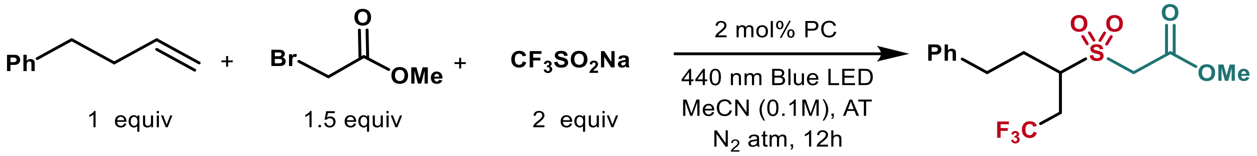

1 2 3

Photocatalysts	Yield% (3)
**Eosin Y sodium**	trace
**Fluorescein**	0%
**Ir(dtbbpy)(ppy)$_2$PF$_6$**	0%
**Ir(dFCF$_3$ppy)$_2$(bpy)PF$_6$**	0%
**4CzIPN**	trace
**Ru(bpy)$_3$(PF$_6$)$_2$**	51%
**Ru(bpz)$_3$(PF$_6$)$_2$**	75%
fac*-Ir(ppy)$_3$	0%
**9-Mesityl-10-phenylacridinium tetrafluoroborate (MPAT)**	55%
**2,4,6-Triphenylpyrilium tetrafluoroborate (TPT)**	0%
**Anthracene-9,10-dicarbonitrile**	0%
**Eosin Y**	0%
**[Ir(dFCF$_3$ppy)$_2$]$_2$**	0%
**Rhodamine B**	0%
**N,N'-Dimethyl perylenediimide (PDI)**	0%

*carbobrominated product was observed in 52% NMR yield.

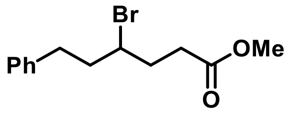

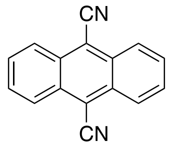

Anthracene-9,10-dicarbonitrile

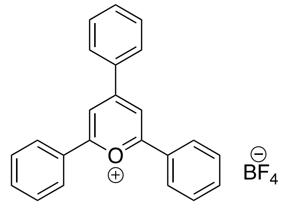

2,4,6-Tripehnylpyrilium tetrafluoroborate (TPT)

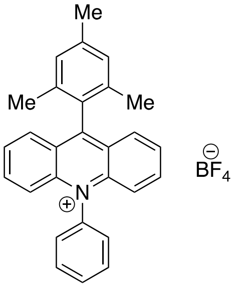

9-Mesityl-10-phenylacridinium tetrafluoroborate (MPAT)

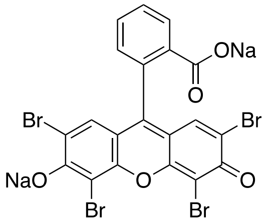

Eosin Y sodium

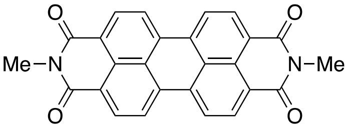

N,N'-Dimethyl perylenediimide (PDI)

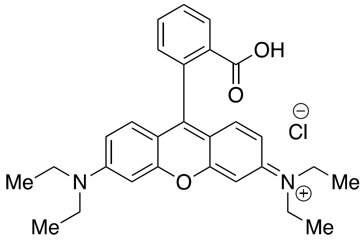

Rhodamine B

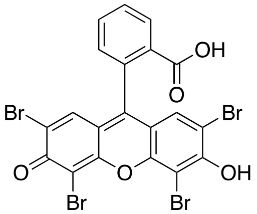

Eosin Y

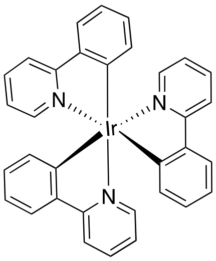

$Ir(ppy)_3$

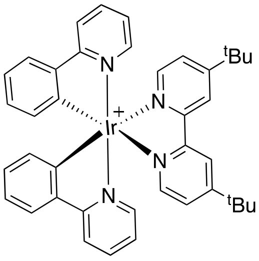

$Ir(dtbbpy)(ppy)_2PF_6$

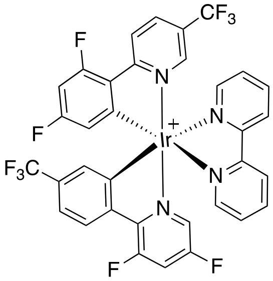

$Ir(dFCF_3ppy)_2(bpy)PF_6$

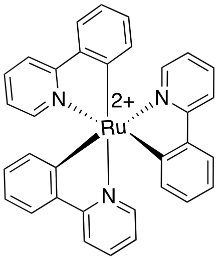

$Ru(bpy)_3(PF_6)_2$

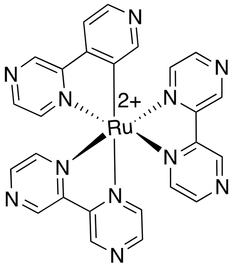

$Ru(bpz)_3(PF_6)_2$

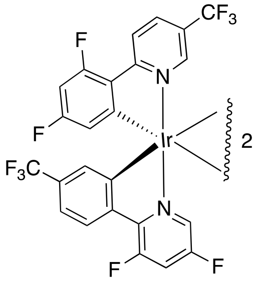

$[Ir(dFCF_3ppy)_2]_2$

Supplementary Figure 6. Different photocatalysts were used in this study.

Supplementary Table 3. Evaluation of equivalent of Methyl bromo acetate.

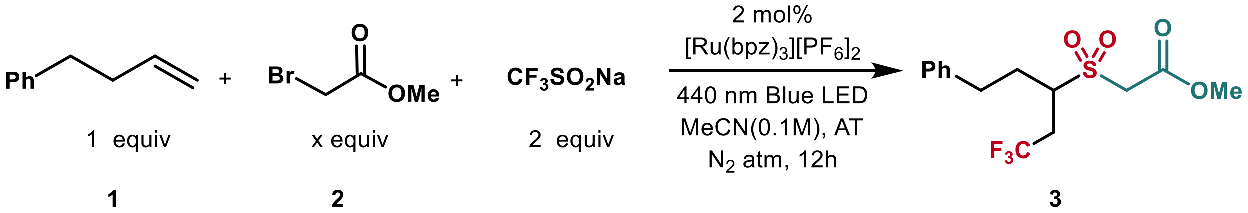

Methyl Bromo Acetate	Yield% (3)
**1 eq.**	61%
**1.5 eq.**	76%
**3 eq.**	71%
**4 eq.**	75%

Supplementary Table 4. Evaluation of equivalent of fluoroalkyl salt.

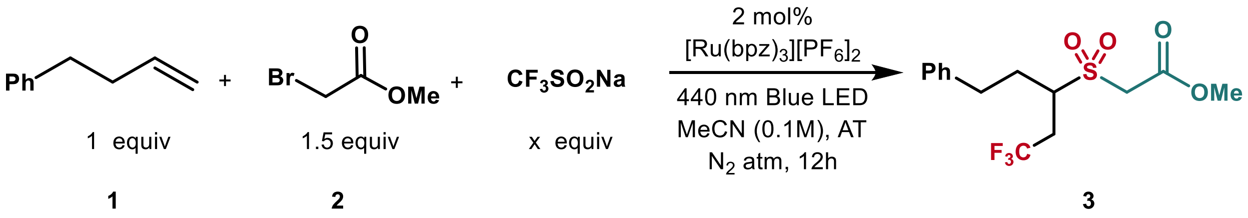

CF_3SO_2Na	Yield% (3)
**1.0 eq.**	49%
**1.5 eq.**	61%
**2.0 eq.**	72%
**3.0 eq.**	66%

Supplementary Table 5. Evaluation of catalyst loading.

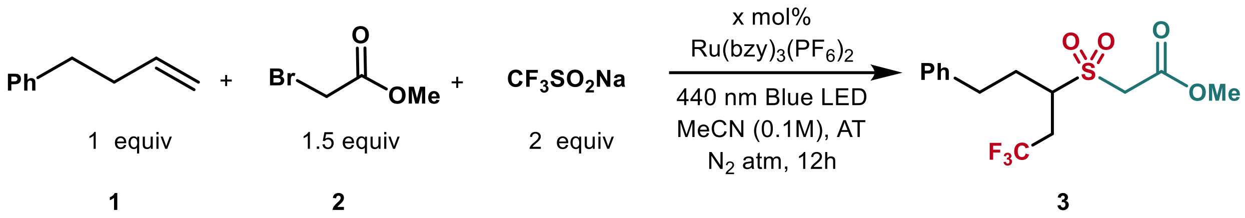

Ru(bpz)$_3$(PF$_6$)$_2$	Yield% (3)
**0.5 mol%**	26%
**1.0 mol%**	44%
**2.0 mol%**	75%
**3.0 mol%**	72%

Supplementary Table 5. Evaluation of different solvents.

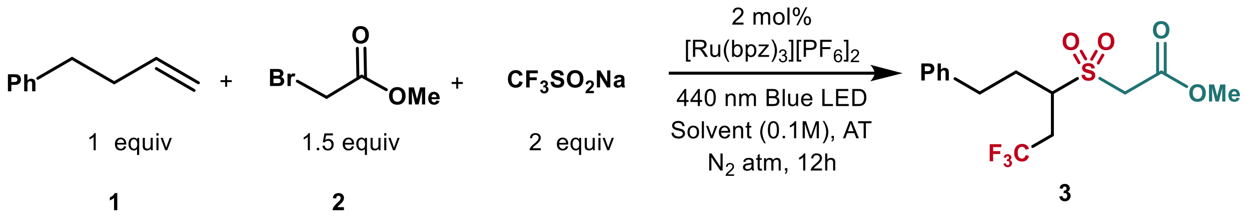

Solvents	Yield% (3)
**1,4-Dioxane**	0%
**Toluene**	0%
**Dimethoxyethane**	0%
**DMF**	trace
**Dichloromethane (DCM)**	0%

**Acetonitrile**	75%
**Dimethyl sulfoxide (DMSO)**	0%
**N-Methyl-2-pyrrolidone (NMP)**	trace
**Tetrahydrofuran (THF)**	0%
**Dichloroethane (DCE)**	0%
**Dimethyl acetamide (DMA)**	trace

Supplementary Table 6. Evaluation of dilution of the solvent.

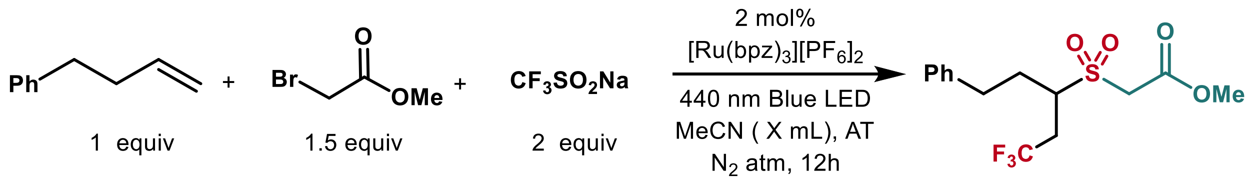

1 2 3

MeCN volume/ mL	Yield% (3)
**0.5**	76%
**1.0**	77%
**1.5**	74%
**2.0**	68%

Supplementary Table 7. Evaluation of Reaction time

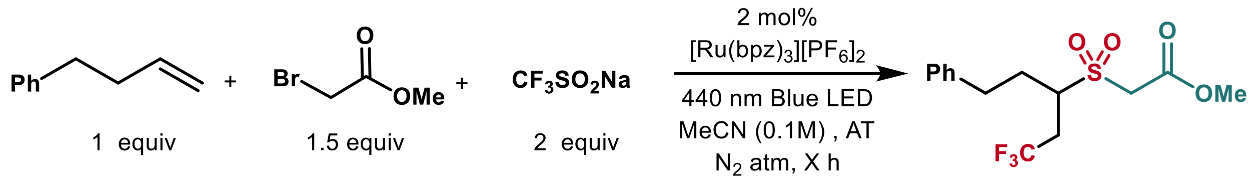

1 2 3

Time	Yield% (3)
30 min	60%
1 hours	64%
2 hours	68%
3 hours	76%
4 Hours	70%
12 hours	75%

Supplementary Table 8. Reactivity profile study of I, Br, Cl and F alkyl reagents

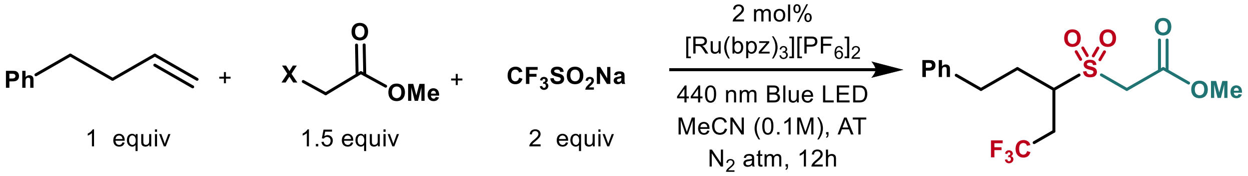

1 2 3

Halo alkyl reagent	Yield% (3)
F	0%
Cl	31%
Br	74%
I	0%

Supplementary Table 8. Evaluation of various sulfinate salt

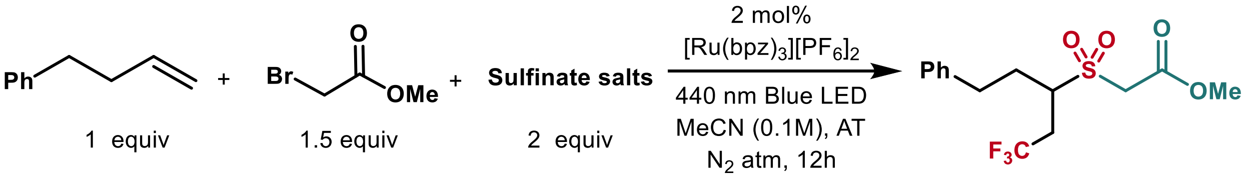

1 2 3

Sulfinate salt	Yield% (3)
**$(CF_3SO_2)_2Zn$**	0%
**Umemoto reagent**	0%

Supplementary Table 9. Evaluation of different LED lights

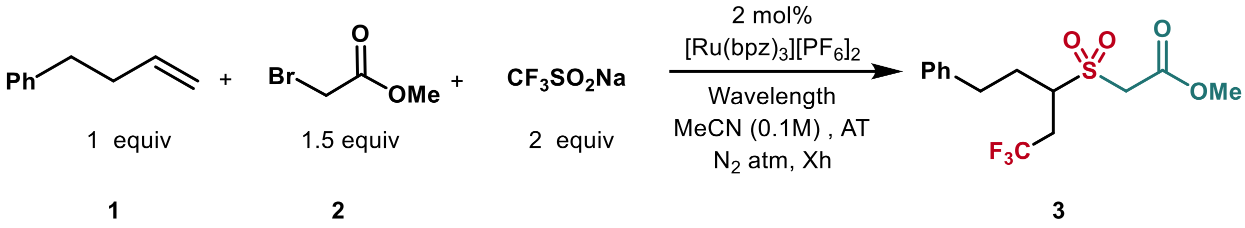

Wavelength	Yield% (3)
**390 nm**	38%
**440 nm**	76%
**467 nm**	57%

2.4 Starting material synthesis

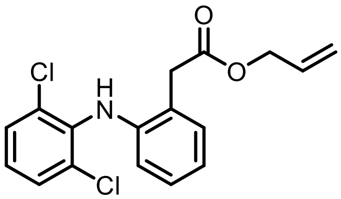

Diclofenac (1equiv.) and K_2CO_3 (3 equiv.) in anhydrous DMF were stirred at rt for 30 min. Allyl bromide (2 eq) was added and the reaction mixture was heated to 60 °C. After 20 hours, the reaction mixture was put into water, extracted by EtOAc (3x), washed with water (5x), dried over anhydrous Na_2SO_4, and concentrated in vacuo. The crude was purified by silica gel column chromatography.

^{1}H NMR (400 MHz, CDCl3): δ 7.34 (d, J = 8.1 Hz, 2H), 7.27 – 7.23 (m, 1H), 7.13 (td, J = 7.7, 1.6 Hz, 1H), 7.03 – 6.86 (m, 3H), 6.56 (d, J = 8.0 Hz, 1H), 5.94 (ddt, J = 16.4, 11.0, 5.8 Hz, 1H), 5.40 – 5.14 (m, 2H), 4.65 (dd, J = 5.8, 1.5 Hz, 2H), 3.85 (s, 2H); **^{13}C NMR (101 MHz, $CDCl_3$):**

δ171.9, 142.7, 137.8, 131.8, 130.9, 129.5, 128.8, 128.0, 124.2, 124.0, 122.0, 118.7, 118.3, 65.9, 38.6. **IR (neat, ν_{max}):** 3319, 1720, 1450, 1143, 769 cm^{-1}. **HRMS-ESI** (*m/z*): Calcd. for $C_{17}H_{16}F_3Cl_2NO_4$ $[M+H]^+$ 336.0553, found 336.0552.

2.5 General optimized method of fluoroalkyl sulfonylation alkenes and alkynes

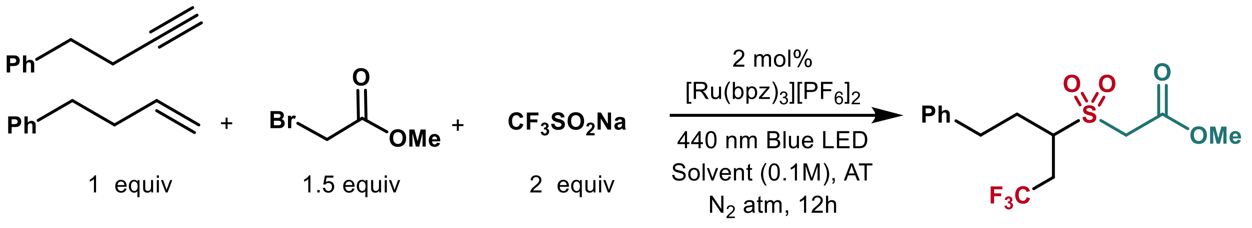

An oven-dried 6-dram vial was charged with a magnetic stir bar and photocatalyst $[Ru(bpz)]_3[PF_6]_2$ (2 mol%, 8.7 mg) inside the glove box. Corresponding alkene/ alkyne (0.5 mmol, 1 equiv.), sulfinate salt (1.0 mmol, 2 equiv.), alkyl bromide (0.75 mmol, 1.5 equiv.), and anhydrous MeCN (5.0 mL) were added to the vial. The resulting mixture was then irradiated with 440nm Blue LED. After 12 hours, the reaction mixture was concentrated in vacuo and check the NMR yield by using the 1,1,2,2-tetrachloroethane as NMR standard. The crude was purified by silica gel column chromatography using hexane – EtOAc as a mobile phase.

2.6 General optimized method of fluoroalkyl sulfonylation gaseous alkenes and alkynes

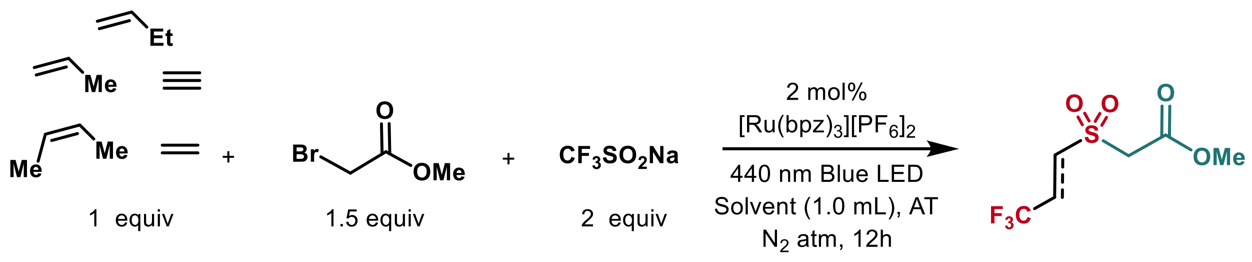

An oven-dried 20 mL seal tube was charged with a magnetic stir bar and photocatalyst [Ru(bpz)]$_3$[PF6]$_2$, (2 mol%, 8.7 mg) inside the glove box. Sulfinate salt (1.0 mmol, 2 equiv.), alkyl bromide (0.5 mmol, 1.0 equiv.), and anhydrous MeCN (5.0 mL) were added to the vial. The seal tube was closed using a cap connected with a regulator as shown in **figure S2**. The seal tube was then connected to the gas reaction setup through tubing, allowing gas flow between the vial and the setup. The entire system, including both the seal tube, is evacuated using a vacuum pump to remove air (N_2). Once the desired vacuum is achieved, the system is flushed with gaseous alkene to replace the evacuated air, creating an inert or specific gaseous environment. The pressure inside the seal tube can vary depending on the gaseous alkene used (table xx). The resulting mixture was then irradiated with two 440nm Blue LED (maximum 45W). After 12 hours, the reaction mixture was concentrated in vacuo and checked the NMR yield by using the 1,1,2,2-tetrachloroethane as NMR standard. The crude was purified by silica gel column chromatography using hexane – EtOAc as a mobile phase.

Supplementary Table 10. Gaseous alkenes and their corresponding pressure used

Gas	Pressure/ atm
**Ethylene**	50
**Propylene**	50
**1-Butene**	25
**Cis-2-butene**	50
**Acetylene**	7

2.7 General procedure for large scale reaction

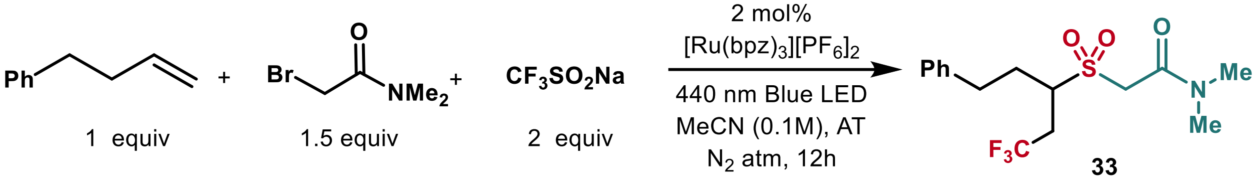

An oven-dried 100 mL round bottom flask was charged with a magnetic stir bar and photocatalyst $[Ru(bpz)]_3[PF6]_2$ (2 mol%, 21.8 mg) inside the glove box. Corresponding alkene/ alkyne (5 mmol, 1 equiv.), sulfinate salt (10.0 mmol, 2 equiv.), alkyl bromide (7.5 mmol, 1.5 equiv.), and anhydrous MeCN (50.0 mL) were added to the vial. The resulting mixture was then irradiated with two 440nm Blue LED kessil lamps. After 12 hours, the reaction mixture was concentrated in vacuo. The crude was purified by silica gel column chromatography using hexane – EtOAc as a mobile phase.

2.8 Sequential two-pot preparation of fluoroalkyl sulfonylation

2.8.1 Sequential one-pot preparation of fluoroalkyl sulfonyl-alkylation from simple alkyl bromides[1]

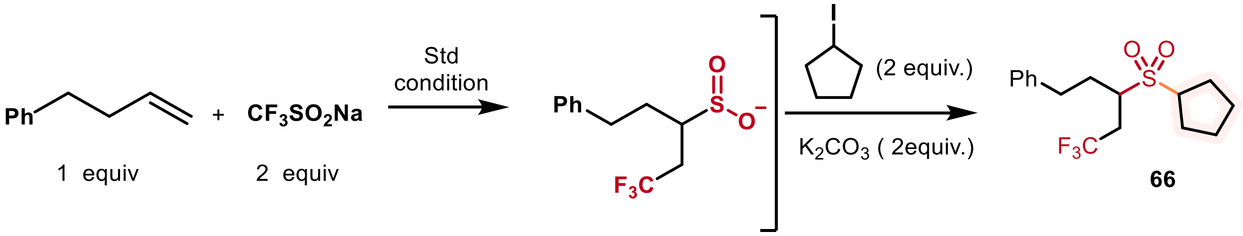

An oven-dried 6-dram vial was charged with a magnetic stir bar and photocatalyst $[Ru(bpz)]_3[PF_6]_2$ (2 mol%, 8.7 mg) inside the glove box. Corresponding alkene (0.5 mmol, 1 equiv.), sulfinate salt (1.0 mmol, 2 equiv.), and anhydrous MeCN (5.0 mL) were added to the vial. The resulting mixture was then exposed to a 440 nm blue LED for 12 hours. After irradiation, potassium carbonate (2.0 mmol, 4 equiv.) and the corresponding alkyl iodide (1.0 mmol, 2 equiv.)

were added to the same vial, and stirring was continued for an additional 6 hours at 60 °C. The reaction mixture concentrated in vacuo and check the NMR yield by using the 1,1,2,2-tetrachloroethane as NMR standard. The crude was purified by silica gel column chromatography using hexane – EtOAc as a mobile phase.

2.8.2 Sequential two-pot preparation of fluoroalkyl sulfonyl-arylation[2]

An oven-dried 6-dram vial was charged with a magnetic stir bar and photocatalyst $[Ru(bpz)]_3[PF_6]_2$ (2 mol%, 8.7 mg) inside the glove box. Corresponding alkene (0.5 mmol, 1 equiv.), sulfinate salt (1.0 mmol, 2 equiv.), and anhydrous MeCN (5 mL) were added to the vial. The resulting mixture was then exposed to a 440 nm blue LED for 12 hours. After irradiation, the solvent was removed by evaporation.

Method A:

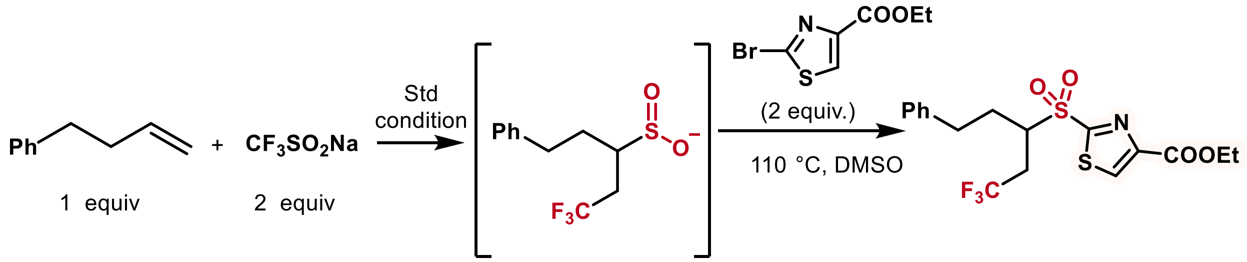

DMSO (5 mL) was added to the same vial to dissolve crude along with ethyl 2-bromothiazole-4-carboxylate (1.0 mmol, 2 equiv.) and was stirred at the 110 °C temperature for 12 h. The mixture was then transferred to a separatory funnel, diluted with water (10 mL) and extracted with EtOAc (2x3mL). The combined extracts were washed with brine, dried over $NaSO_4$, and concentrated to check the NMR yield by using the 1,1,2,2-tetrachloroethane as NMR standard. The crude was purified by silica gel column chromatography using hexane – EtOAc as a mobile phase.

Method B:

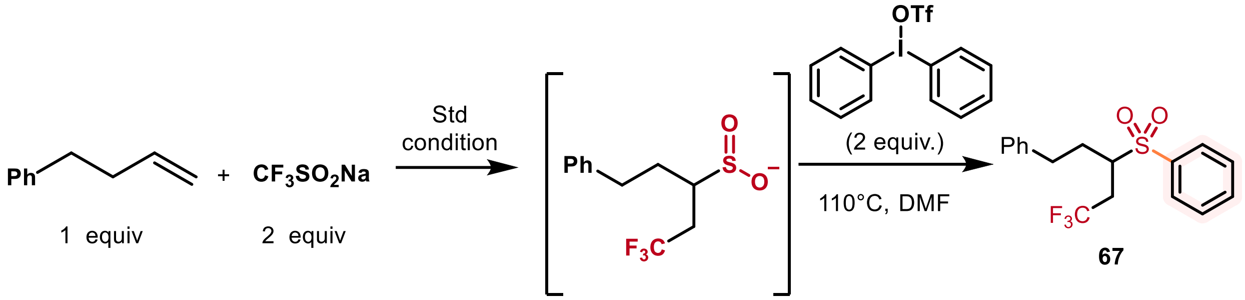

DMF (5 mL) was added to the same vial to dissolve crude along with diphenyliodonium trifluoromethanesulfonate (1.0 mmol, 2 equiv.) and was stirred at the 110 °C temperature for 12 h. The mixture was then transferred to a separatory funnel, diluted with water (10 mL) and extracted with EtOAc (2x3mL). The combined extracts were washed with brine, dried over $NaSO_4$, and concentrated to check the NMR yield by using the 1,1,2,2-tetrachloroethane as NMR standard. The crude was purified by silica gel column chromatography using hexane – EtOAc as a mobile phase.

2.8.3 Sequential two-pot preparation of fluoroalkyl sulfonamide

An oven-dried 6-dram vial was charged with a magnetic stir bar and photocatalyst $[Ru(bpz)]_3[PF_6]_2$ (2 mol%, 8.7 mg) inside the glove box. Corresponding alkene (0.5 mmol, 1 equiv.), sulfinate salt (1.0 mmol, 2 equiv.), and anhydrous MeCN (5 mL) were added to the vial and irradiated with a 440 nm blue LED for 12 hours. After irradiation, the solvent was removed by evaporation.

Method A[3]:

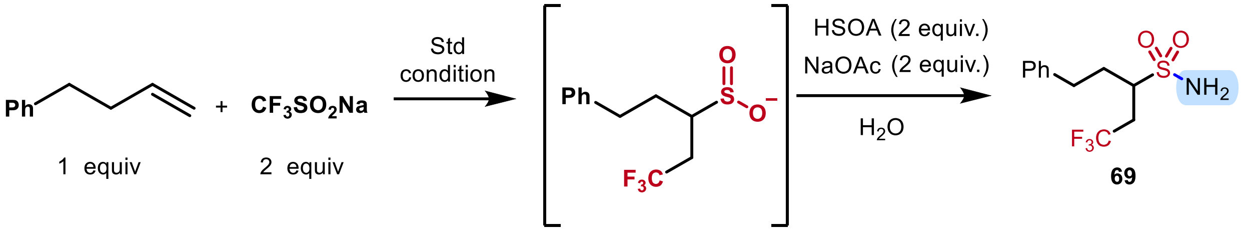

69

A solution of hydroxylamine-O-sulfonic acid (2.0 mmol, 4.0 equiv.) and sodium acetate (3.5 mmol, 7.0 equiv.) in water (5 mL) was added to the same vial dropwise under stirring and the resulting suspension was further stirred at the room temperature for 12 h. The mixture was extracted with EtOAc (2x3mL). The combined extracts were washed with brine, dried over $NaSO_4$, and concentrated to check the NMR yield by using the 1,1,2,2-tetrachloroethane as NMR standard. The crude was purified by silica gel column chromatography using hexane – EtOAc as a mobile phase.

Method B[2]:

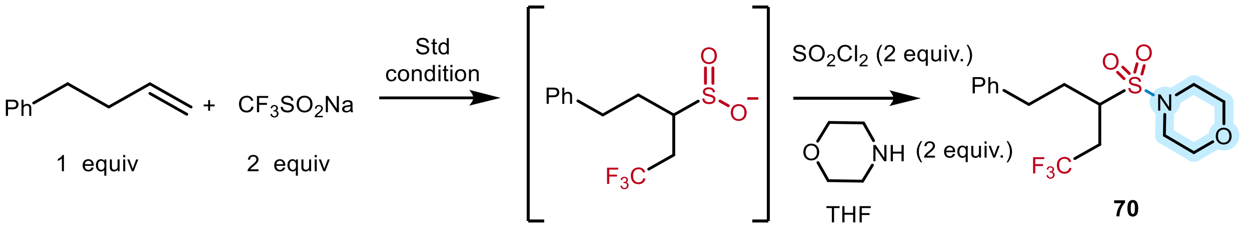

70

THF (5 mL) was added to the same vial to dissolve crude. The resultant suspension cooled to 0°C and sulfuryl chloride (2 equiv.) added dropwise. Corresponding amine (2 equiv.) Was then added dropwise while stirring and the resulting suspension was further stirred at the room temperature for 6 h. Upon completion, the reaction mixture was poured onto NH_4Cl (20 mL) and extracted with DCM (3 x 20 mL). The combined organic fractions were dried ($MgSO_4$), filtered and concentrated in vacuo to check the NMR yield by using the 1,1,2,2-tetrachloroethane as NMR standard. The crude was purified by silica gel column chromatography using hexane – EtOAc as a mobile phase.

2.8.4 Sequential two-pot preparation of fluoroalkyl sulfonyl fluorides[1]

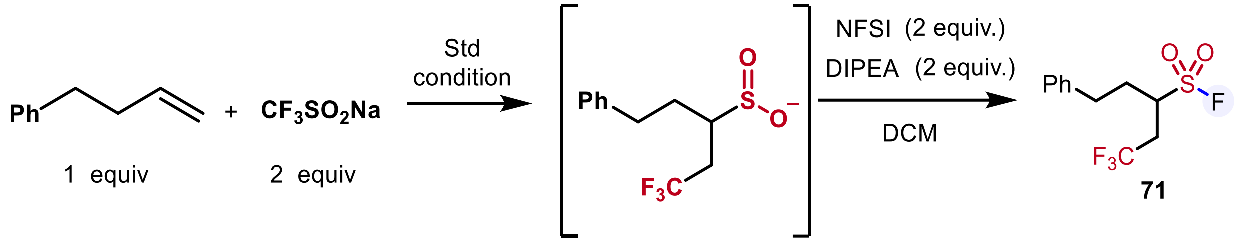

An oven-dried 6-dram vial was charged with a magnetic stir bar and photocatalyst [Ru(bpz)]3[PF6]2 (2 mol%, 8.7 mg) inside the glove box. Corresponding alkene (0.5 mmol, 1 equiv.), sulfinate salt (1.0 mmol, 2 equiv.), and anhydrous MeCN (5 mL) were added to the vial. The resulting mixture was then exposed to a 440 nm blue LED for 12 hours. After irradiation, solvent was removed by evaporation. DCM (5 mL) was added to the same vial to dissolve crude and DIPEA (0.175 mL,1.0 mmol, 2.0 equiv.) and the NFSI (2 equiv.) were added dropwise under stirring and the resulting suspension was stirred at the room temperature for 12 h. The mixture was concentrated to check the NMR yield by using the 1,1,2,2-tetrachloroethane as NMR standard. The crude was purified by silica gel column chromatography using hexane – EtOAc as a mobile phase.

2.8.5 Sequential two-pot preparation of fluoroalkyl sulfonylchlorination[2]

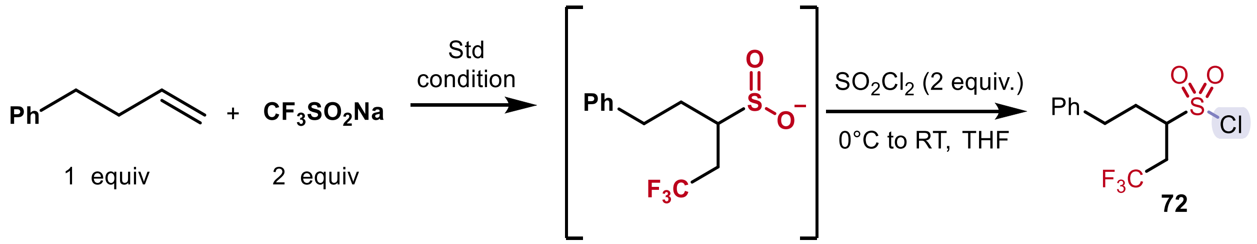

An oven-dried 6-dram vial was charged with a magnetic stir bar and photocatalyst [Ru(bpz)]3[PF6]2 (2 mol%, 8.7 mg) inside the glove box. Corresponding alkene (0.5 mmol, 1 equiv.), sulfinate salt (1.0 mmol, 2 equiv.), and anhydrous MeCN (5 mL) were added to the vial. The resulting mixture was then exposed to a 440 nm blue LED for 12 hours. After irradiation, the solvent was removed by evaporation. THF (5 mL) was added to the same vial to dissolve crude.

The resultant suspension cooled to 0°C and sulfuryl chloride (2 equiv.) added dropwise. Upon completion, the reaction mixture was poured onto NH_4Cl (20 mL) and extracted with DCM (3 x 20 mL). The combined organic fractions were dried ($MgSO_4$), filtered and concentrated in vacuo to check the NMR yield by using the 1,1,2,2-tetrachloroethane as NMR standard. The crude was purified by silica gel column chromatography using hexane – EtOAc as a mobile phase.

3.0 Mechanistic Studies

3.1 Radical clock experiments

3.1.1 Ring-opening radical clock experiments

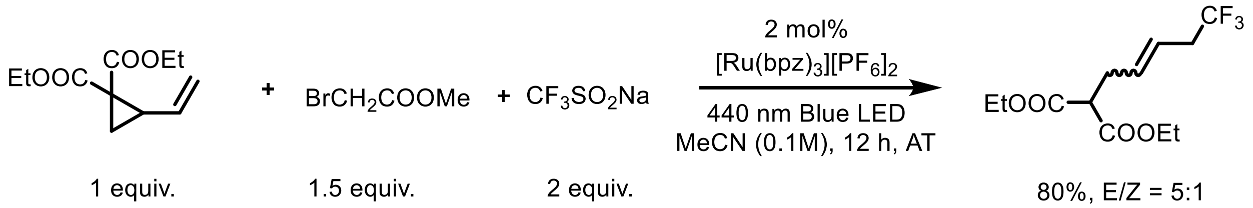

90

Diethyl 2-(5,5,5-trifluoropent-2-en-1-yl) malonate (90)[4]: Following the general method of fluoroalkyl sulfonylalkylation 2.4, title compound **90** was obtained as a transparent oil (113.0 mg, 80% yield) after purification by silica gel flash chromatography (hexanes:EtOAc = 20:1 to 1:1). The reaction mixture was irradiated for 12h.

^{1}H NMR (400 MHz, $CDCl_3$):) δ 5.77 – 5.62 (m, 1H), 5.57 – 5.44 (m, 1H), 4.23 – 4.14 (m, 4H), 3.40 (t, J = 7.5 Hz, 1H), 2.98 – 2.83 (m, 0.36H), 2.83 – 2.68 (m, 1.66H), 2.69 – 2.57 (m, 2H), 1.26 (td, J = 7.1, 1.7 Hz, 6H); **^{13}C NMR (101 MHz, $CDCl_3$):** δ 168.66, 133.39, 131.39, 125.74 (q, $^1J_{CF}$ = 276.5 Hz), 121.02 (q, $^3J_{CF}$ = 3.5 Hz), 120.13 (d, $^1J_{CF}$ = 3.6 Hz). 61.57, 61.48, 51.52, 51.34, 37.19 (q, $^2J_{CF}$ = 29.8 Hz), 31.57, 13.98; **^{19}F NMR (376 MHz, CDCl3):** δ -66.6.

3.1.2 Ring-closing radical clock experiments

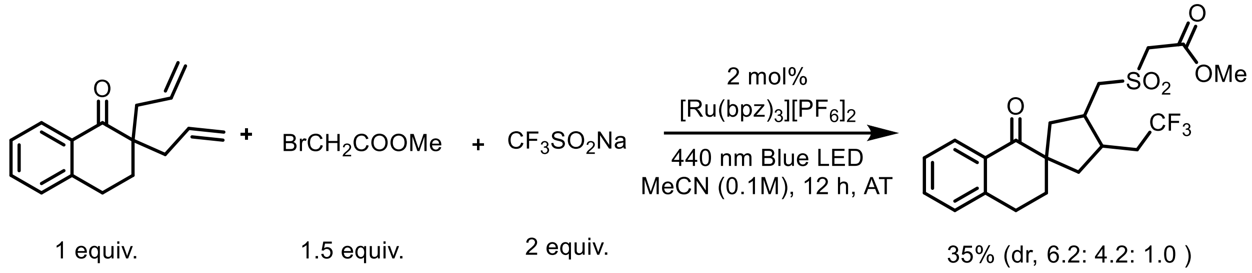

92

***Methyl-2-(((1'-oxo-3-(2,2,2-trifluoroethyl)-3',4'-dihydro-1'H-spiro[cyclopentane-1,2'-naphthalen]-4-yl)methyl)sulfonyl)acetate (92)*:** Following the general method of fluoroalkyl sulfonylalkylation 2.4, title compound **92** was obtained as a transparent oil (75.7 mg, 35% yield) after purification by silica gel flash chromatography (hexanes:EtOAc = 20:1 to 1:2). The reaction mixture was irradiated for 12h. Compound was isolated as a mixture of inseparable diastereomers in a 6.2: 4.2: 1.0 ratio as determined in a crude reaction mixture by ^{1}H NMR.

^{1}H NMR (400 MHz, $CDCl_3$): δ 8.06 – 7.98 (m, 1H), 7.51 – 7.41 (m, 1H), 7.30 (t, J = 7.6 Hz, 1H), 7.21 (d, J = 7.7 Hz, 1H), 4.05-3.94 (m, 2H), 3.84 (m, 1.86H), 3.82 (m, 0.77H), 3.81 (m, 0.36H), 3.57 – 3.49 (m, 0.73H), 3.45 (dd, J = 13.8, 4.6 Hz, 0.27H), 3.37 (dd, J = 13.7, 4.2 Hz, 0.64H), 3.21 – 3.12 (m, 0.33H), 3.11 – 2.92 (m, 2.37H), 2.92 – 2.81 (m, 0.77H), 2.78 – 2.67 (m, 0.29H), 2.63 – 2.49 (m, 1H), 2.42 (dd, J = 13.9, 5.4 Hz, 1.14H), 2.34 – 2.21 (m, 1.79H), 2.21 – 2.09 (m, 2.84H), 1.97 – 1.79 (m, 1.72H), 1.55 (ddd, J = 13.4, 10.0, 3.1 Hz, 0.37H); **^{13}C NMR (101 MHz, $CDCl_3$):** δ 201.4, 201.2, 200.7, 163.7, 163.5, 143.4, 143.3, 143.2, 133.5, 133.5, 133.4, 131.1, 130.7, 128.7, 128.6, 128.3, 128.3, 128.2, 128.1, 128.1, 126.8, 125.5, 58.5, 58.3, 57.8, 57.5, 57.1, 56.7, 54.0, 53.4, 52.7, 52.5, 52.2, 51.6, 51.3, 41.6, 39.9, 39.6, 39.5, 39.0, 38.9, 38.7, 37.9, 37.2, 36.9, 36.9, 36.8, 36.8, 36.8, 36.4, 36.3, 36.1, 36.1, 35.9, 35.8, 34.4, 34.1, 33.8, 33.5, 33.2, 32.9, 26.5, 26.4, 25.8. *The ^{13}C NMR is complex due to presence of three diastereomers containing CF_3 groups; therefore, it is difficult to assign the corresponding peaks of ^{13}C-attached to the fluorine. Hence, the complete list of the representative peaks of ^{13}C is shown*; **^{19}F NMR (376 MHz, $CDCl_3$):** δ -63.8, 64.1, -64.5;

IR (neat, ν_{max}): 2930, 1742, 1674, 1315, 1119, 841 cm^{-1}. **HRMS-ESI** (*m/z*): Calcd. For $C_{20}H_{22}F_3O_5S$ $[M-H]^-$ 431.1146, found 431.1144.

3.2 Radical trap experiment[5]

An oven-dried 6-dram vial was charged with a magnetic stir bar and photocatalyst $[Ru(bpz)]_3[PF_6]_2$ (2 mol%, 8.7 mg) inside the glove box. Corresponding alkene/ alkyne (0.5 mmol, 1 equiv.), sulfinate salt (1.0 mmol, 2 equiv.), alkyl bromide (0.75 mmol, 1.5 equiv.), TEMPO (2.0

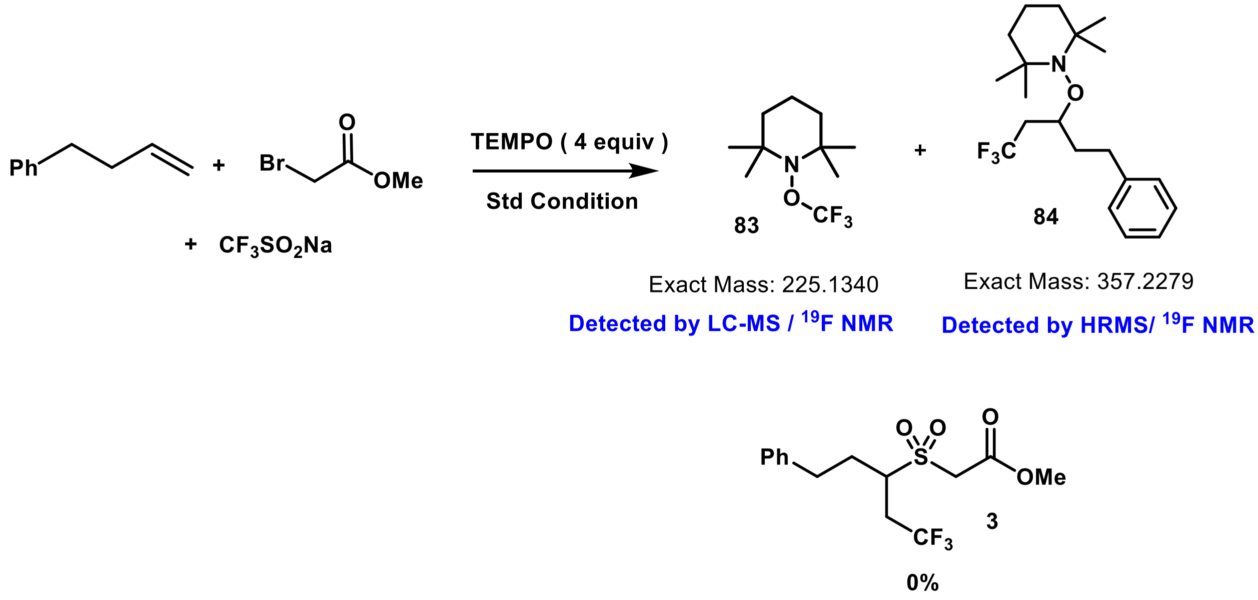

mmol, 4 equiv.) and anhydrous MeCN (5 mL) were added to the vial. The resulting mixture was then irradiated with 440nm Blue LED. After 12 hours, the reaction mixture was concentrated in vacuo and analyzed the F^{19} NMR and HRMS.

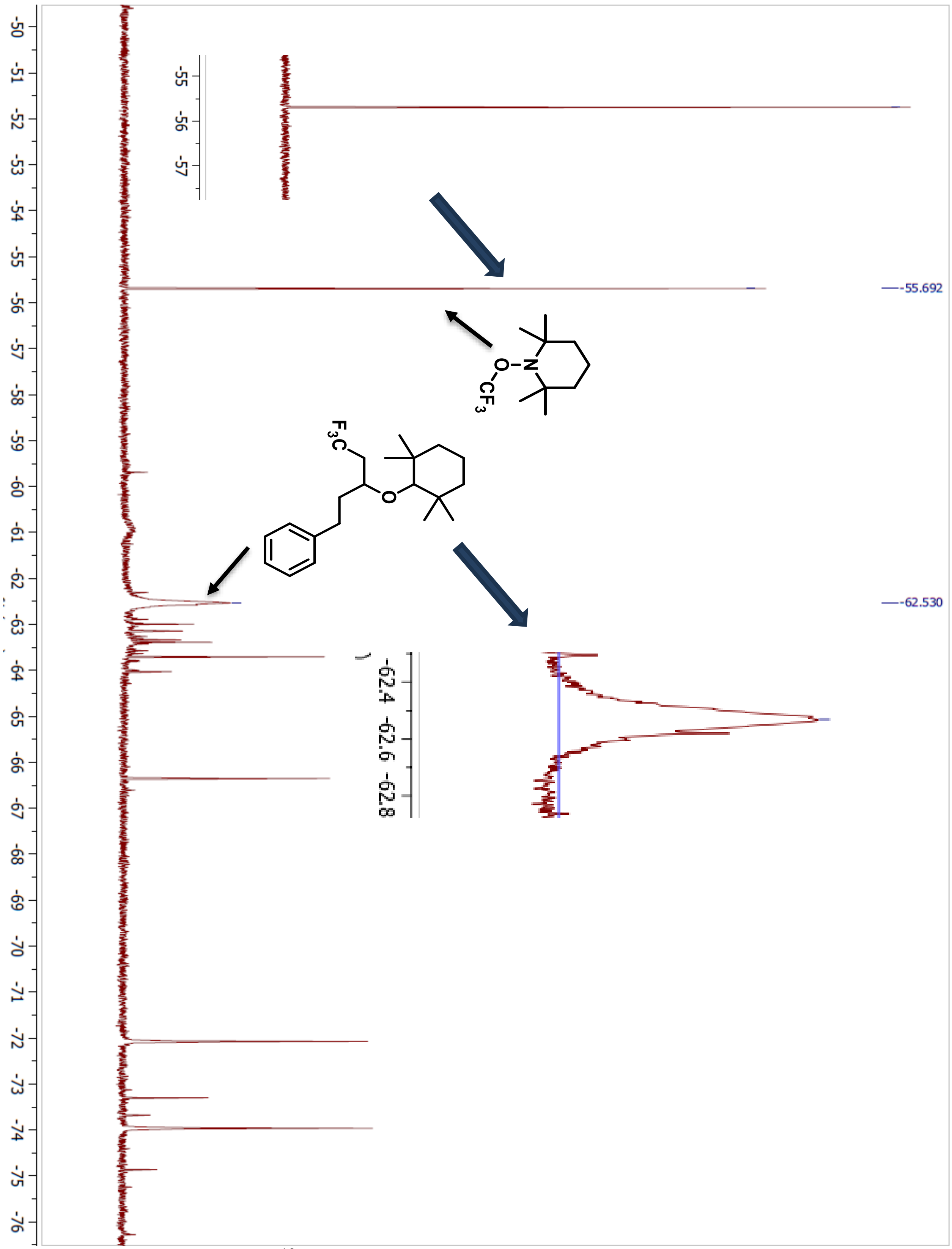

Supplementary Figure 04: ^{19}F NMR spectra for radical trap experiment

3.3 Observation of radical dimer

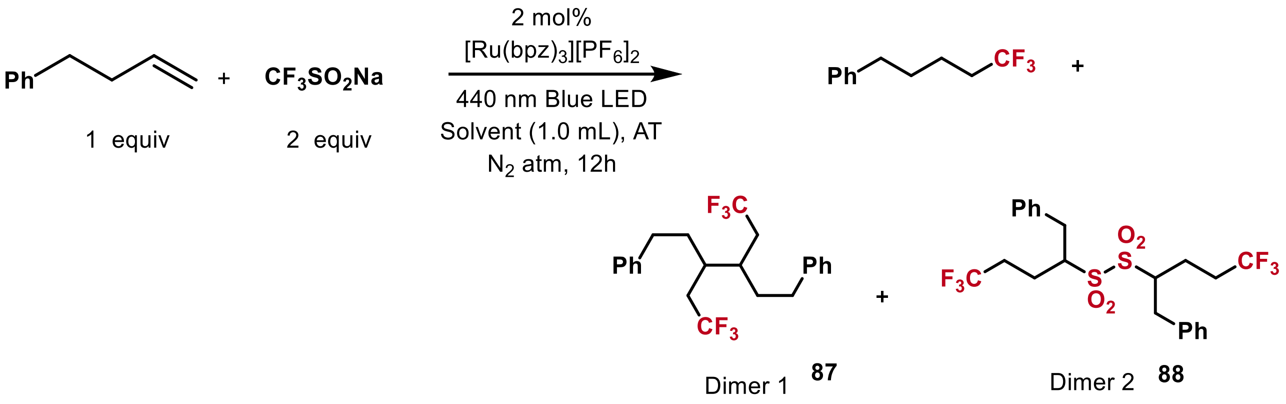

Following the general method of fluoroalkyl sulfonyl alkylation 2.4, title xx dimers were observed by GC-MS and HRMS.

Dimer	Calculated mass	Found
**Dimer 1 (87): $C_{22}H_{24}F_6$** 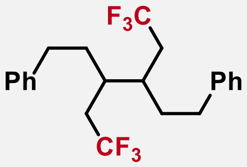	**$C_{22}H_{25}F_6$** 402.2	**$C_{22}H_{25}F_6$** 402.2
**Dimer 2 (88): $C_{22}H_{24}F_6O_4S_2$** 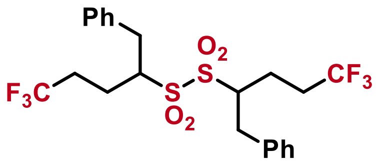	$[M+H]^+$: $C_{22}H_{25}F_6O_4S_2$ 531.1093	$[M+H]^+$: $C_{22}H_{25}F_6O_4S_2$ 531.1103

3.4 Light- dark experiment

On/off experiments were performed using a 0.2 mmol scale reaction (0.2M) under standard reaction condition. At different time intervals, 50μL aliquot of the reaction mixture was taken out via a syringe and analyzed by ^{1}H NMR. Yields were calculated using 1,1,2,2-tetrachloroethane as NMR standard. First, 50μL aliquot was syringed out from reaction mixture after irradiating the solution 10 min under 440 nm blue LED and the yield was calculated. Then, the blue LED was turned off and the reaction continued stirring for 10 min under dark. 50μL aliquot of the reaction solution syringed out and yield was calculated again. This process continued for 6.5 h and corresponding yields were recorded.

Reaction time	Yield %
**10 min ON**	5
**10 min OFF**	5
**15 min ON**	10
**15 min OFF**	10
**20 min ON**	15
**20 min OFF**	15
**30 min ON**	25
**30 min OFF**	25
**60 min ON**	43
**60 min OFF**	43
**120 min ON**	53

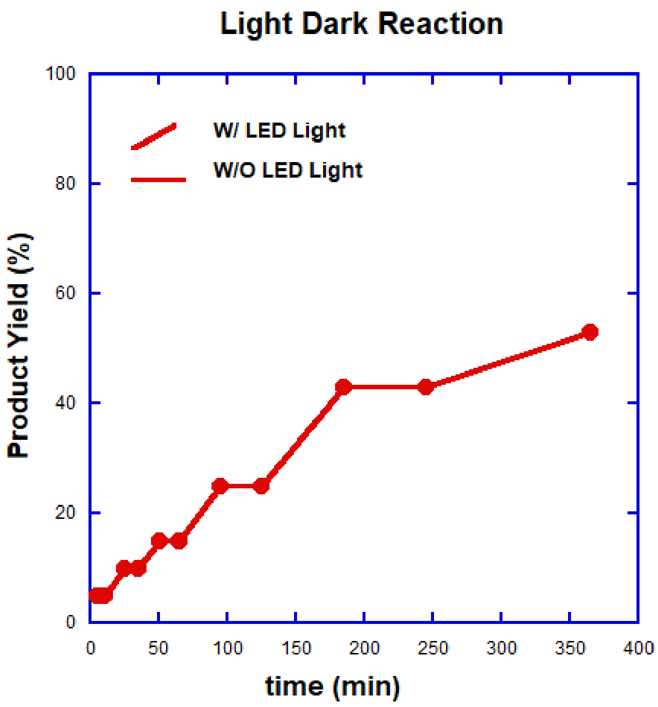

Supplementary Figure 05: Graphical representation of light-dark experiments.

3.5 Quantum yield

Quantum yield was determined according to the literature.[6]

Photon flux of blue LEDs used in fluoroalkyl-sulfonylalkylation was measured using established Hatchard Parker ferrioxalate actinometry[7, 8]. To address the high intensity of the light source, the procedure was modified slightly with an increased excess of 1,10-phenanthroline. To meet the prerequisite of actinometric determination of quantum yields, the light source used during these experiments were considered monochromatic. Unless stated otherwise, the optical path length of 1 cm was used in the experiments reported herein.

<u>Preparation of stock solutions:</u>

a. **Ferrioxalate solution (0.15 M ferrioxalate/0.05 M H_2SO_4):** In a 100 mL volumetric flask, was added $FeCl_3.6H_2O$ (4.05 g, 15 mmol) and 15 mL of water. After complete dissolution, $(COOK)_2.H_2O$ (8.29 g, 45 mmol) followed by 60 mL of water. The mixture was stirred for 1 h before adding 10 mL of 0.5 M H_2SO_4. Finally, water was added to the graduation mark, shaken well, and the green-colored solution was stored in the dark.

b. **Developer solution:** In a 100 mL volumetric flask, H_2SO_4 (18 M, 1.11 mL) was added to 80 mL water followed by $CH_3COONa.3H_2O$ (10.2 g, 75.0 mmol) and 1,10-phenanthroline (0.901 g, 5.0 mmol). Water was added to the graduation mark and the solution was made homogenous before storing in the dark.

c. **Developer solution without 1,10-phenanthroline:** In a 100 mL volumetric flask, H_2SO_4 (18 M, 1.11 mL) was added to 80 mL water followed by $CH_3COONa.3H_2O$ (10.2 g, 75.0 mmol). Water was added to the graduation mark and the solution was made homogenous before storing it in the dark.

All photophysical measurements were performed in duplicate to determine the photon flux of the photoreactor used for fluoroalkyl-sulfonylalkylation reactions. 3 mL of 0.15 M ferrioxalate solution was added in a screw cap transparent glass vial and placed 1.5 cm away from the 440 nm blue LEDs. The solution was irradiated for 30, 60, 90, and 120 seconds. After each irradiation, 0.1 mL of aliquot was added to 5 mL of developer solution and allowed to stand 1 h for full complexation. After 1 h, 0.1 mL of aliquot was diluted to 2 mL in a cuvette using developer solution without 1,10- phenanthroline. Absorbance of both irradiated (for different intervals) and non-irradiated solutions were measured using UV-Vis spectroscopy.

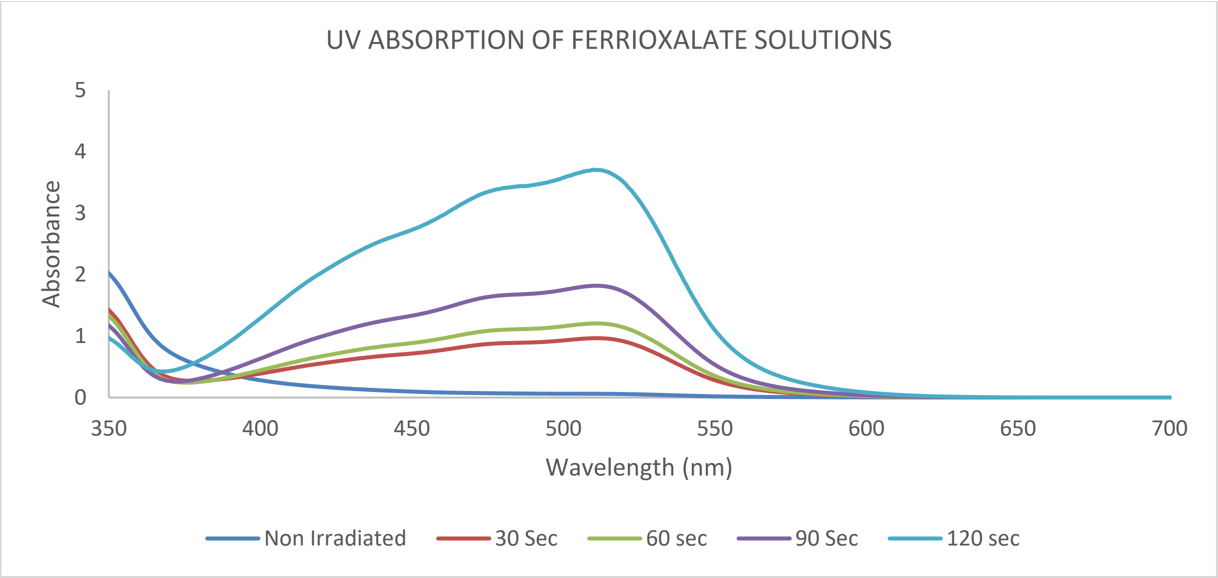

Supplementary Figure 06: Absorption of ferrioxalate solution under irradiation with 440 nm blue LEDs at different time intervals.

Absorbance at 510 nm was obtained from a plot of absorbance against the wavelength and are given below:

	**Absorbance (A) at 510 nm**
Non-Irradiated Solution	0.062
Irradiated Solution, 30s	0.966
Irradiated Solution, 60s	1.205
Irradiated Solution, 90s	1.818
Irradiated Solution, 120s	3.706

Number of ferrous ions formed during irradiation was calculated using following equation:

$$mol\ Fe^{2+} = \frac{\mathbf{D\,.\,\Delta A\,.\,V}}{\mathbf{l\,.\,\varepsilon}}$$

where, ε = molar absorptivity of ferrioxalate actinometer at 510 nm (11,100 $Lmol^{-1}cm^{-1}$)

l = path length (1 cm)

V = Volume of solution (L)

ΔA = difference in absorbance between irradiated and non-irradiated solution

D = dilution factor (20, in this case)

$$At\ 30s, mol\ Fe^{2+} = \frac{20 \text{ x } 0.904 \text{ x } 0.153}{1 \text{ X } 11{,}100} = 2.49\ x\ 10^{-4}\ mol$$

$$At\ 60s, mol\ Fe^{2+} = \frac{20 \text{ x } 1.143 \text{ x } 0.153}{1 \text{ X } 11{,}100} = 3.15\ x\ 10^{-4}\ mol$$

$$At\ 90s, mol\ Fe^{2+} = \frac{20 \text{ x } 1.756 \text{ x } 0.153}{1 \text{ X } 11{,}100} = 4.84\ x\ 10^{-4}\ mol$$

$$At\ 120s, mol\ Fe^{2+} = \frac{20 \text{ x } 3.644 \text{ x } 0.153}{1 \text{ X } 11{,}100} = 1.00\ x\ 10^{-3}\ mol$$

Fraction of light absorbed, f, required to determine the photon flux of photoreactor was calculated using following equation:

Fraction of light absorbed, $\mathbf{f = 1 - 10^{-A}}$, where A is absorbance

At 30s, $f = 1 - 10^{-0.966} = 0.8918$

At 60s, $f = 1 - 10^{-1.205} = 0.9376$

At 90s, $f = 1 - 10^{-1.818} = 0.9848$

At 120s, $f = 1 - 10^{-3.706} = 0.9998$

average fraction of light absorbed (f) = 0.9535

Now, photon flux of the photoreactor used in the fluoroalkyl-sulfonylalkylation was obtained from the equation given below:

$$\boldsymbol{Photon\ Flux = \frac{mol\ Fe^{2+}}{\phi\ .\ t\ .\ f}}$$

where, ϕ = quantum yield of 0.15 M ferrioxalate solution (1.14 at 405 nm)

t = time of irradiation (s)

f = fraction of light absorbed at a certain time

$$At\ 30s, Photon\ Flux = \frac{2.69 \text{ x } 10^{-4}}{1.14 \text{ x } 30 \text{ x } 0.8918} = 8.164\ x\ 10^{-6}\ Einstein\ s^{-1}$$

$$At\ 60s, Photon\ Flux = \frac{3.15 \text{ x } 10^{-4}}{1.14 \text{ x } 60 \text{ x } 0.9376} = 4.912\ x\ 10^{-6}\ Einstein\ s^{-1}$$

$$At\ 90s, Photon\ Flux = \frac{4.84 \text{ x } 10^{-4}}{1.14 \text{ x } 90 \text{ x } 0.9848} = 4.790\ x\ 10^{-6}\ Einstein\ s^{-1}$$

$$At\ 120s, Photon\ Flux = \frac{1.00 \text{ x } 10^{-3}}{1.14 \text{ x } 120 \text{ x } 0.9998} = 7.311\ x\ 10^{-6}\ Einstein\ s^{-1}$$

average photon flux of LED = 6.294 x 10^{-6} Einstein s^{-1}

Reaction 1

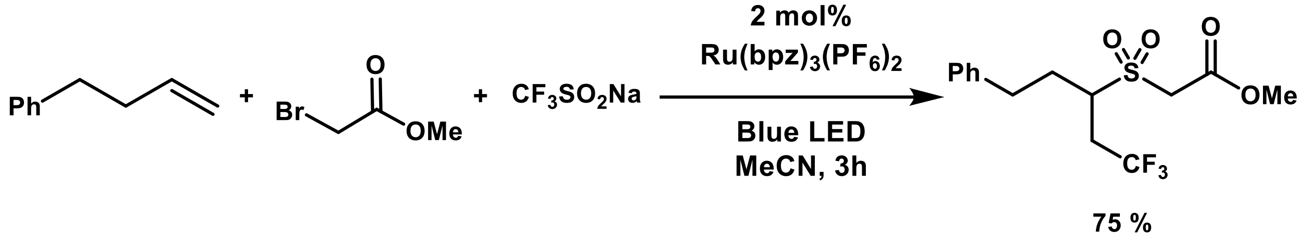

Reaction 2

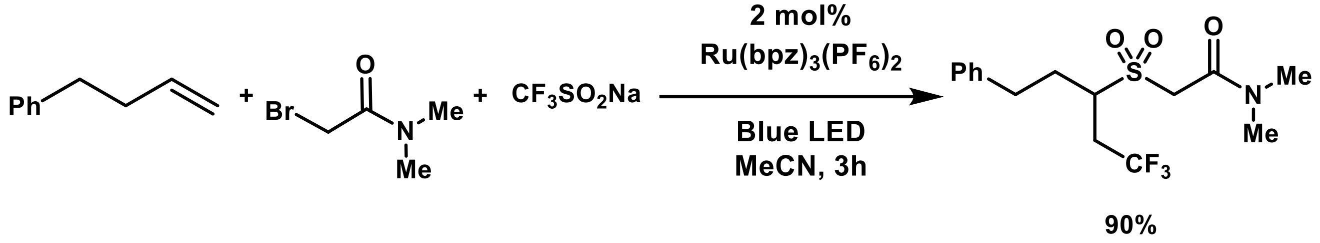

To determine the quantum yield, two different reactions (shown below) were performed in duplicate, and an average NMR yield was calculated.

For reaction 1, mol product = 75% of 0.1 mmol = 7.5×10^{-5} mol

For reaction 2, mol product = 90% of 0.1 mmol = 9.0×10^{-5} mol

Reaction time = 3 h = 10800 s

Finally, the quantum yield of above two fluoroalkyl-sulfonylalkylation was determined from the following equation:

$$\textbf{\textit{Quantum Yield}}\ (\boldsymbol{\phi}) = \frac{\textbf{mol product}}{\textbf{photon flux . t . f}}$$

$$\phi = \frac{7.5 \text{ x } 10^{-5}}{6.294 \text{ x } 10^{-6} \text{ x } 10800 \text{ x } 0.9535} = \mathbf{0.00115\ [Reaction\ 1]}$$

$$\phi = \frac{9.0 \text{ x } 10^{-5}}{6.294 \text{ x } 10^{-6} \text{ x } 10800 \text{ x } 0.9535} = \mathbf{0.00138\ [Reaction\ 2]}$$

3.6 Stern-Volmer quenching experiment

To perform Stern-Volmer quenching experiments, MeCN was degassed during sonication. 100 mL of 0.1 mM solution of photocatalyst $[Ru(bpz)_3]$ $[PF_6]_2$ in MeCN was prepared as stock solution. Different amounts of CF_3SO_2Na and methyl bromoacetate were dissolved in 5 mL stock solution of photocatalyst to get the following approximate concentrations:

**Concentration**	**CF_3SO_2Na**	**$BrCH_2CO_2Me$**
0 M	0.8603 mg	0.5 µl
0.001 M	1.7206 mg	1.0 µl
0.002 M	2.5809 mg	1.5 µl
0.003 M	3.4412 mg	2.5 µl
0.004 M	4.3015 mg	2.5 µl

These solutions were transferred to a quartz cuvette (d = 1.0 cm) and irradiated at λ_{max} (440 nm) of absorbance of photocatalyst. The resulting fluorescence spectra were recorded separately for each solution under study. The fluorescence spectrum of blank solution (0.1 mM PC, stock solution) was also recorded prior to the above test solutions.

Quenching With Methyl Bromoacetate:

Concentration	Intensity	I_0/I
0 M	134111.8	1
0.001 M	135766.6	0.98781137
0.002 M	136072.7	0.985589423
0.003 M	134588.5	0.996458337
0.004 M	138466.3	0.96855207

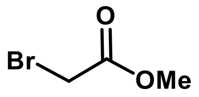

Quenching With Sodium trifluoromethanesulfinate:

Concentration	Intensity	I_0/I
0 M	130403.3	1
0.001 M	69585.89	1.873991
0.002 M	28557.93	4.566274
0.003 M	19772.03	6.595344
0.004 M	17481.04	7.459701

CF_3SO_2Na

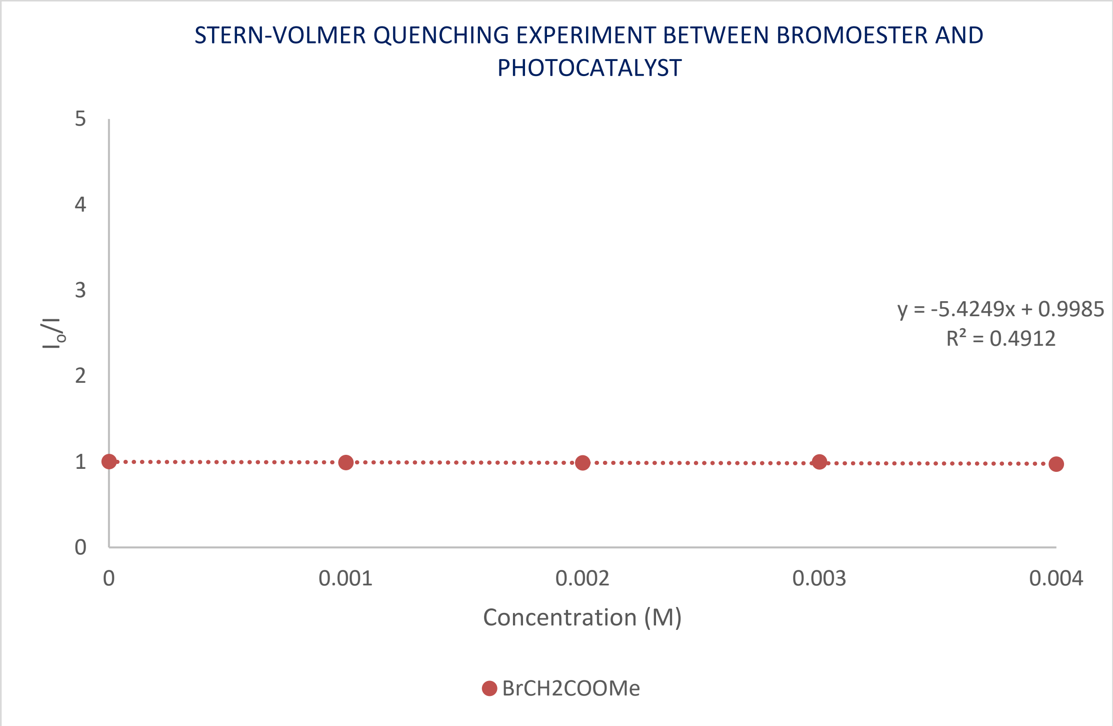

Supplementary Figure 07. The Stern-Volmer plot (quenching by methyl bromoacetate)

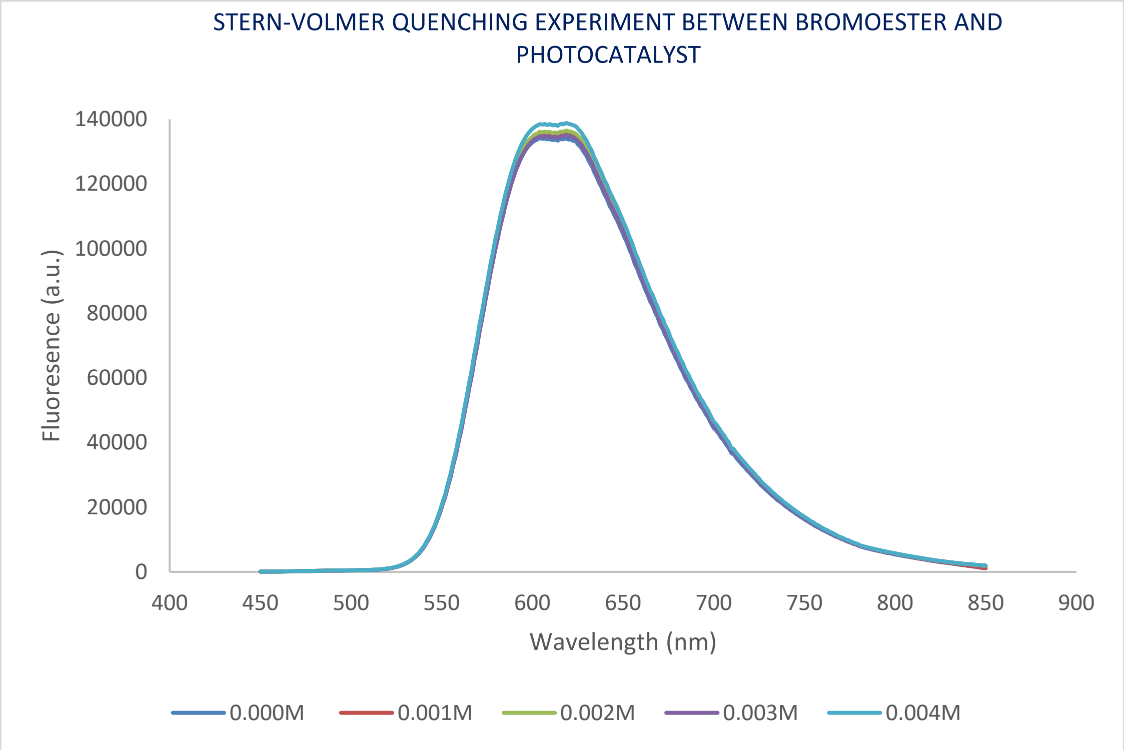

Supplementary Figure 08. Fluorescence emission spectra of [Ru(bpz)$_3$] [PF$_6$]$_2$with increasing concentrations of methyl bromoacetate.

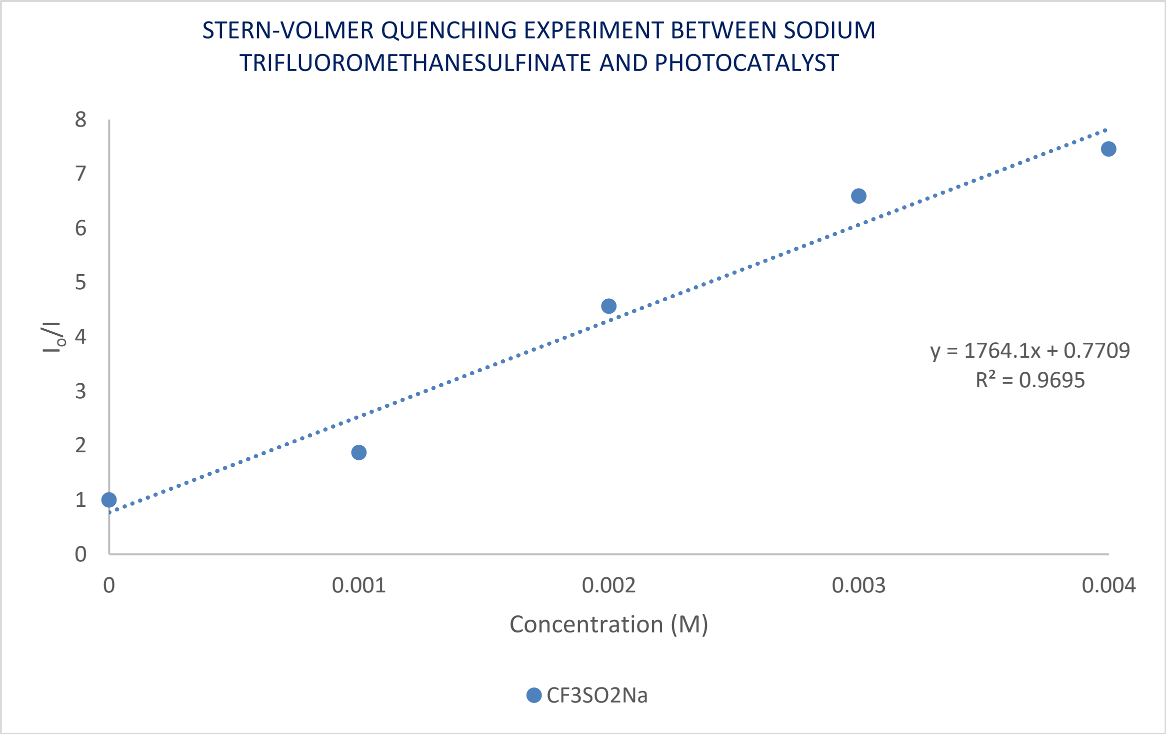

Supplementary Figure 09. The Stern-Volmer plot (quenching by CF_3SO_2Na).

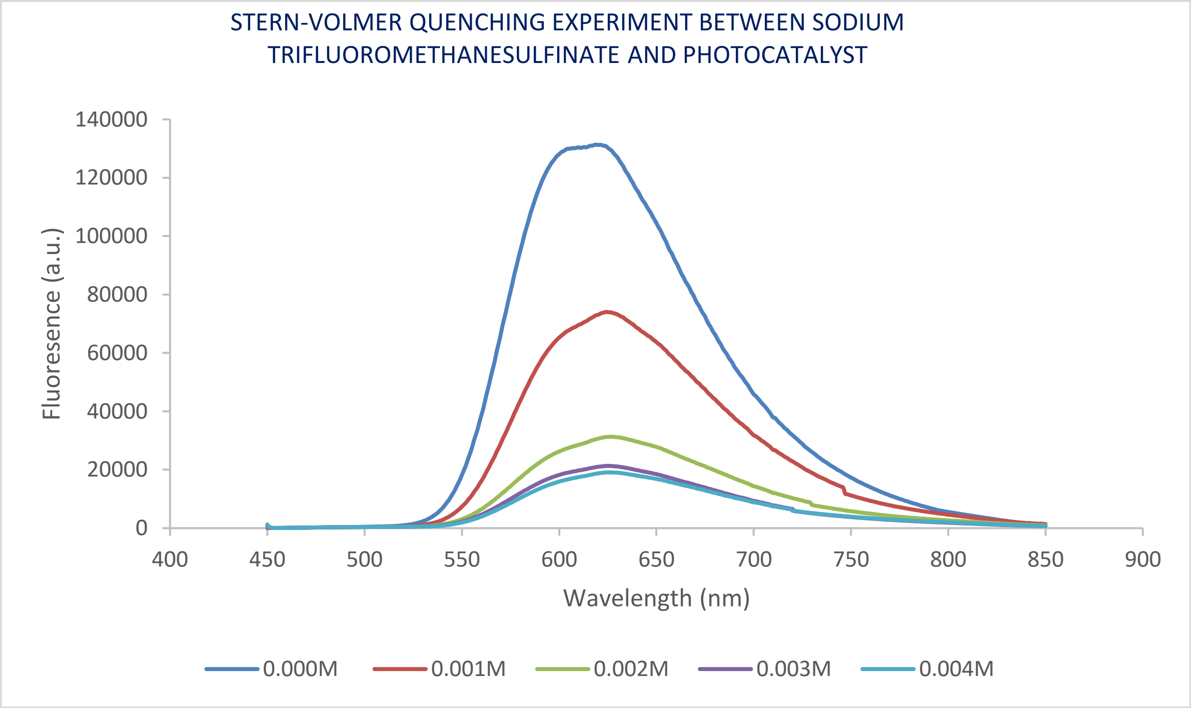

Supplementary Figure 10. Fluorescence emission spectra of $[Ru(bpz)_3]$ $[PF_6]_2$ with increasing concentrations of CF_3SO_2Na.

3.7 General optimized method of iodotriflouromethylation alkenes and alkynes

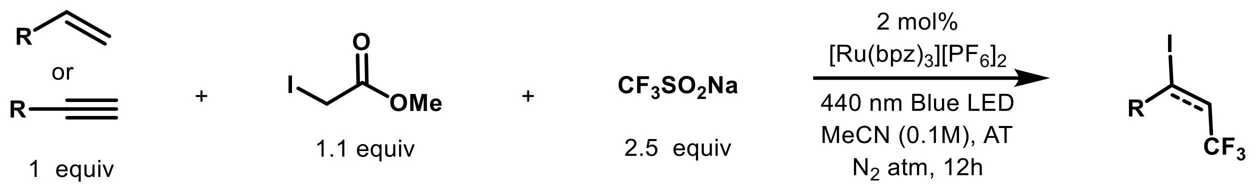

An oven-dried 6-dram vial was charged with a magnetic stir bar and photocatalyst $[Ru(bpz)]_3[PF_6]_2$ (2 mol%, 8.7 mg) inside the glove box. Corresponding alkene/ alkyne (0.5 mmol, 1 equiv.), sulfinate salt (1.25 mmol, 2.5 equiv.), alkyl iodide (0.55 mmol, 1.1 equiv.), and anhydrous MeCN (5.0 mL) were added to the vial. The resulting mixture was then irradiated with 440nm Blue LED. After 12 hours, the reaction mixture was concentrated in vacuo and check the NMR yield by using the 1,1,2,2-tetrachloroethane as NMR standard. The crude was purified by silica gel column chromatography using hexane – EtOAc as a mobile phase.

3.8 Competition study between α-halo esters

3.8.1 Competition experiments between chloro methyl acetate and bromo ethyl acetate

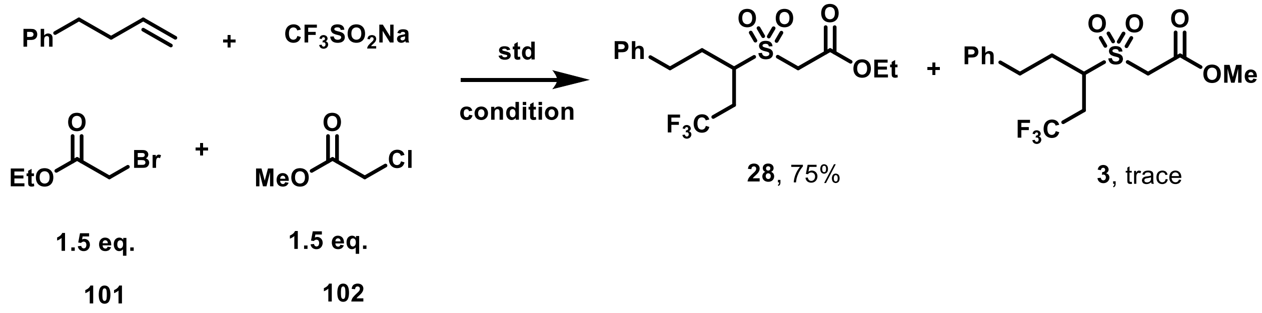

An oven-dried 6-dram vial was charged with a magnetic stir bar and photocatalyst $[Ru(bpz)]_3[PF_6]_2$ (2 mol%, 8.7 mg) inside the glove box. Phenyl butene (0.1 mmol, 1 equiv.),

sulfinate salt (0.2 mmol, 2 equiv.), ethyl 2-bromoacetate (0.3 mmol, 1.5 equiv.), methyl 2-chloroacetate (0.3 mmol, 1.5 equiv.), and anhydrous MeCN (5.0 mL) were added to the vial. The resulting mixture was then irradiated with 440nm Blue LED. After 12 hours, the reaction mixture was concentrated in vacuo and check the NMR yield by using the 1,1,2,2-tetrachloroethane as NMR standard. The crude was purified by silica gel column chromatography using hexane – EtOAc as a mobile phase.

3.8.2 Competition experiments between chloro methyl acetate and bromo ethyl acetate

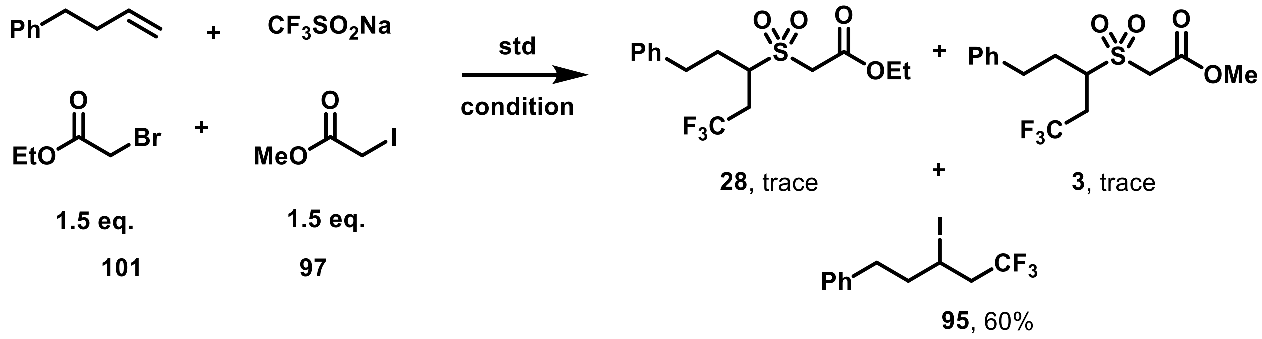

An oven-dried 6-dram vial was charged with a magnetic stir bar and photocatalyst $[Ru(bpz)]_3[PF_6]_2$ (2 mol%, 8.7 mg) inside the glove box. Phenyl butene (0.1 mmol, 1 equiv.), sulfinate salt (0.2 mmol, 2 equiv.), ethyl 2-bromoacetate (0.3 mmol, 1.5 equiv.), methyl 2-iodoacetate (0.3 mmol, 1.5 equiv.), and anhydrous MeCN (5.0 mL) were added to the vial. The resulting mixture was then irradiated with 440nm Blue LED. After 12 hours, the reaction mixture was concentrated in vacuo and check the NMR yield by using the 1,1,2,2-tetrachloroethane as NMR standard. The crude was purified by silica gel column chromatography using hexane – EtOAc as a mobile phase.

3.9 General optimized method of bromotriflouromethylation alkenes and alkynes

Method A

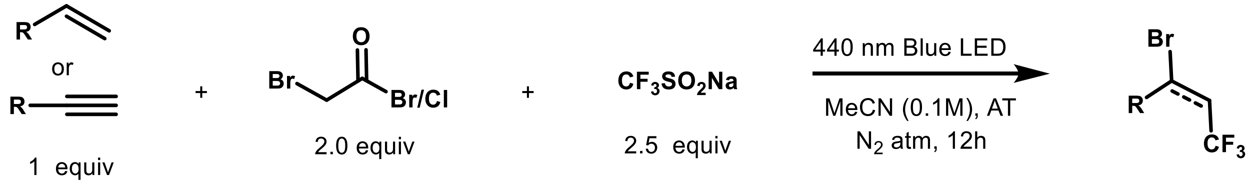

An oven-dried 6-dram vial was charged with a magnetic stir bar, Corresponding alkene/ alkyne (0.5 mmol, 1 equiv.), sulfinate salt (1.25 mmol, 2.5 equiv.), bromoacetyl bromides/ bromoacetyl chloride (1.0 mmol, 2.0 equiv.), and anhydrous MeCN (5.0 mL) were added to the vial. The resulting mixture was then irradiated with 440nm Blue LED. After 12 hours, the reaction mixture was concentrated in vacuo and check the NMR yield by using the 1,1,2,2-tetrachloroethane as NMR standard. The crude was purified by silica gel column chromatography using hexane – EtOAc as a mobile phase.

Control experiments.

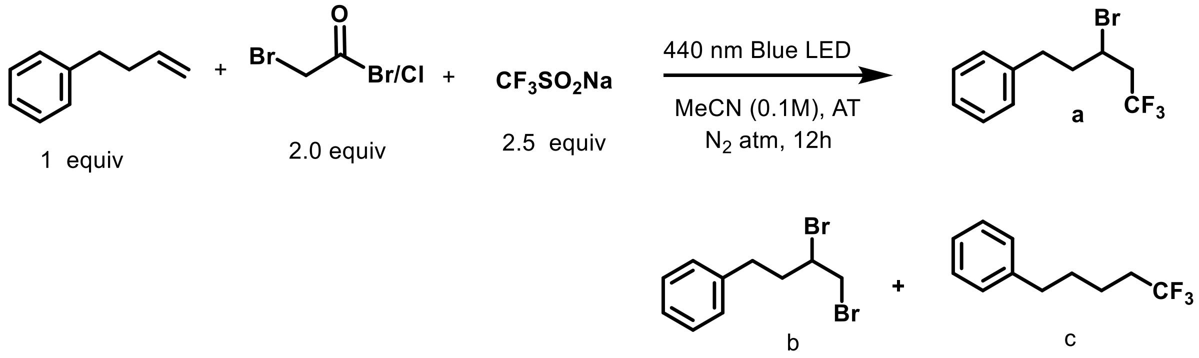

Deviation from the standard condition	Yield% (a)	Yield% (b)	Yield% (c)
**Without 440 nm blue LEDs**	-	80%	-
**Reaction at ambient light**	-	80%	-
**Reaction at dark**	-	81%	-
**Without bromoacetyl bromide**	-	-	14%

Method B

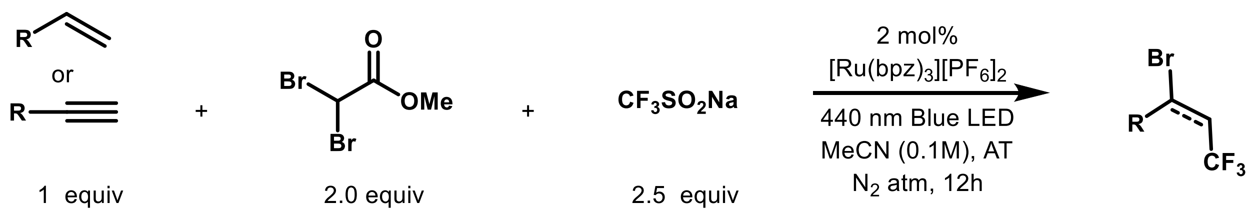

An oven-dried 6-dram vial was charged with a magnetic stir bar and photocatalyst $[Ru(bpz)]_3[PF_6]_2$ (2 mol%, 8.7 mg) inside the glove box. Corresponding alkene/ alkyne (0.5 mmol, 1 equiv.), sulfinate salt (1.25 mmol, 2.5 equiv.), methyl 2,2-dibromoacetate/ activated bromoacetate (0.55 mmol, 1.1 equiv.), and anhydrous MeCN (5.0 mL) were added to the vial. The resulting mixture was then irradiated with 440nm Blue LED. After 12 hours, the reaction mixture was concentrated in vacuo and check the NMR yield by using the 1,1,2,2-tetrachloroethane as NMR standard. The crude was purified by silica gel column chromatography using hexane – EtOAc as a mobile phase.

3.8 Unsuccessful substrates

Alkenes

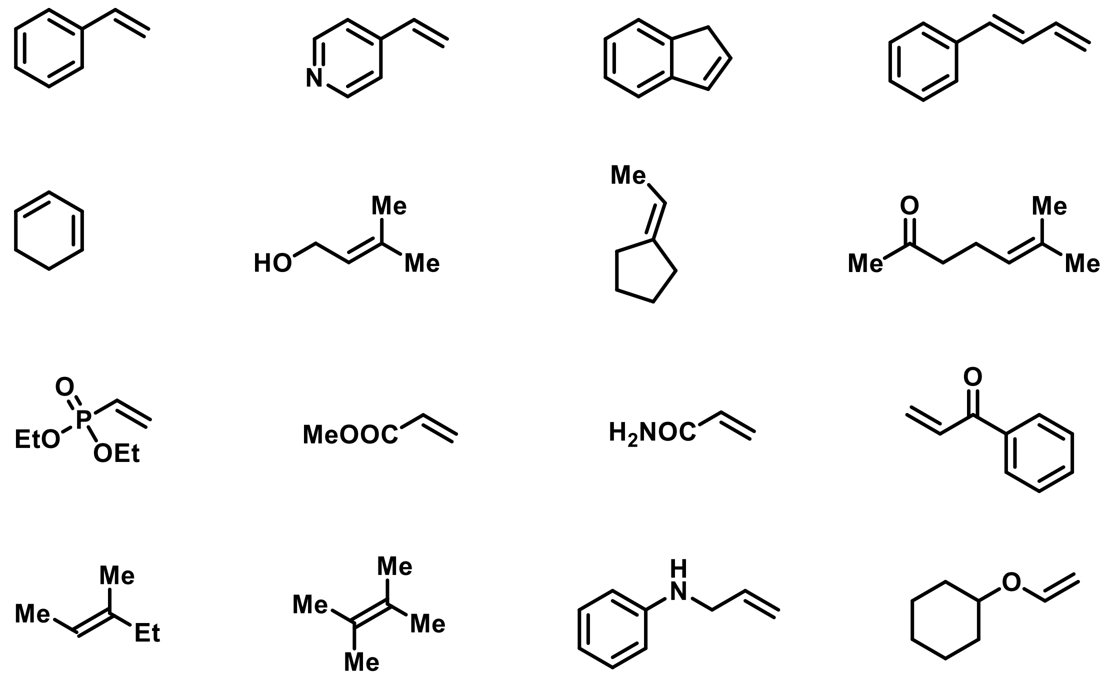

Alkyl bromides

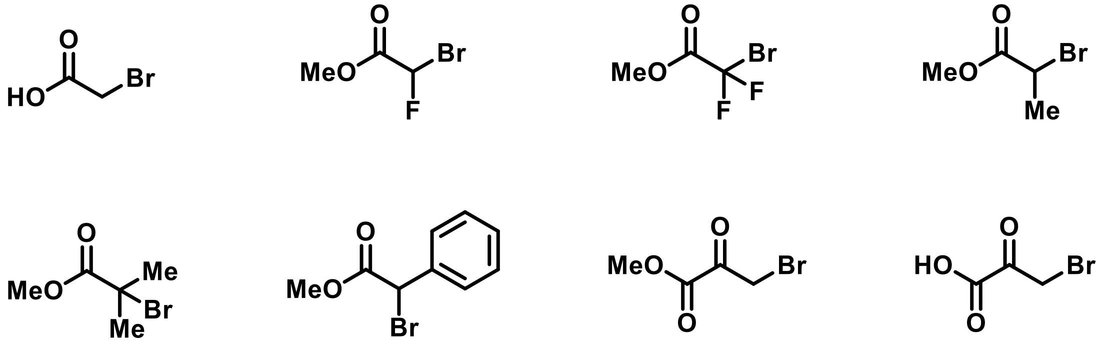

4.0 X-ray Crystal of Compounds

- Compound no: **21**

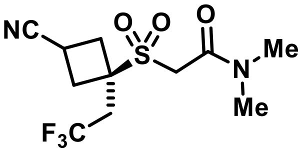

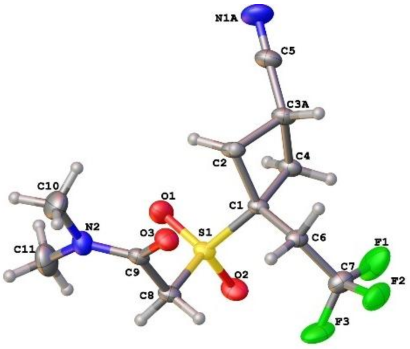

sin23_auto

Table 1 Crystal data and structure refinement for sin23_auto.

Identification code	sin23_auto
Empirical formula	$C_{11}H_{15}F_3N_2O_3S$
Formula weight	312.31
Temperature/K	173.01(10)
Crystal system	monoclinic
Space group	$P2_1/c$
a/Å	10.8398(2)
b/Å	15.1545(3)
c/Å	8.8060(2)
α/°	90
β/°	110.042(2)
γ/°	90
Volume/Å^3	1358.97(5)
Z	4

ρ_{calc}g/cm^3	1.526
μ/mm^{-1}	2.567
F(000)	648.0
Crystal size/mm^3	0.1 × 0.08 × 0.02
Radiation	Cu Kα (λ = 1.54184)
2Θ range for data collection/°	8.684 to 151.72
Index ranges	-13 ≤ h ≤ 12, -18 ≤ k ≤ 17, -8 ≤ l ≤ 10
Reflections collected	8220
Independent reflections	2710 [R_{int} = 0.0298, R_{sigma} = 0.0298]
Data/restraints/parameters	2710/0/202
Goodness-of-fit on F^2	1.050
Final R indexes [I>=2σ (I)]	R_1 = 0.0380, wR_2 = 0.1046
Final R indexes [all data]	R_1 = 0.0421, wR_2 = 0.1076
Largest diff. peak/hole / e Å^{-3}	0.41/-0.46

Table 2 Fractional Atomic Coordinates (×10^4) and Equivalent Isotropic Displacement Parameters (Å^2×10^3) for sin23_auto. U_{eq} is defined as 1/3 of the trace of the orthogonalised U_{IJ} tensor.

**Atom**	***x***	***y***	***z***	**U(eq)**
S1	3901.8(4)	5260.9(3)	2014.7(5)	21.91(14)
F1	3030.7(18)	2749.7(9)	1146.1(17)	62.6(4)
F2	1605.2(13)	2903.1(8)	-1243.5(15)	46.3(3)

Table 2 Fractional Atomic Coordinates (×10^4) and Equivalent Isotropic Displacement Parameters ($Å^2$×10^3) for sin23_auto. U_{eq} is defined as 1/3 of the trace of the orthogonalised U_{IJ} tensor.

**Atom**	***x***	***y***	***z***	**U(eq)**
F3	3433.8(12)	3581.0(9)	-588.6(17)	49.7(4)
O1	4178.2(13)	5889.1(9)	3321.7(15)	31.1(3)
O2	4935.9(12)	4671.7(9)	1993.8(17)	32.2(3)
O3	1123.8(12)	6195.3(9)	-528.6(16)	29.6(3)
N1A	1527(4)	4721(2)	6640(4)	46.4(8)
N2	2496.8(16)	7322.5(10)	558(2)	31.8(4)
C1	2525.8(16)	4599.9(11)	2050.1(19)	19.3(3)
C2	1521.7(18)	5108.5(12)	2637(2)	24.8(4)
C3A	1580(2)	4316.7(14)	3786(2)	26.4(6)
C4	2928.4(17)	4058.5(12)	3657(2)	24.8(4)
C5	1580(2)	4548.6(13)	5422(2)	32.4(4)
C6	1820.8(16)	4095.7(11)	489.5(19)	22.4(3)
C7	2490.1(19)	3342.1(13)	-15(2)	31.8(4)
C8	3388.6(16)	5871.2(12)	164(2)	23.9(4)
C9	2250.8(17)	6488.7(11)	42.6(19)	23.0(3)
C10	1367(3)	7875.0(14)	477(3)	46.8(6)
C11	3802(2)	7713.3(15)	1296(3)	48.3(6)
C3B	2436(10)	4859(7)	4496(13)	12(3)
N1B	1150(20)	4403(16)	6380(30)	39(5)

Table 3 Anisotropic Displacement Parameters (Å^2×10^3) for sin23_auto. The Anisotropic displacement factor exponent takes the form: $-2\pi^2[h^2a^{*2}U_{11}+2hka^*b^*U_{12}+…]$.

**Atom**	**U_{11}**	**U_{22}**	**U_{33}**	**U_{23}**	**U_{13}**	**U_{12}**
S1	14.9(2)	30.1(2)	20.5(2)	1.71(15)	5.81(16)	-0.56(15)
F1	91.8(12)	44.5(7)	47.4(8)	5.1(6)	18.5(8)	33.6(8)
F2	41.9(7)	47.5(7)	48.6(7)	-24.0(6)	14.3(6)	-7.2(6)
F3	32.5(7)	68.8(9)	57.4(8)	-23.1(7)	28.0(6)	-1.8(6)
O1	29.5(7)	37.6(7)	23.0(6)	-3.9(5)	4.8(5)	-7.0(5)
O2	16.9(6)	41.5(7)	39.6(7)	6.4(6)	11.3(6)	5.6(5)
O3	19.1(6)	34.5(7)	35.4(7)	1.5(5)	9.5(5)	-0.7(5)
N1A	60(2)	59(2)	25.1(15)	1.3(14)	21.3(15)	7.0(15)
N2	32.5(9)	26.9(7)	36.0(8)	-0.2(6)	12.0(7)	-3.3(6)
C1	15.3(7)	26.5(8)	17.2(7)	2.6(6)	6.9(6)	1.9(6)
C2	23.3(8)	33.5(9)	21.4(8)	4.7(7)	12.5(7)	6.4(7)
C3A	26.0(11)	35.3(11)	20.4(10)	2.6(8)	11.1(8)	0.7(8)
C4	22.5(8)	32.9(9)	19.8(8)	6.1(7)	8.3(7)	4.0(7)
C5	33.4(10)	42.2(10)	25.7(10)	6.1(8)	15.6(8)	3.1(8)
C6	16.2(7)	30.4(8)	21.5(8)	-1.3(7)	7.8(6)	-1.2(6)
C7	28.9(10)	36.2(10)	29.9(9)	-5.0(8)	9.5(8)	2.7(8)
C8	19.5(8)	32.2(9)	22.7(8)	2.7(7)	10.8(7)	-0.3(7)
C9	22.9(8)	28.4(8)	20.5(8)	4.0(6)	10.9(7)	-0.5(7)
C10	54.3(15)	30.9(10)	60.1(14)	0.3(10)	25.9(12)	9.3(10)
C11	48.5(14)	38.2(11)	52.5(13)	-4.2(10)	10.0(11)	-17.6(10)

Table 3 Anisotropic Displacement Parameters (Å^2×10^3) for sin23_auto. The Anisotropic displacement factor exponent takes the form: $-2\pi^2[h^2a^{*2}U_{11}+2hka^*b^*U_{12}+…]$.

**Atom**	**U_{11}**	**U_{22}**	**U_{33}**	**U_{23}**	**U_{13}**	**U_{12}**
C3B	10(5)	20(6)	8(5)	-4(4)	5(4)	-5(4)
N1B	39(11)	51(13)	29(9)	-2(9)	15(8)	-10(8)

Table 4 Bond Lengths for sin23_auto.

**Atom**	**Atom**	**Length/Å**	**Atom**	**Atom**	**Length/Å**
S1	O1	1.4437(13)	C1	C2	1.559(2)
S1	O2	1.4382(13)	C1	C4	1.563(2)
S1	C1	1.8058(16)	C1	C6	1.529(2)
S1	C8	1.7887(17)	C2	C3A	1.557(3)
F1	C7	1.336(2)	C2	C3B	1.641(11)
F2	C7	1.350(2)	C3A	C4	1.555(2)
F3	C7	1.337(2)	C3A	C5	1.482(3)
O3	C9	1.234(2)	C4	C3B	1.604(11)
N1A	C5	1.124(4)	C5	C3B	1.506(10)
N2	C9	1.338(2)	C5	N1B	1.12(2)
N2	C10	1.465(3)	C6	C7	1.500(2)
N2	C11	1.465(3)	C8	C9	1.522(2)

Table 5 Bond Angles for sin23_auto.

Atom	Atom	Atom	Angle/°	Atom	Atom	Atom	Angle/°
O1	S1	C1	107.40(7)	C5	C3A	C4	116.36(17)
O1	S1	C8	107.47(8)	C1	C4	C3B	89.2(4)
O2	S1	O1	118.03(8)	C3A	C4	C1	87.50(12)
O2	S1	C1	107.92(8)	N1A	C5	C3A	177.3(3)
O2	S1	C8	108.04(8)	N1B	C5	C3B	164.7(12)
C8	S1	C1	107.56(8)	C7	C6	C1	120.23(14)
C9	N2	C10	117.19(17)	F1	C7	F2	106.69(17)
C9	N2	C11	125.61(17)	F1	C7	F3	106.23(17)
C11	N2	C10	117.06(18)	F1	C7	C6	114.52(15)
C2	C1	S1	113.95(12)	F2	C7	C6	109.25(15)
C2	C1	C4	88.24(12)	F3	C7	F2	104.84(14)
C4	C1	S1	109.32(11)	F3	C7	C6	114.60(16)
C6	C1	S1	115.04(10)	C9	C8	S1	111.92(11)
C6	C1	C2	110.76(13)	O3	C9	N2	122.21(16)
C6	C1	C4	116.74(14)	O3	C9	C8	118.18(15)
C1	C2	C3B	88.0(4)	N2	C9	C8	119.61(15)
C3A	C2	C1	87.54(13)	C4	C3B	C2	84.1(5)
C4	C3A	C2	88.63(13)	C5	C3B	C2	109.8(6)
C5	C3A	C2	115.80(17)	C5	C3B	C4	112.1(7)

Table 6 Hydrogen Bonds for sin23_auto.

D	H	A	d(D-H)/Å	d(H-A)/Å	d(D-A)/Å	D-H-A/°
C2	H2A	O3[1]	0.99	2.54	3.453(2)	152.6
C4	H4B	F2[2]	0.99	2.34	3.315(2)	168.5
C6	H6A	O3[1]	0.99	2.28	3.2340(19)	161.0
C8	H8A	F3[3]	0.99	2.53	3.437(2)	151.5

[1]-X,1-Y,-Z; [2]+X,1/2-Y,1/2+Z; [3]1-X,1-Y,-Z

Table 7 Hydrogen Atom Coordinates (Å×10^4) and Isotropic Displacement Parameters (Å^2×10^3) for sin23_auto.

Atom	*x*	*y*	*z*	U(eq)
H2AA	1863.73	5668.41	3204.53	30
H2AB	652.24	5194.77	1789.79	30
H2A	635.56	4840.15	2286.64	30
H2B	1473.45	5748.62	2399.32	30
H3A	891.24	3867.1	3257.92	32
H4AA	3033.24	3418.19	3513.87	30
H4AB	3694.16	4302.17	4531.35	30
H4A	3883.19	3942.93	4133.09	30
H4B	2418.1	3508.18	3578.85	30
H6A	991.62	3865.27	572.48	27
H6B	1575.86	4530.49	-403.58	27
H8A	4138.07	6220.83	91.91	29

Table 7 Hydrogen Atom Coordinates (Å×10[4]) and Isotropic Displacement Parameters (Å[2]×10[3]) for sin23_auto.

**Atom**	***x***	***y***	***z***	**U(eq)**
H8B	3118.29	5454.56	-757.52	29
H10A	782.45	7925.57	-649.75	70
H10B	1673.05	8463.23	903.16	70
H10C	888.86	7605.81	1122.77	70
H11A	4465.21	7315.52	1148.77	72
H11B	3978.8	7804.74	2452.71	72
H11C	3835.98	8281.29	779.14	72
H3B	3120.55	5304.8	5059.92	15

Table 8 Atomic Occupancy for sin23_auto.

**Atom**	***Occupancy***	**Atom**	***Occupancy***	**Atom**	***Occupancy***
N1A	0.872(5)	H2AA	0.872(5)	H2AB	0.872(5)
H2A	0.128(5)	H2B	0.128(5)	C3A	0.872(5)
H3A	0.872(5)	H4AA	0.872(5)	H4AB	0.872(5)
H4A	0.128(5)	H4B	0.128(5)	C3B	0.128(5)
H3B	0.128(5)	N1B	0.128(5)		

Experimental

Single crystals of $C_{11}H_{15}F_3N_2O_3S$ [sin23_auto] were grown from solution of []. A suitable crystal was selected and [mounted on a nylon loop with a dab of paratone oil] on a ROD, Synergy Custom system, HyPix-Arc 150 diffractometer. The crystal was kept at 173.01(10) K during data collection. Using Olex2 [1], the structure was solved with the SHELXT [2] structure solution program using Intrinsic Phasing and refined with the SHELXL [3] refinement package using Least Squares minimisation.

1. Dolomanov, O.V., Bourhis, L.J., Gildea, R.J, Howard, J.A.K. & Puschmann, H. (2009), J. Appl. Cryst. 42, 339-341.

2. Sheldrick, G.M. (2015). Acta Cryst. A71, 3-8.

3. Sheldrick, G.M. (2015). Acta Cryst. C71, 3-8.

Crystal structure determination of [sin23_auto]

Crystal Data for $C_{11}H_{15}F_3N_2O_3S$ (*M* =312.31 g/mol): monoclinic, space group $P2_1/c$ (no. 14), a = 10.8398(2) Å, b = 15.1545(3) Å, c = 8.8060(2) Å, β = 110.042(2)°, V = 1358.97(5) Å^3, Z = 4, T = 173.01(10) K, μ(Cu Kα) = 2.567 mm^{-1}, *Dcalc* = 1.526 g/cm^3, 8220 reflections measured (8.684° ≤ 2Θ ≤ 151.72°), 2710 unique (R_{int} = 0.0298, R_{sigma} = 0.0298) which were used in all calculations. The final R_1 was 0.0380 (I > 2σ(I)) and wR_2 was 0.1076 (all data).

Refinement model description

Number of restraints - 0, number of constraints - unknown.

Details:

1. Fixed Uiso
 At 1.2 times of:
  All C(H) groups, All C(H,H) groups, All C(H,H,H,H) groups
 At 1.5 times of:
  All C(H,H,H) groups
2. Others
 Sof(H2A)=Sof(H2B)=Sof(H4A)=Sof(H4B)=Sof(C3B)=Sof(H3B)=Sof(N1B)=1-FVAR(1)
 Sof(N1A)=Sof(H2AA)=Sof(H2AB)=Sof(C3A)=Sof(H3A)=Sof(H4AA)=Sof(H4AB)=FVAR(1)
3.a Ternary CH refined with riding coordinates:
 C3A(H3A), C3B(H3B)
3.b Secondary CH2 refined with riding coordinates:
 C2(H2AA,H2AB), C2(H2A,H2B), C4(H4AA,H4AB), C4(H4A,H4B), C6(H6A,H6B), C8(H8A, H8B)
3.c Idealised Me refined as rotating group:
 C10(H10A,H10B,H10C), C11(H11A,H11B,H11C)

- Compound no: **40**

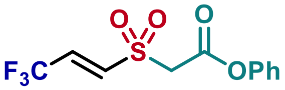

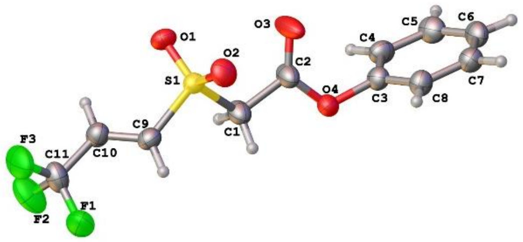

sin24c_auto

Table 1 Crystal data and structure refinement for sin24c_auto.

Identification code	sin24c_auto
Empirical formula	$C_{11}H_9F_3O_4S$
Formula weight	294.24
Temperature/K	173.00(10)
Crystal system	monoclinic
Space group	Pc
a/Å	14.3976(5)
b/Å	5.1553(2)
c/Å	8.3782(3)
α/°	90
β/°	93.540(3)
γ/°	90
Volume/Å^3	620.68(4)
Z	2
ρ_{calc}g/cm^3	1.574

μ/mm^{-1}	2.795
F(000)	300.0
Crystal size/mm^3	0.2 × 0.05 × 0.02
Radiation	Cu Kα (λ = 1.54184)
2Θ range for data collection/°	6.15 to 152.53
Index ranges	-18 ≤ h ≤ 18, -3 ≤ k ≤ 6, -10 ≤ l ≤ 10
Reflections collected	5818
Independent reflections	2246 [R_{int} = 0.0382, R_{sigma} = 0.0340]
Data/restraints/parameters	2246/2/173
Goodness-of-fit on F^2	1.096
Final R indexes [I>=2σ (I)]	R_1 = 0.0794, wR_2 = 0.2149
Final R indexes [all data]	R_1 = 0.0803, wR_2 = 0.2161
Largest diff. peak/hole / e Å^{-3}	1.45/-0.43
Flack parameter	0.02(2)

Table 2 Fractional Atomic Coordinates (×10^4) and Equivalent Isotropic Displacement Parameters (Å^2×10^3) for sin24c_auto. U_{eq} is defined as 1/3 of the trace of the orthogonalised U_{IJ} tensor.

**Atom**	***x***	***y***	***z***	**U(eq)**
S1	3935.9(13)	3280(3)	6315.5(17)	29.2(5)
F1	1609(5)	-642(13)	3432(10)	70(2)
F2	1427(6)	2818(17)	2081(8)	74(2)

Table 2 Fractional Atomic Coordinates (×10[4]) and Equivalent Isotropic Displacement Parameters ($Å^2$×10[3]) for sin24c_auto. U_{eq} is defined as 1/3 of the trace of the orthogonalised U_{IJ} tensor.

**Atom**	***x***	***y***	***z***	**U(eq)**
F3	758(5)	2417(18)	4242(10)	75(2)
O1	3784(5)	6011(11)	6290(9)	41.6(15)
O2	4021(5)	2008(13)	7842(8)	46.2(16)
O3	5914(5)	4760(20)	7142(12)	77(3)
O4	6493(4)	1796(12)	5529(8)	39.5(15)
C1	4906(6)	2450(20)	5207(11)	43(2)
C2	5818(6)	3190(20)	6091(12)	44(2)
C3	7422(6)	2203(17)	6196(11)	36.3(17)
C4	7931(7)	4239(17)	5636(11)	41.3(19)
C5	8848(8)	4466(19)	6232(16)	48(2)
C6	9235(6)	2710(20)	7321(12)	43(2)
C7	8684(7)	708(18)	7891(12)	44(2)
C8	7766(6)	424(18)	7305(11)	39.9(19)
C9	3025(6)	1729(16)	5166(10)	36.1(18)
C10	2360(6)	3109(17)	4469(11)	38.8(19)
C11	1532(7)	1940(20)	3626(13)	49(2)

Table 3 Anisotropic Displacement Parameters (Å^2×10^3) for sin24c_auto. The Anisotropic displacement factor exponent takes the form: $-2\pi^2[h^2a^{*2}U_{11}+2hka^*b^*U_{12}+\ldots]$.

**Atom**	U_{11}	U_{22}	U_{33}	U_{23}	U_{13}	U_{12}
S1	32.4(8)	28.8(9)	26.4(8)	0.2(8)	2.4(5)	1.2(8)
F1	53(4)	51(4)	103(6)	-20(4)	-19(4)	-2(3)
F2	73(5)	97(6)	50(4)	18(3)	-19(3)	-20(4)
F3	31(3)	106(6)	87(5)	-23(5)	4(3)	3(3)
O1	48(4)	28(3)	49(3)	-6(3)	0(3)	3(3)
O2	54(4)	49(4)	35(3)	10(3)	-4(3)	1(3)
O3	34(3)	108(7)	87(6)	-67(6)	-5(3)	3(4)
O4	36(3)	40(3)	41(3)	-8(3)	-8(2)	4(2)
C1	35(4)	56(5)	36(4)	-10(4)	1(3)	2(4)
C2	31(4)	60(6)	42(5)	-15(4)	1(3)	2(4)
C3	30(4)	39(4)	41(4)	-3(3)	5(3)	2(3)
C4	49(5)	32(4)	42(5)	1(3)	1(4)	1(4)
C5	39(5)	55(5)	52(5)	-7(5)	10(4)	-3(5)
C6	29(4)	52(5)	48(5)	-6(4)	5(3)	2(4)
C7	42(5)	41(5)	47(5)	-2(4)	0(4)	4(4)
C8	37(4)	39(5)	43(5)	4(3)	3(3)	2(3)
C9	37(4)	33(4)	39(4)	-3(3)	6(3)	2(3)
C10	35(4)	36(4)	46(5)	0(3)	3(4)	1(3)
C11	34(4)	60(6)	51(6)	9(4)	-5(4)	-4(4)

Table 4 Bond Lengths for sin24c_auto.

Atom	Atom	Length/Å	Atom	Atom	Length/Å
S1	O1	1.425(6)	C1	C2	1.516(12)
S1	O2	1.436(7)	C3	C4	1.379(13)
S1	C1	1.777(10)	C3	C8	1.376(13)
S1	C9	1.769(9)	C4	C5	1.388(15)
F1	C11	1.347(13)	C5	C6	1.379(16)
F2	C11	1.371(12)	C6	C7	1.402(14)
F3	C11	1.280(13)	C7	C8	1.389(13)
O3	C2	1.201(12)	C9	C10	1.302(12)
O4	C2	1.317(11)	C10	C11	1.476(13)
O4	C3	1.433(11)			

Table 5 Bond Angles for sin24c_auto.

Atom	Atom	Atom	Angle/˚	Atom	Atom	Atom	Angle/˚
O1	S1	O2	118.0(4)	C3	C4	C5	117.1(9)
O1	S1	C1	110.8(5)	C6	C5	C4	121.3(10)
O1	S1	C9	109.3(4)	C5	C6	C7	119.6(9)
O2	S1	C1	109.2(5)	C8	C7	C6	120.2(9)
O2	S1	C9	107.4(4)	C3	C8	C7	117.6(9)
C9	S1	C1	100.7(4)	C10	C9	S1	119.8(6)
C2	O4	C3	118.2(7)	C9	C10	C11	122.8(8)

Table 5 Bond Angles for sin24c_auto.

Atom	Atom	Atom	Angle/˚	Atom	Atom	Atom	Angle/˚
C2	C1	S1	111.7(7)	F1	C11	F2	102.5(9)
O3	C2	O4	125.2(9)	F1	C11	C10	113.0(8)
O3	C2	C1	125.8(9)	F2	C11	C10	110.9(8)
O4	C2	C1	108.9(8)	F3	C11	F1	108.6(9)
C4	C3	O4	118.8(8)	F3	C11	F2	105.6(9)
C8	C3	O4	117.0(8)	F3	C11	C10	115.2(10)
C8	C3	C4	124.1(8)				

Table 6 Hydrogen Bonds for sin24c_auto.

D	H	A	d(D-H)/Å	d(H-A)/Å	d(D-A)/Å	D-H-A/°
C1	H1A	O2[1]	0.99	2.52	3.242(11)	129.9
C1	H1B	O1[2]	0.99	2.80	3.655(12)	145.5
C1	H1B	O2[2]	0.99	2.87	3.661(13)	137.7
C1	H1B	O3[2]	0.99	2.54	3.350(13)	138.9
C6	H6	F1[3]	0.95	2.78	3.645(11)	151.2
C7	H7	F3[3]	0.95	2.82	3.518(11)	131.2
C8	H8	O4[4]	0.95	2.83	3.547(12)	132.6
C9	H9	O1[5]	0.95	2.48	3.262(10)	139.9
C10	H10	F1[6]	0.95	2.68	3.491(11)	143.7

[1]+X,-Y,-1/2+Z; [2]+X,1-Y,-1/2+Z; [3]1+X,-Y,1/2+Z; [4]+X,-Y,1/2+Z; [5]+X,-1+Y,+Z; [6]+X,1+Y,+Z

Table 7 Hydrogen Atom Coordinates (Å×10^4) and Isotropic Displacement Parameters (Å^2×10^3) for sin24c_auto.

**Atom**	***x***	***y***	***z***	**U(eq)**
H1A	4899.99	557.35	5000.3	51
H1B	4854.11	3349.56	4161.76	51
H4	7665.77	5434.54	4874.61	50
H5	9216.96	5861.12	5884.48	58
H6	9871.78	2847.05	7682.34	52
H7	8939.51	-456.01	8680.06	52
H8	7389.39	-949.88	7656.76	48
H9	3021.82	-105.24	5057.63	43
H10	2410.03	4945.77	4503.83	47

Experimental

Single crystals of $C_{11}H_9F_3O_4S$ [sin24c_auto] were []. A suitable crystal was selected and [mounted on nylon loop with a dab of paratone oil] on a ROD, Synergy Custom system, HyPix-Arc 150 diffractometer. The crystal was kept at 173.00(10) K during data collection. Using Olex2 [1], the structure was solved with the SHELXT [2] structure solution program using Intrinsic Phasing and refined with the SHELXL [3] refinement package using Least Squares minimisation.

1. Dolomanov, O.V., Bourhis, L.J., Gildea, R.J, Howard, J.A.K. & Puschmann, H. (2009), J. Appl. Cryst. 42, 339-341.

2. Sheldrick, G.M. (2015). Acta Cryst. A71, 3-8.

3. Sheldrick, G.M. (2015). Acta Cryst. C71, 3-8.

Crystal structure determination of [sin24c_auto]

Crystal Data for $C_{11}H_9F_3O_4S$ (M =294.24 g/mol): monoclinic, space group Pc (no. 7), a = 14.3976(5) Å, b = 5.1553(2) Å, c = 8.3782(3) Å, β = 93.540(3)°, V = 620.68(4) Å^3, Z = 2, T = 173.00(10) K, μ(Cu Kα) = 2.795 mm^{-1}, $Dcalc$ = 1.574 g/cm^3, 5818 reflections measured (6.15° ≤ 2Θ ≤ 152.53°), 2246 unique (R_{int} = 0.0382, R_{sigma} = 0.0340) which were used in all calculations. The final R_1 was 0.0794 (I > 2σ(I)) and wR_2 was 0.2161 (all data).

Refinement model description

Number of restraints - 2, number of constraints - unknown.

Details:

1. Fixed Uiso
At 1.2 times of:
All C(H) groups, All C(H,H) groups
2.a Secondary CH2 refined with riding coordinates:
C1(H1A,H1B)
2.b Aromatic/amide H refined with riding coordinates:
C4(H4), C5(H5), C6(H6), C7(H7), C8(H8), C9(H9), C10(H10)

- Compound no: **48**

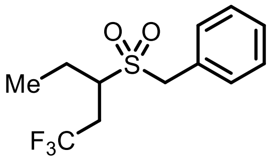

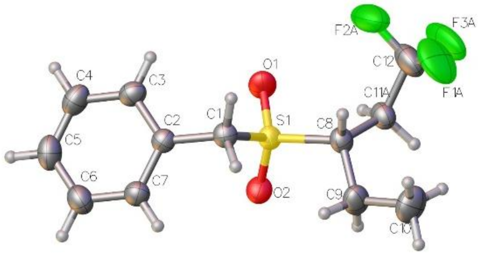

sin22c_auto

Table 1 Crystal data and structure refinement for sin22c_auto.

Identification code	sin22c_auto
Empirical formula	$C_{12}H_{15}F_3O_2S$
Formula weight	280.30
Temperature/K	173.00(10)
Crystal system	monoclinic
Space group	$P2_1/n$

a/Å	6.35896(17)
b/Å	22.8038(6)
c/Å	9.0697(2)
α/°	90
β/°	96.585(3)
γ/°	90
Volume/Å^3	1306.51(6)
Z	4
ρ_{calc}g/cm^3	1.425
μ/mm^{-1}	2.498
F(000)	584.0
Crystal size/mm^3	0.2 × 0.04 × 0.02
Radiation	Cu Kα (λ = 1.54184)
2Θ range for data collection/°	7.754 to 153.384
Index ranges	$-7 \le h \le 7$, $-27 \le k \le 27$, $-10 \le l \le 11$
Reflections collected	7279
Independent reflections	2545 [R_{int} = 0.0263, R_{sigma} = 0.0301]
Data/restraints/parameters	2545/0/192
Goodness-of-fit on F^2	1.075
Final R indexes [I>=2σ (I)]	R_1 = 0.0433, wR_2 = 0.1177
Final R indexes [all data]	R_1 = 0.0501, wR_2 = 0.1221
Largest diff. peak/hole / e Å^{-3}	0.29/-0.38

Table 2 Fractional Atomic Coordinates (×10^4) and Equivalent Isotropic Displacement Parameters (Å^2×10^3) for sin22c_auto. U_{eq} is defined as 1/3 of the trace of the orthogonalised U_{IJ} tensor.

**Atom**	***x***	***y***	***z***	**U(eq)**
S1	3533.7(8)	2795.9(2)	6795.4(5)	31.99(17)
F1A	3165.4	4713.4	5863.6	111.3(12)
F1B	4940(30)	4883(6)	6402(18)	132(7)
F2A	5737.5	4279	7074.19	92.4(9)
F2B	4480(20)	4328(6)	8277(13)	96(5)
F3A	3722.6	4823.2	8178.3	109.3(12)
F3B	1730(30)	4686(5)	6976(18)	106(5)
O1	5424(3)	2956.6(7)	7744.3(18)	45.8(4)
O2	2019(2)	2430.6(7)	7421.9(17)	41.1(4)
C1	4322(3)	2450.1(9)	5180(2)	34.6(5)
C2	5205(3)	1849.6(9)	5568(2)	33.2(4)
C3	7292(4)	1776.4(11)	6182(2)	40.9(5)
C4	8065(4)	1218.3(12)	6536(3)	50.2(6)
C5	6777(5)	735.0(12)	6283(3)	54.2(7)
C6	4699(4)	806.0(11)	5658(3)	50.5(6)
C7	3913(4)	1361.0(10)	5304(3)	40.8(5)
C8	2204(4)	3457.9(9)	6106(2)	37.5(5)
C9	-25(4)	3321.9(11)	5393(3)	52.5(7)

Table 2 Fractional Atomic Coordinates (×10^4) and Equivalent Isotropic Displacement Parameters ($Å^2$×10^3) for sin22c_auto. U_{eq} is defined as 1/3 of the trace of the orthogonalised U_{IJ} tensor.

**Atom**	*x*	*y*	*z*	**U(eq)**
C10	-1136(5)	3824.3(15)	4579(3)	66.9(8)
C11A	2334(5)	3919.8(12)	7331(3)	43.0(7)
C11B	3590(30)	3936(7)	5897(17)	53(4)
C12	3694(6)	4431.6(13)	7057(4)	67.6(9)

Table 3 Anisotropic Displacement Parameters ($Å^2$×10^3) for sin22c_auto. The Anisotropic displacement factor exponent takes the form: $-2\pi^2[h^2a^{*2}U_{11}+2hka^*b^*U_{12}+\ldots]$.

**Atom**	U_{11}	U_{22}	U_{33}	U_{23}	U_{13}	U_{12}
S1	34.3(3)	32.2(3)	28.3(3)	-0.85(19)	-1.5(2)	0.99(19)
F1A	157(3)	53.2(14)	127(2)	37.1(16)	31(2)	-15.0(17)
F1B	198(18)	74(9)	121(12)	-7(8)	3(11)	-96(11)
F2A	66.8(15)	75.9(16)	140(2)	-29.7(15)	35.2(15)	-25.8(12)
F2B	144(13)	84(9)	53(7)	-10(6)	-16(7)	-20(8)
F3A	128(2)	61.4(16)	149(3)	-59.3(16)	60(2)	-37.2(15)
F3B	142(13)	53(7)	128(12)	-14(7)	42(10)	16(8)
O1	44.0(9)	47.1(9)	42.1(9)	-7.8(7)	-13.4(7)	1.2(7)
O2	43.0(9)	41.8(9)	39.8(9)	5.8(7)	10.1(7)	2.5(7)
C1	38.6(11)	35.9(11)	29.1(10)	-0.8(8)	2.9(9)	0.8(9)
C2	34.0(10)	35.5(11)	29.7(10)	-1.6(8)	2.7(8)	0.9(9)

Table 3 Anisotropic Displacement Parameters ($Å^2×10^3$) for sin22c_auto. The Anisotropic displacement factor exponent takes the form: $-2\pi^2[h^2a^{*2}U_{11}+2hka^*b^*U_{12}+…]$.

**Atom**	**U_{11}**	**U_{22}**	**U_{33}**	**U_{23}**	**U_{13}**	**U_{12}**
C3	35.3(11)	47.5(13)	39.0(12)	-3.3(10)	0.6(9)	-0.7(10)
C4	43.0(13)	60.6(16)	45.5(14)	-0.4(12)	-1.3(11)	14.8(12)
C5	66.4(17)	43.8(14)	52.3(15)	4.1(11)	6.0(13)	16.5(12)
C6	57.0(15)	36.8(12)	58.1(15)	-2.4(11)	7.3(12)	-1.2(11)
C7	37.9(12)	39.5(12)	44.1(13)	-3.4(10)	1.9(10)	-0.7(9)
C8	42.2(12)	32.4(11)	37.1(11)	1.5(9)	0.5(9)	1.8(9)
C9	49.4(14)	45.9(14)	58.0(16)	-0.9(12)	-12.2(12)	7.2(11)
C10	62.0(18)	74(2)	62.0(18)	10.7(15)	-6.3(14)	20.9(15)
C11A	47.8(16)	35.0(14)	47.4(16)	-4.2(12)	10.6(13)	-0.7(12)
C11B	64(10)	48(9)	46(8)	2(7)	6(7)	-14(7)
C12	83(2)	37.2(14)	86(2)	-11.3(15)	25.2(19)	-10.4(14)

Table 4 Bond Lengths for sin22c_auto.

**Atom**	**Atom**	**Length/Å**	**Atom**	**Atom**	**Length/Å**
S1	O1	1.4434(16)	C2	C7	1.389(3)
S1	O2	1.4387(16)	C3	C4	1.389(3)
S1	C1	1.785(2)	C4	C5	1.377(4)
S1	C8	1.806(2)	C5	C6	1.386(4)
F1A	C12	1.270(4)	C6	C7	1.385(3)

Table 4 Bond Lengths for sin22c_auto.

Atom	Atom	Length/Å	Atom	Atom	Length/Å
F1B	C12	1.463(13)	C8	C9	1.521(3)
F2A	C12	1.343(4)	C8	C11A	1.526(3)
F2B	C12	1.185(12)	C8	C11B	1.429(15)
F3A	C12	1.352(3)	C9	C10	1.496(4)
F3B	C12	1.371(15)	C11A	C12	1.490(4)
C1	C2	1.506(3)	C11B	C12	1.539(16)
C2	C3	1.389(3)			

Table 5 Bond Angles for sin22c_auto.

Atom	Atom	Atom	Angle/°	Atom	Atom	Atom	Angle/°
O1	S1	C1	107.96(10)	C11B	C8	S1	114.4(7)
O1	S1	C8	108.60(10)	C11B	C8	C9	131.2(7)
O2	S1	O1	117.45(10)	C10	C9	C8	114.7(2)
O2	S1	C1	109.38(10)	C12	C11A	C8	113.8(2)
O2	S1	C8	108.22(10)	C8	C11B	C12	116.6(11)
C1	S1	C8	104.45(10)	F1A	C12	F2A	107.6(2)
C2	C1	S1	110.02(14)	F1A	C12	F3A	106.6(2)
C3	C2	C1	121.1(2)	F1A	C12	C11A	115.8(3)
C7	C2	C1	119.44(19)	F1B	C12	C11B	102.8(9)
C7	C2	C3	119.5(2)	F2A	C12	F3A	103.5(2)

Table 5 Bond Angles for sin22c_auto.

Atom	Atom	Atom	Angle/°	Atom	Atom	Atom	Angle/°
C4	C3	C2	120.0(2)	F2A	C12	C11A	112.0(2)
C5	C4	C3	120.5(2)	F2B	C12	F1B	109.2(10)
C4	C5	C6	119.7(2)	F2B	C12	F3B	114.8(11)
C7	C6	C5	120.2(2)	F2B	C12	C11B	118.1(9)
C6	C7	C2	120.2(2)	F3A	C12	C11A	110.5(3)
C9	C8	S1	110.68(16)	F3B	C12	F1B	102.2(10)
C9	C8	C11A	114.9(2)	F3B	C12	C11B	107.9(10)
C11A	C8	S1	109.93(17)				

Table 6 Hydrogen Bonds for sin22c_auto.

D	H	A	d(D-H)/Å	d(H-A)/Å	d(D-A)/Å	D-H-A/°
C1	H1A	O1[1]	0.99	2.30	3.260(3)	162.9

[1]-1/2+X,1/2-Y,-1/2+Z

Table 7 Hydrogen Atom Coordinates (Å×10^4) and Isotropic Displacement Parameters (Å^2×10^3) for sin22c_auto.

Atom	*x*	*y*	*z*	U(eq)
H1A	3087.47	2415.71	4413.81	42
H1B	5409.15	2691.96	4769.46	42
H3	8190.12	2107.93	6359.36	49
H4	9492.52	1169.67	6956.09	60

Table 7 Hydrogen Atom Coordinates (Å×10^4) and Isotropic Displacement Parameters (Å^2×10^3) for sin22c_auto.

**Atom**	***x***	***y***	***z***	**U(eq)**
H5	7309.45	354.47	6536.31	65
H6	3811.8	472.75	5470.9	61
H7	2485.6	1407.64	4880.53	49
H8A	3006.26	3614.54	5305.81	45
H8B	1789.39	3592.87	7081.54	45
H9A	47.84	2992.02	4691.15	63
H9B	-880.67	3190.44	6176.81	63
H10A	-400.94	3927.34	3722.69	100
H10B	-1138.34	4162.89	5244.17	100
H10C	-2598.48	3711.74	4236.55	100
H11A	888.07	4062.17	7434.66	52
H11B	2895.81	3733.84	8281.27	52
H11C	3153.19	4109.78	4908.93	64
H11D	5037.16	3777.71	5881.01	64

Table 8 Atomic Occupancy for sin22c_auto.

**Atom**	***Occupancy***	**Atom**	***Occupancy***	**Atom**	***Occupancy***
F1A	0.831(4)	F1B	0.169(4)	F2A	0.831(4)
F2B	0.169(4)	F3A	0.831(4)	F3B	0.169(4)

Table 8 Atomic Occupancy for sin22c_auto.

Atom	*Occupancy*	Atom	*Occupancy*	Atom	*Occupancy*
H8A	0.831(4)	H8B	0.169(4)	C11A	0.831(4)
H11A	0.831(4)	H11B	0.831(4)	C11B	0.169(4)
H11C	0.169(4)	H11D	0.169(4)		

Experimental

Single crystals of $C_{12}H_{15}F_3O_2S$ [sin22c_auto] were []. A suitable crystal was selected and [mounted on a nylon loop with a dab of paratone oil.] on a ROD, Synergy Custom system, HyPix-Arc 150 diffractometer. The crystal was kept at 173.00(10) K during data collection. Using Olex2 [1], the structure was solved with the SHELXT [2] structure solution program using Intrinsic Phasing and refined with the SHELXL [3] refinement package using Least Squares minimisation.

1. Dolomanov, O.V., Bourhis, L.J., Gildea, R.J, Howard, J.A.K. & Puschmann, H. (2009), J. Appl. Cryst. 42, 339-341.
2. Sheldrick, G.M. (2015). Acta Cryst. A71, 3-8.
3. Sheldrick, G.M. (2015). Acta Cryst. C71, 3-8.

Crystal structure determination of [sin22c_auto]

Crystal Data for $C_{12}H_{15}F_3O_2S$ (M =280.30 g/mol): monoclinic, space group $P2_1/n$ (no. 14), a = 6.35896(17) Å, b = 22.8038(6) Å, c = 9.0697(2) Å, β = 96.585(3)°, V = 1306.51(6) Å^3, Z = 4, T = 173.00(10) K, μ(Cu Kα) = 2.498 mm^{-1}, $Dcalc$ = 1.425 g/cm^3, 7279 reflections measured (7.754° ≤ 2Θ ≤ 153.384°), 2545 unique (R_{int} = 0.0263, R_{sigma} = 0.0301) which were used in all calculations. The final R_1 was 0.0433 (I > 2σ(I)) and wR_2 was 0.1221 (all data).

Refinement model description

Number of restraints - 0, number of constraints - unknown.

Details:

1. Fixed Uiso
   At 1.2 times of:
   All C(H) groups, All C(H,H) groups
   At 1.5 times of:
   All C(H,H,H) groups
   2. Others
   Sof(F1B)=Sof(F2B)=Sof(F3B)=Sof(H8B)=Sof(C11B)=Sof(H11C)=Sof(H11D)=1-FVAR(1)

Sof(F1A)=Sof(F2A)=Sof(F3A)=Sof(H8A)=Sof(C11A)=Sof(H11A)=Sof(H11B)=FVAR(1)
Fixed X: F1A(0.31654) F2A(0.57375) F3A(0.37226)
Fixed Y: F1A(0.47134) F2A(0.4279) F3A(0.48232)
Fixed Z: F1A(0.58636) F2A(0.707419) F3A(0.81783)
3.a Ternary CH refined with riding coordinates:
C8(H8A), C8(H8B)
3.b Secondary CH2 refined with riding coordinates:
C1(H1A,H1B), C9(H9A,H9B), C11A(H11A,H11B), C11B(H11C,H11D)
3.c Aromatic/amide H refined with riding coordinates:
C3(H3), C4(H4), C5(H5), C6(H6), C7(H7)
3.d Idealised Me refined as rotating group:
C10(H10A,H10B,H10C)

- Compound no: **56**

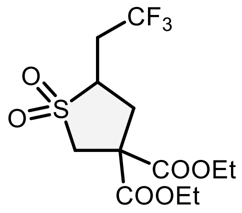

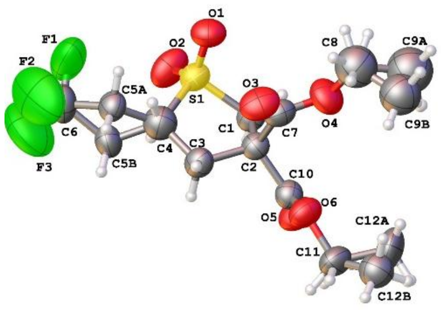

sin21_auto

Table 1 Crystal data and structure refinement for sin21_auto.

Identification code	sin21_auto
Empirical formula	$C_{12}H_{17}F_3O_6S$
Formula weight	346.31
Temperature/K	293(2)
Crystal system	monoclinic
Space group	$P2_1/c$
a/Å	12.8077(10)
b/Å	13.3519(9)
c/Å	9.8528(8)
α/°	90
β/°	106.248(8)
γ/°	90
Volume/Å^3	1617.6(2)

Z	4
ρ_{calc}g/cm^3	1.422
μ/mm^{-1}	2.319
F(000)	720.0
Crystal size/mm^3	0.3 × 0.02 × 0.01
Radiation	Cu Kα (λ = 1.54184)
2Θ range for data collection/°	9.778 to 151.974
Index ranges	-15 ≤ h ≤ 15, -16 ≤ k ≤ 13, -11 ≤ l ≤ 12
Reflections collected	9100
Independent reflections	3164 [R_{int} = 0.0469, R_{sigma} = 0.0471]
Data/restraints/parameters	3164/35/239
Goodness-of-fit on F^2	0.993
Final R indexes [I>=2σ (I)]	R_1 = 0.0766, wR_2 = 0.2283
Final R indexes [all data]	R_1 = 0.1146, wR_2 = 0.2640
Largest diff. peak/hole / e Å^{-3}	0.53/-0.44

Table 2 Fractional Atomic Coordinates (×10^4) and Equivalent Isotropic Displacement Parameters (Å^2×10^3) for sin21_auto. U_{eq} is defined as 1/3 of the trace of the orthogonalised U_{IJ} tensor.

**Atom**	***x***	***y***	***z***	**U(eq)**
S1	4064.4(9)	7114.2(8)	1865.2(11)	59.0(4)
F1	1650(3)	6752(4)	2317(6)	153.7(19)
F2	1676(6)	5611(5)	3767(5)	203(3)
F3	1113(5)	5323(5)	1740(7)	203(3)
O1	4404(3)	7806(2)	3012(3)	74.4(9)
O2	3196(3)	7386(3)	666(3)	90.5(11)
O3	6318(3)	6309(3)	5022(3)	84.4(10)
O4	7186(3)	7044(3)	3613(4)	87.8(11)
O5	6458(3)	5198(3)	717(3)	83.6(11)
O6	7223(2)	4824(2)	2993(3)	71.9(9)
C1	5777(3)	5956(3)	2502(4)	53.2(9)
C2	5227(4)	6726(3)	1375(4)	60.5(10)
C3	4847(4)	5313(3)	2767(5)	62.7(11)
C4	3834(4)	5921(4)	2579(6)	84.3(15)
C5A	3024(7)	5868(9)	3308(10)	63(3)

Table 2 Fractional Atomic Coordinates (×10^4) and Equivalent Isotropic Displacement Parameters (Å^2×10^3) for sin21_auto. U_{eq} is defined as 1/3 of the trace of the orthogonalised U_{IJ} tensor.

Atom	*x*	*y*	*z*	U(eq)
C5B	2810(6)	5407(6)	2122(9)	71(3)
C6	1857(4)	5817(5)	2580(7)	87.9(16)
C7	6439(4)	6452(3)	3871(5)	64.9(11)
C8	7931(6)	7534(7)	4828(8)	128(2)
C9A	8876(9)	7801(12)	4426(15)	151(6)
C9B	9009(11)	7160(20)	5120(40)	121(13)
C10	6530(3)	5294(3)	1950(4)	58.1(10)
C11	7967(4)	4109(4)	2630(7)	85.7(15)
C12A	8938(6)	4625(8)	2564(11)	127(4)
C12B	9060(30)	3950(30)	3550(40)	86(12)

Table 3 Anisotropic Displacement Parameters (Å^2×10^3) for sin21_auto. The Anisotropic displacement factor exponent takes the form: $-2\pi^2[h^2a^{*2}U_{11}+2hka^*b^*U_{12}+\ldots]$.

Atom	U_{11}	U_{22}	U_{33}	U_{23}	U_{13}	U_{12}
S1	62.1(6)	60.6(7)	55.8(6)	2.8(4)	18.9(5)	8.0(5)
F1	103(3)	117(3)	263(6)	37(4)	88(4)	26(3)
F2	275(7)	225(6)	149(4)	1(4)	125(5)	-71(6)
F3	201(6)	208(6)	189(5)	-61(5)	35(4)	-93(5)
O1	88(2)	65.6(19)	69.8(19)	-11.9(15)	22.6(17)	2.6(16)
O2	74(2)	126(3)	66(2)	8(2)	11.6(17)	34(2)
O3	105(3)	97(3)	50.3(16)	-5.6(16)	19.6(17)	17(2)
O4	81(2)	93(3)	83(2)	-20.1(18)	13.0(19)	-24(2)
O5	92(2)	103(3)	61.2(19)	-6.6(17)	29.5(17)	23(2)
O6	64.7(18)	75(2)	72.3(19)	-4.0(15)	13.5(15)	16.4(15)
C1	56(2)	57(2)	48.0(19)	0.2(17)	16.0(17)	1.3(18)
C2	63(2)	68(2)	54(2)	7.3(19)	22.0(19)	6(2)
C3	64(3)	62(2)	66(2)	9(2)	24(2)	4(2)
C4	82.5(17)	82.9(17)	88.2(17)	0.9(10)	25.0(10)	0.3(10)
C5A	60(6)	70(7)	65(7)	-1(5)	26(5)	-1(5)
C5B	56(4)	73(5)	83(6)	-2(4)	18(4)	-11(4)
C6	62(3)	100(4)	105(4)	10(3)	29(3)	-12(3)
C7	67(3)	64(3)	62(3)	-5(2)	16(2)	10(2)
C8	127(3)	128(3)	128(3)	-1.8(10)	34.6(12)	-1.5(10)

Table 3 Anisotropic Displacement Parameters ($Å^2×10^3$) for sin21_auto. The Anisotropic displacement factor exponent takes the form: $-2\pi^2[h^2a^{*2}U_{11}+2hka^*b^*U_{12}+…]$.

Atom	U_{11}	U_{22}	U_{33}	U_{23}	U_{13}	U_{12}
C9A	150(6)	150(6)	152(6)	-1(2)	40(3)	-2(2)
C9B	121(13)	121(13)	121(13)	-1(2)	33(4)	0(2)
C10	56(2)	63(2)	55(2)	-3.2(18)	15.5(18)	-2.1(19)
C11	66(3)	75(3)	116(4)	-12(3)	25(3)	13(2)
C12A	64(4)	137(8)	195(10)	-7(7)	60(5)	1(4)
C12B	86(13)	86(13)	86(13)	0(2)	24(4)	0(2)

Table 4 Bond Lengths for sin21_auto.

Atom	Atom	Length/Å	Atom	Atom	Length/Å
S1	O1	1.430(3)	C1	C2	1.532(5)
S1	O2	1.424(3)	C1	C3	1.548(5)
S1	C2	1.767(4)	C1	C7	1.528(6)
S1	C4	1.799(6)	C1	C10	1.517(5)
F1	C6	1.288(7)	C3	C4	1.498(7)
F2	C6	1.284(6)	C4	C5A	1.4196(10)
F3	C6	1.261(7)	C4	C5B	1.437(9)
O3	C7	1.202(5)	C5A	C6	1.466(11)
O4	C7	1.320(6)	C5B	C6	1.517(9)
O4	C8	1.460(8)	C8	C9A	1.4199(11)
O5	C10	1.199(5)	C8	C9B	1.4198(11)
O6	C10	1.314(5)	C11	C12A	1.438(9)
O6	C11	1.463(5)	C11	C12B	1.45(4)

Table 5 Bond Angles for sin21_auto.

Atom	Atom	Atom	Angle/°	Atom	Atom	Atom	Angle/°
O1	S1	C2	108.2(2)	C4	C5A	C6	122.9(8)
O1	S1	C4	108.0(2)	C4	C5B	C6	118.3(6)
O2	S1	O1	119.0(2)	F1	C6	C5A	100.2(7)
O2	S1	C2	111.7(2)	F1	C6	C5B	115.3(5)
O2	S1	C4	112.0(3)	F2	C6	F1	108.2(7)
C2	S1	C4	95.2(2)	F2	C6	C5A	89.1(6)
C7	O4	C8	117.0(5)	F2	C6	C5B	124.0(7)

Table 5 Bond Angles for sin21_auto.

Atom	Atom	Atom	Angle/°	Atom	Atom	Atom	Angle/°
C10	O6	C11	117.7(4)	F3	C6	F1	107.5(6)
C2	C1	C3	106.0(3)	F3	C6	F2	100.9(6)
C7	C1	C2	112.3(4)	F3	C6	C5A	145.6(8)
C7	C1	C3	110.7(3)	F3	C6	C5B	98.0(6)
C10	C1	C2	109.9(3)	O3	C7	O4	124.6(5)
C10	C1	C3	109.5(3)	O3	C7	C1	125.3(5)
C10	C1	C7	108.5(3)	O4	C7	C1	110.1(4)
C1	C2	S1	104.5(3)	C9A	C8	O4	107.2(8)
C4	C3	C1	111.1(4)	C9B	C8	O4	112.5(13)
C3	C4	S1	107.3(3)	O5	C10	O6	125.8(4)
C5A	C4	S1	117.7(6)	O5	C10	C1	123.3(4)
C5A	C4	C3	129.9(6)	O6	C10	C1	110.8(3)
C5B	C4	S1	122.4(5)	C12A	C11	O6	109.2(5)
C5B	C4	C3	117.6(5)	C12B	C11	O6	121.3(15)

Table 6 Hydrogen Atom Coordinates (Å×10^4) and Isotropic Displacement Parameters (Å^2×10^3) for sin21_auto.

Atom	*x*	*y*	*z*	U(eq)
H2A	5021.99	6423.79	441.82	73
H2B	5706.93	7287.32	1373.79	73
H3A	4698.39	4753.86	2113.22	75
H3B	5073.31	5045.82	3720.26	75
H5AA	3127.43	6447.57	3925.2	76
H5AB	3192.29	5284.74	3917.57	76
H5BA	2926.69	4720.14	2448.68	85
H5BB	2597.01	5388.75	1096.46	85
H8AA	7594.93	8126.13	5090.69	153
H8AB	8120.25	7081.75	5630.6	153
H8BC	7936.42	8247.84	4649.23	153
H8BD	7671.11	7435.44	5653.06	153
H9AA	9205.25	7208.36	4177.3	227
H9AB	9383.32	8131.07	5200.55	227
H9AC	8678.07	8244.22	3627.45	227
H9BA	9021.55	6468.61	5392.13	182
H9BB	9479.7	7540.06	5869.37	182

Table 6 Hydrogen Atom Coordinates (Å×10^4) and Isotropic Displacement Parameters (Å^2×10^3) for sin21_auto.

Atom	*x*	*y*	*z*	U(eq)
H9BC	9254.44	7215.71	4284.32	182
H11A	8151.3	3584.66	3338.2	103
H11B	7620.73	3802.09	1723.66	103
H11C	7606.74	3463.11	2504.02	103
H11D	8042.34	4305	1713.83	103
H12A	8762.64	5091.79	1792.02	191
H12B	9458.43	4148.02	2421.79	191
H12C	9240.32	4979.03	3433.02	191
H12D	9573.72	4003.45	3011.09	129
H12E	9104.99	3299.65	3971.88	129
H12F	9216.37	4452.22	4282.14	129
H4A	3490(90)	5770(80)	1670(30)	129
H4B	3820(60)	6100(60)	3450(30)	129

Table 7 Atomic Occupancy for sin21_auto.

Atom	*Occupancy*	Atom	*Occupancy*	Atom	*Occupancy*
C5A	0.395(11)	H5AA	0.395(11)	H5AB	0.395(11)
C5B	0.605(11)	H5BA	0.605(11)	H5BB	0.605(11)
H8AA	0.75(2)	H8AB	0.75(2)	H8BC	0.25(2)
H8BD	0.25(2)	C9A	0.75(2)	H9AA	0.75(2)
H9AB	0.75(2)	H9AC	0.75(2)	C9B	0.25(2)
H9BA	0.25(2)	H9BB	0.25(2)	H9BC	0.25(2)
H11A	0.843(16)	H11B	0.843(16)	H11C	0.157(16)
H11D	0.157(16)	C12A	0.843(16)	H12A	0.843(16)
H12B	0.843(16)	H12C	0.843(16)	C12B	0.157(16)
H12D	0.157(16)	H12E	0.157(16)	H12F	0.157(16)
H4A	0.395(11)	H4B	0.605(11)		

Experimental

Single crystals of $C_{12}H_{17}F_3O_6S$ [sin21_auto] were []. A suitable crystal was selected and [mounted on a nylon loop.] on a ROD, Synergy Custom system, HyPix-Arc 150 diffractometer. The crystal was kept at 293(2) K during data collection. Using Olex2 [1], the structure was solved with the SHELXT [2] structure solution program using Intrinsic Phasing and refined with the SHELXL [3] refinement package using Least Squares minimisation.

1. Dolomanov, O.V., Bourhis, L.J., Gildea, R.J, Howard, J.A.K. & Puschmann, H. (2009), J. Appl. Cryst. 42, 339-341.
2. Sheldrick, G.M. (2015). Acta Cryst. A71, 3-8.
3. Sheldrick, G.M. (2015). Acta Cryst. C71, 3-8.

Crystal structure determination of [sin21_auto]

Crystal Data for $C_{12}H_{17}F_3O_6S$ (*M* =346.31 g/mol): monoclinic, space group P2$_1$/c (no. 14), a = 12.8077(10) Å, b = 13.3519(9) Å, c = 9.8528(8) Å, β = 106.248(8)°, V = 1617.6(2) Å^3, Z = 4, T = 293(2) K, μ(Cu Kα) = 2.319 mm^{-1}, *Dcalc* = 1.422 g/cm^3, 9100 reflections measured (9.778° ≤ 2Θ ≤ 151.974°), 3164 unique (R_{int} = 0.0469, R_{sigma} = 0.0471) which were used in all calculations. The final R_1 was 0.0766 (I > 2σ(I)) and wR_2 was 0.2640 (all data).

Refinement model description

Number of restraints - 35, number of constraints - unknown.

Details:

```
1. Fixed Uiso
 At 1.2 times of:
  All C(H,H) groups, 6H8BD of C8, {H11A,H11B,H11C,H11D}8 of C11
 At 1.5 times of:
  All C(H,H,H) groups, {H12D,H12E,H12F,H4B} of C12B
 At 1.501 times of:
  All C(H) groups
2. Restrained distances
 C4-H4A
 0.9 with sigma of 0.001
 C4-H4B
 0.9 with sigma of 0.001
 C4-C5A = C8-C9A = C8-C9B
 1.42 with sigma of 0.001
3. Uiso/Uaniso restraints and constraints
Uanis(C4) ≈ Ueq, Uanis(C12B) ≈ Ueq, Uanis(C8) ≈ Ueq, Uanis(C9A)
≈ Ueq, Uanis(C9B) ≈ Ueq: with sigma of 0.001 and sigma for terminal
atoms of 0.002
4. Others
 Sof(C5B)=Sof(H5BA)=Sof(H5BB)=Sof(H4B)=1-FVAR(1)
 Sof(C5A)=Sof(H5AA)=Sof(H5AB)=Sof(H4A)=FVAR(1)
 Sof(H11C)=Sof(H11D)=Sof(C12B)=Sof(H12D)=Sof(H12E)=Sof(H12F)=1-FVAR(2)
 Sof(H11A)=Sof(H11B)=Sof(C12A)=Sof(H12A)=Sof(H12B)=Sof(H12C)=FVAR(2)
 Sof(H8BC)=Sof(H8BD)=Sof(C9B)=Sof(H9BA)=Sof(H9BB)=Sof(H9BC)=1-FVAR(3)
 Sof(H8AA)=Sof(H8AB)=Sof(C9A)=Sof(H9AA)=Sof(H9AB)=Sof(H9AC)=FVAR(3)
5.a Secondary CH2 refined with riding coordinates:
 C2(H2A,H2B), C3(H3A,H3B), C5A(H5AA,H5AB), C5B(H5BA,H5BB), C8(H8AA,H8AB),
 C8(H8BC,H8BD), C11(H11A,H11B), C11(H11C,H11D)
5.b Idealised Me refined as rotating group:
 C9A(H9AA,H9AB,H9AC), C9B(H9BA,H9BB,H9BC), C12A(H12A,H12B,H12C), C12B(H12D,
 H12E,H12F)
```

This report has been created with Olex2, compiled on 2022.04.12 svn.rca3783a0 for Rigaku Oxford Diffraction. Please let us know if there are any errors or if you would like to have additional features.

5.0 Experimental Data for Flouroalkyl Sulfonylation Compounds:

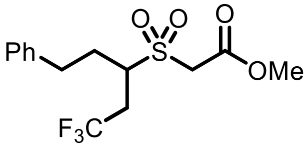

Methyl 2-((1,1,1-trifluoro-5-phenylpentan-3-yl)sulfonyl)acetate (3): Following the general method of fluoroalkyl sulfonylalkylation 2.4, the title compound **3** was obtained as a transparent oil (118 mg, 70% yield) after purification by silica gel flash chromatography (hexanes:EtOAc = 20:1 to 1:2). The reaction mixture was irradiated for 12 h.

^{1}H NMR (400 MHz, $CDCl_3$): δ 7.37 – 7.27 (m, 2H), 7.27 – 7.15 (m, 3H), 4.10 – 3.93 (m, 2H), 3.79 (s, 3H), 3.02 – 2.87 (m, 2H), 2.86 – 2.76 (m, 1H), 2.64 – 2.48 (m, 1H), 2.46 – 2.32 (m, 1H), 2.25 – 2.10 (m, 1H); **^{13}C NMR (101 MHz, $CDCl_3$):** δ 162.9, 139.7, 128.7, 128.4, 126.5, 125.5 (q, $^1J_{CF}$ = 278.1 Hz), 56.2 (q, $^3J_{CF}$ = 2.33 Hz), 55.7, 53.4, 32.5 (q, $^2J_{CF}$ = 31.80 Hz), 32.0, 30.1; **^{19}F NMR (376 MHz, $CDCl_3$):** δ -63.5; **IR (neat, ν_{max}):** 3010, 1746, 1322, 1263, 1117, 700 cm^{-1}; **HRMS-ESI** (*m/z*): Calcd. for $C_{14}H_{17}F_3O_4SNa$ $[M+Na]^+$ 361.0692, found 361.0696.

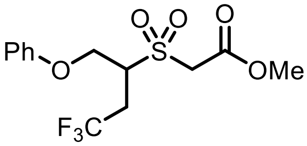

Methyl 2-((4,4,4-trifluoro-1-phenoxybutan-2-yl)sulfonyl)acetate (6): Following the general method of fluoroalkyl sulfonylalkylation 2.4, the title compound **6** was obtained as a transparent oil (104 mg, 61% yield) after purification by silica gel flash chromatography (hexanes:EtOAc = 20:1 to 1:2). The reaction mixture was irradiated for 12 h.

^{1}H NMR (400 MHz, $CDCl_3$): δ 7.38 – 7.27 (m, 2H), 7.05 (td, *J* = 7.3, 1.1 Hz, 1H), 6.95 – 6.87 (m, 2H), 4.52 (dd, J = 10.7, 3.1 Hz, 1H), 4.46 – 4.38 (m, 2H), 4.25 – 4.13 (m, 2H), 3.82 (s, 3H), 3.18 – 3.01 (m, 1H), 2.68 – 2.48 (m, 1H); **^{13}C NMR (101 MHz, $CDCl_3$):** δ 163.1, 129.8, 125.5 (q, $^1J_{CF}$ = 277.2 Hz) 122.4, 114.6, 65.2, 58.53, 57.18 (q, $^3J_{CF}$ = 2.4 Hz), 53.3, 28.5 (q, $^2J_{CF}$ = 31.4 Hz); **^{19}F NMR (376 MHz, $CDCl_3$):** δ -63.70; **IR (neat, ν_{max}):** 3621, 3463, 1748, 1317, 1120 cm^{-1}; **HRMS-ESI** (*m/z*): Calcd. for $C_{13}H_{15}F_3O_5SNa$ $[M+Na]^+$ 363.0485, found 363.0486.

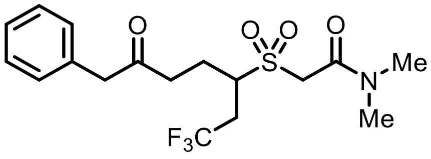

***Methyl 2-((1,1,1-trifluoro-4-oxo-5-phenylpentan-3-yl)sulfonyl)acetate (7)*:** Following the general method of fluoroalkyl sulfonylalkylation 2.4, the title compound **7** was obtained as a transparent oil (115 mg, 65% yield) after purification by silica gel flash chromatography (hexanes:EtOAc = 20:1 to 1:2). The reaction mixture was irradiated for 12 h.

^{1}H NMR (400 MHz, $CDCl_3$): δ 7.38 – 7.22 (m, 3H), 7.22 – 7.11 (m, 2H), 4.22 (d, *J* = 14.9 Hz, 1H), 4.09 (d, *J* = 14.8 Hz, 1H), 3.92 – 3.82 (m, 1H), 3.71 (s, 2H), 3.12 (s, 3H), 2.99 (s, 3H), 2.96 – 2.64 (m, 3H), 2.46 – 2.18 (m, 3H); **^{13}C NMR (101 MHz, $CDCl_3$):** δ 206.4, 161.6, 133.7, 129.4, 128.7, 127.1, 125.6 (q, $^1J_{CF}$ = 277.6 Hz). 56.2 (d, $^3J_{CF}$ = 2.4 Hz), 54.0, 49.9, 38.5, 37.5, 36.0, 31.7 (q, $^2J_{CF}$ = 30.5 Hz), 21.9; **^{19}F NMR (376 MHz, $CDCl_3$):** δ -63.3; **IR (neat, ν_{max}):** 2947, 1714, 1647, 1311, 1117 cm^{-1}; **HRMS-ESI** (*m/z*): Calcd. for $C_{17}H_{23}F_3NO_4S$ $[M+H]^+$ 394.1294, found 394.1284.

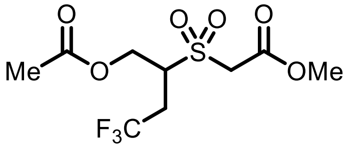

***Methyl 2-((1-acetoxy-4,4,4-trifluorobutan-2-yl)sulfonyl)acetate (8)*:** Following the general method of fluoroalkyl sulfonylalkylation 2.4, the title compound **8** was obtained as a transparent oil (74 mg, 48% yield) after purification by silica gel flash chromatography (hexanes:EtOAc = 20:1 to 1:2). The reaction mixture was irradiated for 12 h.

^{1}H NMR (400 MHz, $CDCl_3$): δ 4.78 (dd, J = 12.8, 3.4 Hz, 1H), 4.43 – 4.29 (m, 2H), 4.13 – 4.00 (m, 2H), 3.84 (s, 3H), 3.07 – 2.89 (m, 1H), 2.63 – 2.44 (m, 1H), 2.11 (s, 3H); **^{13}C NMR (101 MHz, $CDCl_3$):** δ 169.8, 163.1, 125.4 (q, $^1J_{CF}$ = 277.1 Hz), 60.3, 57.4, 56.0 (q, $^3J_{CF}$ = 2.4 Hz), 53.6, 28.8 (q, $^2J_{CF}$ = 31.4 Hz), 20.6; **^{19}F NMR (376 MHz, $CDCl_3$):** δ -63.84; **IR (neat, ν_{max}):** 2985, 1745, 1369, 1125, 1004 cm^{-1}; **HRMS-ESI** (*m/z*): Calcd. for $C_9H_{13}F_3O_6SNa$ $[M+Na]^+$ 329.0277, found 329.0279.

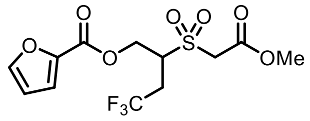

***Methyl 2-((4-(trifluoromethyl)hexan-3-yl)sulfonyl)acetate (9)*:** Following the general method of fluoroalkyl sulfonylalkylation 2.4, the title compound **9** was obtained as a transparent oil (73 mg, 41% yield) after purification by silica gel flash chromatography (hexanes:EtOAc = 20:1 to 1:2). The reaction mixture was irradiated for 12 h.

^{1}H NMR (400 MHz, $CDCl_3$): δ 7.66 – 7.54 (m, 1H), 7.27 (dd, *J* = 3.5, 0.9 Hz, 1H), 6.55 (dd, *J* = 3.5, 1.7 Hz, 1H), 4.95 (dd, *J* = 12.5, 3.4 Hz, 1H), 4.67 – 4.49 (m, 2H), 4.28 – 4.20 (m, 1H), 4.14 (d, *J* = 15.4 Hz, 1H), 3.79 (s, 3H), 3.13 – 2.97 (m, 1H), 2.62 – 2.47 (m, 1H); **^{13}C NMR (101 MHz, $CDCl_3$):** δ 163.2, 157.0, 147.0, 143.4, 125.4 (q, $^1J_{CF}$ = 277.2 Hz), 119.5, 112.3, 61.0, 57.8, 56.0 (q, $^3J_{CF}$ = 2.3 Hz), 53.4, 28.7 (q, $^2J_{CF}$ = 31.4 Hz); **^{19}F NMR (376 MHz, $CDCl_3$):** δ -63.8; **IR (neat, ν_{max}):** 2917, 1733, 1326, 1257, 1077 cm^{-1}; **HRMS-ESI** (*m/z*): Calcd. for $C_{12}H_{13}F_3O_7SNa$ $[M+Na]^+$ 381.0226, found 381.0215.

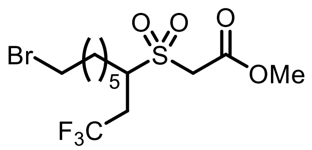

Methyl 2-((7-bromo-1,1,1-trifluoroheptan-3-yl)sulfonyl)acetate (10): Following the general method of fluoroalkyl sulfonylalkylation 2.4, the title compound **10** was obtained as a transparent oil (119 mg, 60% yield) after purification by silica gel flash chromatography (hexanes:EtOAc = 20:1 to 1:2). The reaction mixture was irradiated for 12 h.

^{1}H NMR (400 MHz, CDCl$_3$): δ 4.05 (d, J = 1.4 Hz, 2H), 3.83 (s, 3H), 3.77 – 3.67 (m, 1H), 3.41 (t, J = 6.7 Hz, 2H), 3.00 – 2.81 (m, 1H), 2.54 – 2.37 (m, 1H), 2.11 – 1.98 (m, 1H), 1.93 – 1.79 (m, 3H), 1.59 – 1.42 (m, 4H), 1.43 – 1.32 (m, 2H); **^{13}C NMR (101 MHz, CDCl$_3$):** δ 163.1, 125.5 (q, $^1J_{CF}$ = 277.1 Hz), 56.7 (q, $^3J_{CF}$ = 2.4 Hz), 55.8, 53.5, 33.7, 32.4 (q, $^2J_{CF}$ = 30.2 Hz), 28.4, 28.1, 27.6, 25.7; **^{19}F NMR (376 MHz, CDCl$_3$):** δ -63.8; **IR (neat, ν_{max}):** 2938, 1743, 1319, 1257, 1115 cm^{-1}. **HRMS-ESI** (*m/z*): Calcd. for $C_{12}H_{19}BrF_3O_4S$ [M-H]$^-$ 395.0145, found 395.0142.

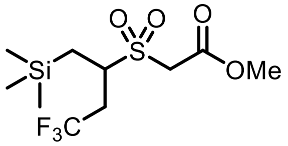

Methyl 2-((4,4,4-trifluoro-1-(trimethylsilyl)butan-2-yl)sulfonyl)acetate (11): Following the general method of fluoroalkyl sulfonylalkylation 2.4, the title compound **11** was obtained as a transparent oil (64 mg, 40% yield) after purification by silica gel flash chromatography (hexanes:EtOAc = 20:1 to 1:2). The reaction mixture was irradiated for 12 h.

^{1}H NMR (400 MHz, CDCl$_3$): δ 4.04 (s, 2H), 3.90 – 3.72 (m, 4H), 3.05 – 2.85 (m, 1H), 2.49 – 2.31 (m, 1H), 1.38 (dd, J = 15.0, 4.0 Hz, 1H), 1.01 (dd, J = 15.1, 10.0 Hz, 1H), 0.14 (s, 9H); **^{13}C NMR (101 MHz, CDCl$_3$):** δ 163.2, 125.5 (q, $^1J_{CF}$ = 277.3 Hz), 54.8 (q, $^3J_{CF}$ = 2.8 Hz), 53.4, 35.1 (q, $^2J_{CF}$ = 30.1 Hz), 16.1, -1.2; **^{19}F NMR (376 MHz, CDCl$_3$):** δ -63.3; **IR (neat, ν_{max}):** cm^{-1}. **HRMS-ESI** (*m/z*): Calcd. for $C_{10}H_{19}F_3O_4SSiNa$ [M+Na]$^+$ 343.0618, found 343.0618.

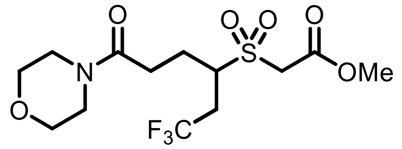

***Methyl 2-((1,1,1-trifluoro-6-morpholino-6-oxohexan-3-yl)sulfonyl)acetate (12)*:** Following the general method of fluoroalkyl sulfonylalkylation 2.4, the title compound **12** was obtained as a transparent oil (116 mg, 62% yield) after purification by silica gel flash chromatography (hexanes:EtOAc = 20:1 to 1:9). The reaction mixture was irradiated for 12 h.

^{1}H NMR (400 MHz, $CDCl_3$): δ 4.33 (d, J = 15.0 Hz, 1H), 4.09 (d, J = 15.0 Hz, 1H), 3.91–3.78 (m, 4H), 3.68 – 3.64 (m, 4H), 3.62 – 3.52 (m, 2H), 3.48 – 3.41 (m, 2H), 3.05 – 2.88 (m, 1H), 2.68 – 2.38 (m, 4H), 2.28 – 2.17 (m, 1H); **^{13}C NMR (101 MHz, $CDCl_3$):** δ 169.5, 163.1, 125.7 (q,$^1J_{CF}$ = 278.8 Hz), 66.7, 66.4, 56.1, 56.1, 56.1 (q,$^3J_{CF}$ = 2.52 Hz), 45.6, 42.1, 30.5 (q,$^2J_{CF}$ = 31.80 Hz), 28.5, 24.0; **^{19}F NMR (376 MHz, $CDCl_3$):** δ -63.5; **IR (neat, ν_{max}):** 2960, 1743, 1634, 1438cm^{-1}; **HRMS-ESI** (*m/z*): Calcd. For $C_{13}H_{19}F_3NO_6S$ [M-H]$^-$ 374.0891, found 374.0890.

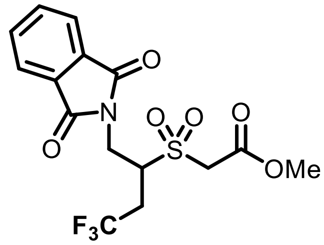

***Methyl 2-((1-(1,3-dioxoisoindolin-2-yl)-4,4,4-trifluorobutan-2-yl)sulfonyl)acetate--methane (13)*:** Following the general method of fluoroalkyl sulfonylalkylation 2.4, the title compound **13** was obtained as a white solid (117 mg, 57% yield) after purification by silica gel flash chromatography (hexanes:EtOAc = 20:1 to 1:2). The reaction mixture was irradiated for 12 h.

^{1}H NMR (400 MHz, $CDCl_3$): δ 7.94 – 7.71 (m, 4H), 4.56 – 4.14 (m, 5H), 3.84 (s, 3H), 3.15 – 2.97 (m, 1H), 2.69 (tt, J = 10.2, 8.4 Hz, 1H); **^{13}C NMR (101 MHz, $CDCl_3$):** δ 168.0, 162.9, 134.6, 131.5, 125.4 (q, $^1J_{CF}$ = 276.9 Hz). 123.8, 56.0, 55.3 (q, $^3J_{CF}$ = 2.4 Hz), 53.6, 36.8 (q, $^2J_{CF}$ = 31.3

Hz); **^{19}F NMR (376 MHz, $CDCl_3$):** δ -64.6; **IR (neat, ν_{max}):** 2960, 1774, 1746, 1705, 1302, 1194 cm^{-1}; **HRMS-ESI** (*m/z*): Calcd. for $C_{15}H_{15}F_3NO_6S$ $[M+H]^+$ 394.0567, found 394.0566.

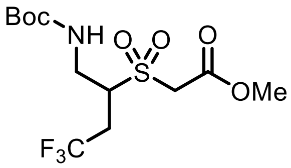

***Methyl 2-((5-((tert-butoxycarbonyl)amino)-1,1,1-trifluoropentan-3-yl)sulfonyl)acetate (14)*:** Following the general method of fluoroalkyl sulfonylalkylation 2.4, the title compound **14** was obtained as a white solid (130 mg, 69% yield) after purification by silica gel flash chromatography (hexanes:EtOAc = 20:1 to 1:3). The reaction mixture was irradiated for 12 h.

^{1}H NMR (400 MHz, $CDCl_3$): δ 5.25- 5.04(t, J = 6.5 Hz, 1H), 4.36 (d, J = 15.3 Hz, 1H), 4.08 (d, J = 15.2 Hz, 1H), 4.02 – 3.92 (m, 1H), 3.91 – 3.65 (m, 5H), 3.00 – 2.82 (m, 1H), 2.55 (s, 1H), 1.42 (s, 9H); **^{13}C NMR (101 MHz, $CDCl_3$):** δ 163.0, 155.9, 125.55 (q,$^1J_{CF}$ = 277.2 Hz), 80.5, 57.0, 56.6, 53.4, 39.3, 29.8 (q, $^2J_{CF}$ = 30.9 Hz), 28.2 ; **^{19}F NMR (376 MHz, $CDCl_3$):** δ -63.8 ; **IR (neat, ν_{max}):** 3364, 2987, 1746, 1686, 1529, 1145 cm^{-1}; **HRMS-ESI** (*m/z*): Calcd. for $C_{13}H_{22}F_3NO_6SNa$ $[M+Na]^+$ 386.0856, found 386.0859.

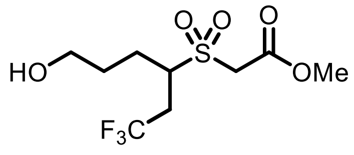

***Methyl 2-(1,1,1-trifluoro-6-hydroxyhexan-3-yl)sulfonyl)acetate (15)*:** Following the general method of fluoroalkyl sulfonylalkylation 2.4, the title compound **15** was obtained as a transparent oil (86 mg, 59% yield) after purification by silica gel flash chromatography (hexanes:EtOAc = 20:1 to 1:2). The reaction mixture was irradiated for 12 h.

^{1}H NMR (400 MHz, $CDCl_3$): δ 4.13 (d, J = 15.0 Hz, 1H), 4.04 (d, J = 15.0 Hz, 1H), 3.90 – 3.76 (m, 4H), 3.77 – 3.65 (m, 2H), 3.02 – 2.85 (m, 1H), 2.58 – 2.39 (m, 1H), 2.28 – 2.14 (m, 1H), 2.06 – 1.92 (m, 1H), 1.92 – 1.69 (m, 2H), 1.59 (s, 1H); **^{13}C NMR (101 MHz, $CDCl_3$):** δ 163.2, 125.6

(q, $^{1}J_{CF}$ = 277.2 Hz), 61.6, 56.5 (q, $^{3}J_{CF}$ = 2.3 Hz), 55.6, 53.5, 32.3 (q, $^{2}J_{CF}$ = 30.4 Hz), 28.5, 24.9; **^{19}F NMR (376 MHz, CDCl$_3$):** δ -63.8; **IR (neat, ν_{max}):** 3549, 3030, 1749, 1320, 1121 cm^{-1}; **HRMS-ESI** (*m/z*): Calcd. for $C_9H_{15}F_3O_5SNa$ [M+Na]$^+$ 315.0485, found 315.0488.

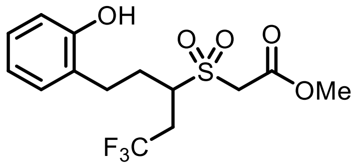

***Methyl 2-((1,1,1-trifluoro-5-(2-hydroxyphenyl)pentan-3-yl)sulfonyl)acetate (16)*:** Following the general method of fluoroalkyl sulfonylalkylation 2.4, the title compound **16** was obtained as a transparent oil (96 mg, 54% yield) after purification by silica gel flash chromatography (hexanes:EtOAc = 20:1 to 1:2). The reaction mixture was irradiated for 12 h.

^{1}H NMR (400 MHz, CDCl$_3$): δ 7.24 – 7.14 (m, 2H), 6.95 – 6.87 (m, 1H), 6.81 (dd, *J* = 8.0, 1.2 Hz, 1H), 5.85 (s, 1H), 4.48 – 4.36 (m, 1H), 3.82 – 3.71 (m, 4H), 3.53 (dd, *J* = 15.0, 1.3 Hz, 1H), 3.32 – 3.19 (m, 2H), 3.15 – 3.00 (m, 1H), 2.49 – 2.35 (m, 1H); **^{13}C NMR (101 MHz, CDCl$_3$):** δ 163.3, 154.2, 131.8, 129.5, 125.70 (q, $^{1}J_{CF}$ = 277.2 Hz) 121.9, 121.3, 116.1, 57.2, 56.3 (q, $^{3}J_{CF}$ = 2.3 Hz), 53.4, 31.5 (q, $^{2}J_{CF}$ = 30.8 Hz), 31.3; **^{19}F NMR (376 MHz, CDCl$_3$):** δ -63.7; **IR (neat, ν_{max}):** 3622, 3463, 1748, 1317, 1140 cm^{-1}; **HRMS-ESI** (*m/z*): Calcd. for $C_{14}H_{17}F_3O_5SNa$ [M+Na]$^+$ 363.0485, found 363.0486.

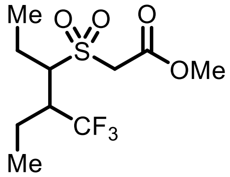

***Methyl 2-((4-(trifluoromethyl)hexan-3-yl)sulfonyl)acetate (17)*:** Following the general method of fluoroalkyl sulfonylalkylation 2.4, the title compound **17** was obtained as a transparent oil (71 mg, 49% yield) after purification by silica gel flash chromatography (hexanes:EtOAc = 20:1 to 1:1). The reaction mixture was irradiated for 12 h. Major isomer was isolated as a mixture of separable diastereomers in a 9:1 ratio as determined in a crude reaction mixture by ^{1}H NMR.

^{1}H NMR (400 MHz, CDCl$_3$): δ 4.07 – 3.96 (m, 2H), 3.86 – 3.75 (m, 4H), 3.03 – 2.88 (m, 1H), 2.14 – 1.97 (m, 1H), 1.98 – 1.84 (m, 1H), 1.86 – 1.64 (m, 2H), 1.18 (t, J = 7.5 Hz, 3H), 1.09 (t, J = 7.5 Hz, 3H); **^{13}C NMR (101 MHz, CDCl$_3$):** δ 163.1, 127.3 (q, $^{1}J_{CF}$ = 281.5 Hz), 62.6 (q, $^{3}J_{CF}$ = 2.8 Hz), 58.0, 53.4, 43.2 (q, $^{2}J_{CF}$ = 25.7 Hz), 18.1, 17.8 (q, $^{3}J_{CF}$ = 1.8 Hz), 13.2, 12.7 (q, $^{3}J_{CF}$ = 1.1 Hz); **^{19}F NMR (376 MHz, CDCl$_3$):** δ -67.1; **IR (neat, ν_{max}):** 2956, 1744, 1317, 1173, 1107 cm^{-1}; **HRMS-ESI** (*m/z*): Calcd. for $C_{10}H_{17}F_3O_4SNa$ [M+Na]$^+$ 313.0692, found 313.0696.

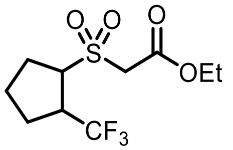

***Methyl 2-((2-(trifluoromethyl)cyclopentyl)sulfonyl)acetate (18)[9]*:** Following the general method of fluoroalkyl sulfonylalkylation 2.4, the title compound **18** was obtained as a transparent oil (75 mg, 55% yield) after purification by silica gel flash chromatography (hexanes:EtOAc = 20:1 to 1:1). The reaction mixture was irradiated for 12 h.

^{1}H NMR (400 MHz, CDCl$_3$): δ 4.28 (q, J = 7.1 Hz, 2H), 4.15 – 4.06 (m, 1H), 4.06 – 3.95 (m, 2H), 3.36 – 3.19 (m, J = 4.8 Hz, 1H), 2.36 – 2.23 (m, 1H), 2.24 – 2.03 (m, 2H), 2.01 – 1.76 (m, 3H), 1.31 (t, J = 7.1 Hz, 3H); **^{13}C NMR (101 MHz, CDCl$_3$):** δ 162.6, 127.1 (q, $^{1}J_{CF}$ = 278.3 Hz), 62.8, 62.0 (q, $^{3}J_{CF}$ = 2.0 Hz), 44.0 (q, $^{2}J_{CF}$ = 28.3 Hz),28.2, 27.7 (q, $^{3}J_{CF}$ = 2.2 Hz), 25.6, 13.9; **^{19}F NMR (376 MHz, CDCl$_3$):** δ -70.2; **IR (neat, ν_{max}):** 2982, 1742, 1314, 1141, 1065 cm^{-1}; **HRMS-ESI** (*m/z*): Calcd. for $C_9H_{13}F_3O_4SNa$ [M+Na]$^+$ 311.0535, found 311.0539.

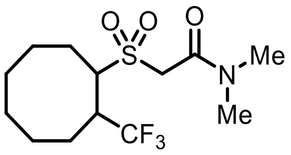

***N,N-dimethyl-2-((2-(trifluoromethyl)cyclooctyl)sulfonyl)acetamide (19)*:** Following the general method of fluoroalkyl sulfonylalkylation 2.4, the title compound **19** was obtained as a transparent oil (82 mg, 50% yield) after purification by silica gel flash chromatography (hexanes:EtOAc =

20:1 to 1:5). The reaction mixture was irradiated for 12 h. Compound was isolated as a mixture of inseparable diastereomers in a 1:1 ratio as determined in a crude reaction mixture by ^{1}H NMR.

^{1}H NMR (400 MHz, CDCl$_3$): δ 4.13 – 4.01 (m, 2H), 3.66 – 3.49 (m, 1H), 3.18 (s, 3H), 3.01 (s, 3H), 2.47 – 2.36 (m, 1H), 2.36 – 2.12 (m, 3H), 2.09 – 1.93 (m, 2H), 1.92 – 1.74 (m, 4H), 1.72 – 1.42 (m, 4H); **^{13}C NMR (101 MHz, CDCl$_3$):** δ 162.2, 162.1, 128.3 (q, $^{1}J_{CF}$ = 280.0 Hz), 128.3 (q, $^{1}J_{CF}$ = 280.0 Hz), 61.3, 60.3, 53.4, 53.3, 42.12 (q, $^{2}J_{CF}$ = 24.9 Hz), 41.50 (q, $^{2}J_{CF}$ = 24.7 Hz), 38.76, 36.14, 25.41, 25.39, 25.30, 25.14 (q, $^{3}J_{CF}$ = 2.5 Hz), 24.85, 24.74, 24.63, 24.6(1), 24.6(0), 24.21, 23.73, 22.94. 22.74 (q, $^{3}J_{CF}$ = 2.9 Hz); **^{19}F NMR (376 MHz, CDCl$_3$):** δ -72.9, -73.3; **IR (neat, ν_{max}):** 2942, 1643, 1302, 1134, 1070 cm^{-1}; **HRMS-ESI** (*m/z*): Calcd. for $C_{13}H_{23}F_3NO_3S$ [M+H]$^{+}$ 330.1345, found 330.1336.

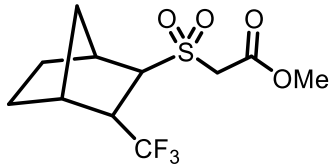

***Methyl 2-(((1S,3S,4R)-3-(trifluoromethyl)bicyclo[2.2.1]heptan-2-yl)sulfonyl)acetate (20)*:** Following the general method of fluoroalkyl sulfonylalkylation 2.4, the title compound **20** was obtained as a transparent oil (113 mg, 75% yield) after purification by silica gel flash chromatography (hexanes:EtOAc = 20:1 to 1:1). The reaction mixture was irradiated for 12 h.

^{1}H NMR (400 MHz, CDCl$_3$): δ 4.12 – 4.02 (m, 2H), 3.90 (d, J = 15.1 Hz, 1H), 3.81 (s, 3H), 3.00 – 2.92 (m, 1H), 2.82 – 2.72 (m, 1H), 2.66 (d, J = 4.6 Hz, 1H), 2.14 – 2.05 (m, 1H), 1.82 – 1.67 (m, 2H), 1.60 – 1.41 (m, 3H); **^{13}C NMR (101 MHz, CDCl$_3$):** δ 163.5, 126.4 (q, $^{1}J_{CF}$ = 278.5 Hz), 63.6 (q, J_{CF} = 1.8 Hz), 58.0 (q, J_{CF} = 2.2 Hz), 53.32, 48.1 (q, $^{2}J_{CF}$ = 28.0 Hz), 39.7, 39.4 (q, J_{CF} = 1.8 Hz), 38.3 (q, J_{CF} = 1.5 Hz), 28.3, 23.6; **^{19}F NMR (376 MHz, CDCl$_3$):** δ -68.4; **IR (neat, ν_{max}):** 2885, 1745, 1248, 1234, 1032 cm^{-1}; **HRMS-ESI** (*m/z*): Calcd. for $C_{11}H_{15}F_3O_4SNa$ [M+Na]$^{+}$ 323.0535, found 323.0540.

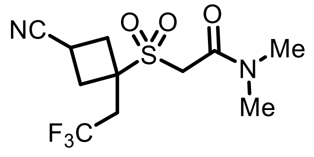

***2-((3-cyano-1-(2,2,2-trifluoroethyl)cyclobutyl)sulfonyl)-N,N-dimethylacetamide(21)*:** Following the general method of fluoroalkyl sulfonylalkylation 2.4, the title compound **21** was obtained as a transparent oil (79 mg, 51% yield) after purification by silica gel flash chromatography (hexanes:EtOAc = 20:1 to 1:5). The reaction mixture was irradiated for 12 h. Compound was isolated as a mixture of inseparable diastereomers in a 1.5:1 ratio as determined in a crude reaction mixture by ^{1}H NMR.

^{1}H NMR (400 MHz, CDCl$_3$): δ 4.17– 4.11 (m, 1.42H), 3.83 (s, 0.57H), 3.34 (q, J = 9.0 Hz, 0.72H), 3.28 – 2.85 (m, 11.6H), 2.84 – 2.74 (m, 0.68H); **^{13}C NMR (101 MHz, CDCl$_3$):** δ 166.6, 161.0, 160.9, 125.5 (q, $^1J_{CF}$ = 278.2 Hz), 125.1 (q, $^1J_{CF}$ = 278.3 Hz), 120.3, 119.9, 61.7 (q, $^3J_{CF}$ = 2.1 Hz), 60.5 (q, $^3J_{CF}$ = 1.9 Hz), 53.3, 52.9, 38.7, 38.0, 37.1 (q, $^2J_{CF}$ = 29.3 Hz), 36.1, 36.0, 35.9, 35.6 (q, $^2J_{CF}$ = 29.4 Hz), 31.4, 30.4, 26.1, 16.8, 16.0; **^{19}F NMR (376 MHz, CDCl$_3$):** δ -59.5, -60.2; **IR (neat, ν_{max}):** 2941, 2245, 1635, 1398, 1134 cm^{-1}; **HRMS-ESI** (*m/z*): Calcd. for $C_{11}H_{14}F_3N_2O_3S$ [M-H]$^-$ 311.0683, found 311.0682.

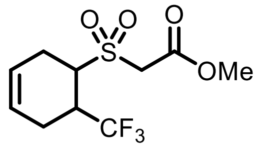

***Methyl 2-((6-(trifluoromethyl)cyclohex-3-en-1-yl)sulfonyl)acetate (22)*:** Following the general method of fluoroalkyl sulfonylalkylation 2.4, the title compound **22** was obtained as a transparent oil (63 mg, 44% yield) after purification by silica gel flash chromatography (hexanes:EtOAc = 20:1 to 1:1). The reaction mixture was irradiated for 12 h.

^{1}H NMR (400 MHz, CDCl$_3$): δ 5.86 – 5.65 (m, 2H), 4.12 (d, *J* = 14.7 Hz, 1H), 4.08 – 3.97 (m, 2H), 3.82 (s, 3H), 3.35 – 3.17 (m, 1H), 2.71 – 2.51 (m, 3H), 2.42 – 2.30 (m, 1H); **^{13}C NMR (101**

MHz, CDCl3): δ 163.11, 126.66 (q, $^{1}J_{CF}$ = 280.7 Hz), 123.80, 122.04, 56.35, 54.33 (q, $^{3}J_{CF}$ = 2.2 Hz), 53.46, 34.54 (q, $^{2}J_{CF}$ = 27.8 Hz), 21.21, 20.52 (q, $^{3}J_{CF}$ = 2.4 Hz); **^{19}F NMR (376 MHz, CDCl3):** δ -70.1; **IR (neat, ν_{max}):** 2985, 1746, 1669, 1320, 1150 cm^{-1}. **HRMS-ESI** (*m/z*): Calcd. for $C_{10}H_{13}F_3O_4SNa$ [M+Na]$^+$ 309.0379, found 309.0381.

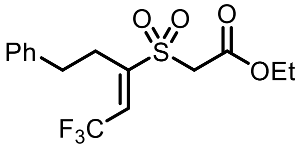

***Ethyl (E)-2-(1,1,1-trifluoro-5-phenylpent-2-en-3-yl)sulfonyl)acetate (23)*:** Following the general method of fluoroalkyl sulfonylalkylation 2.4, the title compound **23** was obtained as a transparent oil (88 mg, 50 % yield) after purification by silica gel flash chromatography (hexanes:EtOAc = 20:1 to 1:1). The reaction mixture was irradiated for 12 h.

^{1}H NMR (400 MHz, CDCl3): δ 7.40 – 7.28 (m, 2H), 7.25 (dd, *J* = 7.5, 5.8 Hz, 3H), 6.82 (q, *J* = 7.8 Hz, 1H), 4.23 (q, *J* = 7.2 Hz, 2H), 4.04 (s, 2H), 3.06 – 2.85 (m, 4H), 1.29 (dd, *J* = 7.8, 6.6 Hz, 3H); **^{13}C NMR (101 MHz, CDCl3):** δ 161.6, 151.14 (q, $^{1}J_{CF}$ = 5.0 Hz), 139.7, 128.9 (d, $^{2}J_{CF}$ = 36.5 Hz), 128.7, 128.4, 126.8, 121.84 (q, $^{1}J_{CF}$ = 273.4 Hz), 62.9, 57.7, 35.47 (d, $^{1}J_{CF}$ = 1.8 Hz), 30.3, 13.8; **^{19}F NMR (376 MHz, CDCl3):** δ -59.8; **IR (neat, ν_{max}):** 3068,1744, 1664, 1368, 1135 cm^{-1}; **HRMS-ESI** (*m/z*): Calcd. for $C_{15}H_{16}F_3O_4S$ [M-H]$^-$ 349.0727, found 349.0723.

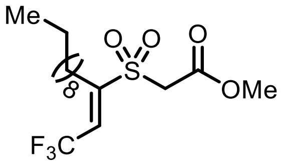

***Methyl (E)-2-((1,1,1-trifluorohex-2-en-3-yl)sulfonyl)acetate (24)*:** Following the general method of fluoroalkyl sulfonylalkylation 2.4, the title compound **24** was obtained as a transparent oil (86 mg, 46% yield) after purification by silica gel flash chromatography (hexanes:EtOAc = 20:1 to 1:1). The reaction mixture was irradiated for 12 h.

^{1}H NMR (400 MHz, CDCl$_3$): δ 6.73 (q, J = 7.8 Hz, 1H), 4.08 (s, 2H), 3.79 (s, 3H), 2.60 (ddt, *J* = 11.0, 7.6, 1.6 Hz, 2H), 1.72 – 1.60 (m, 2H), 1.45 – 1.18 (m, 15H), 0.88 (t, *J* = 6.8 Hz, 3H); **^{13}C NMR (101 MHz, CDCl$_3$):** δ 162.1, 152.2 (q, $^{3}J_{CF}$ = 4.8 Hz), 128.3 (q, $^{2}J_{CF}$ = 36.4 Hz), 121.8 (q, $^{1}J_{CF}$ = 273.2 Hz), 57.8, 53.4, 31.8, 29.8, 29.5, 29.4, 29.3, 28.9, 28.0, 22.6, 14.1; **^{19}F NMR (376 MHz, CDCl3):** δ -59.9; **IR (neat, ν_{max}):** 2925, 1748, 1332, 1871, 1136 cm^{-1} **HRMS-ESI** (*m/z*): Calcd. for $C_{16}H_{26}F_3O_4S$ [M-H]$^-$ 371.1509, found 371.1511.

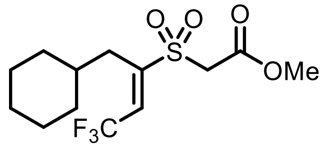

***Methyl (E)-2-((1-cyclohexyl-4,4,4-trifluorobut-2-en-2-yl)sulfonyl)acetate (25)*:** Following the general method of fluoroalkyl sulfonylalkylation 2.4, the title compound **25** was obtained as a transparent oil (66 mg, 40% yield) after purification by silica gel flash chromatography (hexanes:EtOAc = 20:1 to 1:1). The reaction mixture was irradiated for 12 h.

^{1}H NMR (400 MHz, CDCl$_3$): δ 6.79 (q, *J* = 7.8 Hz, 1H), 4.06 (s, 2H), 3.79 (s, 3H), 2.63 – 2.50 (m, 2H), 1.81 – 1.65 (m, 6H), 1.32 – 1.07 (m, 4H), 1.03 – 0.93 (m, 2H) ; **^{13}C NMR (101 MHz, CDCl$_3$):** δ 162.2, 150.8 (q, $^{3}J_{CF}$ = 5.0 Hz), 129.2 (q, $^{2}J_{CF}$ = 36.2 Hz), 121.8 (q, $^{1}J_{CF}$ = 273.4 Hz), 58.3, 53.4, 36.9, 35.2, 33.0, 26.1, 26.0; **^{19}F NMR (376 MHz, CDCl$_3$):** δ -59.0; **IR (neat, ν_{max}):** 2922, 2795, 1741, 1664, 1280, 1116 cm^{-1}; **HRMS-ESI** (*m/z*): Calcd. for $C_{13}H_{19}F_3O_4SNa$ [M+Na]$^+$ 351.0848, found 351.0851.

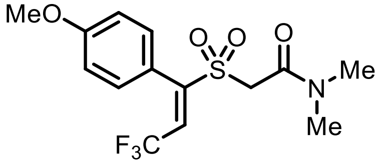

***(E)-N,N-dimethyl-2-((3,3,3-trifluoro-1-(4-methoxyphenyl)prop-1-en-1-yl)sulfonyl)acetamide (26)*:** Following the general method of fluoroalkyl sulfonylalkylation 2.4, the title compound **26**

was obtained as a light yellowish solid (70 mg, 40% yield) after purification by silica gel flash chromatography (hexanes:EtOAc = 20:1 to 1:9). The reaction mixture was irradiated for 12 h.

^{1}H NMR (400 MHz, CDCl$_3$): δ 7.54 (d, J = 8.8 Hz, 2H), 7.00 – 6.88 (m, 3H), 3.92 (s, 2H), 3.85 (s, 3H), 3.07 (s, 3H), 2.99 (s, 3H); **^{13}C NMR (101 MHz, CDCl$_3$):** δ 161.4, 161.0, 150.3 (d, $^3J_{CF}$ = 5.2 Hz)., 127.1 (q, $^2J_{CF}$ = 35.7 Hz), 121.53 (q, $^1J_{CF}$ = 273.6 Hz), 119.4, 114.0, 55.3, 53.7, 38.4, 36.0 ; **^{19}F NMR (376 MHz, CDCl$_3$):** δ -58.3; **IR (neat, ν_{max}):** 2919, 1650, 1606, 1510, 1135 cm^{-1}; **HRMS-ESI** (*m/z*): Calcd. for $C_{14}H_{17}F_3NO_4S$ [M+H]$^+$ 352.0825, found 352.0819.

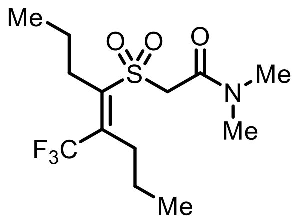

***(E)-N,N-dimethyl-2-((5-(trifluoromethyl)oct-4-en-4-yl)sulfonyl)acetamide (27)*:** Following the general method of fluoroalkyl sulfonylalkylation 2.4, the title compound **27** was obtained as a transparent oil (69 mg, 42% yield) after purification by silica gel flash chromatography (hexanes:EtOAc = 20:1 to 1:9). The reaction mixture was irradiated for 12 h. Major isomer was isolated as a mixture of separable diastereomers in a 7.5:1 ratio as determined in a crude reaction mixture by ^{1}H NMR.

^{1}H NMR (400 MHz, CDCl$_3$; δ 4.17 (s, 2H), 3.18 (s, 3H), 2.99 (s, 3H), 2.65 – 2.48 (m, 4H), 1.59 (p, J = 7.5 Hz, 4H), 0.97 (q, J = 7.7 Hz, 6H).; **^{13}C NMR (101 MHz, CDCl$_3$):** δ 160.7, 148.4 (q, $^3J_{CF}$ = 2.9 Hz), 142.1 (q, $^2J_{CF}$ = 28.2 Hz), 123.4 (q, $^1J_{CF}$ = 280.5 Hz), 59.6,38.7, 36.0, 33.23 (q, $^3J_{CF}$ = 2.1 Hz), 31.75 (q, $^2J_{CF}$ = 2.4 Hz), 23.5, 22.6, 14.2(3), 14.2(0); **^{19}F NMR (376 MHz, CDCl$_3$):** δ -60.1; **IR (neat, ν_{max}):** 2966, 1650, 1467, 1326, 1129 cm^{-1}. **HRMS-ESI** (*m/z*): Calcd. for $C_{13}H_{23}F_3NO_3S$ [M+H]$^+$ 330.1345, found 330.1337.

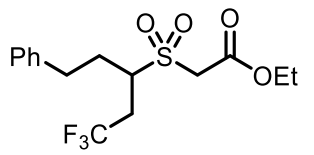

***Methyl 2-(1,1,1-trifluoro-5-phenylpentan-3-yl)sulfonyl)acetate (28)*:** Following the general method of fluoroalkyl sulfonylalkylation 2.4, the title compound **28** was obtained as a transparent oil (109 mg, 62% yield) after purification by silica gel flash chromatography (hexanes:EtOAc = 20:1 to 1:1). The reaction mixture was irradiated for 12 h.

^{1}H NMR (400 MHz, CDCl$_3$): δ 7.36 – 7.27 (m, 2H), 7.25 – 7.18 (m, 3H), 4.31 – 4.19 (m, 2H), 4.04 – 3.93 (m, 2H), 3.85 – 3.73 (m, 1H), 3.03 – 2.77 (m, 3H), 2.64 – 2.46 (m, 1H), 2.47 – 2.33 (m, 1H), 2.24 – 2.12 (m, 1H), 1.30 (t, J = 7.2 Hz, 3H); **^{13}C NMR (101 MHz, CDCl$_3$):** δ 162.4, 139.8, 128.7, 128.6, 126.6, 125.6 (q, $^1J_{CF}$ = 277.2 Hz), 63.0, 56.3 (q, $^3J_{CF}$ = 2.3 Hz), 56.0, 32.6 (d, $^2J_{CF}$ = 30.4 Hz), 32.1, 30.2, 13.8; **^{19}F NMR (376 MHz, CDCl$_3$):** δ -63.5; **IR (neat, ν_{max}):** 3095, 1745, 1325, 1139 cm^{-1}; **HRMS-ESI** (*m/z*): Calcd. for $C_{15}H_{19}F_3O_4SNa$ [M+Na]$^+$ 375.0848, found 375.0852.

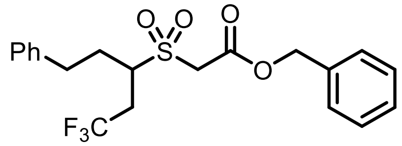

***Benzyl 2-((1,1,1-trifluoro-5-phenylpentan-3-yl)sulfonyl)acetate (29)*:** Following the general method of fluoroalkyl sulfonylalkylation 2.4, the title compound **29** was obtained as a transparent oil (114 mg, 55% yield) after purification by silica gel flash chromatography (hexanes:EtOAc = 20:1 to 1:2). The reaction mixture was irradiated for 12 h.

^{1}H NMR (400 MHz, CDCl$_3$): δ 7.44 – 7.33 (m, 5H), 7.33 – 7.27 (m, 2H), 7.25 – 7.16 (m, 3H), 5.22 (s, 2H), 4.10 – 3.93 (m, 2H), 3.78 – 3.65 (m, 1H), 2.98 – 2.70 (m, 3H), 2.59 – 2.43 (m, 1H), 2.40 – 2.26 (m, 1H), 2.21 – 2.05 (m, 1H).; **^{13}C NMR (101 MHz, CDCl$_3$):** δ 162.3, 139.8, 134.2, 128.9, 128.7, 128.7, 128.6, 128.3, 126.6, 125.5 (q, $^1J_{CF}$ = 277.2 Hz), 68.5, 56.4 (q, $^3J_{CF}$ = 2.3 Hz),

55.9, 32.5 (q, $^2J_{CF}$ = 30.0 Hz), 32.1, 30.2; **^{19}F NMR (376 MHz, CDCl$_3$):** δ -63.5; **IR (neat, ν_{max}):** 3090, 1745, 1323, 1138 cm^{-1}; **HRMS-ESI** (*m/z*): Calcd. for $C_{20}H_{21}F_3O_4SNa$ [M+Na]$^+$ 437.1005, found 437.1010.

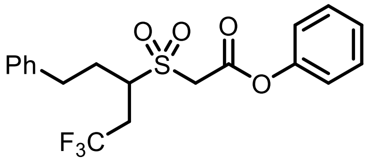

Phenyl 2-((1,1,1-trifluoro-5-phenylpentan-3-yl)sulfonyl)acetate (30)**:** Following the general method of fluoroalkyl sulfonylalkylation 2.4, the title compound **30** was obtained as a transparent oil (100 mg, 50% yield) after purification by silica gel flash chromatography (hexanes:EtOAc = 20:1 to 1:2). The reaction mixture was irradiated for 12 h.

^{1}H NMR (400 MHz, CDCl$_3$): δ 7.43 – 7.35 (m, 2H), 7.33 – 7.24 (m, 3H), 7.24 – 7.15 (m, 3H), 7.14 – 7.06 (m, 2H), 4.25 – 4.11 (m, 2H), 3.87 – 3.75 (m, 1H), 3.07 – 2.74 (m, 3H), 2.65 – 2.49 (m, 1H), 2.47 – 2.35 (m, 1H), 2.25 – 2.12 (m, 1H); **^{13}C NMR (101 MHz, CDCl$_3$):** δ 161.3, 149.8, 139.6, 129.6, 128.7, 128.3, 126.8, 126.6, 125.5 (q, $^1J_{CF}$ = 277.3 Hz), 121.0, 56.6 (q, $^3J_{CF}$ = 2.4 Hz), 56.0, 32.6 (q, $^2J_{CF}$ = 30.4 Hz), 32.1, 30.2; **^{19}F NMR (376 MHz, CDCl$_3$):** δ -63.4; **IR (neat, ν_{max}):** 3029, 1756, 1323, 1135 cm^{-1}; **HRMS-ESI** (*m/z*): Calcd. for $C_{19}H_{19}F_3O_4SNa$ [M+Na]$^+$ 423.0848, found 423.0853.

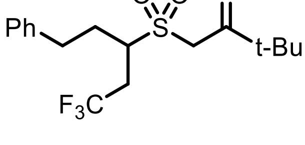

3,3-Dimethyl-1-((1,1,1-trifluoro-5-phenylpentan-3-yl)sulfonyl)butan-2-one (31)**:** Following the general method of fluoroalkyl sulfonylalkylation 2.4, the title compound **31** was obtained as a transparent oil (109 mg, 60% yield) after purification by silica gel flash chromatography (hexanes:EtOAc = 20:1 to 1:1). The reaction mixture was irradiated for 12 h.

^{1}H NMR (400 MHz, CDCl$_3$): δ 7.34 – 7.28 (m, 2H), 7.27 – 7.19 (m, 3H), 3.96 – 3.85 (m, 2H), 3.84 – 3.74 (m, 1H), 3.04 – 2.86 (m, 2H), 2.88 – 2.76 (m, 1H), 2.64 – 2.46 (m, 1H), 2.46 – 2.31 (m, 1H), 2.23 – 2.11 (m, 1H), 1.49 (s, 9H); **^{13}C NMR (101 MHz, CDCl$_3$):** δ 161.4, 139.9, 128.7, 128.4, 126.6, 125.7 (q, $^1J_{CF}$ = 277.2 Hz), 84.7, 57.1, 56.1, 56.0, 56.1 (q, $^3J_{CF}$ = 2.2 Hz), 32.6 (q, $^2J_{CF}$ = 30.4 Hz), 30.2, 27.8; **^{19}F NMR (376 MHz, CDCl$_3$):** δ -63.5; **IR (neat, ν$_{max}$):** 2984, 1748, 1326, 1136 cm^{-1}; **HRMS-ESI** (*m/z*): Calcd. for $C_{17}H_{23}F_3O_3SNa$ [M+Na]$^+$ 403.1161, found 403.1164.

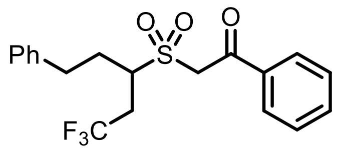

1-Phenyl-2-((1,1,1-trifluoro-5-phenylpentan-3-yl)sulfonyl)ethan-1-one (32): Following the general method of fluoroalkyl sulfonylalkylation 2.4, the title compound **32** was obtained as a transparent oil (110 mg, 57% yield) after purification by silica gel flash chromatography (hexanes:EtOAc = 20:1 to 1:2). The reaction mixture was irradiated for 12 h.

^{1}H NMR (400 MHz, CDCl$_3$): δ 8.05 – 7.92 (m, 2H), 7.72 – 7.62 (m, 1H), 7.53 (t, J = 7.8 Hz, 2H), 7.34 – 7.16 (m, 5H), 4.69 – 4.52 (m, 2H), 3.88 – 3.77 (m, 1H), 3.07 – 2.79 (m, 3H), 2.66 – 2.51 (m, 1H), 2.49 – 2.35 (m, 1H), 2.28 – 2.13 (m, 1H); **^{13}C NMR (101 MHz, CDCl$_3$):** δ 188.6, 139.9, 135.5, 134.8, 129.2, 129.0, 128.6, 128.4, 125.5(6) (q, $^1J_{CF}$ = 277.4 Hz), 126.5(0), 58.0, 56.9 (q, $^3J_{CF}$ = 2.3 Hz), 32.5 (q, $^2J_{CF}$ = 30.3 Hz), 32.1, 30.2; **^{19}F NMR (376 MHz, CDCl$_3$):** δ -63.3; **IR (neat, ν$_{max}$):** 3067, 1731, 1302, 1138 cm^{-1}; **HRMS-ESI** (*m/z*): Calcd. for $C_{19}H_{19}F_3O_3SNa$ [M+Na] $^+$ 407.0899, found 407.0903.

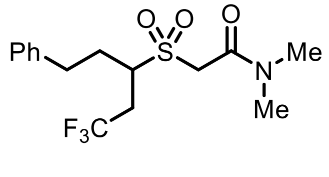

N,N-dimethyl-2-((1,1,1-trifluoro-5-phenylpentan-3-yl)sulfonyl)acetamide (33): Following the general method of fluoroalkyl sulfonylalkylation 2.4, the title compound **33** was obtained as a light yellowish solid (86 mg, 50 % yield) after purification by silica gel flash chromatography (hexanes:EtOAc = 20:1 to 1:9). The reaction mixture was irradiated for 12 h.

^{1}H NMR (400 MHz, CDCl$_3$): δ 7.35 – 7.27 (m, 2H), 7.25 – 7.16 (m, 3H), 4.17 – 4.05 (m, 2H), 3.96 – 3.84 (m, 1H), 3.17 (s, 3H), 3.02 (s, 3H), 3.01 – 2.79 (m, 3H), 2.65 – 2.49 (m, 1H), 2.44 – 2.31 (m, 1H), 2.27 – 2.13 (m, 1H); **^{13}C NMR (101 MHz, CDCl$_3$):** δ 161.1, 140.2, 128.6, 128.4, 126.4, 125.61 (q, $^{1}J_{CF}$ = 277.6 Hz). 56.7 (q, $^{3}J_{CF}$ = 2.4 Hz), 54.6, 38.6, 36.1, 32.3 (q, $^{2}J_{CF}$ = 30.3 Hz), 32.2, 30.2; **^{19}F NMR (376 MHz, CDCl$_3$):** δ -63.3; **IR (neat, ν_{max}):** 2924, 2854, 1647, 1421, 1312, 1137 cm^{-1}. **HRMS-ESI** (*m/z*): Calcd. for $C_{15}H_{21}F_3NO_3S$ [M+H]$^+$ 352.1189, found 352.1184.

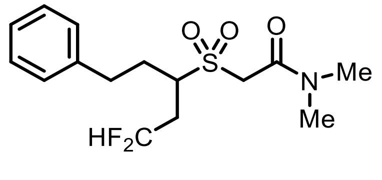

2-((1,1-Difluoro-5-phenylpentan-3-yl)sulfonyl)-N,N-dimethylacetamide (34): Following the general method of fluoroalkyl sulfonylalkylation 2.4, the title compound **34** was obtained as a transparent oil (167 mg, 70% yield) after purification by silica gel flash chromatography (hexanes:EtOAc = 20:1 to 0:100). The reaction mixture was irradiated for 12 h.

^{1}H NMR (400 MHz, CDCl$_3$): δ 7.35 – 7.26 (m, 2H), 7.26 – 7.16 (m, 3H), 6.14 (tdd, *J* = 56.4, 5.8, 3.7 Hz, 1H), 4.09 (s, 2H), 3.78 – 3.65 (m, 1H), 3.17 (s, 3H), 3.02 (s, 3H), 2.93 – 2.70 (m, 2H), 2.70 – 2.51 (m, 1H), 2.40 – 2.21 (m, 2H), 2.12 – 1.99 (m, 1H); **^{13}C NMR (101 MHz, CDCl$_3$):** δ 161.7, 140.1, 128.6, 128.4, 126.5, 115.2 (t, $^{1}J_{CF}$ = 240.1 Hz), 57.0 (t, $^{3}J_{CF}$ = 5.3 Hz), 54.4, 38.7, 36.1, 32.3, 32.2 (t, $^{2}J_{CF}$ = 23.6 Hz), 30.6; **^{19}F NMR (376 MHz, CDCl$_3$):** δ -112.90 (d, $^{1}J_{FF}$ = 286.4 Hz, 1F), -116.69 (d, $^{1}J_{FF}$ = 286.4 Hz, 1F); **IR (neat, ν_{max}):** 2936, 1642, 1497, 1262, 1089 cm^{-1}. **HRMS-ESI** (*m/z*): Calcd. for $C_{10}H_{19}F_3O_4S$ [M+Na]$^+$ 334.1282, found 334.

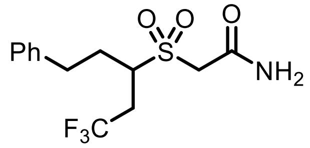

***2-((1,1,1-Trifluoro-5-phenylpentan-3-yl)sulfonyl)acetamide (35)*:** Following the general method of fluoroalkyl sulfonylalkylation 2.4, the title compound **35** was obtained as a white solid (121 mg, 75% yield) after purification by silica gel flash chromatography (hexanes:EtOAc = 20:1 to 1:9). The reaction mixture was irradiated for 12 h.

^{1}H NMR (400 MHz, CDCl$_3$): δ 7.29 – 7.06 (m, 5H), 6.45 (s, 1H), 5.82 (s, 1H), 3.91- 3.77 (m, 2H), 3.58 – 3.43 (m, 1H), 2.96 – 2.67 (m, 3H), 2.56 – 2.39 (m, 1H), 2.39 – 2.23 (m, 1H), 2.19 – 2.04 (m, 1H); **^{13}C NMR (101 MHz, CDCl$_3$):** δ 162.5, 139.6, 128.7, 128.4, 127.5 (q, $^1J_{CF}$ = 280.0 Hz), 126.7, 124.1, 56.9 (q, $^3J_{CF}$ = 2.9 Hz)., 32.41 (q, $^2J_{CF}$ = 30.6 Hz), 32.0, 30.28; **^{19}F NMR (376 MHz, CDCl$_3$):** δ -63.4; **IR (neat, ν_{max}):** 3391, 3170, 1729, 1497, 1094 cm^{-1}; **HRMS-ESI** (*m/z*): Calcd. for $C_{13}H_{16}F_3NO_3SNa$ [M+Na]$^+$ 346.0695, found 346.0699.

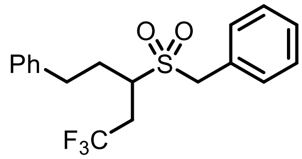

***(3-(benzylsulfonyl)-5,5,5-trifluoropentyl)benzene (36)*:** Following the general method of fluoroalkyl sulfonylalkylation 2.4, the title compound **36** was obtained as a transparent oil (93 mg, 52% yield) after purification by silica gel flash chromatography (hexanes:EtOAc = 20:1 to 1:1). The reaction mixture was irradiated for 12 h.

^{1}H NMR (400 MHz, CDCl$_3$): δ 7.45 – 7.37 (m, 3H), 7.36 – 7.19 (m, 5H), 7.19 – 7.08 (m, 2H), 4.29 – 4.17 (m, 2H), 3.24 – 3.12 (m, 1H), 2.91 – 2.69 (m, 3H), 2.52 – 2.38 (m, 1H), 2.38 – 2.26 (m, 1H), 2.16 – 2.05 (m, 1H); **^{13}C NMR (101 MHz, CDCl$_3$):** δ 139.9, 130.7, 129.3, 129.1, 128.7, 128.5, 127.0, 126.5, 125.6 (q, $^1J_{CF}$ = 277.2 Hz), 58.1, 53.6 (q, $^3J_{CF}$ = 2.2 Hz), 32.8 (q, $^2J_{CF}$ = 30.0

Hz), 32.1, 30.3; **^{19}F NMR (376 MHz, CDCl3):** δ -63.4; **IR (neat, ν_{max}):** 3068, 1302, 1138, 699 cm^{-1}; **HRMS-ESI** (*m/z*): Calcd. For $C_{18}H_{19}F_3O_2S$ $[M]^+$ 356.1052, found 356.1056.

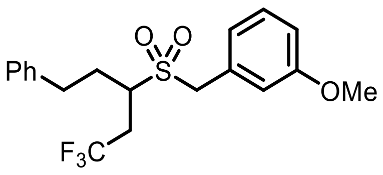

***1-methoxy-3-(((1,1,1-trifluoro-5-phenylpentan-3-yl)sulfonyl)methyl)benzene (37)*:** Following the general method of fluoroalkyl sulfonylalkylation 2.4, the title compound **37** was obtained as a white solid (137 mg, 71% yield) after purification by silica gel flash chromatography (hexanes:EtOAc = 20:1 to 1:2). The reaction mixture was irradiated for 12 h.

^{1}H NMR (400 MHz, $CDCl_3$): δ 7.24 – 7.15 (m, 3H), 7.14 – 7.09 (m, 1H), 7.05 – 6.99 (m, 2H), 6.89 – 6.77 (m, 3H), 4.16 – 4.05 (m, 2H), 3.70 (s, 3H), 3.15 – 3.03 (m, 1H), 2.79 – 2.58 (m, 3H), 2.44 – 2.28 (m, 1H), 2.28 – 2.16 (m, 1H), 2.06 – 1.95 (m, 1H); **^{13}C NMR (101 MHz, $CDCl_3$):** δ 160.0, 139.8, 130.1, 128.57, 128.36, 128.29, 126.41, 125.55 (q, $^1J_{CF}$ = 277.6 Hz), 122.78, 115.90, 115.05, 58.00, 55.21, 53.58, 53.57 (q, $^3J_{CF}$ = 2.3 Hz), 53.56, 32.78 (q, $^2J_{CF}$ = 30.0 Hz), 32.02, 30.09; **^{19}F NMR (376 MHz, $CDCl_3$):** δ -63.40; **IR (neat, ν_{max}):** 2940, 2839, 1601, 1491, 1311, 1262, 1117 cm^{-1}; **HRMS-ESI** (*m/z*): Calcd. for $C_{19}H_{22}F_3O_3S$ $[M+H]^+$ 387.1236, found 387.1234.

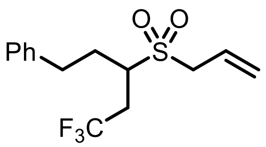

***(3-(allylsulfonyl)-5,5,5-trifluoropentyl)benzene (38)*:** Following the general method of fluoroalkyl sulfonylalkylation 2.4, the title compound **38** was obtained as a transparent oil (86 mg, 56% yield) after purification by silica gel flash chromatography (hexanes:EtOAc = 20:1 to 1:1). The reaction mixture was irradiated for 12 h.

^{1}H NMR (400 MHz, $CDCl_3$): δ 7.37 – 7.14 (m, 5H), 5.90 – 5.74 (m, 1H), 5.56 – 5.24 (m, 2H), 3.79 – 3.60 (m, 2H), 3.33 – 3.22 (m, 1H), 2.96 – 2.70 (m, 3H), 2.56 – 2.41 (m, 1H), 2.38 – 2.24

(m, 1H), 2.15 – 2.03 (m, 1H). ; **^{13}C NMR (101 MHz, $CDCl_3$):** δ 139.8, 128.7, 128.5, 128.4, 126.6, 125.7 (q, $^{1}J_{CF}$ = 277.2 Hz). 125.2, 124.2, 56.2, 53.52 (q, $^{3}J_{CF}$ = 2.1 Hz), 32.6 (q, $^{2}J_{CF}$ = 30.2 Hz), 32.0, 30.2; **^{19}F NMR (376 MHz, CDCl3):** δ -63.84; **IR (neat, ν_{max}):** 3029, 1603, 1389, 1313, 1259, 1128 cm^{-1}. **HRMS-ESI** (*m/z*): Calcd. for $C_{14}H_{18}F_3O_2S$ $[M+H]^+$ 307.0974, found 307.0969.

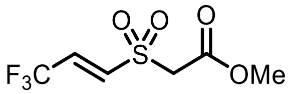

***Methyl (E)-2-((3,3,3-trifluoroprop-1-en-1-yl)sulfonyl)acetate (39)*:** Following the general method of fluoroalkyl sulfonylalkylation 2.5, the title compound **39** was obtained as a white soild (46 mg, 40% yield) after purification by silica gel flash chromatography (hexanes:EtOAc = 20:1 to 1:2). The reaction mixture was irradiated for 12 h.

^{1}H NMR (400 MHz, $CDCl_3$): δ 7.33 (dq, *J* = 15.3, 1.8 Hz, 1H), 6.87 (dq, *J* = 15.3, 6.0 Hz, 1H), 4.11 (s, 2H), 3.84 (s, 3H); **^{13}C NMR (101 MHz, $CDCl_3$):** δ 162.5, 136.72 (q, $^{3}J_{CF}$ = 5.9 Hz), 132.26 (q, $^{2}J_{CF}$ = 37.2 Hz), 120.95 (q, $^{1}J_{CF}$ = 272.1 Hz), 59.0, 53.6; **^{19}F NMR (376 MHz, $CDCl_3$):** δ -65.2; **IR (neat, ν_{max}):** 3082, 2987, 1747, 1668, 1459, 1327, 1114 cm^{-1}; **HRMS-ESI** (*m/z*): Calcd. for $C_6H_6F_3O_4S$ $[M-H]^-$ 230.9944, found 230.9942.

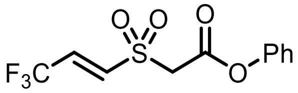

***Phenyl (E)-2-((3,3,3-trifluoroprop-1-en-1-yl)sulfonyl)acetate (40)*:** Following the general method of fluoroalkyl sulfonylalkylation 2.5, the title compound **40** was obtained as a white solid (52 mg, 35% yield) after purification by silica gel flash chromatography (hexanes:EtOAc = 20:1 to 1:2). The reaction mixture was irradiated for 12 h.

^{1}H NMR (400 MHz, $CDCl_3$): δ 7.46 – 7.28 (m, 4H), 7.17 – 7.11 (m, 2H), 6.97 – 6.87 (m, 1H), 4.34 (s, 2H); **^{13}C NMR (101 MHz, $CDCl_3$):** δ 160.9, 149.8, 136.7 (q, $^3J_{CF}$ = 5.9 Hz), 132.6 (q, $^2J_{CF}$ = 37.2 Hz), 129.8, 127.0, 120.9(4), 120.8(9) (q, $^1J_{CF}$ = 272.3 Hz); **^{19}F NMR (376 MHz, $CDCl_3$):** δ -65.1; **IR (neat, ν_{max}):** 3082, 2987, 1747, 1668, 1459, 1327, 1114 cm^{-1}; **HRMS-ESI** (*m/z*): Calcd. for $C_{11}H_8F_3O_4S$ $[M-H]^-$ 293.0101, found 293.0102.

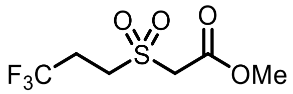

***Methyl 2-((3,3,3-trifluoropropyl)sulfonyl)acetate (41)*:** Following the general method of fluoroalkyl sulfonylalkylation 2.5, the title compound **41** was obtained as a white solid (73 mg, 62% yield) after purification by silica gel flash chromatography (hexanes:EtOAc = 20:1 to 1:1). The reaction mixture was irradiated for 12 h.

^{1}H NMR (400 MHz, $CDCl_3$): δ 4.04 (s, 2H), 3.85 (s, 3H), 3.56 – 3.48 (m, 2H), 2.77 – 2.63 (m, 2H); **^{13}C NMR (101 MHz, $CDCl_3$):** δ 163.1, 125.4 (q, $^1J_{CF}$ = 276.4 Hz), 57.7, 53.6, 46.8 (q, $^3J_{CF}$ = 3.0 Hz), 27.0 (q, $^2J_{CF}$ = 31.9 Hz); **^{19}F NMR (376 MHz, $CDCl_3$):** δ -66.0; **IR (neat, ν_{max}):** 2979, 1731, 1502, 1353, 1195 cm^{-1}; **HRMS-ESI** (*m/z*): Calcd. for $C_6H_9F_3O_4SNa$ $[M+Na]^+$ 257.0066, found 257.0065.

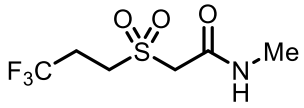

***N-methyl-2-((3,3,3-trifluoropropyl)sulfonyl)acetamide (42)*:** Following the general method of fluoroalkyl sulfonylalkylation 2.5, the title compound **42** was obtained as a white solid (87 mg, 75% yield) after purification by silica gel flash chromatography (hexanes:EtOAc = 20:1 to 1:9). The reaction mixture was irradiated for 12 h.

^{1}H NMR (400 MHz, $(CD_3)_2CO$): δ 7.62 (s, 1H), 4.11 (s, 2H), 3.68 – 3.58 (m, 2H), 2.91 – 2.70 (m, 5H); **^{13}C NMR (101 MHz, $(CD_3)_2CO$):** δ 163.0, 127.2 (q, $^1J_{CF}$ = 275.5 Hz), 47.04 (q, $^3J_{CF}$ = 3.1 Hz), 59.1, 27.69 (q, $^2J_{CF}$ = 31.0 Hz), 26.5; **^{19}F NMR (376 MHz, $(CD_3)_2CO$):** δ -66.6; **IR (neat, ν_{max}):** 3297, 2997, 1654, 1558, 1276, 1130 cm^{-1}; **HRMS-ESI** (*m/z*): Calcd. For $C_6H_{11}F_3NO_3S$ $[M+H]^+$ 234.0406, found 234.0408.

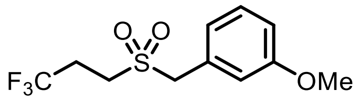

1-Methoxy-3-(((3,3,3-trifluoropropyl)sulfonyl)methyl)benzene (43)**:** Following the general method of fluoroalkyl sulfonylalkylation 2.5, the title compound **43** was obtained as a transparent oil (64 mg, 45% yield) after purification by silica gel flash chromatography (hexanes:EtOAc = 20:1 to 1:1). The reaction mixture was irradiated for 12 h.

^{1}H NMR (400 MHz, $CDCl_3$): δ 7.40 – 7.28 (m, 1H), 7.01– 6.91 (m, 3H), 4.26 (s, 2H), 3.82 (s, 3H), 3.11 – 3.00 (m, 2H), 2.62 – 2.46 (m, 2H); **^{13}C NMR (101 MHz, $CDCl_3$):** δ 160.1, 130.3, 128.5, 125.4 (q, $^1J_{CF}$ = 279.6 Hz), 122.6, 116.0, 115.0, 60.2, 55.3, 44.2 (q, $^3J_{CF}$ = 2.9 Hz), 27.0 (q, $^2J_{CF}$ = 31.6 Hz); **^{19}F NMR (376 MHz, $CDCl_3$):** δ -66.0 ; **IR (neat, ν_{max}):** 3016, 1603, 1298, 1083 cm^{-1}; **HRMS-ESI** (*m/z*): Calcd. for $C_{11}H_{14}F_3O_3S$ $[M+H]^+$ 283.0610, found 283.0613.

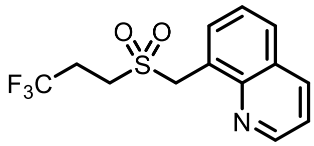

8-(((3,3,3-Trifluoropropyl)sulfonyl)methyl)quinoline (44)**:** Following the general method of fluoroalkyl sulfonylalkylation 2.5, the title compound **44** was obtained as a white solid (70 mg, 46% yield) after purification by silica gel flash chromatography (hexanes:EtOAc = 20:1 to 1:2). The reaction mixture was irradiated for 12 h.

^{1}H NMR (400 MHz, CDCl$_3$): δ 8.93 (dd, J = 4.2, 1.8 Hz, 1H), 8.23 (dd, J = 8.3, 1.8 Hz, 1H), 7.93 (ddd, J = 13.7, 7.7, 1.4 Hz, 2H), 7.68 – 7.59 (m, 1H), 7.50 (dd, J = 8.3, 4.2 Hz, 1H), 5.08 (s, 2H), 3.06 – 2.90 (m, 2H), 2.83 – 2.65 (m, 2H); **^{13}C NMR (101 MHz, CDCl$_3$):** δ 150.4, 145.9, 136.8, 133.3, 129.5, 128.4, 126.7, 126.6, 125.5 (q, $^1J_{CF}$ = 277.1 Hz), 121.9, 54.3, 45.3 (q, $^3J_{CF}$ = 3.0 Hz), 27.9 (q, $^2J_{CF}$ = 31.2 Hz) ; **^{19}F NMR (376 MHz, CDCl3):** δ -66.1; **IR (neat, ν$_{max}$):** cm^{-1}. **HRMS-ESI** (*m/z*): Calcd. for $C_{13}H_{12}F_3NO_2S$ [M+H]$^+$ 304.0614, found 304.0617.

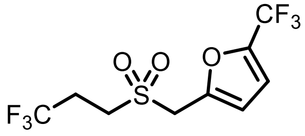

***2-(Trifluoromethyl)-5-(((3,3,3-trifluoropropyl)sulfonyl)methyl)furan (45)*:** Following the general method of fluoroalkyl sulfonylalkylation 2.5, the title compound **45** was obtained as a white solid (65 mg, 42% yield) after purification by silica gel flash chromatography (hexanes:EtOAc = 20:1 to 1:2). The reaction mixture was irradiated for 12 h.

^{1}H NMR (400 MHz, CDCl$_3$): δ 6.91 – 6.82 (m, 1H), 6.71 – 6.59 (m, 1H), 4.41 (s, 2H), 3.27 – 3.14 (m, 2H), 2.70 – 2.54 (m, 2H); **^{13}C NMR (101 MHz, CDCl$_3$):** δ 144.8 (d, $^3J_{CF}$ = 1.6 Hz), 143.5 (q, $^2J_{CF}$ = 43.5 Hz), 125.4 (q, $^1J_{CF}$ = 276.5 Hz), 118.6 (q, $^1J_{CF}$ = 267.4 Hz), 113.7, 113.5 (q, $^3J_{CF}$ = 2.8 Hz), 52.9, 45.9 (q, $^3J_{CF}$ = 3.0 Hz), 27.3 (q, $^2J_{CF}$ = 31.9 Hz); **^{19}F NMR (376 MHz, CDCl3):** δ -64.4, -66.2 ; **IR (neat, ν$_{max}$):** 2995, 1558, 1294, 1121 cm^{-1}; **HRMS-ESI** (*m/z*): Calcd. for $C_9H_7F_6O_3S$ [M-H]$^-$ 309.0026, found 309.0026.

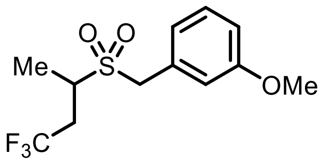

***(((4,4,4-Trifluorobutan-2-yl)sulfonyl)methyl)benzene (46)*:** Following the general method of fluoroalkyl sulfonylalkylation 2.5, the title compound **46** was obtained as a white solid (77 mg,

52% yield) after purification by silica gel flash chromatography (hexanes:EtOAc = 20:1 to 1:2). The reaction mixture was irradiated for 12 h.

^{1}H NMR (400 MHz, CDCl$_3$): δ 7.38 – 7.29 (m, 1H), 7.00 – 6.90 (m, 3H), 4.29 – 4.21 (m, 2H), 3.82 (s, 3H), 3.27 – 3.11 (m, 1H), 2.91 – 2.74 (m, 1H), 2.35 – 2.16 (m, 1H), 1.51 (dd, J = 6.8, 1.0 Hz, 3H); **^{13}C NMR (101 MHz, CDCl$_3$):** δ 160.0, 130.2, 128.3, 125.7 (d, $^1J_{CF}$ = 277.9 Hz), 122.7, 116.0, 115.0, 56.7, 55.3, 50.5 (d, $^3J_{CF}$ = 2.5 Hz), 33.2 (q, $^2J_{CF}$ = 30.0 Hz), 13.6, 13.5(9), 13.5(7) ; **^{19}F NMR (376 MHz, CDCl3):** δ -63.2; **IR (neat, ν$_{max}$):** cm^{-1}. **HRMS-ESI** (*m/z*): Calcd. for $C_{12}H_{16}F_3O_3S$ [M+H]$^+$ 297.0768, found 297.0767.

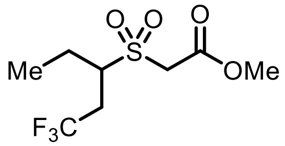

***Ethyl 2-((1,1,1-trifluoropentan-3-yl)sulfonyl)acetate (47)*:** Following the general method of fluoroalkyl sulfonylalkylation 2.5, the title compound **47** was obtained as a transparent oil (62 mg, 47% yield) after purification by silica gel flash chromatography (hexanes:EtOAc = 20:1 to 1:2). The reaction mixture was irradiated for 12 h.

^{1}H NMR (400 MHz, CDCl$_3$): δ 4.03 (s, 2H), 3.83 (s, 3H), 3.74 – 3.64 (m, 1H), 2.99 – 2.81 (m, 1H), 2.58 – 2.40 (m, 1H), 2.18 – 1.89 (m, 2H), 1.15 (t, *J* = 7.5 Hz, 3H); **^{13}C NMR (101 MHz, CDCl$_3$):** δ 163.1, 125.6 (q, $^1J_{CF}$ = 277.0 Hz), 57.8 (q, $^3J_{CF}$ = 2.3 Hz), 55.9, 53.5, 31.6 (q, $^2J_{CF}$ = 30.5 Hz), 21.5, 10.2; **^{19}F NMR (376 MHz, CDCl3):** δ -64.0; **IR (neat, ν$_{max}$):** 2959, 1741, 1315, 1130 cm^{-1}. **HRMS-ESI** (*m/z*): Calcd. For $C_8H_{12}F_3O_4S$ [M-H]$^-$ 261.0414, found 261.0413.

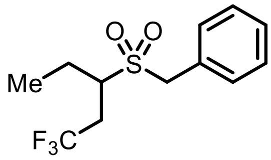

***(((1,1,1-Trifluoropentan-3-yl)sulfonyl)methyl)benzene (48)*:** Following the general method of fluoroalkyl sulfonylalkylation 2.5, the title compound **48** was obtained as a white solid (102 mg, 73% yield) after purification by silica gel flash chromatography (hexanes:EtOAc = 20:1 to 1:1). The reaction mixture was irradiated for 12 h.

^{1}H NMR (400 MHz, $CDCl_3$): δ 7.47 – 7.37 (m, 5H), 4.27 (s, 2H), 3.11 – 3.02 (m, 1H), 2.88 – 2.68 (m, 1H), 2.50 – 2.32 (m, 1H), 2.09 – 1.84 (m, 2H), 1.09 (t, J = 7.5 Hz, 3H); **^{13}C NMR (101 MHz, $CDCl_3$):** δ 130.6, 129.2, 129.1, 127.0, 125.6 (q, $^{1}J_{CF}$ = 277.7 Hz), 55.6 (q, $^{3}J_{CF}$ = 2.3 Hz), 58.0, 31.7 (q, $^{2}J_{CF}$ = 30.2 Hz), 21.5, 10.2; **^{19}F NMR (376 MHz, CDCl3):** δ -63.8; **IR (neat, ν_{max}):** 2977, 1300, 1111, 718 cm^{-1}; **HRMS-ESI** (*m/z*): Calcd. for $C_{12}H_{16}F_3O_2S$ $[M+H]^+$ 281.0818, found 281.0822.

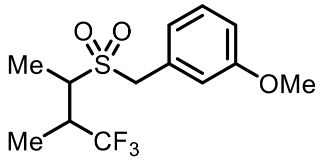

***1-Methoxy-3-(((4,4,4-trifluoro-3-methylbutan-2-yl)sulfonyl)methyl)benzene (49)*:** Following the general method of fluoroalkyl sulfonylalkylation 2.5, the title compound **49** was obtained as a transparent oil (74 mg, 48% yield) after purification by silica gel flash chromatography (hexanes:EtOAc = 20:1 to 1:2). The reaction mixture was irradiated for 12 h.

^{1}H NMR (400 MHz, $CDCl_3$): δ 7.35 – 7.27 (m, 1H), 7.01 – 6.90 (m, 3H), 4.27 – 4.17 (m, 2H), 3.81 (s, 3H), 3.31 (qd, J = 7.1, 1.6 Hz, 1H), 3.20 – 3.05 (m, 1H), 1.41 (d, J = 7.1 Hz, 3H), 1.27 (d, J = 7.2 Hz, 3H); **^{13}C NMR (101 MHz, $CDCl_3$):** δ 160.0, 130.1, 128.5, 127.0 (d, $^{1}J_{CF}$ = 277.9 Hz), 122.6, 116.0, 114.9, 52.6 (q, J_{CF} = 2.2 Hz), 35.5 (q, $^{2}J_{CF}$ = 27.3 Hz), 9.14 (q, J_{CF} = 1.5 Hz), 8.0 (q, $^{3}J_{CF}$ = 2.7 Hz); **^{19}F NMR (376 MHz, CDCl3):** δ -71.0 ; **IR (neat, ν_{max}):** cm^{-1}; **HRMS-ESI** (*m/z*): Calcd. for $C_{13}H_{18}F_3O_3S$ $[M+H]^+$ 311.0923, found 311.0923.

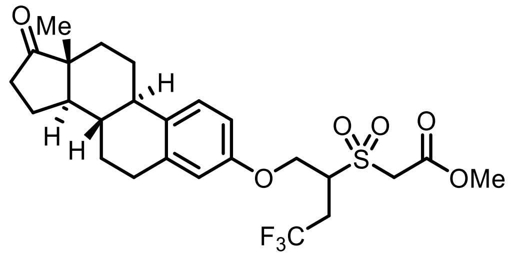

***Methyl 2-((4,4,4-trifluoro-1-(((8S,9R,13R,14R)-13-methyl-17-oxo-7,8,9,11,12,13,14,15,16,17-decahydro-6H-cyclopenta[a]phenanthren-3-yl)oxy)butan-2-yl)sulfonyl)acetate (50)*:** Following the general method of fluoroalkyl sulfonylalkylation 2.4, the title compound **50** was obtained as a white solid (41 mg, 41% yield) after purification by silica gel flash chromatography (hexanes:EtOAc = 20:1 to 1:3). The reaction mixture was irradiated for 12 h. The reaction mixture was irradiated for 12 h. Compound was isolated as a mixture of inseparable diastereomers in a 1:1 ratio as determined in a crude reaction mixture by ^{19}F NMR.

^{1}H NMR (400 MHz, $CDCl_3$): δ 7.90 – 7.80 (m, 1H), 7.40 – 7.14 (m, 2H), 5.19 – 4.95 (m, 3H), 4.83 – 4.69 (m, 2H), 4.44 (s, 3H), 3.79 – 3.61 (m, 1H), 3.58 – 3.43 (m, 2H), 3.26 – 3.08 (m, 2H), 3.07 – 2.96 (m, 1H), 2.96 – 2.45 (m, 6H), 2.38 – 1.97 (m, 7H), 1.52 (s, 3H); **^{13}C NMR (101 MHz, $CDCl_3$):** δ 163.1, 155.0, 138.3, 134.0, 126.7, 125.5 (q, $^{1}J_{CF}$ = 277.2 Hz), 114.7, 114.6, 112.2, 112.1, 65.3, 58.6, 57.3 (q, $^{3}J_{CF}$ = 2.5 Hz), 53.3, 50.3, 47.9, 43.9, 38.2, 35.8, 31.5, 29.6, 28.5 (q, $^{2}J_{CF}$ = 31.4 Hz), 26.4, 25.9, 21.5, 13.8; **^{19}F NMR (376 MHz, $CDCl_3$):** δ -63.7(0), -63.7(1); **IR (neat, ν_{max}):** 2931, 1735, 1325, 1123 cm^{-1}; **HRMS-ESI** (*m/z*): Calcd. for $C_{25}H_{30}F_3O_6S$ [M-H]$^-$ 515.1721, found 515.1723.

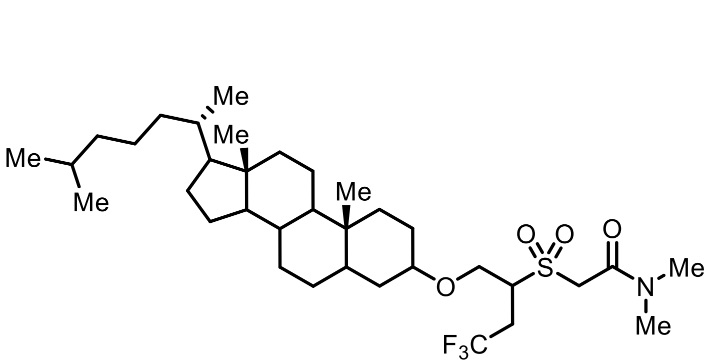

***2-((1-(((10R,13S)-10,13-dimethyl-17-((S)-6-methylheptan-2-yl)hexadecahydro-1H-cyclopenta[a]phenanthren-3-yl)oxy)-4,4,4-trifluorobutan-2-yl)sulfonyl)-N,N-dimethylacetamide (51)*:** Following the general method of fluoroalkyl sulfonylalkylation 2.4, the title compound **51** was obtained as a white solid (72 mg, 56% yield) after purification by silica gel flash chromatography (hexanes:EtOAc = 20:1 to 1:4). The reaction mixture was irradiated for 12 h. Compound was isolated as a mixture of inseparable diastereomers in a 1:1 ratio as determined in a crude reaction mixture by ^{19}F NMR.

^{1}H NMR (400 MHz, CDCl$_3$): δ 4.47 (dd, J = 14.6, 3.9 Hz, 1H), 4.23 (d, J = 14.6 Hz, 1H), 4.08 – 3.97 (m, 2H), 3.91 – 3.79 (m, 1H), 3.40 – 3.27 (m, 1H), 3.12 (s, 3H), 3.01 (s, 3H), 2.98 – 2.82 (m, 1H), 2.56 – 2.39 (m, 1H), 2.01 – 1.91 (m, 1H), 1.89 – 1.58 (m, 6H), 1.57 – 1.42 (m, 4H), 1.41 – 0.76 (m, 33H), 0.64 (s, 3H); **^{13}C NMR (101 MHz, CDCl$_3$):** δ 162.01, 125.87 (q, $^{1}J_{CF}$ = 277.3 Hz)., 79.88, 79.85, 65.07, 58.18, 57.78, 56.58, 56.41, 54.42, 44.76, 42.73, 40.13, 39.65, 38.48, 36.86, 36.30, 36.09, 35.93, 35.85, 35.61, 34.58, 34.51, 32.18, 28.98, 28.92, 28.68, 28.38, 28.15, 28.04, 27.96, 24.35, 23.97, 22.96, 22.70, 21.37, 18.81, 12.44, 12.20. *The ^{13}C NMR is complex due to presence of diastereomers containing CF_3 groups; therefore, it is difficult to assign the corresponding peaks of ^{13}C-attached to the fluorine. Hence, the complete list of the representative peaks of ^{13}C is shown;* **^{19}F NMR (376 MHz, CDCl$_3$):** δ -63.5(4), -64.5(6); **IR (neat, ν_{max}):** 2930, 2865, 1649, 1305, 1254, 1110 cm^{-1}. **HRMS-ESI** (*m/z*): Calcd. for $C_{35}H_{61}F_3NO_4S$ [M+H]$^+$ 648.4268, found 648.4252.

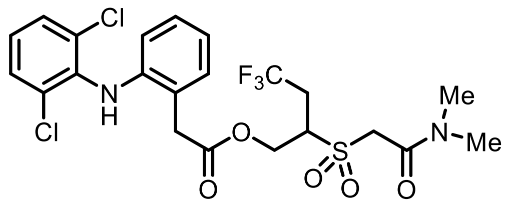

***2-((2-(Dimethylamino)-2-oxoethyl)sulfonyl)-4,4,4-trifluorobutyl 2-(2-((2,6-dichlorophenyl)amino)phenyl)acetate (52)*:** Following the general method of fluoroalkyl

sulfonylalkylation 2.4, the title compound **52** was obtained as a transparent oil (153 mg, 55% yield) after purification by silica gel flash chromatography (hexanes:EtOAc = 20:1 to 1:4). The reaction mixture was irradiated for 12 h.

^{1}H NMR (400 MHz, $CDCl_3$): δ 7.34 (d, J = 8.0 Hz, 2H), 7.23 – 7.17 (m, 1H), 7.16 – 7.09 (m, 1H), 7.02 – 6.91 (m, 2H), 6.56 – 6.51 (m, 2H), 4.98 (dd, J = 12.8, 3.0 Hz, 1H), 4.44 (dd, J = 12.9, 6.6 Hz, 1H), 4.34 – 4.25 (m, 1H), 4.18 (d, J = 15.0 Hz, 1H), 4.09 (d, J = 15.0 Hz, 1H), 3.93 – 3.82 (m, 2H), 3.08 (s, 3H), 2.97 (s, 3H), 2.65 – 2.48 (m, 1H); **^{13}C NMR (101 MHz, $CDCl_3$):** δ ; **^{19}F NMR (376 MHz, CDCl3):** δ 171.2, 161.6, 142.8, 137.6, 131.1, 129.8, 128.8, 128.2, 125.4 (d, $^1J_{CF}$ = 279.0 Hz), 124.3, 123.4, 121.9, 118.1, 60.6, 55.4, 55.90 (q, $^3J_{CF}$ = 2.4 Hz), 38.4, 38.2, 38.1, 36.0, 28.49 (q, $^2J_{CF}$ = 31.4 Hz); **^{19}F NMR (376 MHz, $CDCl_3$):** δ -63.5; **IR (neat, ν_{max}):** 3371, 1736, 1452, 1253, 1139 cm^{-1}. **HRMS-ESI** (*m/z*): Calcd. for $C_{22}H_{24}C_{12}F_3N_2O_5S$ $[M+H]^+$ 555.0730, found 555.0722.

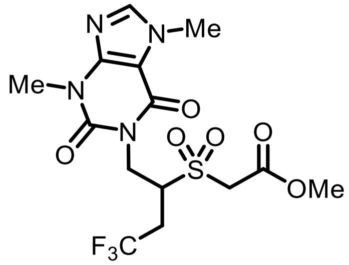

***2-((1-(3,7-Dimethyl-2,6-dioxo-2,3,6,7-tetrahydro-1H-purin-1-yl)-4,4,4-trifluorobutan-2-yl)sulfonyl)-N,N-dimethylacetamide (53)*:** Following the general method of fluoroalkyl sulfonylalkylation 2.4, the title compound **53** was obtained as a white solid (99 mg, 45% yield) after purification by silica gel flash chromatography (hexanes:EtOAc = 20:1 to 0:100). The reaction mixture was irradiated for 12 h.

^{1}H NMR (400 MHz, $CDCl_3$): δ 7.56 (s, 1H), 4.59 – 4.48 (m, 2H), 4.38 (s, 2H), 4.21 (qd, J = 6.0, 3.0 Hz, 1H), 3.99 (s, 3H), 3.84 (s, 3H), 3.59 (s, 3H), 3.13 – 2.99 (m, 1H), 2.79 – 2.70 (m, 1H). ; **^{13}C NMR (101 MHz, $CDCl_3$):** δ 162.93, 155.10, 151.56, 149.24, 142.17, 125.52 (q, $^1J_{CF}$ = 277.2 Hz), 107.36, 56.30, 55.72 (q, $^3J_{CF}$ = 2.2 Hz), 53.5, 40.5, 33.8, 31.22 (q, $^2J_{CF}$ = 31.1 Hz), 30.0; **^{19}F**

NMR (376 MHz, CDCl3): δ -64.9; **IR (neat, ν_{max}):** 2956, 2923, 2852, 1739, 1707, 1660, 1318, 1145 cm^{-1}. **HRMS-ESI** (*m/z*): Calcd. for $C_{14}H_{18}F_3N_4O_6S$ $[M+H]^+$ 427.0894, found 427.0891.

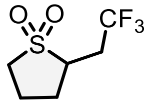

***2-(2,2,2-Trifluoroethyl)tetrahydrothiophene 1,1-dioxide (54)*:** Following the general method of fluoroalkyl sulfonylalkylation 2.4, the title compound **54** was obtained as a transparent oil (71 mg, 70% yield) after purification by silica gel flash chromatography (hexanes:EtOAc = 20:1 to 1:1). The reaction mixture was irradiated for 12 h.

^{1}H NMR (400 MHz, CDCl3): δ 3.27 – 3.11 (m, 2H), 3.06 – 2.93 (m, 1H), 2.88 – 2.71 (m, 1H), 2.59 – 2.47 (m, 1H), 2.37 – 2.21 (m, 2H), 2.20 – 2.04 (m, 1H), 1.96 – 1.81 (m, 1H); **^{13}C NMR (101 MHz, CDCl3):** δ 125.7 (q, $^1J_{CF}$ = 277.0 Hz), 55.1 (q, $^3J_{CF}$ = 2.5 Hz), 50.6, 32.4 (q, $^2J_{CF}$ = 30.4 Hz), 29.2, 20.2; **^{19}F NMR (376 MHz, CDCl3):** δ -64.1; **IR (neat, ν_{max}):** 3009, 1452, 1134 cm^{-1}. **HRMS-ESI** (*m/z*): Calcd. for $C_6H_9F_3O_2S$ $[M]^+$ 202.0270, found 202.0275.

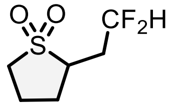

***2-(2,2-Difluoroethyl)tetrahydrothiophene 1,1-dioxide (55)*:** Following the general method of fluoroalkyl sulfonylalkylation 2.4, the title compound **55** was obtained as a transparent oil (65 mg, 71% yield) after purification by silica gel flash chromatography (hexanes:EtOAc = 20:1 to 1:1). The reaction mixture was irradiated for 12 h.

^{1}H NMR (400 MHz, CDCl3): δ 4.92 – 4.77 (m, 1.00H), 4.56 (dt, *J* = 9.0, 7.2 Hz, 0.23H), 4.42 (ddd, *J* = 10.0, 8.7, 6.9 Hz, 0.79H), 3.52 – 3.43 (m, 0.23H), 3.28 – 3.14 (m, 0.77H), 2.85 – 2.71 (m, 1H), 2.58 – 2.37 (m, 1.83H), 2.37 – 2.24 (m, 0.84H), 2.22 – 2.04 (m, 0.50H); **^{13}C NMR (101**

MHz, $CDCl_3$): δ 114.91 (t, ${}^{1}J_{CF}$ = 240.5 Hz), 55.3 (t, ${}^{3}J_{CF}$ = 4.9 Hz), 50.8, 32.7 (t, ${}^{2}J_{CF}$ = 22.5 Hz), 29.67, 20.57; **^{19}F NMR (376 MHz, $CDCl_3$):** δ -114.33 (d, ${}^{1}J_{FF}$ = 285.1 Hz), -117.62 (d, ${}^{1}J_{FF}$ = 285.1 Hz), -123.05 (d, ${}^{1}J_{FF}$ = 282.3 Hz, 1F), -125.38 (d, ${}^{1}J_{FF}$ = 282.4 Hz, 1F); **IR (neat, ν_{max}):** 2957, 1300, 1107, 1030 cm^{-1}; **HRMS-ESI** (*m/z*): Calcd. for $C_6H_{10}F_2O_2S$ $[M]^+$ 184.0364, found 184.0366.

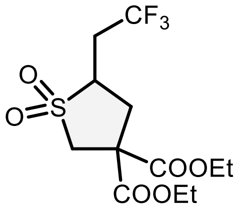

***Diethyl 5-(2,2,2-trifluoroethyl)dihydrothiophene-3,3(2H)-dicarboxylate 1,1-dioxide (56)*:** Following the general method of fluoroalkyl sulfonylalkylation 2.4, the title compound **56** was obtained as a transparent oil (100 mg, 58% yield) after purification by silica gel flash chromatography (hexanes:EtOAc = 20:1 to 1:2). The reaction mixture was irradiated for 12 h.

^{1}H NMR (400 MHz, $CDCl_3$): δ4.38 – 4.13 (m, 4H), 3.83 – 3.63 (m, 2H), 3.49 – 3.37 (m, 1H), 3.08 (dd, J = 14.1, 7.1 Hz, 1H), 2.84 – 2.69 (m, 1H), 2.44 – 2.21 (m, 2H), 1.33 – 1.20 (m, 6H); **^{13}C NMR (101 MHz, $CDCl_3$):** δ 168.1, 167.6, 125.4 (q, ${}^{1}J_{CF}$ = 277.1 Hz), 63.3, 63.2, 54.9, 54.4 (q, ${}^{3}J_{CF}$ = 2.6 Hz), 54.1, 31.8 (q, ${}^{2}J_{CF}$ = 30.9 Hz), 34.9, 13.8, 13.8; **^{19}F NMR (376 MHz, $CDCl_3$):** δ -64.1; **IR (neat, ν_{max}):** 3020, 1743, 1312, 1154 cm^{-1}. **HRMS-ESI** (*m/z*): Calcd. for $C_{12}H_{17}F_3O_6SNa$ $[M+Na]^+$ 369.0590, found 369.0594.

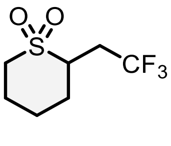

***2-(2,2,2-Trifluoroethyl)tetrahydro-2H-thiopyran 1,1-dioxide (57)*:** Following the general method of fluoroalkyl sulfonylalkylation 2.4, the title compound **57** was obtained as a transparent oil (58

mg, 54% yield) after purification by silica gel flash chromatography (hexanes:EtOAc = 20:1 to 1:1). The reaction mixture was irradiated for 12 h.

^{1}H NMR (400 MHz, $CDCl_3$): δ 3.20 – 3.08 (m, 2H), 3.03 – 2.84 (m, 2H), 2.39 – 2.21 (m, 2H), 2.18 – 2.02 (m, 2H), 1.96 – 1.83 (m, 2H), 1.58 – 1.44 (m, 1H); **^{13}C NMR (101 MHz, $CDCl_3$):** δ 126.2 (q, $^1J_{CF}$ = 279.6 Hz), 58.8, 47.5 (q, $^2J_{CF}$ = 28.2 Hz), 45.6, 20.0, 17.9, 17.6 (q, $^3J_{CF}$ = 3.1 Hz); **^{19}F NMR (376 MHz, $CDCl_3$):** δ -62.9; **IR (neat, ν_{max}):** 2955, 1504, 1252, 1135 cm^{-1}. **HRMS-ESI** (*m/z*): Calcd. for $C_7H_{11}F_3O_2S$ $[M]^+$ 216.0426, found 216.0432.

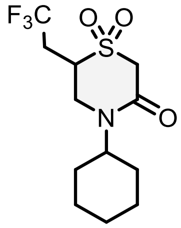

***4-Cyclohexyl-6-(2,2,2-trifluoroethyl)thiomorpholin-3-one 1,1-dioxide (58)*:** Following the general method of fluoroalkyl sulfonylalkylation 2.4, the title compound **XX** was obtained as a white solid (91 mg, 58% yield) after purification by silica gel flash chromatography (hexanes:EtOAc = 20:1 to 1:4). The reaction mixture was irradiated for 12 h.

^{1}H NMR (400 MHz, $CDCl_3$): δ 4.46 (tt, *J* = 11.6, 3.9 Hz, 1H), 4.08 (d, *J* = 1.9 Hz, 2H), 3.84 (dd, *J* = 14.7, 3.8 Hz, 1H), 3.53 (dd, *J* = 14.8, 9.7 Hz, 1H), 3.44 – 3.34 (m, 1H), 3.03 – 2.87 (m, 1H), 2.44 – 2.26 (m, 1H), 1.93 – 1.61 (m, 6H), 1.47 – 1.28 (m, 4H), 1.18 – 1.02 (m, 1H); **^{13}C NMR (101 MHz, $CDCl_3$):** δ 160.2, 125.4 (q, $^1J_{CF}$ = 277.6 Hz), 57.2, 55.16 (q, $^3J_{CF}$ = 2.3 Hz),53.8, 42.1, 30.0, 29.7, 29.1 (q, $^2J_{CF}$ = 31.1 Hz), 25.4(3), 25.3(9), 25.2; **^{19}F NMR (376 MHz, $CDCl_3$):** δ -63.2; **IR (neat, ν_{max}):** 3036, 1750, 1262, 1175 cm^{-1}; **HRMS-ESI** (*m/z*): Calcd. for $C_{12}H_{19}F_3NO_3S$ $[M+H]^+$ 314.1032, found 314.1025.

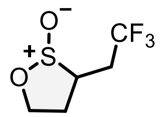

***3-(2,2,2-Trifluoroethyl)-1,2-oxathiolane 2-oxide (59)*[10]:** Following the general method of fluoroalkyl sulfonylalkylation 2.4, the title compound **59** was obtained as a transparent oil (42 mg, 45% yield) after purification by silica gel flash chromatography (hexanes:EtOAc = 20:1 to 1:1). The reaction mixture was irradiated for 12 h. Compound was isolated as a mixture of diastereomers in a 5:1 ratio as determined in a crude reaction mixture by ^{1}H NMR.

^{1}H NMR (400 MHz, CDCl$_3$): δ 6.00 (tdt, *J* = 55.7, 13.4, 3.7 Hz, 1H), 4.92 – 4.73 (m, 1H), 4.53 (dt, J = 9.1, 7.3 Hz, 0.17H), 4.41 – 4.31 (m, 0.81H), 3.48 – 3.36 (m, 0.17H), 3.28 – 3.12 (m, 0.81H), 2.85 – 2.73 (m, 0.17H), 2.59 – 2.11 (m, 3.60H), 2.10 – 1.83 (m, 0.37H); **^{13}C NMR (101 MHz, CDCl$_3$):** δ 125.9 (q, $^1J_{CF}$ = 276.9 Hz), 75.4, 74.8, 66.3, 61.5 (q, $^3J_{CF}$ = 1.9 Hz), 32.8 (d, $^2J_{CF}$ = 30.0 Hz), 31.9 (q, $^2J_{CF}$ = 30.3 Hz), 28.6, 27.6; **^{19}F NMR (376 MHz, CDCl$_3$):** δ -64.5, -64.9; **IR (neat, ν_{max}):** 1636, 1272, 1142, 1106 cm^{-1}. **HRMS-ESI** (*m/z*): Calcd. for $C_5H_8F_3O_2S$ [M+H]$^+$ 189.0192, found 189.0190.

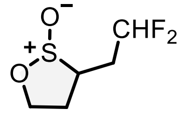

***3-(2,2-Difluoroethyl)-1,2-oxathiolane 2-oxide (60)*[10]:** Following the general method of fluoroalkyl sulfonylalkylation 2.4, the title compound **60** was obtained as a transparent oil (34 mg, 40% yield) after purification by silica gel flash chromatography (hexanes:EtOAc = 20:1 to 1:1). The reaction mixture was irradiated for 12 h. Compound was isolated as a mixture of inseparable diastereomers in a 5:1 ratio as determined in a crude reaction mixture by ^{1}H NMR.

^{1}H NMR (400 MHz, CDCl$_3$): δ 6.05 (tdd, *J* = 55.9, 5.0, 3.2 Hz, 1H), 3.25 – 3.08 (m, 2H), 3.06 – 2.92 (m, 1H), 2.60 – 2.38 (m, 2H), 2.31 – 2.18 (m, 1H), 2.20 – 1.99 (m, 2H), 1.90 – 1.79 (m, 1H)

; **^{13}C NMR (101 MHz, CDCl$_3$):** δ 115.0 (t, $^{1}J_{CF}$ = 240.4 Hz), 114.9 (t, $^{1}J_{CF}$ = 241.0 Hz), 75.0, 74.7, 66.2 (t, $^{3}J_{CF}$ = 3.6 Hz), 61.2 (t, $^{3}J_{CF}$ = 3.1 Hz), 32.70 (t, $^{2}J_{CF}$ = 22.1 Hz), 31.58 (t, $^{2}J_{CF}$ = 21.8 Hz), 28.9, 27.9; **^{19}F NMR (376 MHz, CDCl$_3$):** δ -115.01 (d, $^{1}J_{FF}$ = 285.5 Hz), -115.54 (d, $^{1}J_{FF}$ = 283.5 Hz) -116.41 (d, $^{1}J_{FF}$ = 285.3 Hz, 1F), -116.64 (d, $^{1}J_{FF}$ = 283.3 Hz,1F); **IR (neat, ν_{max}):** 1661, 1141, 1074, 1008 cm^{-1}. **HRMS-ESI** (*m/z*): Calcd. for $C_5H_8F_2O_2S$ $[M]^+$ 170.0208, found 170.0213.

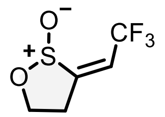

***(Z)-3-(2,2,2-Trifluoroethylidene)-1,2-oxathiolane 2-oxide (61)*:** Following the general method of fluoroalkyl sulfonylalkylation 2.4, the title compound **61** was obtained as a transparent oil (46 mg, 49% yield) after purification by silica gel flash chromatography (hexanes:EtOAc = 20:1 to 1:1). The reaction mixture was irradiated for 12 h. Compound was isolated as a mixture of inseparable diastereomers in a 1.9:1 ratio as determined in a crude reaction mixture by ^{1}H NMR.

^{1}H NMR (400 MHz, CDCl$_3$): δ 6.43 – 6.24 (m, 0.66H), 6.12 – 6.01 (m, 0.34H), 5.02 – 4.86 (m, 1H), 4.86 – 4.80 (m, 0.66H), 4.69 (td, *J* = 8.8, 3.1 Hz, 0.34H), 3.27 – 3.08 (m, 1H), 2.97 – 2.74 (m, 1H); **^{13}C NMR (101 MHz, CDCl$_3$):** δ 162.4 (q, $^{3}J_{CF}$ = 4.7 Hz), 161.03 (d, $^{3}J_{CF}$ = 3.5 Hz), 122.1 (d, $^{1}J_{CF}$ = 272.3 Hz), 121.51 (d, $^{1}J_{CF}$ = 272.1 Hz), 119.0 (q, $^{2}J_{CF}$ = 36.9 Hz), 116.7 (q, $^{2}J_{CF}$ = 37.2 Hz) ; **^{19}F NMR (376 MHz, CDCl$_3$):** δ -59.85, -61.65; **IR (neat, ν_{max}):** 1644, 1252, 1142, 1100 cm^{-1}. **HRMS-ESI** (*m/z*): Calcd. for $C_5H_5F_3O_2S$ $[M]^+$ 185.9957, found 185.9959.

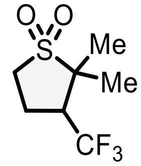

***2,2-Dimethyl-3-(trifluoromethyl)tetrahydrothiophene 1,1-dioxide (65)*:** Following the general method of fluoroalkyl sulfonylalkylation 2.4, the title compound **65** was obtained as a transparent

oil (54 mg, 50% yield) after purification by silica gel flash chromatography (hexanes:EtOAc = 20:1 to 1:1). The reaction mixture was irradiated for 12 h.

^{1}H NMR (400 MHz, CDCl$_3$): δ 3.30 – 3.08 (m, 2H), 2.97 – 2.84 (m, 1H), 2.44 – 2.30 (m, 1H), 2.22 – 2.08 (m, 1H), 1.49 (s, 3H), 1.40 (S, 3H); **^{13}C NMR (101 MHz, CDCl$_3$):** δ 126.2 (q, $^1J_{CF}$ = 279.6 Hz), 58.8, 47.5 (q, $^2J_{CF}$ = 28.2 Hz), 45.6, 19.9, 17.9, 17.6 (q, $^3J_{CF}$ = 3.1 Hz); **^{19}F NMR (376 MHz, CDCl$_3$):** δ -66.27; **IR (neat, ν_{max}):** 2956, 1305, 1270, 1054, 1020 cm^{-1}. **HRMS-ESI** (*m/z*): Calcd. for $C_7H_{12}F_3O_2S$ [M+H]$^+$ 217.0505, found 217.0505.

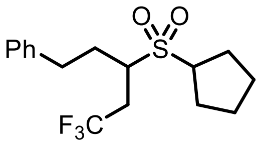

(3-(Cyclopentylsulfonyl)-5,5,5-trifluoropentyl)benzene (66)**:** Following the general method of preparation of fluoroalkyl sulfonamide 2.7.1, he title compound **66** was obtained as a transparent oil (70 mg, 42% yield) after purification by silica gel flash chromatography (hexanes:EtOAc = 20:1 to 1:3)

^{1}H NMR (400 MHz, CDCl$_3$): δ 7.27 – 7.19 (m, 2H), 7.19 – 7.09 (m, 3H), 3.44 – 3.29 (m, 1H), 3.17 – 3.06 (m, 1H), 2.93 – 2.79 (m, 2H), 2.75 – 2.65 (m, 1H), 2.51 – 2.36 (m, 1H), 2.36 – 2.24 (m, 1H), 2.10 – 1.87 (m, 4H), 1.81 – 1.66 (m, 3H), 1.64 – 1.52 (m, 2H); **^{13}C NMR (101 MHz, CDCl$_3$):** δ 140.0, 128.7, 128.5, 126.5, 125.9 (q, $^1J_{CF}$ = 277.2 Hz). 59.1, 53.7 (q, $^3J_{CF}$ = 2.5 Hz). 32.67 (q, $^2J_{CF}$ = 30.2 Hz), 32.1, 30.4, 26.9, 26.6, 25.9(6), 25.9(4); **^{19}F NMR (376 MHz, CDCl$_3$):** δ -63.7; **IR (neat, ν_{max}):** 3029, 1301, 1260, 1118 cm^{-1}; **HRMS-ESI** (*m/z*): Calcd. for $C_{16}H_{21}F_3O_2S$ [M+Na]$^+$ 357.1107, found 357.1096.

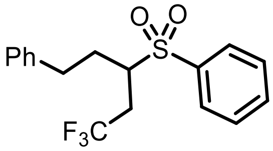

(5,5,5-Trifluoro-3-(phenylsulfonyl)pentyl)benzene (67): Following the general method of preparation of fluoroalkyl sulfonamide 2.7.5, The title compound **67** was obtained as a transparent oil (103 mg, 61% yield) after purification by silica gel flash chromatography (hexanes:EtOAc = 20:1 to 1:2).

^{1}H NMR (400 MHz, $CDCl_3$): δ δ 7.89 (dd, J = 7.5, 1.7 Hz, 2H), 7.71 (t, J = 7.5 Hz, 1H), 7.60 (t, J = 7.7 Hz, 2H), 7.29 – 7.23 (m, 2H), 7.23 – 7.16 (m, 1H), 7.10 – 7.04 (m, 2H), 3.32 – 3.24 (m, 1H), 2.91 – 2.78 (m, 2H), 2.73 – 2.65 (m, 1H), 2.51 – 2.37 (m, 1H), 2.31 – 2.21 (m, 1H), 2.10 – 2.00 (m, 1H); **^{13}C NMR (101 MHz, $CDCl_3$):** δ 139.9, 136.8, 134.3, 129.5, 128.8, 128.6, 128.3, 126.4, 125.6 (q, $^1J_{CF}$ = 277.3 Hz), 58.0 (q, $^3J_{CF}$ = 2.3 Hz), 32.7 (q, $^2J_{CF}$ = 30.1 Hz), 32.2, 30.2; **^{19}F NMR (376 MHz, CDCl3):** δ -63.5; **IR (neat, ν_{max}):** 2924, 2854, 1307, 1260, 1143, 1022 cm^{-1}; **HRMS-ESI** (*m/z*): Calcd. for $C_{17}H_{18}F_3O_2S$ $[M+H]^+$ 343.0974, found 343.0972.

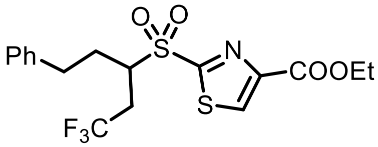

Ethyl 2-((1,1,1-trifluoro-5-phenylpentan-3-yl)sulfonyl)thiazole-4-carboxylate (68): Following the general method of preparation of fluoroalkyl sulfonamide 2.7.5, he title compound **68** was obtained as a transparent oil (97 mg, 46% yield) after purification by silica gel flash chromatography (hexanes:EtOAc = 20:1 to 1:4)

^{1}H NMR (400 MHz, $CDCl_3$): δ 8.52 (s, 1H), 7.32 – 7.12 (m, 5H), 4.46 (q, J = 7.1 Hz, 2H), 3.85 – 3.74 (m, 1H), 3.20 – 3.04 (m, 1H), 2.94 – 2.75 (m, 2H), 2.71 – 2.57 (m, 1H), 2.43 – 2.31 (m, 1H), 2.25 – 2.12 (m, 1H), 1.43 (t, J = 7.1 Hz, 3H); **^{13}C NMR (101 MHz, $CDCl_3$):** δ 164.33, 159.87, 149.60, 139.56, 133.15, 128.62, 128.33, 126.51, 125.3 (q, $^1J_{CF}$ = 277.5 Hz), 62.20, 58.2 (q, $^3J_{CF}$ = 2.4 Hz), 32.7 (q, $^2J_{CF}$ = 30.6 Hz), 32.1, 30.2, 14.2; **^{19}F NMR (376 MHz, $CDCl_3$):** δ -63.4; **IR**

(neat, νmax): 3107, 1723, 1327, 1242, 1214, 1141 cm^{-1}; **HRMS-ESI** (*m/z*): Calcd. for $C_{17}H_{19}F_3NO_4S$ $[M+H]^+$ 422.0702, found 422.0698.

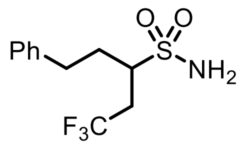

***1,1,1-Trifluoro-5-phenylpentane-3-sulfonamide (69)*:** Following the general method of preparation of fluoroalkyl sulfonamide 2.7.3, the title compound **69** was obtained as a transparent oil (91.4 mg, 65% yield) after purification by silica gel flash chromatography (hexanes:EtOAc = 20:1 to 1:4)

^{1}H NMR (400 MHz, $CDCl_3$): δ 7.35 – 7.25 (m, 2H), 7.27 – 7.15 (m, 3H), 4.70 (s, 2H), 3.34 – 3.24 (m, 1H), 2.99 – 2.77 (m, 3H), 2.59 – 2.28 (m, 2H), 2.23 – 2.09 (m, 1H); **^{13}C NMR (101 MHz, $CDCl_3$):** δ 140.0, 128.7, 128.4, 126.5, 125.7 (q, $^1J_{CF}$ = 277.0 Hz), 57.8 (d, $^3J_{CF}$ = 2.5 Hz), 34.2 (q, $^2J_{CF}$ = 29.9 Hz), 32.4, 31.4; **^{19}F NMR (376 MHz, $CDCl_3$):** δ -63.5; **IR (neat, νmax):** 3375, 3279, 1539, 1314, 1120 cm^{-1}; **HRMS-ESI** (*m/z*): Calcd. for $C_{11}H_{14}F_3NO_2S$ $[M-H]^-$ 280.0625, found 280.0625.

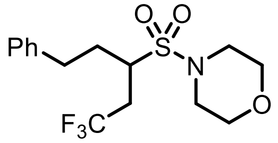

***1-((1,1,1-Trifluoro-5-phenylpentan-3-yl)sulfonyl)piperidine (70)*:** Following the general method of preparation of fluoroalkyl sulfonamide 2.7.3, he title compound **70** was obtained as a transparent oil (95 mg, 54% yield) after purification by silica gel flash chromatography (hexanes:EtOAc = 20:1 to 1:4)

^{1}H NMR (400 MHz, $CDCl_3$): δ 7.36 – 7.27 (m, 2H), 7.26 – 7.17 (m, 3H), 3.70 (dd, J = 5.6, 3.9 Hz, 4H), 3.29 (dd, J = 5.8, 3.8 Hz, 4H), 3.24 – 3.15 (m, 1H), 2.95 – 2.69 (m, 3H), 2.55 – 2.39 (m,

1H), 2.35 – 2.21 (m, 1H), 2.16 – 2.05 (m, 1H); **^{13}C NMR (101 MHz, $CDCl_3$):** δ 140.1, 128.7, 128.4, 126.5, 125.8 (q, $^{1}J_{CF}$ = 277.2 Hz), 54.8 (q, $^{3}J_{CF}$ = 2.4 Hz), 66.7, 46.1, 33.9 (q, $^{2}J_{CF}$ = 29.8 Hz), 32.1, 31.3; **^{19}F NMR (376 MHz, CDCl3):** δ -63.8; **IR (neat, ν_{max}):** 2924, 1740, 1497, 1340, 1261, 1144, 1072 cm^{-1}; **HRMS-ESI** (*m/z*): Calcd. for $C_{16}H_{21}F_3O_2S$ $[M+H]^+$ 352.1189, found 352.1186.

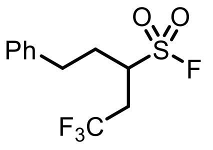

***1,1,1-Trifluoro-5-phenylpentane-3-sulfonyl fluoride (71)[11]*:** Following the general method of fluoroalkyl sulfonyl fluorides 2.7.4, the title compound **71** was obtained as a transparent oil (101 mg, 71% yield) after purification by silica gel flash chromatography (hexanes:EtOAc = 20:1 to 1:1)

^{1}H NMR (400 MHz, $CDCl_3$): δ 7.29 – 7.22 (m, 2H), 7.21 – 7.15 (m, 1H), 7.15 – 7.08 (m, 2H), 3.64 – 3.53 (m, 1H), 2.96 – 2.73 (m, 3H), 2.62 – 2.46 (m, 1H), 2.41 – 2.30 (m, 1H), 2.30 – 2.16 (m, 1H); **^{13}C NMR (101 MHz, $CDCl_3$):** δ 138.7, 128.9, 128.4, 126.9, 124.8 (q, $^{1}J_{CF}$ = 277.6 Hz), 56.2 (q, $^{1}J_{CF}$ = 2.7 Hz), 56.0 (q, $^{3}J_{CF}$ = 2.9 Hz), 33.9 (q, $^{2}J_{CF}$ = 31.0 Hz); **^{19}F NMR (376 MHz, $CDCl_3$):** δ -48.4 (m), -63.8 (m). **IR (neat, ν_{max}):** 2924, 1410, 1260, 1148, 1073 cm^{-1}; **HRMS-ESI** (*m/z*): Calcd. for $C_{11}H_{14}F_3NO_2S$ $[M-H]^-$ 280.0625, found 280.0625.

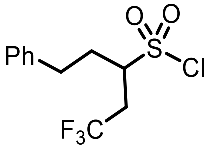

***1,1,1-Trifluoro-5-phenylpentane-3-sulfonyl chloride (72)[12]*:** Following the general method of preparation of fluoroalkyl sulfonamide 2.7.2, The title compound **72** was obtained as a transparent oil (120 mg, 80% yield) after purification by silica gel flash chromatography (hexanes:EtOAc = 20:1 to 1:1)

^{1}H NMR (400 MHz, CDCl$_3$): δ 7.37 – 7.30 (m, 2H), 7.29 – 7.18 (m, 4H), 3.83 – 3.73 (m, 1H), 3.16 – 3.03 (m, 1H), 2.98 – 2.90 (m, 2H), 2.70 – 2.50 (m, 2H), 2.38 – 2.28 (m, 1H); **^{13}C NMR (101 MHz, CDCl$_3$):** δ 138.84, 128.83, 128.42, 126.86, 124.82 (q, $^1J_{CF}$ = 277.8 Hz), δ 68.85 (q, $^3J_{CF}$ = 2.6 Hz), 34.88 (q, $^2J_{CF}$ = 30.8 Hz), 32.10; **^{19}F NMR (376 MHz, CDCl$_3$):** δ -63.4.

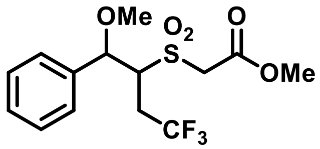

Methyl 2-((4,4,4-trifluoro-1-methoxy-1-phenylbutan-2-yl)sulfonyl)acetate (94): Following the general method of fluoroalkyl sulfonylalkylation 2.4, the title compound **94** was obtained as a transparent oil (159 mg, 45% yield) after purification by silica gel flash chromatography (hexanes:EtOAc = 20:1 to 1:2). The reaction mixture was irradiated for 12 h. Compound was isolated as a separate diastereomers in a 1.2:1 ratio as determined in a crude reaction mixture by ^{1}H NMR.

Minor Diastereomer: **^{1}H NMR (400 MHz, CDCl$_3$):** δ 7.45 – 7.42 (m, 2H), 7.40 – 7.32 (m, 3H), 5.17 (d, J = 3.1 Hz, 1H), 4.03 (d, J = 15.3 Hz, 2H), 3.90 – 3.79 (m, 4H), 3.38 (s, 3H), 3.12 – 2.96 (m, 1H), 2.81 – 2.65 (m, 1H); **^{13}C NMR (101 MHz, CDCl$_3$):** δ 163.7, 136.5, 129.1, 128.7, 127.0 (q, $^1J_{CF}$ = 276.7 Hz), 126.4, 79.1, 63.4, 63.4, 57.5, 57.4, 53.4, 28.7 (q, $^2J_{CF}$ = 31.7 Hz); **^{19}F NMR (376 MHz, CDCl$_3$):** δ -64.6.; *Major Diastereomer:* **^{1}H NMR (400 MHz, CDCl$_3$):** δ 7.43 – 7.40 (m, 3H), 7.38 – 7.34 (m, 2H), 4.76 (d, J = 14.9 Hz, 1H), 4.44 (d, J = 10.4 Hz, 1H), 4.32 (ddd, J = 9.9, 5.4, 3.4 Hz, 1H), 4.13 (d, J = 14.9 Hz, 1H), 3.85 (s, 3H), 3.20 (s, 3H), 2.89 – 2.75 (m, 1H), 2.13 – 2.01 (m, 1H); **^{13}C NMR (101 MHz, CDCl$_3$):** δ 163.8, 135.5, 129.6, 129.1, 129.0, 128.3, 126.4, 125.0 (q, $^1J_{CF}$ = 277.7 Hz), 123.6, 83.4, 60.7, 56.5, 53.2, 28.1(q, $^2J_{CF}$ = 31.1 Hz); **^{19}F NMR (376 MHz, CDCl$_3$):** δ -64.7; HRMS-ESI (m/z): Calcd for $C_{14}H_{17}F_3O_5S$[M+H]$^+$ 355.0822, found 355.0809

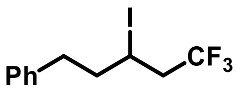

***(5,5,5-trifluoro-3-iodopentyl)benzene (95)*:** Following the general method of preparation of iodotriflouromethylation 3.7, The title compound **95** was obtained as a transparent oil (115 mg, 70% yield) after purification by silica gel flash chromatography (hexanes:EtOAc = 50:1 to 1:1).

^{1}H NMR (400 MHz, CDCl$_3$): δ 7.31 (dd, J = 8.5, 6.4 Hz, 2H), 7.25 – 7.18 (m, 3H), 4.20 – 4.07 (m, 1H), 3.01 – 2.78 (m, 3H), 2.77 – 2.67 (m, 1H), 2.16 – 2.01 (m, 2H); **^{13}C NMR (101 MHz, CDCl$_3$):** δ 139.9, 128.6, 128.5, 128.3, 126.4, 125.5 (q, $^1J_{CF}$ = 278.9 Hz), 44.9 (q, $^1J_{CF}$ = 28.4 Hz), 41.1, 35.5, 20.9(8), 20.9(5), 20.9(2), 20.89; **^{19}F NMR (376 MHz, CDCl$_3$):** δ -63.7. **IR (neat, ν_{max}):** 2924, 1410, 1260, 1148, 1073 cm^{-1}; **HRMS-ESI** (*m/z*): Calcd. for $C_{11}H_{12}F_3I$ [M]$^+$ 327.9931, found 327.9932.

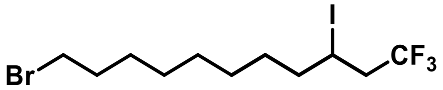

11-bromo-1,1,1-trifluoro-3-iodoundecane (116)*[13]*: Following the general method of preparation of iodotriflouromethylation 3.7, The title compound **116** was obtained as a transparent oil (126 mg, 61% yield) after purification by silica gel flash chromatography (hexanes:EtOAc = 99:1 to 1:1).

^{1}H NMR (400 MHz, CDCl$_3$): δ 4.25 – 4.14 (m, 1H), 3.41 (t, J = 6.9 Hz, 2H), 2.98 – 2.84 (m, 1H), 2.84 – 2.71 (m, 1H), 1.90 – 1.68 (m, 5H), 1.46 – 1.29 (m, 12H); **^{13}C NMR (101 MHz, CDCl$_3$):** δ 125.6 (d, $^1J_{CF}$ = 278.7 Hz), 44.9 (q, $^2J_{CF}$ = 28.2 Hz), 39.6, 34.0, 32.8, 29.4, 29.3, 29.2, 28.7, 28.4, 28.1, 21.9, 21.8(4), 21.8(1), 21.8; **^{19}F NMR (376 MHz, CDCl$_3$):** δ -63.9. . **IR (neat, ν_{max}):** 2927, 1253, 1144, 705 cm^{-1};

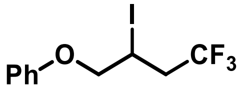

***(4,4,4-trifluoro-2-iodobutoxy)benzene (117)*:** Following the general method of preparation of iodotriflouromethylation 3.7, The title compound **117** was obtained as a transparent oil (76 mg, 46% yield) after purification by silica gel flash chromatography (hexanes:EtOAc = 50:1 to 1:1).

^{1}H NMR (400 MHz, $CDCl_3$): δ 7.34 – 7.28 (m, 2H), 7.05 – 6.98 (m, 1H), 6.94 – 6.87 (m, 2H), 4.45 – 4.35 (m, 1H), 4.34 – 4.25 (m, 1H), 4.21 – 4.11 (m, 1H), 3.23 – 3.06 (m, 1H), 2.87 – 2.72 (m, 1H); **^{13}C NMR (101 MHz, $CDCl_3$):** δ 157.6, 129.7, 123.5 (d, $^{1}J_{CF}$ = 278.7 Hz), 121.8, 114.8, 72.5, 40.9 (q, $^{2}J_{CF}$ = 28.2 Hz), 14.2(0), 14.1(7); **^{19}F NMR (376 MHz, $CDCl_3$):** -64.2. **IR (neat, ν_{max}):** 2928, 1597, 1495, 1236, 1145, 752 cm^{-1}**; HRMS-ESI** (*m/z*): Calcd. for $C_{11}H_{12}F_3I$ $[M]^+$ 329.9723, found 329.9723.

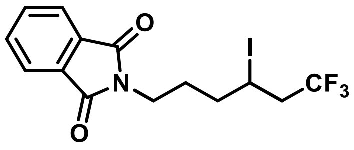

***2-(6,6,6-trifluoro-4-iodohexyl)isoindoline-1,3-dione (118)[14]*:** Following the general method of preparation of iodotriflouromethylation 3.7, The title compound **118** was obtained as a transparent oil (123 mg, 60% yield) after purification by silica gel flash chromatography (hexanes:EtOAc = 50:1 to 1:1).

^{1}H NMR (400 MHz, $CDCl_3$): δ 7.85 (dd, J = 5.4, 3.1 Hz, 2H), 7.72 (dd, J = 5.4, 3.1 Hz, 2H), 4.29 – 4.17 (m, 1H), 3.81 – 3.65 (m, 2H), 2.99 – 2.68 (m, 2H), 2.03 – 1.93 (m, 1H); **^{13}C NMR (101 MHz, $CDCl_3$):** δ 168.3, 134.1, 134.0, 132.0, 125.4 (d, $^{1}J_{CF}$ = 278.7 Hz), 123.31, 44.8 (q, $^{2}J_{CF}$ = 28.4 Hz), 36.7, 36.7, 28.7, 20.0; **^{19}F NMR (376 MHz, $CDCl_3$):** δ -63.9. IR (neat, ν_{max}): 2935, 1772, 1706, 1395, 1103, 718 cm^{-1}; **HRMS-ESI** (*m/z*): Calcd. for $C_{14}H_{13}F_3INO_2Na$ $[M+Na]^+$ 433.9836 found 433.9835.

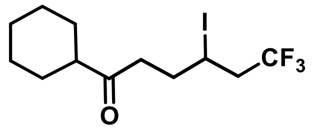

***1-Cyclohexyl-6,6,6-trifluoro-4-iodohexan-1-one (119)*:** Following the general method of preparation of iodotriflouromethylation 3.7, The title compound **119** was obtained as a transparent oil (118 mg, 65% yield) after purification by silica gel flash chromatography (hexanes:EtOAc = 50:1 to 1:1).

^{1}H NMR (400 MHz, CDCl$_3$): δ 4.29 – 4.04 (m, 1H), 2.99 – 2.86 (m, 1H), 2.85 – 2.72 (m, 1H), 2.72 – 2.60 (m, 2H), 2.40 – 2.30 (m, 1H), 2.14 – 2.04 (m, 1H), 2.00 – 1.73 (m, 5H), 1.72 – 1.61 (m, 1H), 1.45 – 1.12 (m, 5H); **^{13}C NMR (101 MHz, CDCl$_3$):** δ 212.0, 125.4 (q, $^1J_{CF}$ = 278.7 Hz), 50.9, 45.13 (q, $^2J_{CF}$ = 28.5 Hz), 40.5, 33.5, 28.6, 28.4, 25.8, 25.6, 25.6, 21.3; **^{19}F NMR (376 MHz, CDCl$_3$):** δ -63.8; **IR (neat, ν_{max}):** 2929, 1706, 1290, 1144, 731 cm^{-1}; **HRMS-ESI** (*m/z*): Calcd. for $C_{12}H_{19}F_3IO$ [M+H]$^+$ 363.0426, found 363.0427.

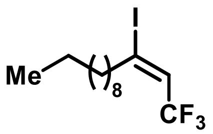

***1,1,1-Trifluoro-3-iodotridec-2-ene (120)*[15]:** Following the general method of preparation of iodotriflouromethylation 3.7. The title compound **120** was obtained as a transparent oil (101 mg, 56% yield) after purification by silica gel flash chromatography (hexanes:EtOAc = 50:1 to 1:1). Compound was isolated as a mixture of *E* and *Z* isomers in a 2:1 ratio as determined in a crude reaction mixture by ^{1}H NMR.

^{1}H NMR (400 MHz, CDCl$_3$): δ 6.45 – 6.35 (m, .67H), 6.33 – 6.24 (m, 0.33H), 2.65 – 2.56 (m, 2H), 1.63 – 1.52 (m, 2H), 1.38 – 1.24 (m, 14H), 0.89 (t, *J* = 6.8 Hz, 3H); **^{13}C NMR (101 MHz, CDCl$_3$):** δ 129.6, 129.3, 128.9, 128.6, 125.1, 124.7, 123.4, 121.3, 121.2, 121.2, 120.7, 47.4, 47.1, 40.9, 38.3, 32.4, 32.2, 32.1, 29.8, 29.7, 29.6, 29.5, 29.4, 29.4, 29.3, 29.2, 28.5, 28.3, 26.3, 26.3,

22.9, 14.3 (*Due to the presence of cis–trans isomers and additional fluorine–carbon coupling, the ^{13}C NMR spectra appear complex. For this reason, J-values are not reported.*); **^{19}F NMR (376 MHz, $CDCl_3$):** δ -57.9, -60.1; **HRMS-ESI** (*m/z*): Calcd. for $C_{13}H_{23}F_3I$ $[M+H]^+$ 363.0792, found 363.0478.

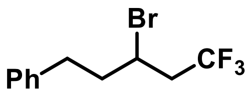

(3-Bromo-5,5,5-trifluoropentyl)benzene (104)[16]**:** Following the general method of preparation of bromotriflouromethylation 3.9 – method A, the title compound **104** was obtained as a transparent oil (112 mg, 80% yield) after purification by silica gel flash chromatography (hexanes:EtOAc = 50:1 to 1:1).

^1H NMR (400 MHz, $CDCl_3$): δ 7.36 – 7.27 (m, 2H), 7.25 – 7.13 (m, 3H), 4.16 – 4.05 (m, 1H), 2.99 – 2.90 (m, 1H), 2.88 – 2.65 (m, 3H), 2.26 – 2.08 (m, 2H).; **^{13}C NMR (101 MHz, $CDCl_3$):** δ 140.0, 128.6, 128.5, 126.4, 125.21 (d, $^1J_{CF}$ = 278.3 Hz), 44.3, 43.1 (q, $^2J_{CF}$ = 28.6 Hz), 40.0, 33.3; **^{19}F NMR (376 MHz, $CDCl_3$):** δ -63.66 ; **HRMS-ESI** (*m/z*): Calcd. for $C_{11}H_{13}BrF_3$ $[M+H]^+$ 281.0148, found 281.0148.

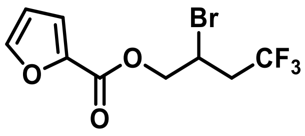

2-Bromo-4,4,4-trifluorobutyl furan-2-carboxylate (121)**:** Following the general method of preparation of bromotriflouromethylation 3.9 – method A, the title compound **121** was obtained as a transparent oil (62 mg, 42% yield) after purification by silica gel flash chromatography (hexanes:EtOAc = 50:1 to 1:1).

^1H NMR (400 MHz, $CDCl_3$): δ 7.46 (d, J = 1.9 Hz, 1H), 7.09 (dd, J = 4.4, 1.5 Hz, 1H), 6.38 (dd, J = 3.6, 1.8 Hz, 1H), 4.45 (dd, J = 11.8, 5.6 Hz, 1H), 4.36 (dd, J = 11.9, 6.2 Hz, 1H), 4.21 (dt, J =

7.8, 5.7 Hz, 1H), 2.84 – 2.54 (m, 2H); **^{13}C NMR (101 MHz, $CDCl_3$):** δ 157.6, 147.0, 143.6, 125.1 (q, $^{1}J_{CF}$ = 277.7 Hz), 119.0, 112.1, 66.6, 39.7 (q, $^{2}J_{CF}$ = 29.5 Hz), 38.7 (q, $^{3}J_{CF}$ = 3.2 Hz).; **^{19}F NMR (376 MHz, $CDCl_3$):** δ -64.0. IR (neat, ν_{max}): 2940, 1720, 1473, 1294, 1177, 750 cm^{-1}; **HRMS-ESI** (*m/z*): Calcd. for $C_9H_8BrF_3O_3Na$ $[M+Na]^+$ 363.0792, found 363.0478.

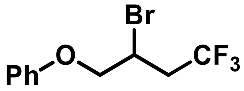

***(2-Bromo-4,4,4-trifluorobutoxy)benzene (122)*:** Following the general method of preparation of bromotriflouromethylation 3.9 – method A, the title compound **122** was obtained as a transparent oil (86 mg, 61% yield) after purification by silica gel flash chromatography (hexanes:EtOAc = 50:1 to 1:1).

^{1}H NMR (400 MHz, $CDCl_3$): δ 7.37 – 7.28 (m, 2H), 7.05 – 6.99 (m, 1H), 6.94 – 6.89 (m, 2H), 4.41 – 4.28 (m, 2H), 4.16 (dd, J = 10.1, 7.0 Hz, 1H), 3.18 – 3.04 (m, 1H), 2.83 – 2.66 (m, 1H).; **^{13}C NMR (101 MHz, $CDCl_3$):** δ 157.9, 129.8, 125.52 (d, $^{1}J_{CF}$ = 277.5 Hz), 122.0, 114.9, 70.9, 40.1, 39.8, 39.7 (q, $^{3}J_{CF}$ = 3.2 Hz), 39.6 (q, $^{2}J_{CF}$ = 29.4 Hz), 39.5, 39.2; **^{19}F NMR (376 MHz, $CDCl_3$):** δ -64.0. IR (neat, ν_{max}): 2930, 1598, 1496, 1237, 1165, 751 cm^{-1}; **HRMS-ESI** (*m/z*): Calcd. for $C_{10}H_{10}BrF_3O$ $[M]^+$ 281.9862, found 282.1278.

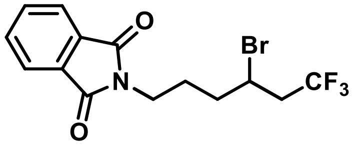

***(2-(4-Bromo-6,6,6-trifluorohexyl)isoindoline-1,3-dione(123)*[16]:** Following the general method of preparation of bromotriflouromethylation 3.9 – method A, the title compound **123** was obtained as a transparent oil (136 mg, 75% yield) after purification by silica gel flash chromatography (hexanes:EtOAc = 50:1 to 1:1).

^{1}H NMR (400 MHz, $CDCl_3$): δ 7.46 (d, J = 1.9 Hz, 1H), 7.09 (dd, J = 4.4, 1.5 Hz, 1H), 6.38 (dd, J = 3.6, 1.8 Hz, 1H), 4.45 (dd, J = 11.8, 5.6 Hz, 1H), 4.36 (dd, J = 11.9, 6.2 Hz, 1H), 4.21 (dt, J =

7.8, 5.7 Hz, 1H), 2.84 – 2.54 (m, 2H); **^{13}C NMR (101 MHz, CDCl$_3$):** δ 168.3, 134.1, 132.0, 125.2 (d, $^{1}J_{CF}$ = 277.7 Hz) 123.3, 44.0, 43.1 (q, $^{2}J_{CF}$ = 28.5 Hz). 36.9, 35.6, 26.5; **^{19}F NMR (376 MHz, CDCl$_3$):** δ-63.8. IR (neat, **ν$_{max}$**): 2944, 1769, 1702, 1288, 721 cm^{-1};

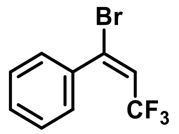

***(E)-(1-Bromo-3,3,3-trifluoroprop-1-en-1-yl)benzene (124)*[16]:** Following the general method of preparation of bromotriflouromethylation 3.9 – method A, the title compound **124** was obtained as a transparent oil (65 mg, 26% yield) after purification by silica gel flash chromatography (hexanes:EtOAc = 50:1 to 1:1). Crude reaction showed a mixture of *E* and *Z* isomers in a 1.4:1ratio by ^{1}H NMR. The isomeric ratio was determined by integration of the diagnostic olefinic proton signals at δ 6.57 (q, *J* = 7.3 Hz, 1H, *Z* isomer) and 6.44 (q, *J* = 7.4 Hz, 1H, *E* isomer). Only the *E* isomer was isolated in analytically pure form; the reported isolated yield corresponds exclusively to the *E* isomer.

^{1}H NMR (400 MHz, CDCl$_3$): δ 7.44 - 7.40 (m, 5H), 6.44 (q, *J* = 7.4 Hz, 1H); **^{13}C NMR (101 MHz, CDCl$_3$):** δ 137.1, 135.8 (q, $^{3}J_{CF}$ = 6.5 Hz), 130.0, 128.2, 128.0 (q, $^{4}J_{CF}$ = 1.7 Hz), 121.8 (q, $^{2}J_{CF}$ = 35.3 Hz), 121.4 (q, $^{1}J_{CF}$ = 272.2 Hz); **^{19}F NMR (376 MHz, CDCl$_3$):** δ -57.2; IR (neat, **ν$_{max}$**): 3070, 1600, 1495, 1400, 1200 cm^{-1}; **HRMS-ESI** (*m/z*): Calcd. for $C_9H_5BrF_3$ [M-H]$^-$ 248.9532, found 249.0386.

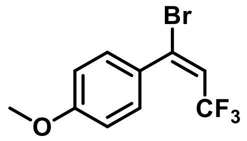

***(E)-1-(1-Bromo-3,3,3-trifluoroprop-1-en-1-yl)-4-methoxybenzene (125)*[17]:** Following the general method of preparation of bromotriflouromethylation 3.9 – method A, the title compound **125** was obtained as a transparent oil (84 mg, 30% yield) after purification by silica gel flash chromatography (hexanes:EtOAc = 50:1 to 1:1). Crude reaction showed a mixture of *E* and *Z*

isomers in a 1.7:1 ratio by ^{1}H NMR. The isomeric ratio was determined by integration of the diagnostic olefinic proton signals at δ 6.49 (q, J = 7.3 Hz, 1H, Z isomer) and 6.38 (q, J = 7.5 Hz, 1H, E isomer). Only the E isomer was isolated in analytically pure form; the reported isolated yield corresponds exclusively to the E isomer.

^{1}H NMR (400 MHz, $CDCl_3$): δ 7.43 – 7.37 (m, 2H), 6.95 – 6.89 (m, 2H), 6.38 (q, J = 7.5 Hz, 1H), 3.86 (s, 3H); **^{13}C NMR (101 MHz, $CDCl_3$):** δ 160.9, 136.1 (q, $^3J_{CF}$ = 6.5 Hz), 129.9 (q, $^4J_{CF}$ 2.0 Hz), 121.6 (q, $^1J_{CF}$ 272.0 Hz), 120.8 (q, $^2J_{CF}$ = 35.2 Hz), 113.6, 55.3; **^{19}F NMR (376 MHz, $CDCl_3$):** δ -57.1; IR (neat, ν_{max}): 2960, 1750, 1270, 1180 cm^{-1}; **HRMS-ESI** (*m/z*): Calcd. for $C_{10}H_7BrF_3O$ $[M-H]^-$ 278.9638, found 279.1642.

6.0 References

(1) Shavnya, A.; Coffey, S. B.; Hesp, K. D.; Ross, S. C.; Tsai, A. S. Reaction of Alkyl Halides with Rongalite: One-Pot and Telescoped Syntheses of Aliphatic Sulfonamides, Sulfonyl Fluorides, and Unsymmetrical Sulfones. *Organic Letters* **2016**, *18* (22), 5848-5851. DOI: 10.1021/acs.orglett.6b02894.
(2) Emmett, E. J.; Hayter, B. R.; Willis, M. C. Palladium-Catalyzed Synthesis of Ammonium Sulfinates from Aryl Halides and a Sulfur Dioxide Surrogate: A Gas- and Reductant-Free Process. *Angewandte Chemie International Edition* **2014**, *53* (38), 10204-10208. DOI: https://doi.org/10.1002/anie.201404527.
(3) Baskin, J. M.; Wang, Z. A mild, convenient synthesis of sulfinic acid salts and sulfonamides from alkyl and aryl halides. *Tetrahedron Letters* **2002**, *43* (47), 8479-8483. DOI: https://doi.org/10.1016/S0040-4039(02)02073-7.
(4) Chandu, P.; Ghosh, K. G.; Das, D.; Sureshkumar, D. Photoredox catalysed allylic trifluoromethylation via ring opening of vinyl cyclopropanes using Langlois reagent. *Tetrahedron* **2019**, *75* (45), 130641. DOI: https://doi.org/10.1016/j.tet.2019.130641.
(5) Nasireddy, S. R.; Upreti, G. C.; Singh, A. Photochemical Trifluoromethylative Difunctionalization of Styrenes and Phenylacetylenes via a Catalytic EDA Platform. *European Journal of Organic Chemistry* **2024**, *27* (17), e202400114. DOI: https://doi.org/10.1002/ejoc.202400114.
(6) Fischer, D. M.; Lindner, H.; Amberg, W. M.; Carreira, E. M. Intermolecular Organophotocatalytic Cyclopropanation of Unactivated Olefins. *Journal of the American Chemical Society* **2023**, *145* (2), 774-780. DOI: 10.1021/jacs.2c11680.
(7) Hatchard, C. G.; Parker, C. A.; Bowen, E. J. A new sensitive chemical actinometer - II. Potassium ferrioxalate as a standard chemical actinometer. *Proceedings of the Royal Society of London. Series A. Mathematical and Physical Sciences* **1956**, *235* (1203), 518-536. DOI: doi:10.1098/rspa.1956.0102.
(8) Kuhn, H. J.; Braslavsky, S. E.; Schmidt, R. Chemical actinometry (IUPAC Technical Report). *Pure and Applied Chemistry* **2004**, *76* (12), 2105-2146. DOI: doi:10.1351/pac200476122105 (acccessed 2024-11-18).
(9) Hou, X.; Liu, H.; Huang, H. Iron-catalyzed fluoroalkylative alkylsulfonylation of alkenes via radical-anion relay. *Nature Communications* **2024**, *15* (1), 1480. DOI: 10.1038/s41467-024-45867-y.
(10) Li, H.; Zhang, Y.; Yang, X.; Deng, Z.; Zhu, Z.; Zhou, P.; Ouyang, X.; Yuan, Y.; Chen, X.; Yang, L.; et al. Synthesis of Multifluoromethylated γ-Sultines by a Photoinduced Radical Addition–Polar Cyclization.

Angewandte Chemie International Edition **2023**, *62* (15), e202300159. DOI: https://doi.org/10.1002/anie.202300159.
(11) Liu, Y.; Wu, H.; Guo, Y.; Xiao, J.-C.; Chen, Q.-Y.; Liu, C. Trifluoromethylfluorosulfonylation of Unactivated Alkenes Using Readily Available Ag(O2CCF2SO2F) and N-Fluorobenzenesulfonimide. *Angewandte Chemie International Edition* **2017**, *56* (48), 15432-15435. DOI: https://doi.org/10.1002/anie.201709663.
(12) Pagire, S. K.; Paria, S.; Reiser, O. Synthesis of β-Hydroxysulfones from Sulfonyl Chlorides and Alkenes Utilizing Visible Light Photocatalytic Sequences. *Organic Letters* **2016**, *18* (9), 2106-2109. DOI: 10.1021/acs.orglett.6b00734.
(13) Beniazza, R.; Douarre, M.; Lastécouères, D.; Vincent, J.-M. Metal-free and light-promoted radical iodotrifluoromethylation of alkenes with Togni reagent as the source of CF3 and iodine. *Chemical Communications* **2017**, *53* (25), 3547-3550, 10.1039/C7CC00214A. DOI: 10.1039/C7CC00214A.
(14) Xu, T.; Cheung, C. W.; Hu, X. Iron-Catalyzed 1,2-Addition of Perfluoroalkyl Iodides to Alkynes and Alkenes. *Angewandte Chemie International Edition* **2014**, *53* (19), 4910-4914. DOI: https://doi.org/10.1002/anie.201402511.
(15) Yajima, T.; Murase, M.; Ofuji, Y. Visible Light-Induced Radical Iodoperfluoroalkylation of Unactivated Olefins Cooperatively Catalyzed by Enamines and Amines. *European Journal of Organic Chemistry* **2020**, *2020* (25), 3808-3811. DOI: https://doi.org/10.1002/ejoc.201901896.
(16) Ouyang, Y.; Tong, C.-L.; Xu, X.-H.; Qing, F.-L. Copper and Zinc Copromoted Bromo(chloro)trifluoromethylation of Alkenes and Alkynes with Trifluoromethanesulfonic Anhydride. *Organic Letters* **2021**, *23* (2), 346-350. DOI: 10.1021/acs.orglett.0c03855.
(17) Shang, X.-J.; Luo, R.; Zhu, J.; Liu, Z.-Q. Free-radical promoted bromotrifluoromethylation of arylalkynes with CF3SO2Na and $NaBrO_3$. *Tetrahedron Letters* **2021**, *62*, 152683. DOI: https://doi.org/10.1016/j.tetlet.2020.152683.

7.0 Spectra

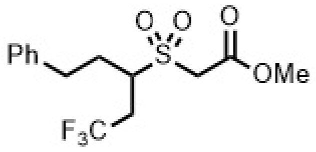

3

Parameter	Value
Solvent	CDCl3
Temperature	298.0
Acquisition Date	2024-02-27T22:12:07
Spectrometer Frequency	400.13
Nucleus	1H

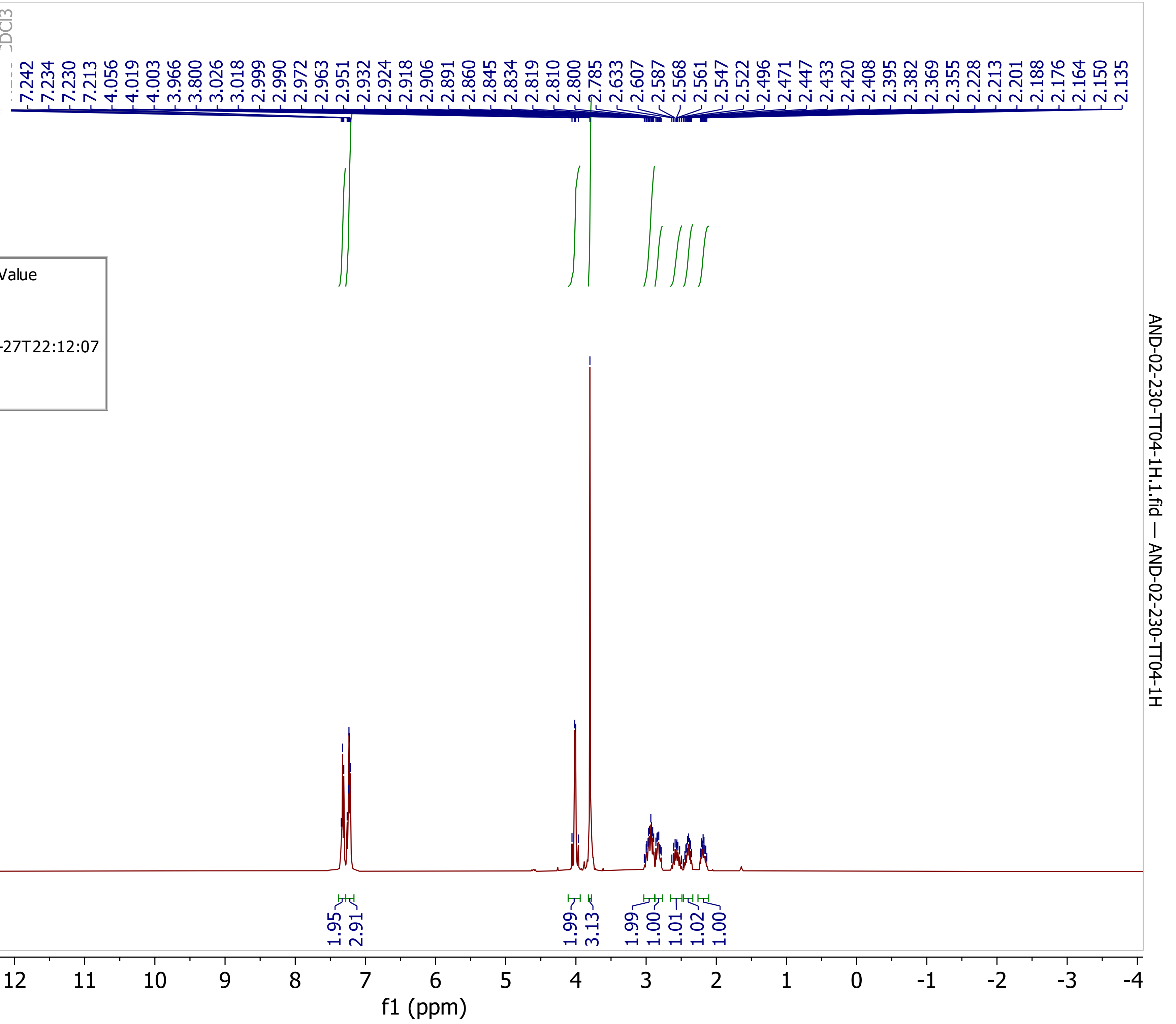

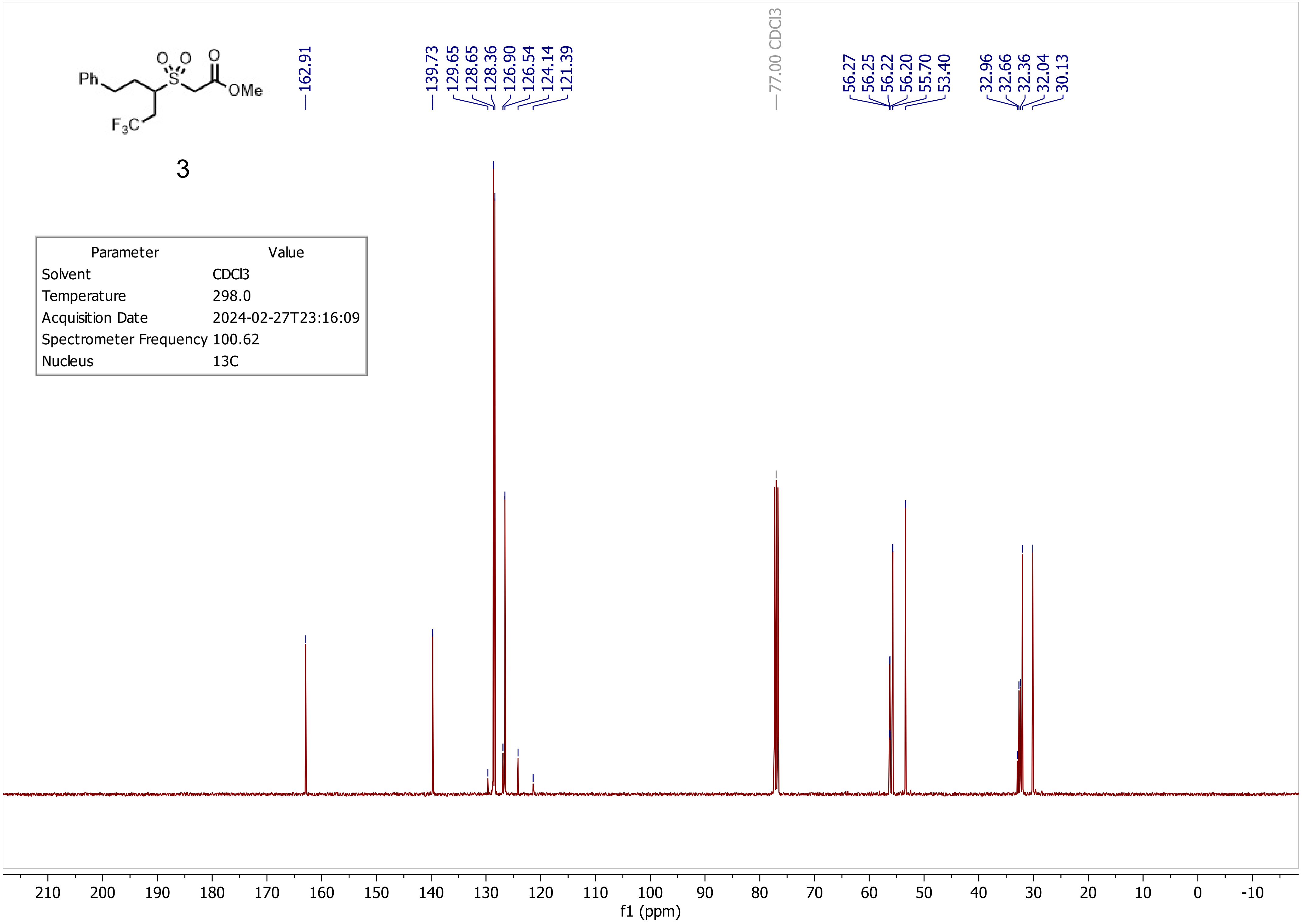

Parameter	Value
Solvent	CDCl3
Temperature	298.0
Acquisition Date	2024-02-27T23:16:09
Spectrometer Frequency	100.62
Nucleus	13C

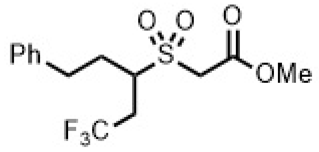

3

Parameter	Value
Solvent	CDCl3
Temperature	298.0
Acquisition Date	2024-02-27T22:15:53
Spectrometer Frequency	376.46
Nucleus	19F

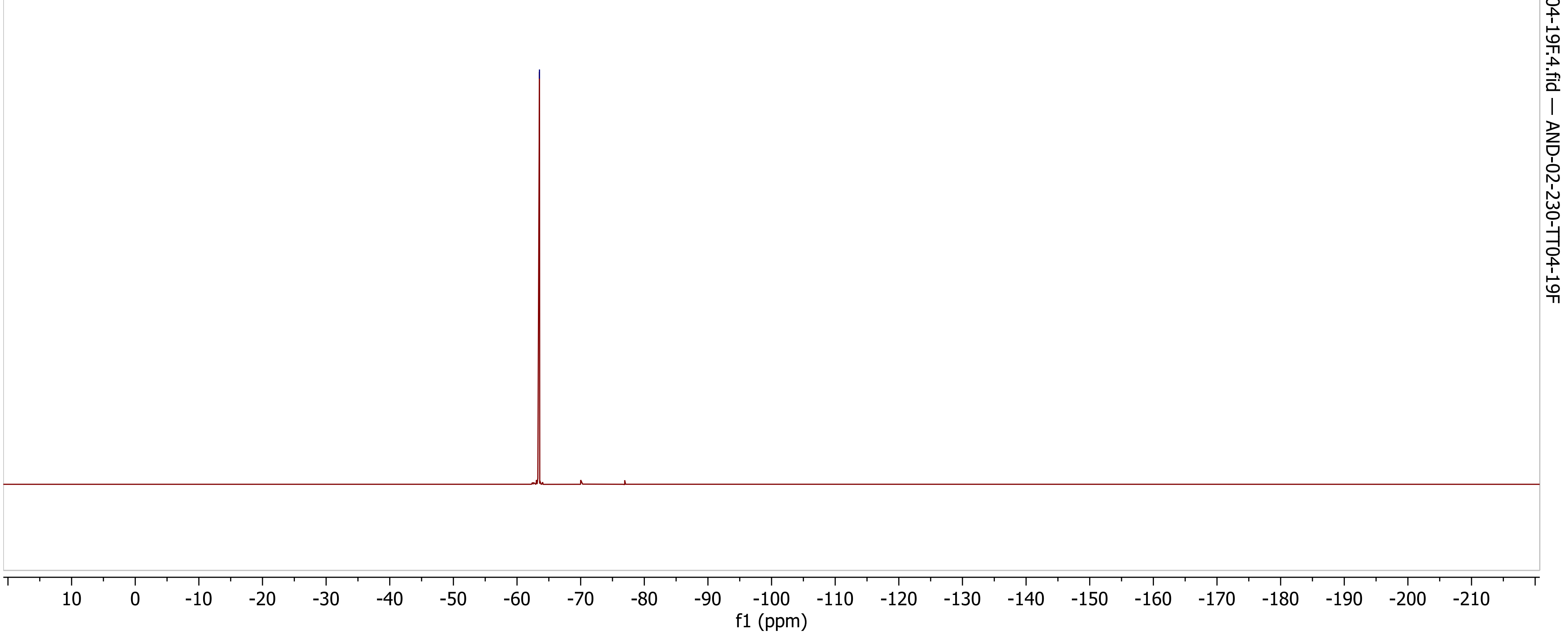

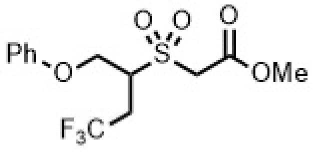

6

Parameter	Value
Solvent	CDCl3
Temperature	298.0
Acquisition Date	2024-03-11T20:41:43
Spectrometer Frequency	400.13
Nucleus	1H

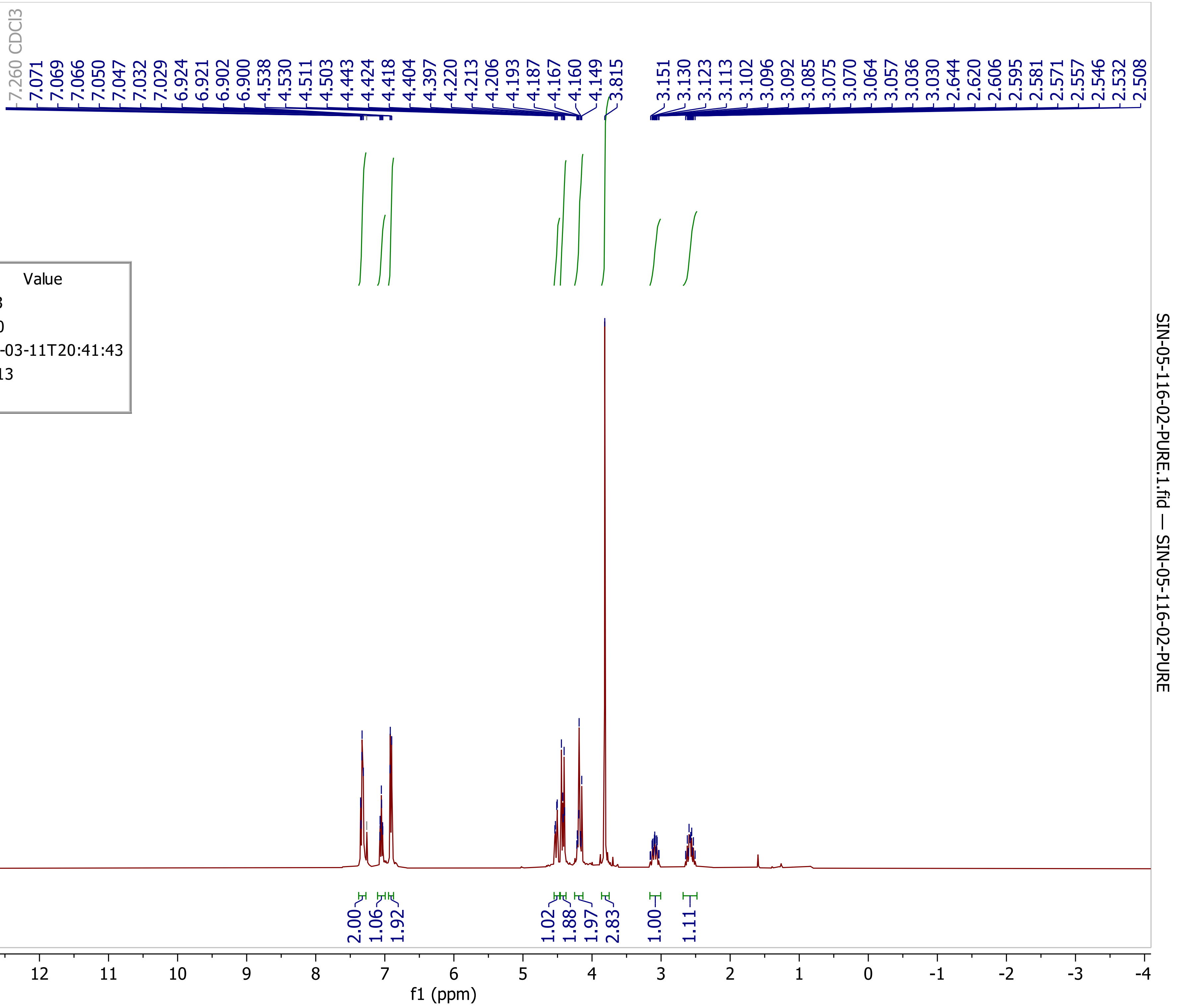

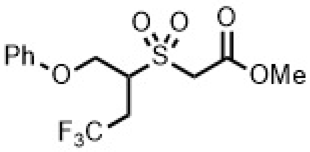

6

Parameter	Value
Solvent	CDCl3
Temperature	298.0
Acquisition Date	2024-03-12T03:01:20
Spectrometer Frequency	100.62
Nucleus	13C

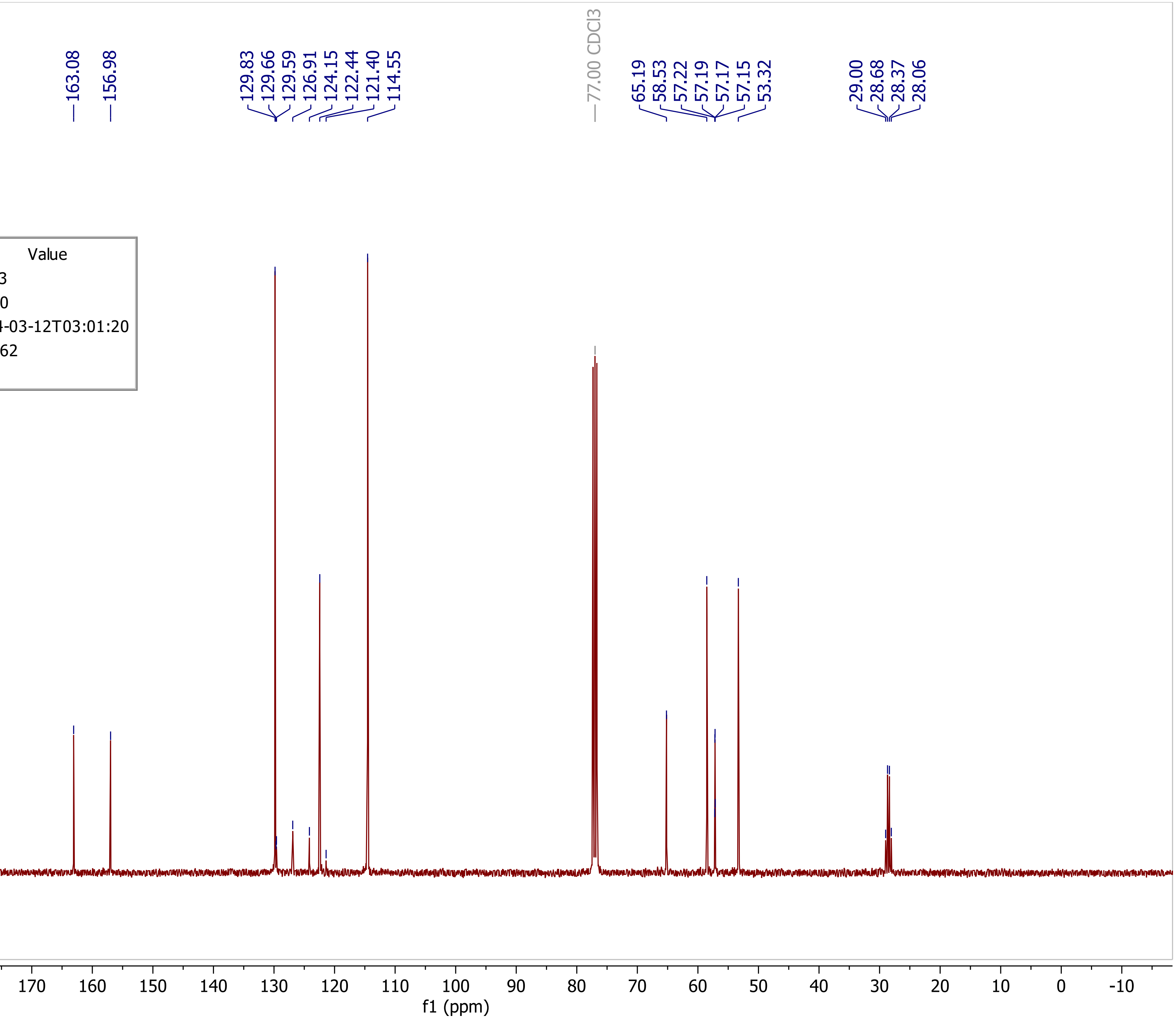

SIN-05-116-02-PURE-C13.5.fid — SIN-05-116-02-PURE-C13

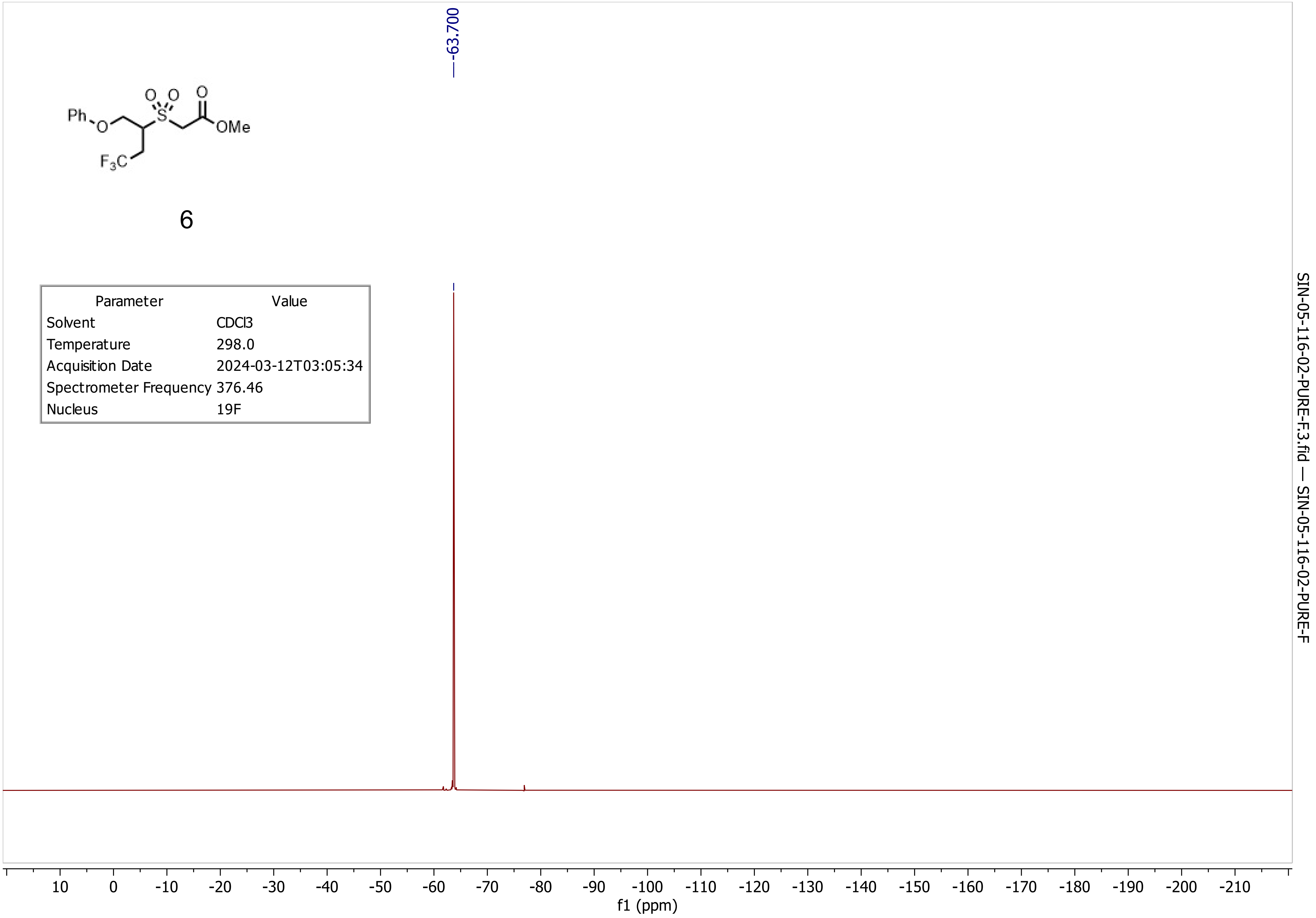

Parameter	Value
Solvent	CDCl3
Temperature	298.0
Acquisition Date	2024-03-12T03:05:34
Spectrometer Frequency	376.46
Nucleus	19F

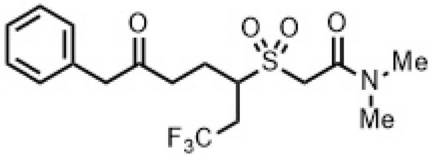

7

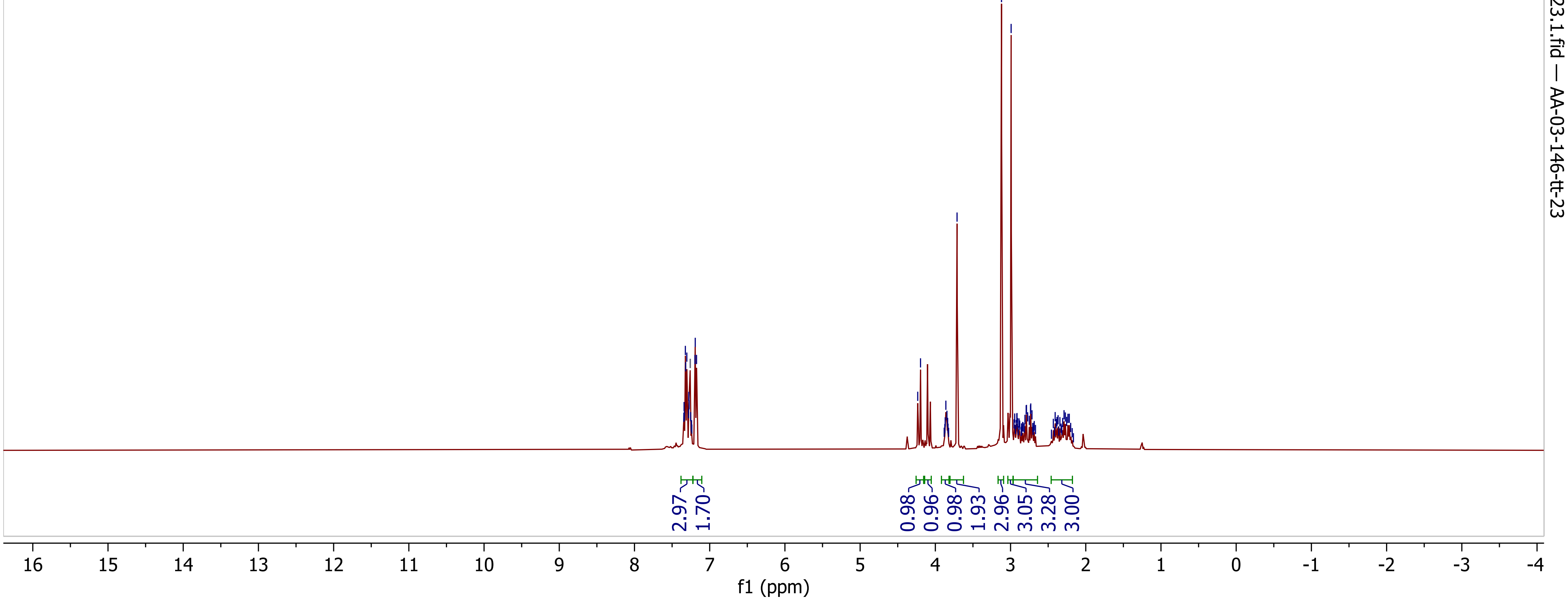

Parameter	Value
Solvent	CDCl3
Temperature	298.0
Acquisition Date	2024-09-18T21:27:23
Spectrometer Frequency	400.13
Nucleus	1H

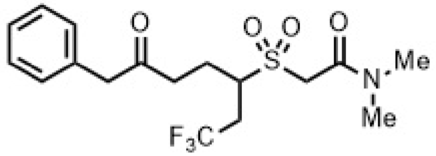

7

Parameter	Value
Solvent	CDCl3
Temperature	298.0
Acquisition Date	2024-09-19T03:28:35
Spectrometer Frequency	100.62
Nucleus	13C

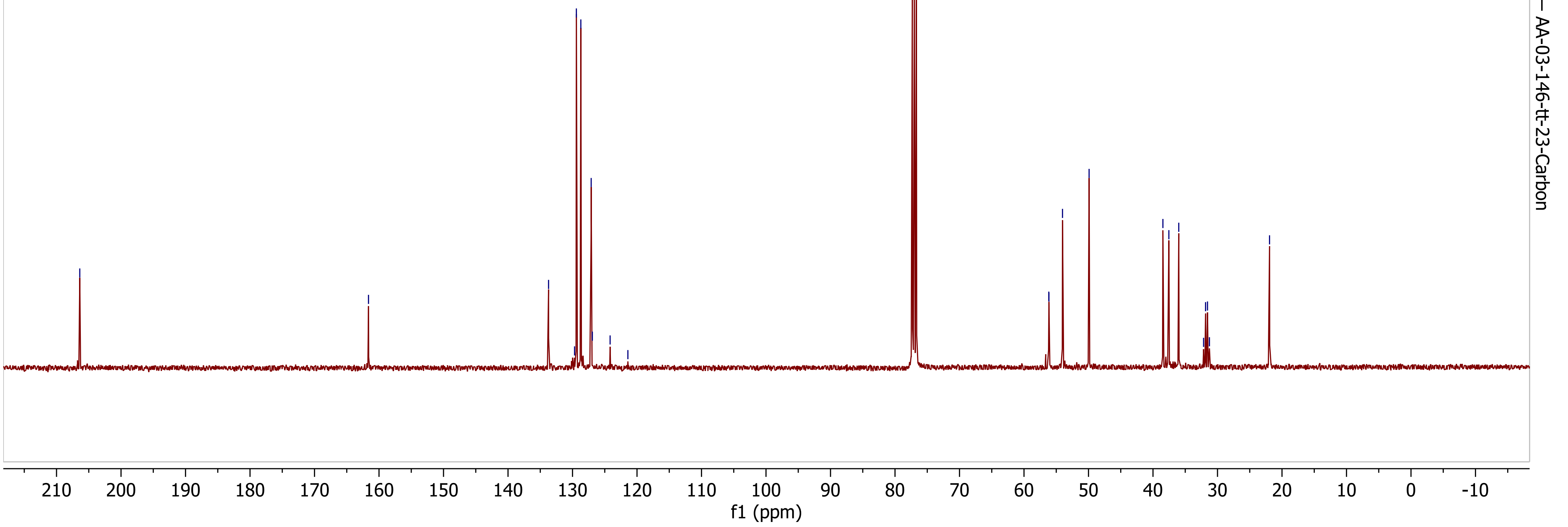

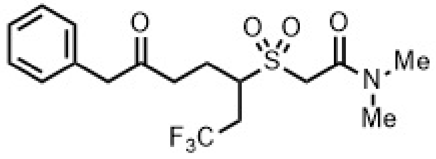

7

Parameter	Value
Solvent	CDCl3
Temperature	298.0
Acquisition Date	2024-09-19T02:21:44
Spectrometer Frequency	376.46
Nucleus	19F

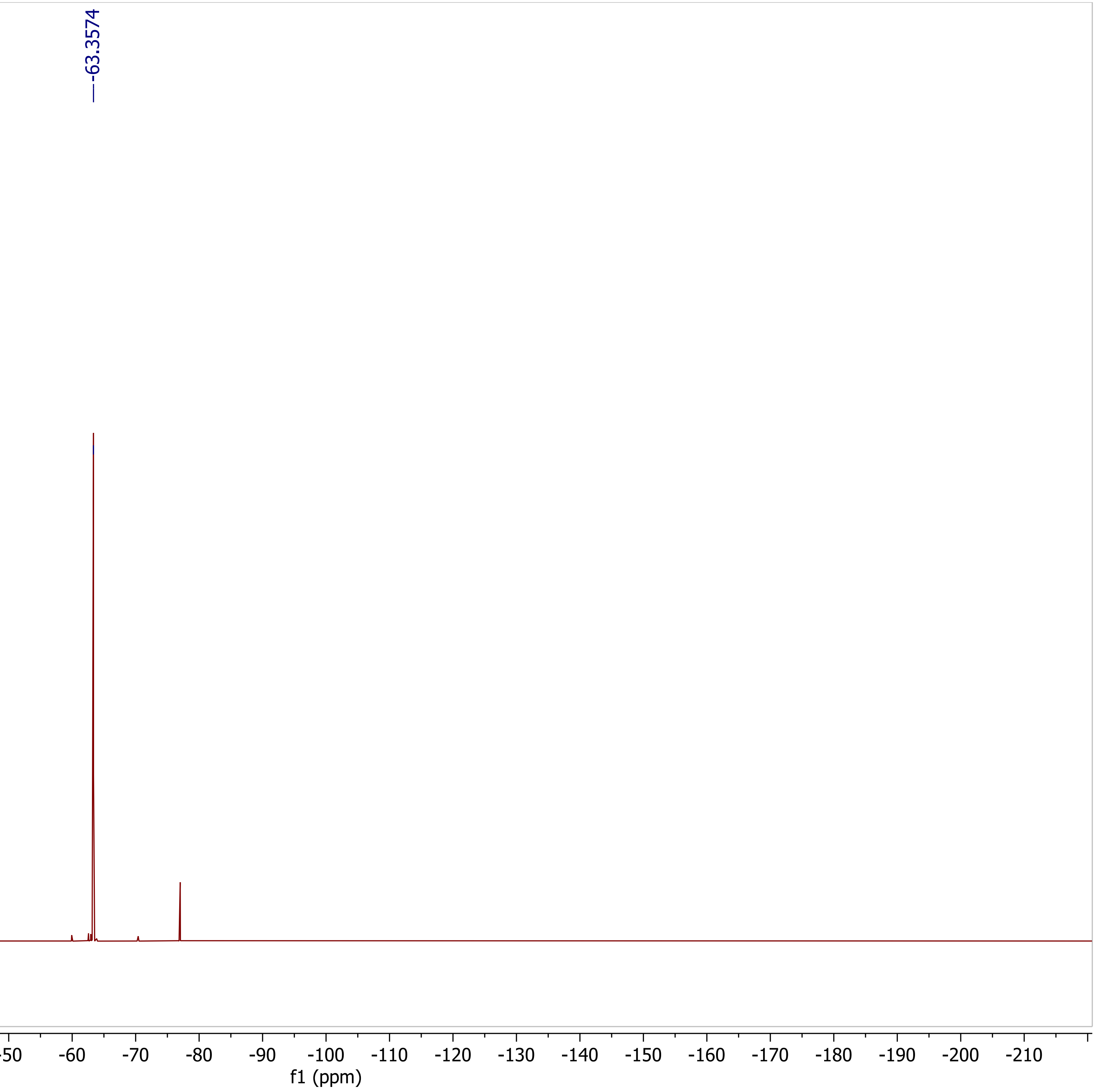

AA-03-146-tt-22-Fluorine.3.fid — AA-03-146-tt-22-Fluorine

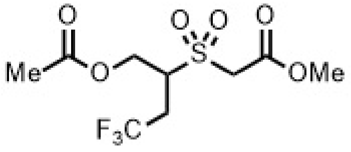

8

Parameter	Value
Solvent	CDCl3
Temperature	298.0
Acquisition Date	2024-03-04T20:17:04
Spectrometer Frequency	400.13
Nucleus	1H

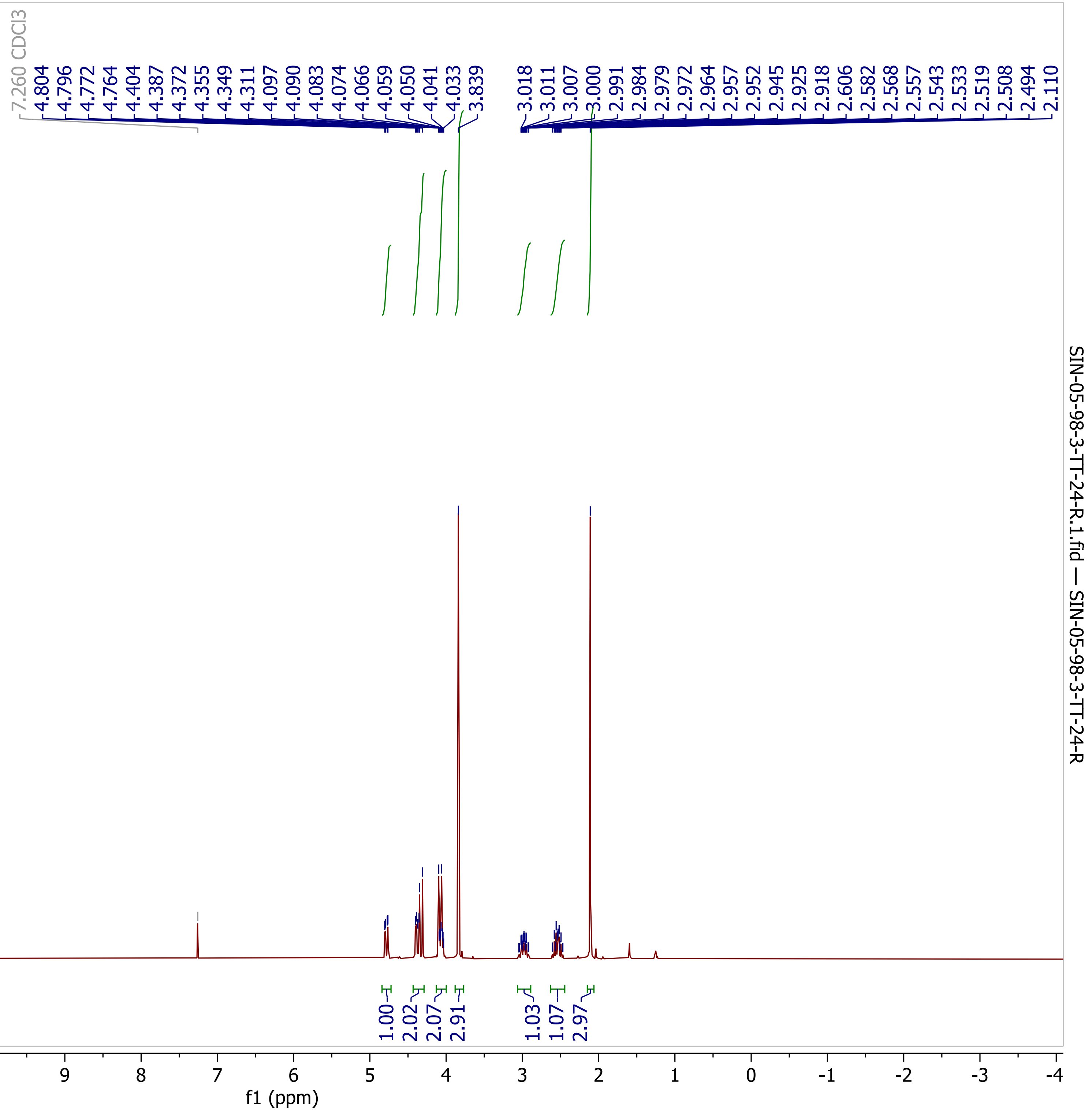

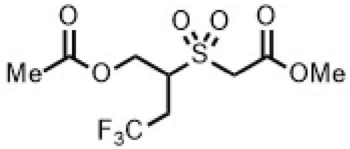

8

Parameter	Value
Solvent	CDCl3
Temperature	298.0
Acquisition Date	2024-03-05T06:29:33
Spectrometer Frequency	100.62
Nucleus	13C

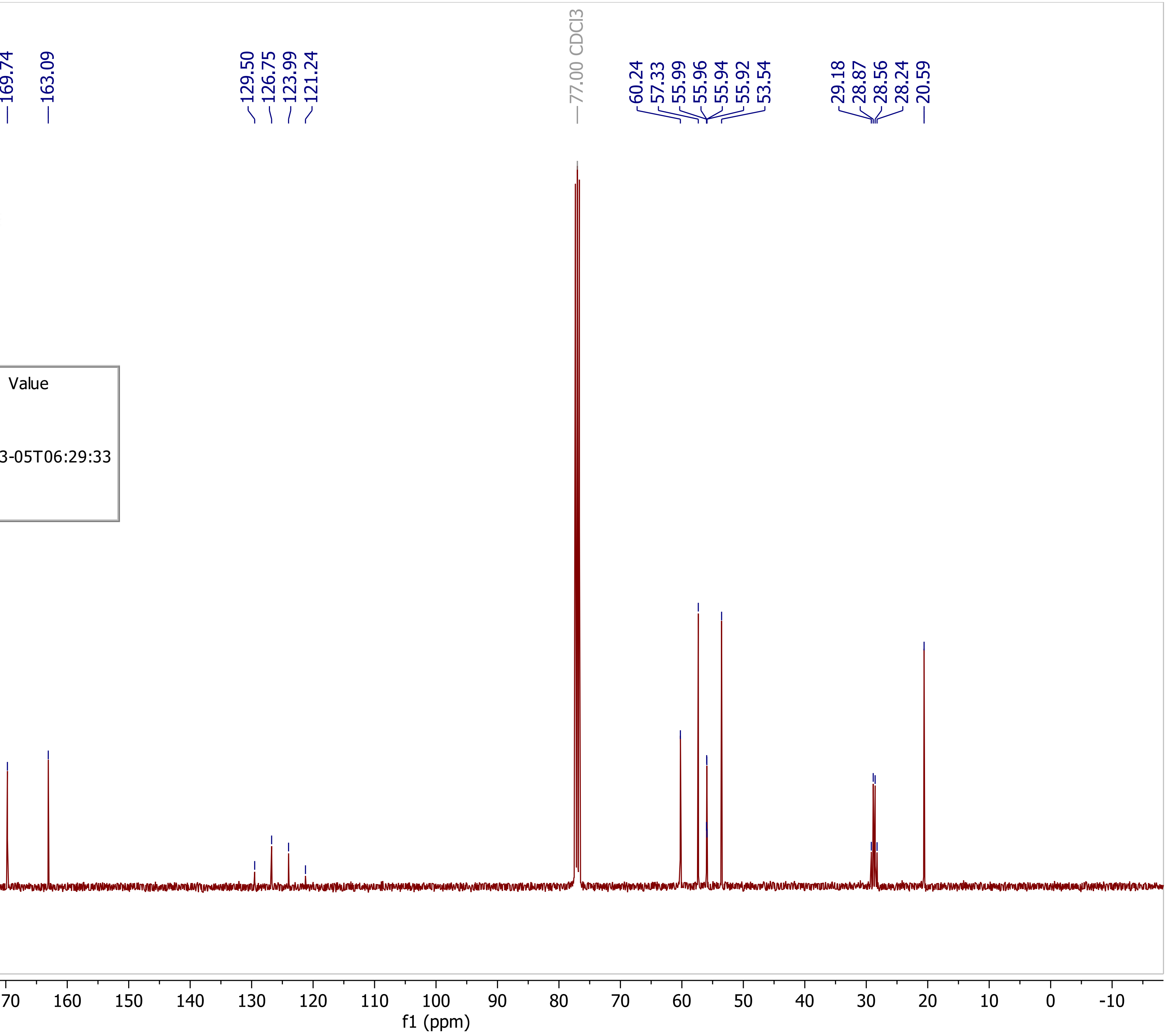

SIN-05-98-3-TT-24-C13.3.fid — SIN-05-98-3-TT-24-C

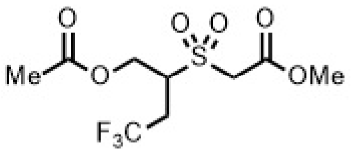

8

Parameter	Value
Solvent	CDCl3
Temperature	298.0
Acquisition Date	2024-03-28T11:55:02
Spectrometer Frequency	376.46
Nucleus	19F

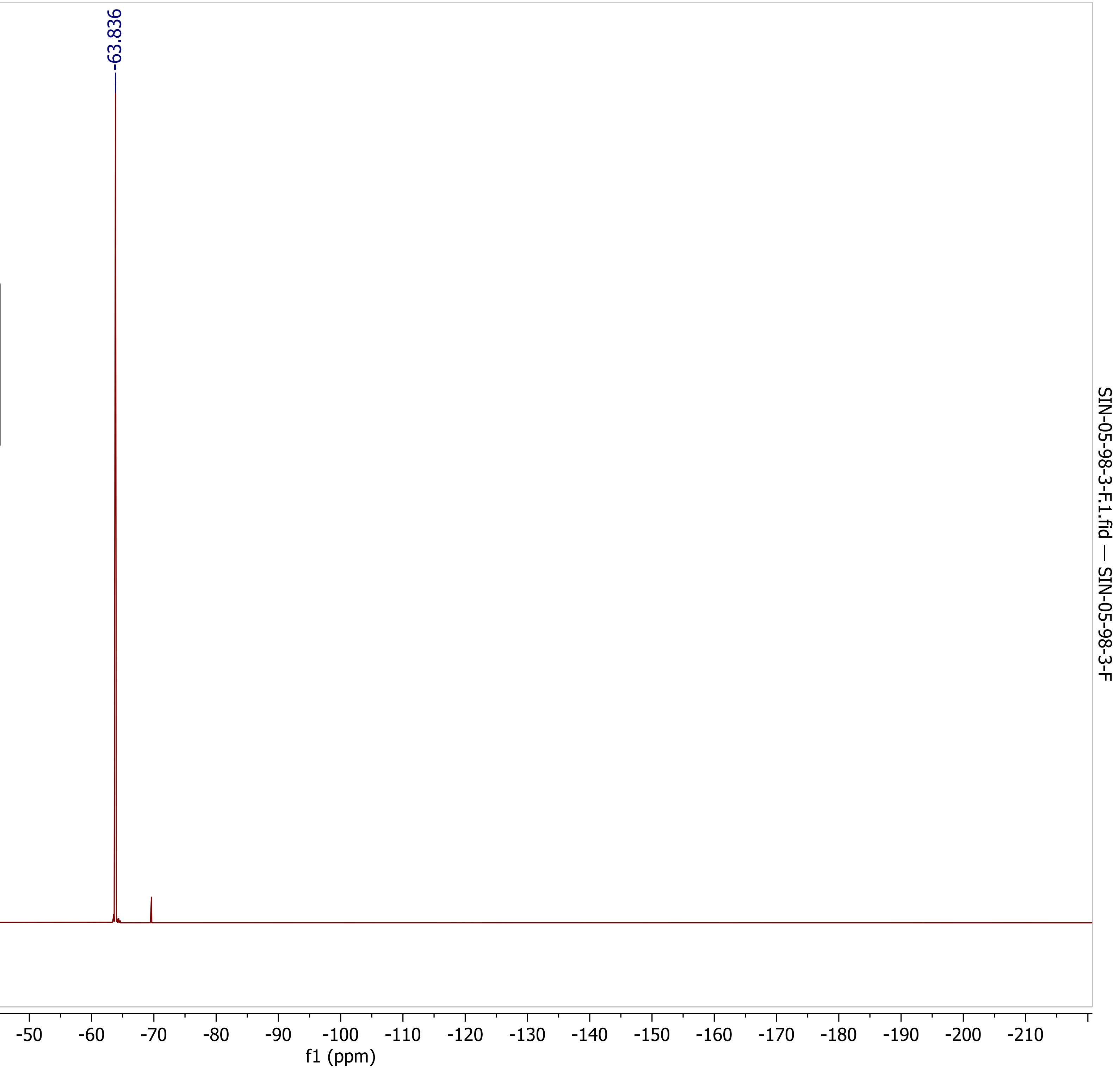

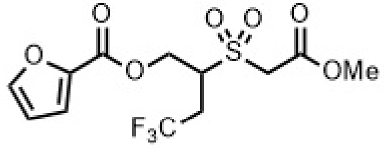

9

Parameter	Value
Solvent	CDCl3
Temperature	298.0
Acquisition Date	2024-09-30T22:08:47
Spectrometer Frequency	400.13
Nucleus	1H

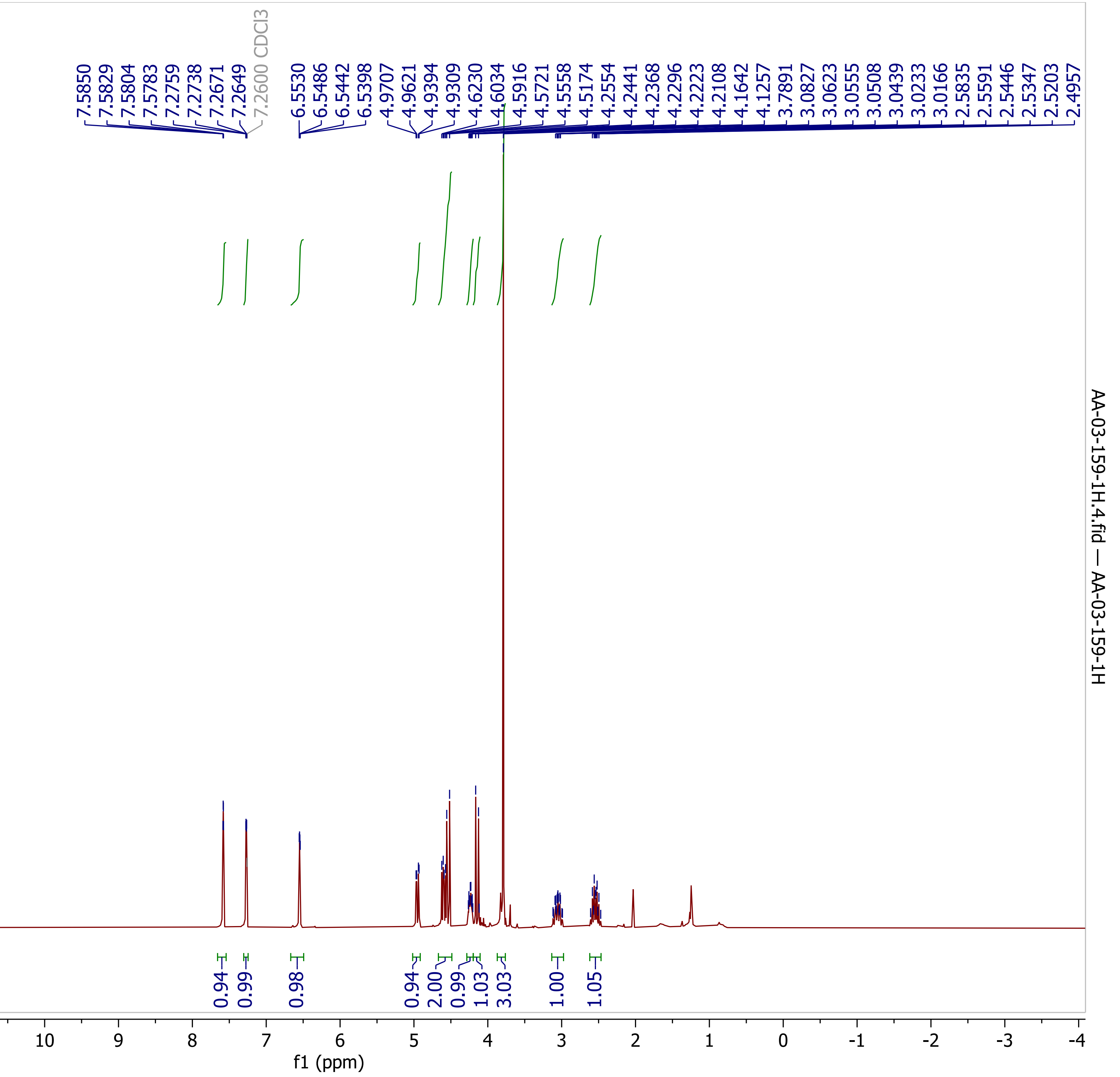

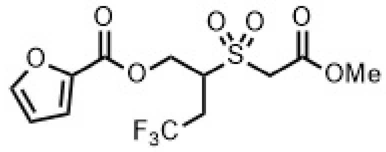

9

Parameter	Value
Solvent	CDCl3
Temperature	298.0
Acquisition Date	2024-09-30T23:09:34
Spectrometer Frequency	100.62
Nucleus	13C

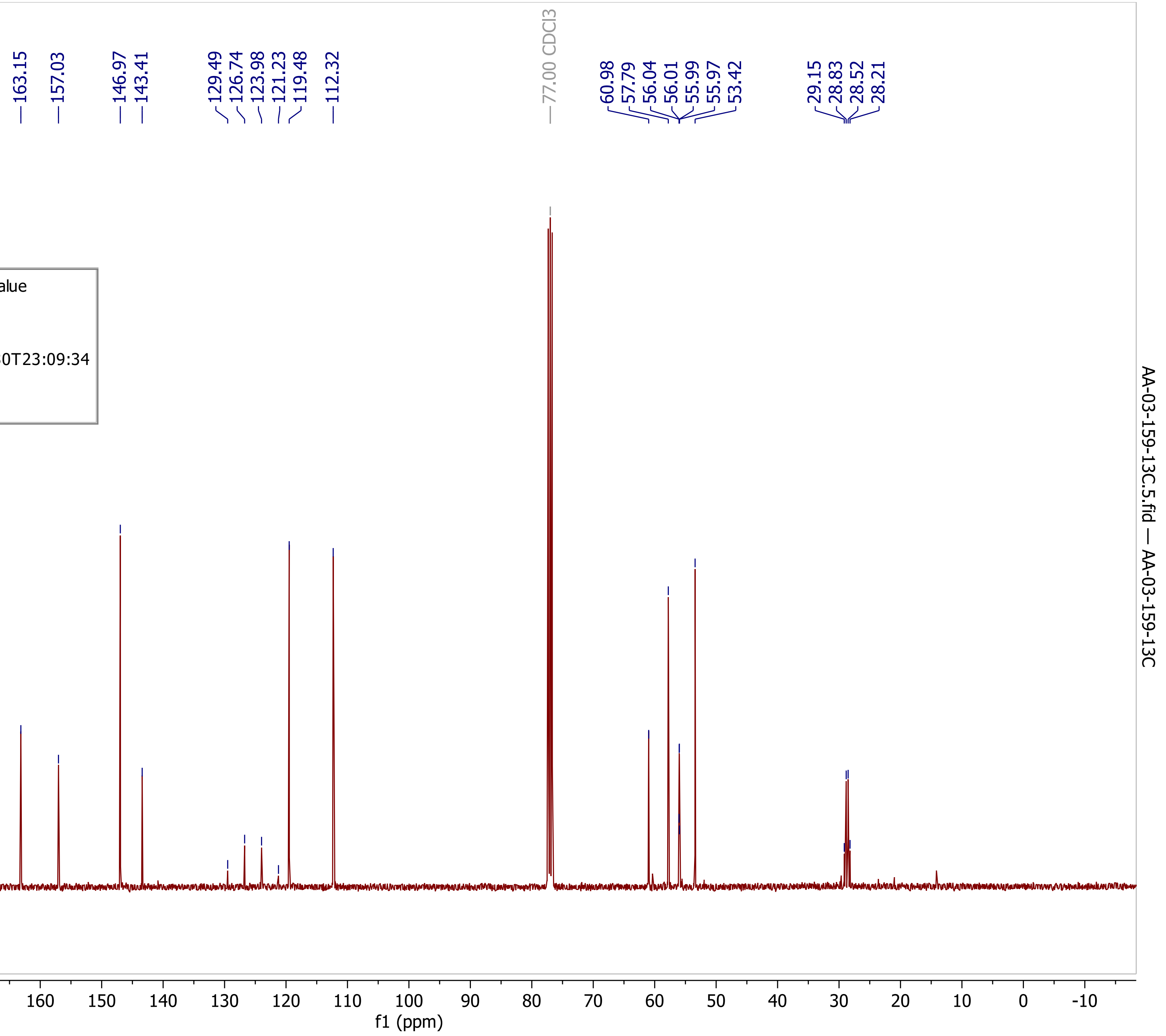

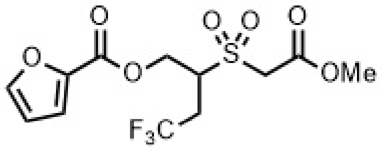

9

Parameter	Value
Solvent	CDCl3
Temperature	298.0
Acquisition Date	2024-09-30T23:14:01
Spectrometer Frequency	376.46
Nucleus	19F

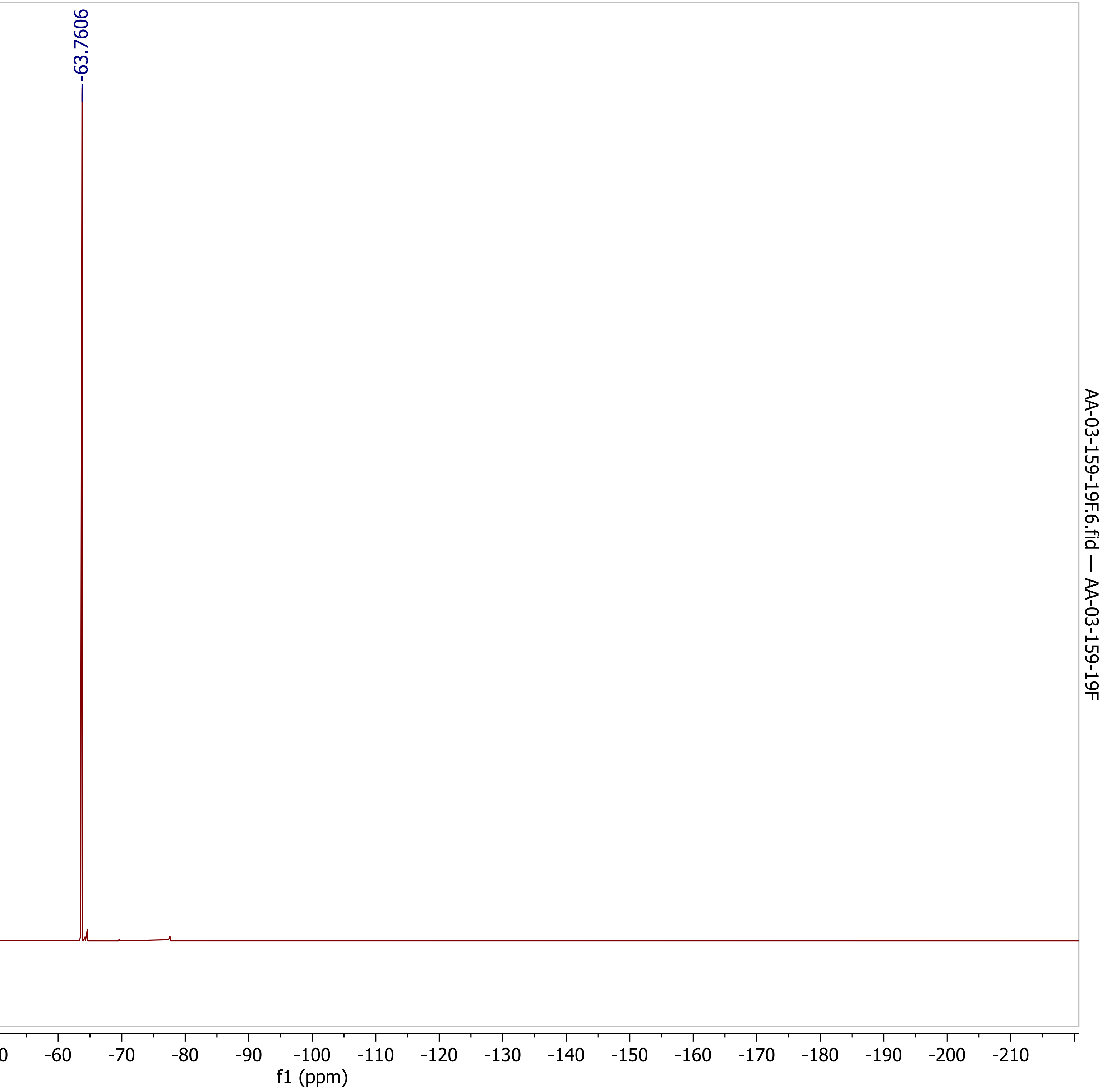

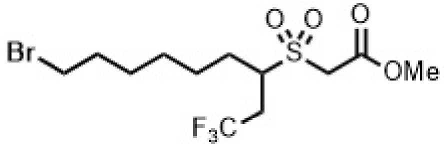

10

Parameter	Value
Solvent	CDCl3
Temperature	298.0
Acquisition Date	2024-05-13T15:24:33
Spectrometer Frequency	400.13
Nucleus	1H

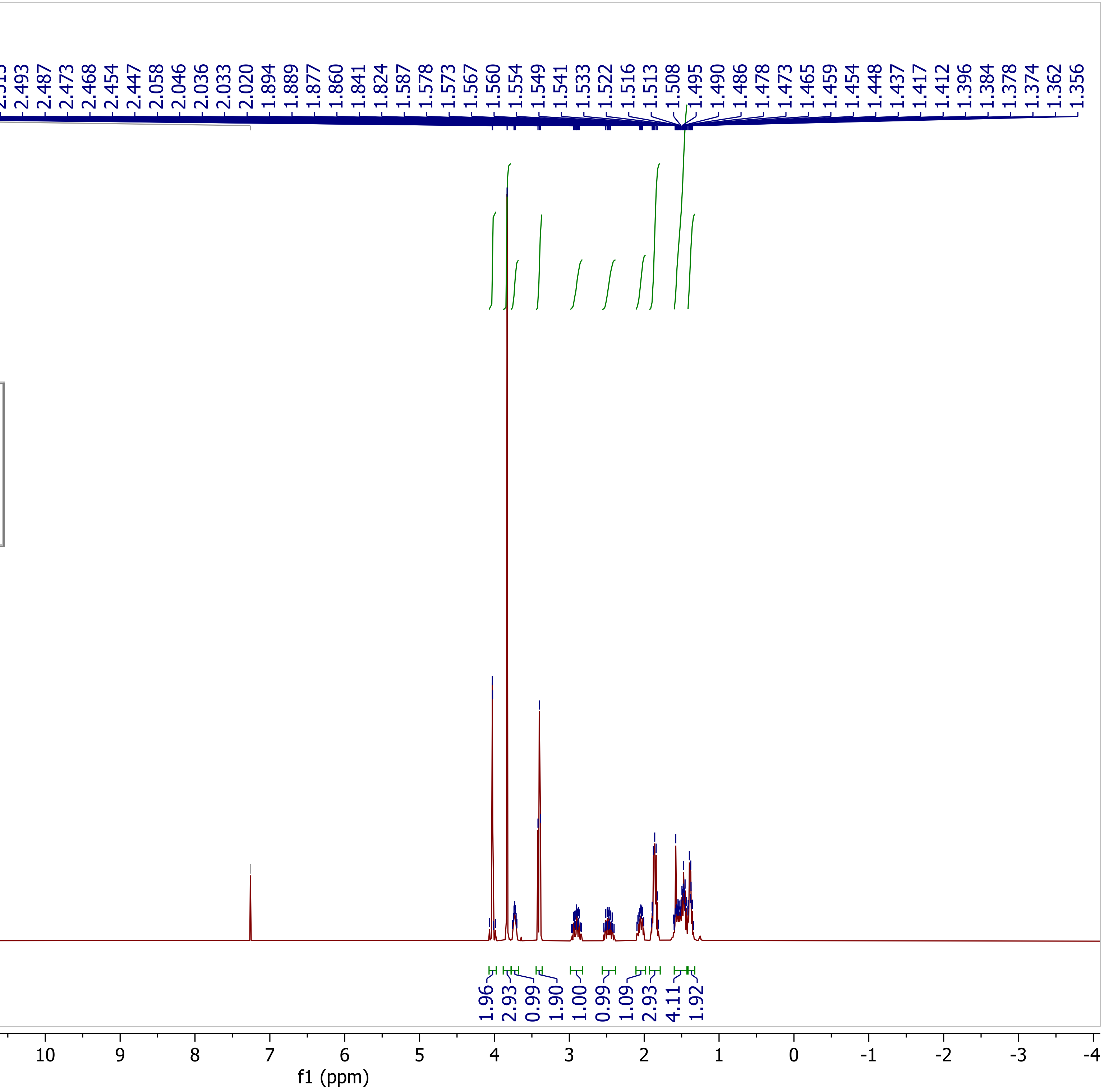

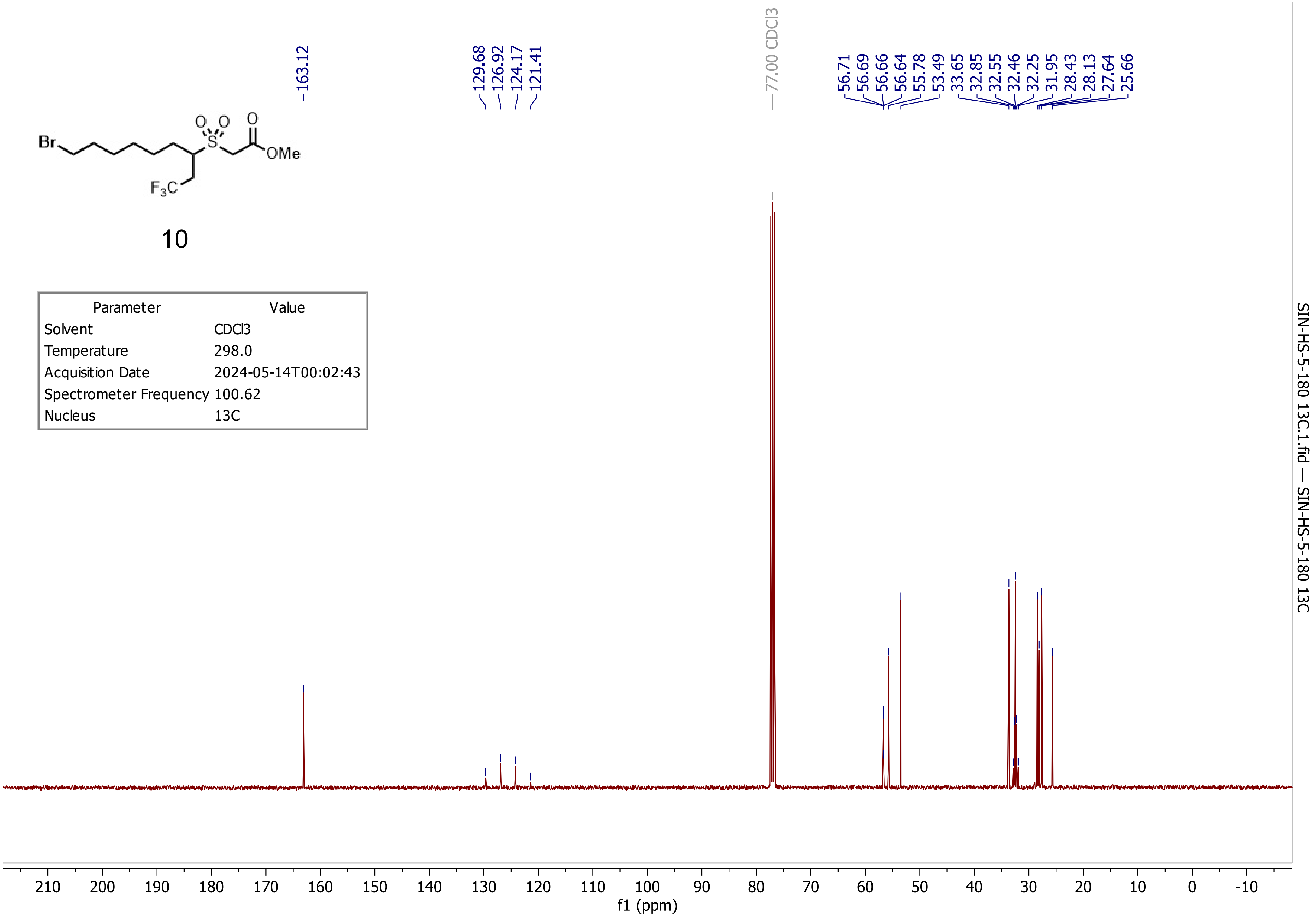

Parameter	Value
Solvent	CDCl3
Temperature	298.0
Acquisition Date	2024-05-14T00:02:43
Spectrometer Frequency	100.62
Nucleus	13C

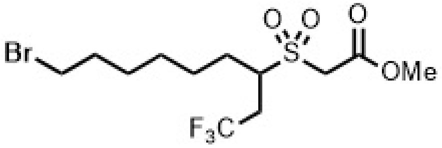

10

Parameter	Value
Solvent	CDCl3
Temperature	298.0
Acquisition Date	2024-07-10T12:35:28
Spectrometer Frequency	376.46
Nucleus	19F

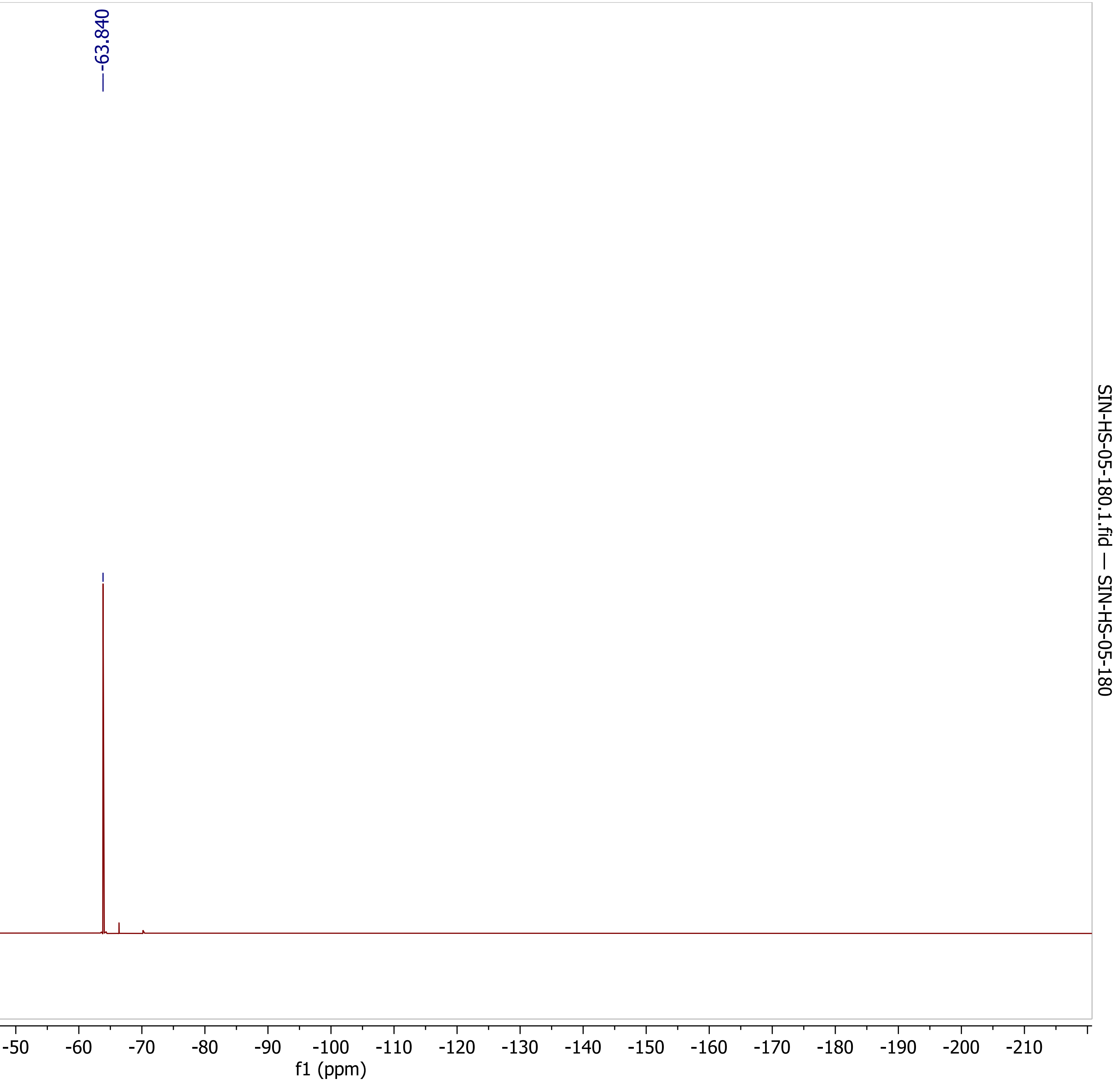

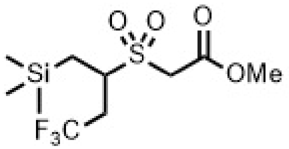

11

Parameter	Value
Solvent	CDCl3
Temperature	298.0
Acquisition Date	2024-03-08T10:49:06
Spectrometer Frequency	400.13
Nucleus	1H

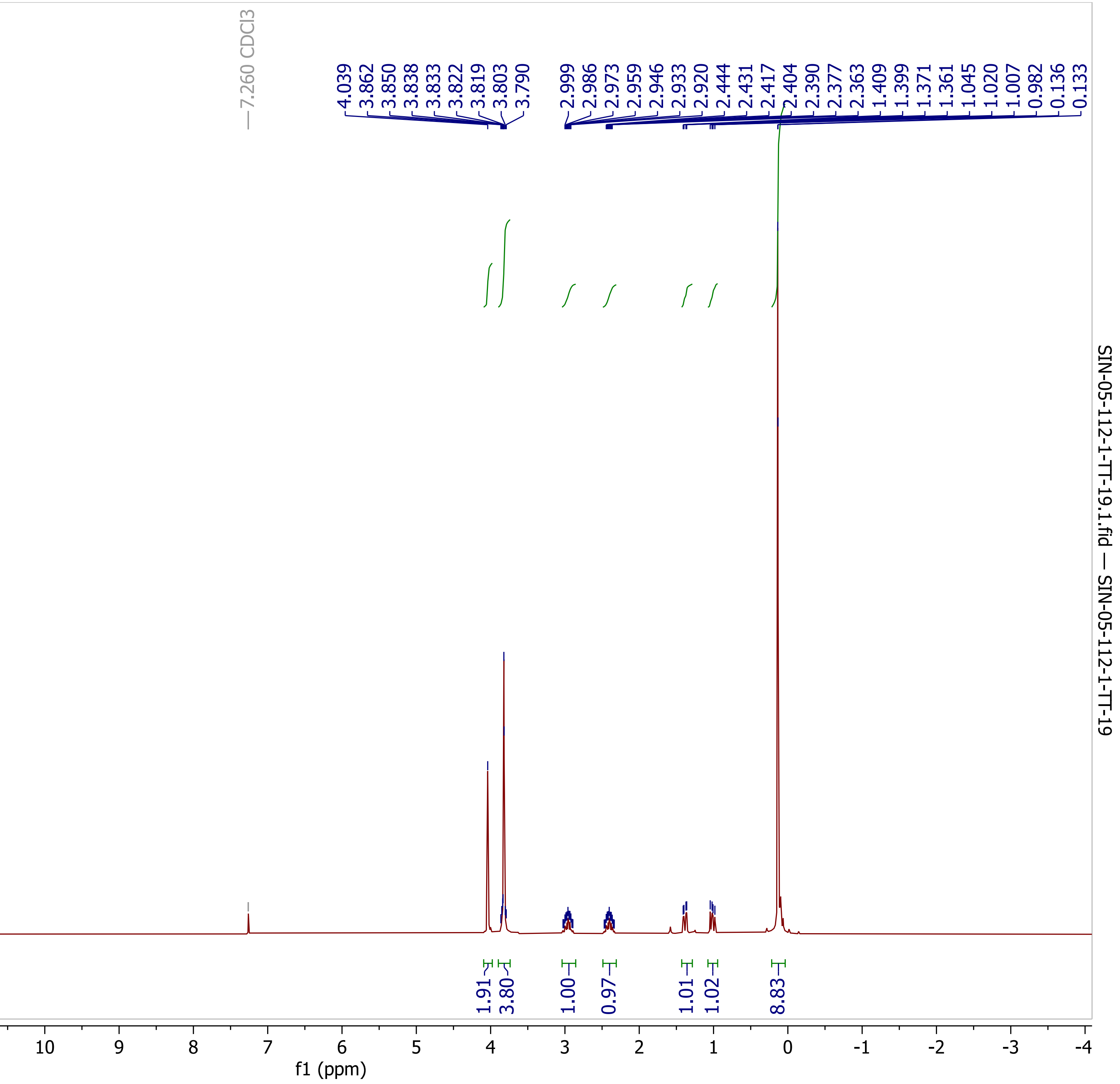

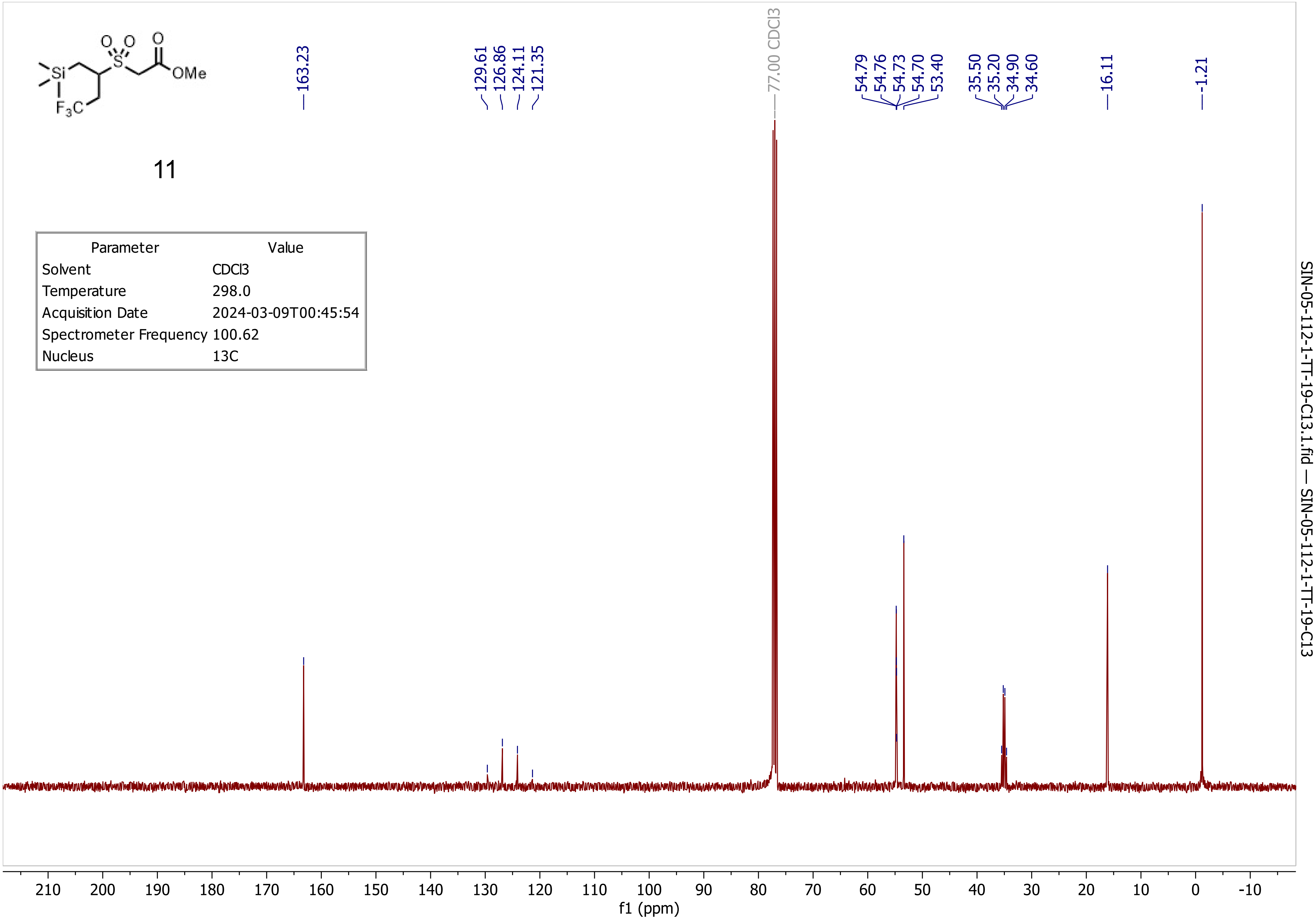

OMe
Si
F3C
11
163.23
129.61
126.86
124.11
121.35
77.00 CDCl3
54.79
54.76
54.73
54.70
53.40
35.50
35.20
34.90
34.60
16.11
-1.21
Parameter Value
Solvent CDCl3
Temperature 298.0
Acquisition Date 2024-03-09T00:45:54
Spectrometer Frequency 100.62
Nucleus 13C
210 200 190 180 170 160 150 140 130 120 110 100 90 80 70 60 50 40 30 20 10 0 -10
f1 (ppm)
SIN-05-112-1-TT-19-C13.1.fid — SIN-05-112-1-TT-19-C13

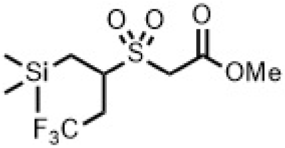

11

Parameter	Value
Solvent	CDCl3
Temperature	298.0
Acquisition Date	2024-07-16T18:46:00
Spectrometer Frequency	376.46
Nucleus	19F

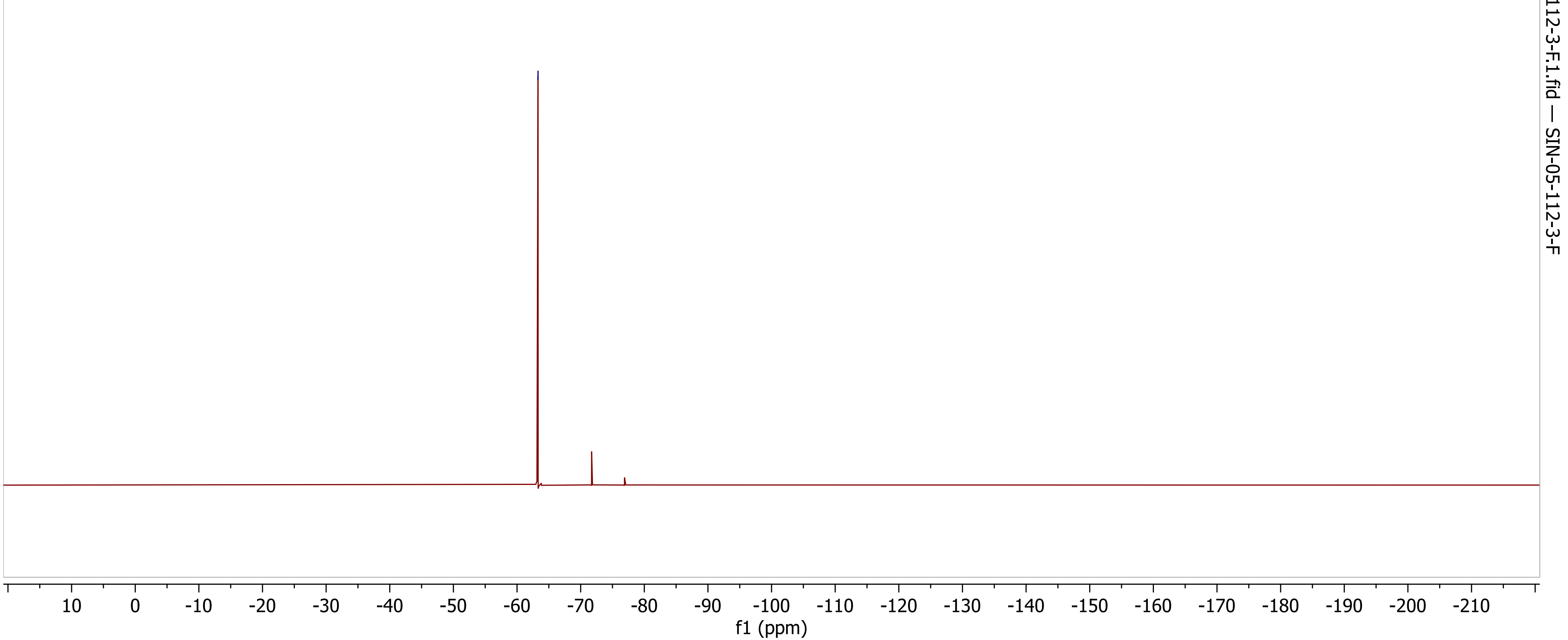

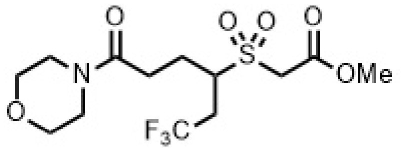

12

Parameter	Value
Solvent	CDCl3
Temperature	298.0
Acquisition Date	2024-04-12T12:25:47
Spectrometer Frequency	400.13
Nucleus	1H

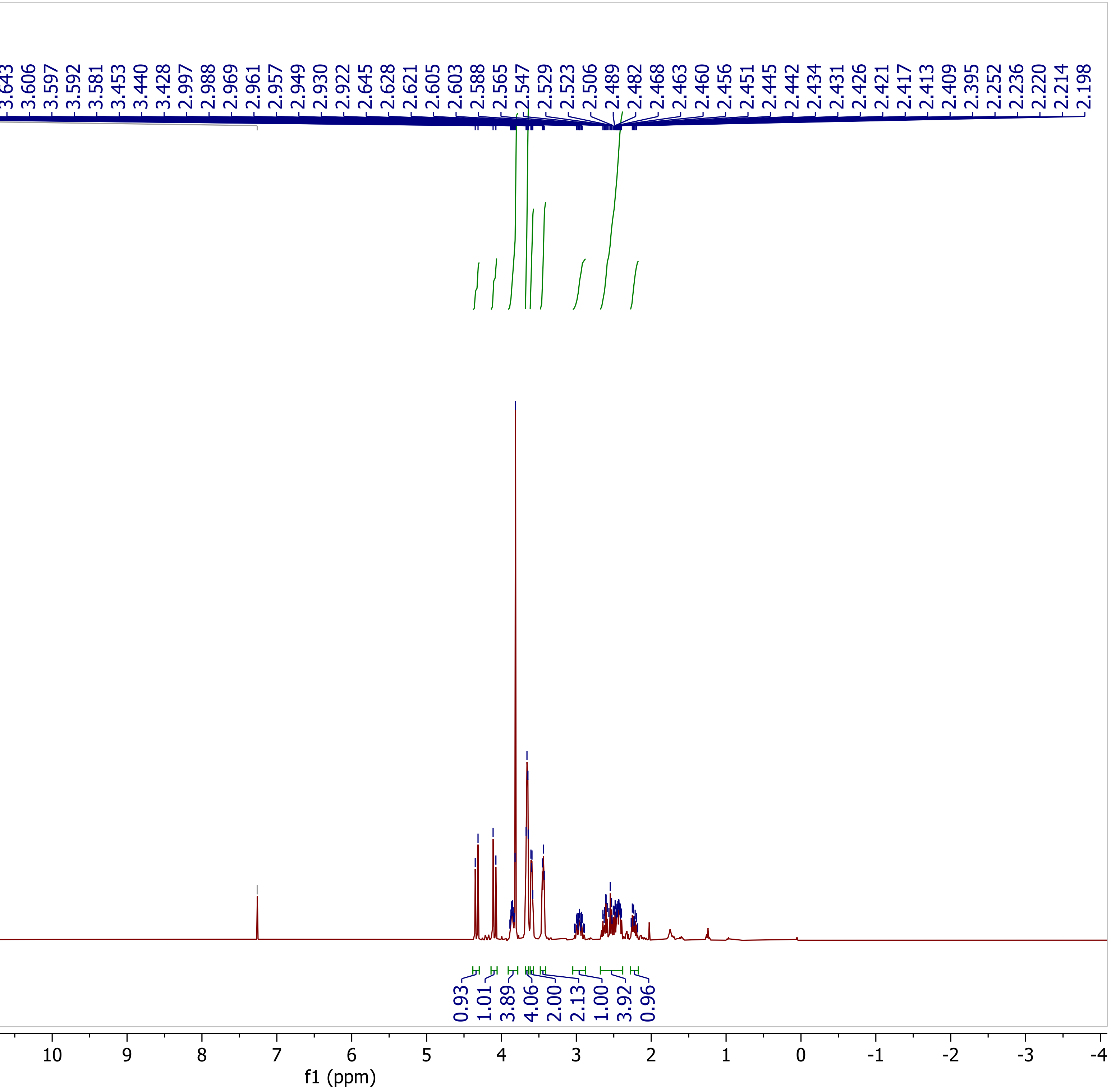

SIN-HS-5-124PURE.1.fid — SIN-HS-5-124PURE

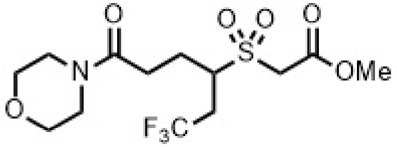

12

Parameter	Value
Solvent	CDCl3
Temperature	298.0
Acquisition Date	2024-04-13T00:25:38
Spectrometer Frequency	100.62
Nucleus	13C

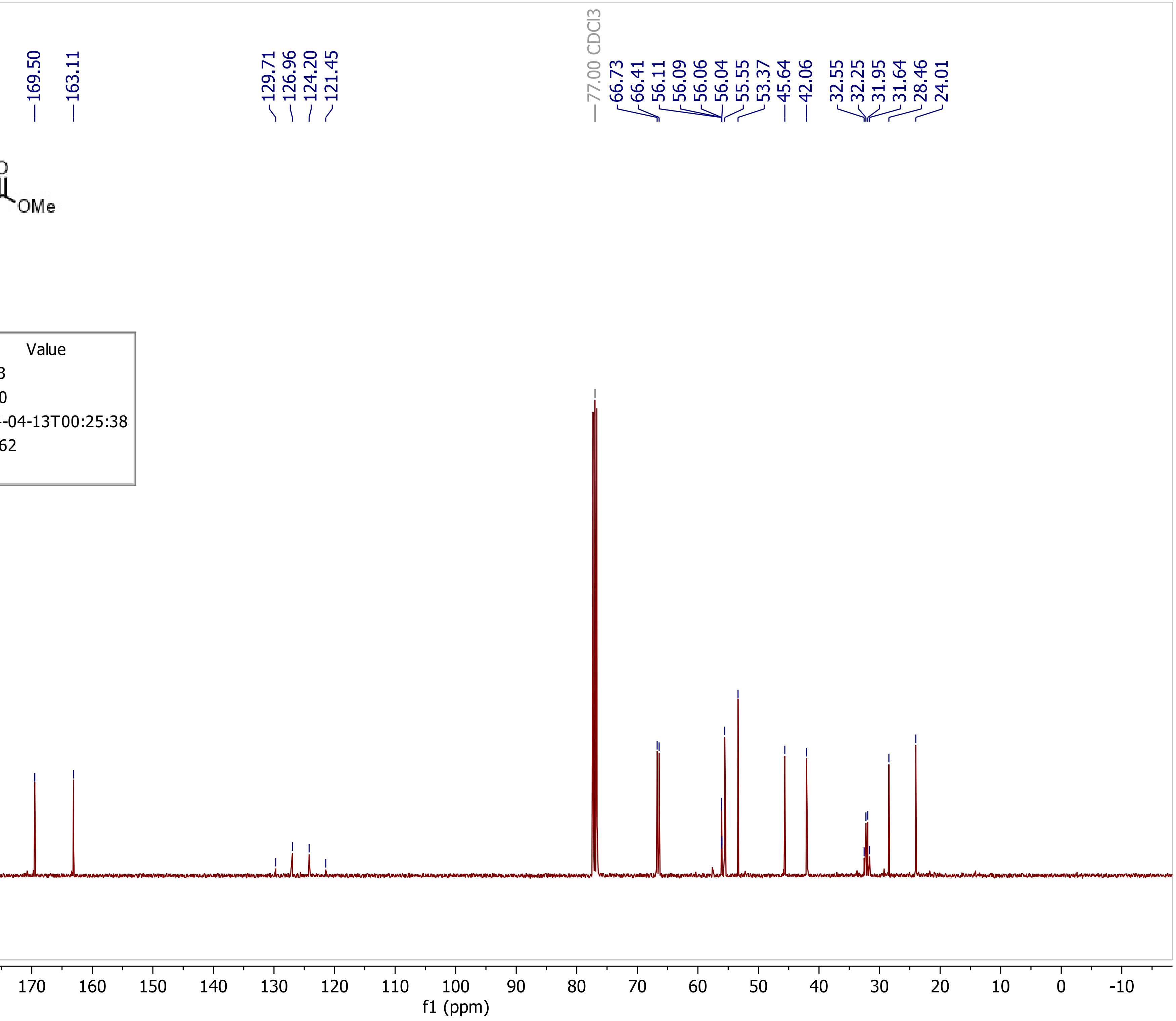

SIN-HS-05-124 PURE 13C.1.fid — SIN-HS-05-124 PURE

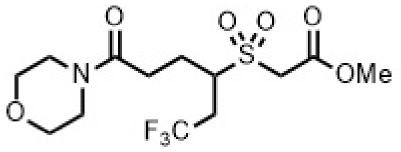

12

Parameter	Value
Solvent	CDCl3
Temperature	298.0
Acquisition Date	2024-08-06T18:49:47
Spectrometer Frequency	376.46
Nucleus	19F

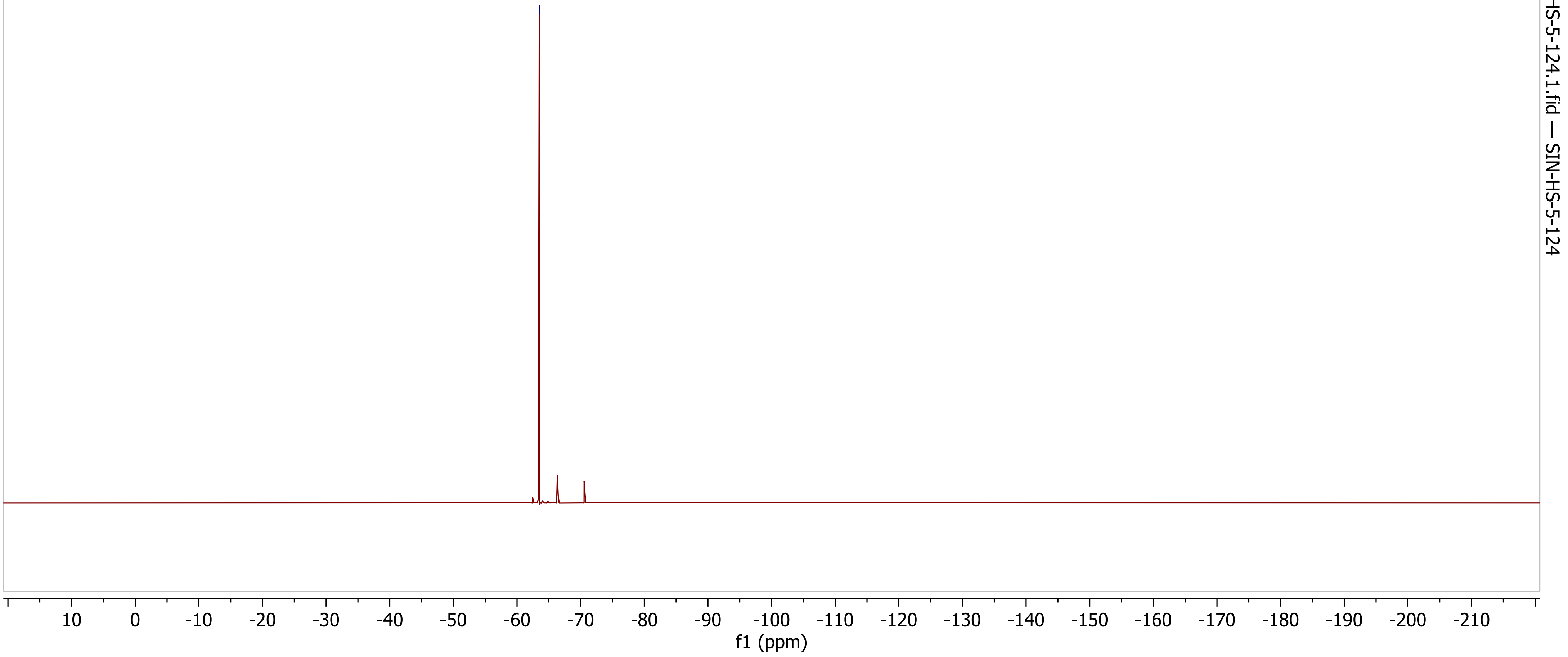

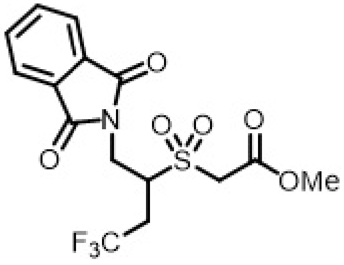

13

Parameter	Value
Solvent	CDCl3
Temperature	298.0
Acquisition Date	2024-07-17T12:21:10
Spectrometer Frequency	400.13
Nucleus	1H

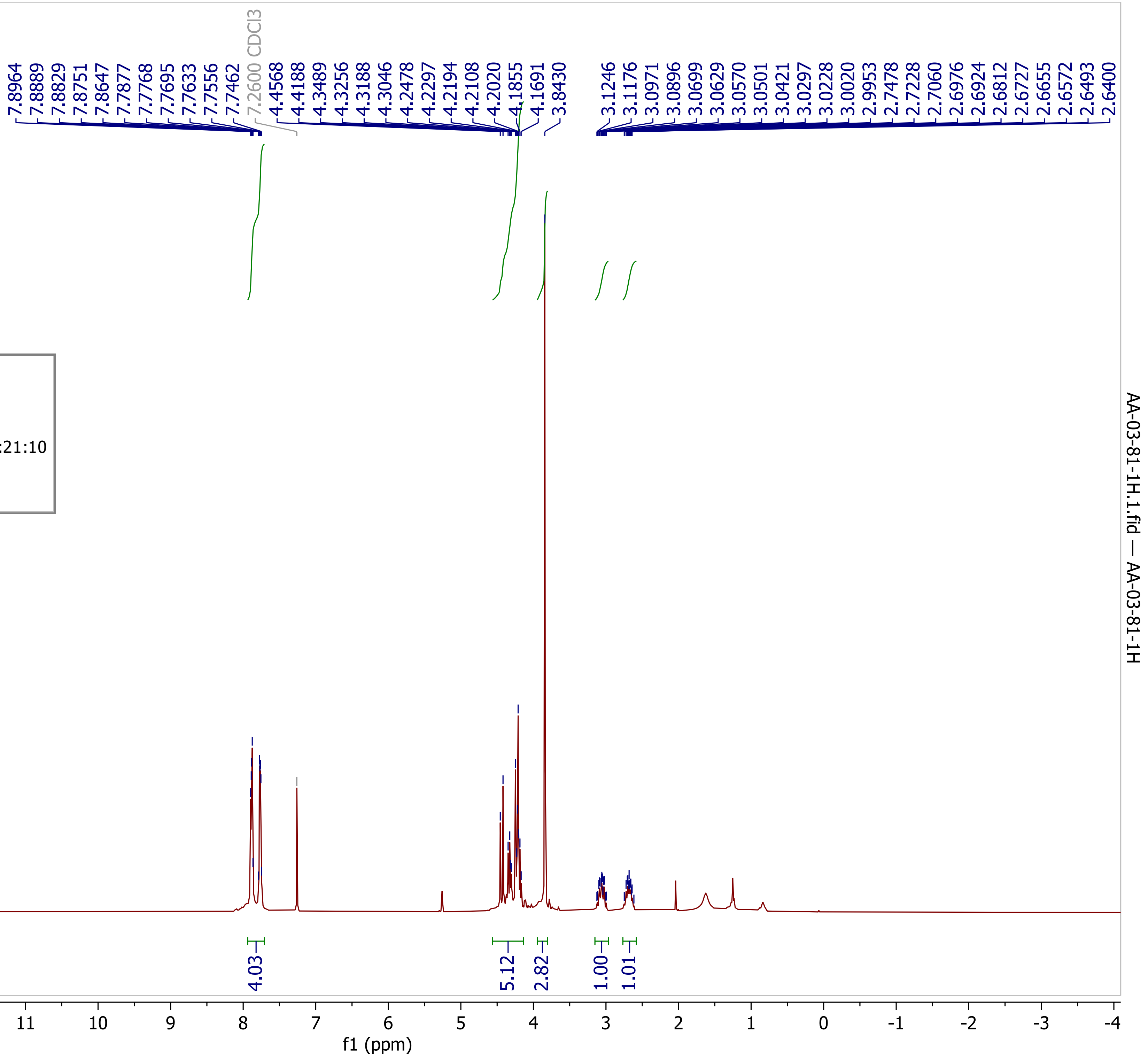

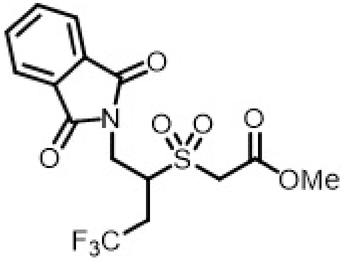

13

Parameter	Value
Solvent	CDCl3
Temperature	298.0
Acquisition Date	2024-09-18T23:15:07
Spectrometer Frequency	100.62
Nucleus	13C

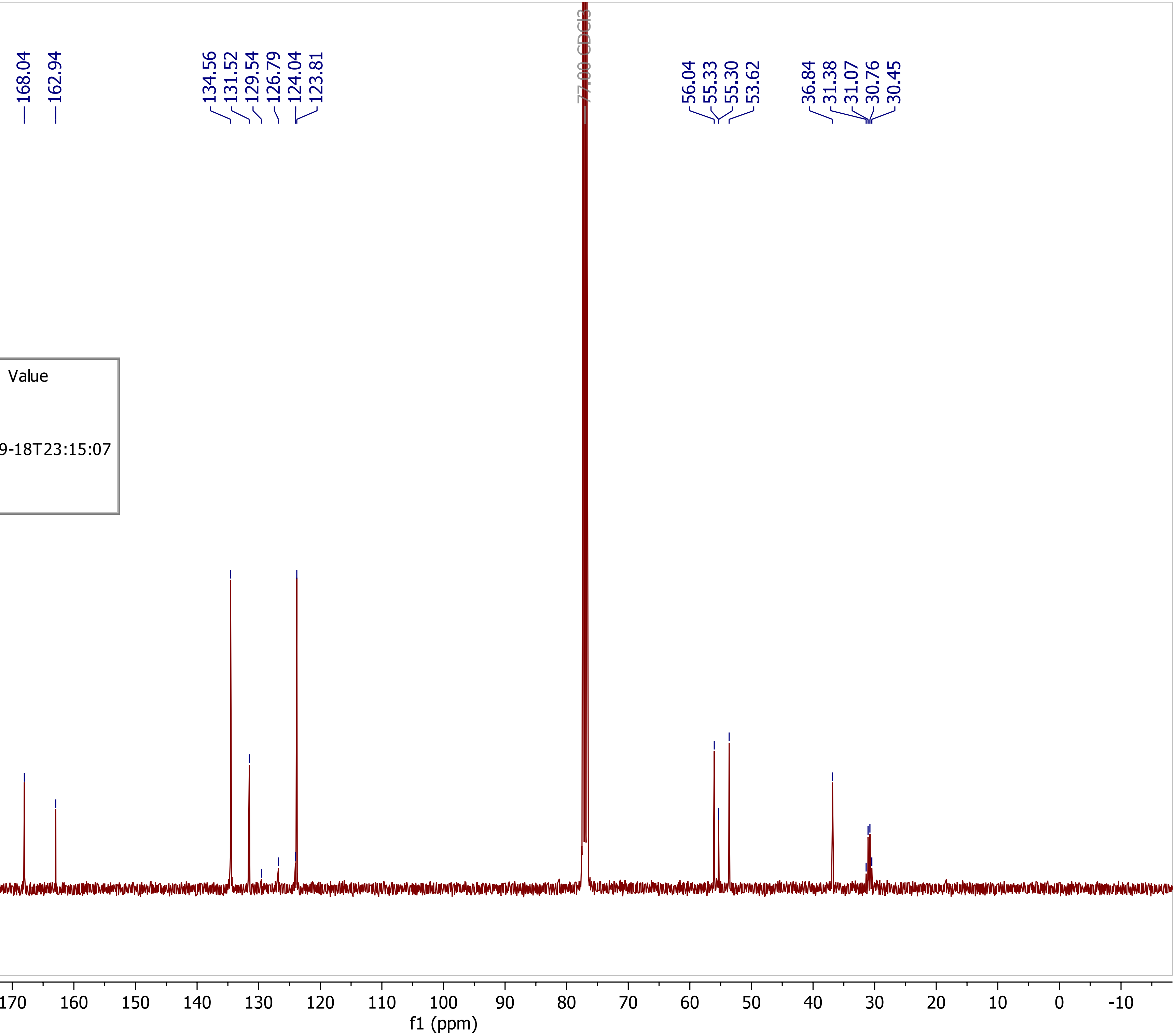

AA-03-81-13C-NMR.1.fid — AA-03-81-13C-NMR

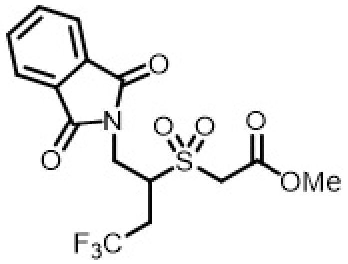

13

Parameter	Value
Solvent	CDCl3
Temperature	298.0
Acquisition Date	2024-07-17T12:25:35
Spectrometer Frequency	376.46
Nucleus	19F

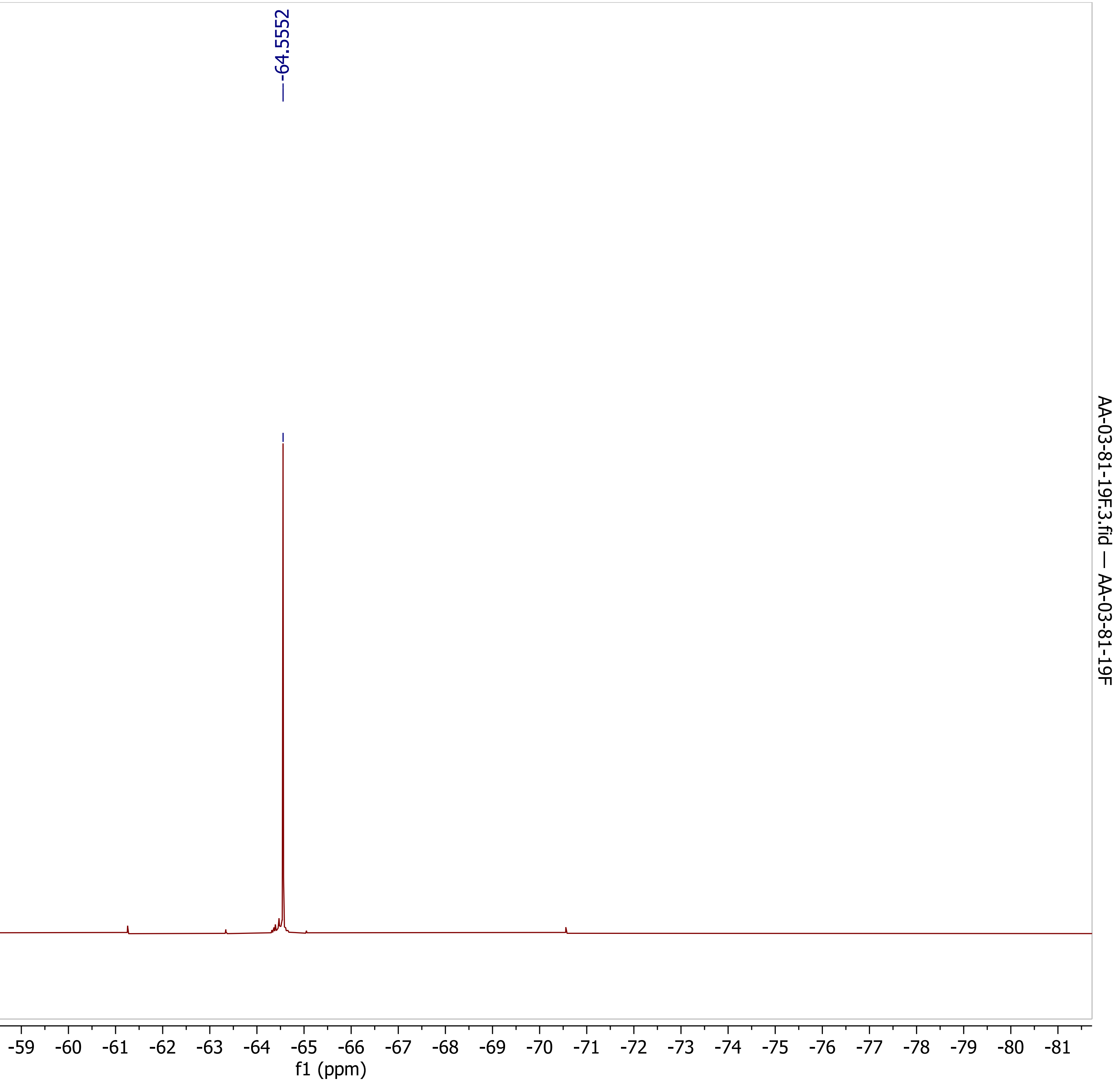

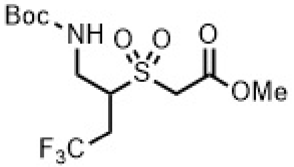

14

Parameter	Value
Solvent	CDCl3
Temperature	298.0
Acquisition Date	2024-09-19T00:37:49
Spectrometer Frequency	400.13
Nucleus	1H

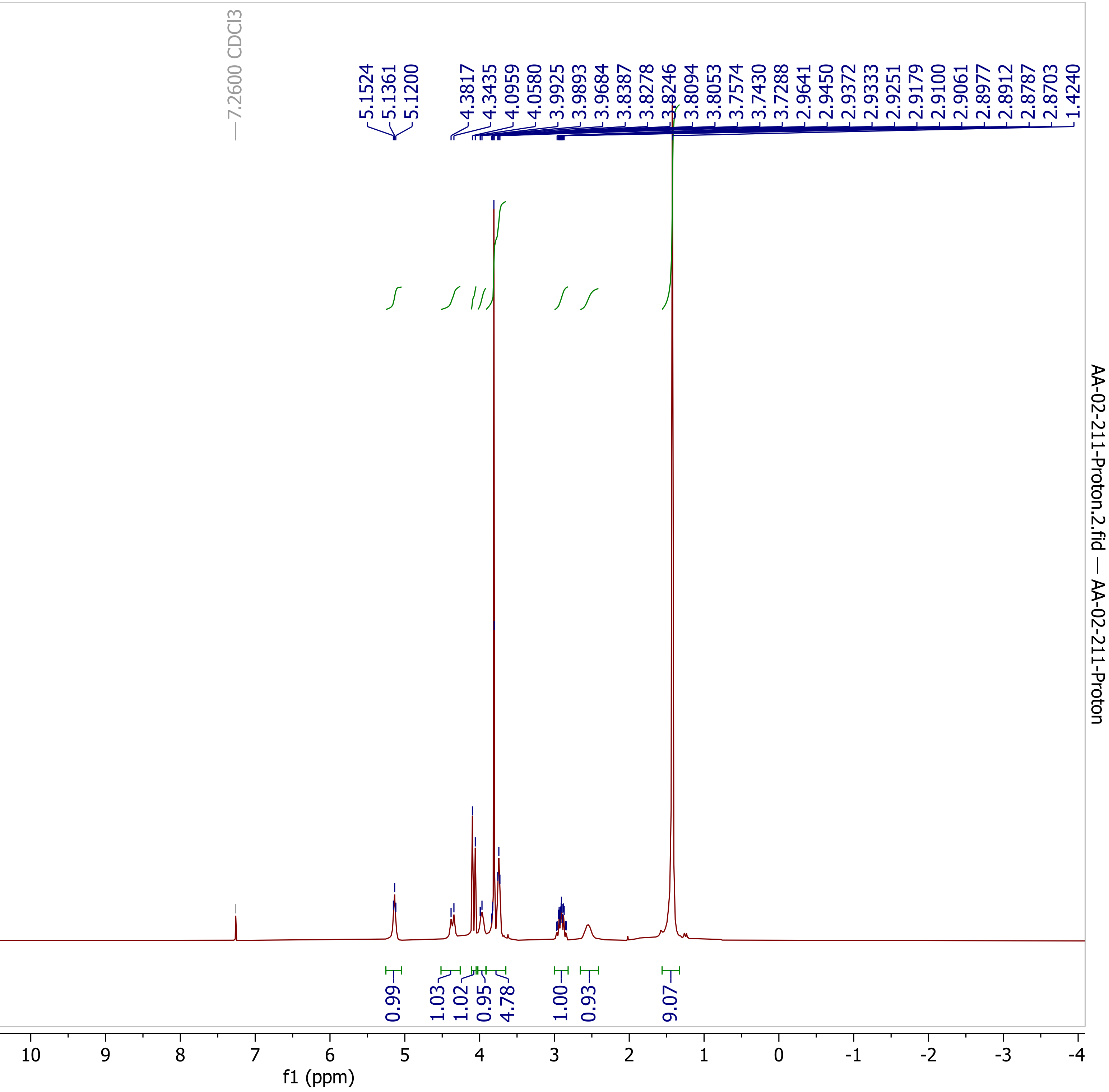

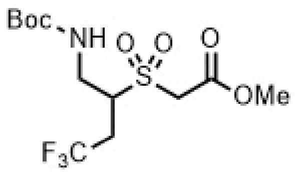

14

Parameter	Value
Solvent	CDCl3
Temperature	298.0
Acquisition Date	2024-09-19T01:38:05
Spectrometer Frequency	100.62
Nucleus	13C

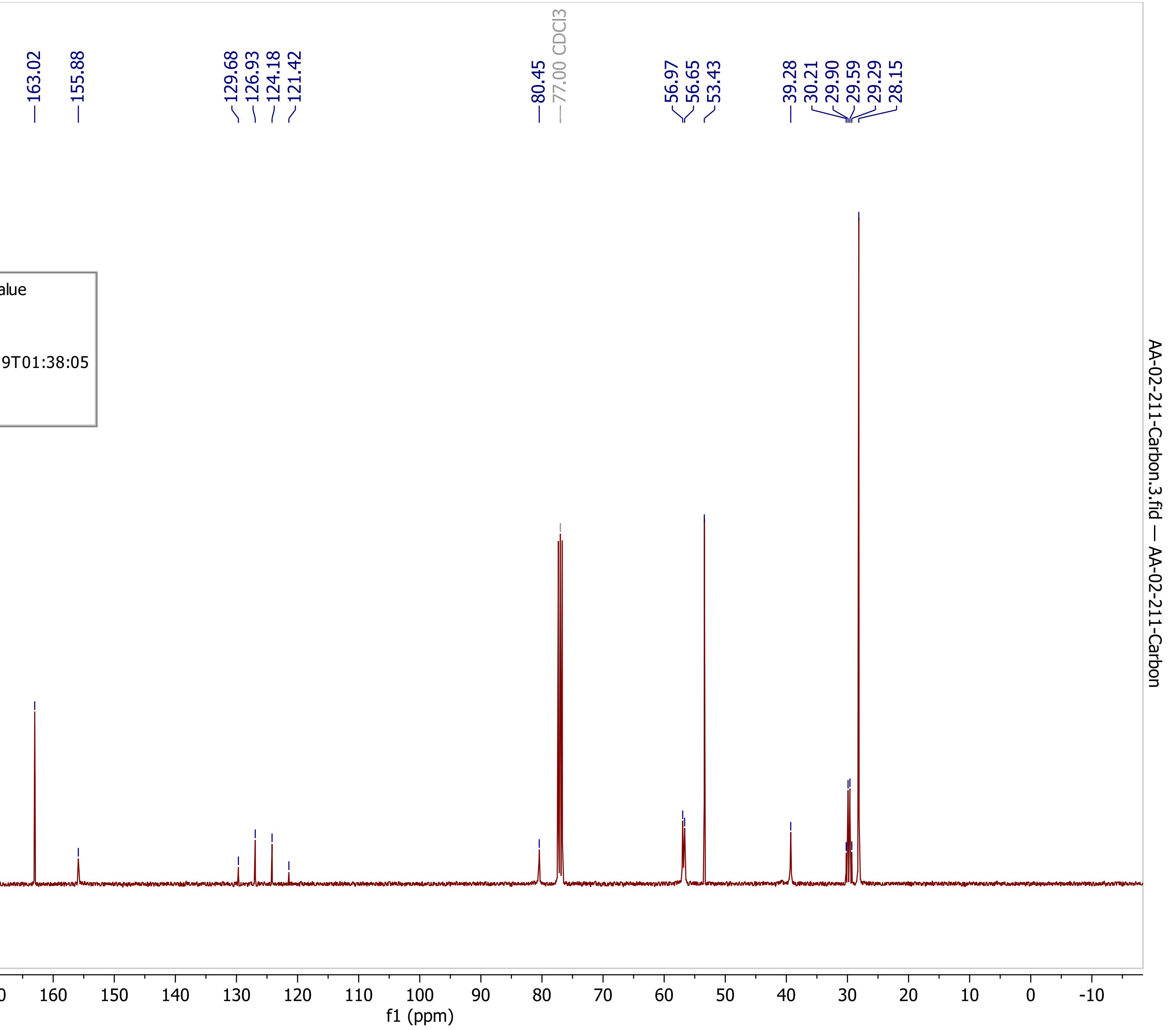

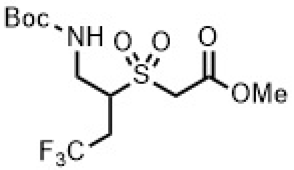

14

Parameter	Value
Solvent	CDCl3
Temperature	298.0
Acquisition Date	2024-03-20T22:50:08
Spectrometer Frequency	376.46
Nucleus	19F

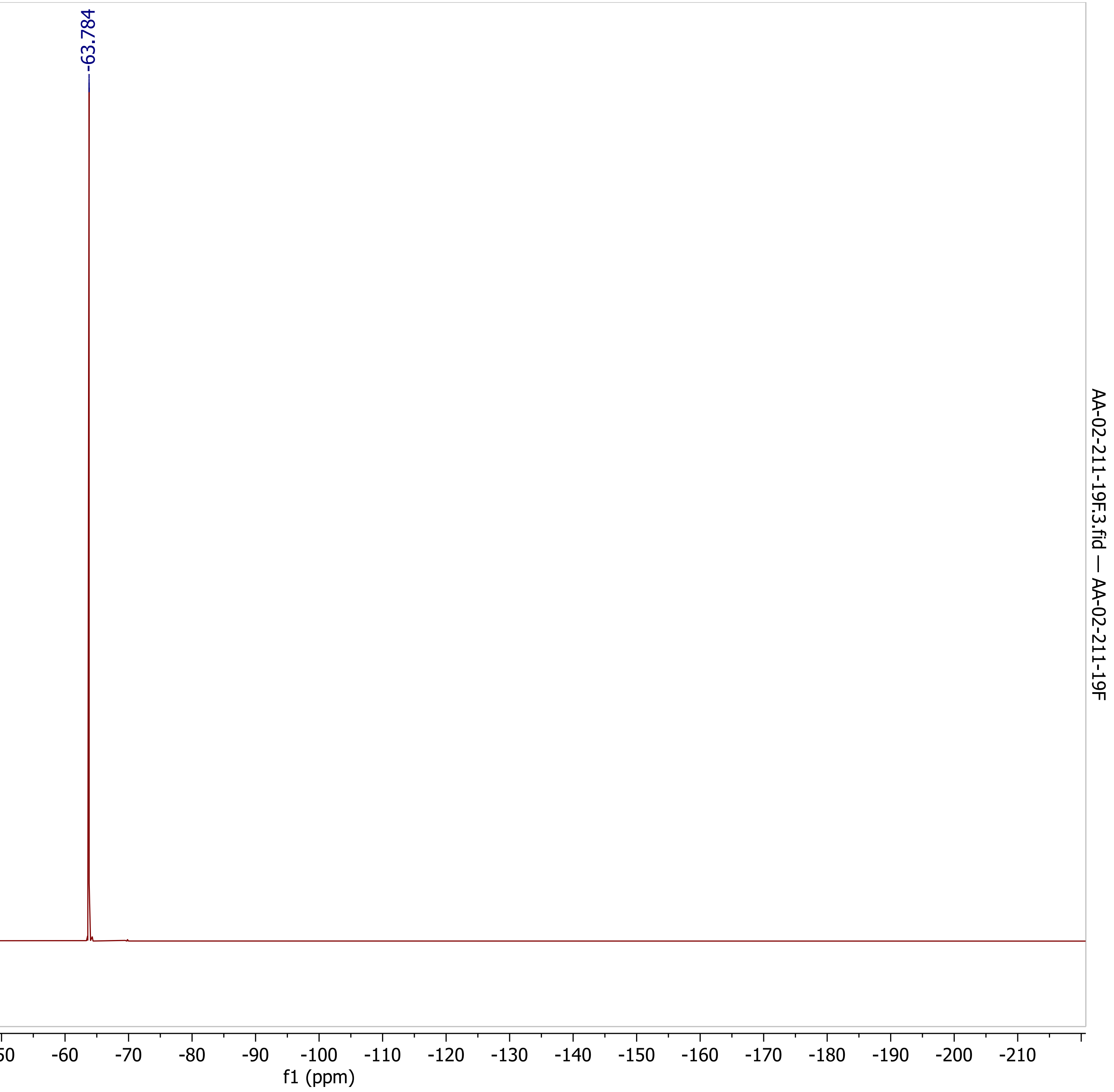

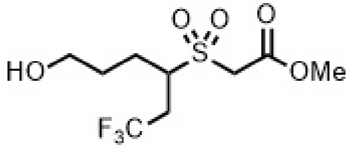

15

Parameter	Value
Solvent	CDCl3
Temperature	298.0
Acquisition Date	2024-03-07T19:13:26
Spectrometer Frequency	400.13
Nucleus	1H

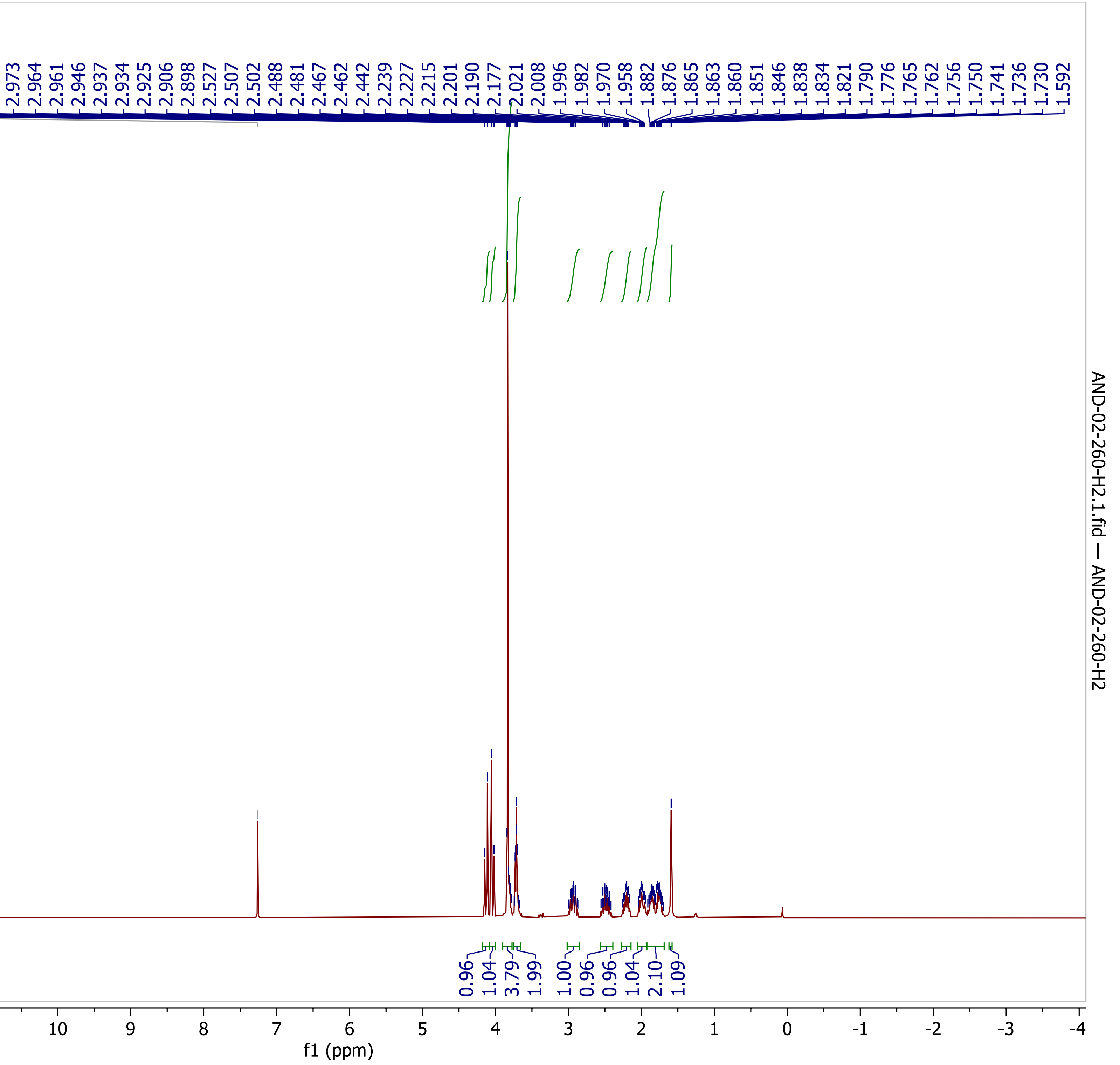

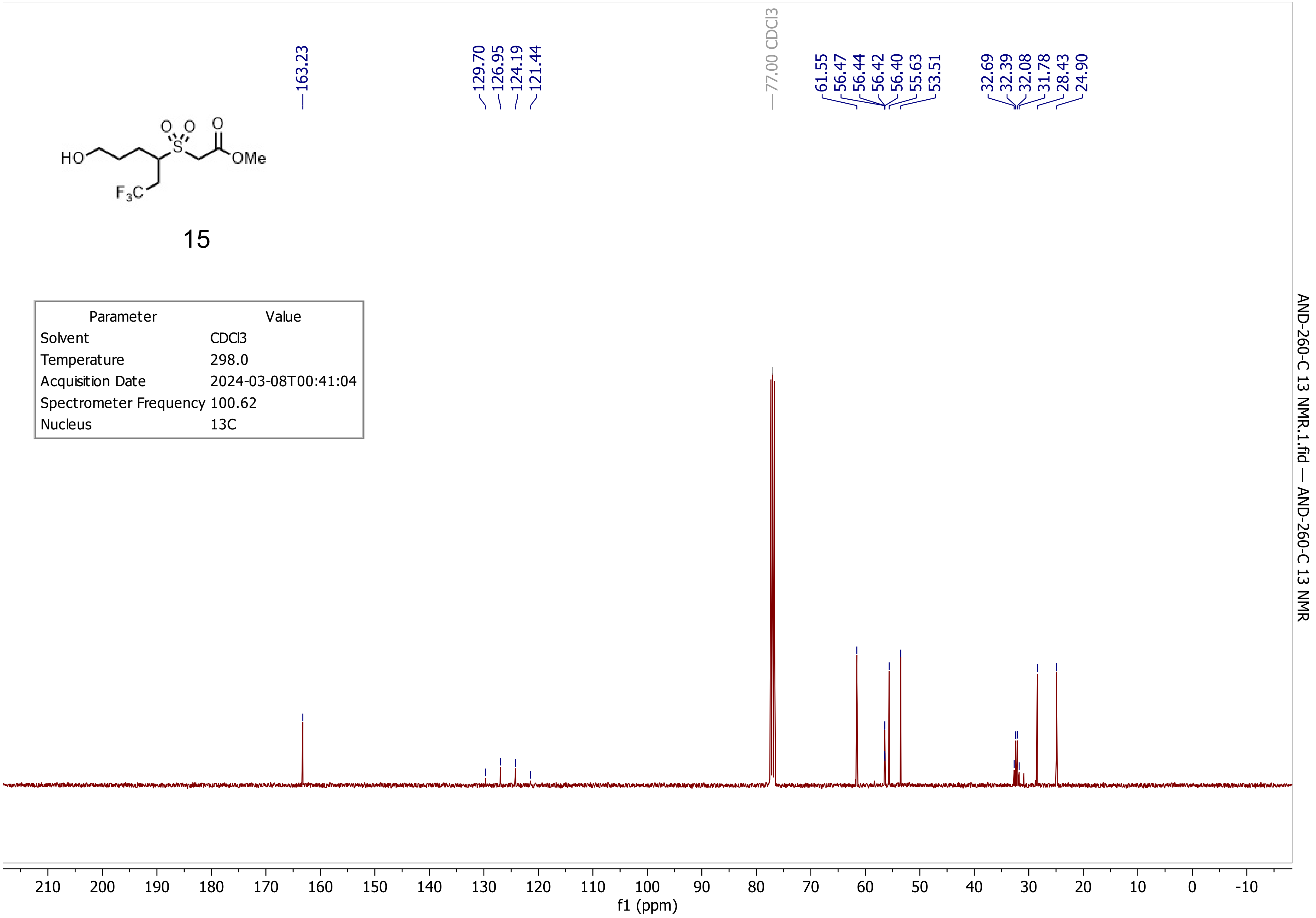

HO
O O
S
O
OMe
F3C
15
Parameter
Value
Solvent
CDCl3
Temperature
298.0
Acquisition Date
2024-03-08T00:41:04
Spectrometer Frequency
100.62
Nucleus
13C
163.23
129.70
126.95
124.19
121.44
77.00 CDCl3
61.55
56.47
56.44
56.42
56.40
55.63
53.51
32.69
32.39
32.08
31.78
28.43
24.90
f1 (ppm)
AND-260-C 13 NMR.1.fid — AND-260-C 13 NMR

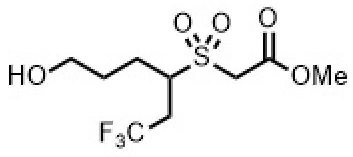

15

Parameter	Value
Solvent	CDCl3
Temperature	298.0
Acquisition Date	2024-03-08T00:45:18
Spectrometer Frequency	376.46
Nucleus	19F

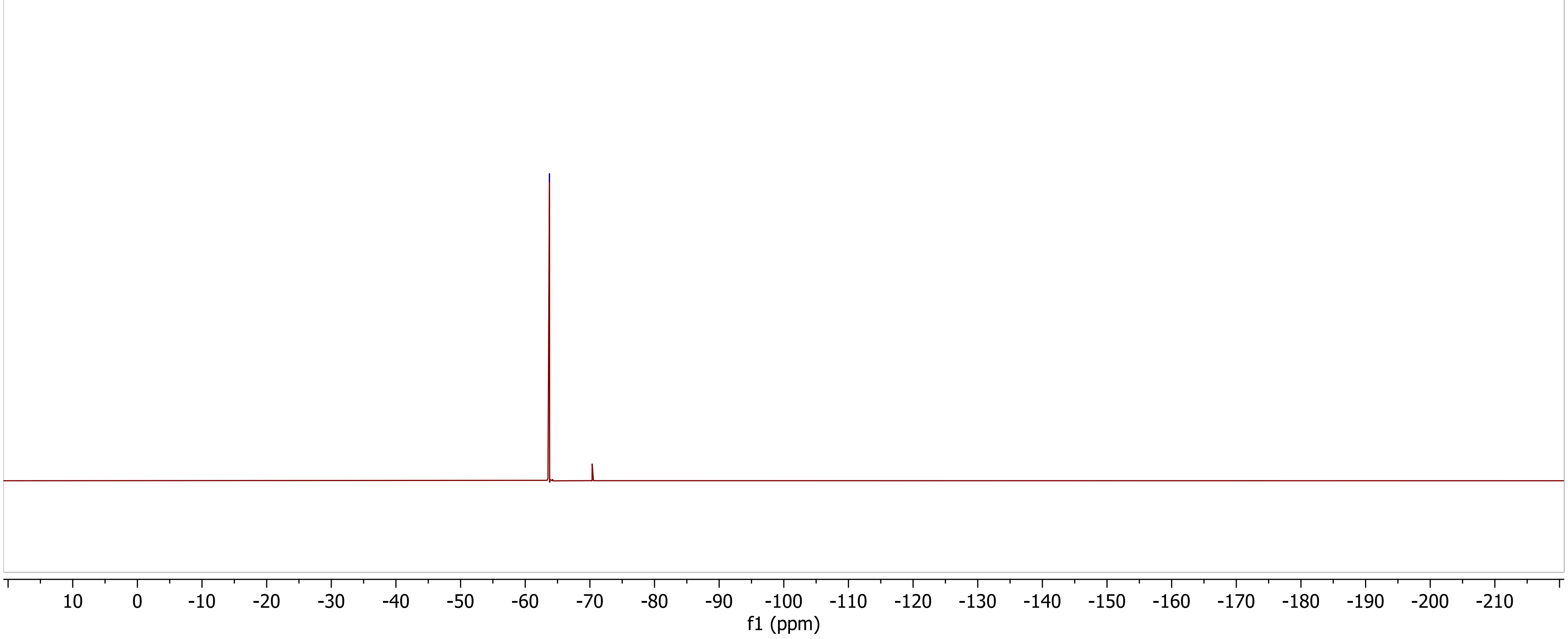

AND-260-F NMR.3.fid — AND-260-F NMR

16

Parameter	Value
Solvent	CDCl3
Temperature	298.0
Acquisition Date	2024-03-20T09:27:51
Spectrometer Frequency	400.13
Nucleus	1H

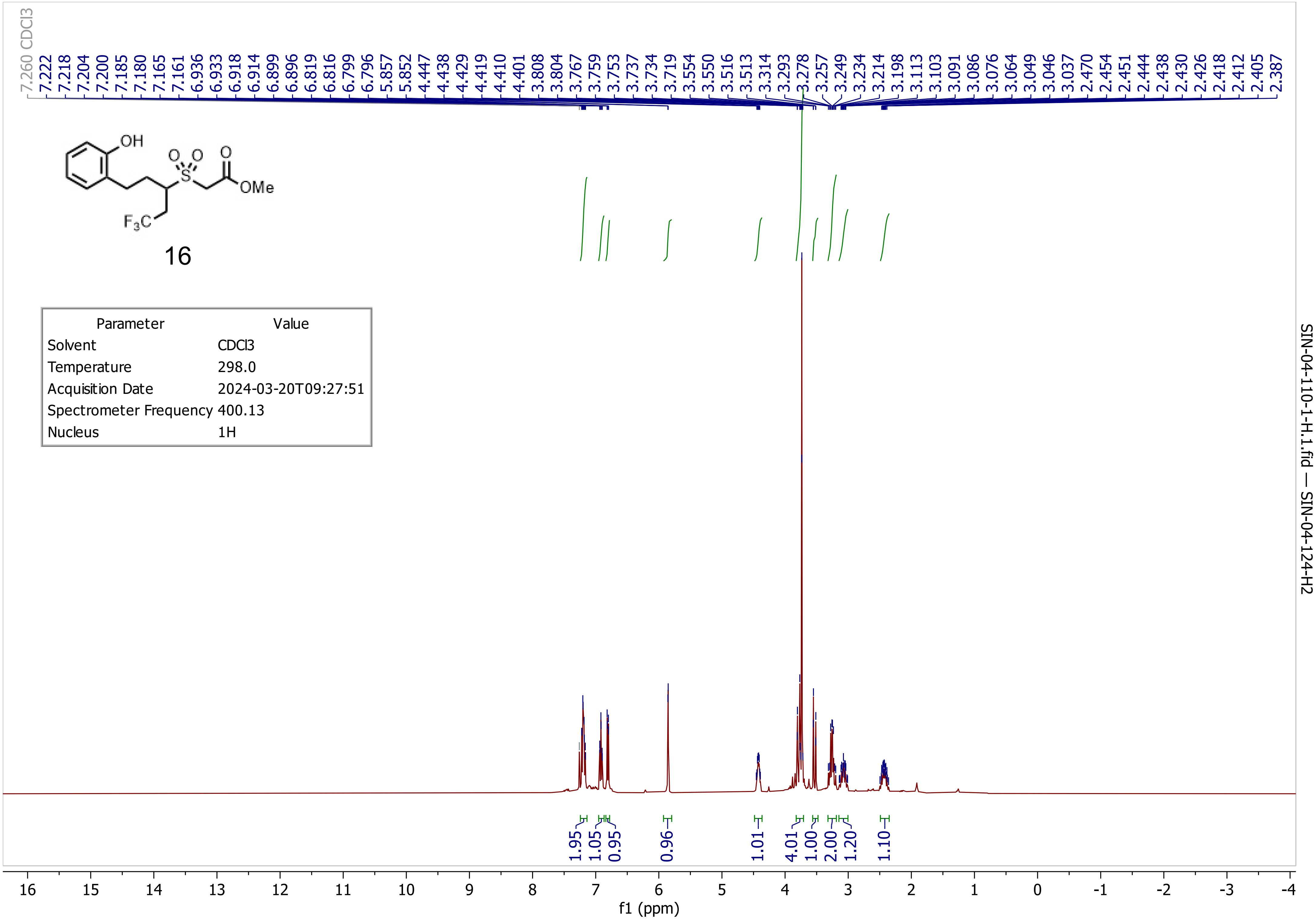

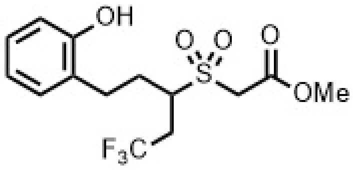

16

Parameter	Value
Solvent	CDCl3
Temperature	298.0
Acquisition Date	2024-03-20T02:12:45
Spectrometer Frequency	100.62
Nucleus	13C

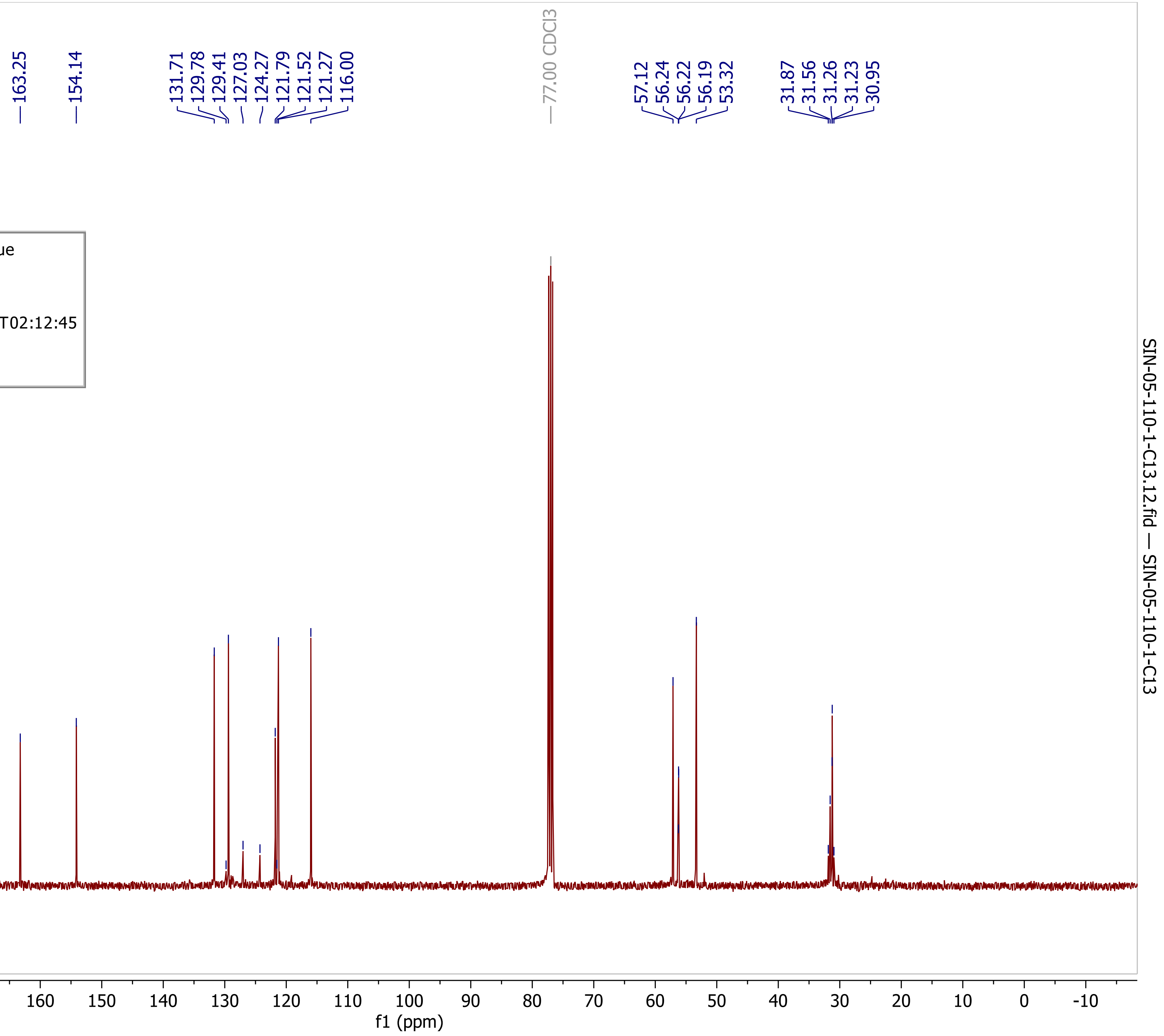

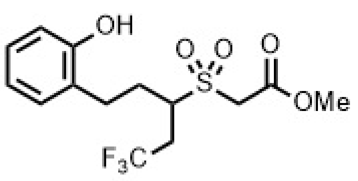

16

Parameter	Value
Solvent	CDCl3
Temperature	298.0
Acquisition Date	2024-03-20T00:53:12
Spectrometer Frequency	376.46
Nucleus	19F

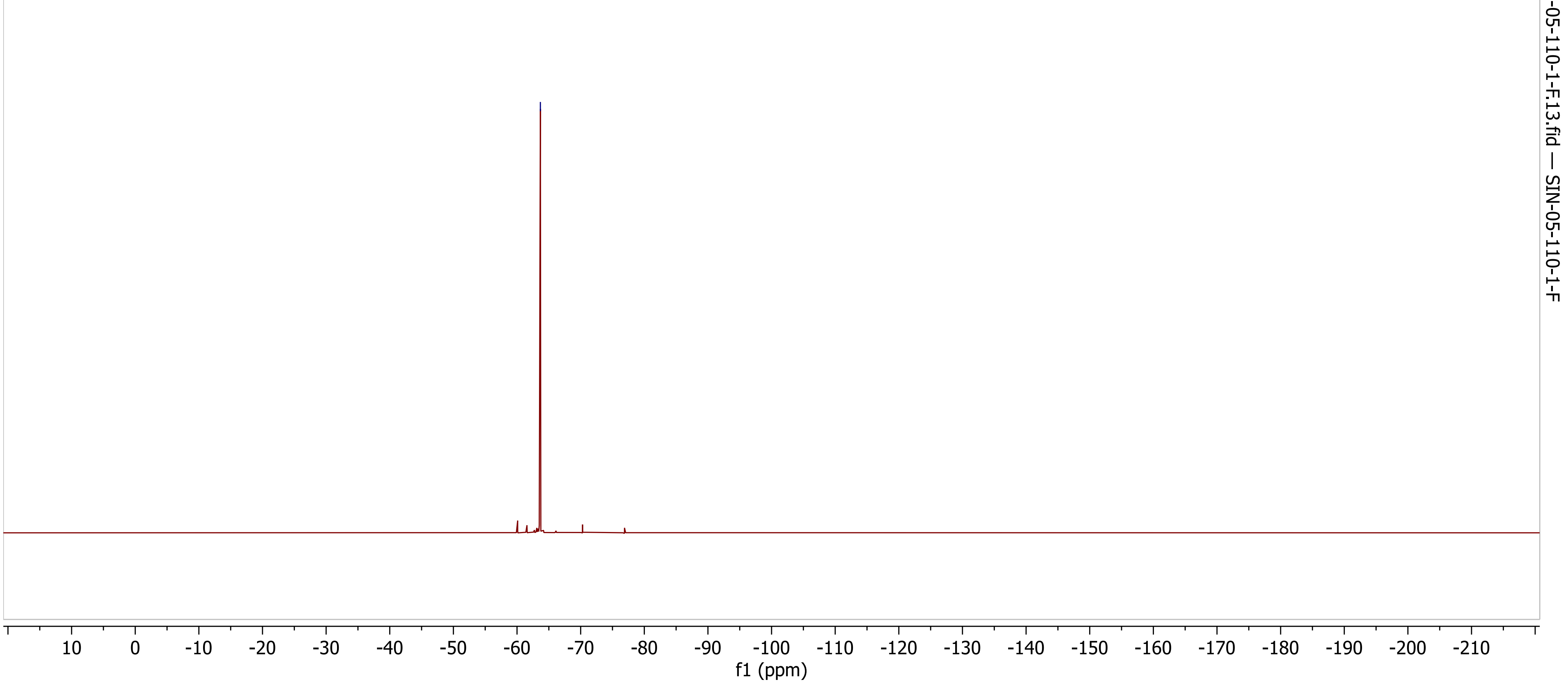

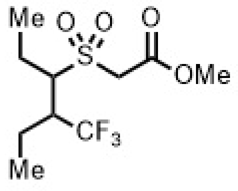

17

Parameter	Value
Solvent	CDCl3
Temperature	298.0
Acquisition Date	2024-05-10T14:49:56
Spectrometer Frequency	400.13
Nucleus	1H

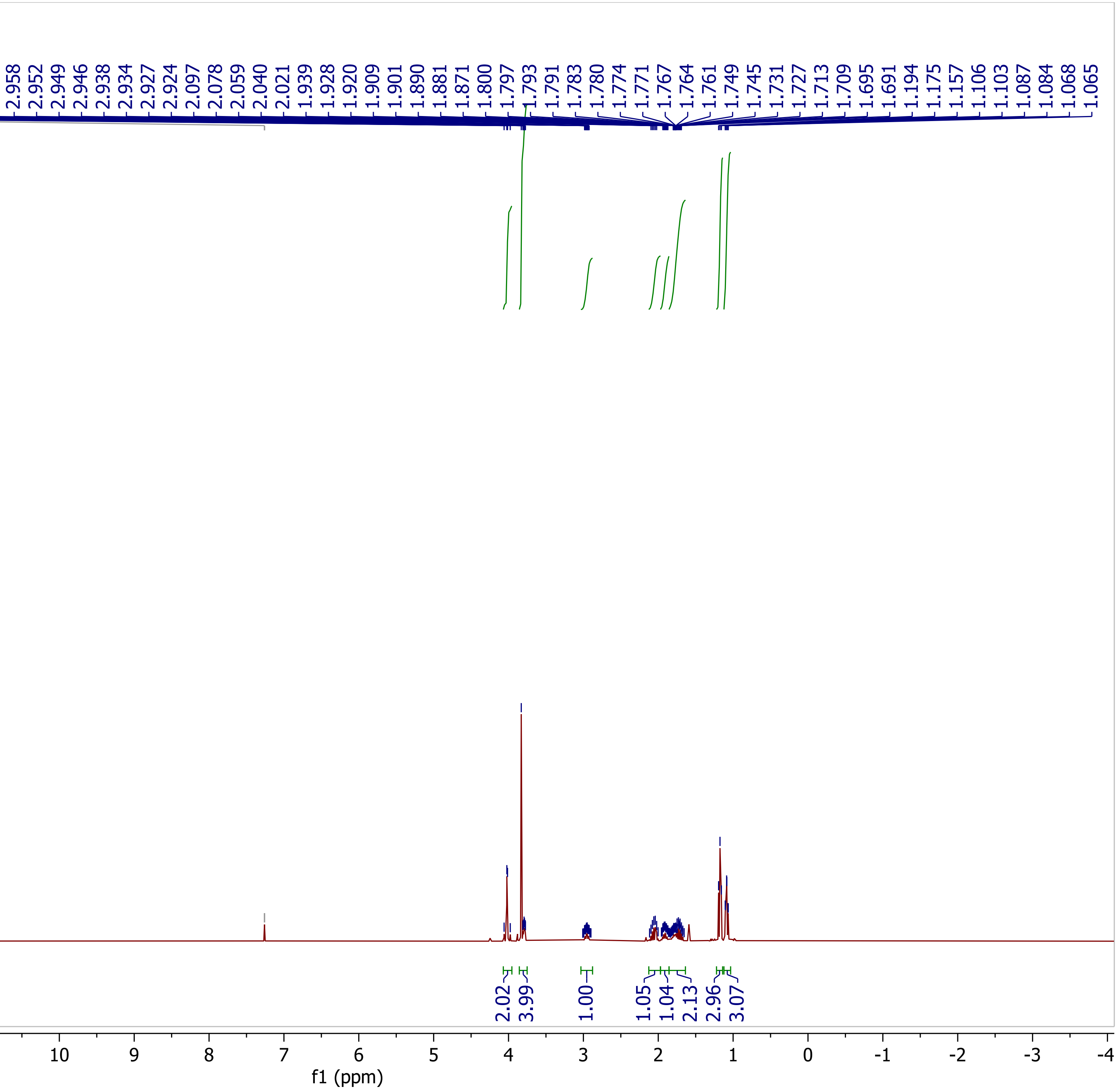

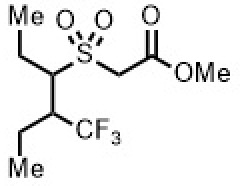

17

Parameter	Value
Solvent	CDCl3
Temperature	298.0
Acquisition Date	2024-05-11T00:57:02
Spectrometer Frequency	100.62
Nucleus	13C

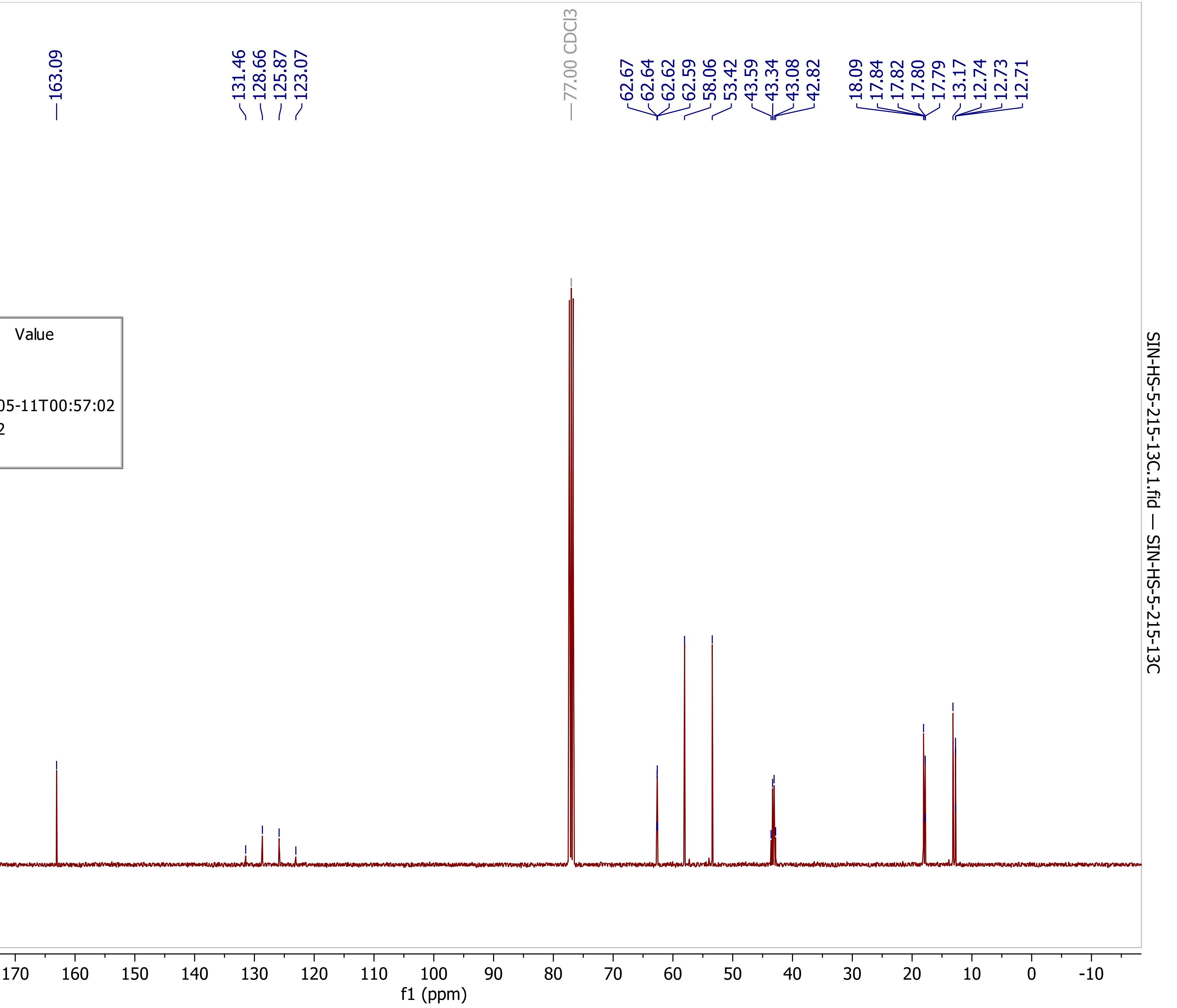

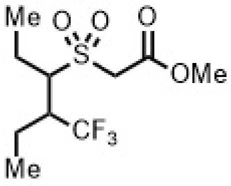

17

Parameter	Value
Solvent	CDCl3
Temperature	298.0
Acquisition Date	2024-08-06T19:16:32
Spectrometer Frequency	376.46
Nucleus	19F

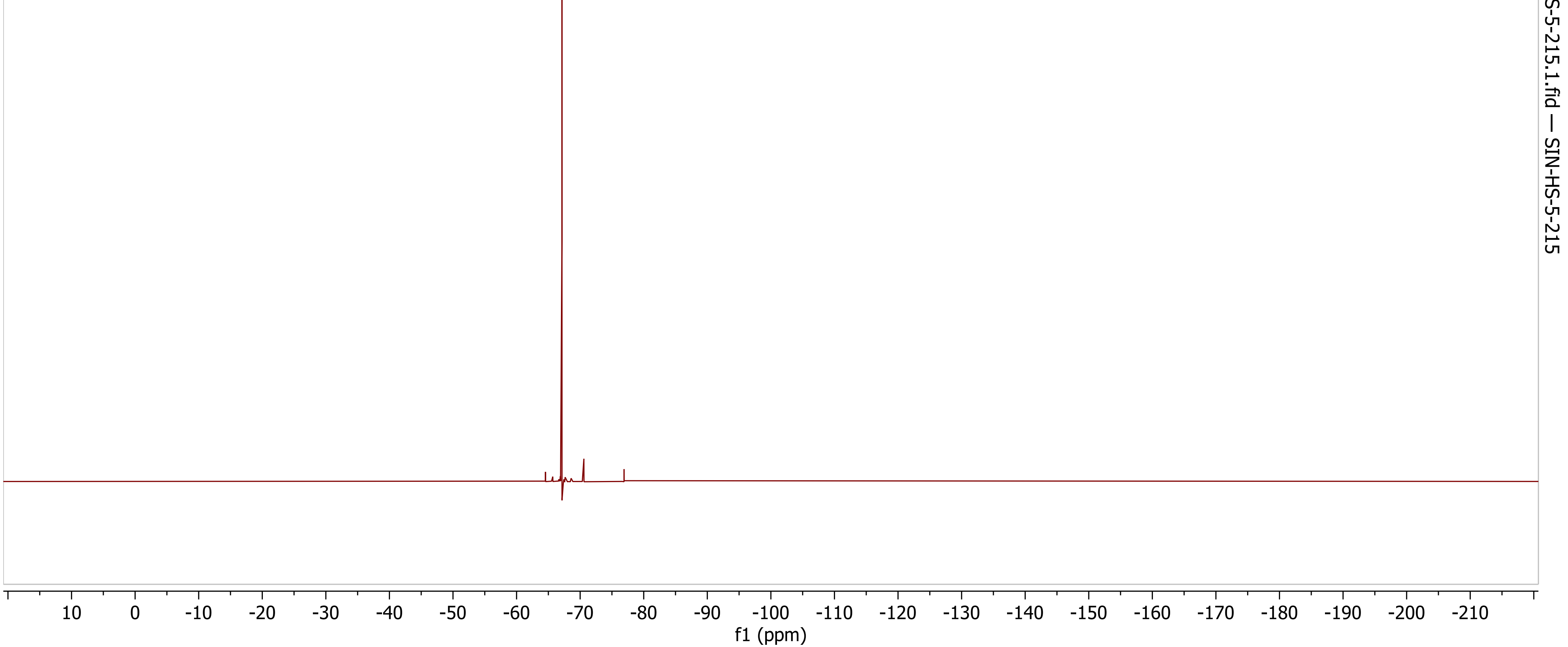

HSQC NMR

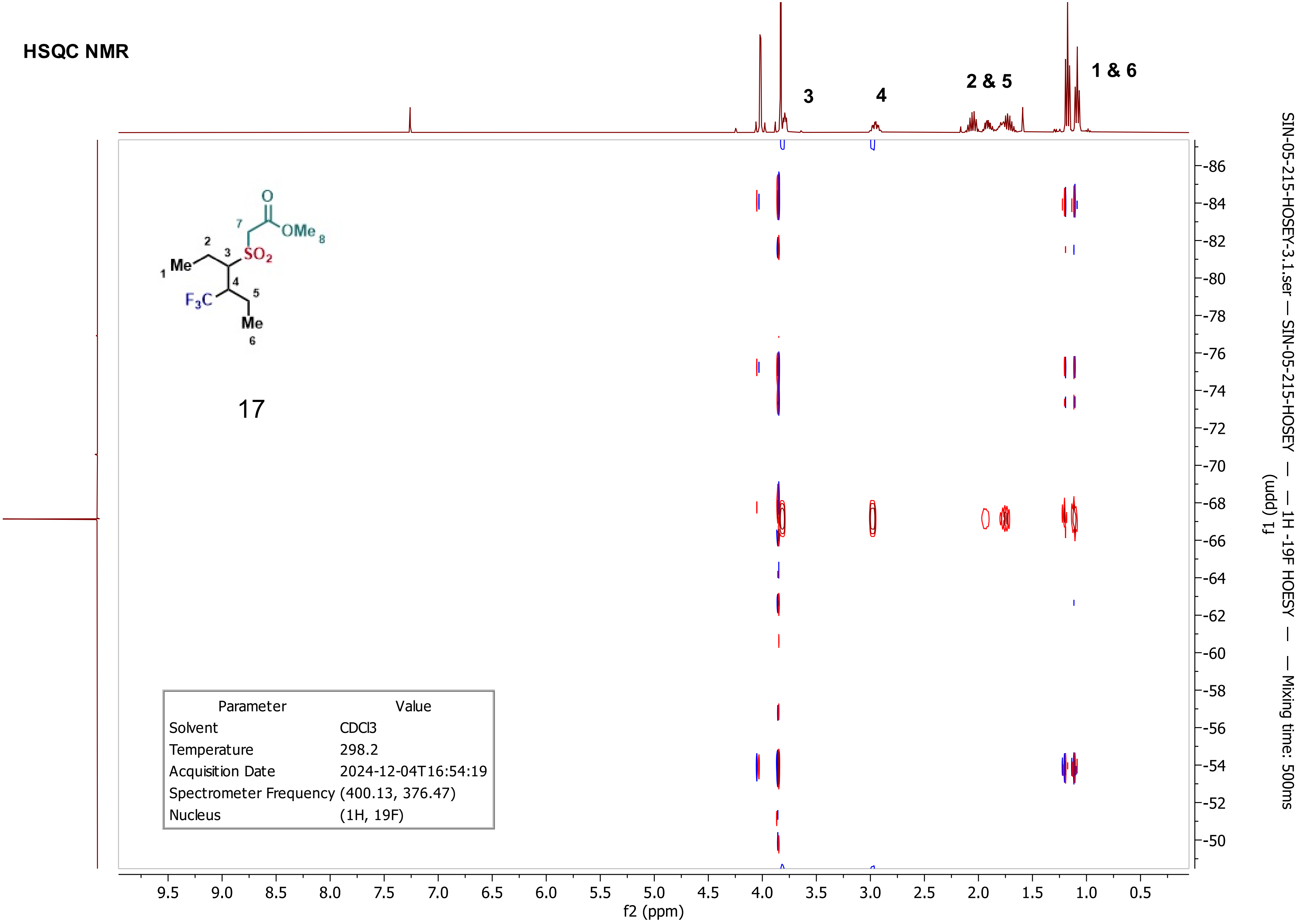

Parameter	Value
Solvent	CDCl3
Temperature	298.2
Acquisition Date	2024-12-04T16:54:19
Spectrometer Frequency	(400.13, 376.47)
Nucleus	(1H, 19F)

HSQC NMR

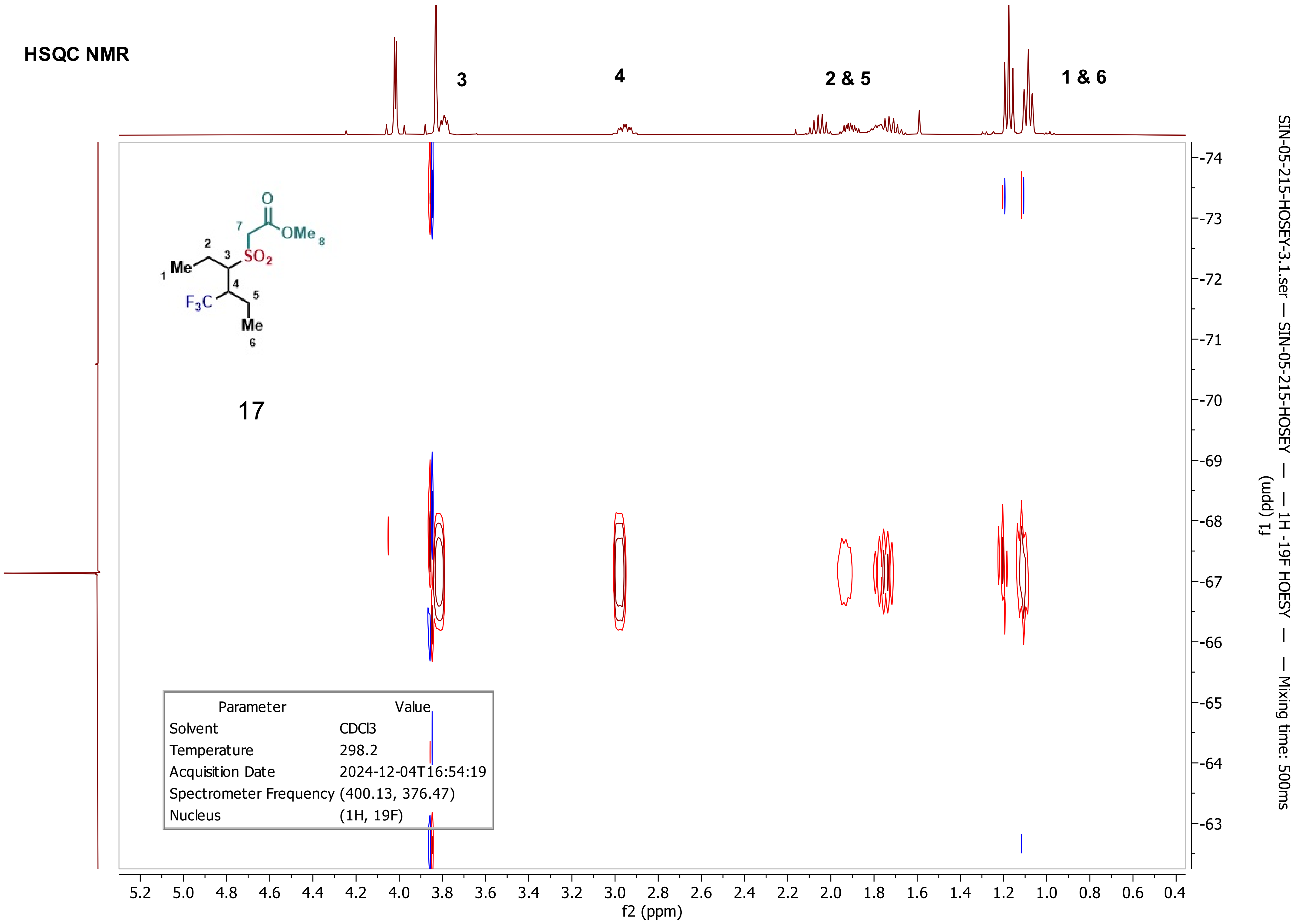
3
4
2 & 5
1 & 6
O
OMe
8
7
2
3
SO2
Me
1
4
5
F3C
Me
6
17
Parameter
Value
Solvent
CDCl3
Temperature
298.2
Acquisition Date
2024-12-04T16:54:19
Spectrometer Frequency
(400.13, 376.47)
Nucleus
(1H, 19F)
f2 (ppm)
f1 (ppm)
SIN-05-215-HOSEY-3.1.ser — SIN-05-215-HOSEY — — 1H -19F HOESY — — Mixing time: 500ms

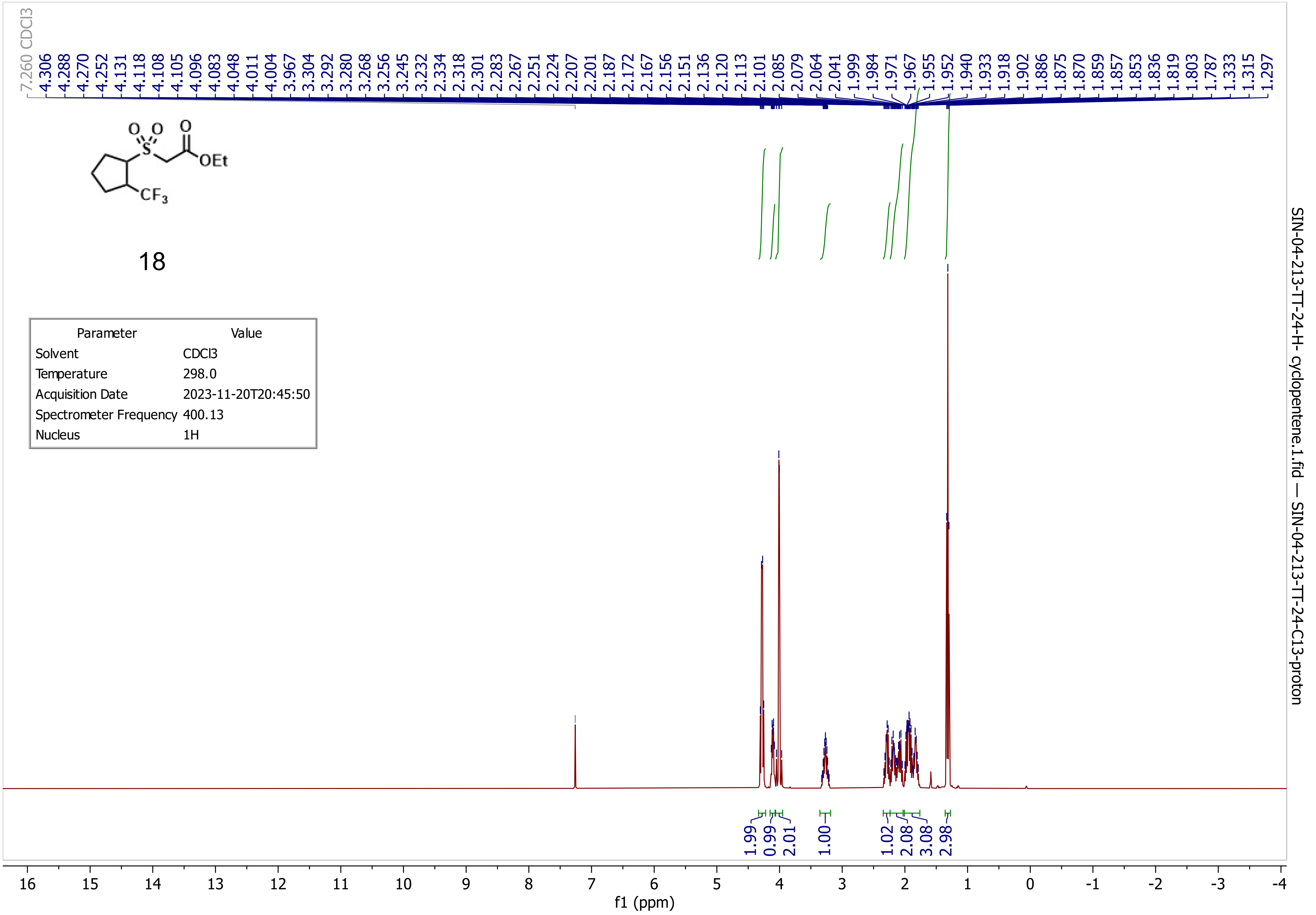

Parameter	Value
Solvent	CDCl3
Temperature	298.0
Acquisition Date	2023-11-20T20:45:50
Spectrometer Frequency	400.13
Nucleus	1H

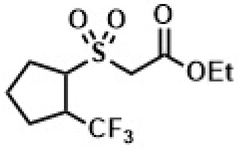

18

Parameter	Value
Solvent	CDCl3
Temperature	298.0
Acquisition Date	2023-11-20T21:50:29
Spectrometer Frequency	100.62
Nucleus	13C

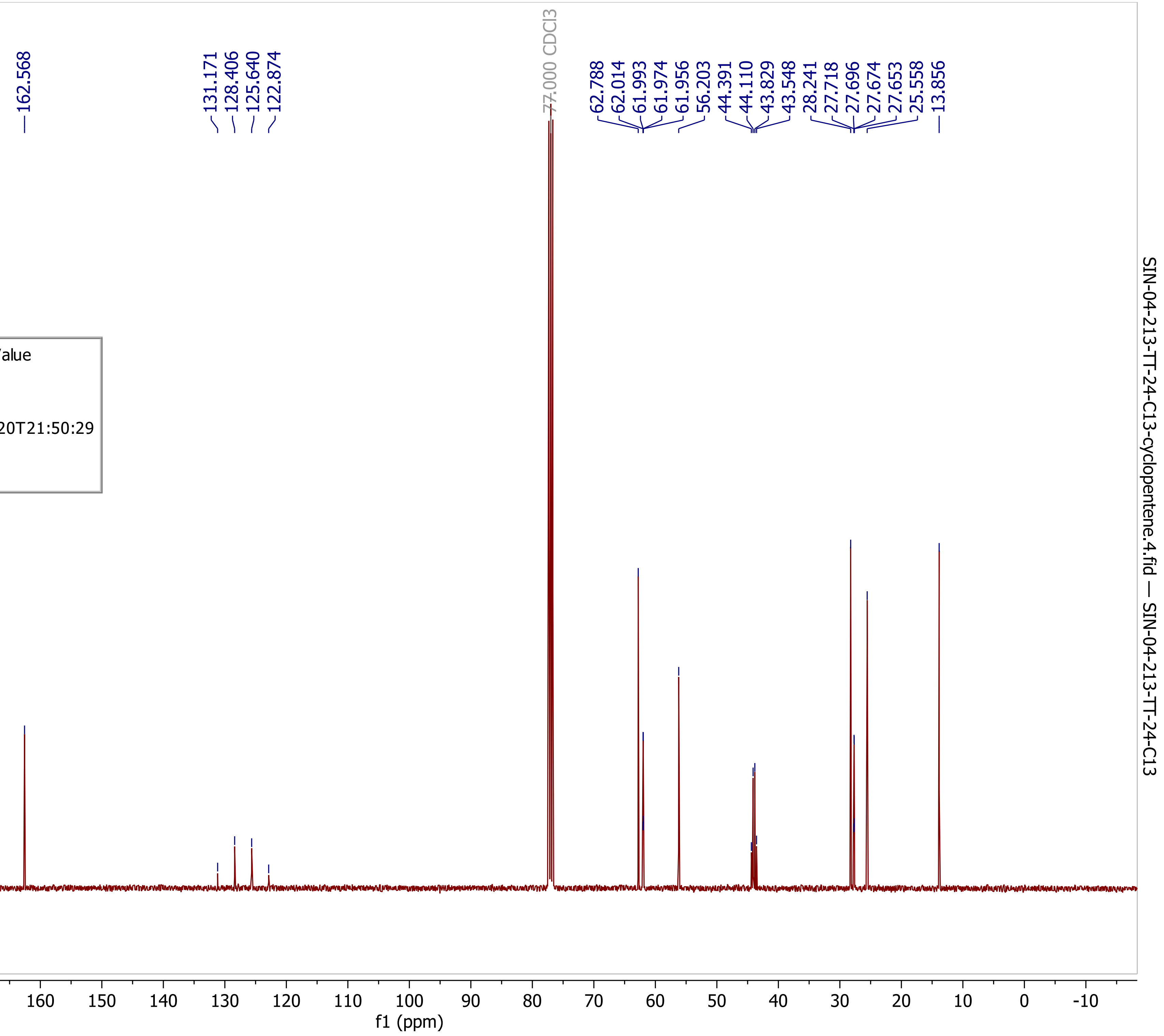

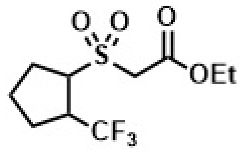

18

Parameter	Value
Solvent	CDCl3
Temperature	298.0
Acquisition Date	2023-11-20T20:50:12
Spectrometer Frequency	376.46
Nucleus	19F

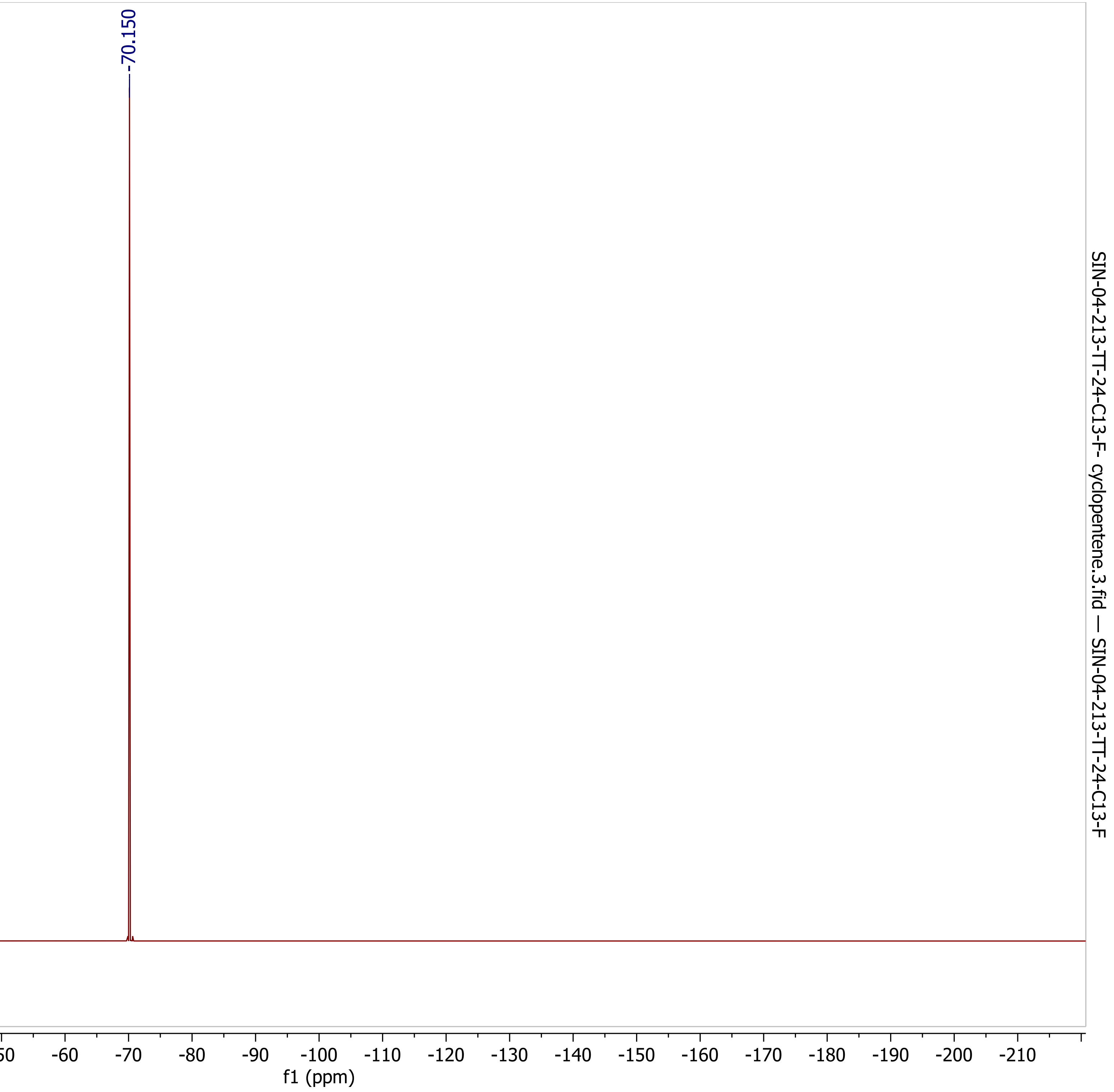

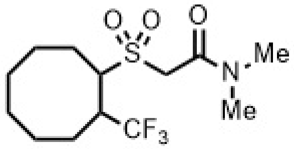

19

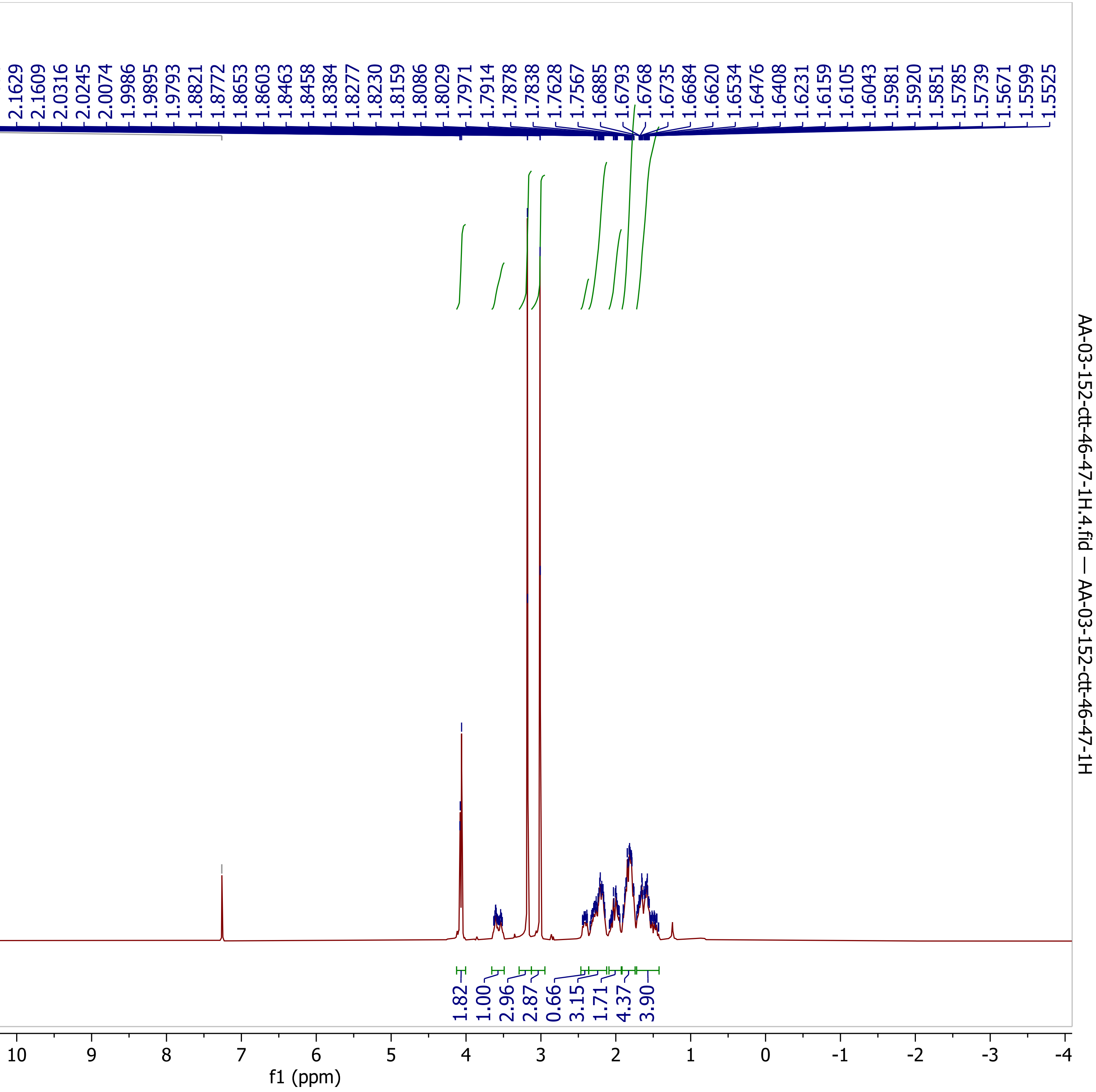

Parameter	Value
Solvent	CDCl3
Temperature	298.0
Acquisition Date	2024-10-03T23:22:18
Spectrometer Frequency	400.13
Nucleus	1H

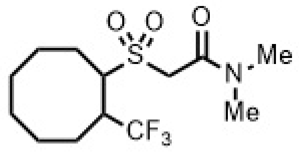

19

Parameter	Value
Solvent	CDCl3
Temperature	298.0
Acquisition Date	2024-11-05T00:35:01
Spectrometer Frequency	100.62
Nucleus	13C

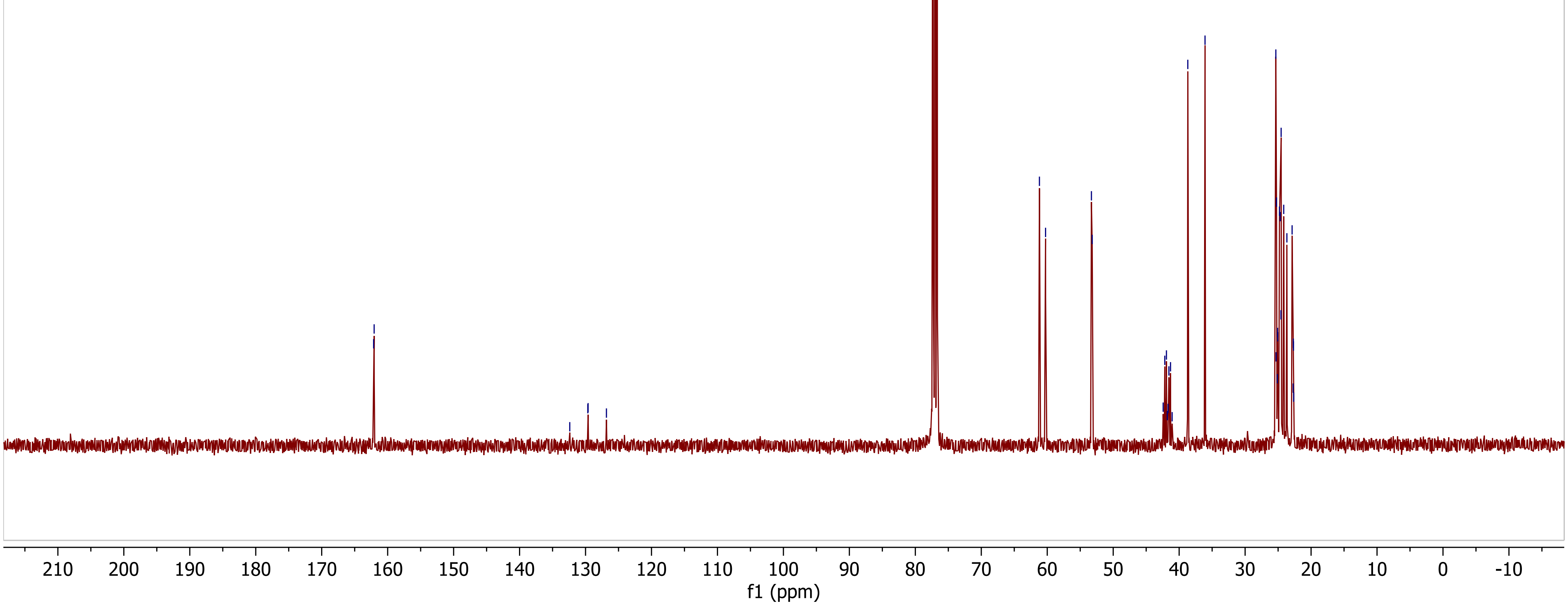

AA-CF3-Octene-Carbon.1.fid — AA-CF3-Octene-Carbon

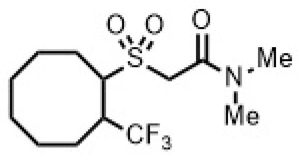

19

Parameter	Value
Solvent	CDCl3
Temperature	298.0
Acquisition Date	2024-10-04T00:27:31
Spectrometer Frequency	376.46
Nucleus	19F

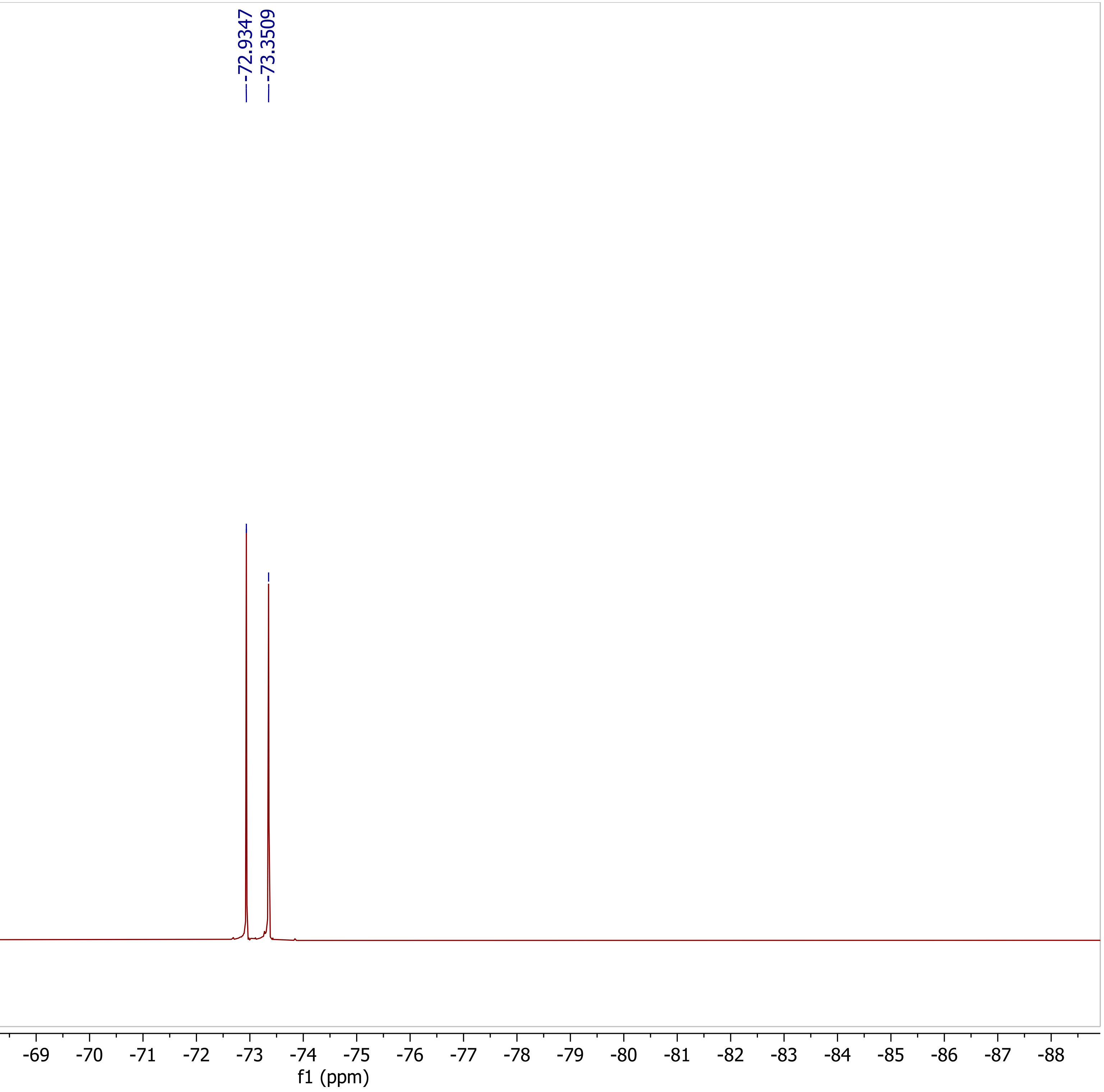

AA-03-152-ctt-46-47-19F6.fid — AA-03-152-ctt-46-47-19F

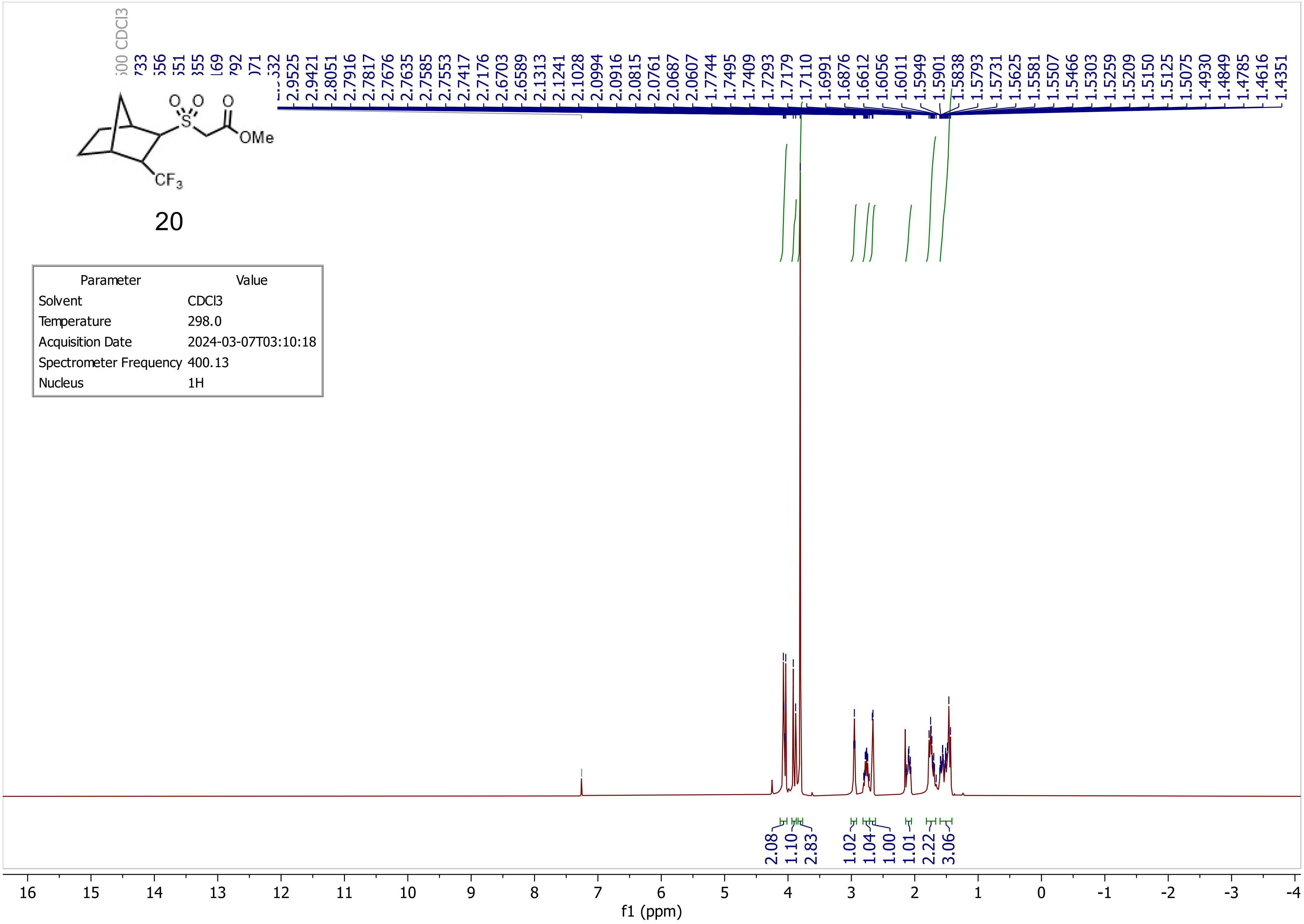

20

Parameter	Value
Solvent	CDCl3
Temperature	298.0
Acquisition Date	2024-03-07T03:10:18
Spectrometer Frequency	400.13
Nucleus	1H

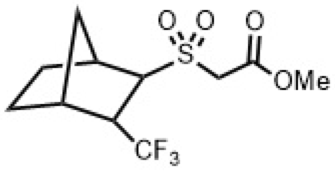

20

Parameter	Value
Solvent	CDCl3
Temperature	298.0
Acquisition Date	2024-03-07T04:20:36
Spectrometer Frequency	100.62
Nucleus	13C

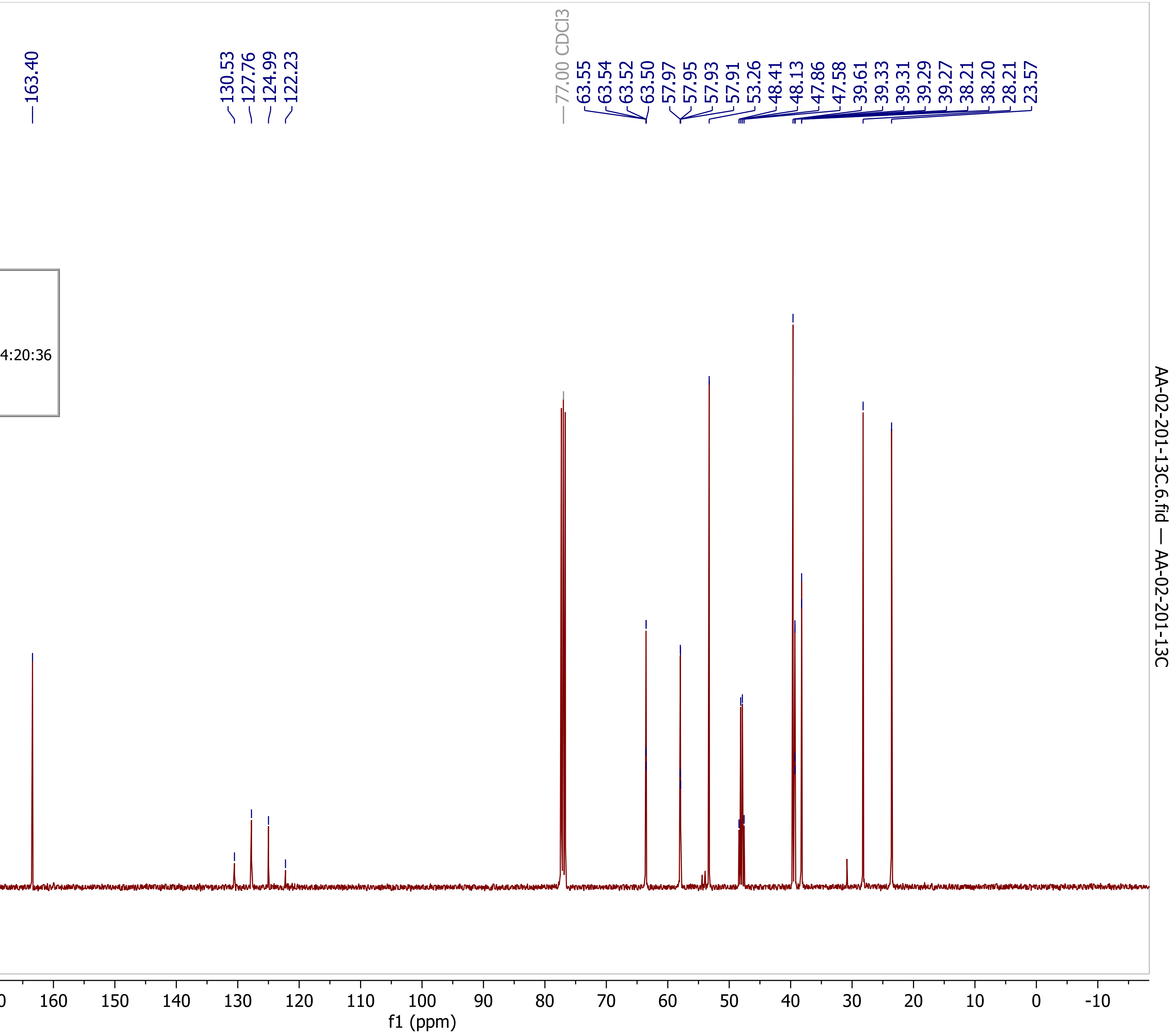

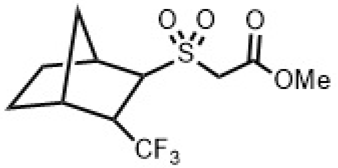

20

Parameter	Value
Solvent	CDCl3
Temperature	298.0
Acquisition Date	2024-03-07T04:24:49
Spectrometer Frequency	376.46
Nucleus	19F

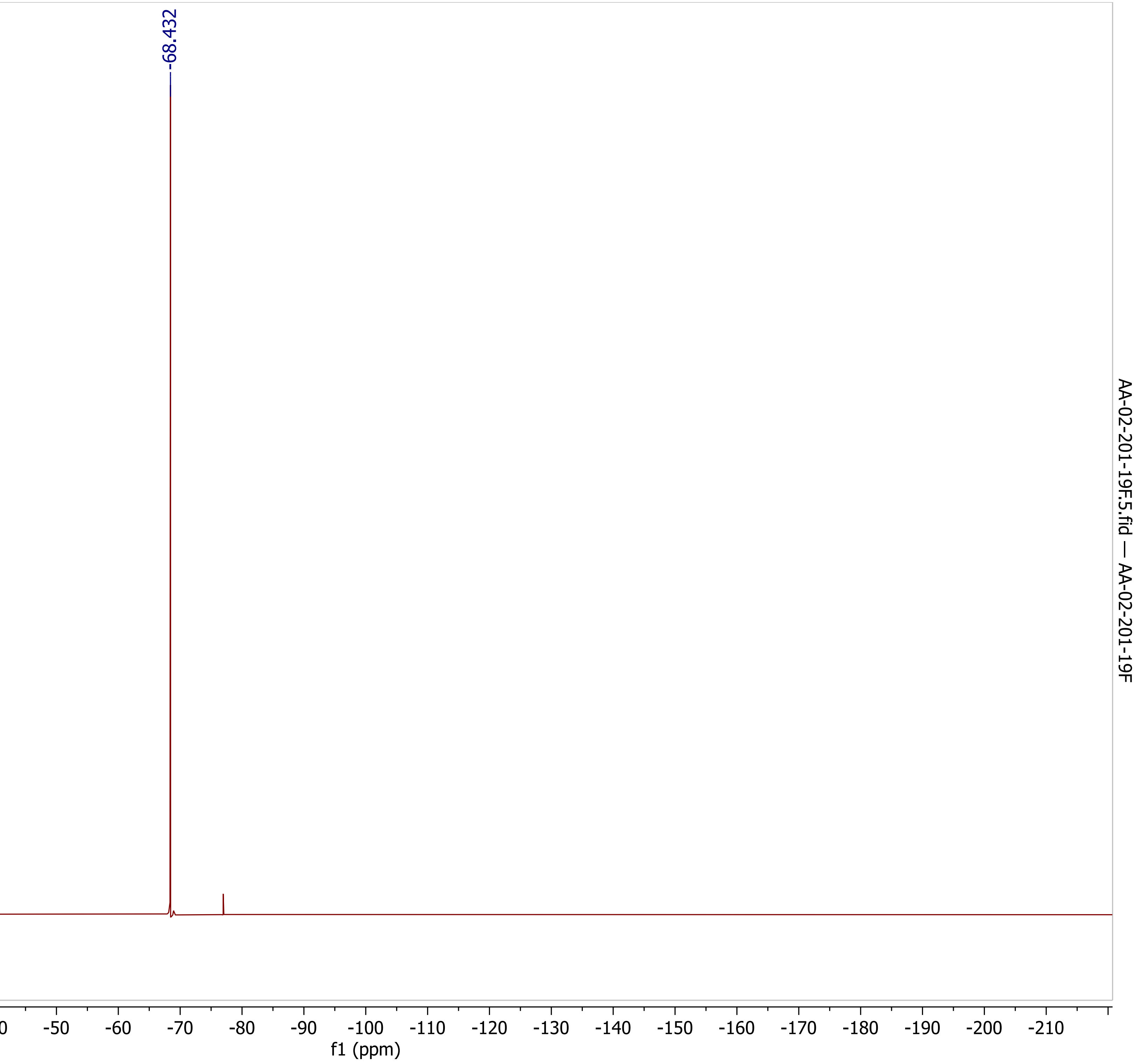

HSQC NMR

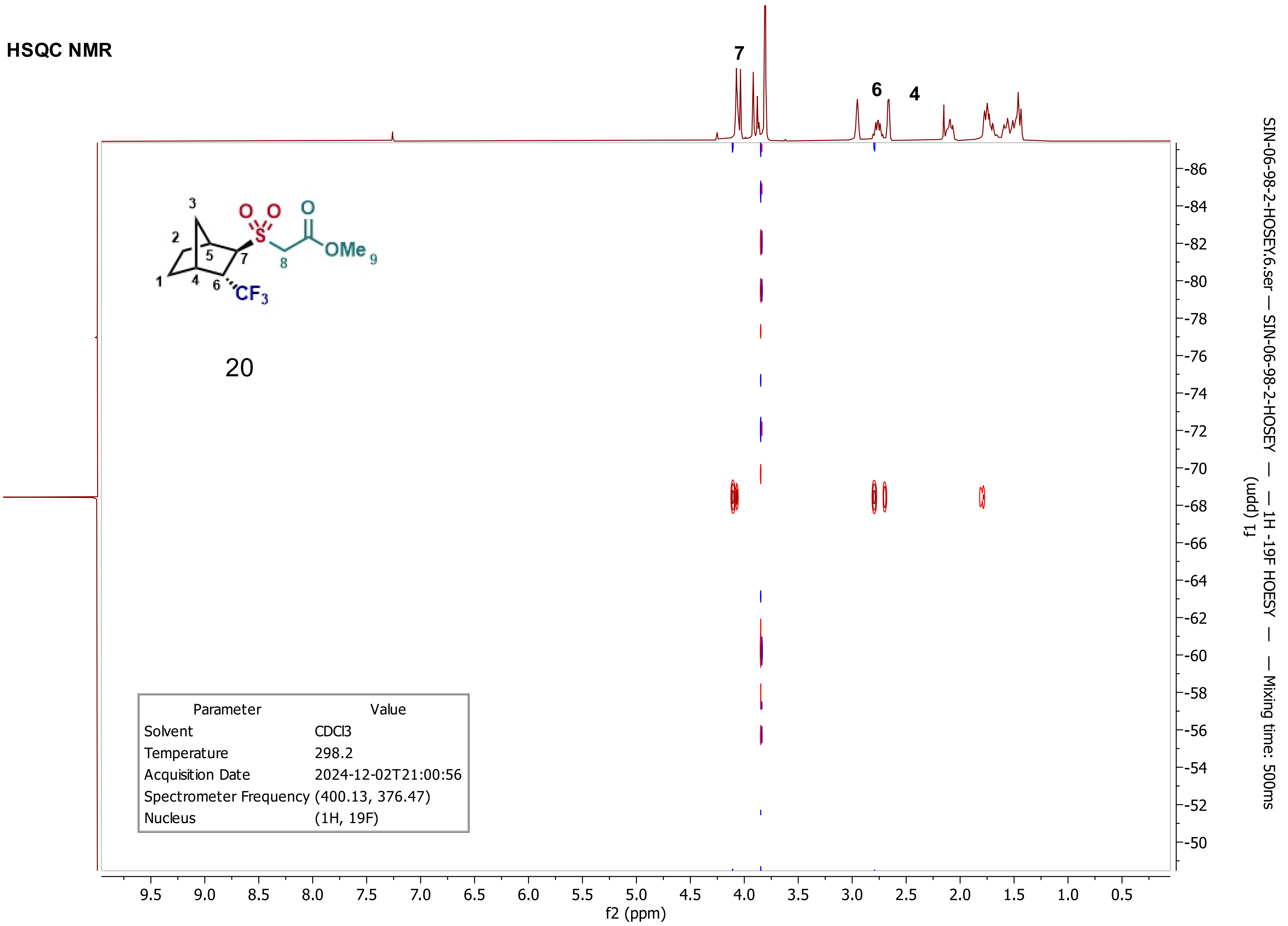

Parameter	Value
Solvent	CDCl3
Temperature	298.2
Acquisition Date	2024-12-02T21:00:56
Spectrometer Frequency	(400.13, 376.47)
Nucleus	(1H, 19F)

HSQC NMR

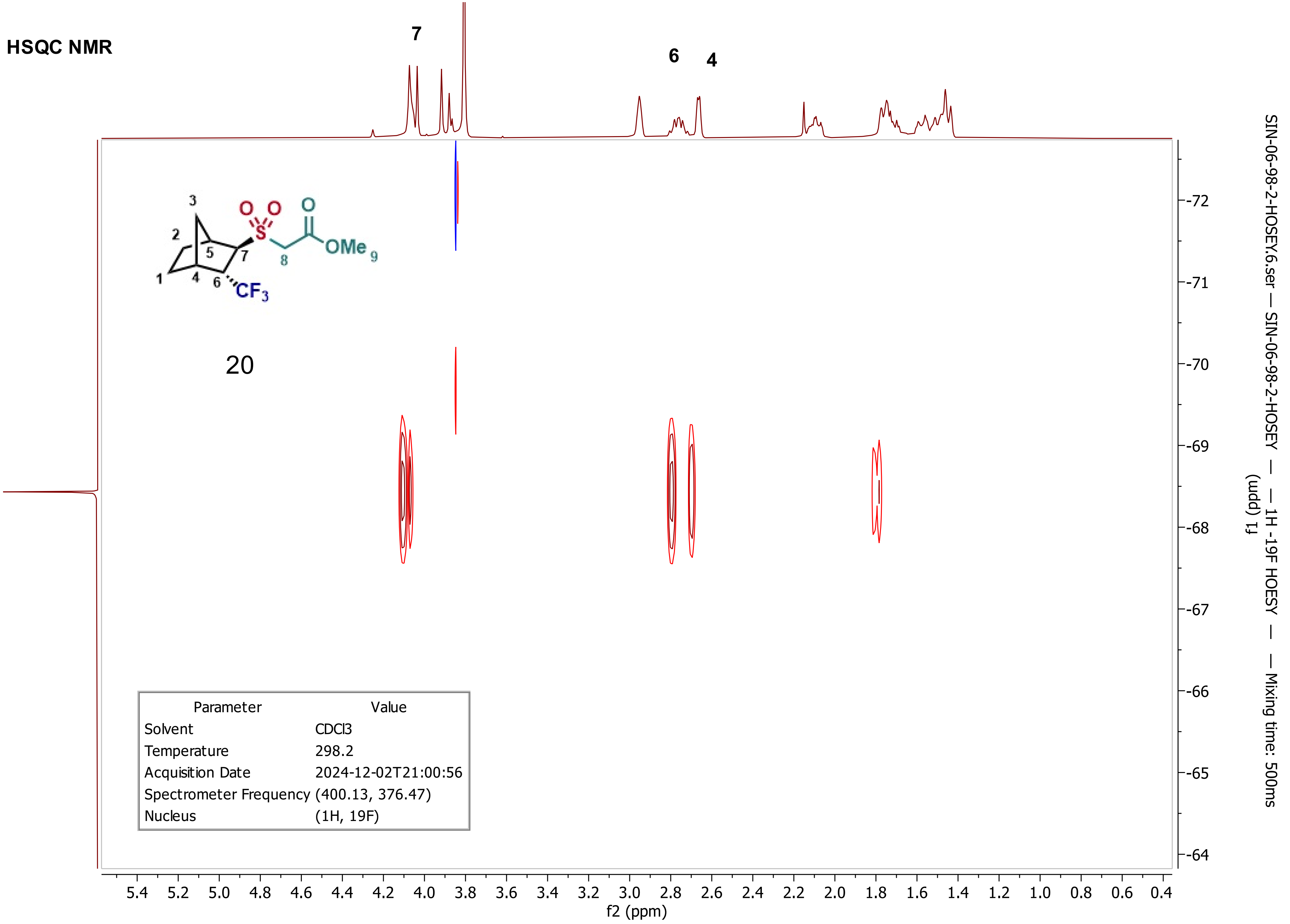

Parameter	Value
Solvent	CDCl3
Temperature	298.2
Acquisition Date	2024-12-02T21:00:56
Spectrometer Frequency	(400.13, 376.47)
Nucleus	(1H, 19F)

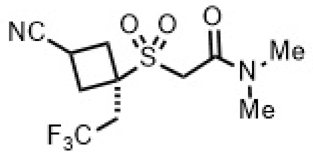

21

Parameter	Value
Solvent	CDCl3
Temperature	298.0
Acquisition Date	2024-09-02T23:07:27
Spectrometer Frequency	400.13
Nucleus	1H

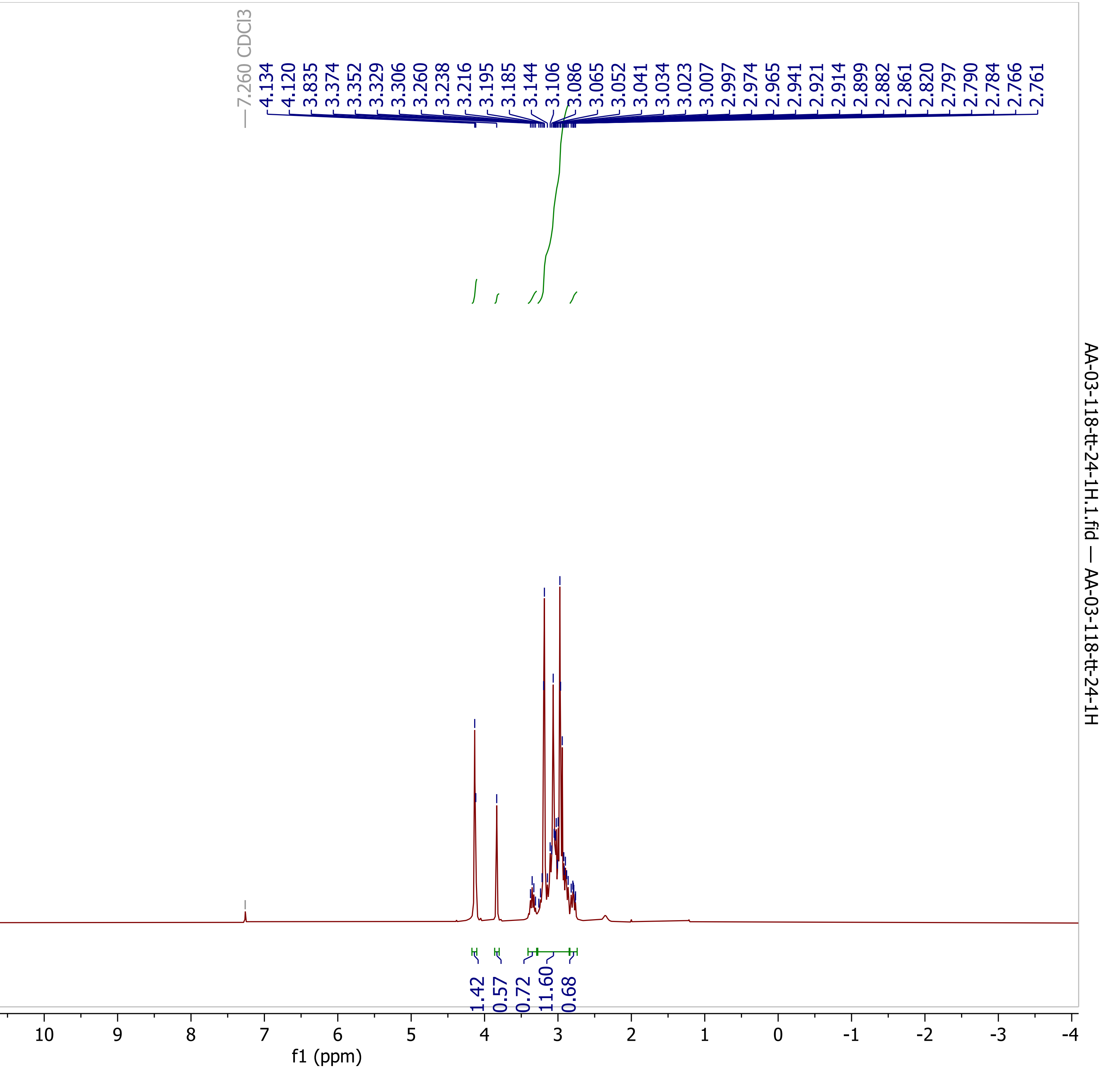

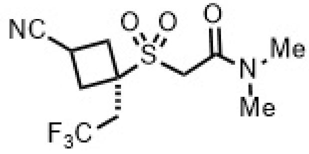

21

Parameter	Value
Solvent	CDCl3
Temperature	298.0
Acquisition Date	2024-09-02T23:37:13
Spectrometer Frequency	100.62
Nucleus	13C

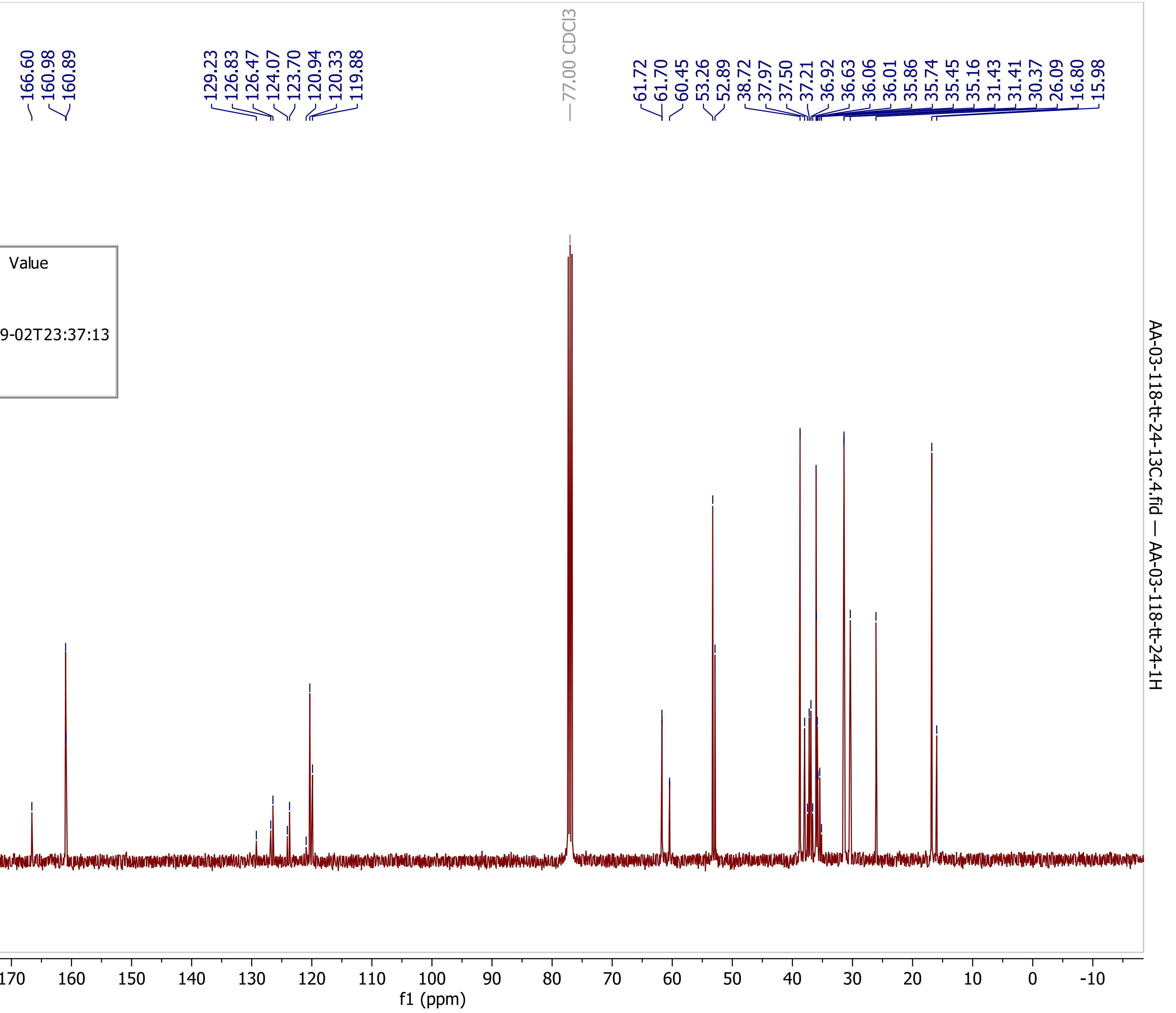

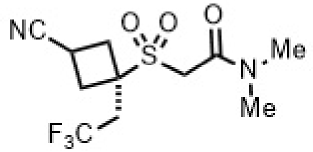

21

Parameter	Value
Solvent	CDCl3
Temperature	298.0
Acquisition Date	2024-09-02T23:41:32
Spectrometer Frequency	376.46
Nucleus	19F

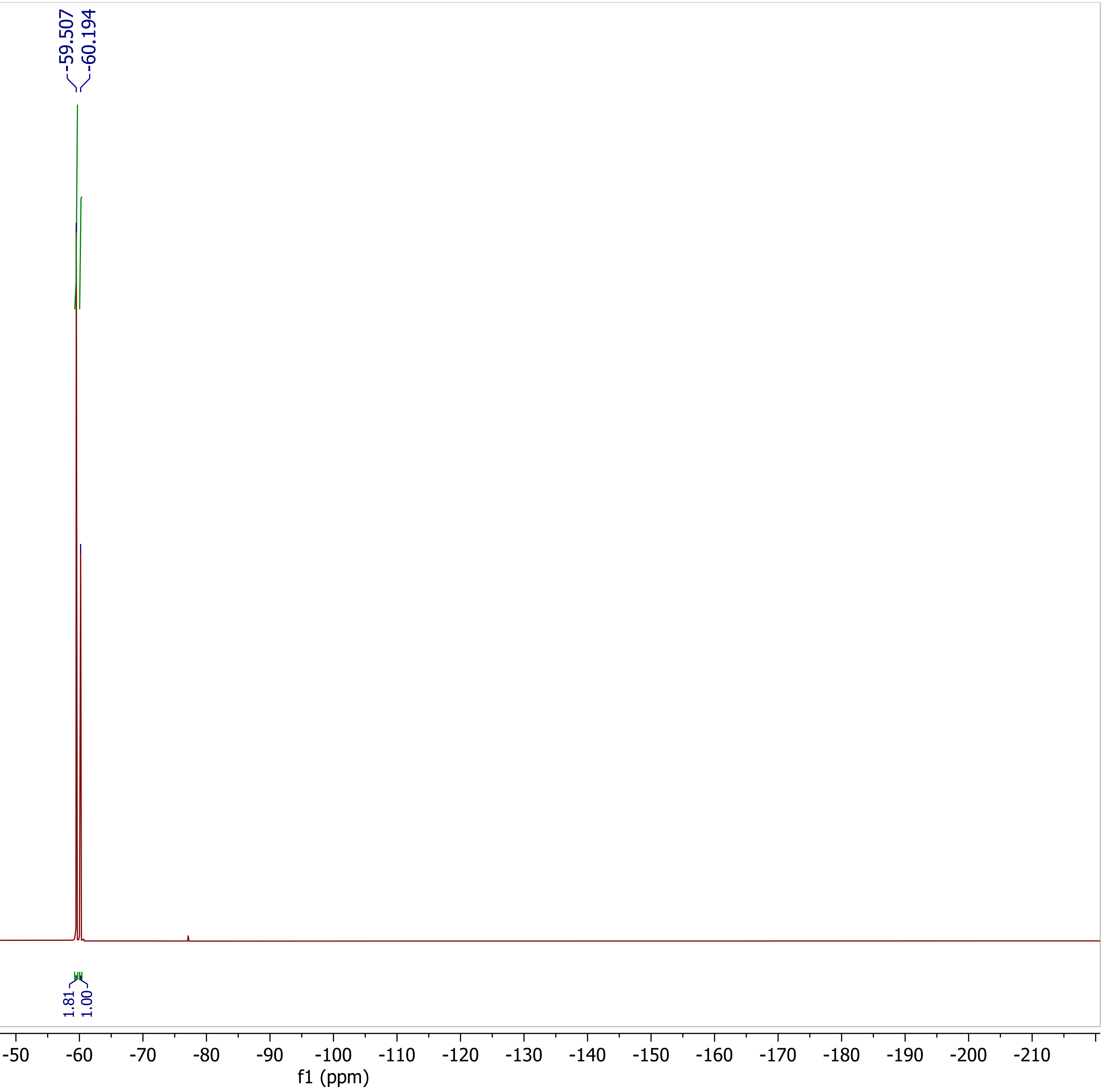

AA-03-118-tt-24-19F.5.fid — AA-03-118-tt-24-1H

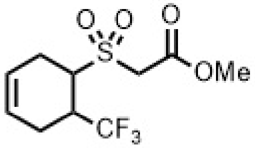

22

Parameter	Value
Solvent	CDCl3
Temperature	298.0
Acquisition Date	2024-03-07T01:48:19
Spectrometer Frequency	400.13
Nucleus	1H

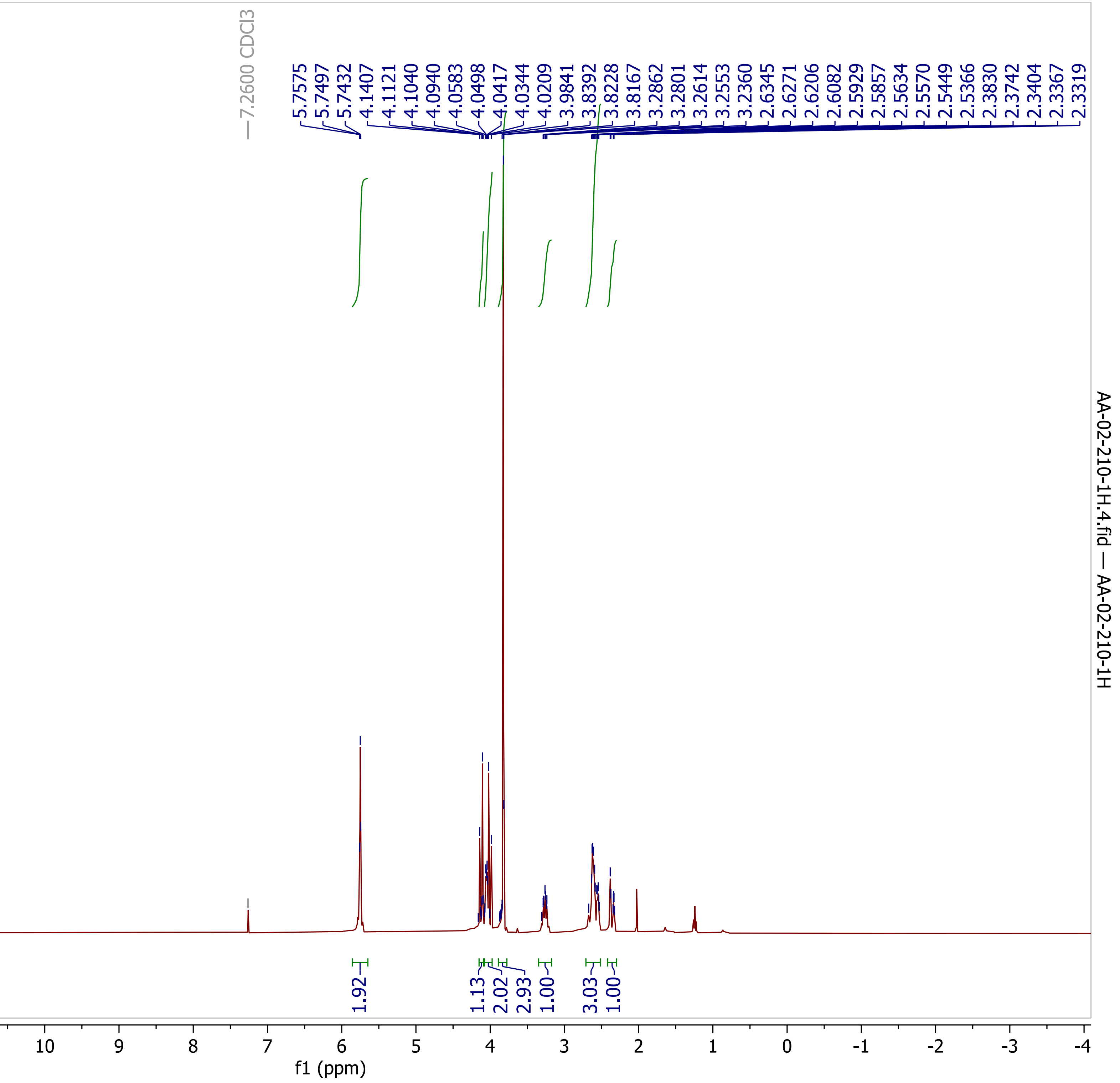

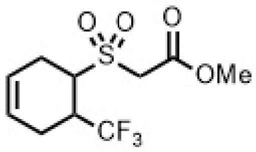

22

Parameter	Value
Solvent	CDCl3
Temperature	298.0
Acquisition Date	2024-03-07T02:58:33
Spectrometer Frequency	100.62
Nucleus	13C

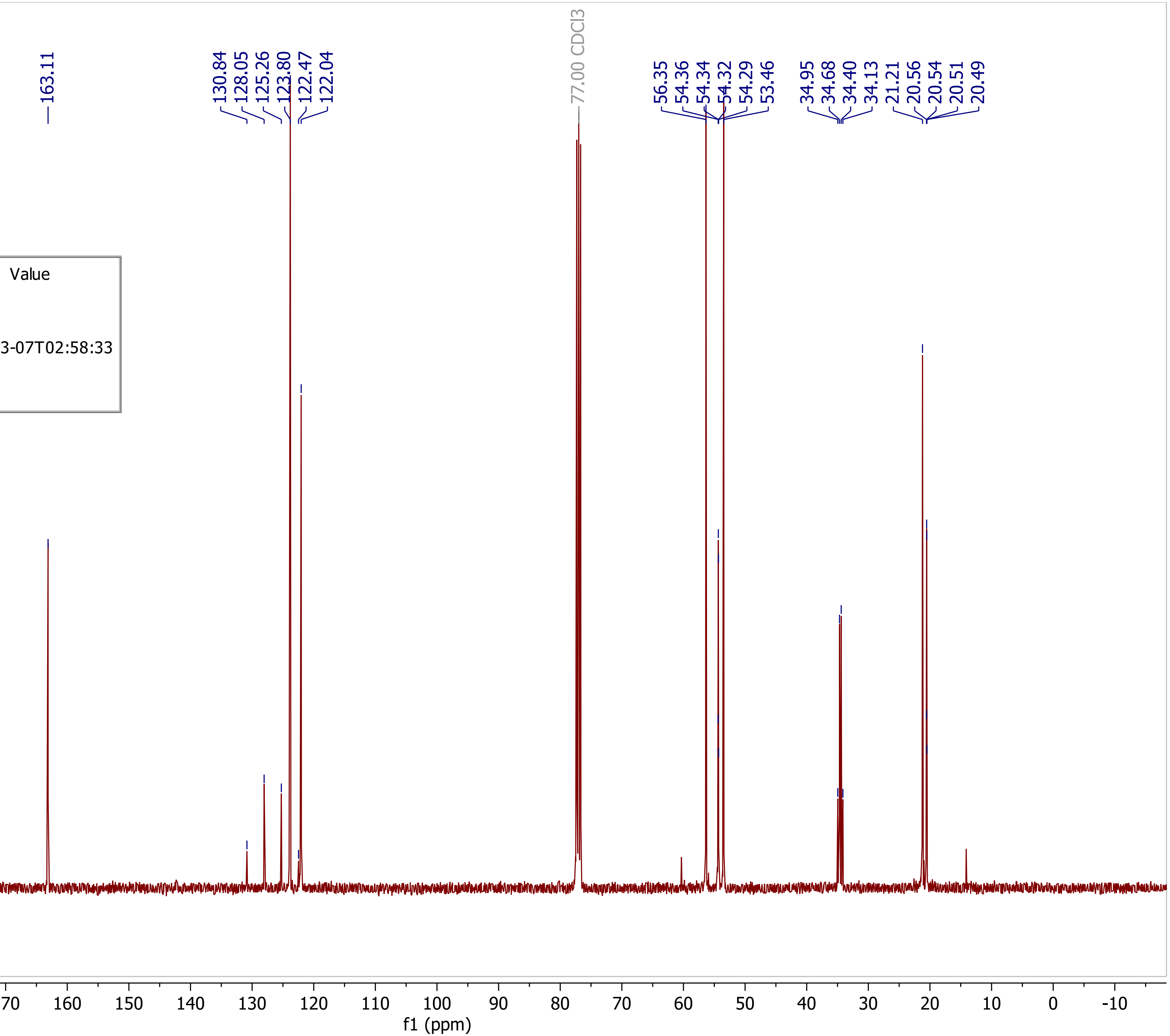

AA-02-210-13C.5.fid — AA-02-210-13C

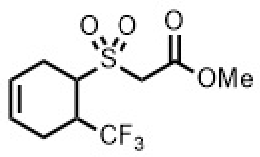

22

Parameter	Value
Solvent	CDCl3
Temperature	298.0
Acquisition Date	2024-03-07T03:02:48
Spectrometer Frequency	376.46
Nucleus	19F

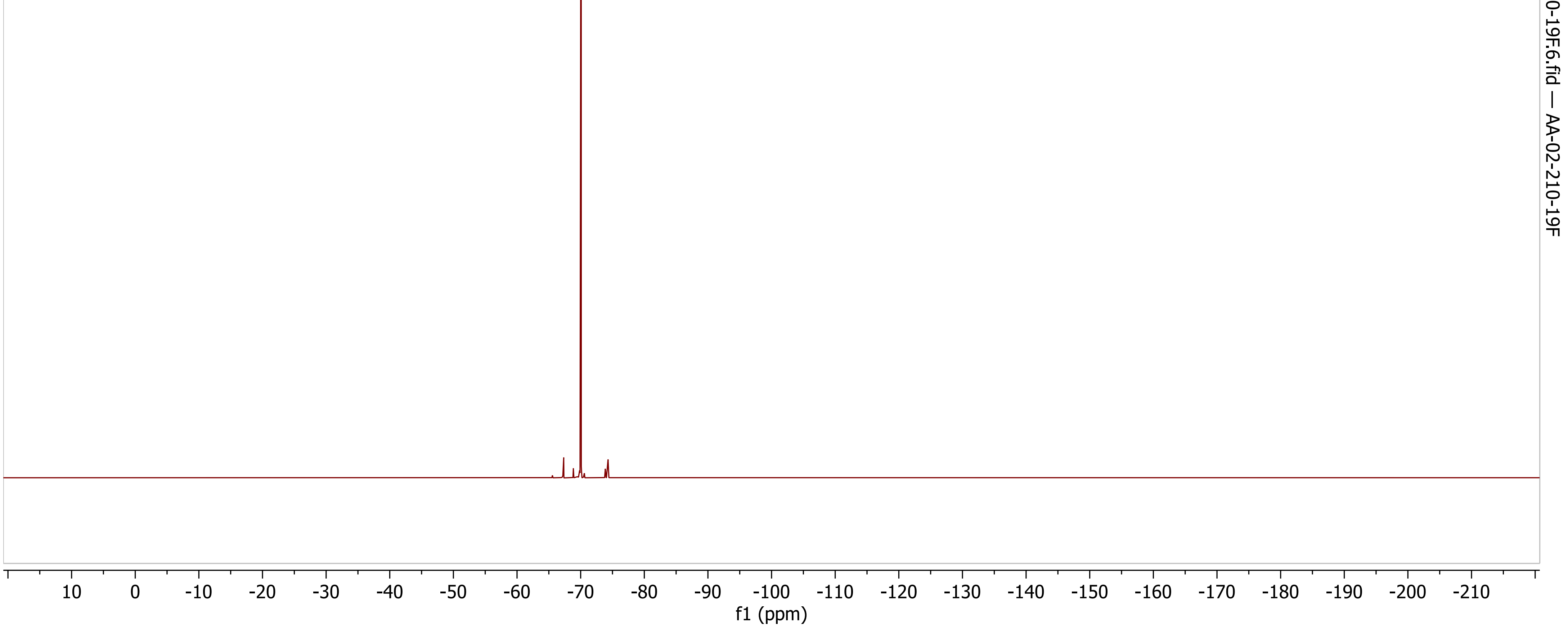

HSQC NMR

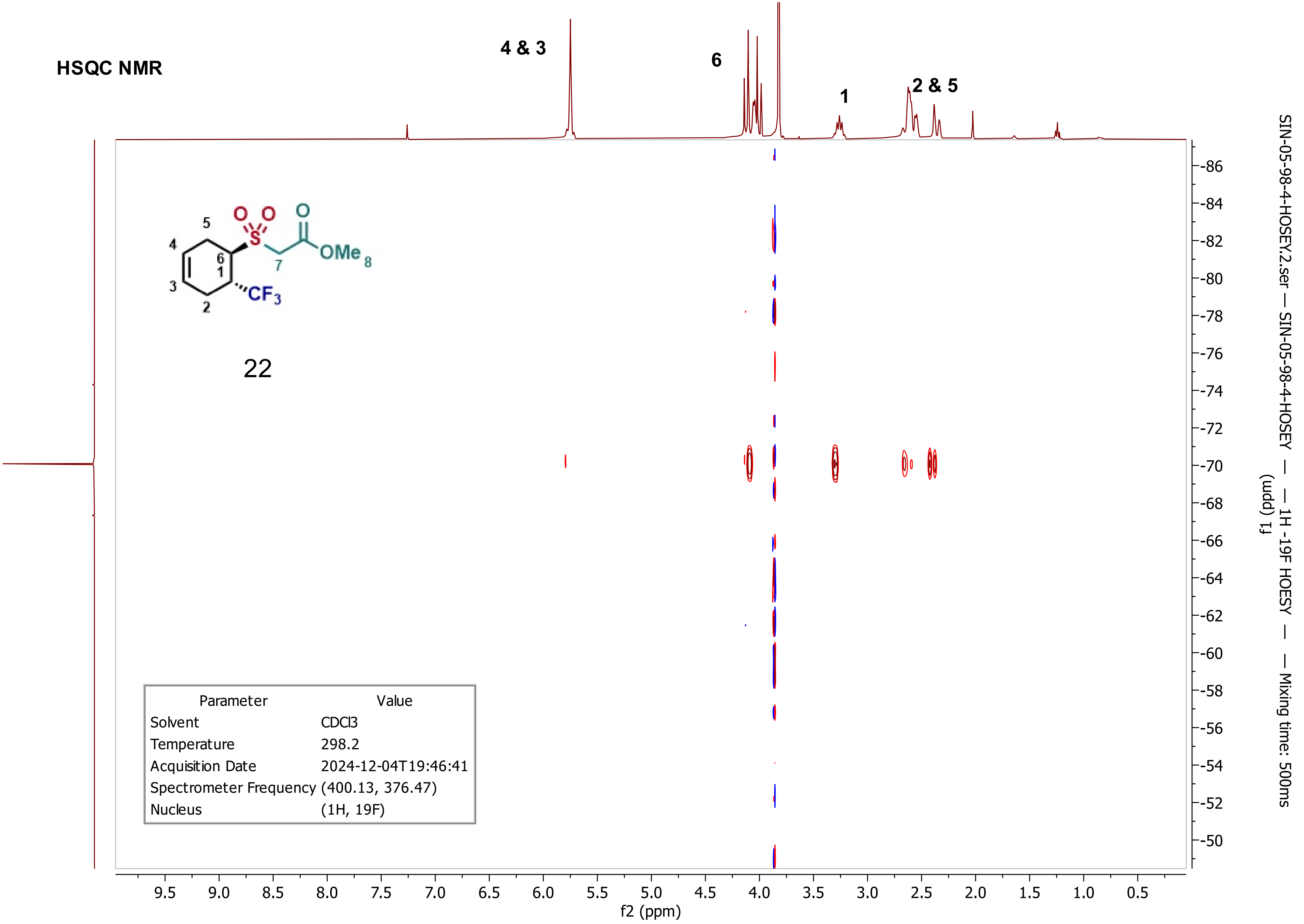

Parameter	Value
Solvent	CDCl3
Temperature	298.2
Acquisition Date	2024-12-04T19:46:41
Spectrometer Frequency	(400.13, 376.47)
Nucleus	(1H, 19F)

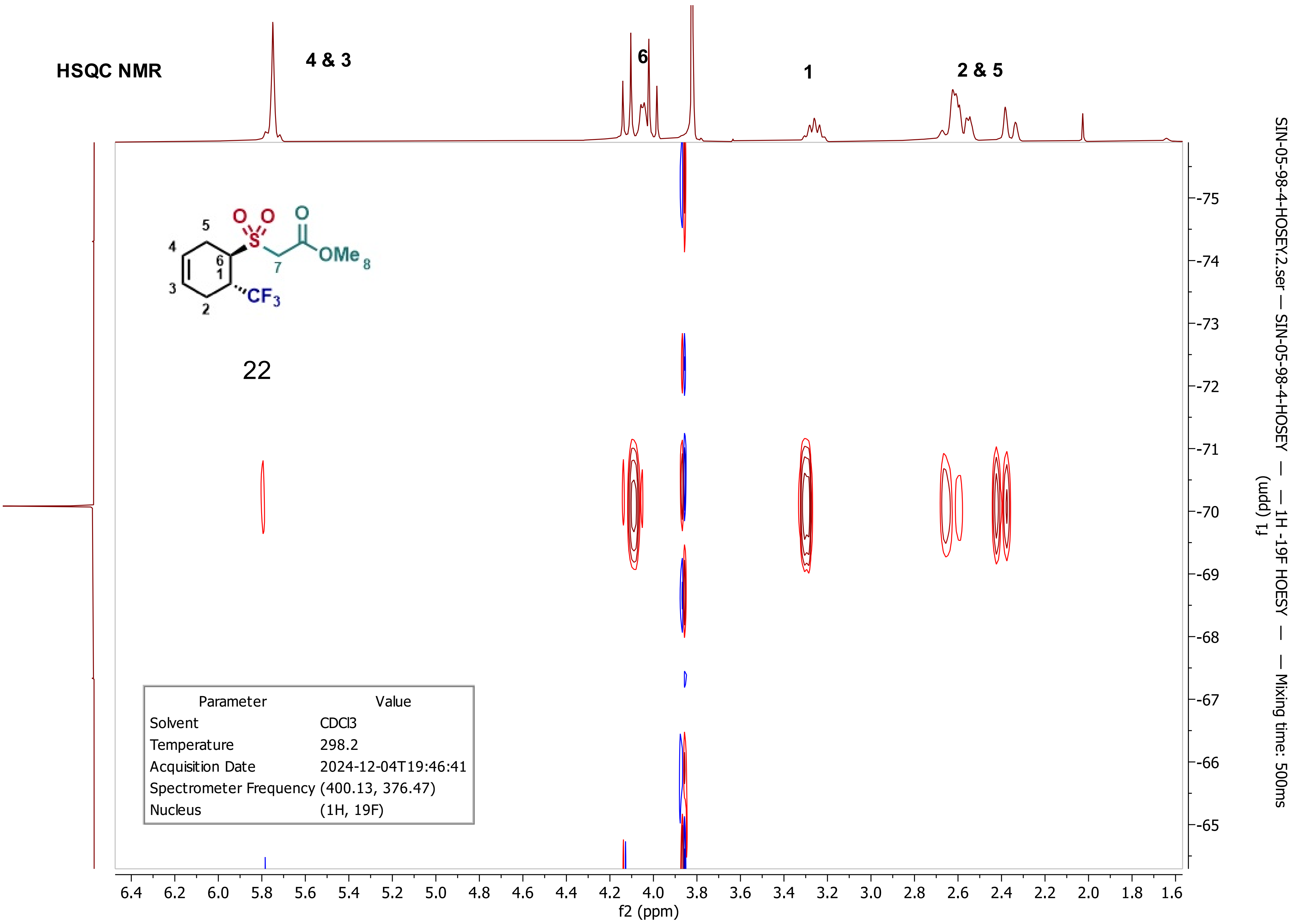

Parameter	Value
Solvent	CDCl3
Temperature	298.2
Acquisition Date	2024-12-04T19:46:41
Spectrometer Frequency	(400.13, 376.47)
Nucleus	(1H, 19F)

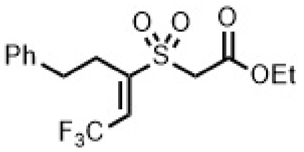

23

Parameter	Value
Solvent	CDCl3
Temperature	298.0
Acquisition Date	2024-03-04T12:14:57
Spectrometer Frequency	400.13
Nucleus	1H

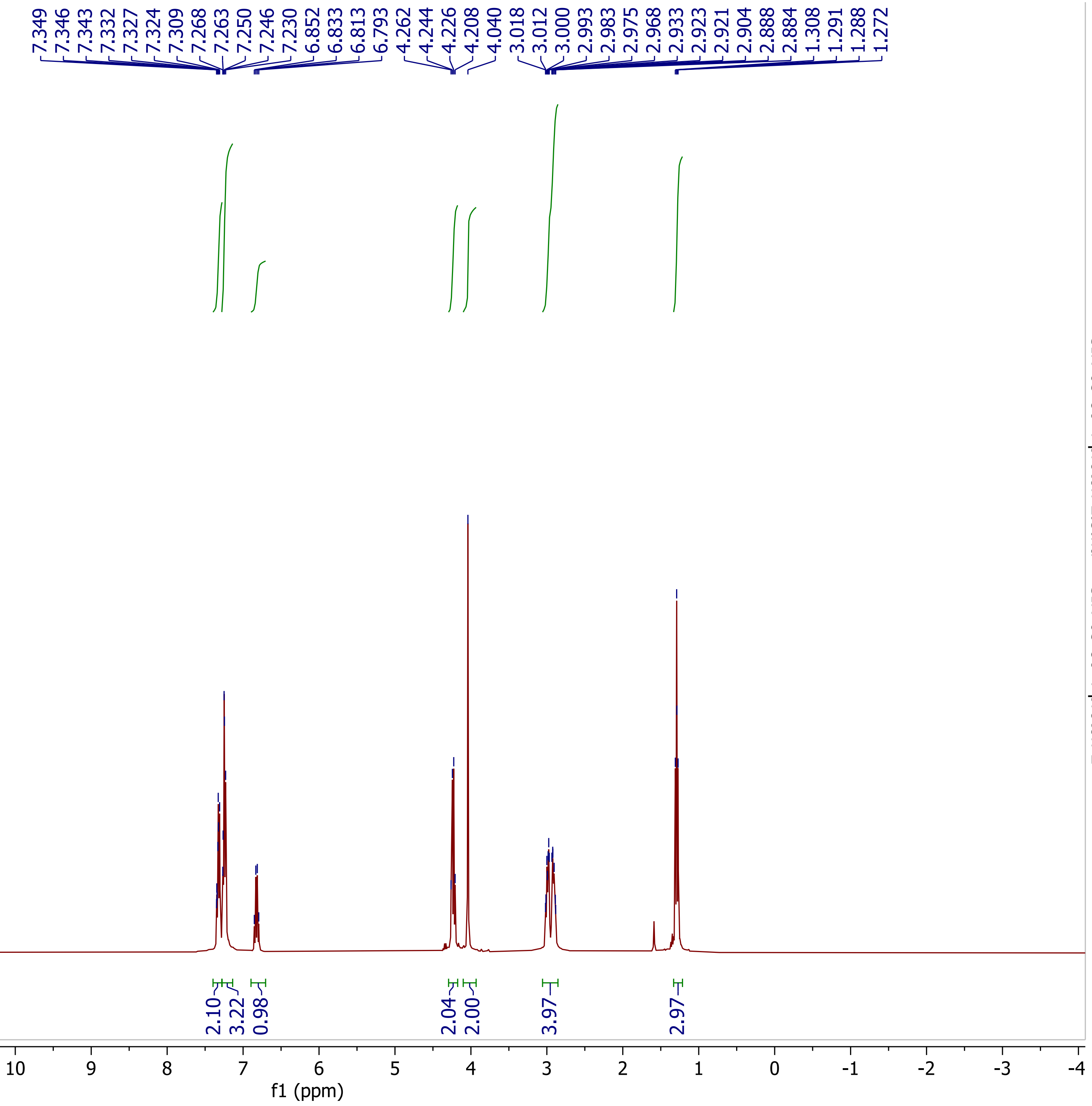

SIN-05-86-4-proton-2.3.fid — SIN-05-86-4-proton-2

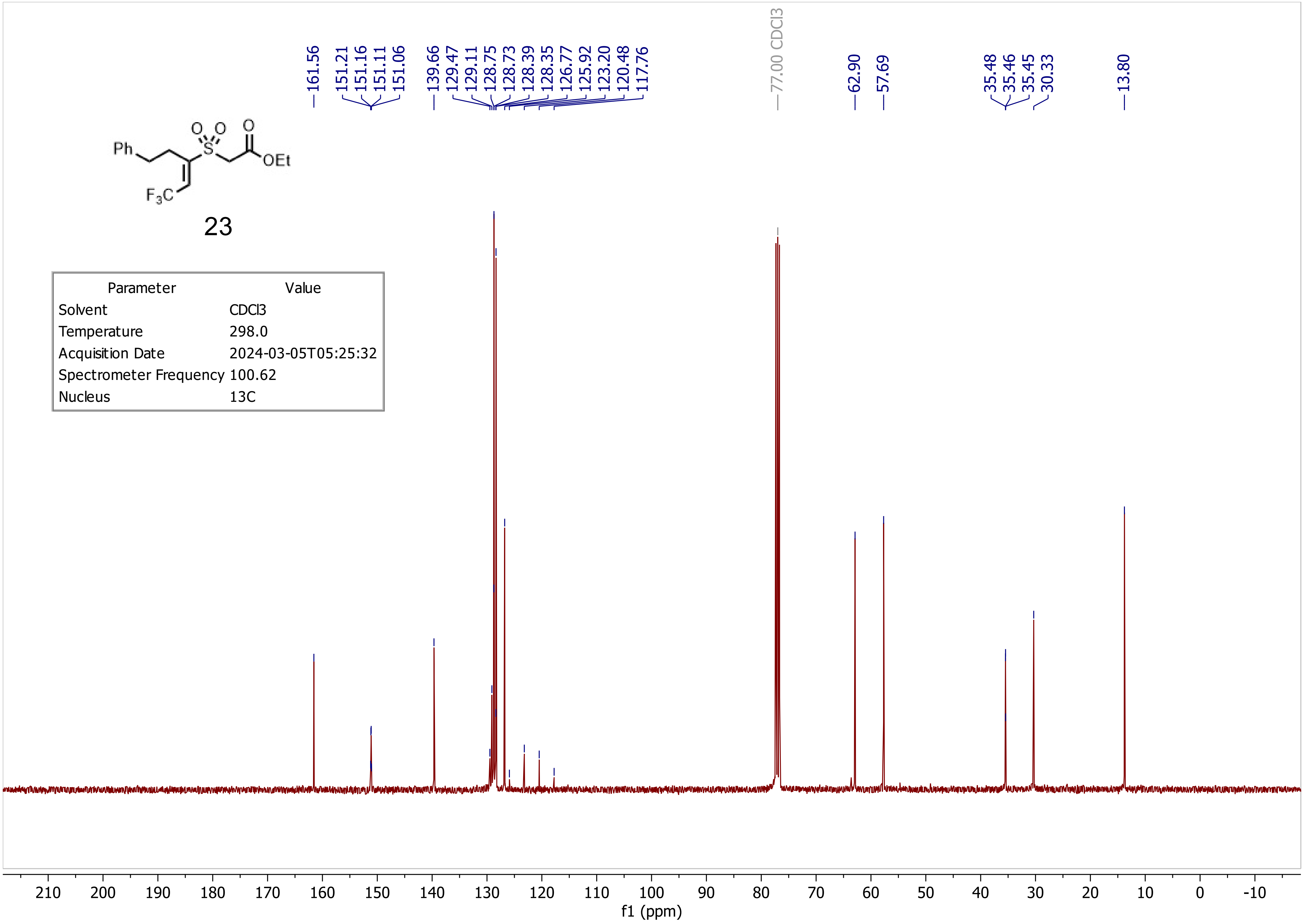

Parameter	Value
Solvent	CDCl3
Temperature	298.0
Acquisition Date	2024-03-05T05:25:32
Spectrometer Frequency	100.62
Nucleus	13C

SIN-05-86-4-TT-17-C13.1.fid — SIN-05-98-3-TT-24-R

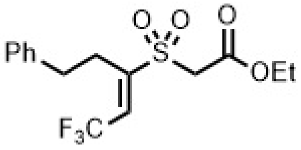

23

Parameter	Value
Solvent	CDCl3
Temperature	298.0
Acquisition Date	2024-03-04T12:19:21
Spectrometer Frequency	376.46
Nucleus	19F

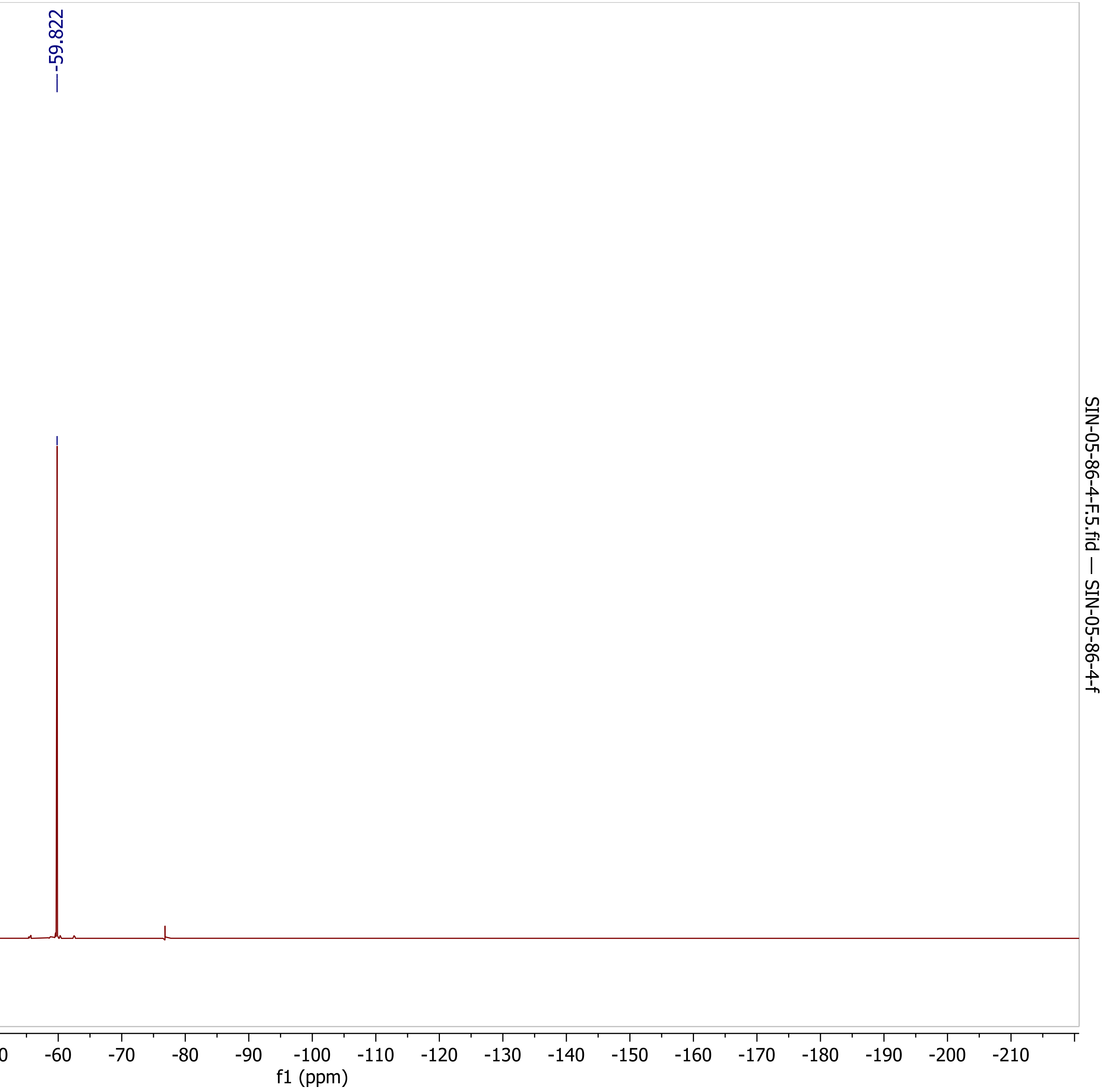

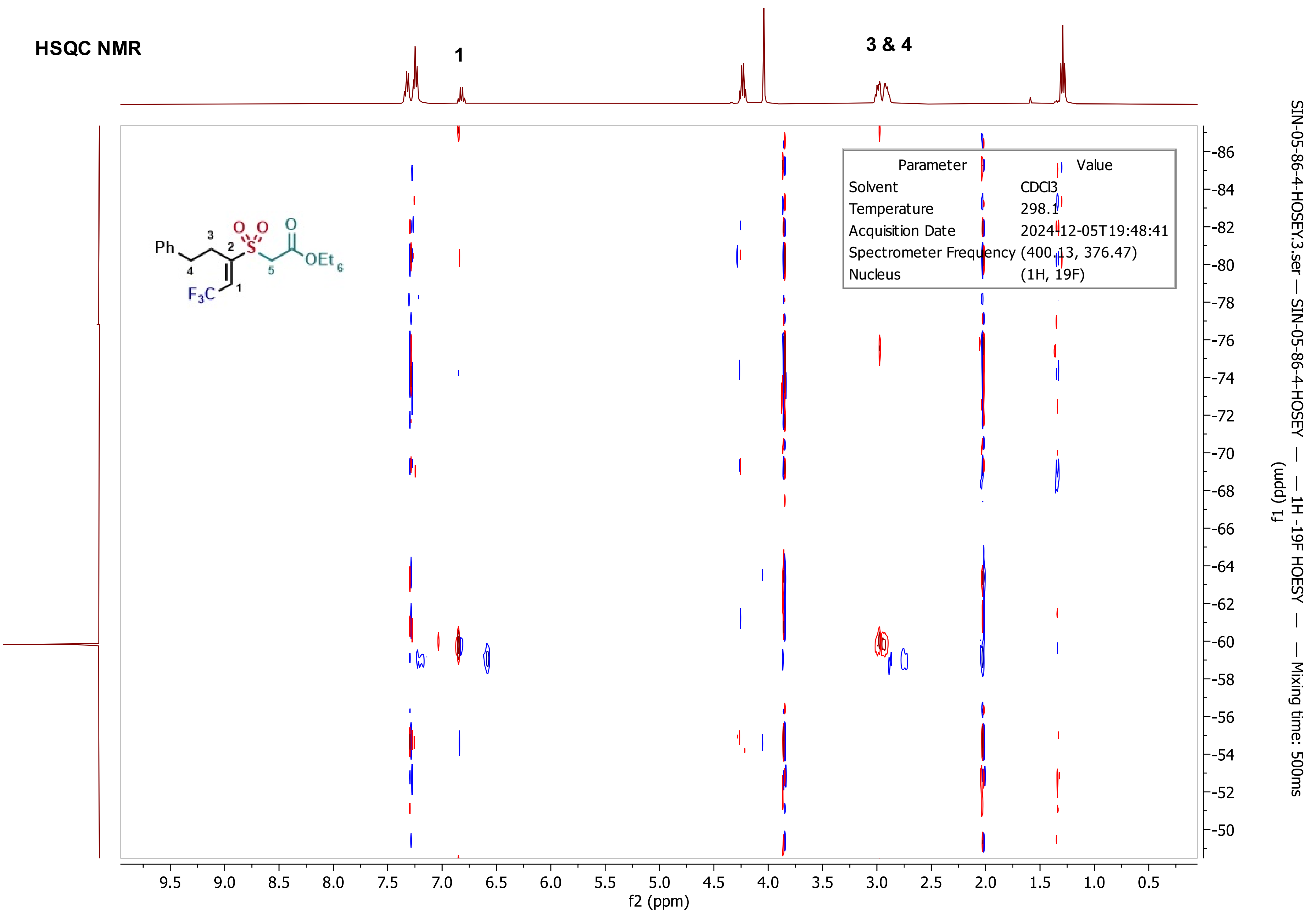

HSQC NMR
1
3 & 4
Ph
3
2
4
5
1
O
O
O
S
OEt
6
F3C
Parameter
Value
Solvent
CDCl3
Temperature
298.1
Acquisition Date
2024-12-05T19:48:41
Spectrometer Frequency
(400.13, 376.47)
Nucleus
(1H, 19F)
f2 (ppm)
f1 (ppm)
SIN-05-86-4-HOSEY.3.ser — SIN-05-86-4-HOSEY — — 1H -19F HOESY — — Mixing time: 500ms

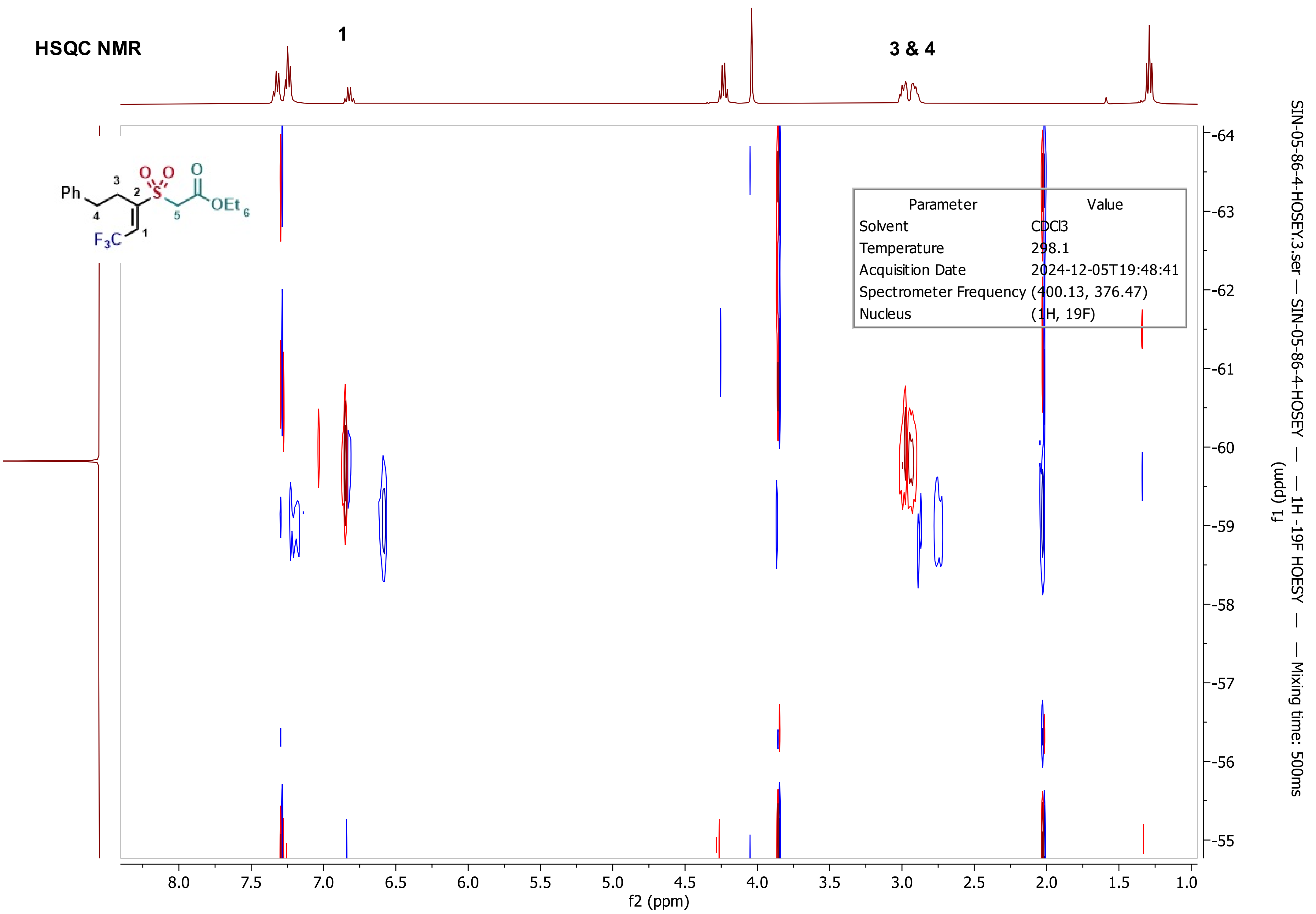
HSQC NMR
1
3 & 4
Ph
F_3C
OEt
Parameter Value
Solvent CDCl3
Temperature 298.1
Acquisition Date 2024-12-05T19:48:41
Spectrometer Frequency (400.13, 376.47)
Nucleus (1H, 19F)
f2 (ppm)
f1 (ppm)
SIN-05-86-4-HOSEY.3.ser — SIN-05-86-4-HOSEY — — 1H -19F HOESY — — Mixing time: 500ms

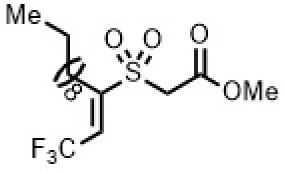

24

Parameter	Value
Solvent	CDCl3
Temperature	298.0
Acquisition Date	2024-04-23T00:24:14
Spectrometer Frequency	400.13
Nucleus	1H

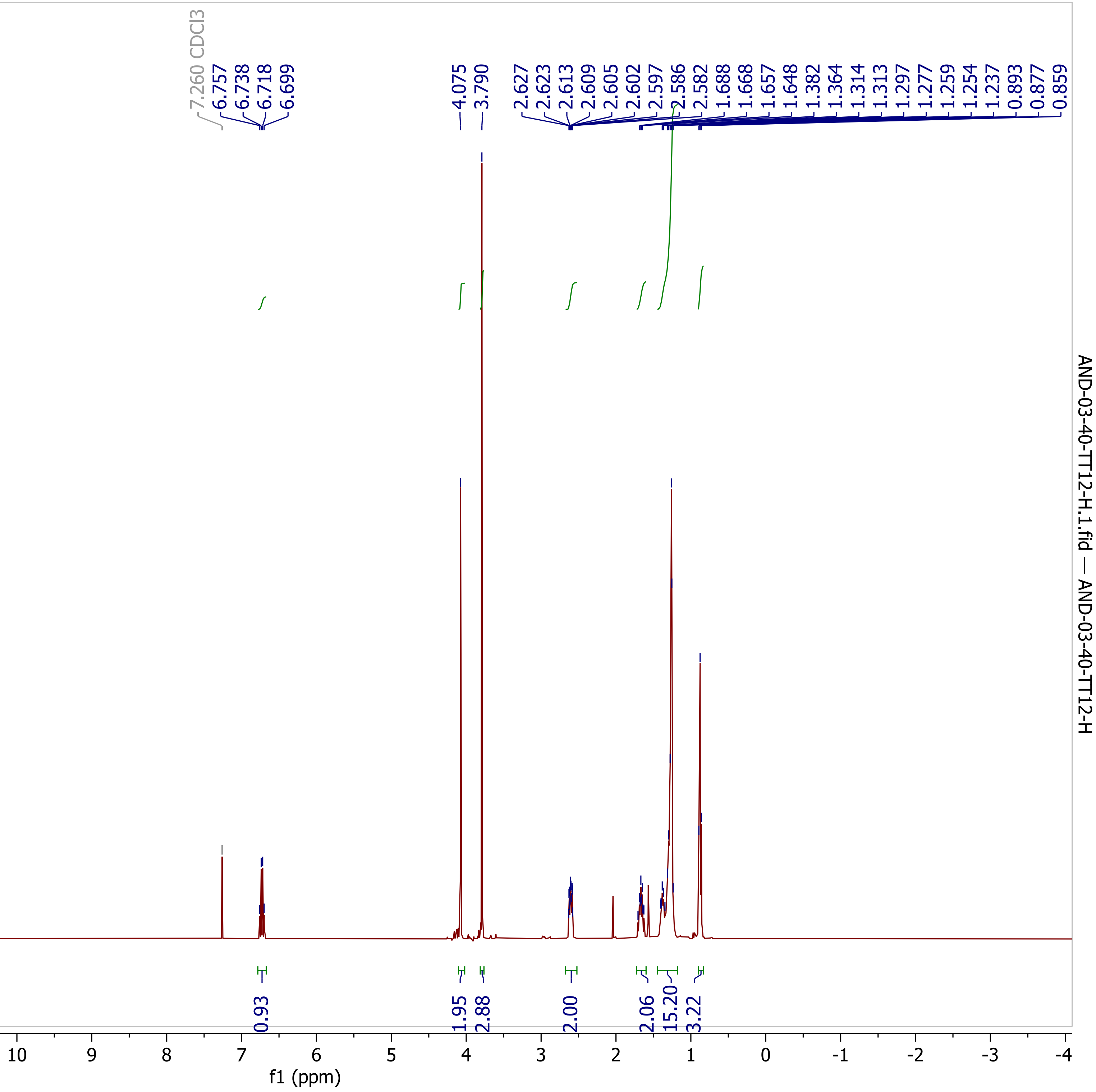

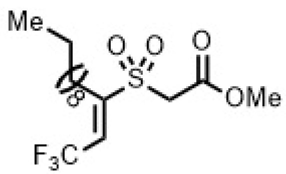

24

Parameter	Value
Solvent	CDCl3
Temperature	298.0
Acquisition Date	2024-04-23T01:24:07
Spectrometer Frequency	100.62
Nucleus	13C

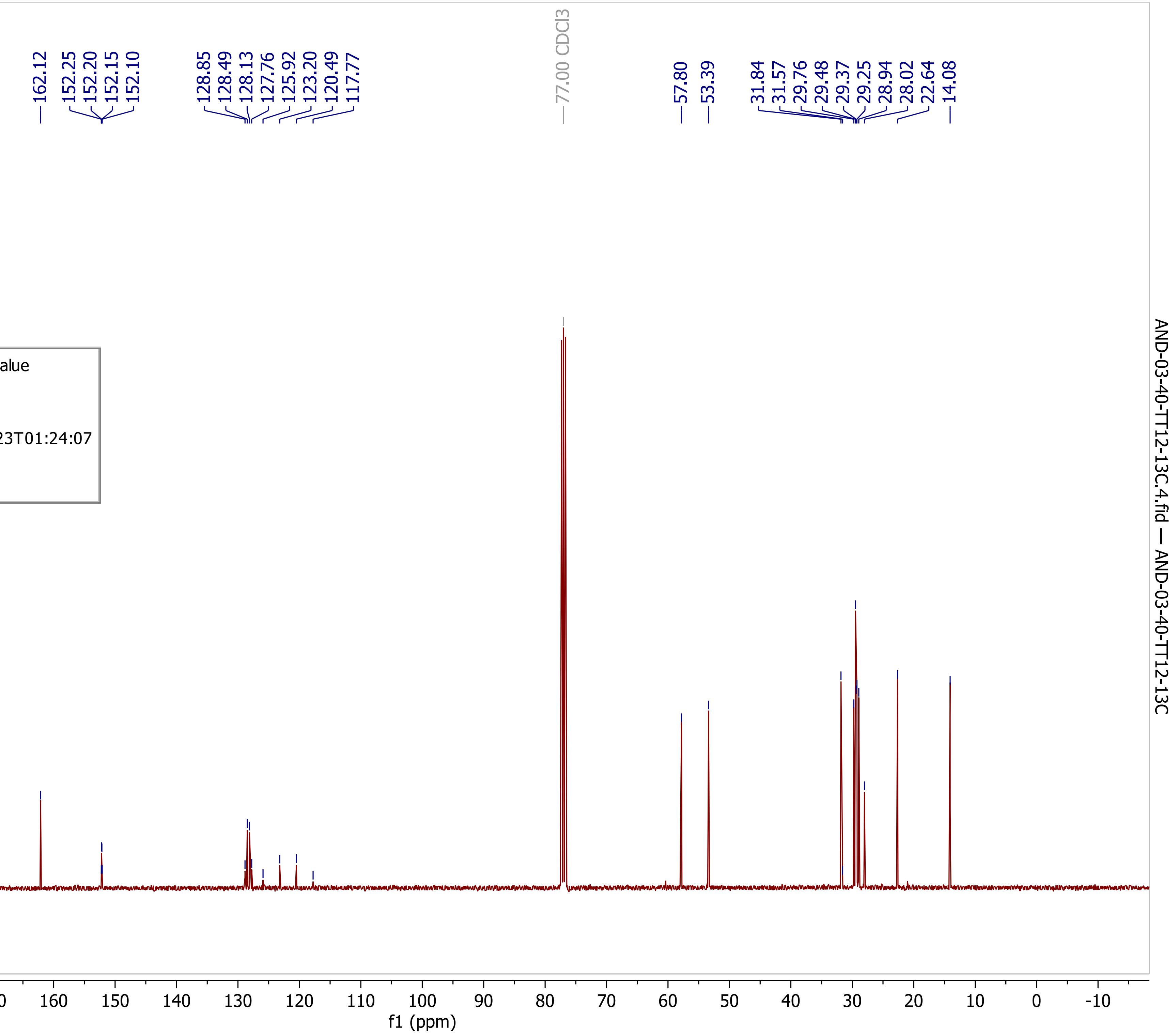

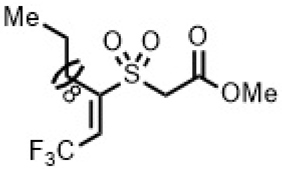

24

Parameter	Value
Solvent	CDCl3
Temperature	298.0
Acquisition Date	2024-04-23T01:28:20
Spectrometer Frequency	376.46
Nucleus	19F

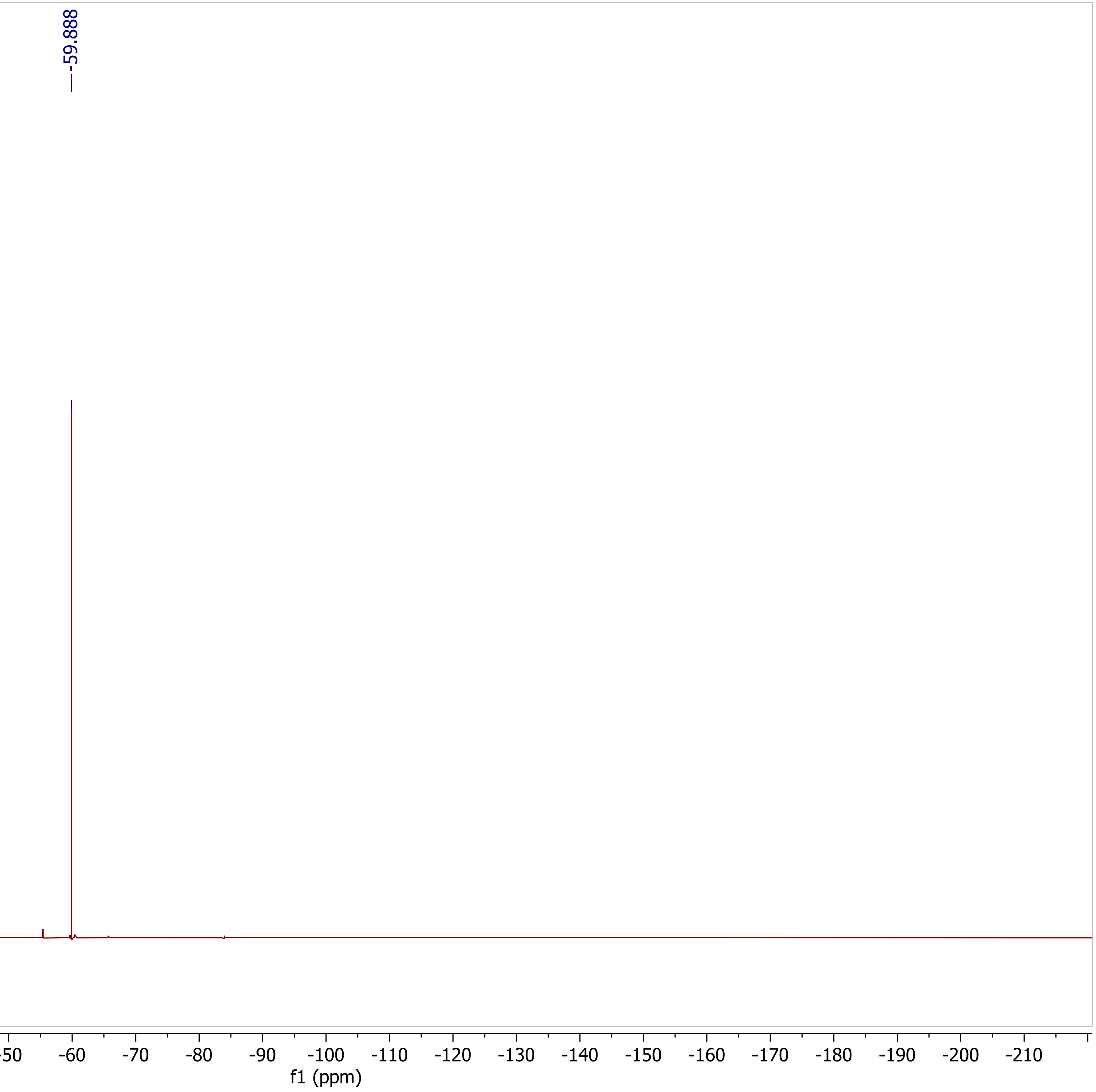

AND-03-40-TT12-19F.5.fid — AND-03-40-TT12-19F

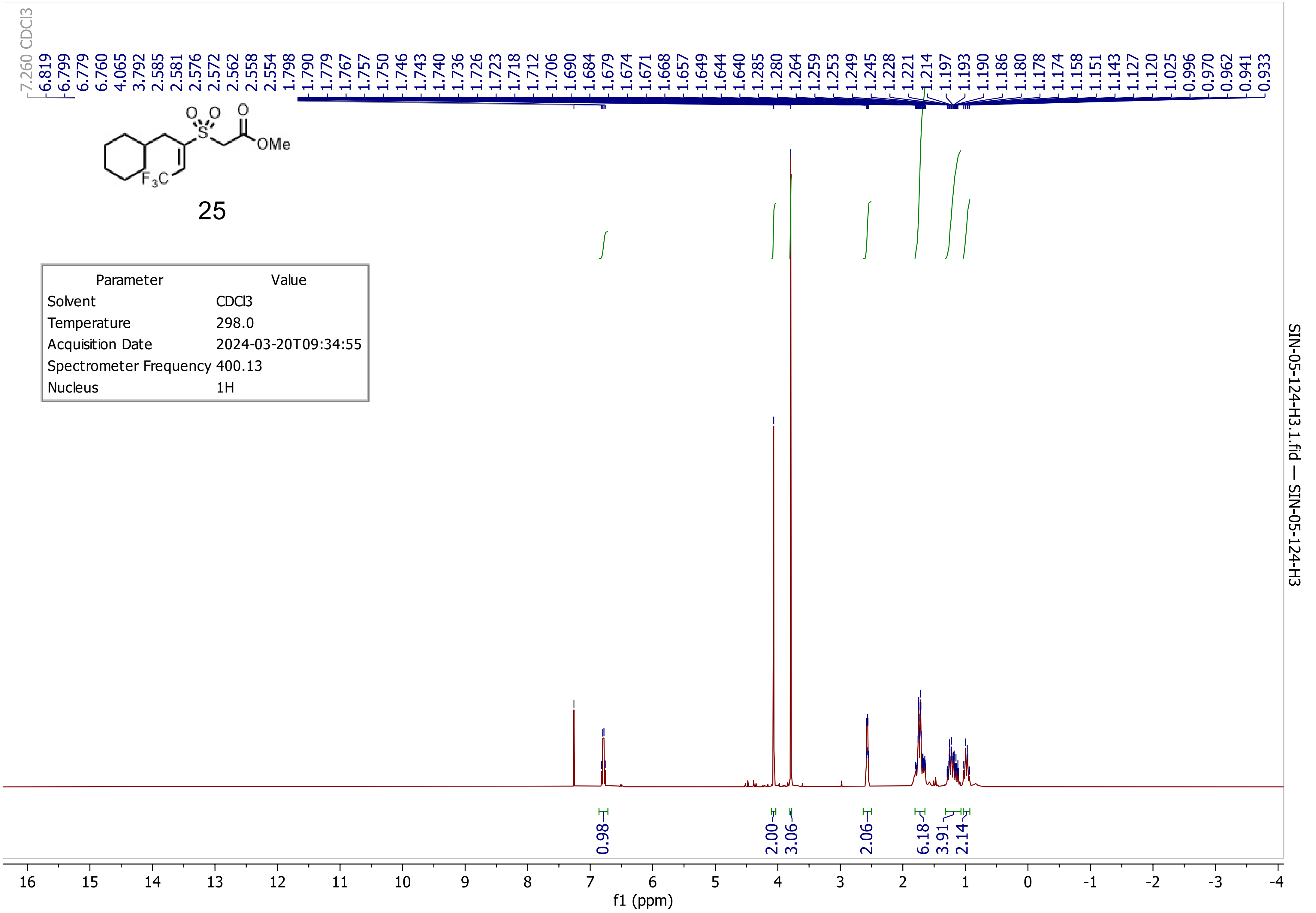

Parameter	Value
Solvent	CDCl3
Temperature	298.0
Acquisition Date	2024-03-20T09:34:55
Spectrometer Frequency	400.13
Nucleus	1H

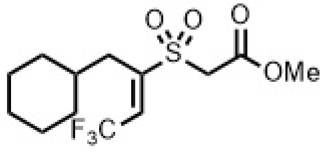

25

Parameter	Value
Solvent	CDCl3
Temperature	298.0
Acquisition Date	2024-03-20T09:57:26
Spectrometer Frequency	100.62
Nucleus	13C

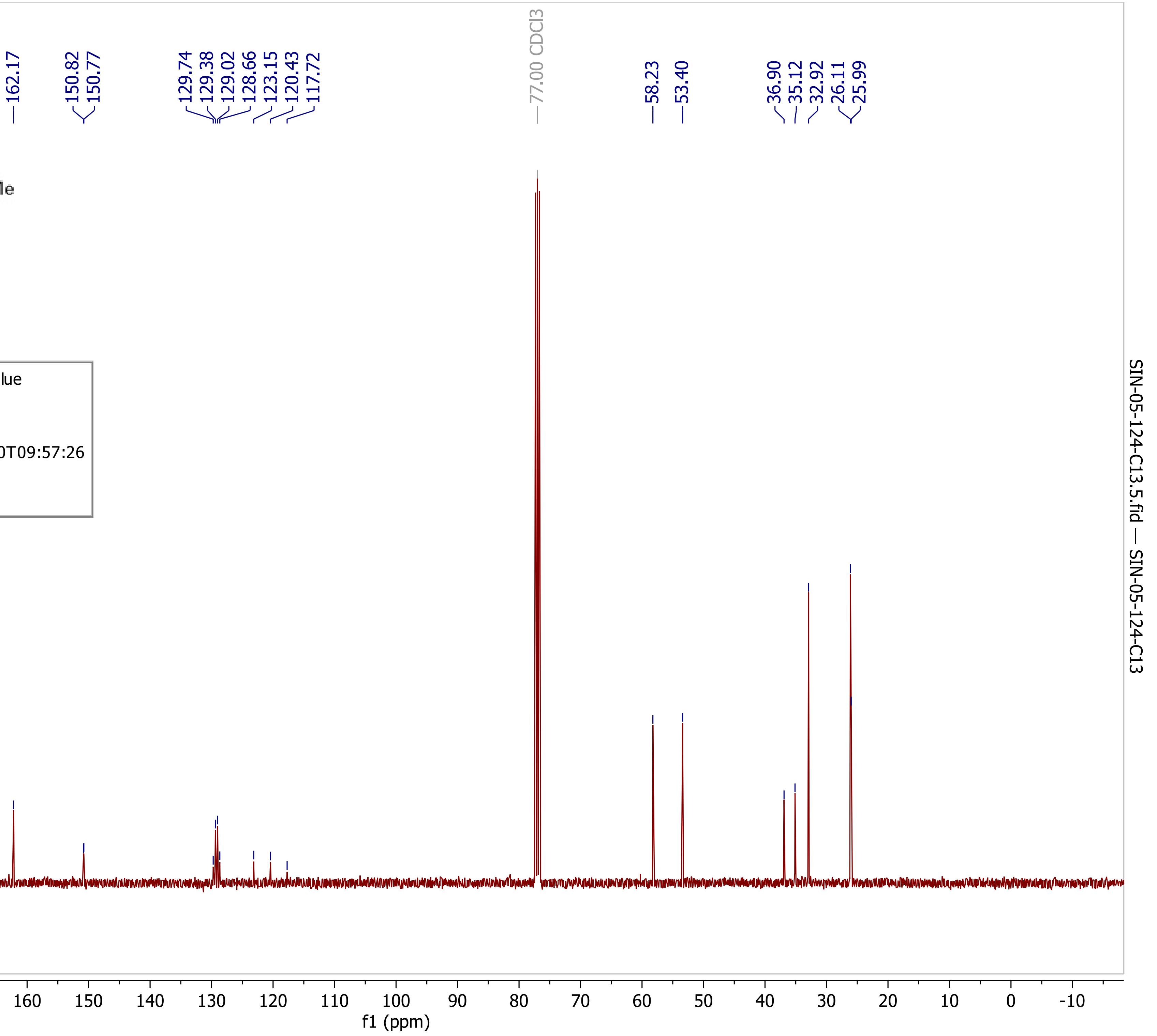

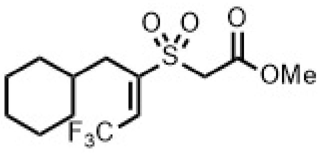

25

Parameter	Value
Solvent	CDCl3
Temperature	298.0
Acquisition Date	2024-03-20T09:38:43
Spectrometer Frequency	376.46
Nucleus	19F

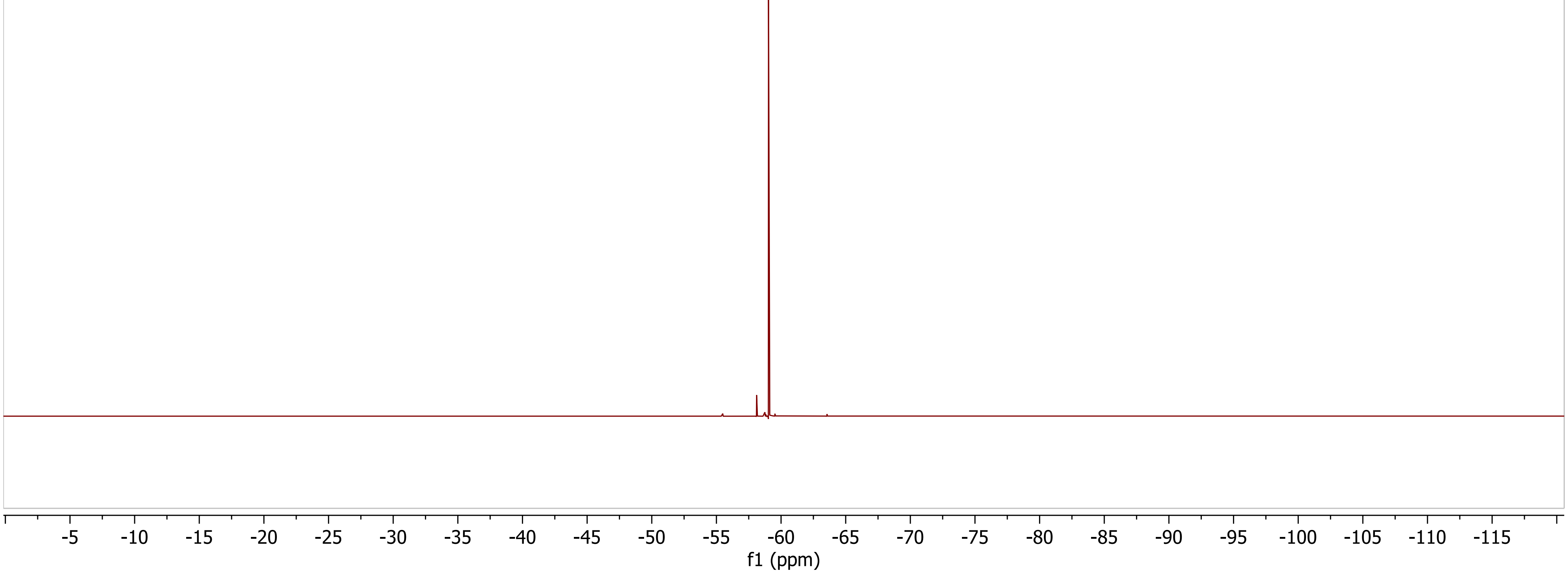

SIN-05-124-F3.4.fid — SIN-05-124-F3

HSQC NMR

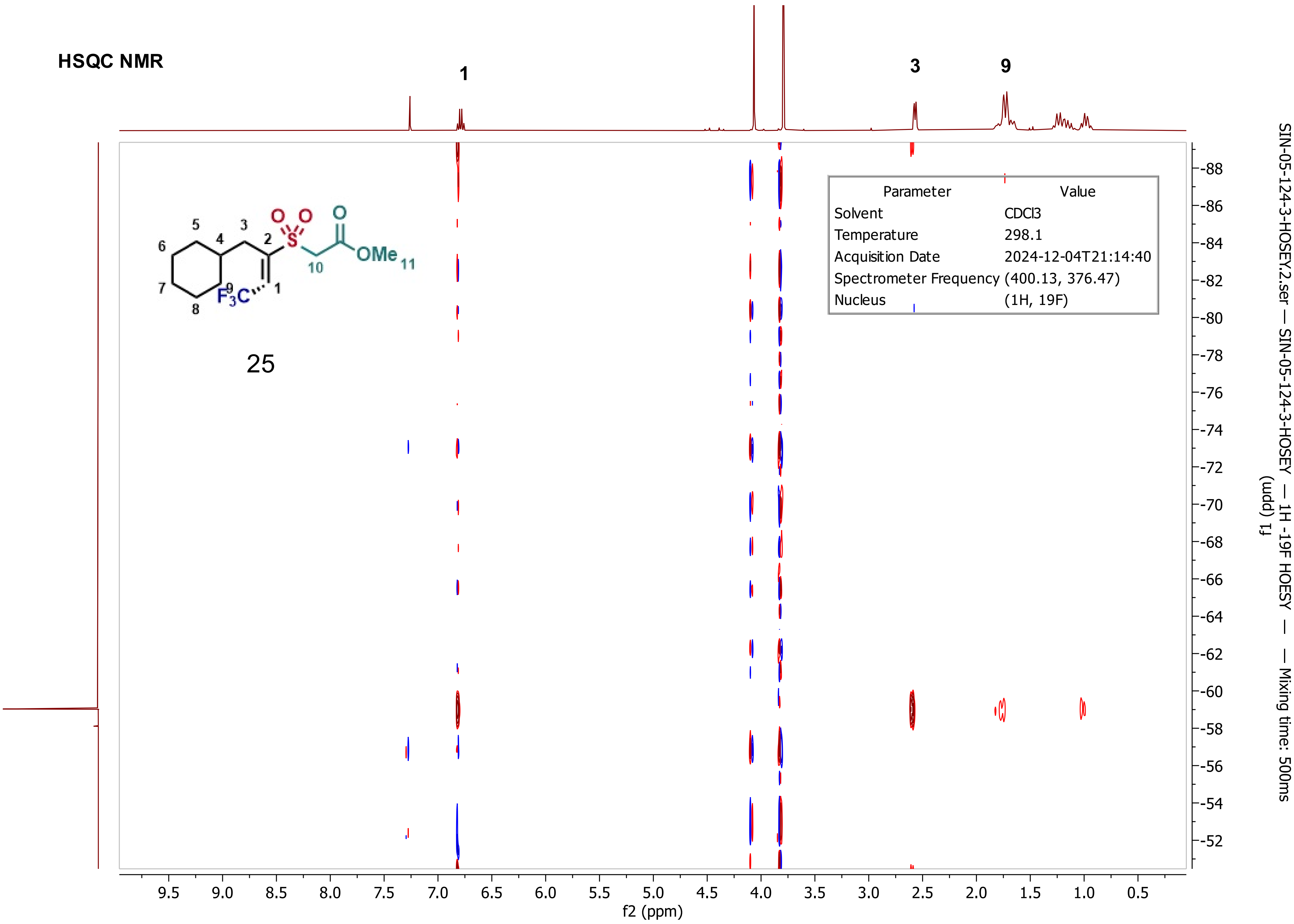

1
3
9
25
OMe
11
10
F3C
Parameter | Value
Solvent | CDCl3
Temperature | 298.1
Acquisition Date | 2024-12-04T21:14:40
Spectrometer Frequency | (400.13, 376.47)
Nucleus | (1H, 19F)
f2 (ppm)
f1 (ppm)
SIN-05-124-3-HOSEY.2.ser — SIN-05-124-3-HOSEY — 1H -19F HOESY — — Mixing time: 500ms

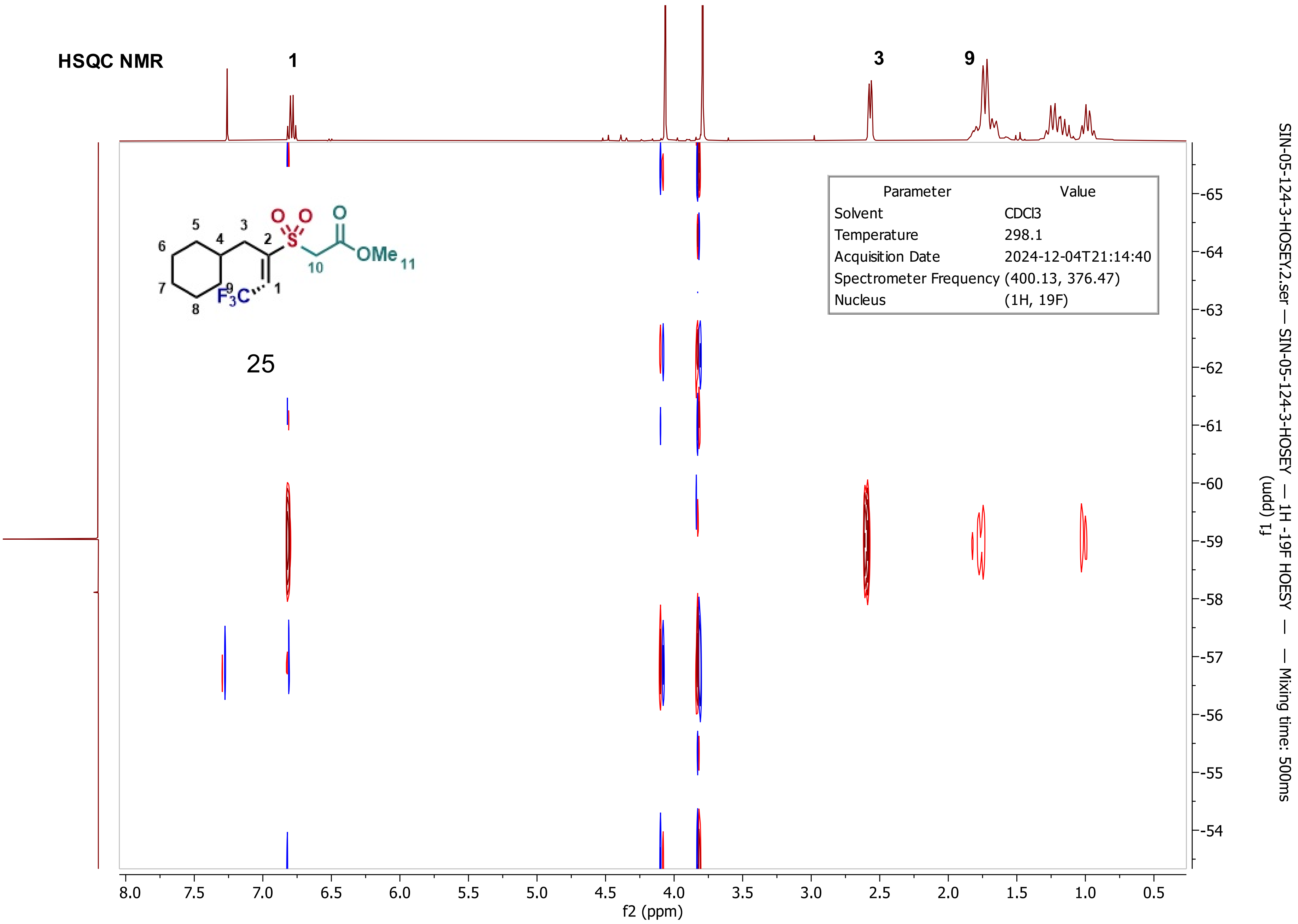

HSQC NMR
1
3
9
5
3
6
4
2
O
O
O
S
OMe
11
10
7
9
F3C
1
8
25
Parameter
Value
Solvent
CDCl3
Temperature
298.1
Acquisition Date
2024-12-04T21:14:40
Spectrometer Frequency
(400.13, 376.47)
Nucleus
(1H, 19F)
f2 (ppm)
f1 (ppm)
SIN-05-124-3-HOSEY.2.ser — SIN-05-124-3-HOSEY — 1H -19F HOESY — — Mixing time: 500ms

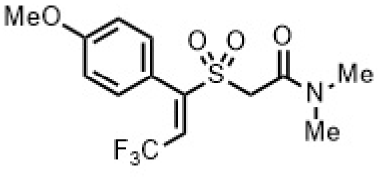

26

Parameter	Value
Solvent	CDCl3
Temperature	298.0
Acquisition Date	2024-09-17T18:03:46
Spectrometer Frequency	400.13
Nucleus	1H

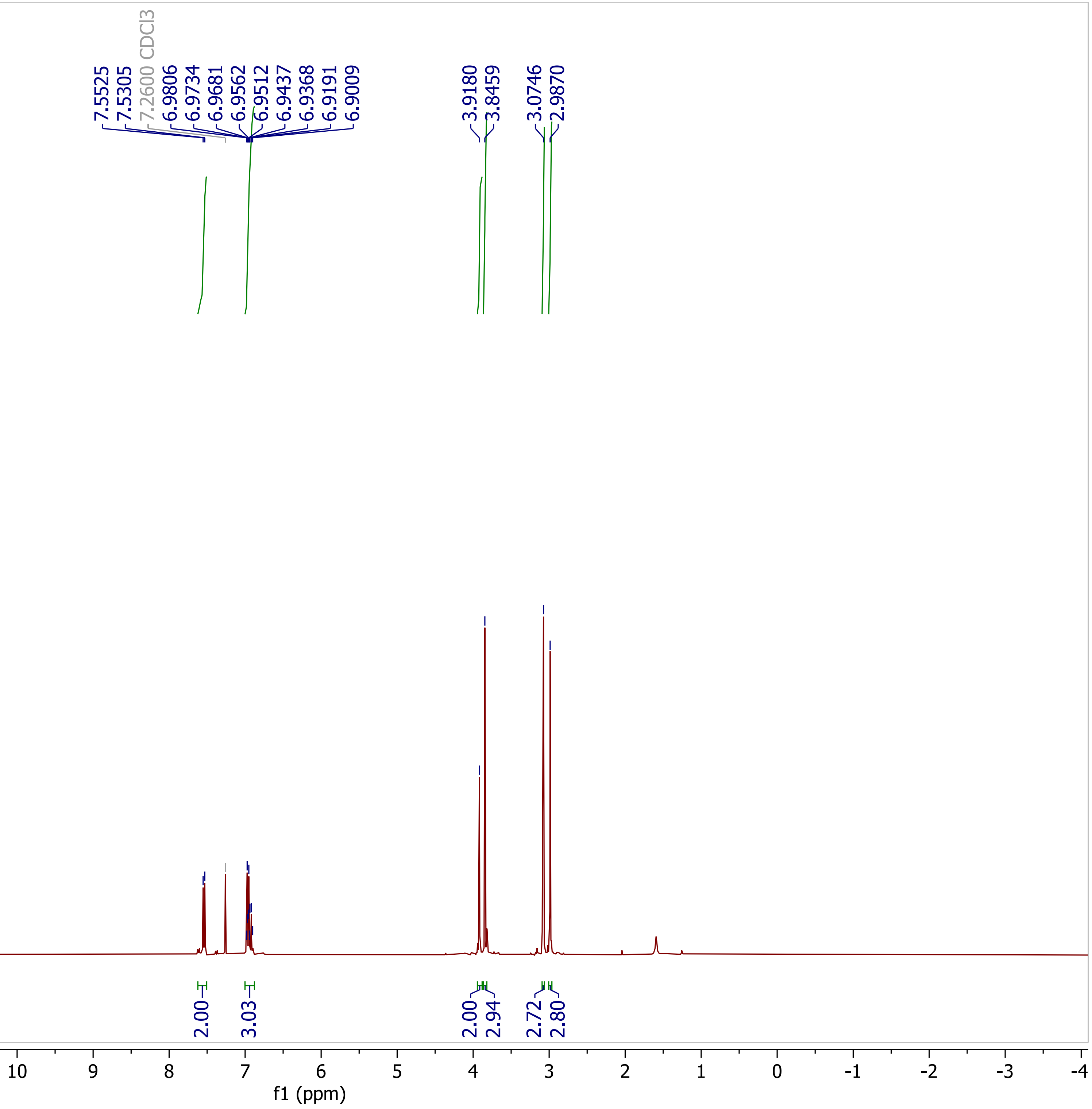

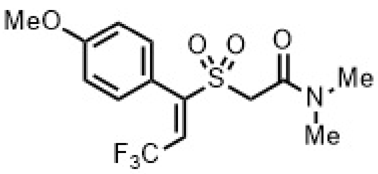

26

Parameter	Value
Solvent	CDCl3
Temperature	298.0
Acquisition Date	2024-11-30T17:45:39
Spectrometer Frequency	100.62
Nucleus	13C

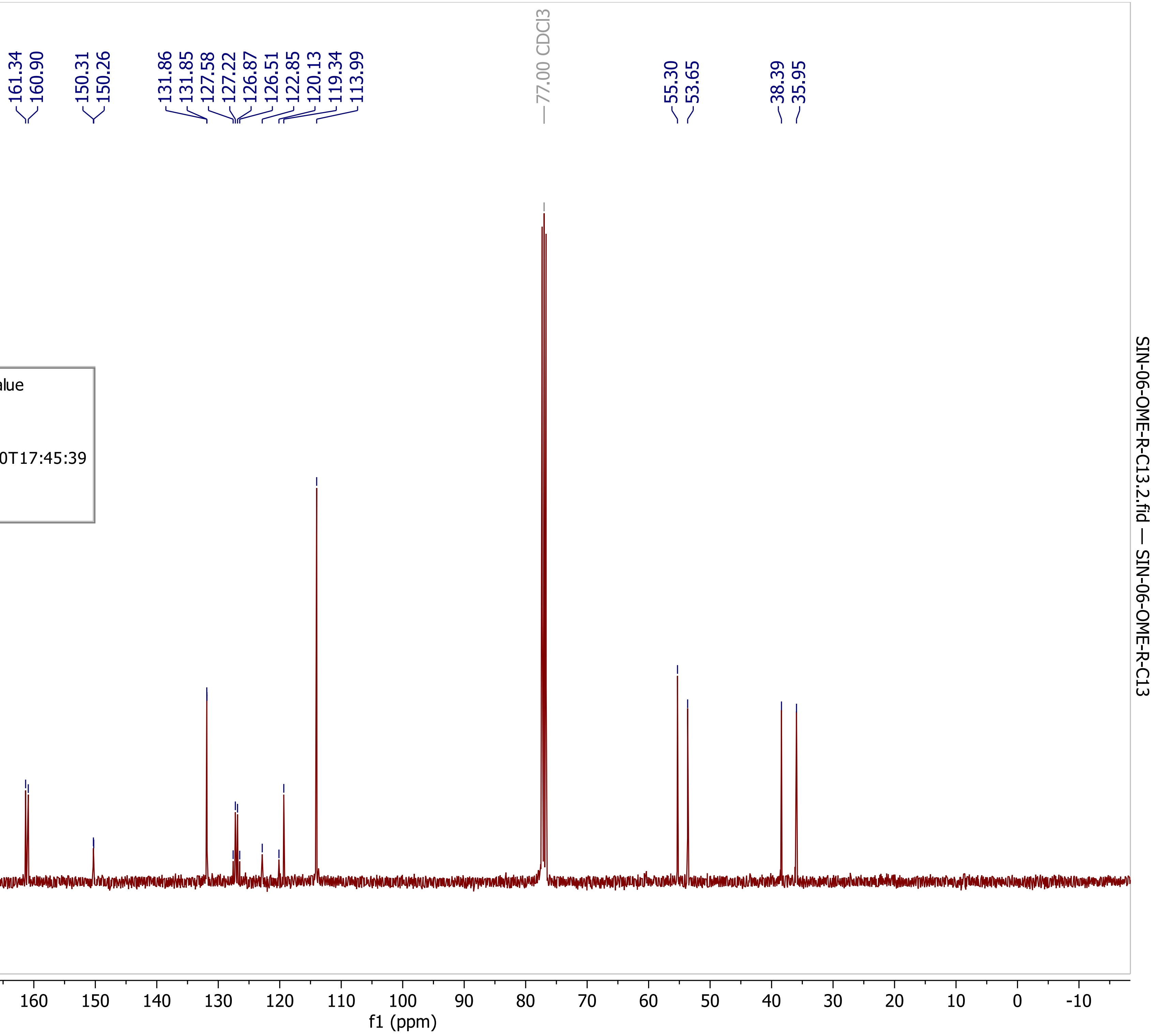

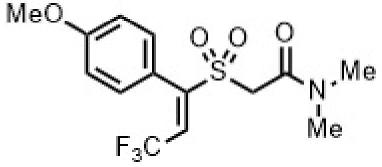

26

Parameter	Value
Solvent	CDCl3
Temperature	299.5
Acquisition Date	2024-09-18T01:16:41
Spectrometer Frequency	376.46
Nucleus	19F

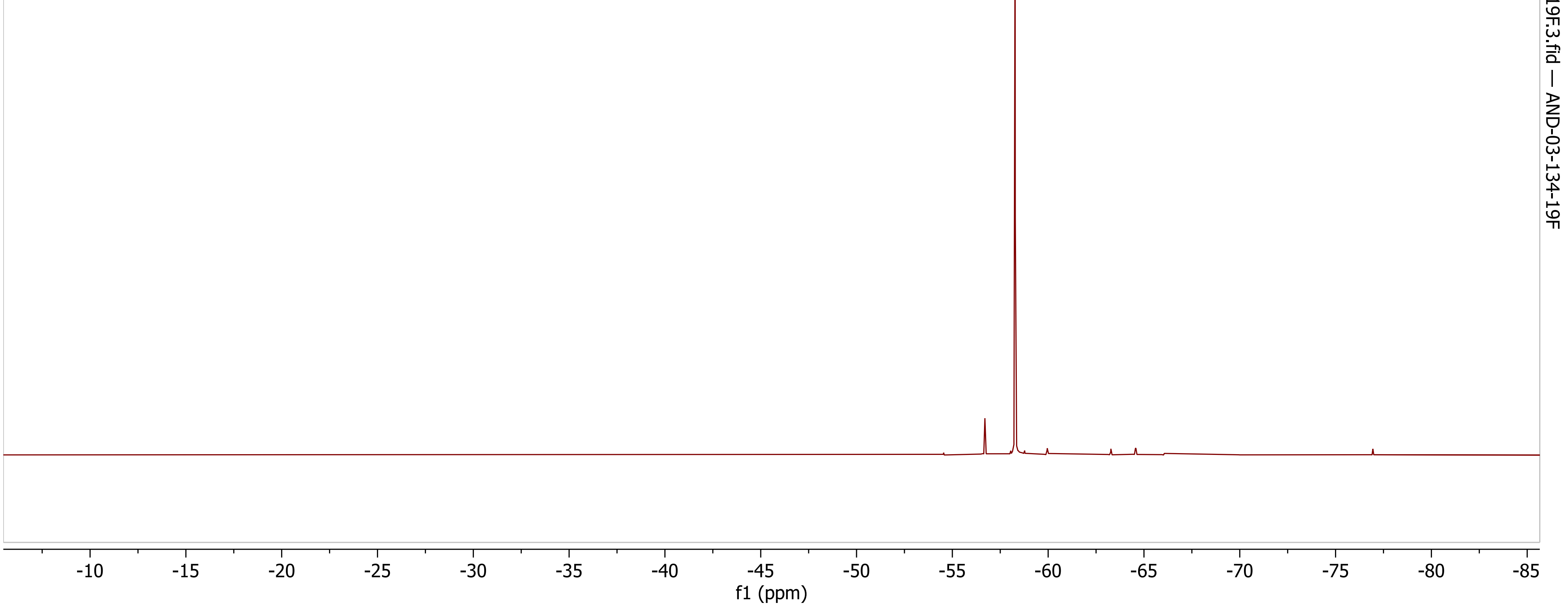

HSQC NMR

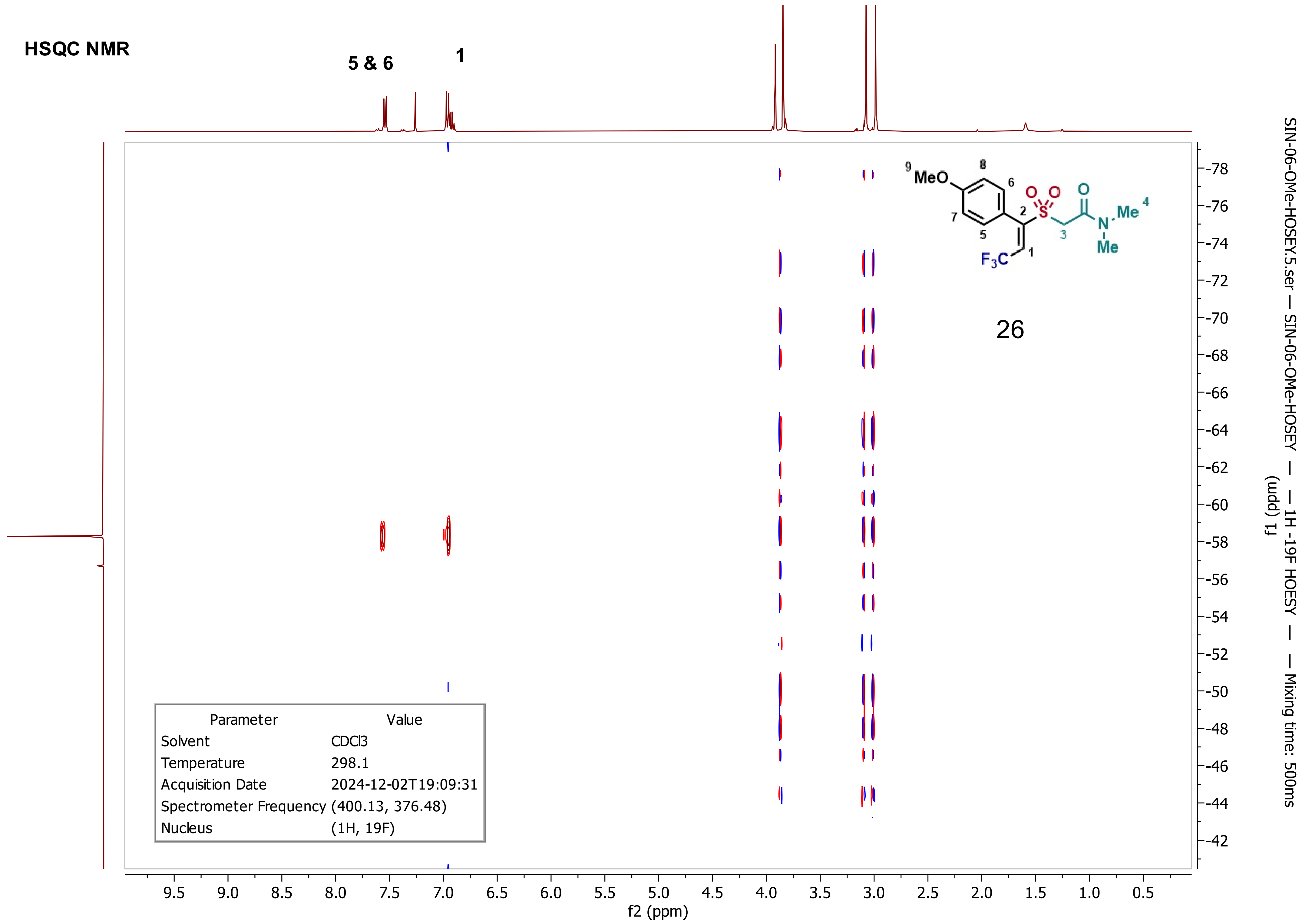

5 & 6
1
MeO
26
f1 (ppm)
f2 (ppm)
SIN-06-OMe-HOSEY.5.ser — SIN-06-OMe-HOSEY — — 1H -19F HOESY — — Mixing time: 500ms
Parameter | Value
Solvent | CDCl3
Temperature | 298.1
Acquisition Date | 2024-12-02T19:09:31
Spectrometer Frequency | (400.13, 376.48)
Nucleus | (1H, 19F)

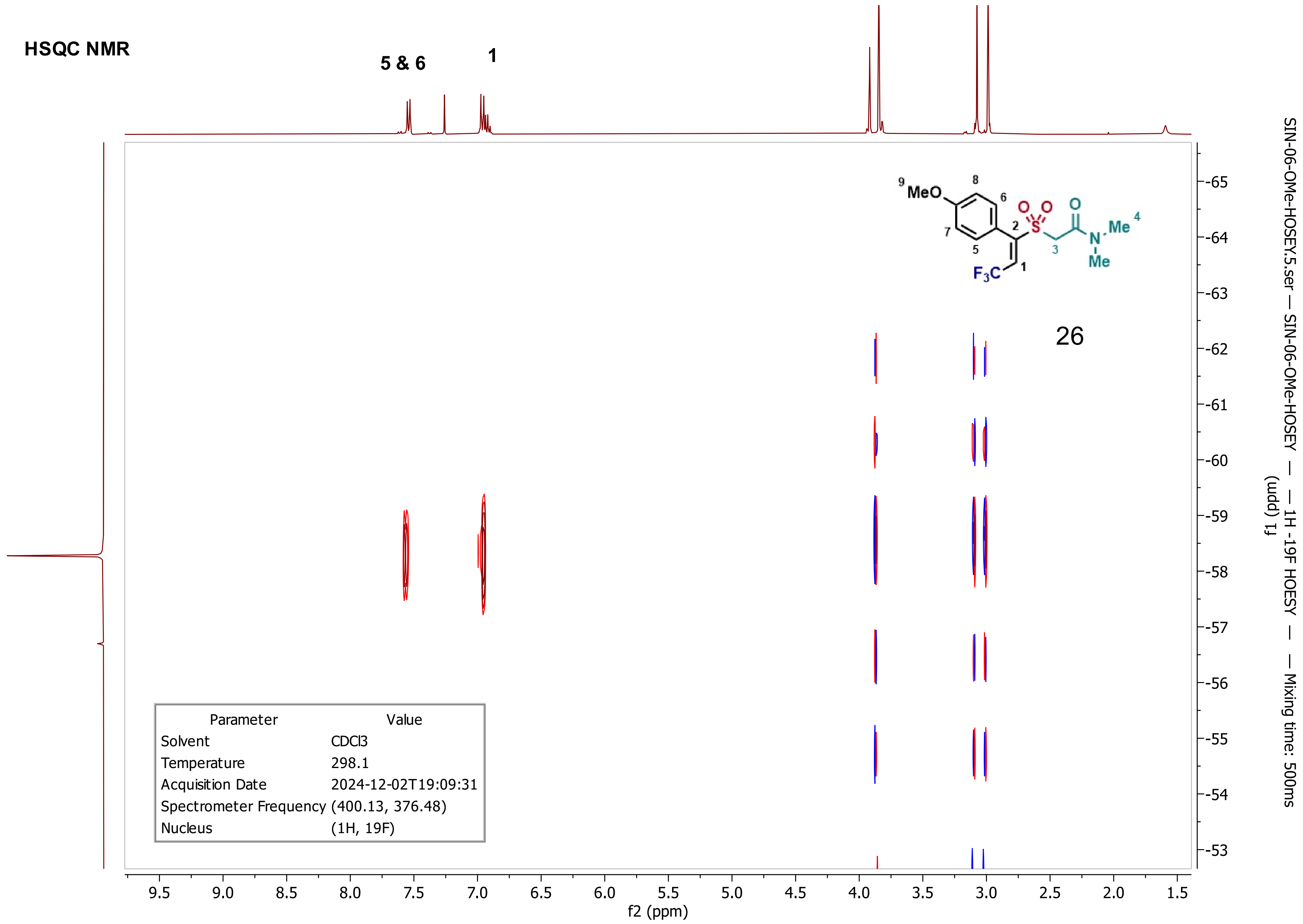

Parameter	Value
Solvent	CDCl3
Temperature	298.1
Acquisition Date	2024-12-02T19:09:31
Spectrometer Frequency	(400.13, 376.48)
Nucleus	(1H, 19F)

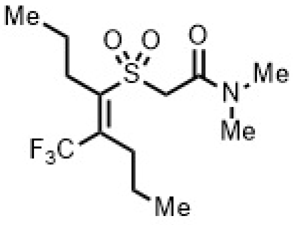

27

Parameter	Value
Solvent	CDCl3
Temperature	298.0
Acquisition Date	2024-09-18T21:34:54
Spectrometer Frequency	400.13
Nucleus	1H

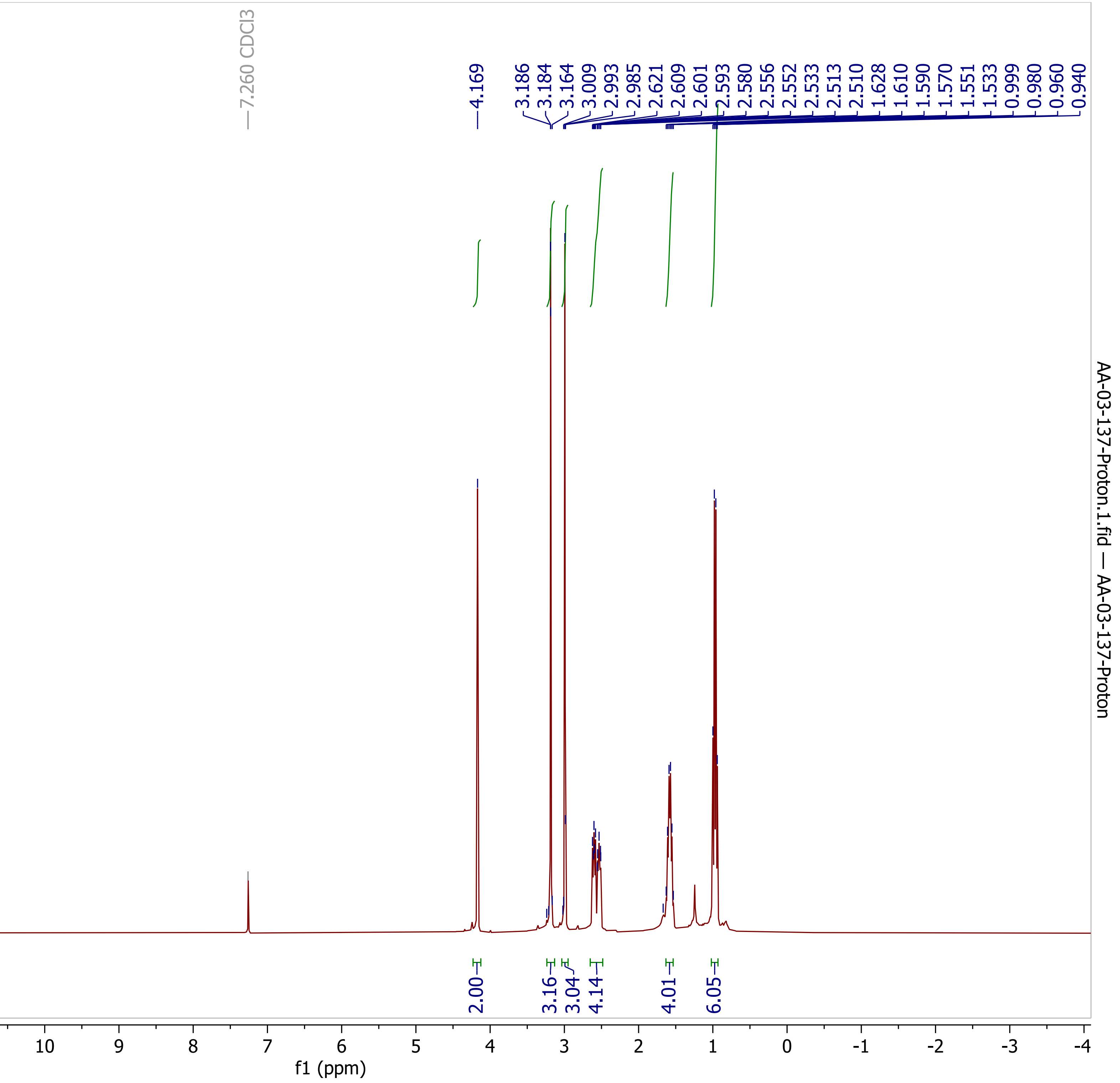

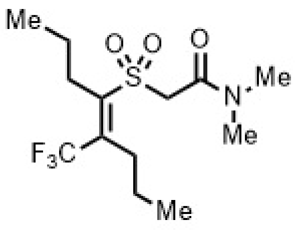

27

Parameter	Value
Solvent	CDCl3
Temperature	298.0
Acquisition Date	2024-09-19T00:22:32
Spectrometer Frequency	100.62
Nucleus	13C

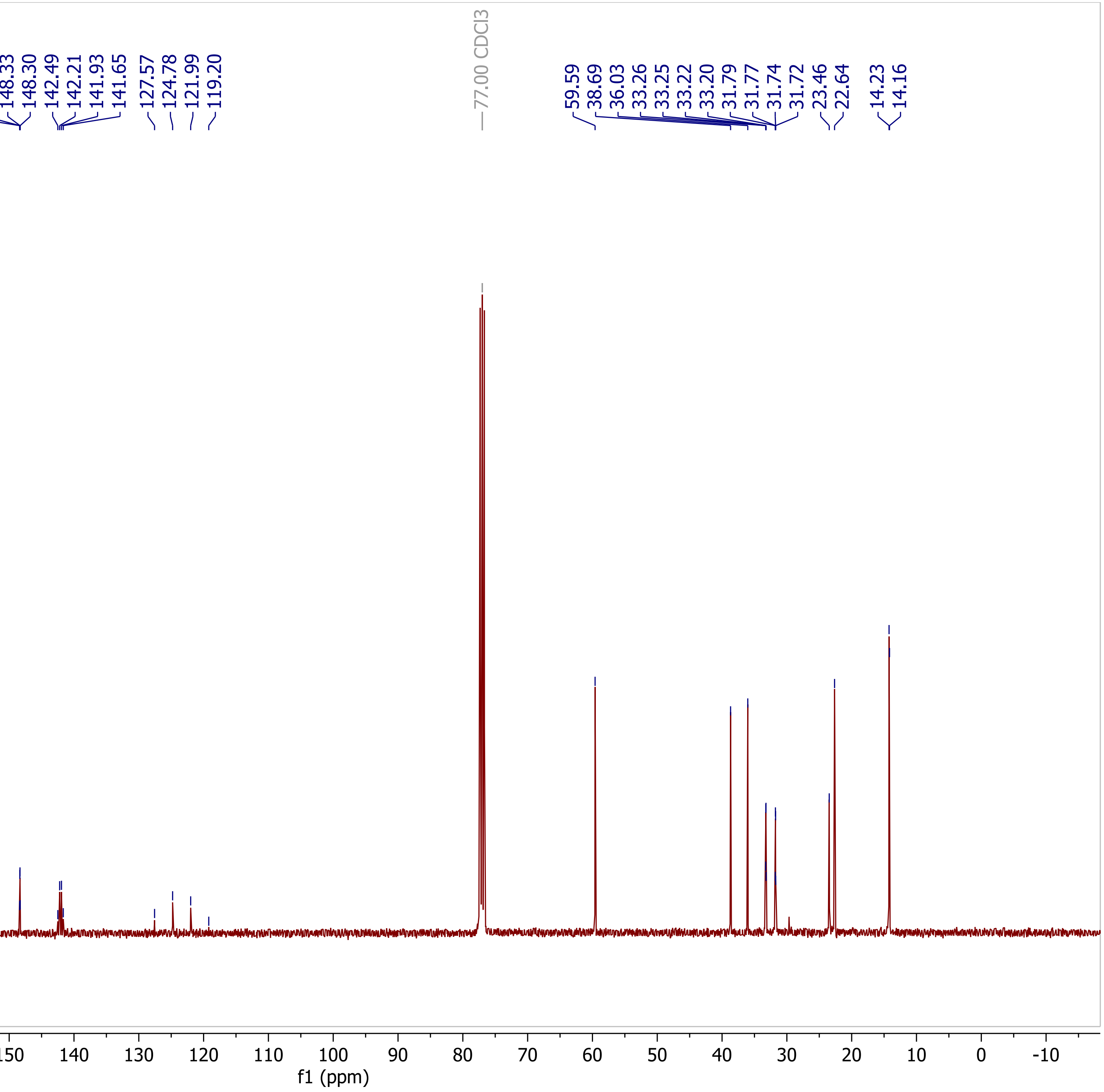

AA-03-137-Carbon.1.fid — AA-03-137-Carbon

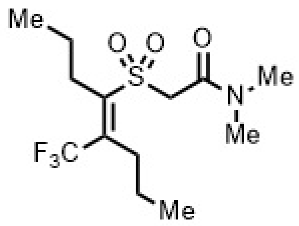

27

Parameter	Value
Solvent	CDCl3
Temperature	298.0
Acquisition Date	2024-09-19T00:27:01
Spectrometer Frequency	376.46
Nucleus	19F

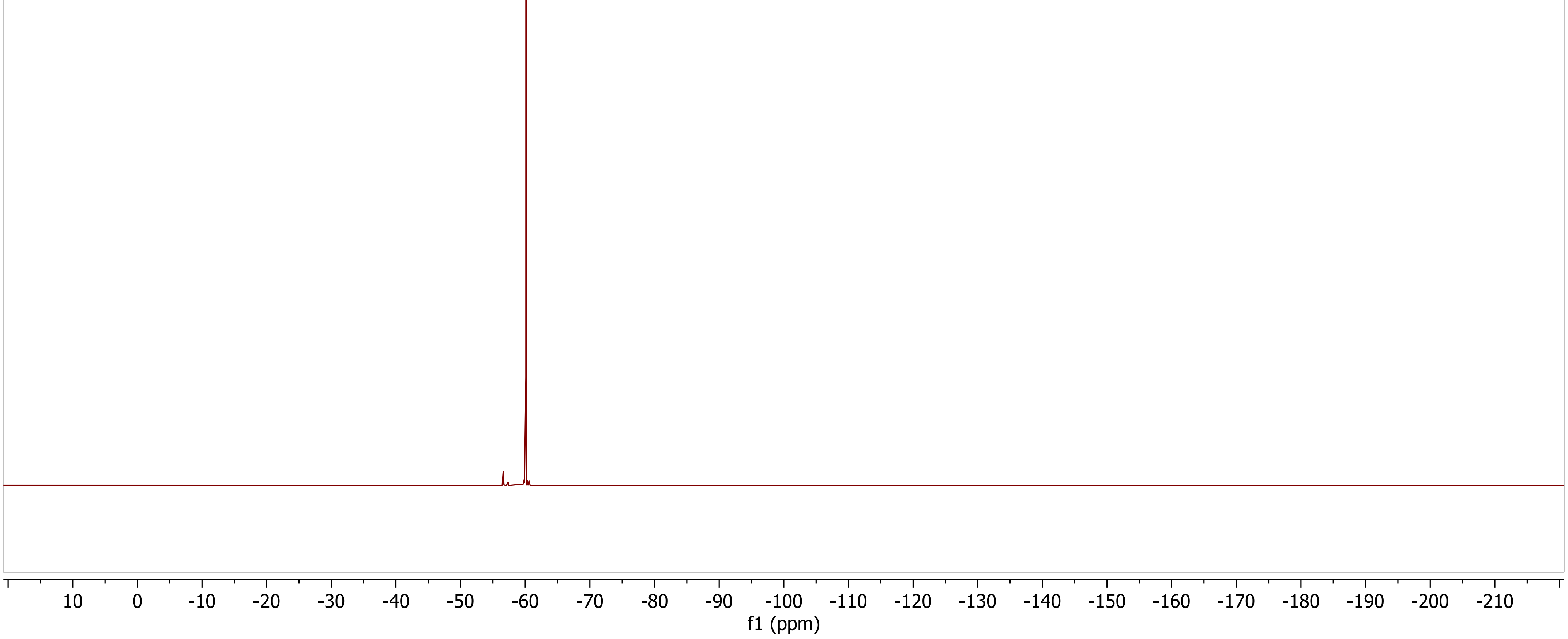

AA-03-137-Fluorine.3.fid — AA-03-137-Fluorine

HSQC NMR

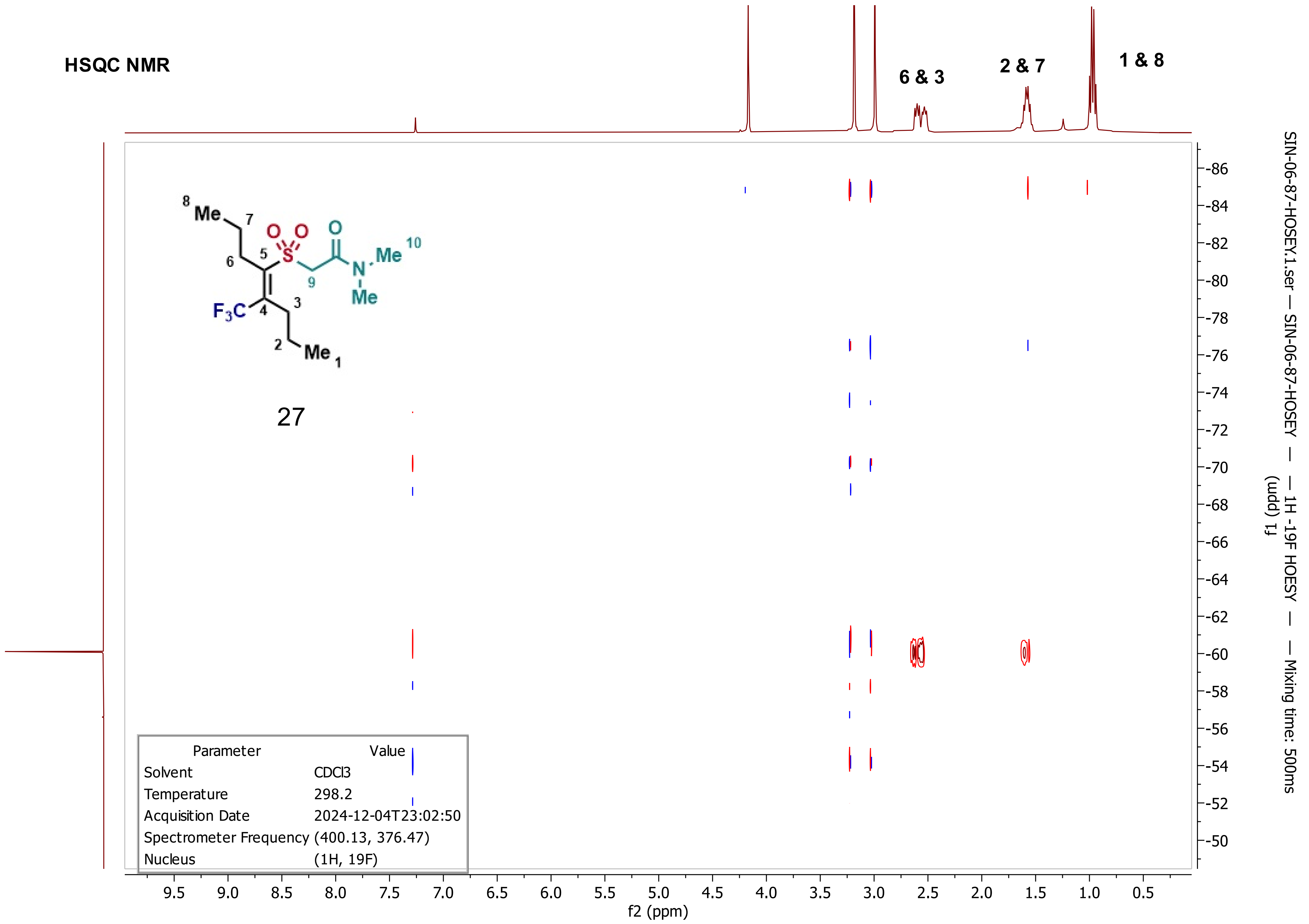

Parameter	Value
Solvent	CDCl3
Temperature	298.2
Acquisition Date	2024-12-04T23:02:50
Spectrometer Frequency	(400.13, 376.47)
Nucleus	(1H, 19F)

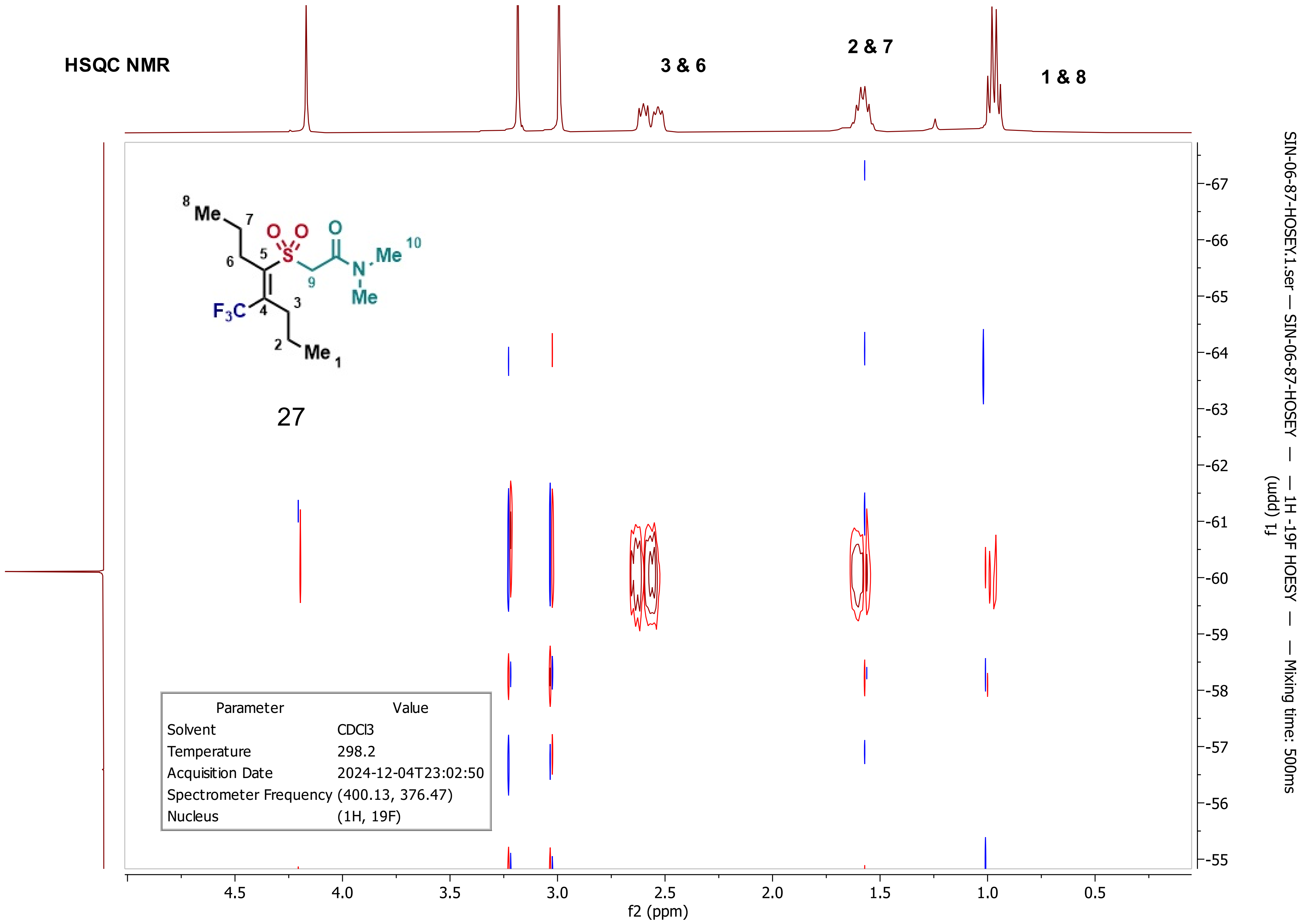

HSQC NMR
3 & 6
2 & 7
1 & 8
27
Parameter Value
Solvent CDCl3
Temperature 298.2
Acquisition Date 2024-12-04T23:02:50
Spectrometer Frequency (400.13, 376.47)
Nucleus (1H, 19F)
f2 (ppm)
f1 (ppm)
SIN-06-87-HOESY.1.ser — SIN-06-87-HOESY — — 1H -19F HOESY — — Mixing time: 500ms

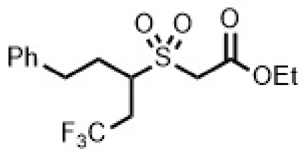

28

Parameter	Value
Solvent	CDCl3
Temperature	298.0
Acquisition Date	2023-11-21T10:07:43
Spectrometer Frequency	400.13
Nucleus	1H

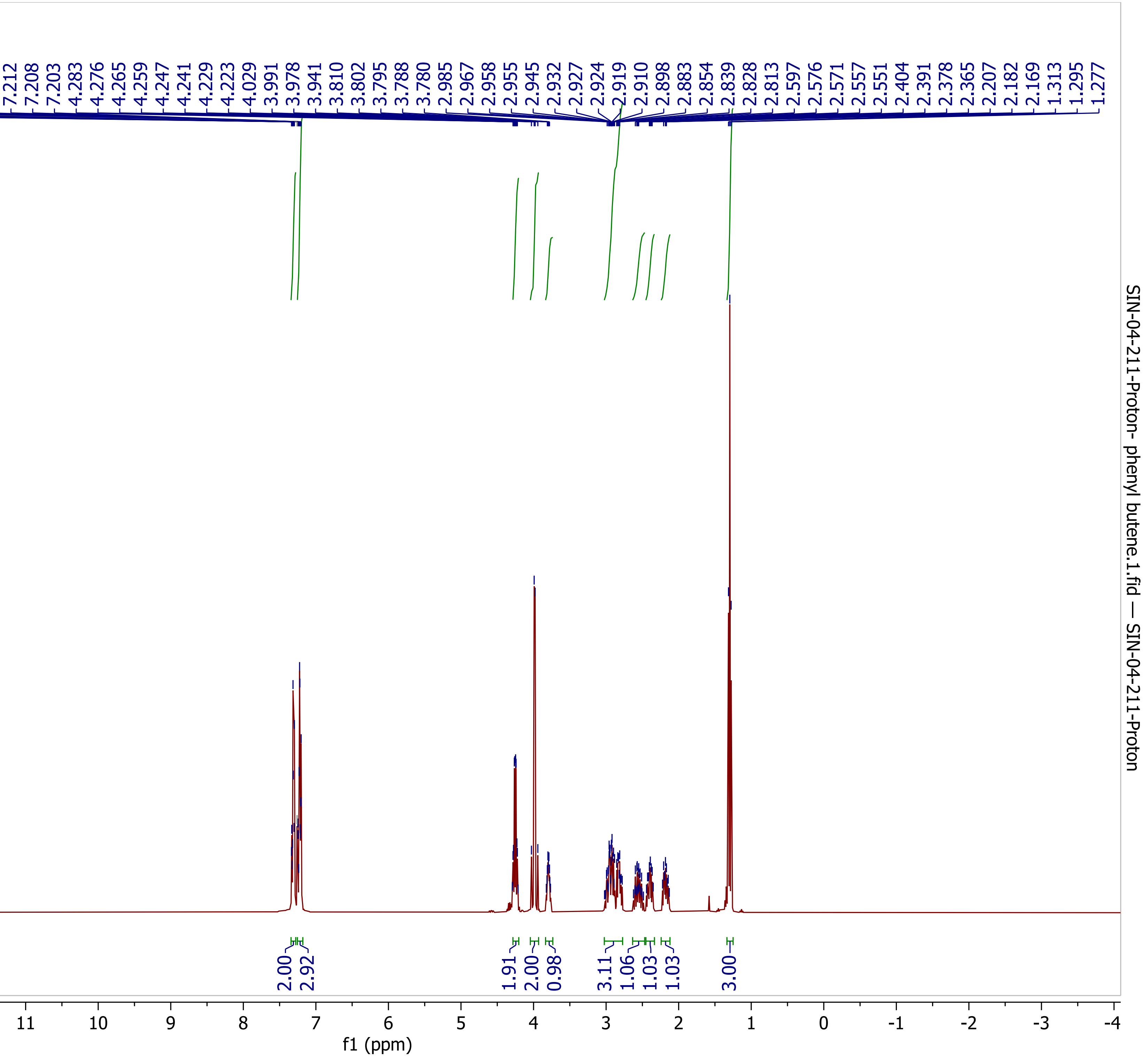

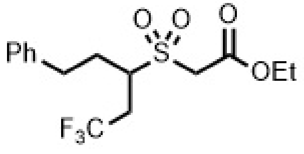

28

Parameter	Value
Solvent	CDCl3
Temperature	298.0
Acquisition Date	2023-11-22T01:25:15
Spectrometer Frequency	100.62
Nucleus	13C

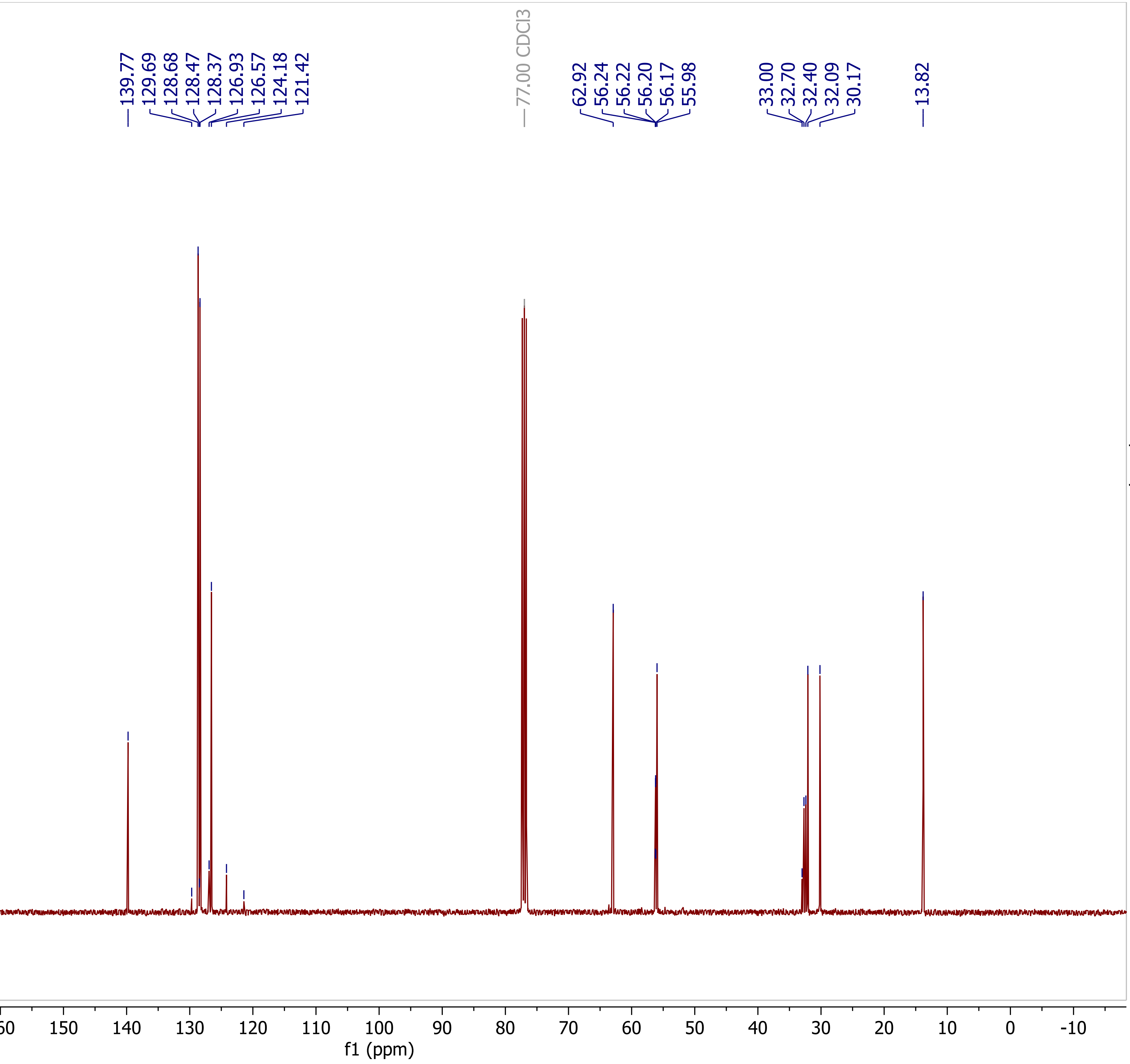

SIN-04-211-C phenyl butene.1.fid — SIN-04-211-H

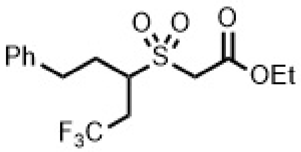

28

Parameter	Value
Solvent	CDCl3
Temperature	298.0
Acquisition Date	2023-11-21T10:12:04
Spectrometer Frequency	376.46
Nucleus	19F

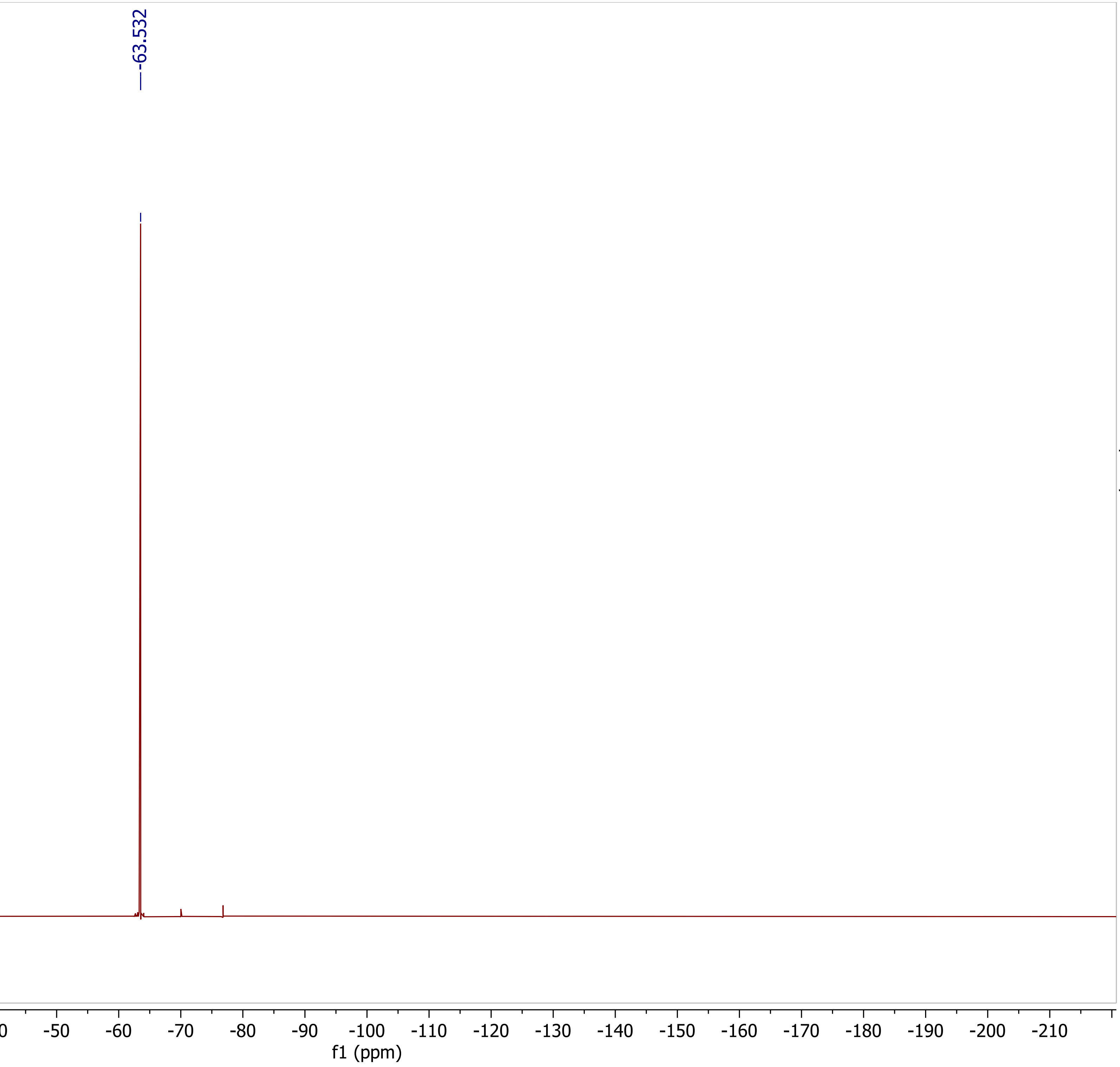

SIN-04-211-F- phenyl butene.3.fid — SIN-04-211-F

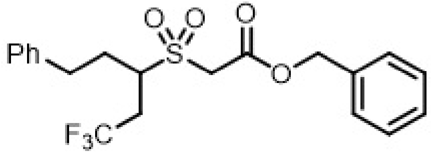

29

Parameter	Value
Solvent	CDCl3
Temperature	298.0
Acquisition Date	2024-03-11T18:18:39
Spectrometer Frequency	400.13
Nucleus	1H

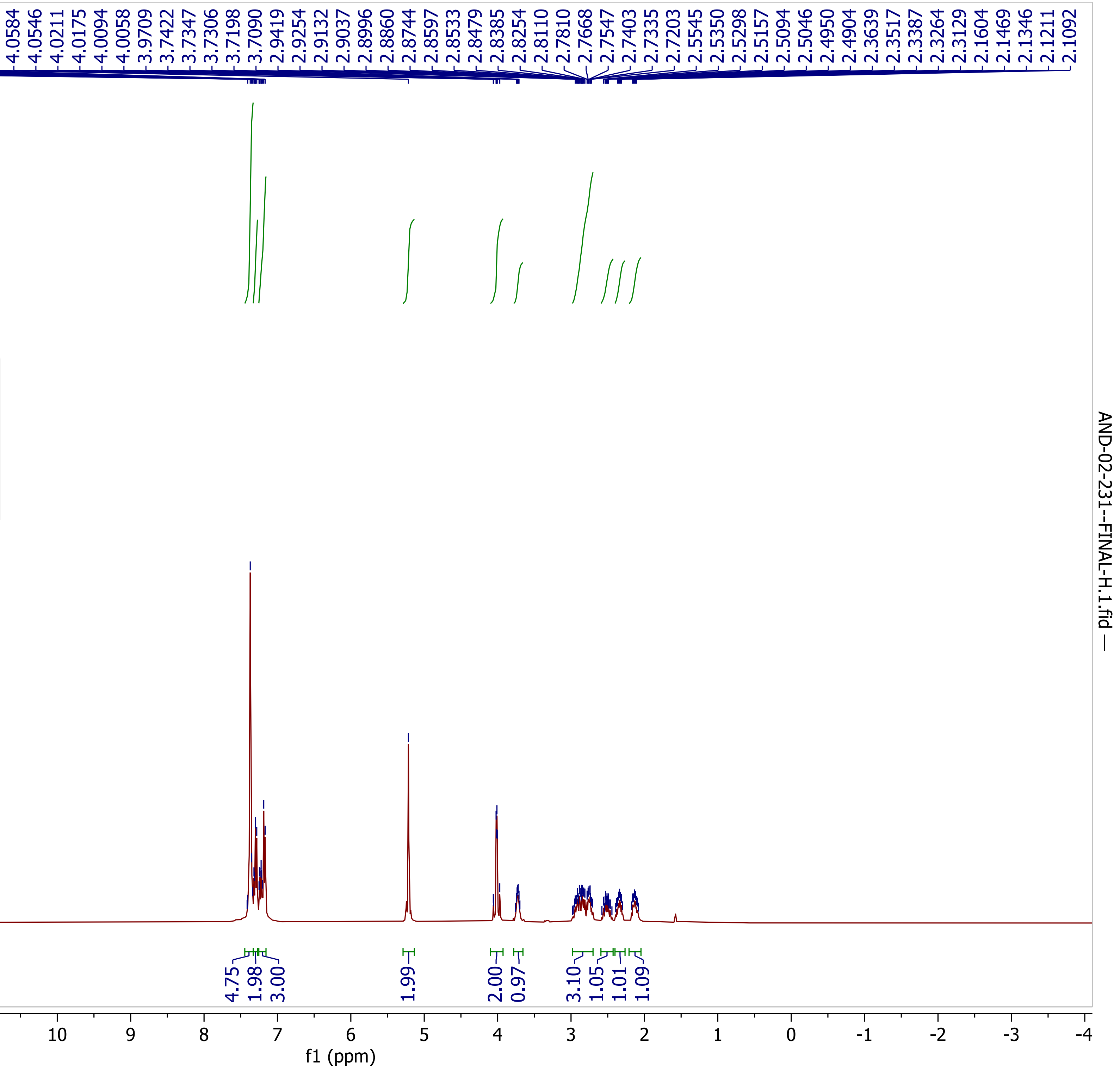

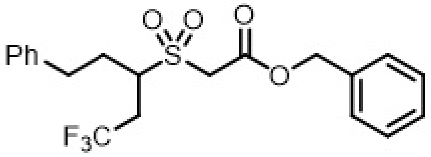

29

Parameter	Value
Solvent	CDCl3
Temperature	298.0
Acquisition Date	2024-03-12T01:55:55
Spectrometer Frequency	100.62
Nucleus	13C

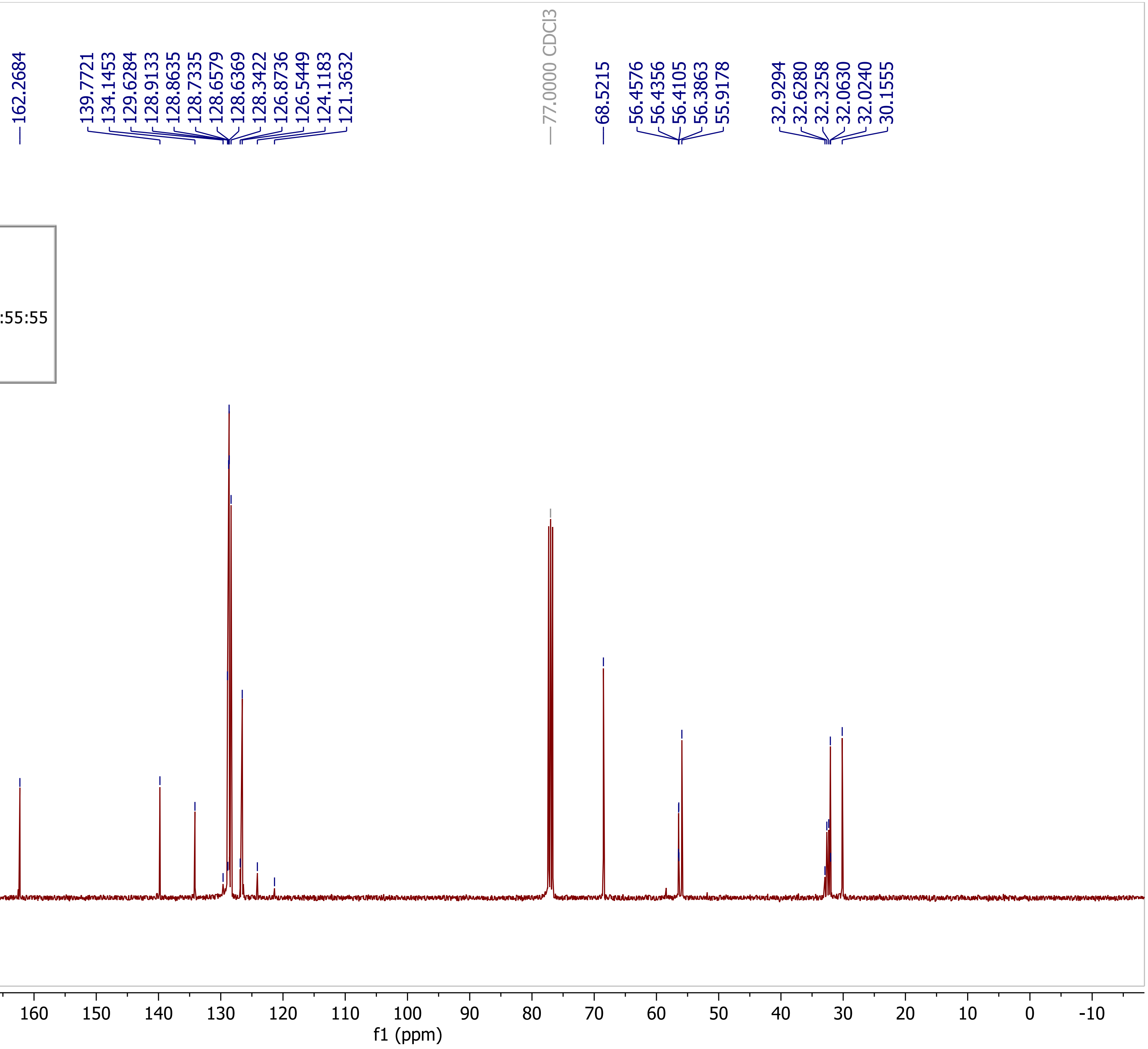

AND-02-231--FINAL-13C.5.fid — AND-02-231--FINAL-13C

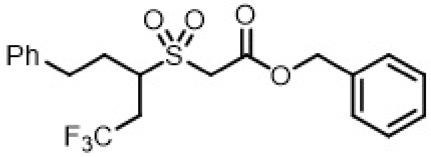

29

Parameter	Value
Solvent	CDCl3
Temperature	298.0
Acquisition Date	2024-03-12T00:53:11
Spectrometer Frequency	376.46
Nucleus	19F

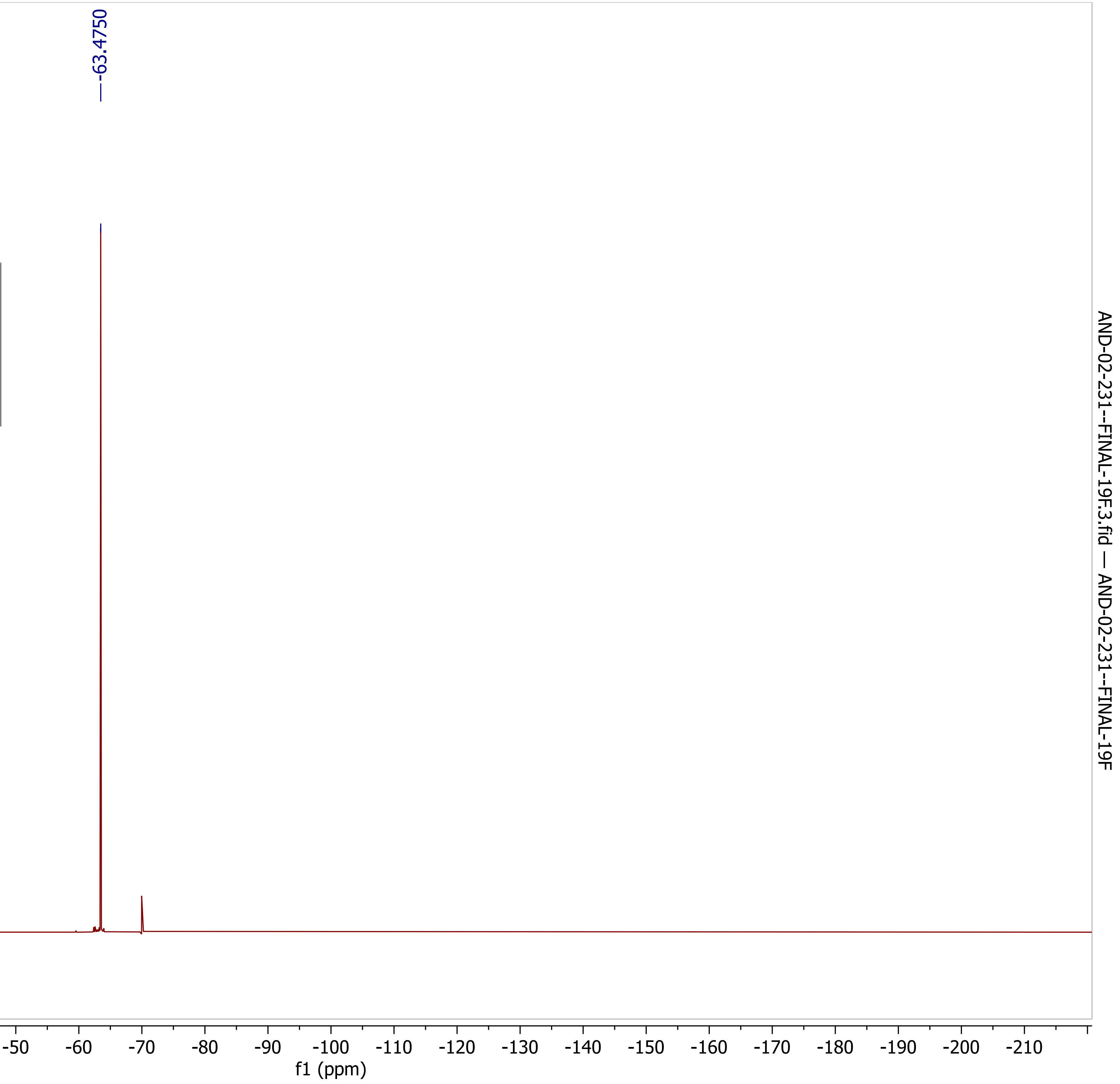

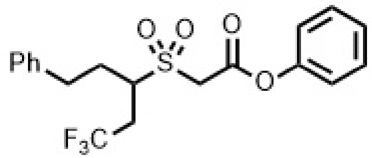

30

Parameter	Value
Solvent	CDCl3
Temperature	298.0
Acquisition Date	2024-03-22T23:04:12
Spectrometer Frequency	400.13
Nucleus	1H

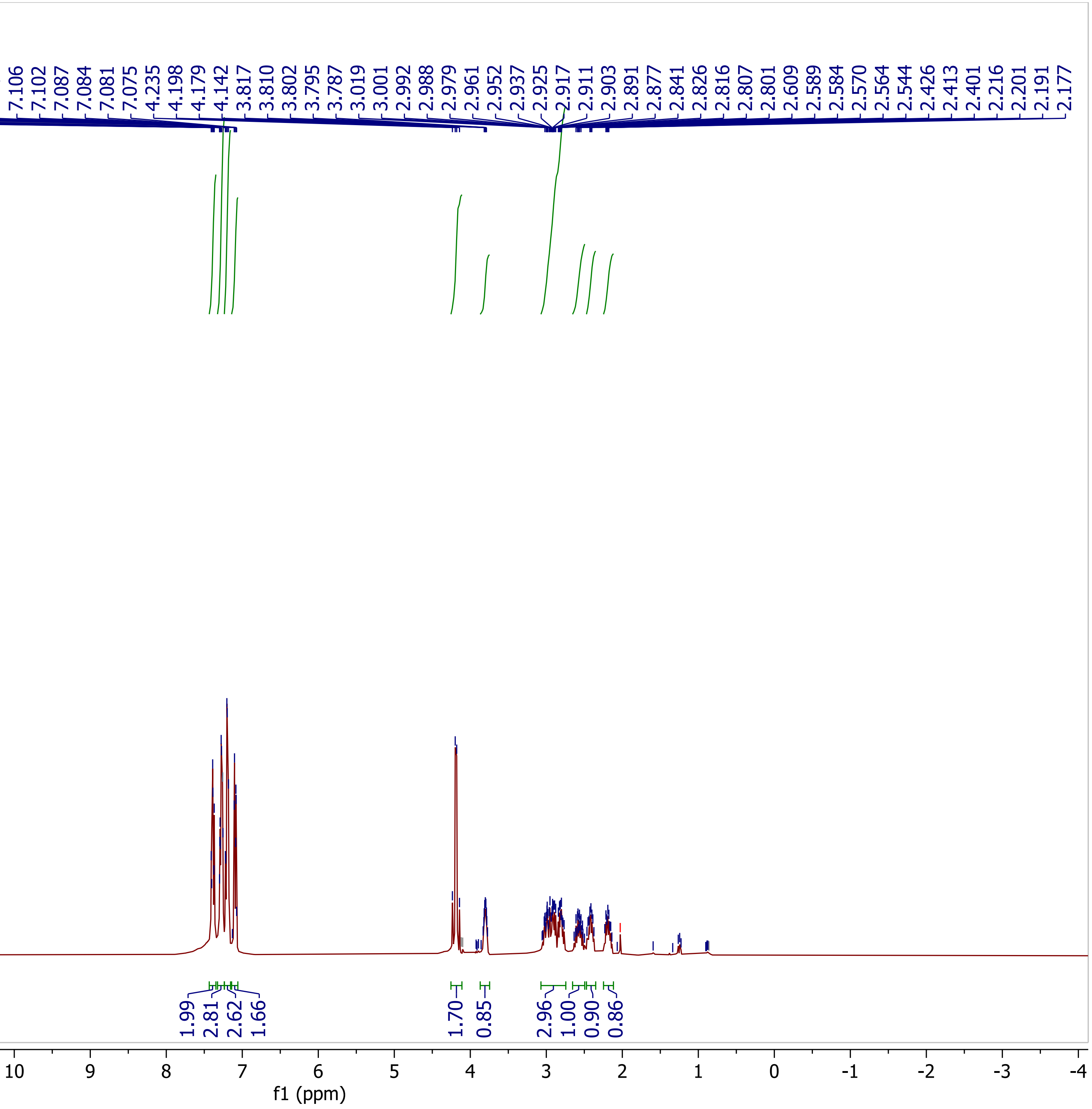

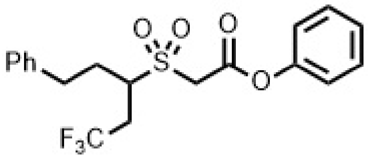

30

Parameter	Value
Solvent	CDCl3
Temperature	298.0
Acquisition Date	2024-03-23T00:06:26
Spectrometer Frequency	100.62
Nucleus	13C

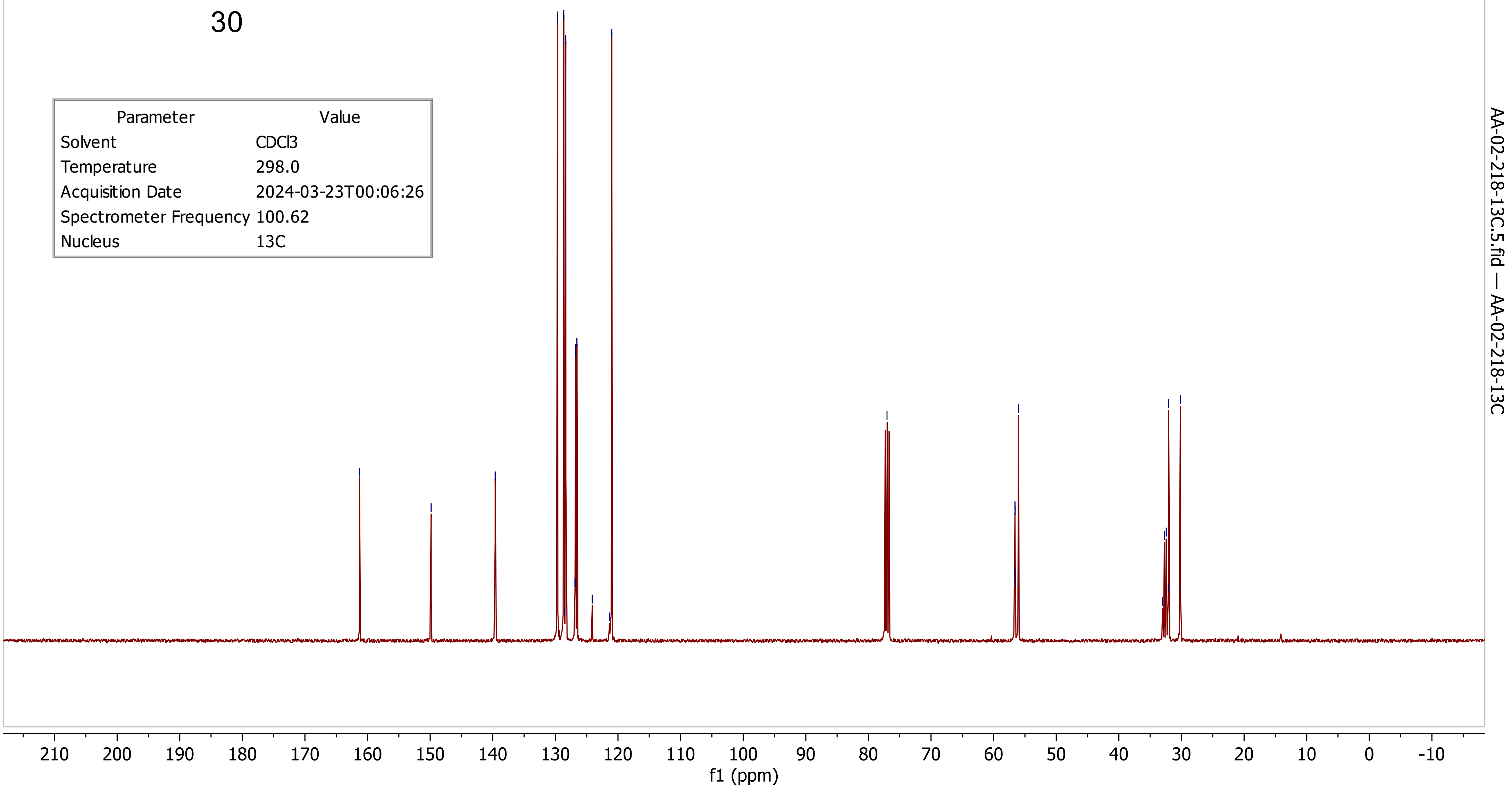

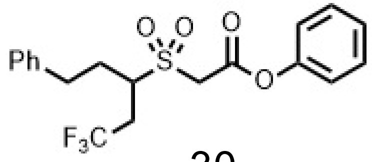

30

Parameter	Value
Solvent	CDCl3
Temperature	298.0
Acquisition Date	2024-03-23T00:10:41
Spectrometer Frequency	376.46
Nucleus	19F

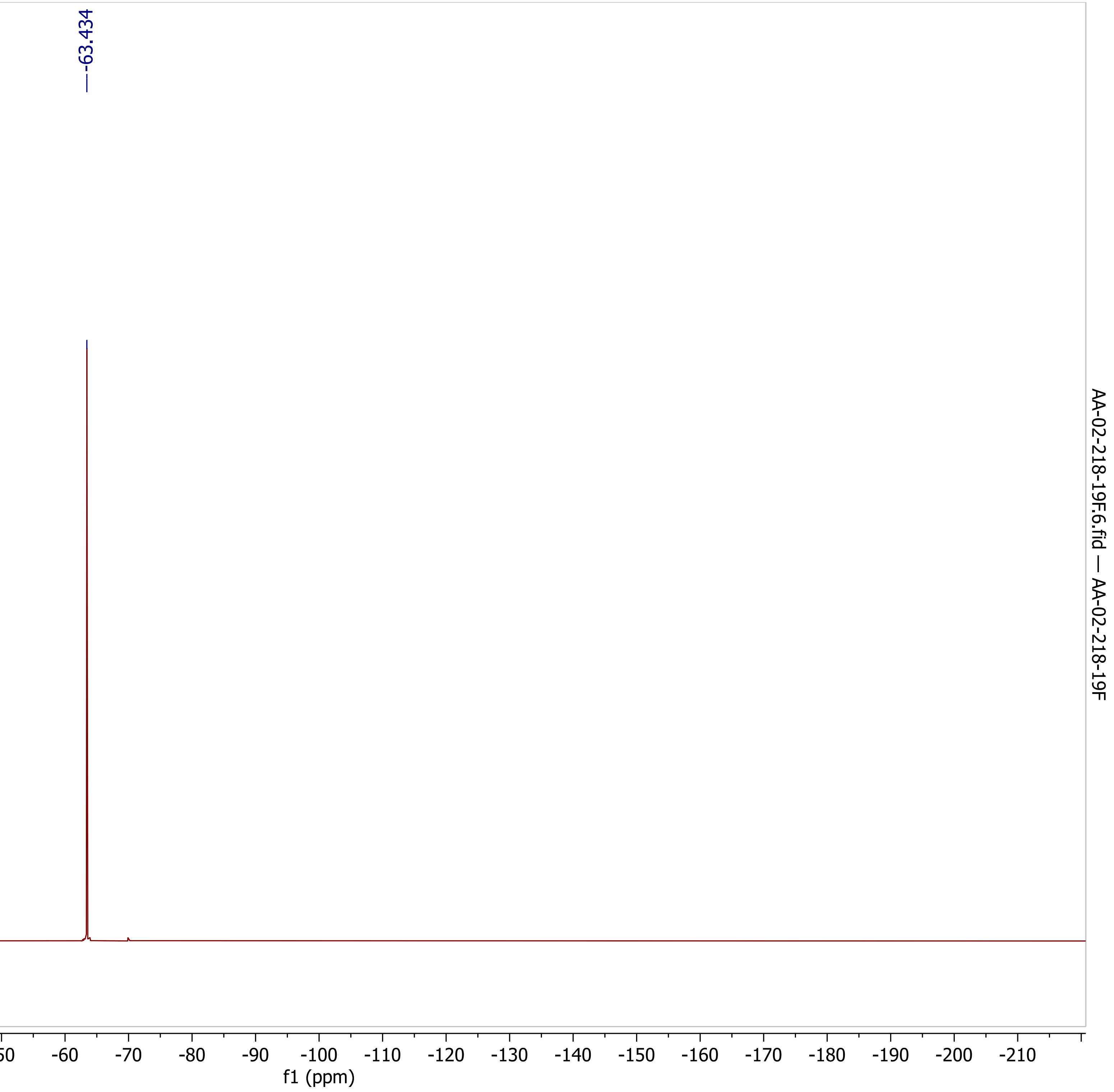

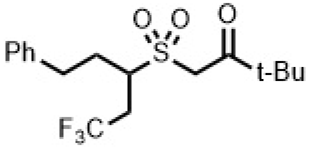

31

Parameter	Value
Solvent	CDCl3
Temperature	298.0
Acquisition Date	2024-03-28T11:40:23
Spectrometer Frequency	400.13
Nucleus	1H

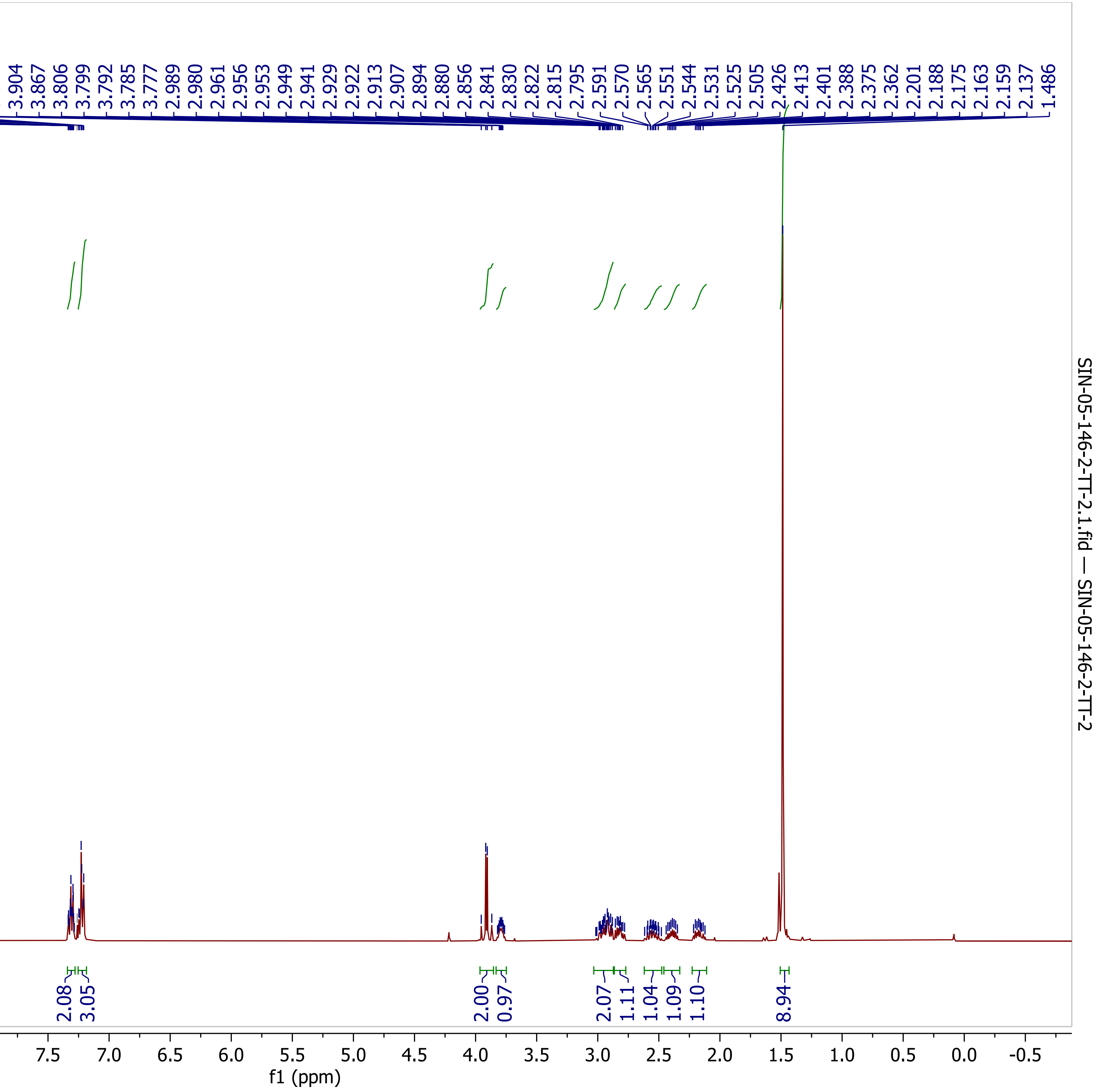

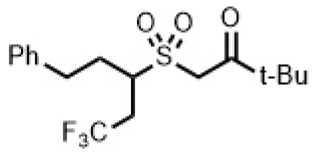

31

Parameter	Value
Solvent	CDCl3
Temperature	298.0
Acquisition Date	2024-03-12T04:12:35
Spectrometer Frequency	100.62
Nucleus	13C

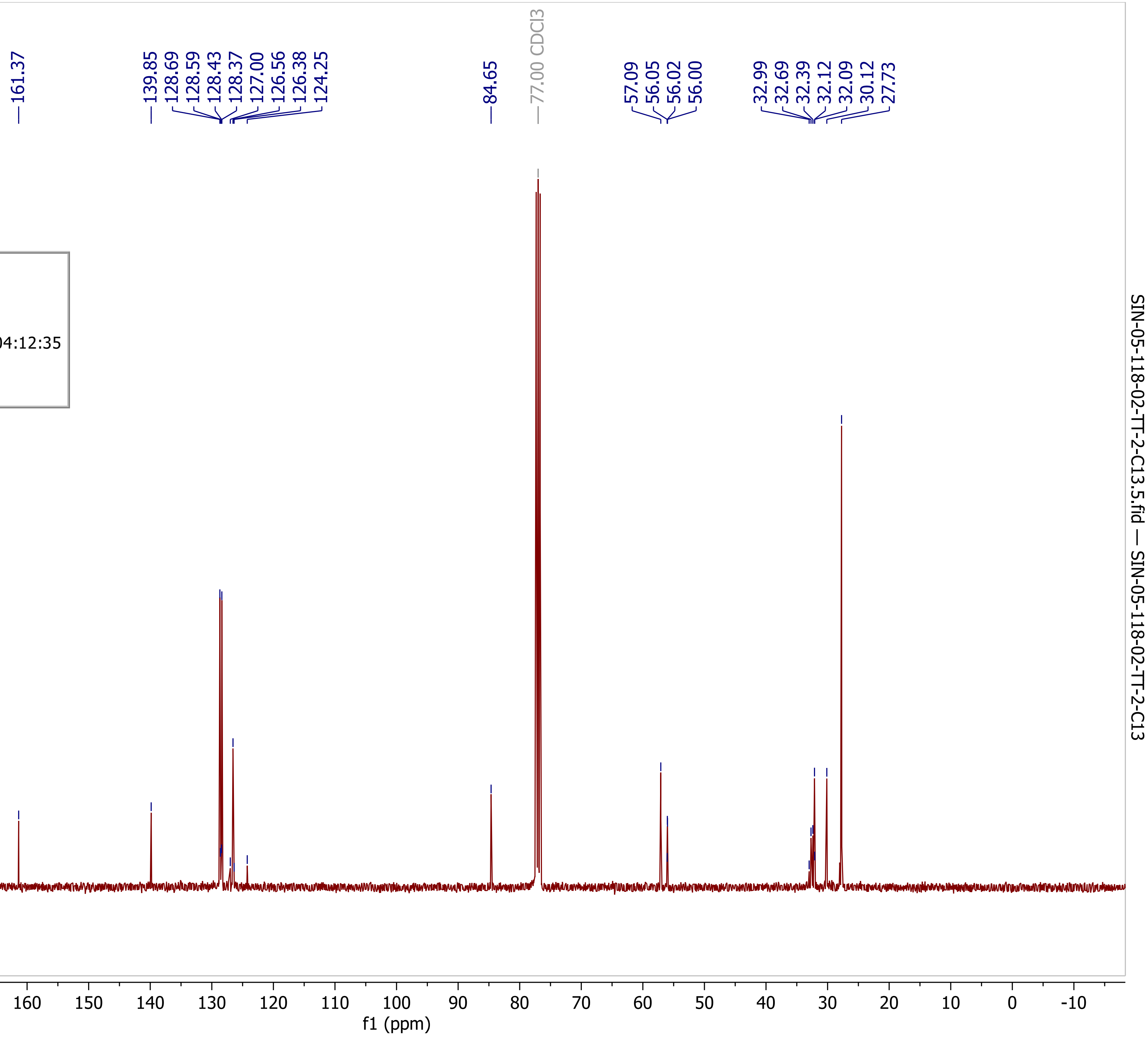

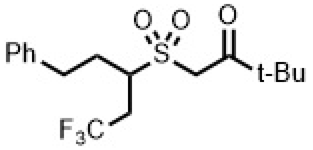

31

Parameter	Value
Solvent	CDCl3
Temperature	298.0
Acquisition Date	2024-03-27T23:45:39
Spectrometer Frequency	376.46
Nucleus	19F

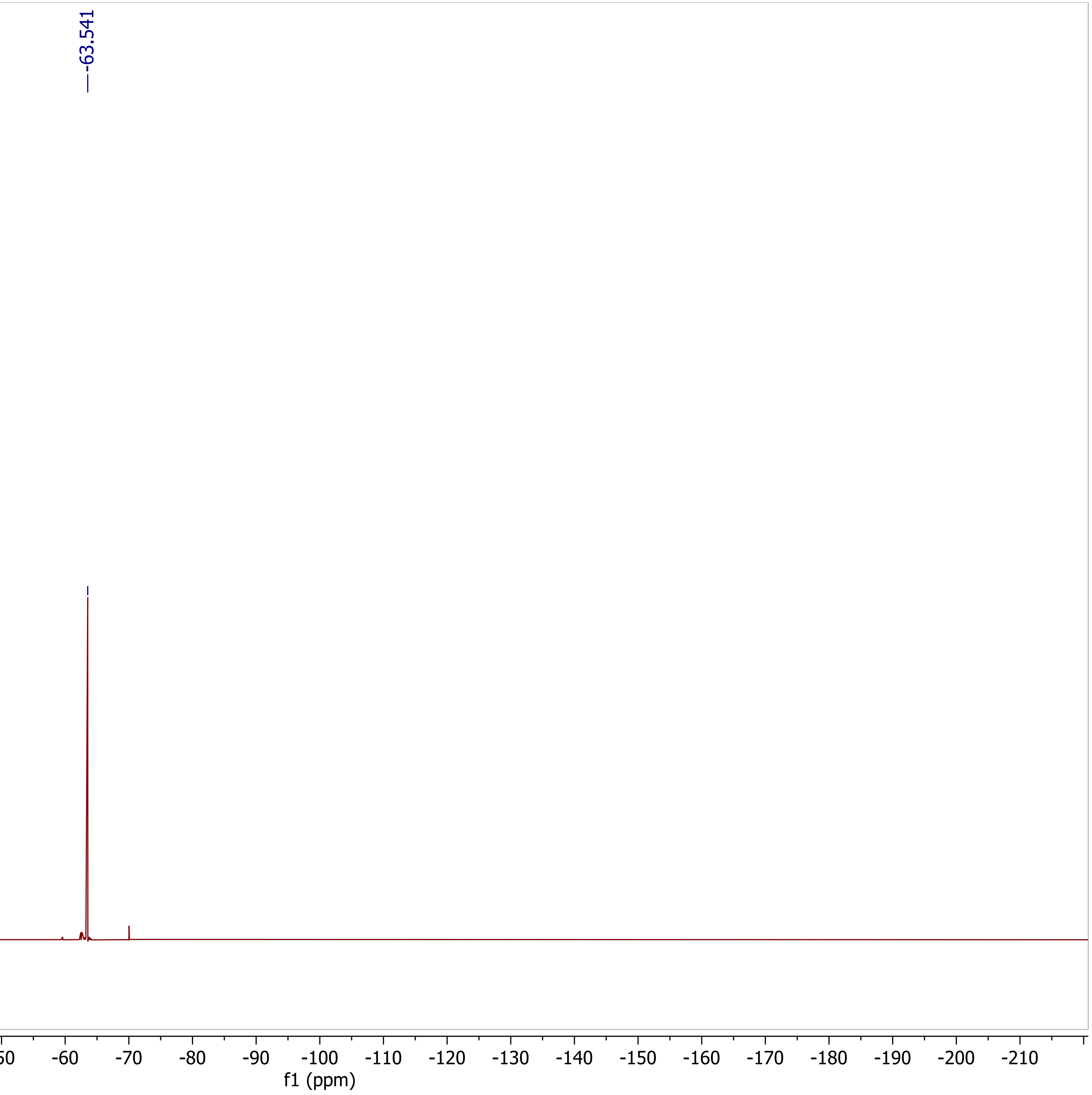

SIN-05-146-2-TT-5-F.3.fid — SIN-05-146-2-TT-5-F

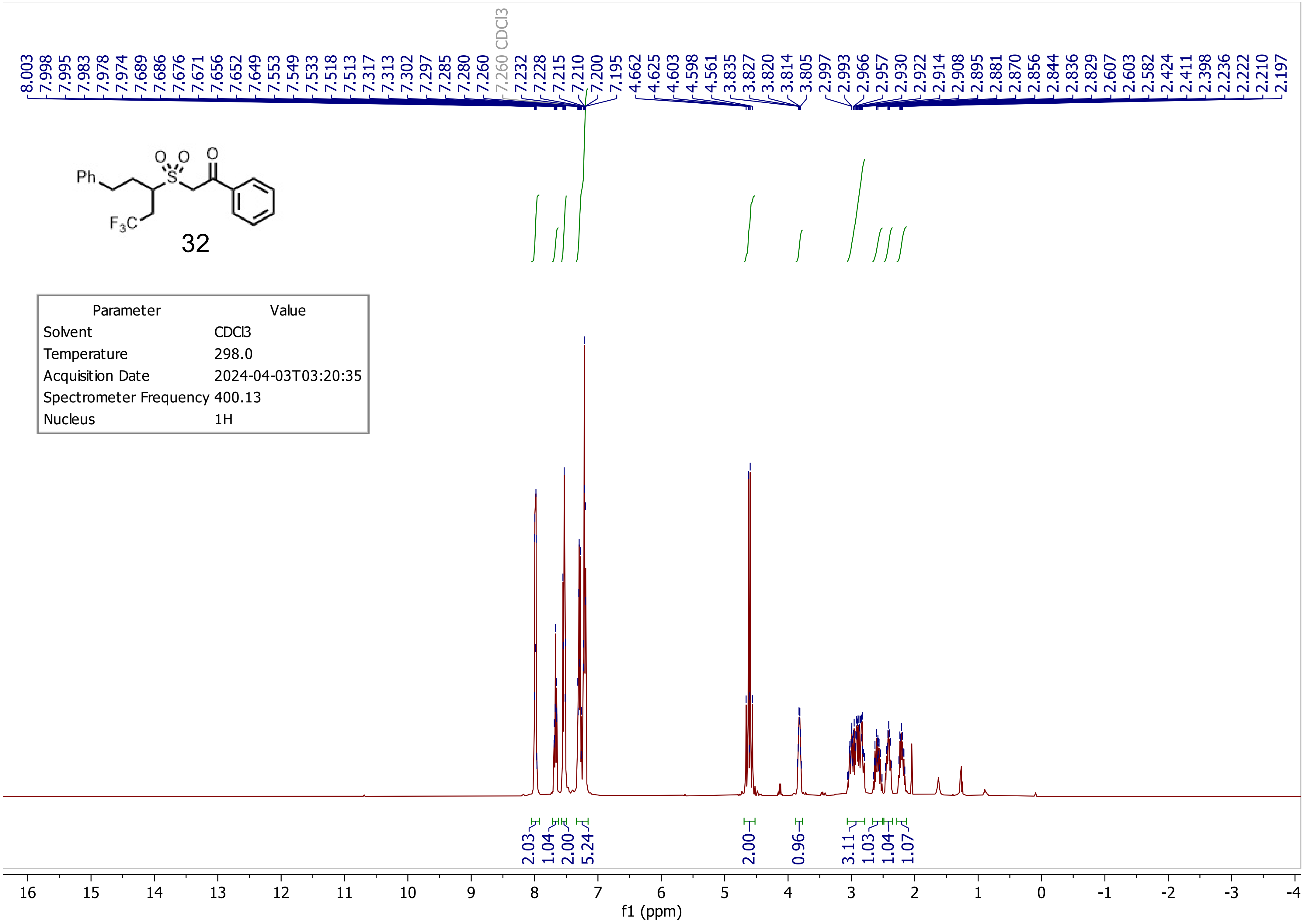

Parameter	Value
Solvent	CDCl3
Temperature	298.0
Acquisition Date	2024-04-03T03:20:35
Spectrometer Frequency	400.13
Nucleus	1H

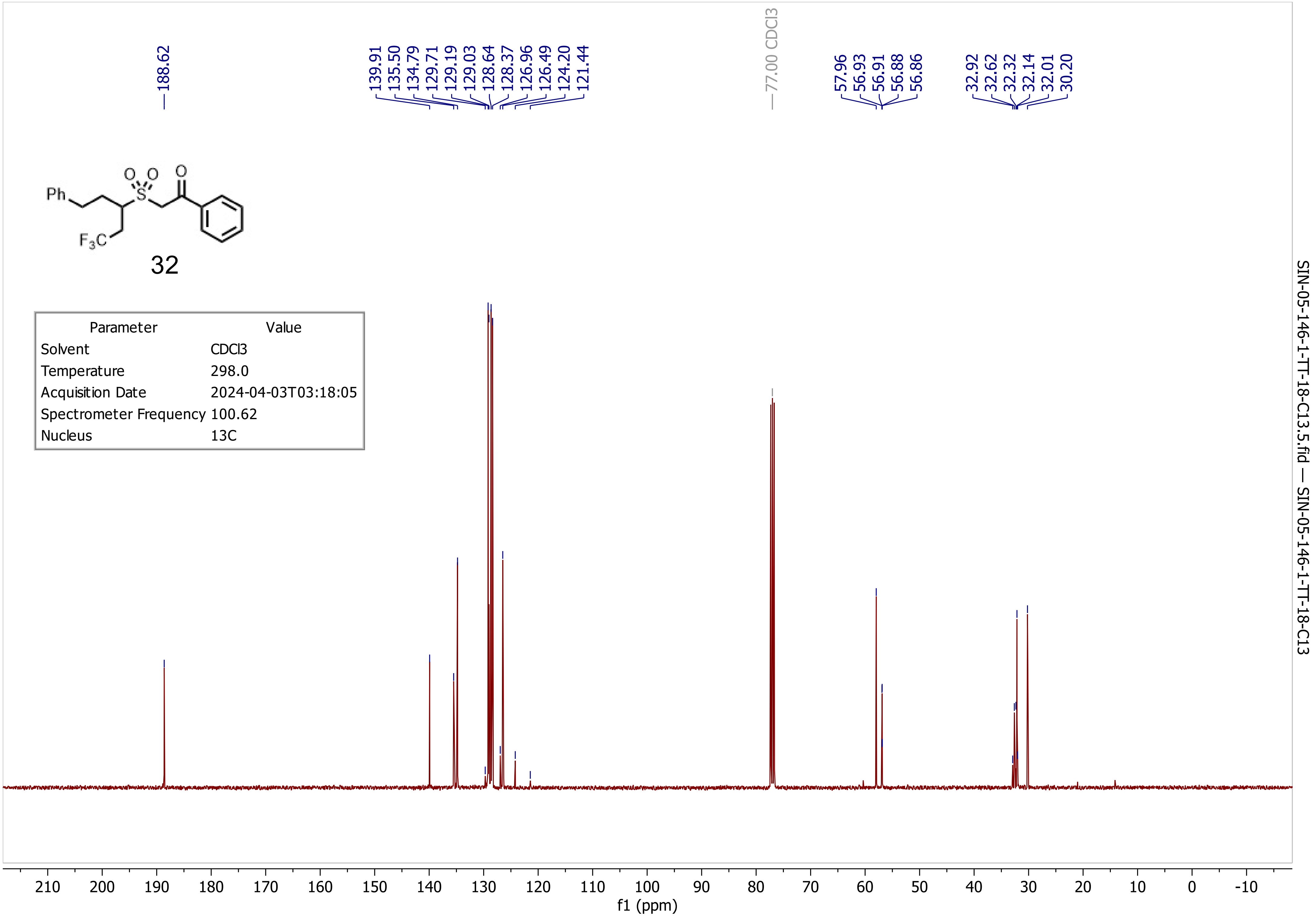

188.62
139.91
135.50
134.79
129.71
129.19
129.03
128.64
128.37
126.96
126.49
124.20
121.44
77.00 CDCl3
57.96
56.93
56.91
56.88
56.86
32.92
32.62
32.32
32.14
32.01
30.20
Ph
O
O
O
S
F3C
32
Parameter Value
Solvent CDCl3
Temperature 298.0
Acquisition Date 2024-04-03T03:18:05
Spectrometer Frequency 100.62
Nucleus 13C
SIN-05-146-1-TT-18-C13.5.fid — SIN-05-146-1-TT-18-C13
210 200 190 180 170 160 150 140 130 120 110 100 90 80 70 60 50 40 30 20 10 0 -10
f1 (ppm)

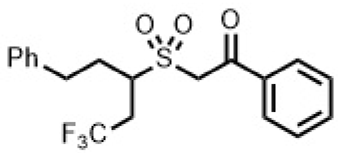

32

Parameter	Value
Solvent	CDCl3
Temperature	298.0
Acquisition Date	2024-04-03T02:17:40
Spectrometer Frequency	376.46
Nucleus	19F

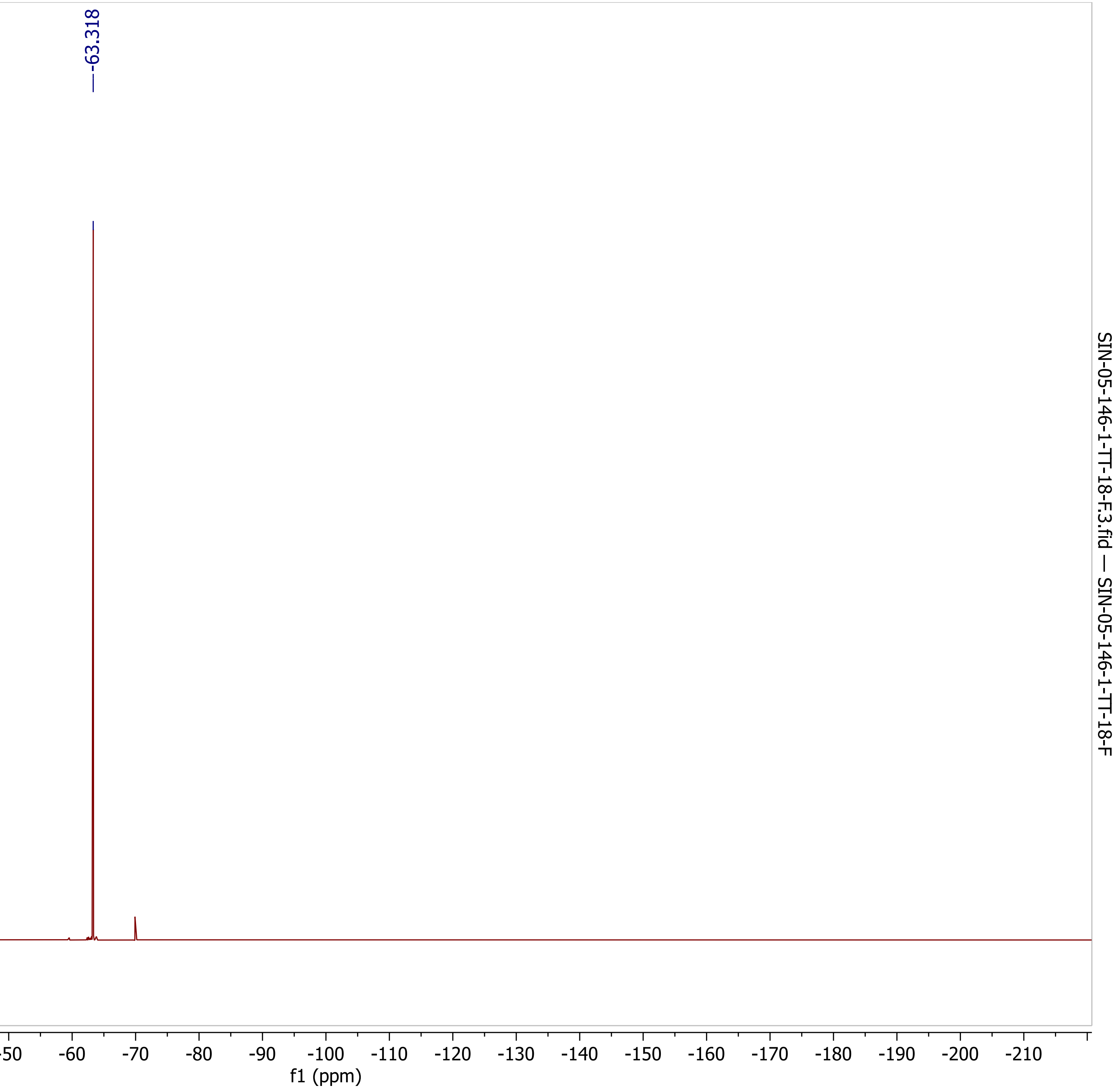

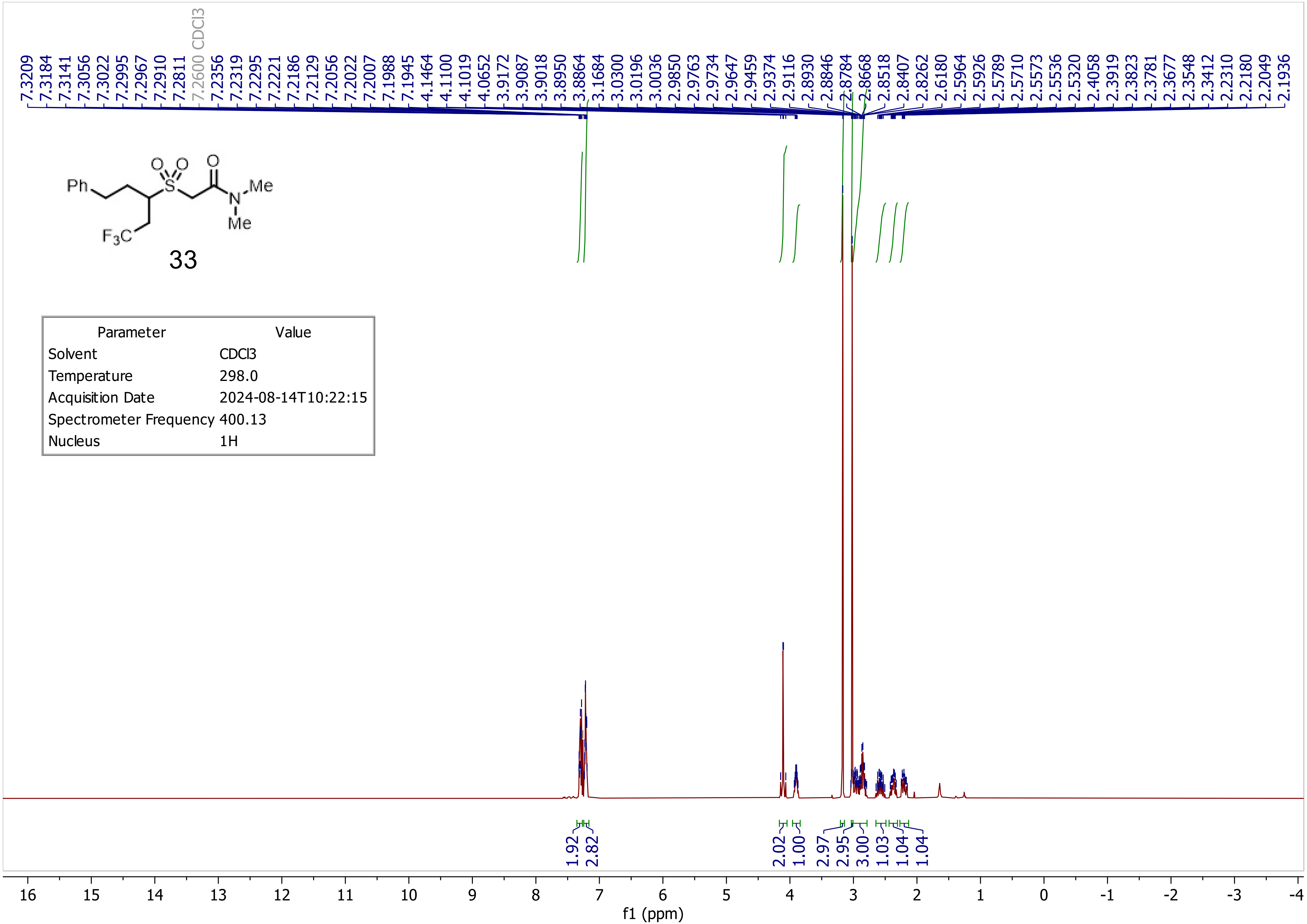

Parameter	Value
Solvent	CDCl3
Temperature	298.0
Acquisition Date	2024-08-14T10:22:15
Spectrometer Frequency	400.13
Nucleus	1H

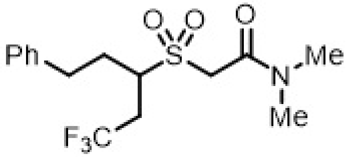

33

Parameter	Value
Solvent	CDCl3
Temperature	298.0
Acquisition Date	2024-08-15T02:21:15
Spectrometer Frequency	100.62
Nucleus	13C

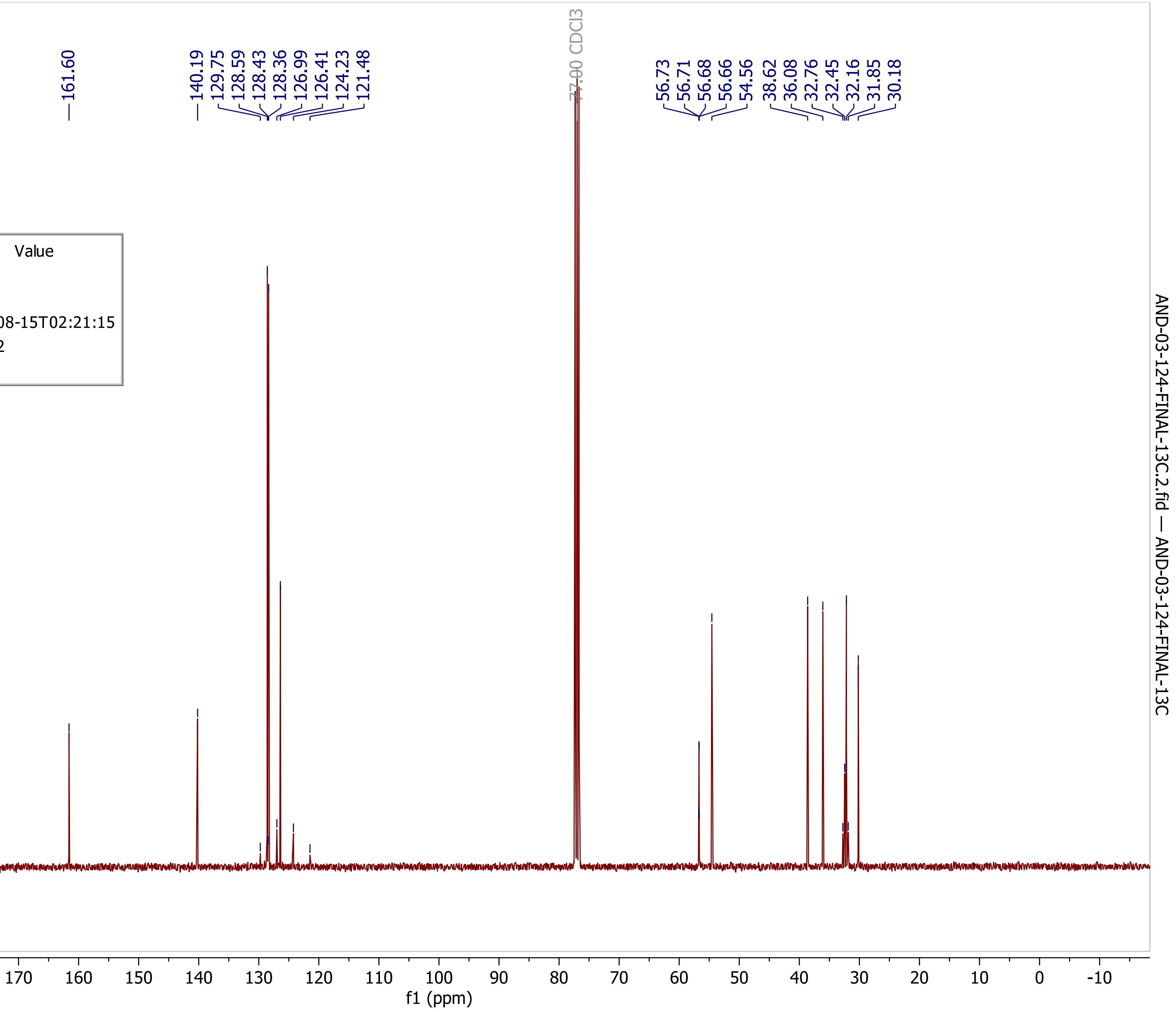

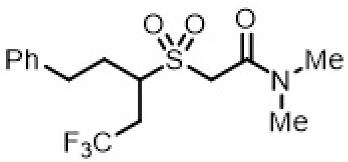

33

Parameter	Value
Solvent	CDCl3
Temperature	298.0
Acquisition Date	2024-08-15T02:25:37
Spectrometer Frequency	376.46
Nucleus	19F

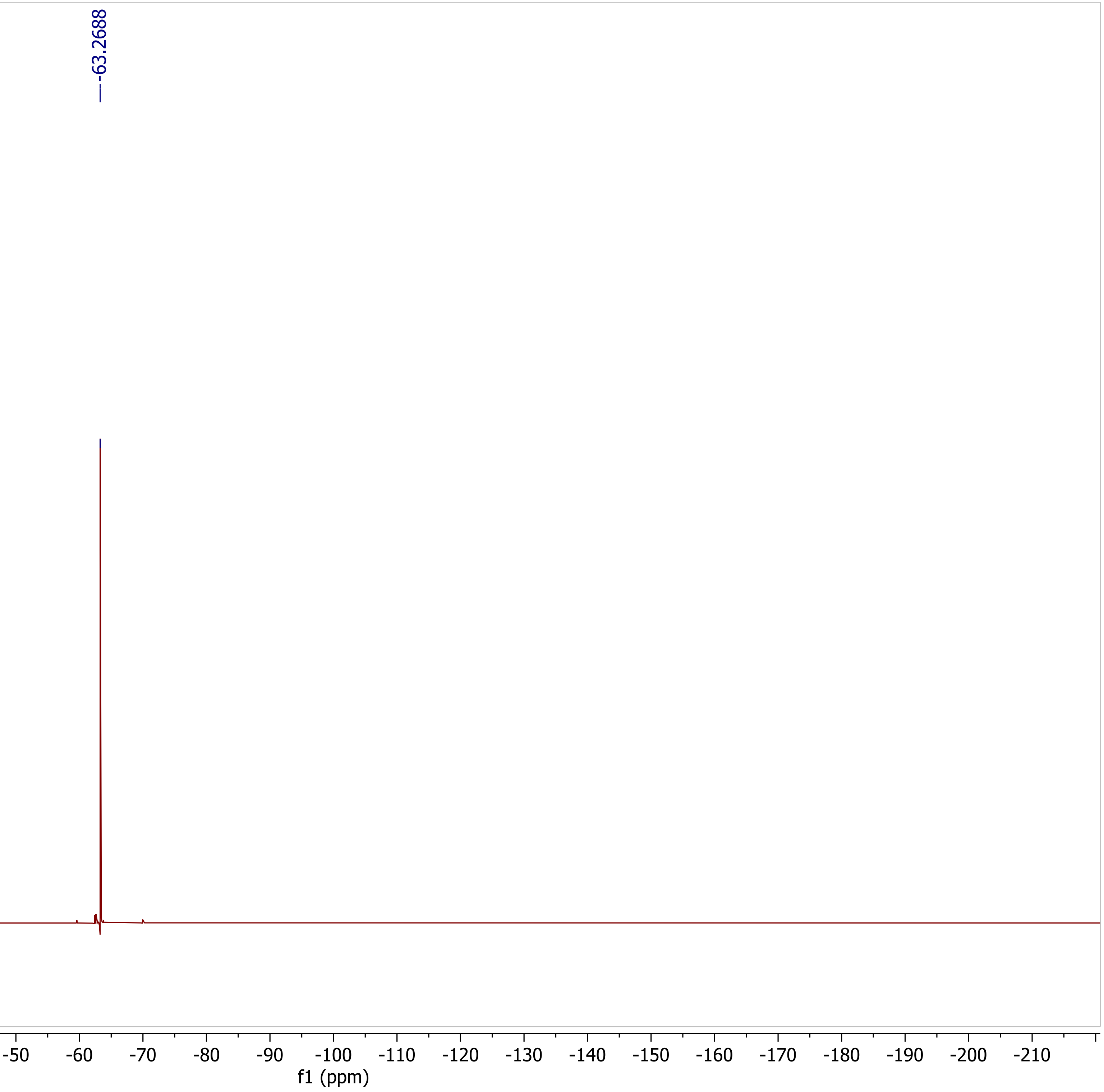

AND-03-124-FINAL-19F.4.fid — AND-03-124-FINAL-19F

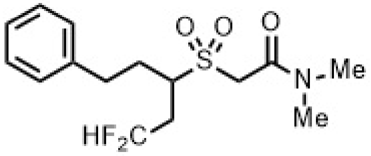

34

Parameter	Value
Solvent	CDCl3
Temperature	298.0
Acquisition Date	2024-10-15T15:30:37
Spectrometer Frequency	400.13
Nucleus	1H

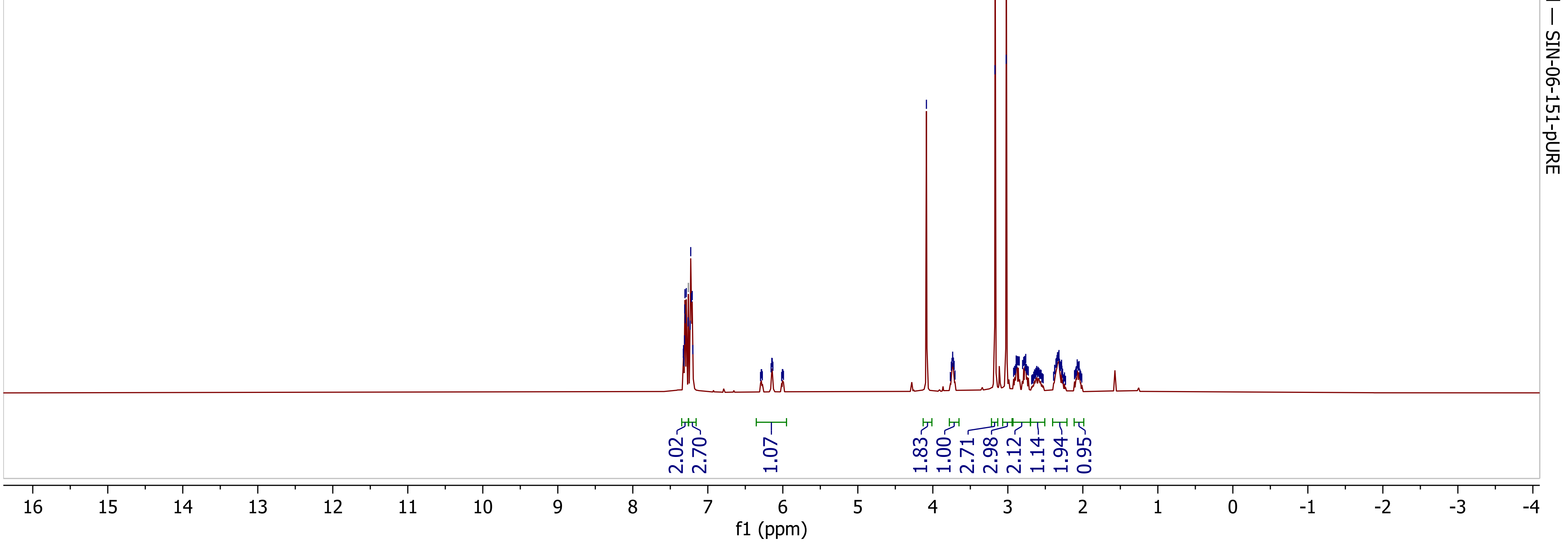

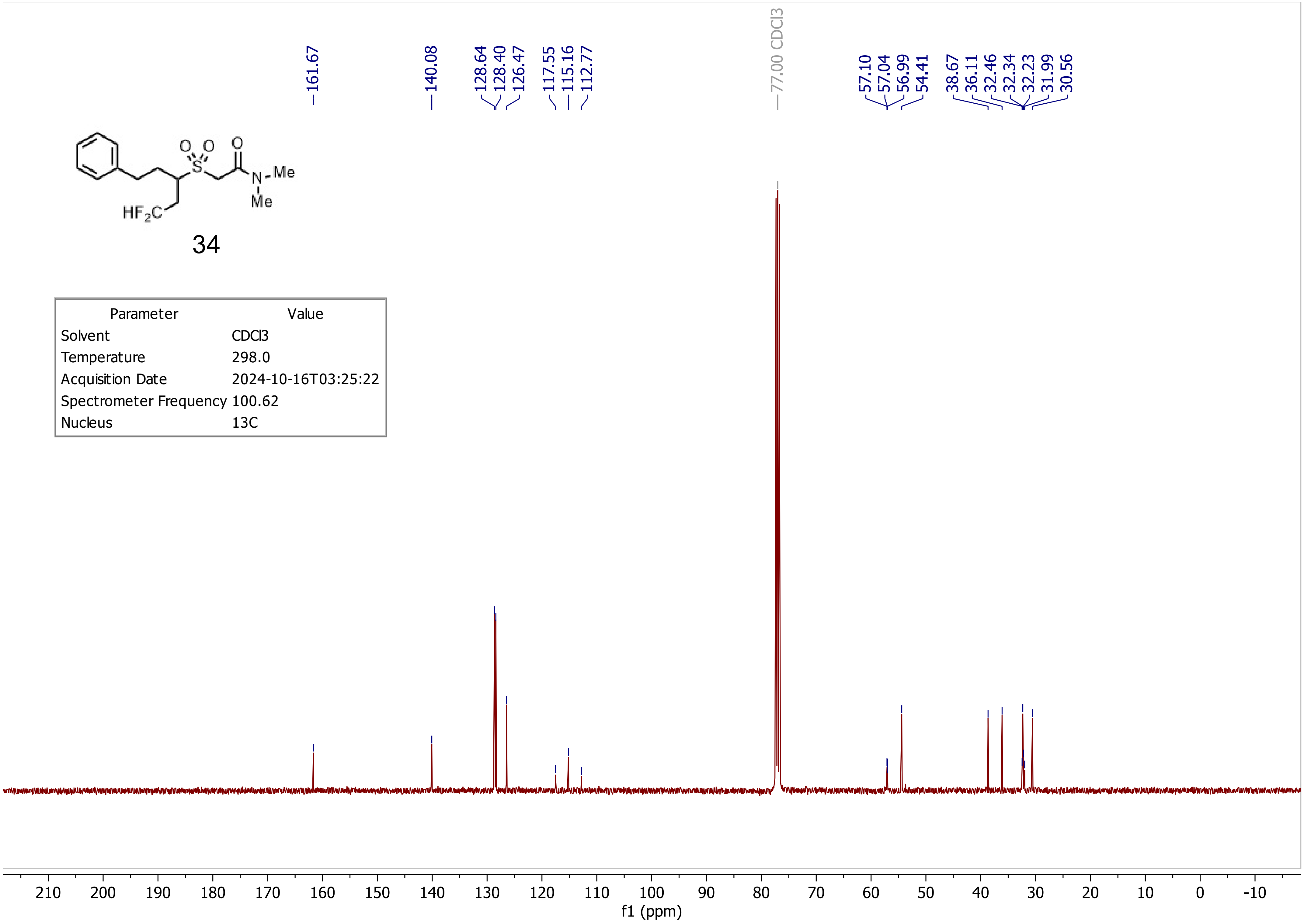

34

Parameter	Value
Solvent	CDCl3
Temperature	298.0
Acquisition Date	2024-10-16T03:25:22
Spectrometer Frequency	100.62
Nucleus	13C

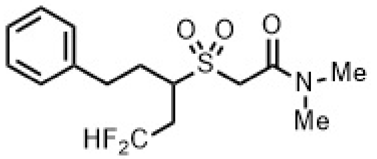

34

Parameter	Value
Solvent	CDCl3
Temperature	298.0
Acquisition Date	2024-10-15T14:11:14
Spectrometer Frequency	376.46
Nucleus	19F

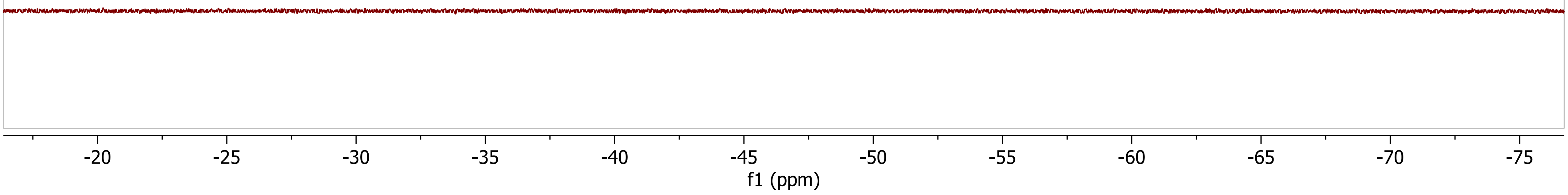

SIN-06-151-TT-8-F.1.fid — SIN-06-151-TT-8-F

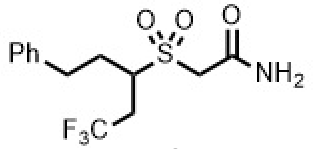

35

Parameter	Value
Solvent	CDCl3
Temperature	298.0
Acquisition Date	2024-02-27T15:09:53
Spectrometer Frequency	400.13
Nucleus	1H

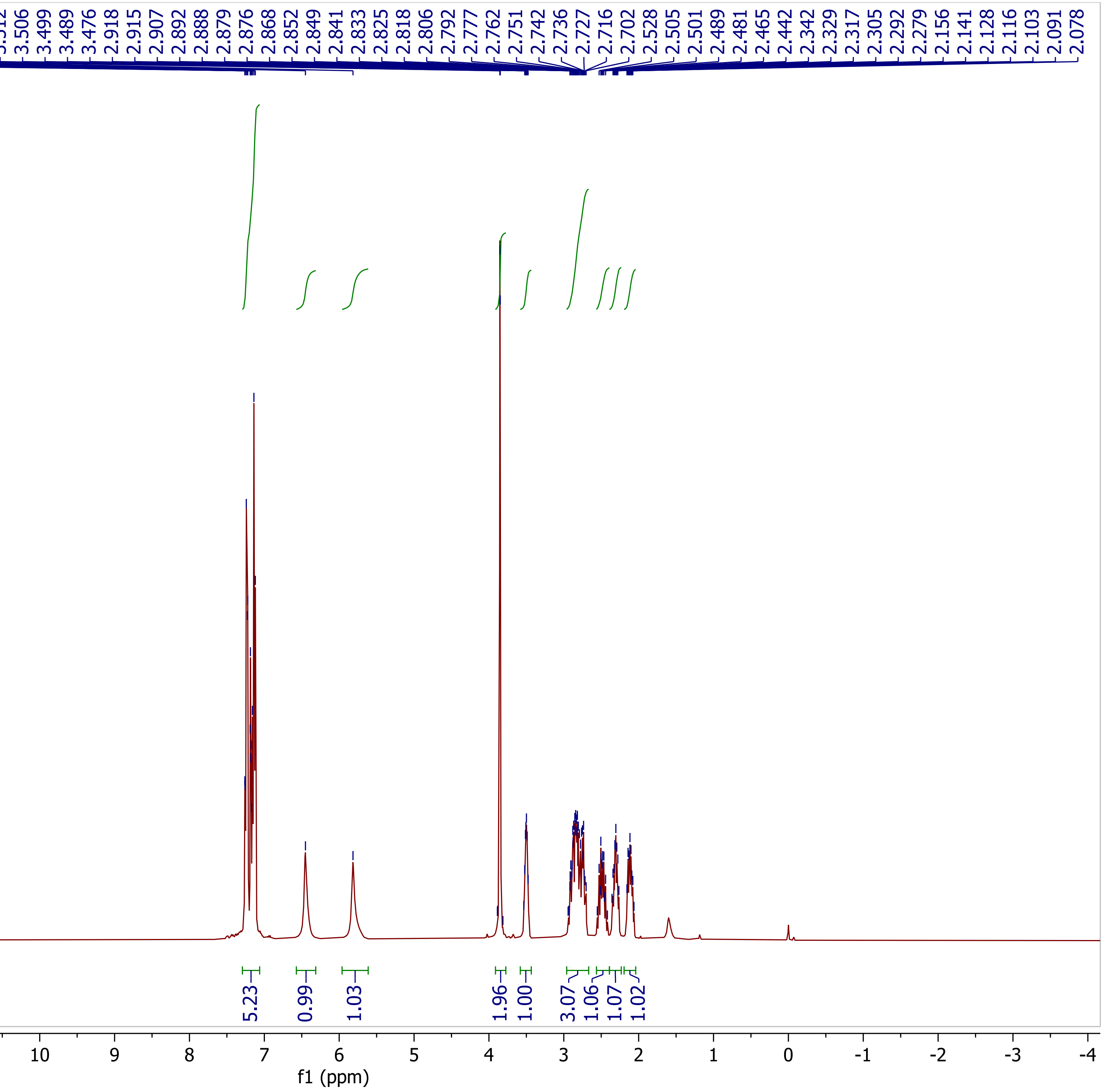

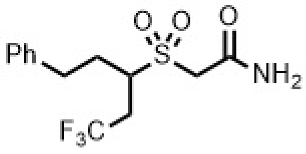

35

Parameter	Value
Solvent	CDCl3
Temperature	298.0
Acquisition Date	2024-03-02T00:58:46
Spectrometer Frequency	100.62
Nucleus	13C

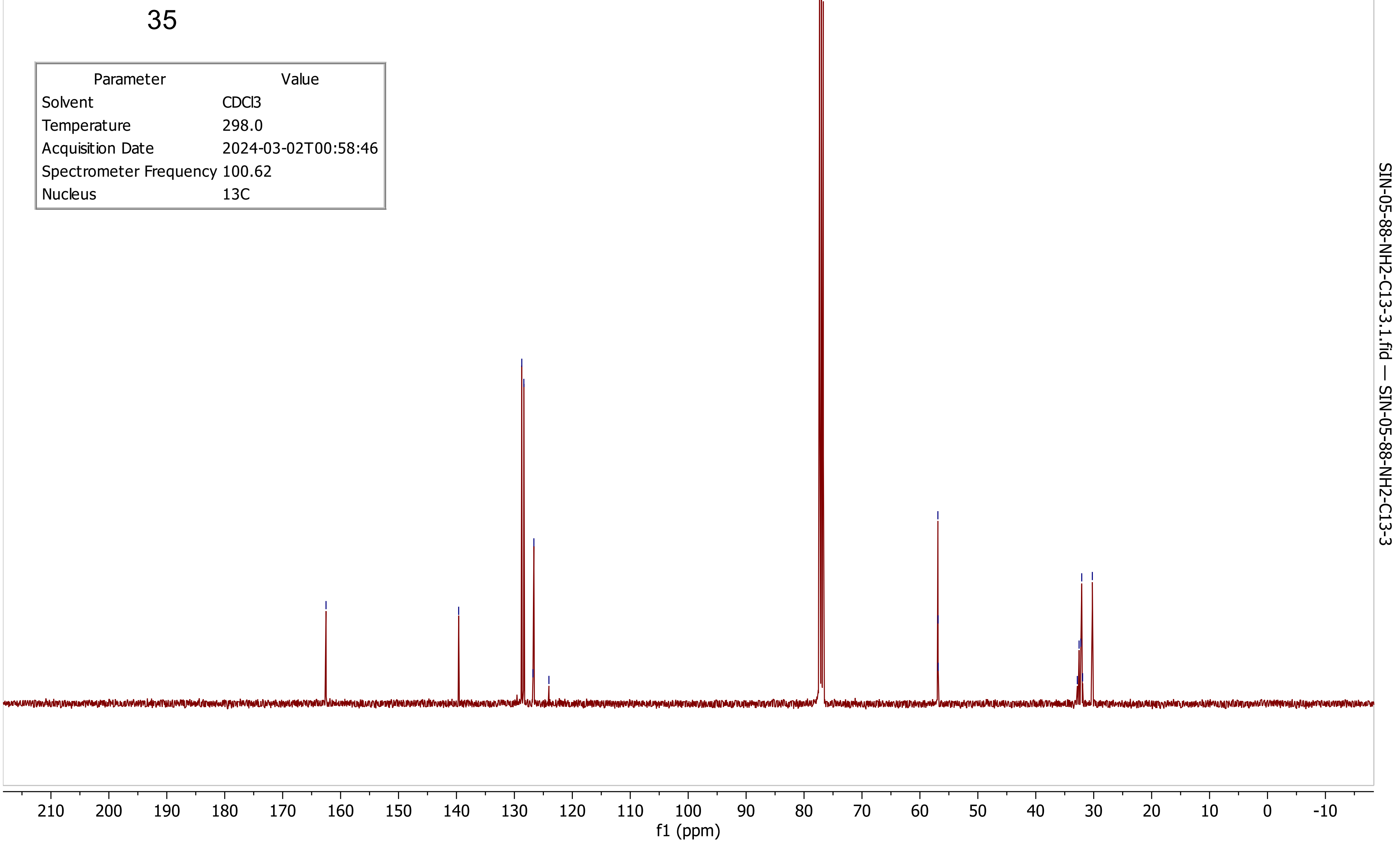

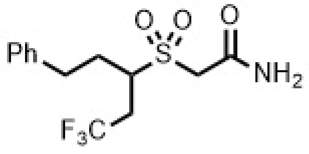

35

Parameter	Value
Solvent	CDCl3
Temperature	298.0
Acquisition Date	2024-02-27T15:13:47
Spectrometer Frequency	376.46
Nucleus	19F

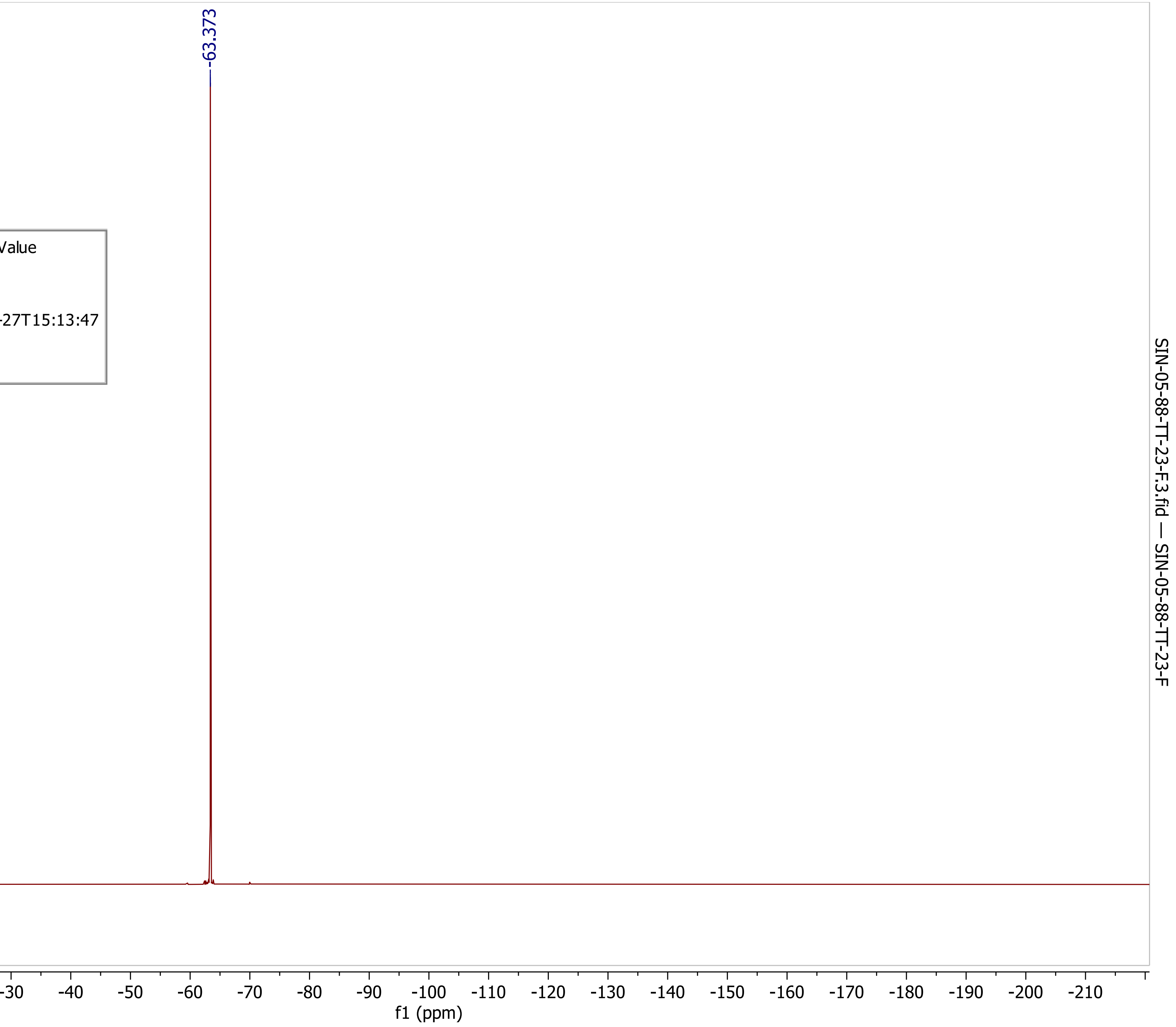

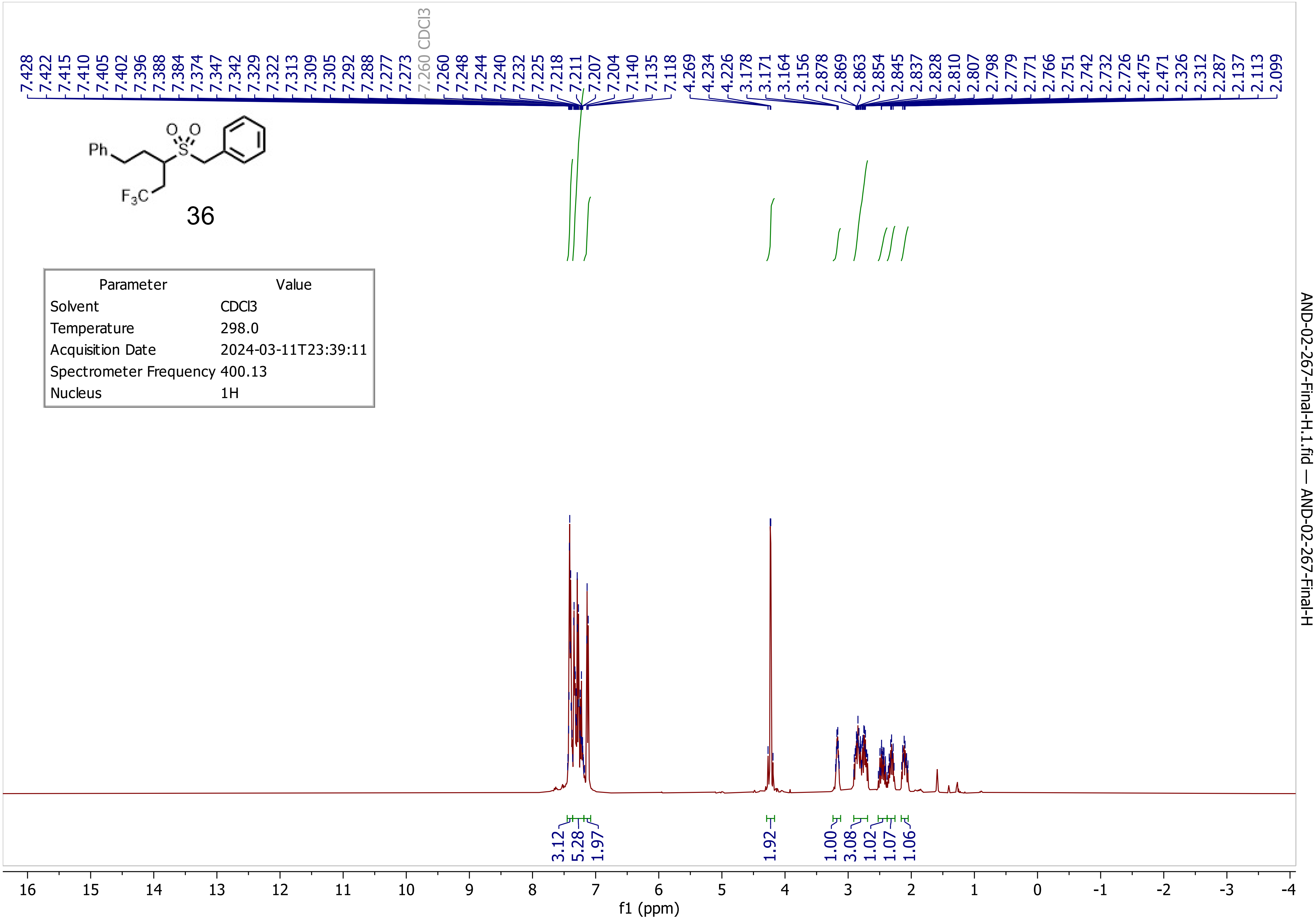

Parameter	Value
Solvent	CDCl3
Temperature	298.0
Acquisition Date	2024-03-11T23:39:11
Spectrometer Frequency	400.13
Nucleus	1H

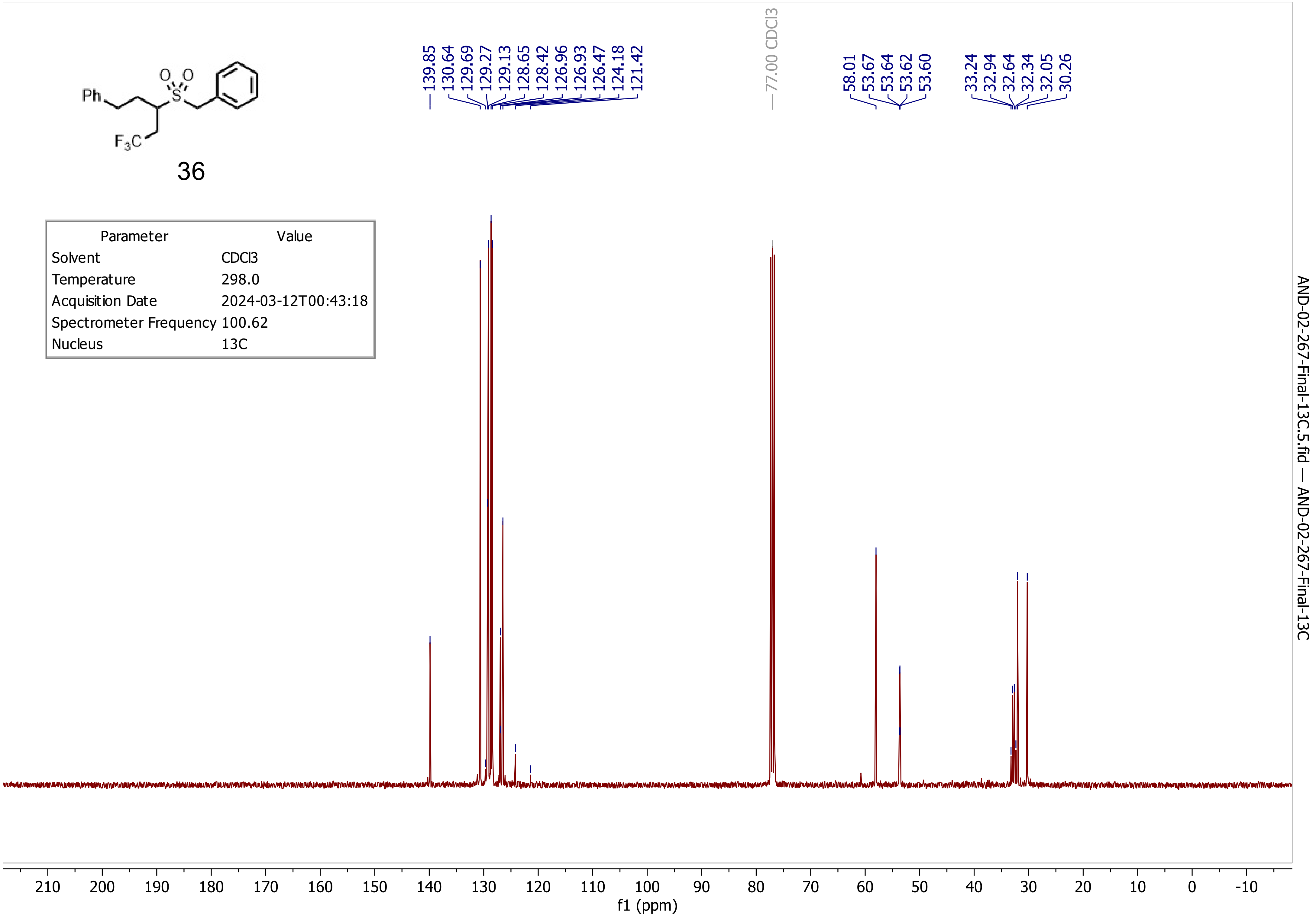
36
Parameter Value
Solvent CDCl3
Temperature 298.0
Acquisition Date 2024-03-12T00:43:18
Spectrometer Frequency 100.62
Nucleus 13C
139.85
130.64
129.69
129.27
129.13
128.65
128.42
126.96
126.93
126.47
124.18
121.42
77.00 CDCl3
58.01
53.67
53.64
53.62
53.60
33.24
32.94
32.64
32.34
32.05
30.26
f1 (ppm)
AND-02-267-Final-13C.5.fid — AND-02-267-Final-13C

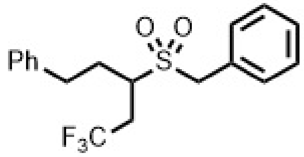

36

Parameter	Value
Solvent	CDCl3
Temperature	298.0
Acquisition Date	2024-03-11T23:42:56
Spectrometer Frequency	376.46
Nucleus	19F

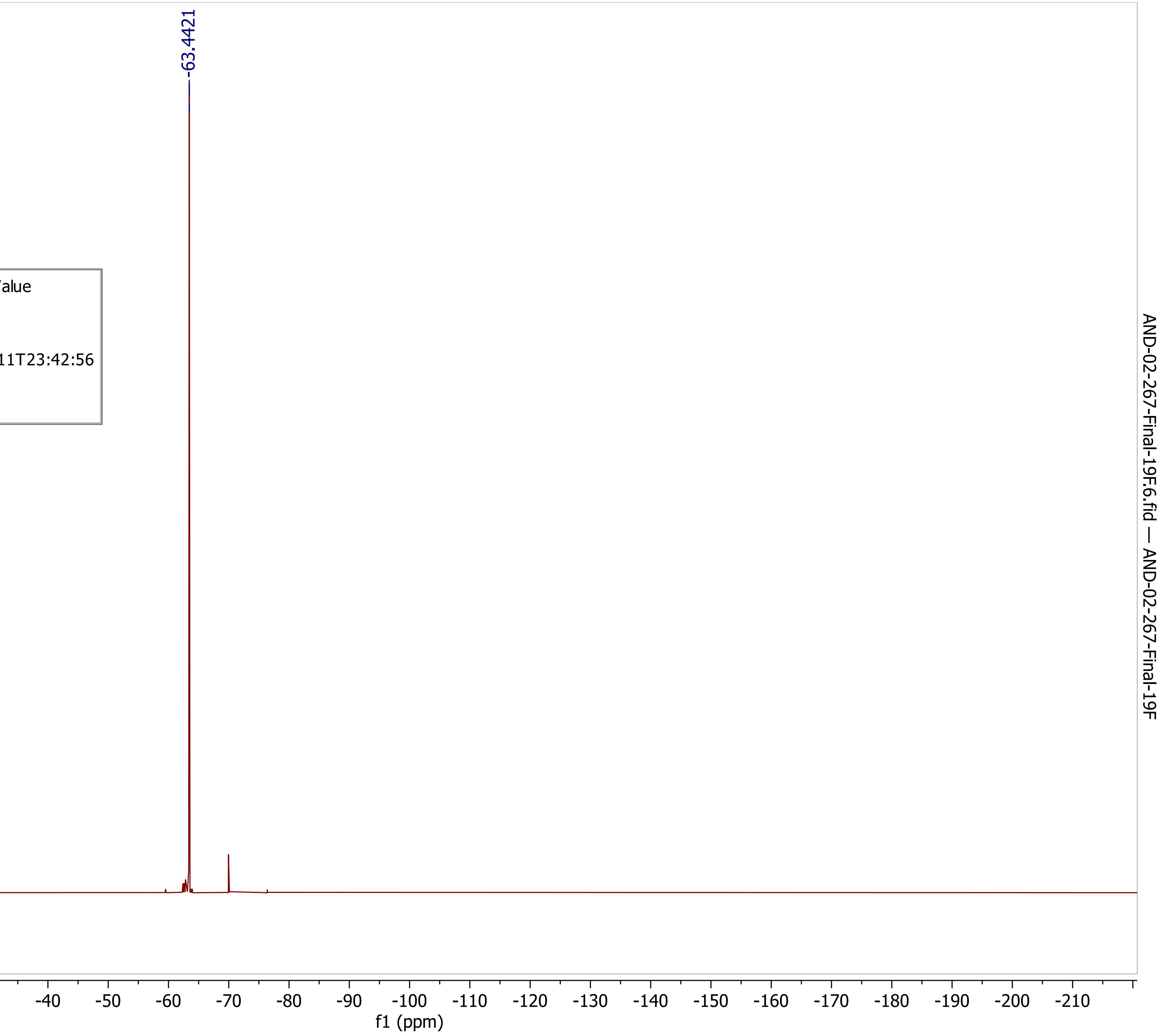

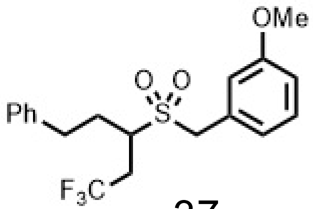

37

Parameter	Value
Solvent	CDCl3
Temperature	298.0
Acquisition Date	2024-05-10T12:29:30
Spectrometer Frequency	400.13
Nucleus	1H

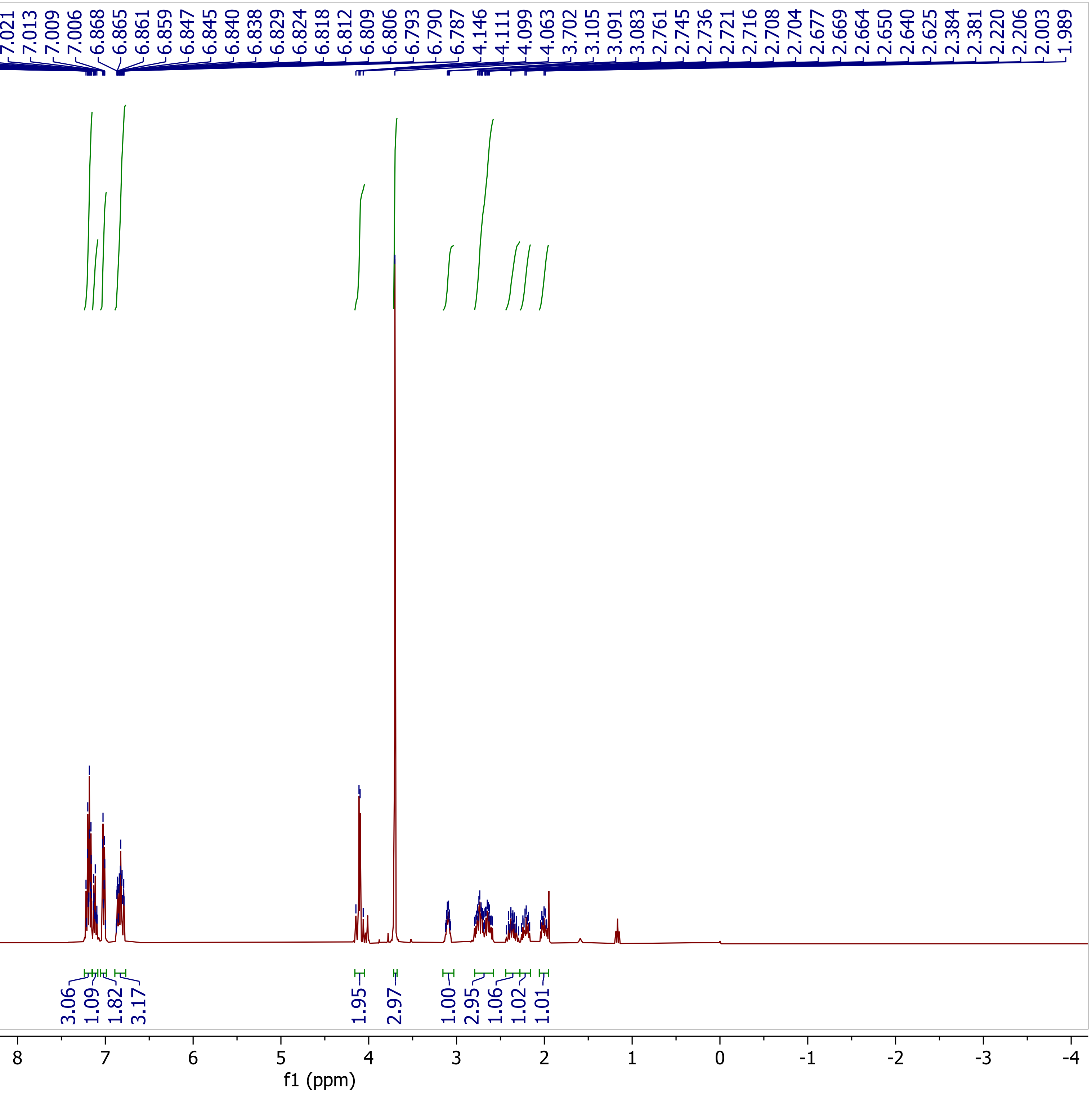

SIN-HS-5-195-3-33tt.1.fid — SIN-HS-5-195-3-33tt

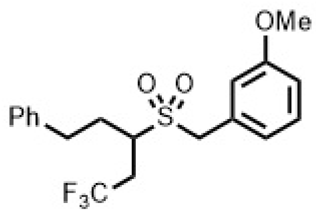

37

Parameter	Value
Solvent	CDCl3
Temperature	298.0
Acquisition Date	2024-05-10T23:54:57
Spectrometer Frequency	100.62
Nucleus	13C

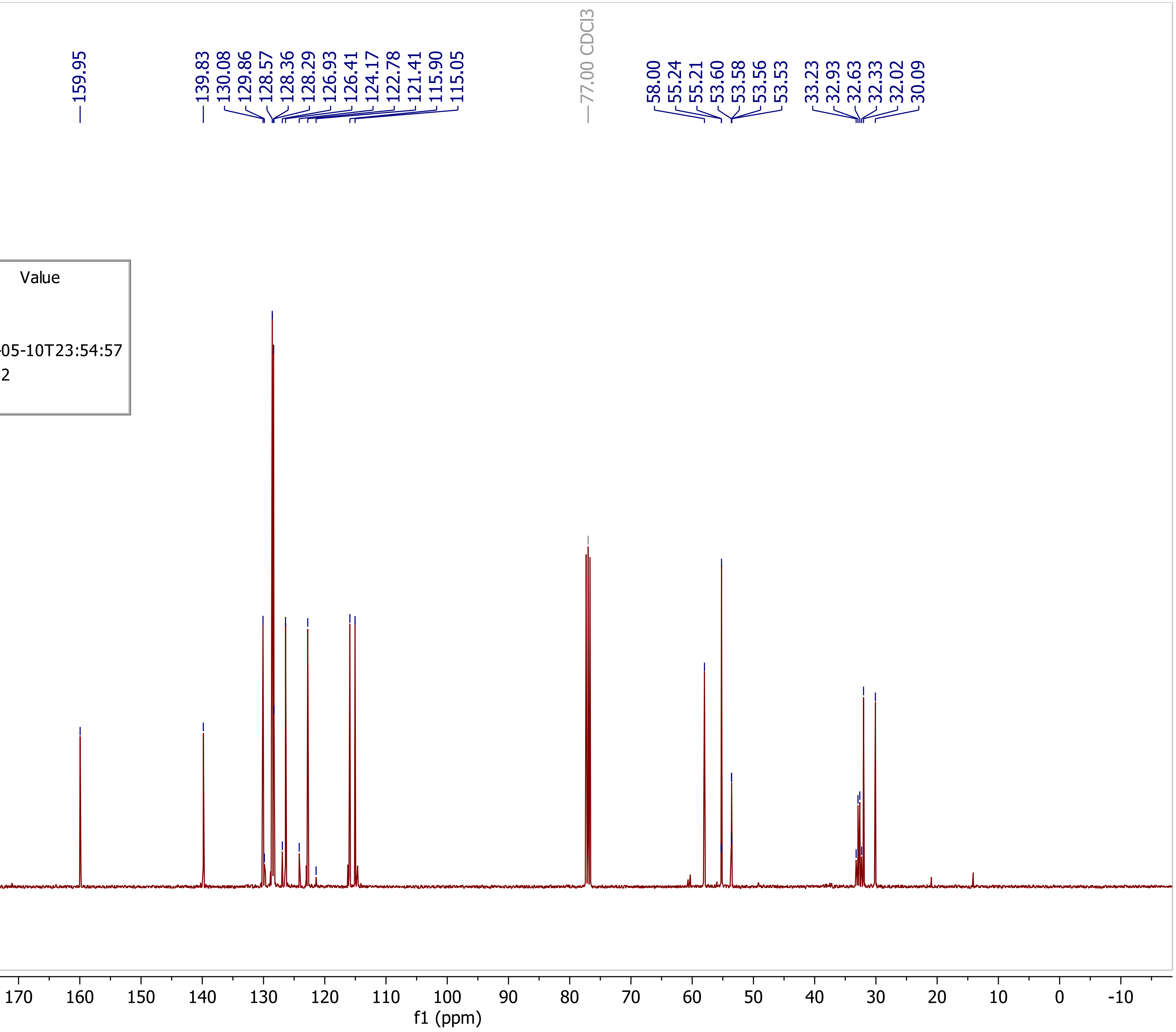

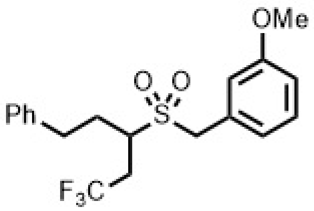

37

Parameter	Value
Solvent	CDCl3
Temperature	298.0
Acquisition Date	2024-08-06T18:56:42
Spectrometer Frequency	376.46
Nucleus	19F

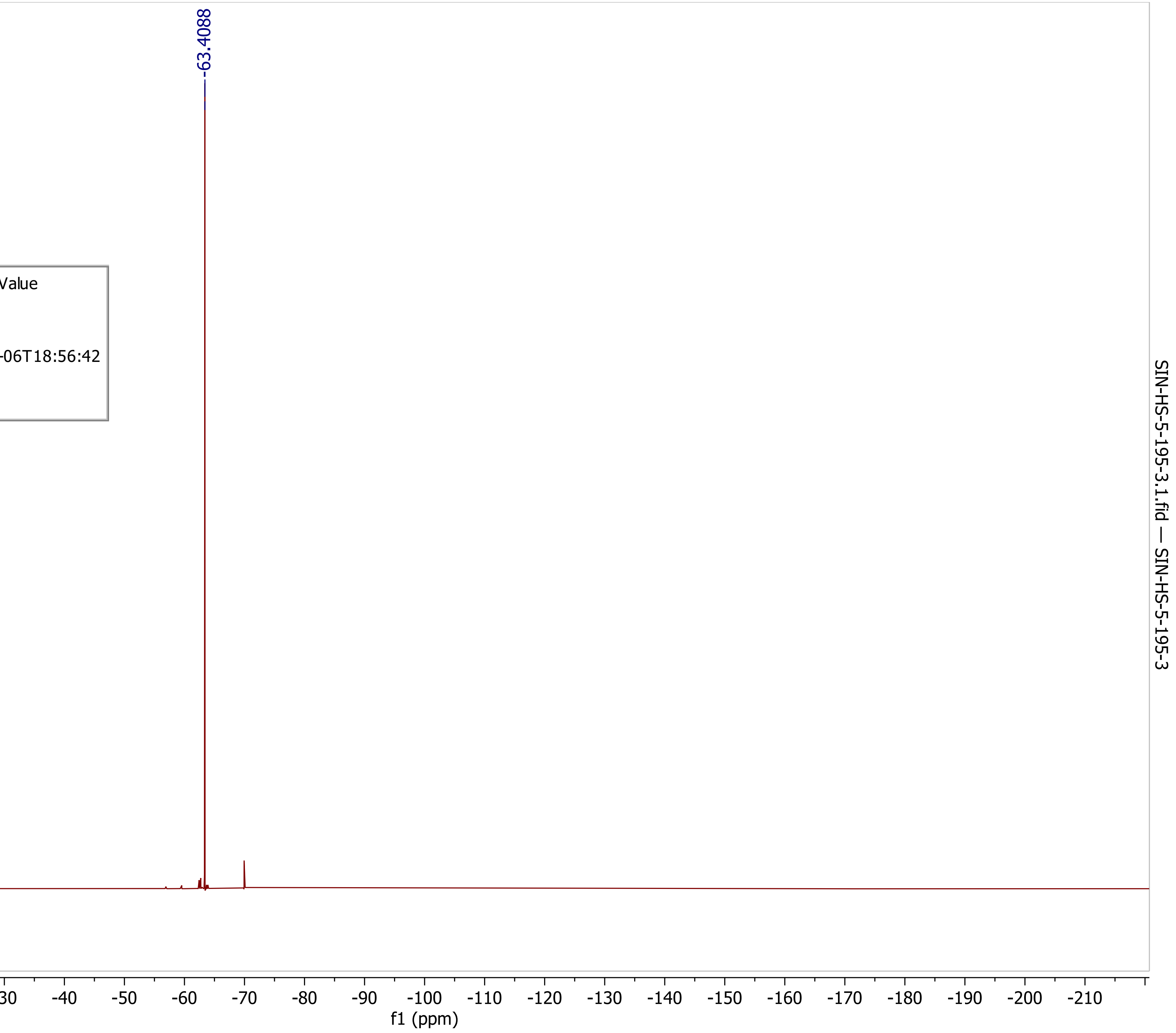

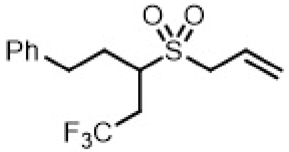

38

Parameter	Value
Solvent	CDCl3
Temperature	298.0
Acquisition Date	2024-07-10T23:25:00
Spectrometer Frequency	400.13
Nucleus	1H

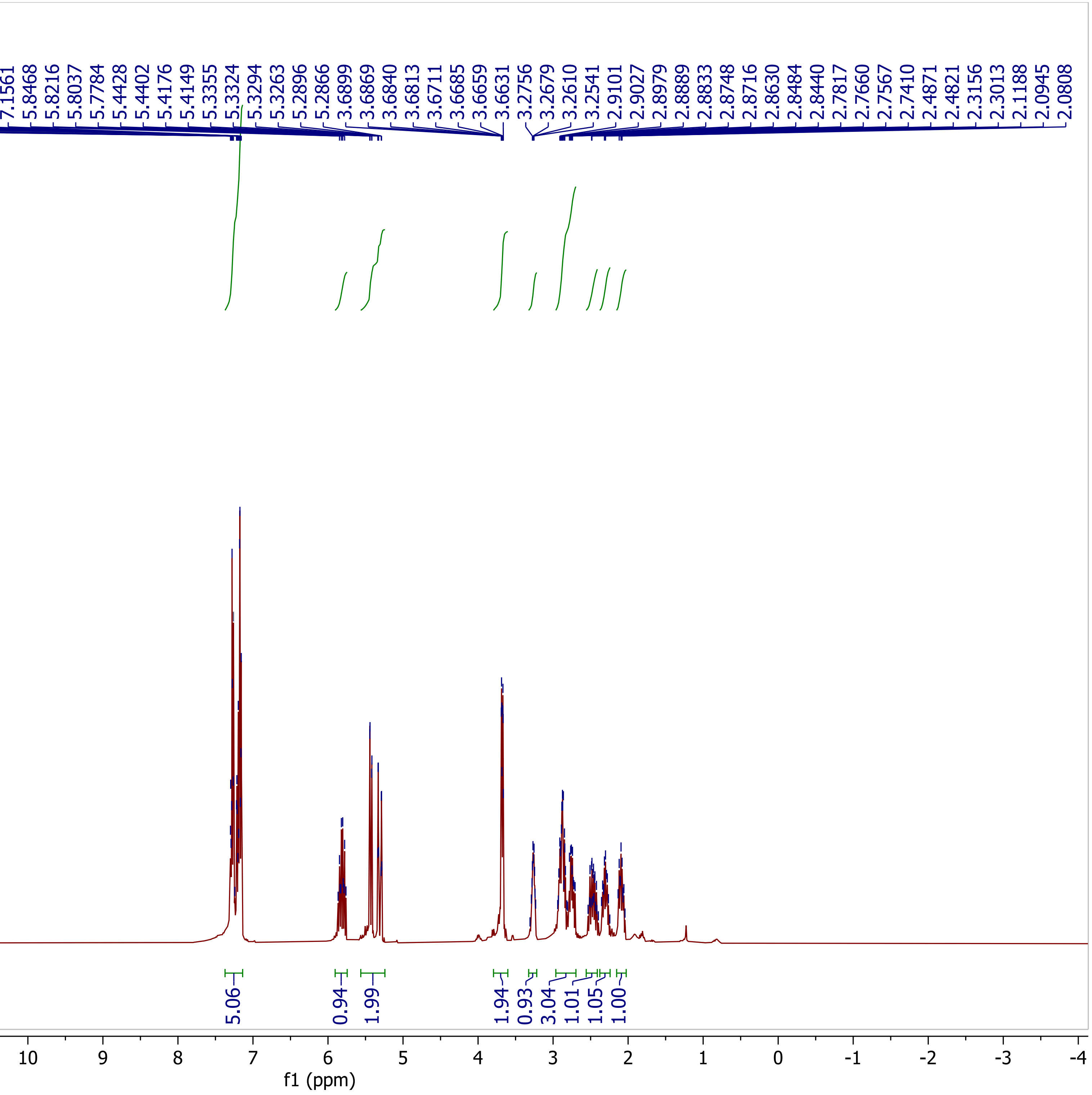

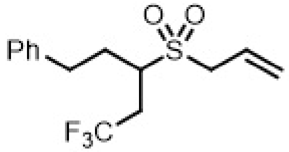

38

Parameter	Value
Solvent	CDCl3
Temperature	298.0
Acquisition Date	2024-07-10T23:48:54
Spectrometer Frequency	100.62
Nucleus	13C

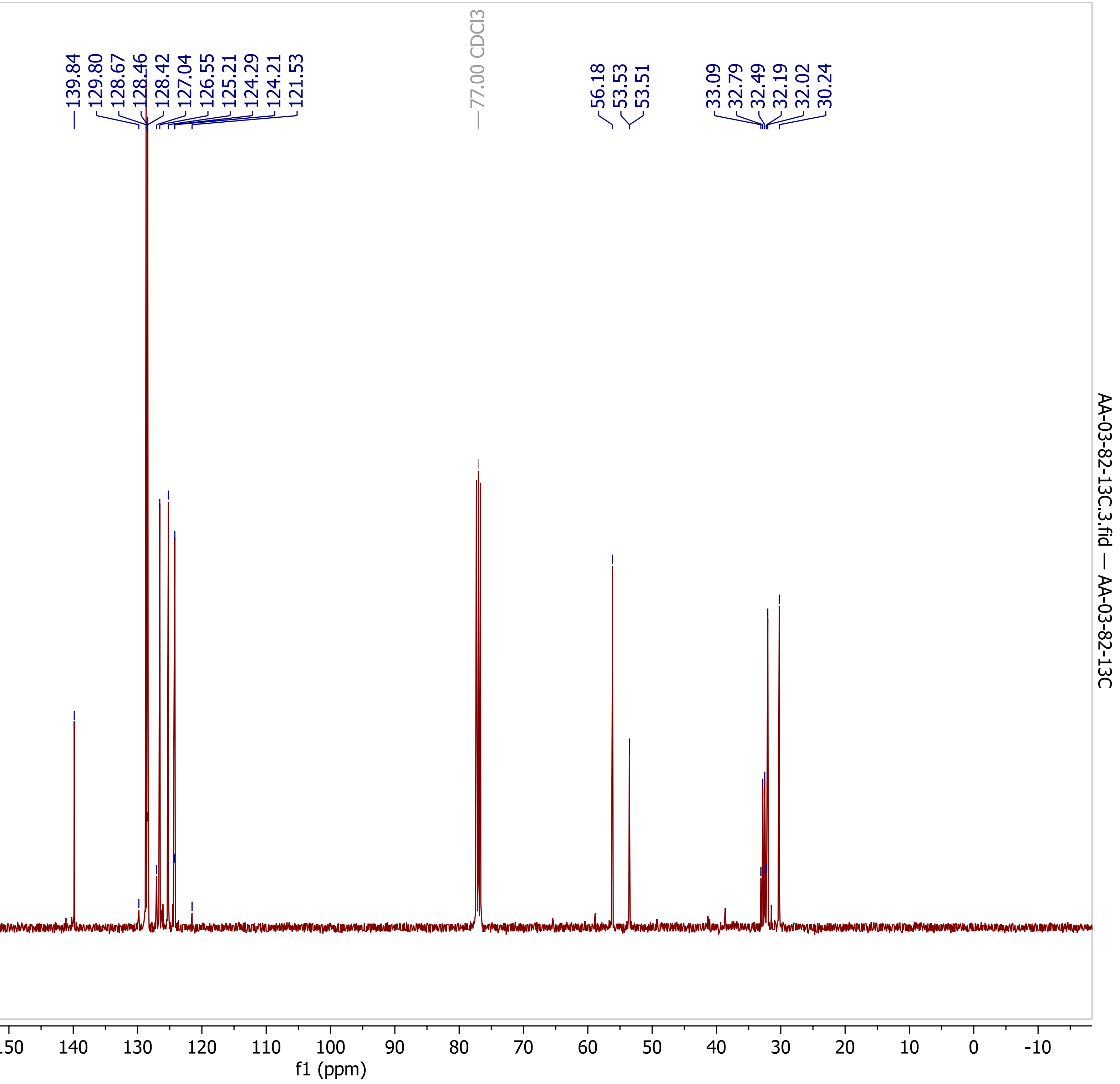

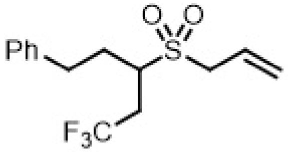

38

Parameter	Value
Solvent	CDCl3
Temperature	298.0
Acquisition Date	2024-07-10T23:53:20
Spectrometer Frequency	376.46
Nucleus	19F

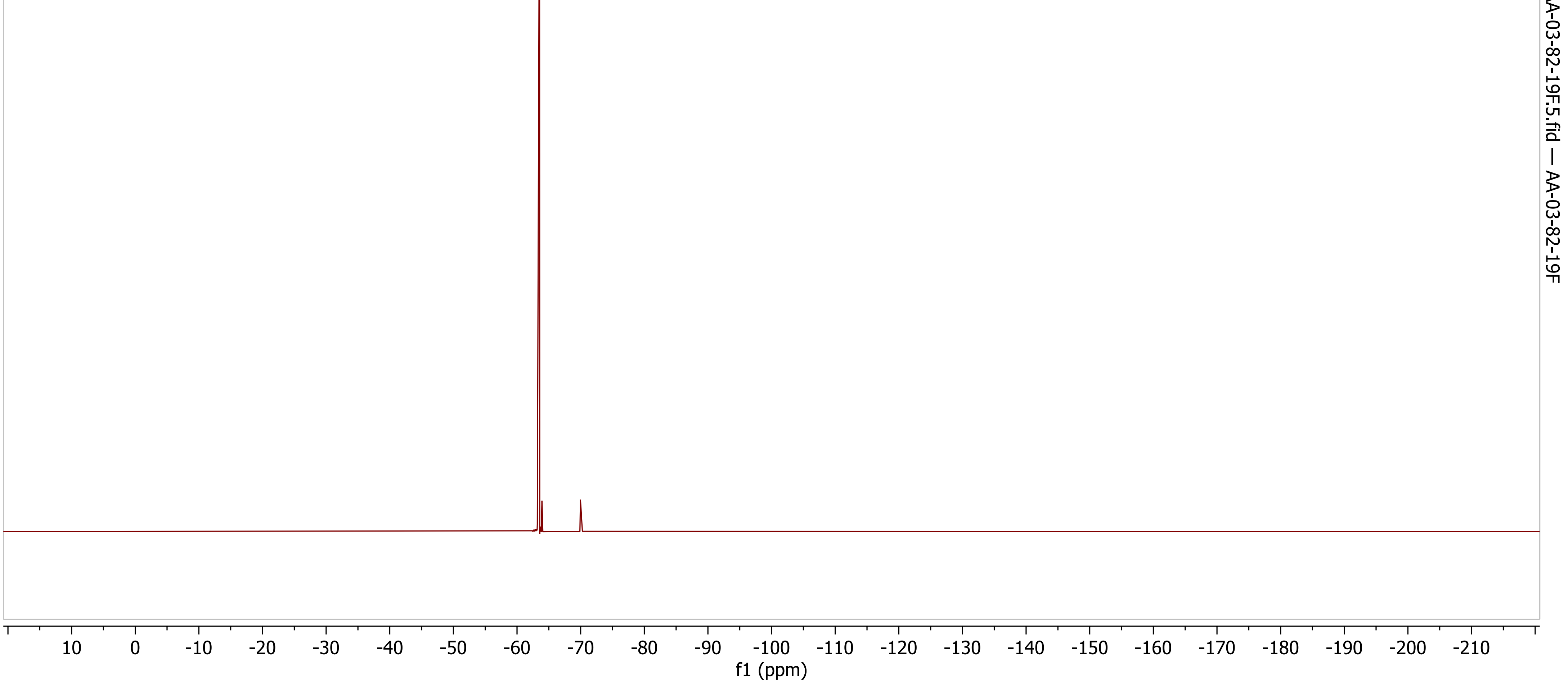

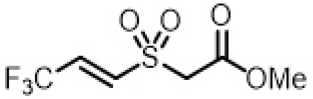

39

Parameter	Value
Solvent	CDCl3
Temperature	298.0
Acquisition Date	2024-09-02T19:38:52
Spectrometer Frequency	400.13
Nucleus	1H

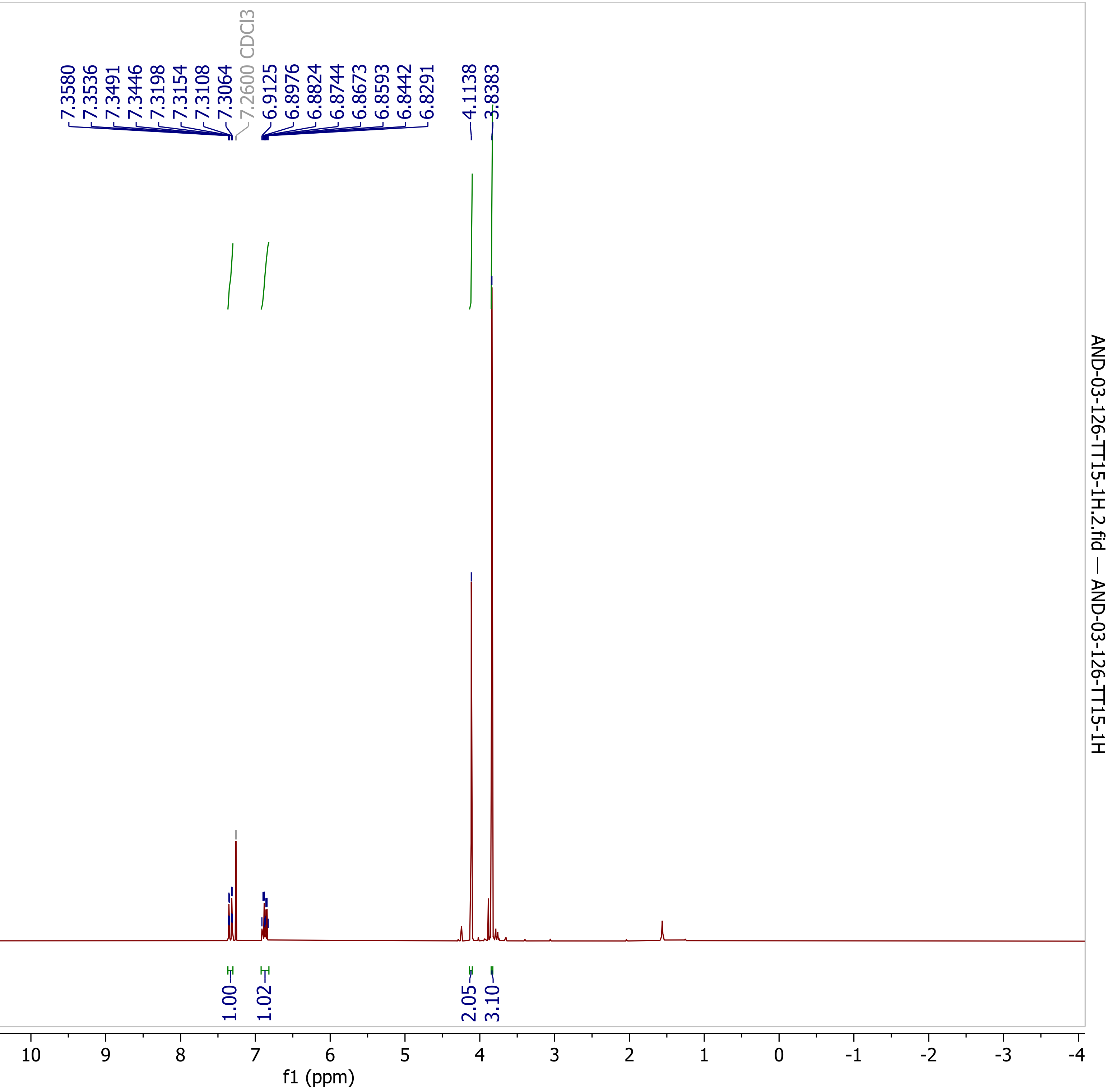

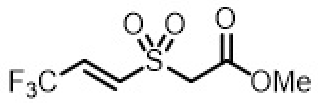

39

Parameter	Value
Solvent	CDCl3
Temperature	298.0
Acquisition Date	2024-09-05T01:12:20
Spectrometer Frequency	100.62
Nucleus	13C

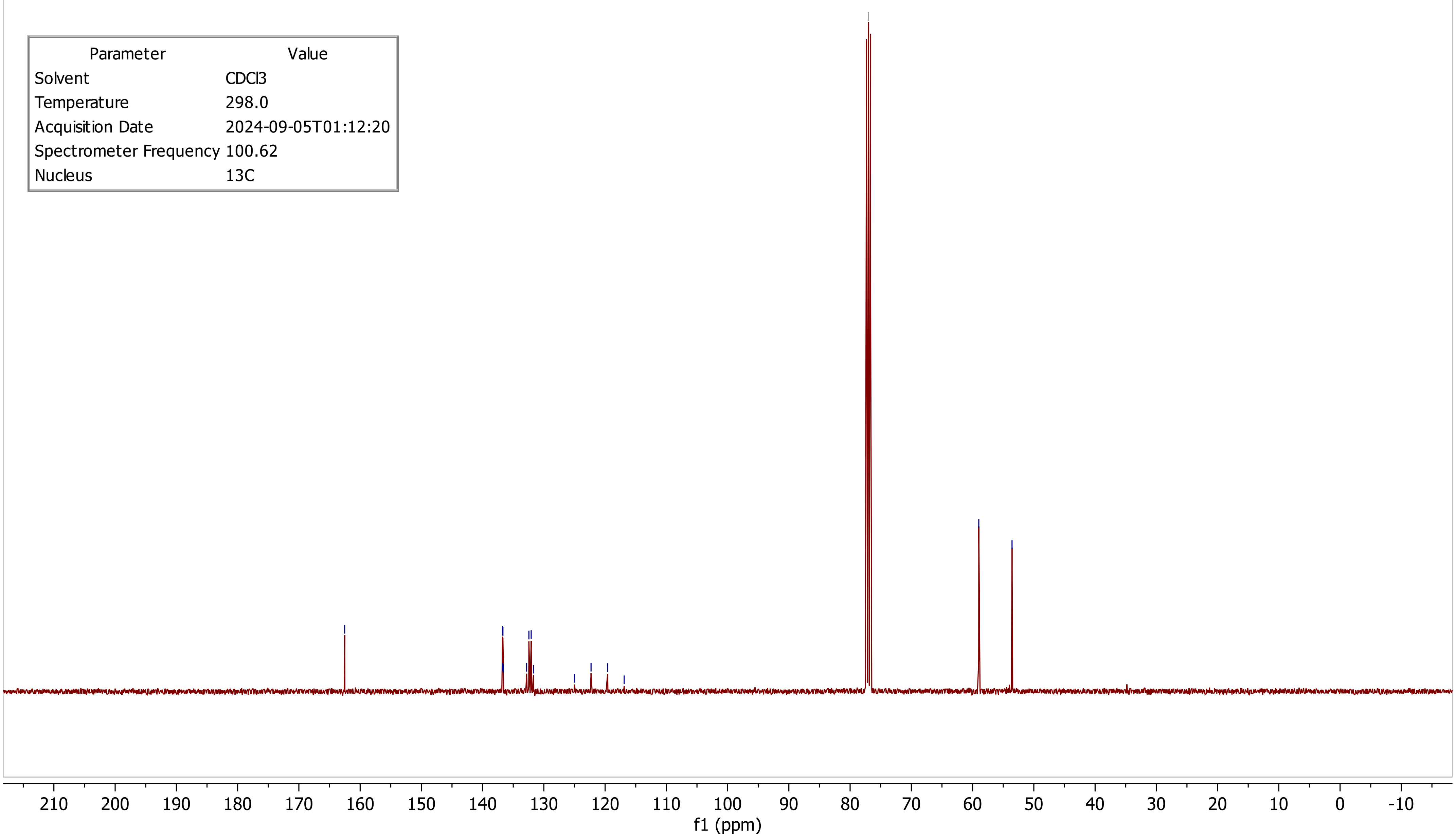

AND-03-126-TT16-13C -RE.1.fid — AND-03-126-TT16-13C -RE

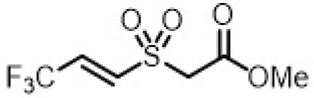

39

Parameter	Value
Solvent	CDCl3
Temperature	298.0
Acquisition Date	2024-09-03T02:45:44
Spectrometer Frequency	376.46
Nucleus	19F

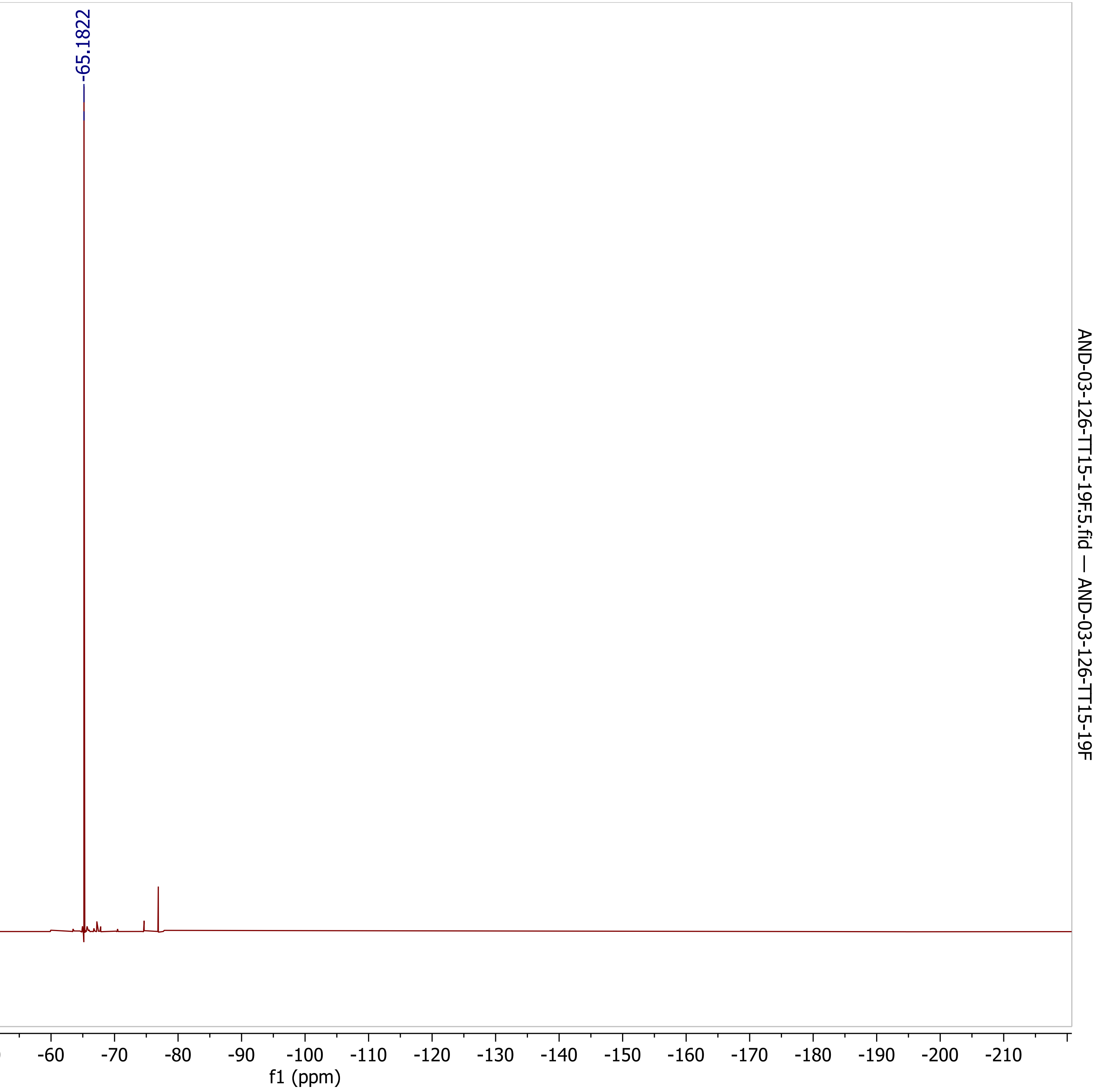

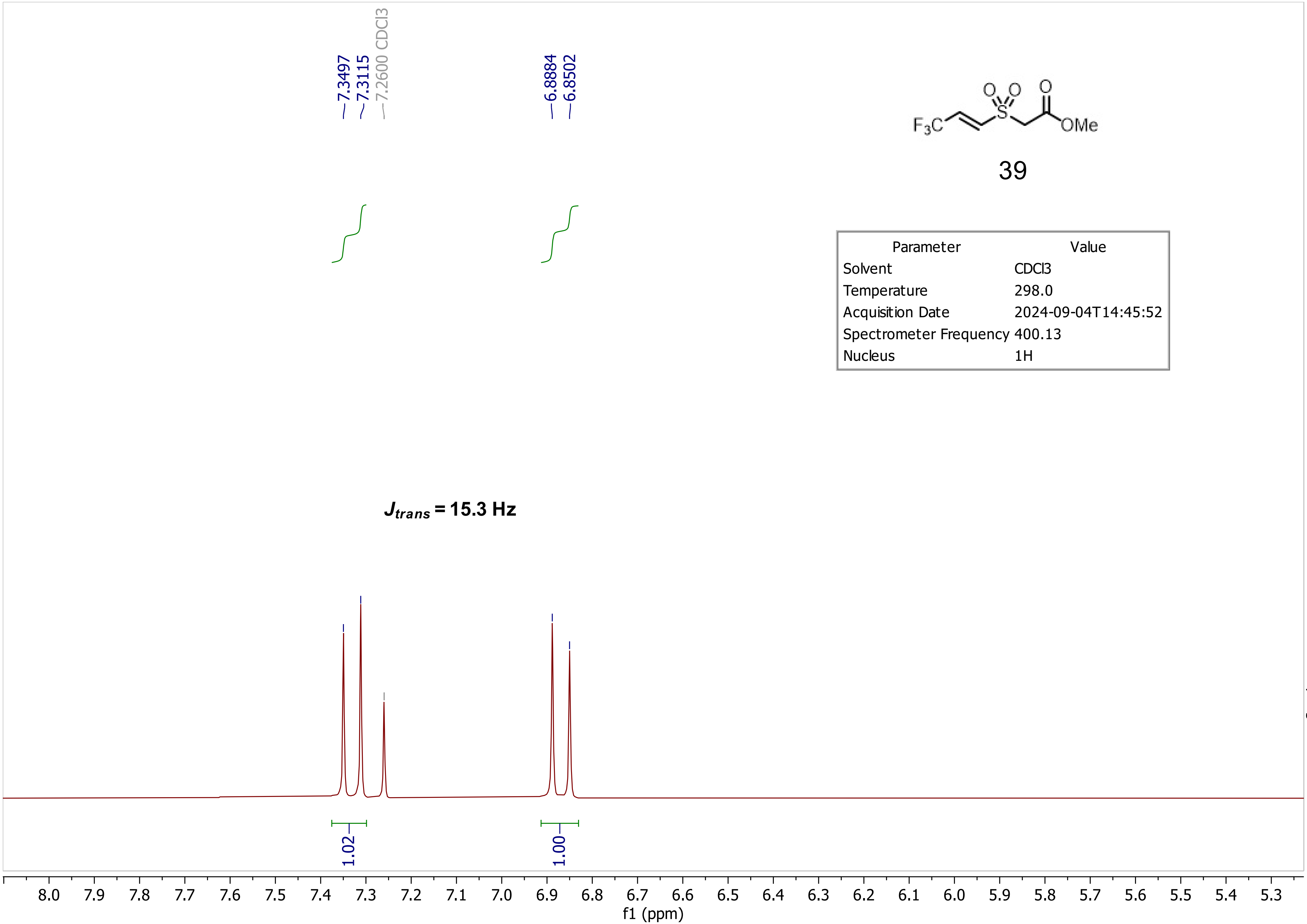

SIN-AND-03-126.3.fid — SIN-AND-03-126 — 1H NMR with 19F decoupling

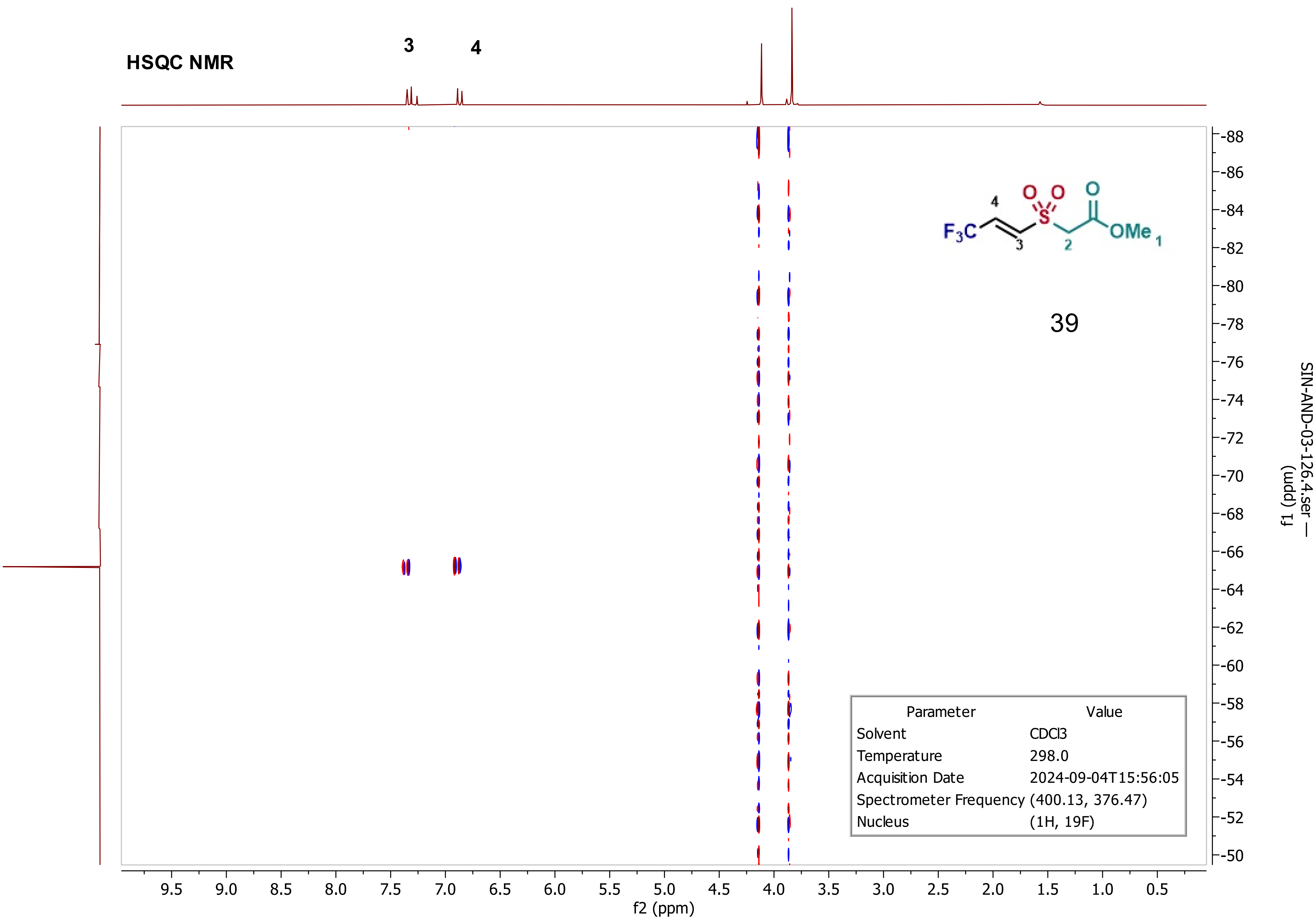

Parameter	Value
Solvent	CDCl3
Temperature	298.0
Acquisition Date	2024-09-04T15:56:05
Spectrometer Frequency	(400.13, 376.47)
Nucleus	(1H, 19F)

HSQC NMR

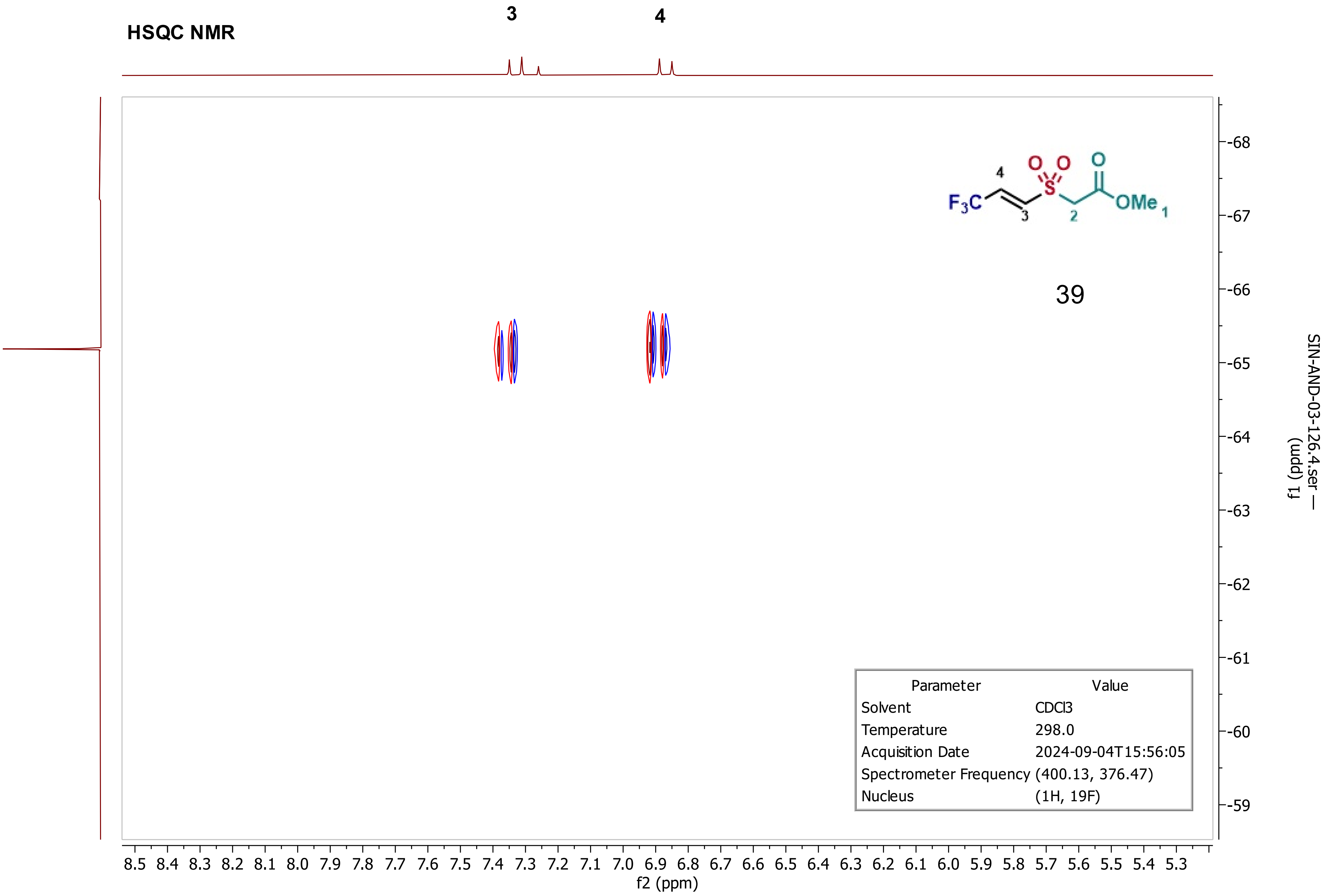

Parameter	Value
Solvent	CDCl3
Temperature	298.0
Acquisition Date	2024-09-04T15:56:05
Spectrometer Frequency	(400.13, 376.47)
Nucleus	(1H, 19F)

40

Parameter	Value
Solvent	CDCl3
Temperature	298.0
Acquisition Date	2024-09-02T19:33:40
Spectrometer Frequency	400.13
Nucleus	1H

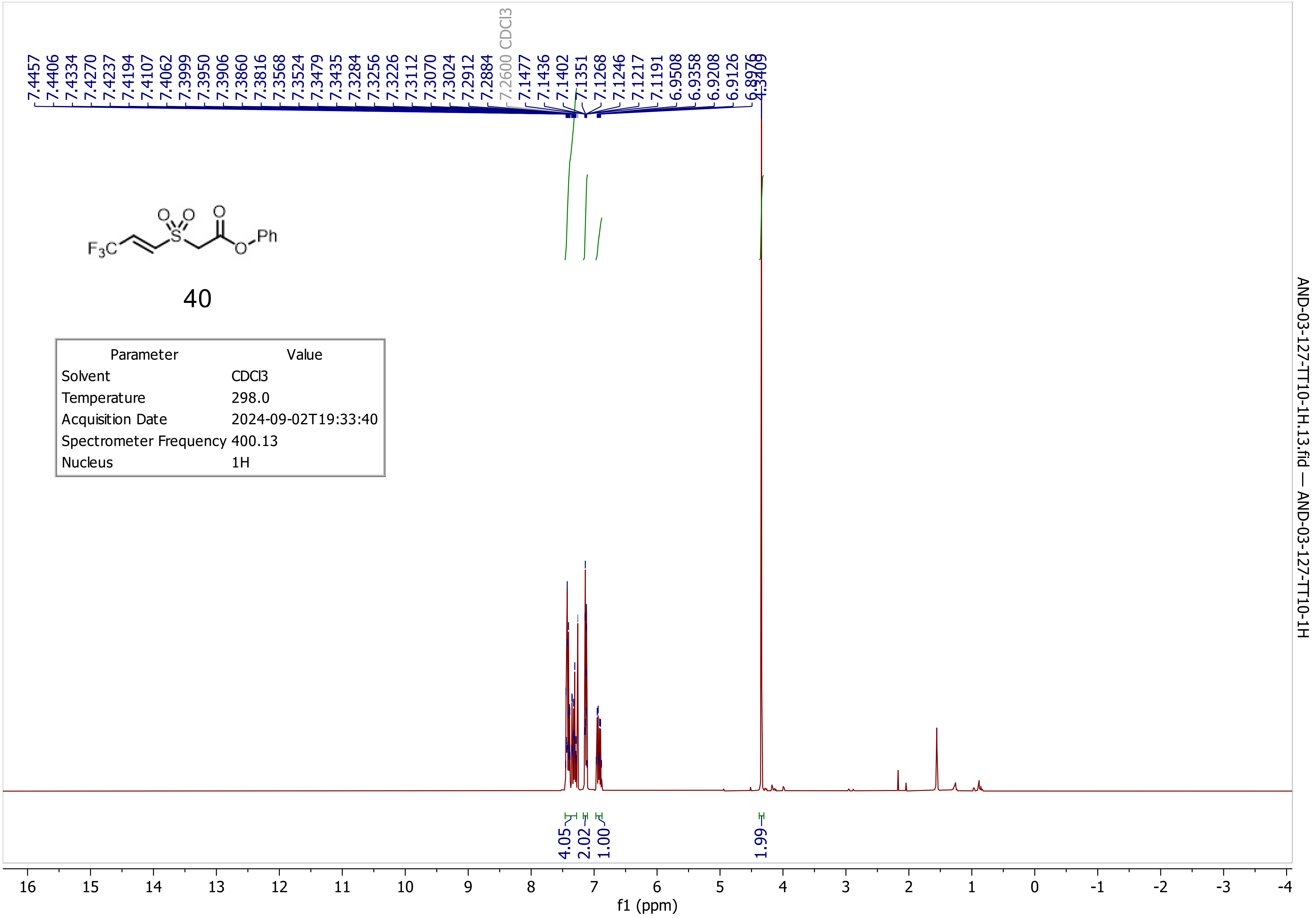

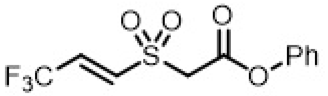

40

Parameter	Value
Solvent	CDCl3
Temperature	298.0
Acquisition Date	2024-09-05T02:16:35
Spectrometer Frequency	100.62
Nucleus	13C

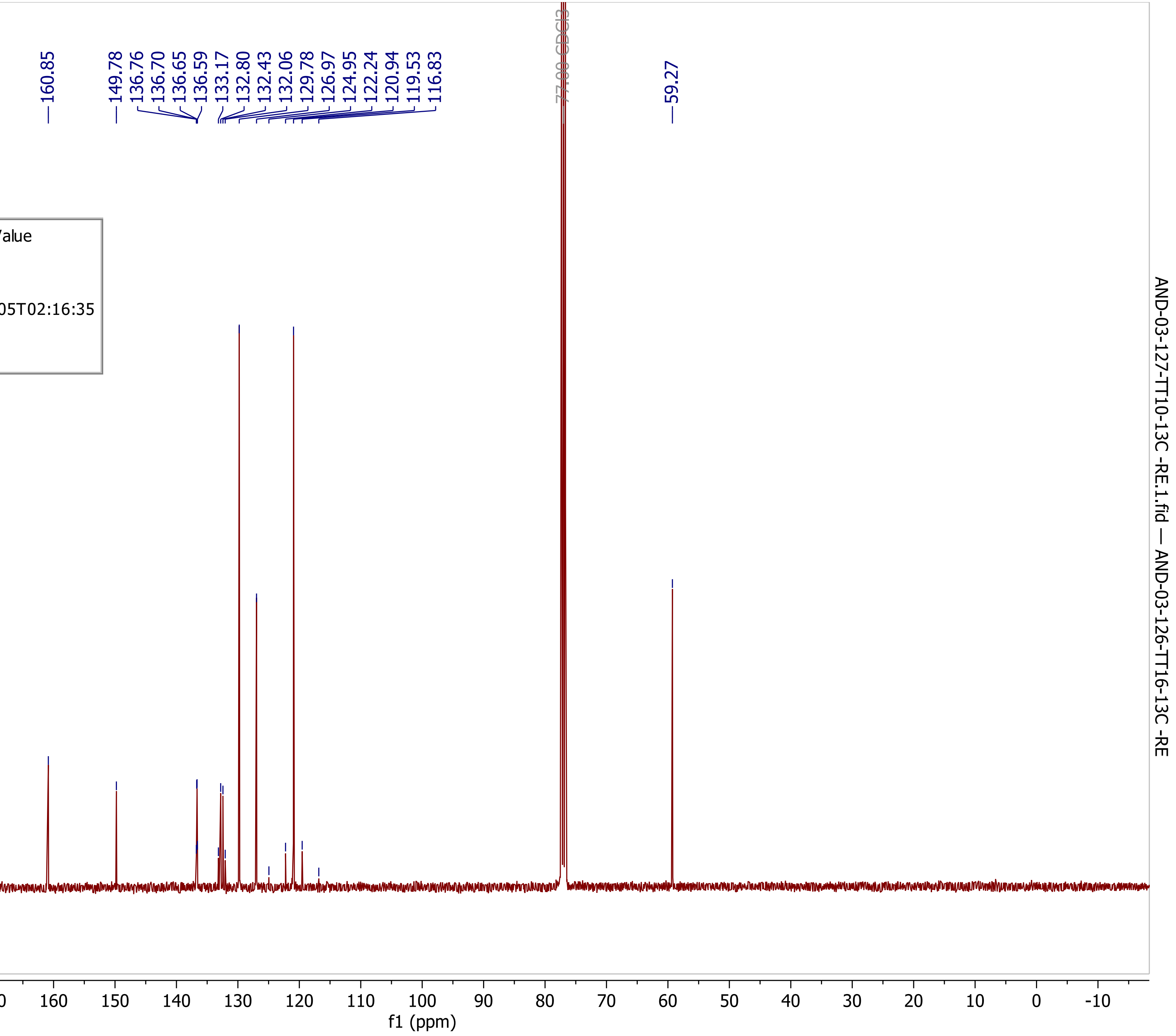

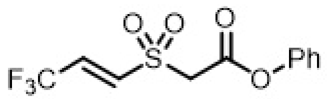

40

Parameter	Value
Solvent	CDCl3
Temperature	298.0
Acquisition Date	2024-09-03T02:39:27
Spectrometer Frequency	376.46
Nucleus	19F

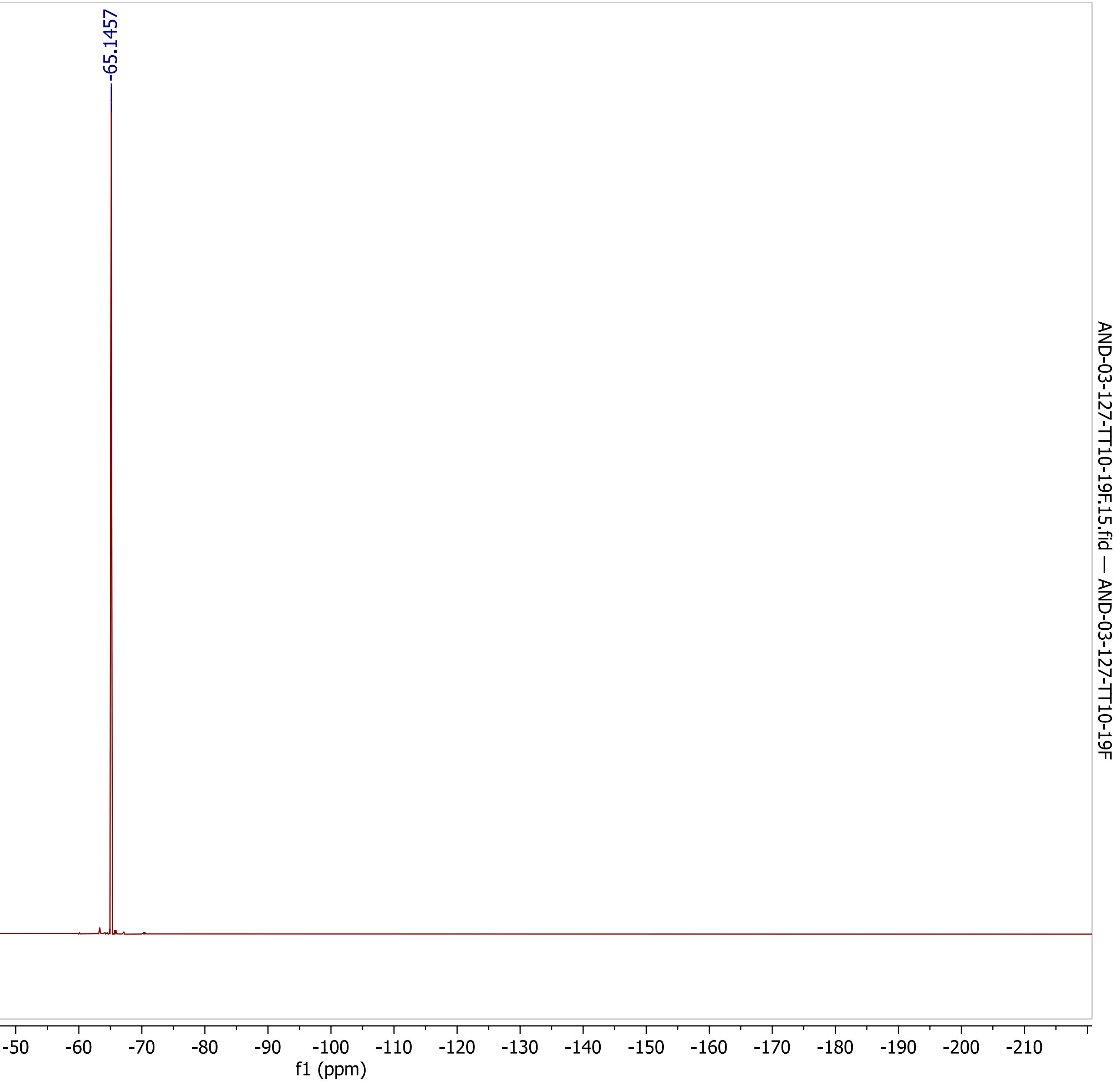

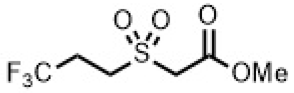

41

Parameter	Value
Solvent	CDCl3
Temperature	298.0
Acquisition Date	2024-05-03T17:59:02
Spectrometer Frequency	400.13
Nucleus	1H

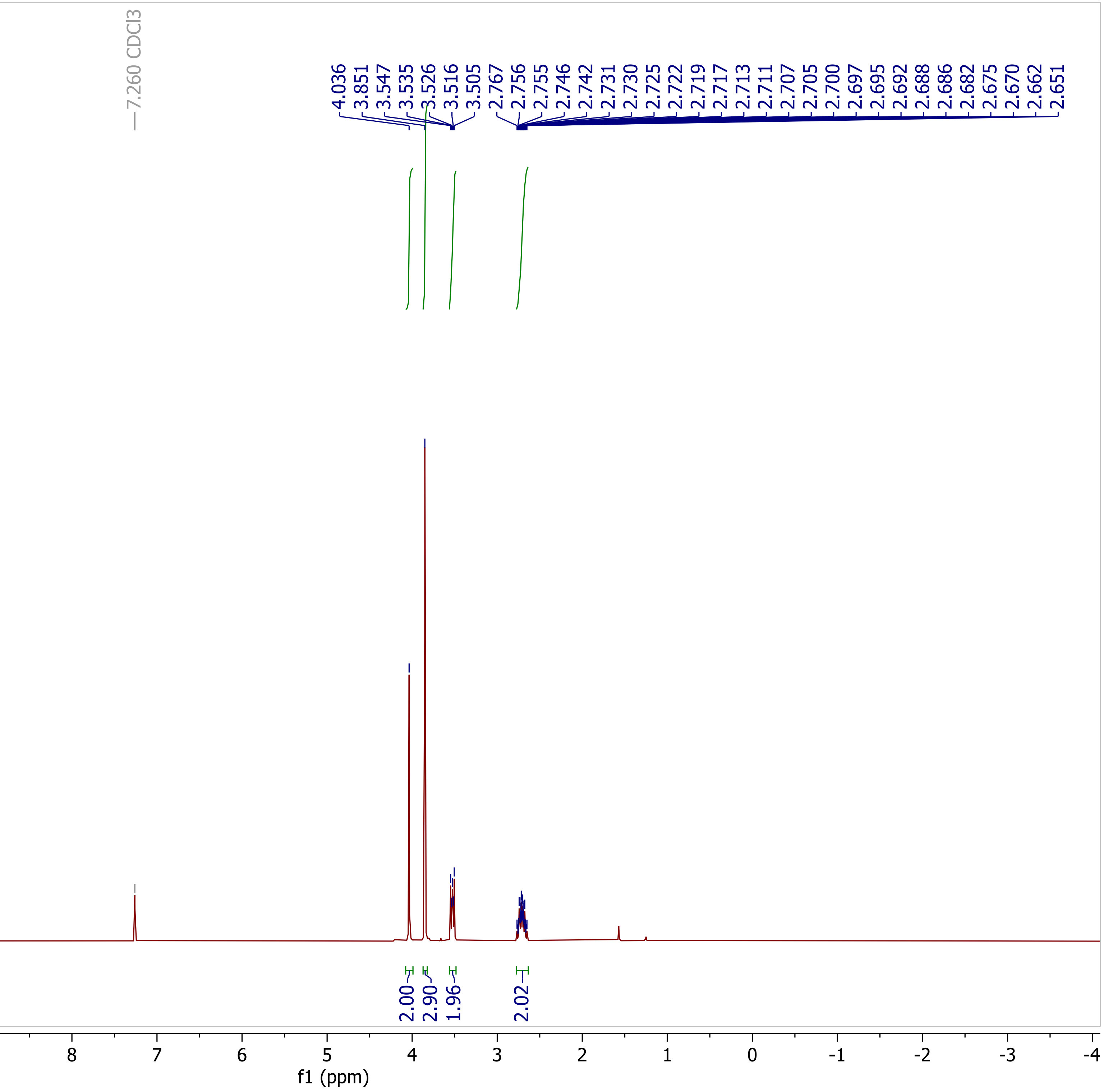

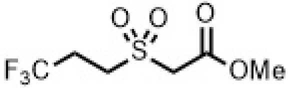

41

Parameter	Value
Solvent	CDCl3
Temperature	298.0
Acquisition Date	2024-05-03T05:17:03
Spectrometer Frequency	100.62
Nucleus	13C

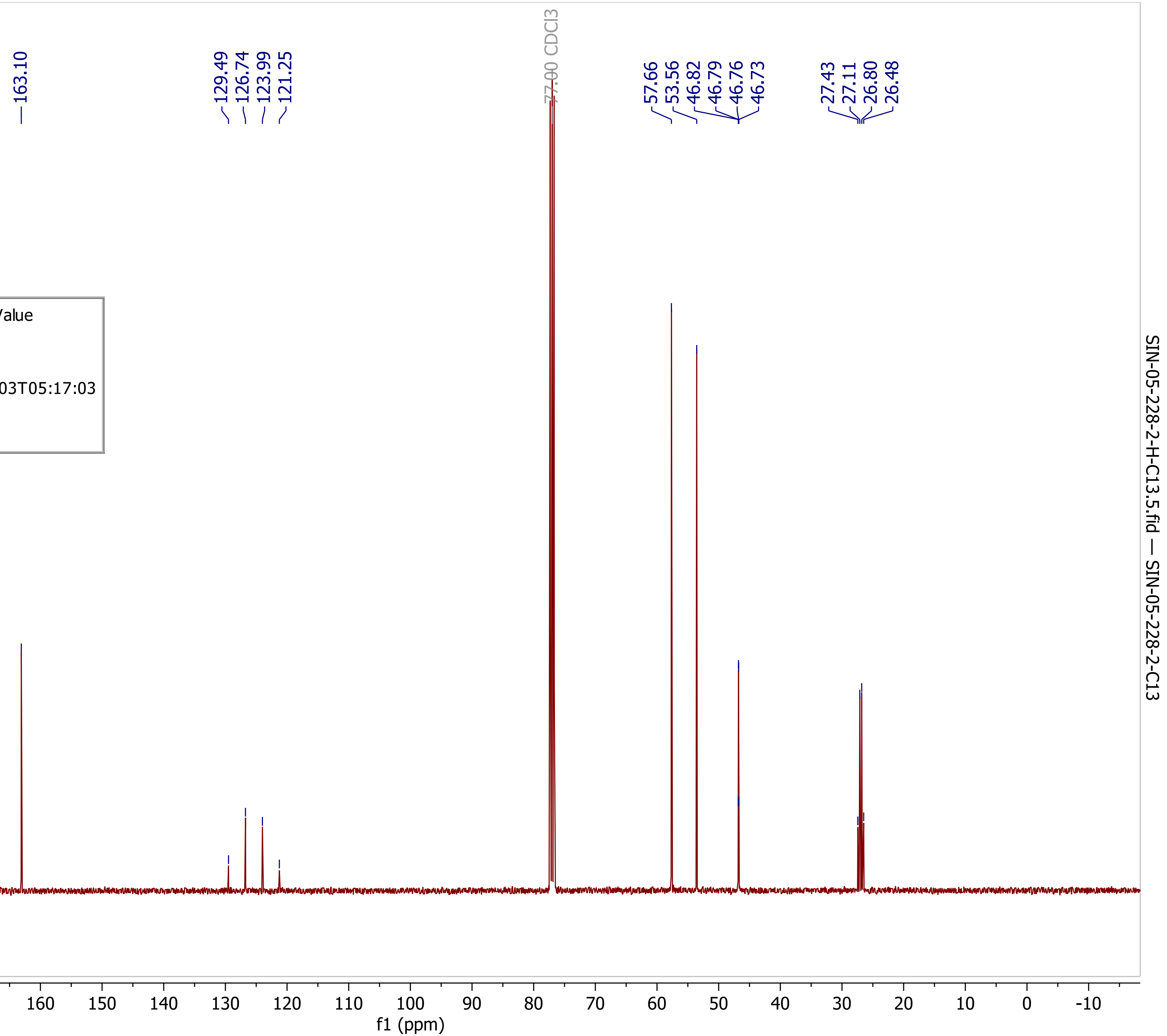

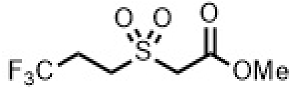

41

Parameter	Value
Solvent	CDCl3
Temperature	298.0
Acquisition Date	2024-05-02T21:07:28
Spectrometer Frequency	376.46
Nucleus	19F

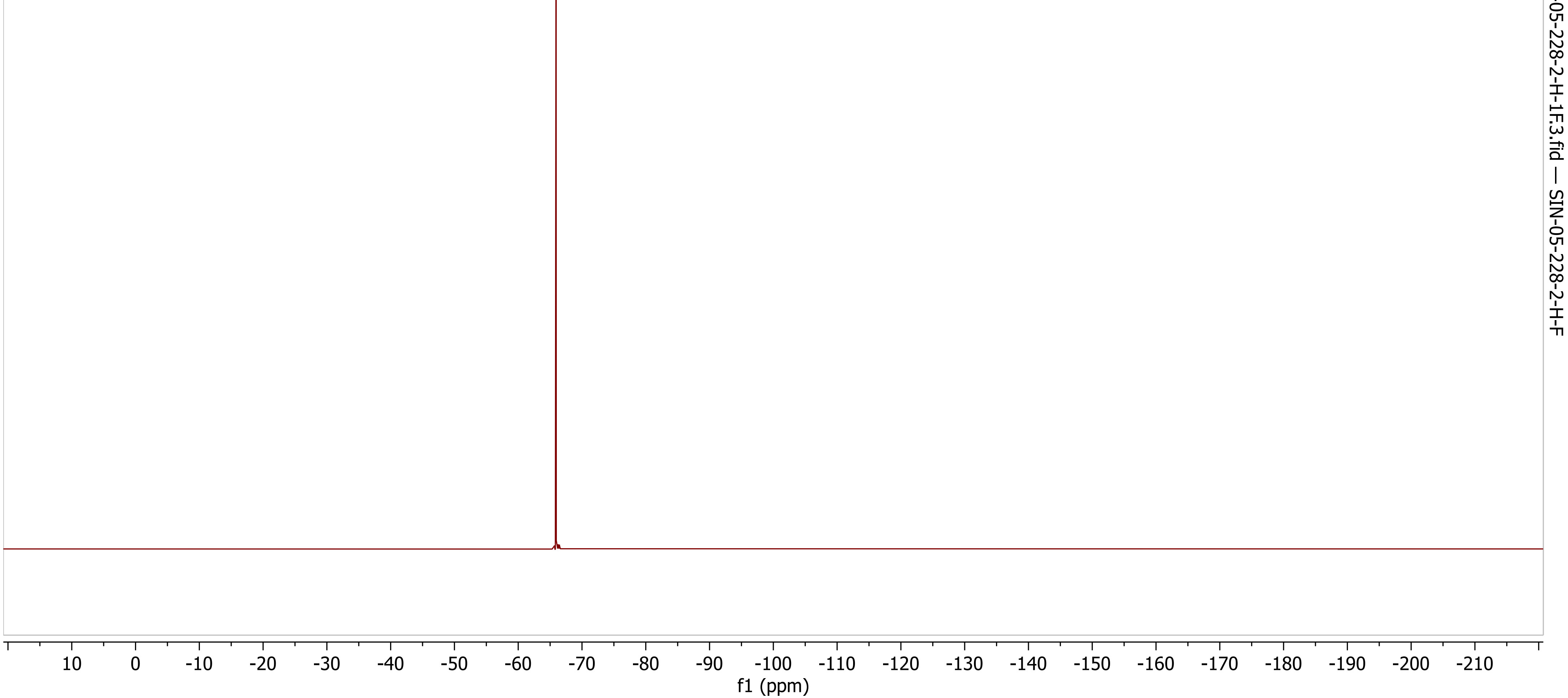

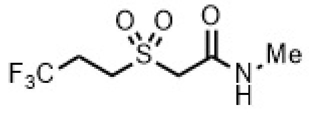

42

Parameter	Value
Solvent	Acetone
Temperature	298.0
Acquisition Date	2024-08-27T18:33:00
Spectrometer Frequency	400.13
Nucleus	1H

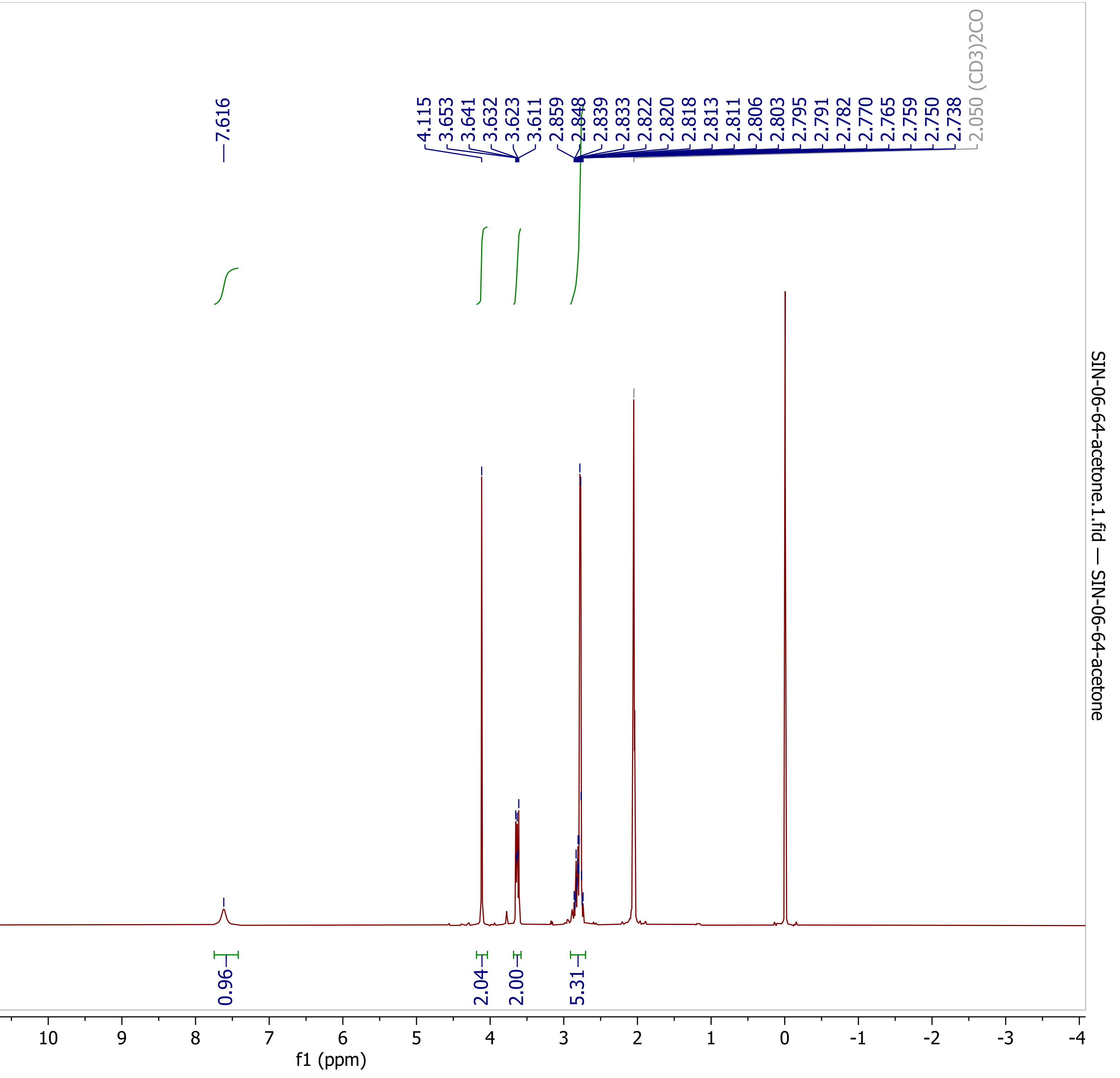

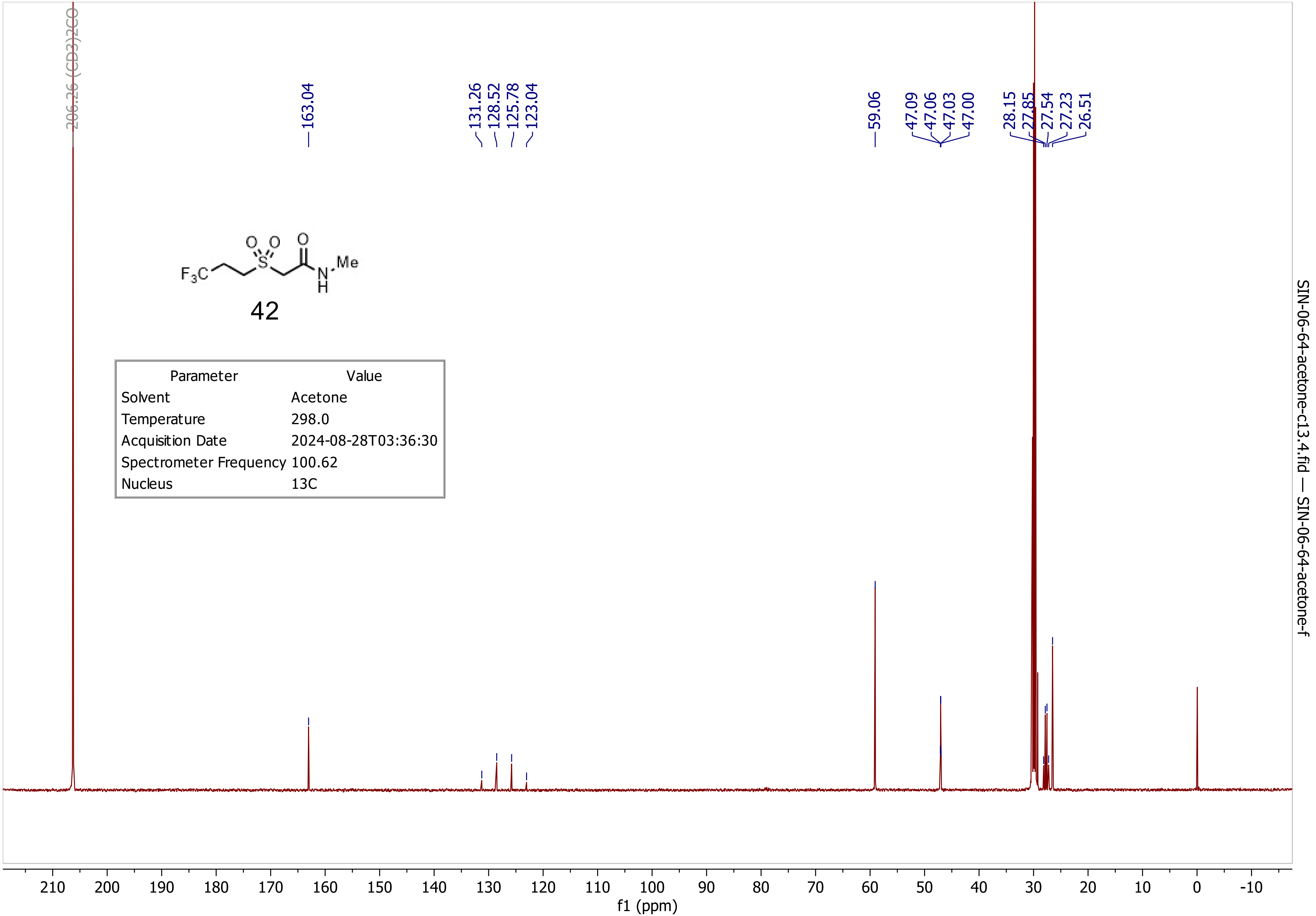

42

Parameter	Value
Solvent	Acetone
Temperature	298.0
Acquisition Date	2024-08-28T03:36:30
Spectrometer Frequency	100.62
Nucleus	13C

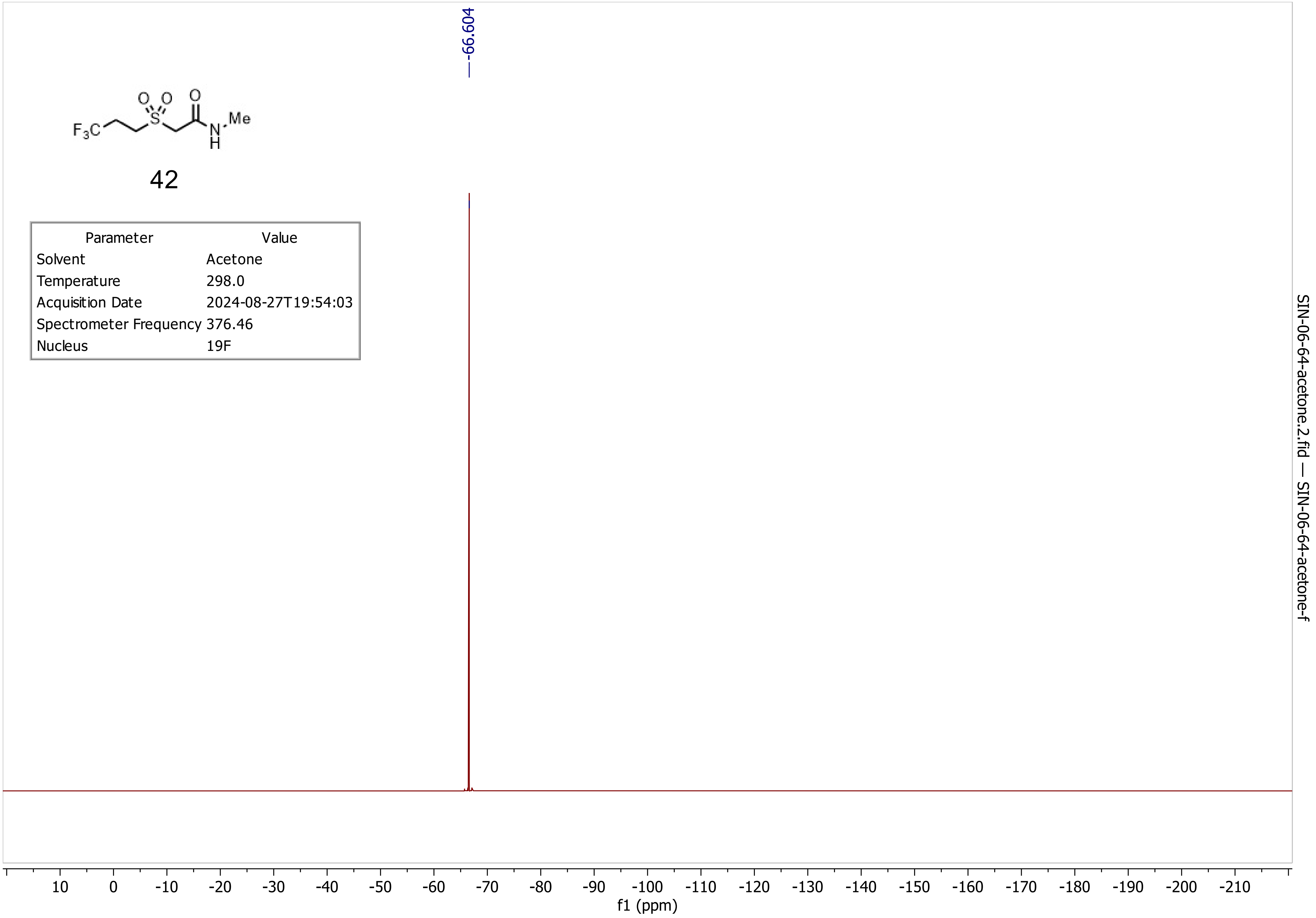

42

Parameter	Value
Solvent	Acetone
Temperature	298.0
Acquisition Date	2024-08-27T19:54:03
Spectrometer Frequency	376.46
Nucleus	19F

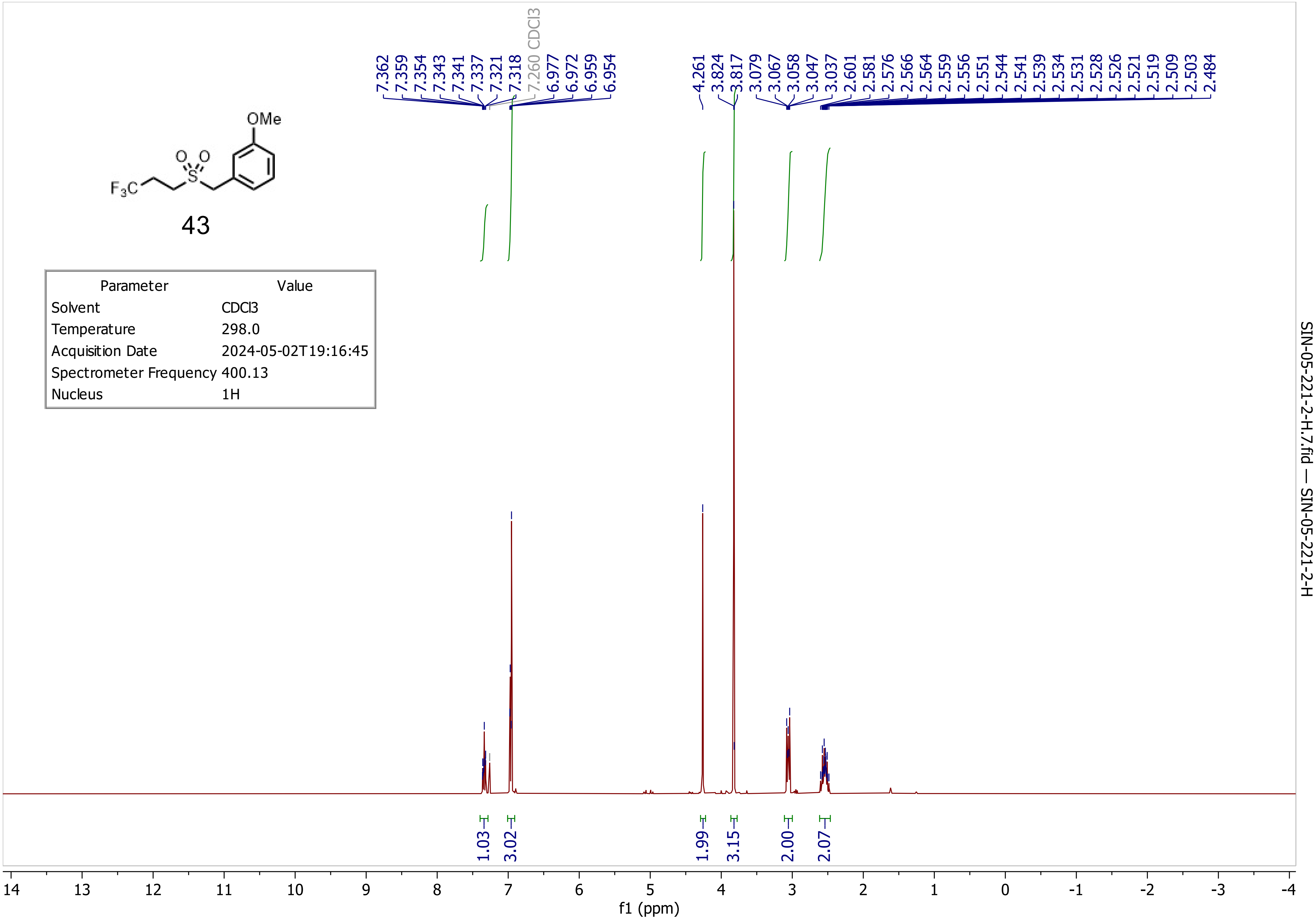

Parameter	Value
Solvent	CDCl3
Temperature	298.0
Acquisition Date	2024-05-02T19:16:45
Spectrometer Frequency	400.13
Nucleus	1H

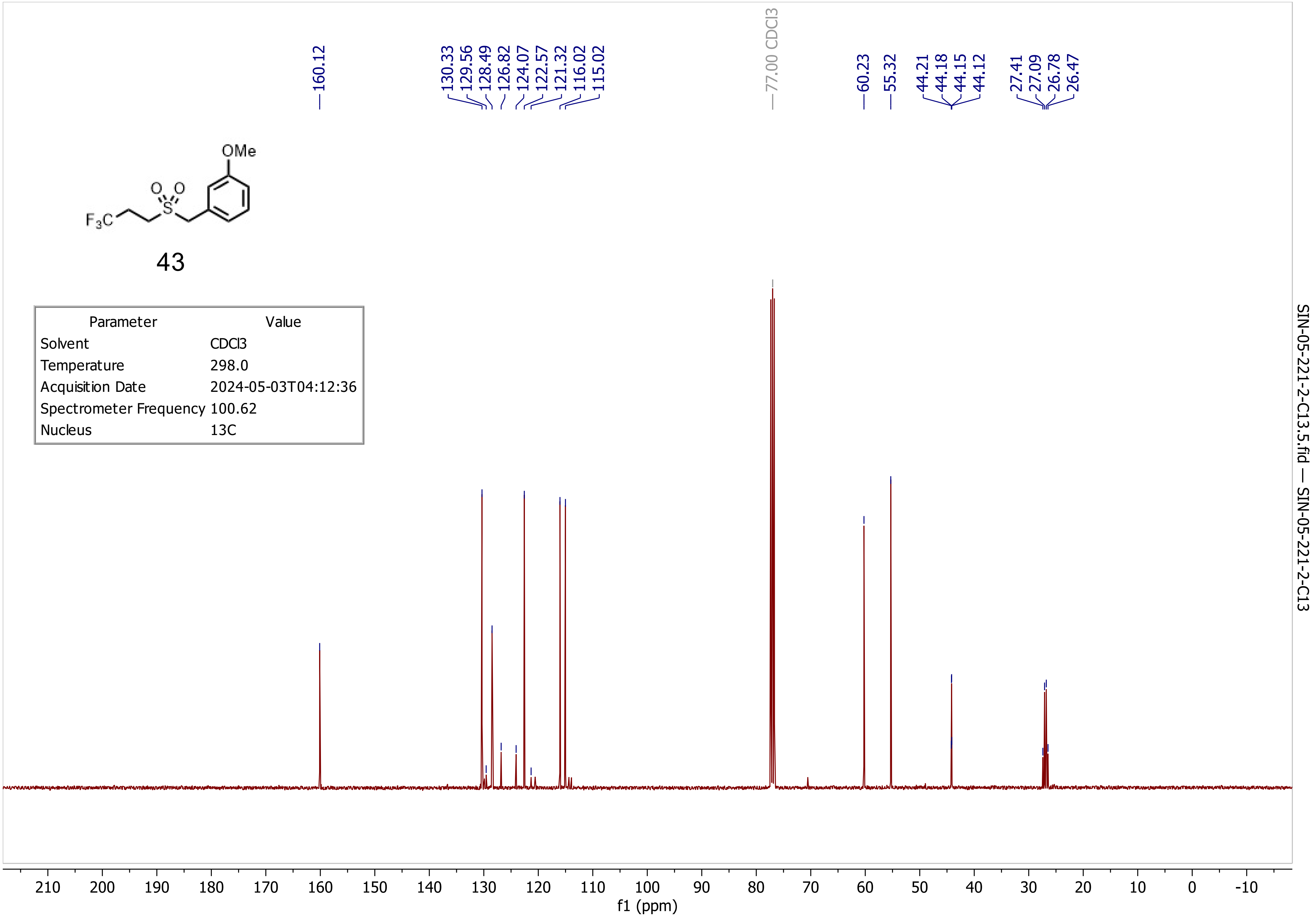

Parameter	Value
Solvent	CDCl3
Temperature	298.0
Acquisition Date	2024-05-03T04:12:36
Spectrometer Frequency	100.62
Nucleus	13C

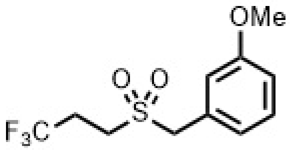

43

Parameter	Value
Solvent	CDCl3
Temperature	298.0
Acquisition Date	2024-05-03T03:12:16
Spectrometer Frequency	376.46
Nucleus	19F

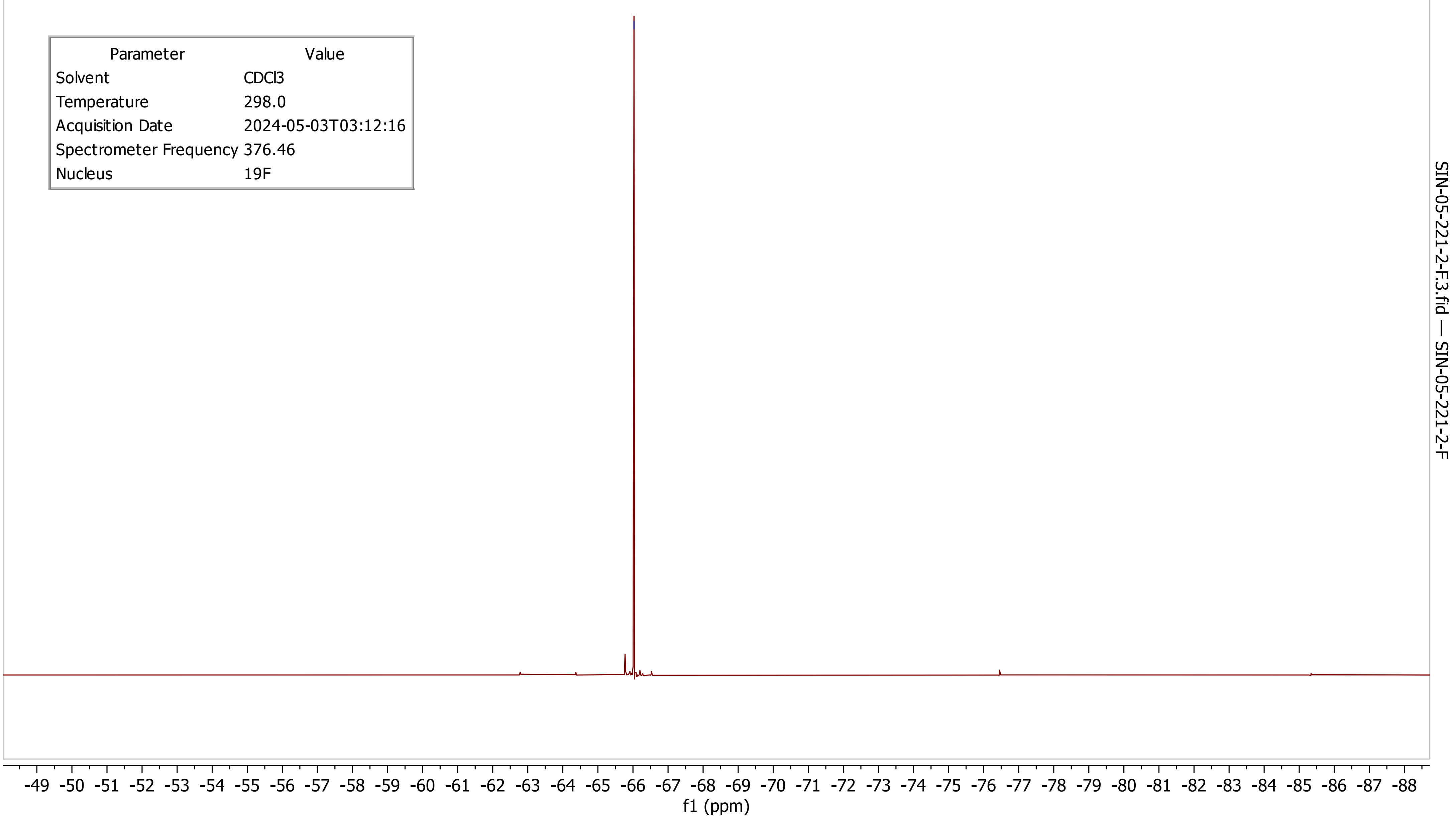

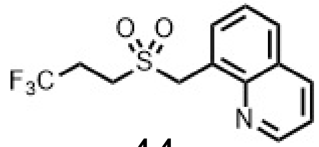

44

Parameter	Value
Solvent	CDCl3
Temperature	298.0
Acquisition Date	2024-05-02T18:52:40
Spectrometer Frequency	400.13
Nucleus	1H

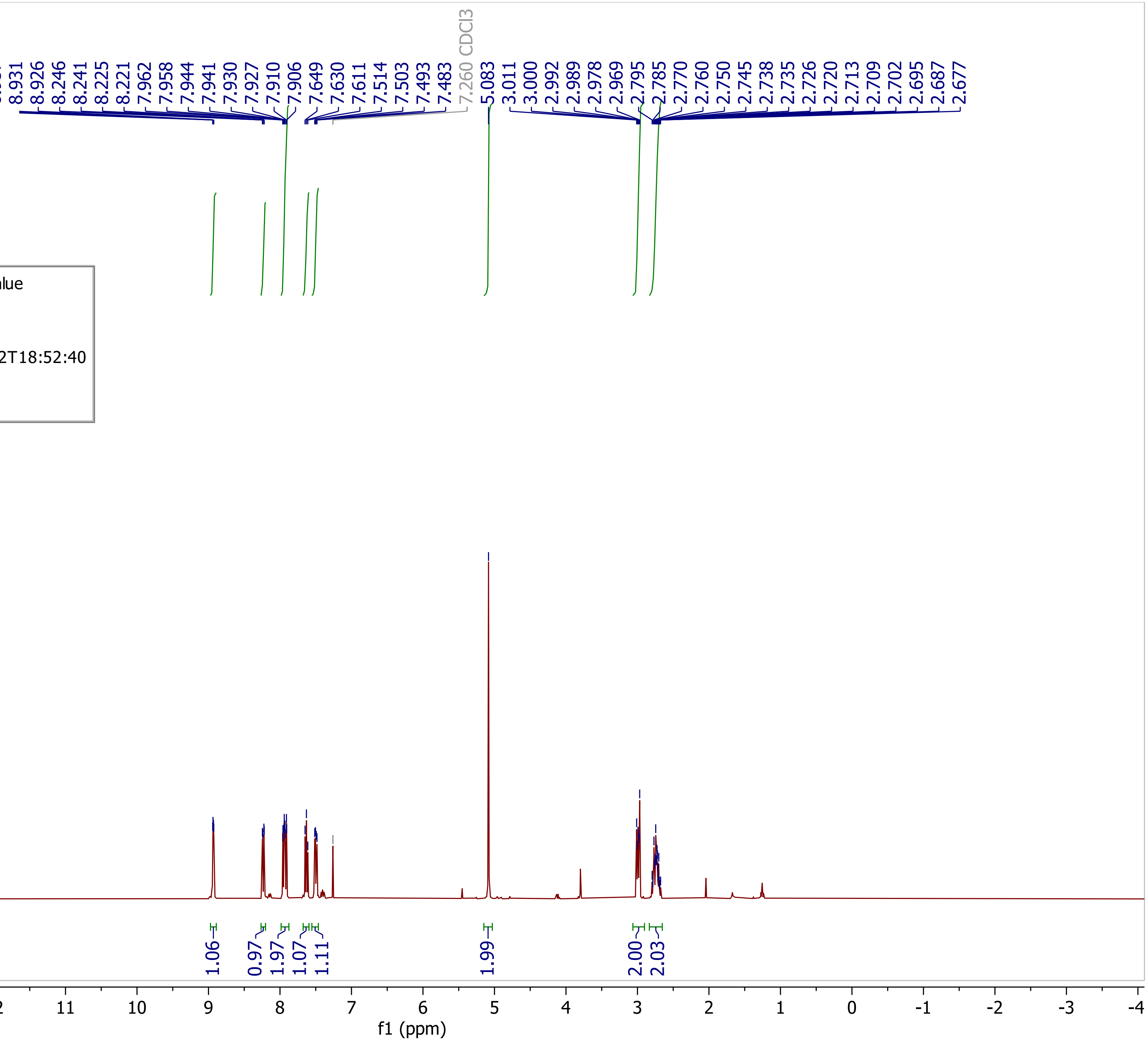

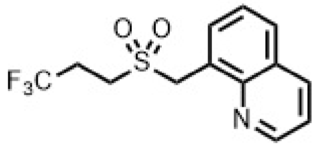

44

Parameter	Value
Solvent	CDCl3
Temperature	298.0
Acquisition Date	2024-05-03T00:12:37
Spectrometer Frequency	100.62
Nucleus	13C

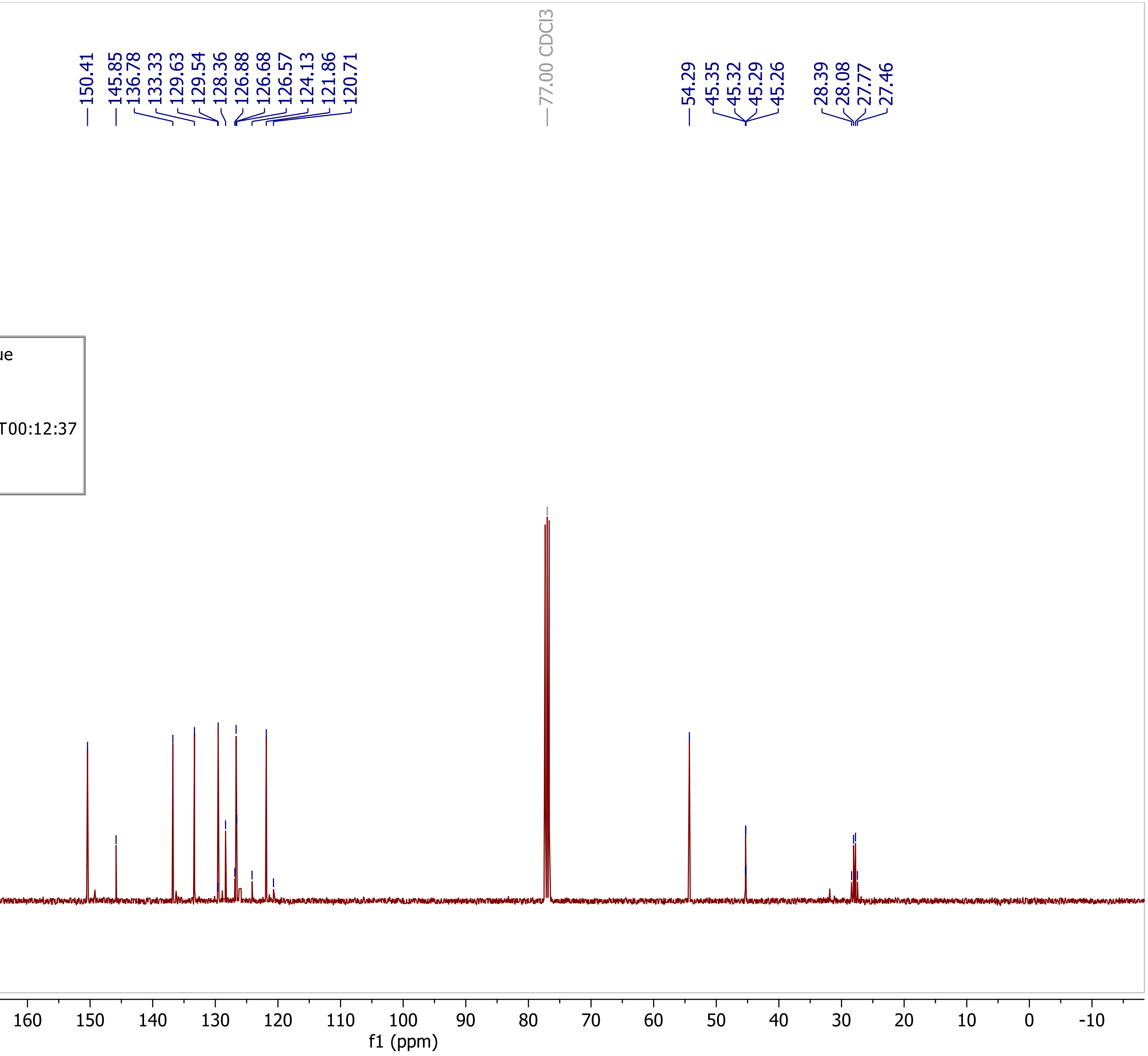

SIN-05-228-1-C13.5.fid — SIN-05-228-1-F

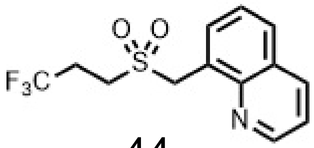

44

Parameter	Value
Solvent	CDCl3
Temperature	298.0
Acquisition Date	2024-05-02T23:53:48
Spectrometer Frequency	376.46
Nucleus	19F

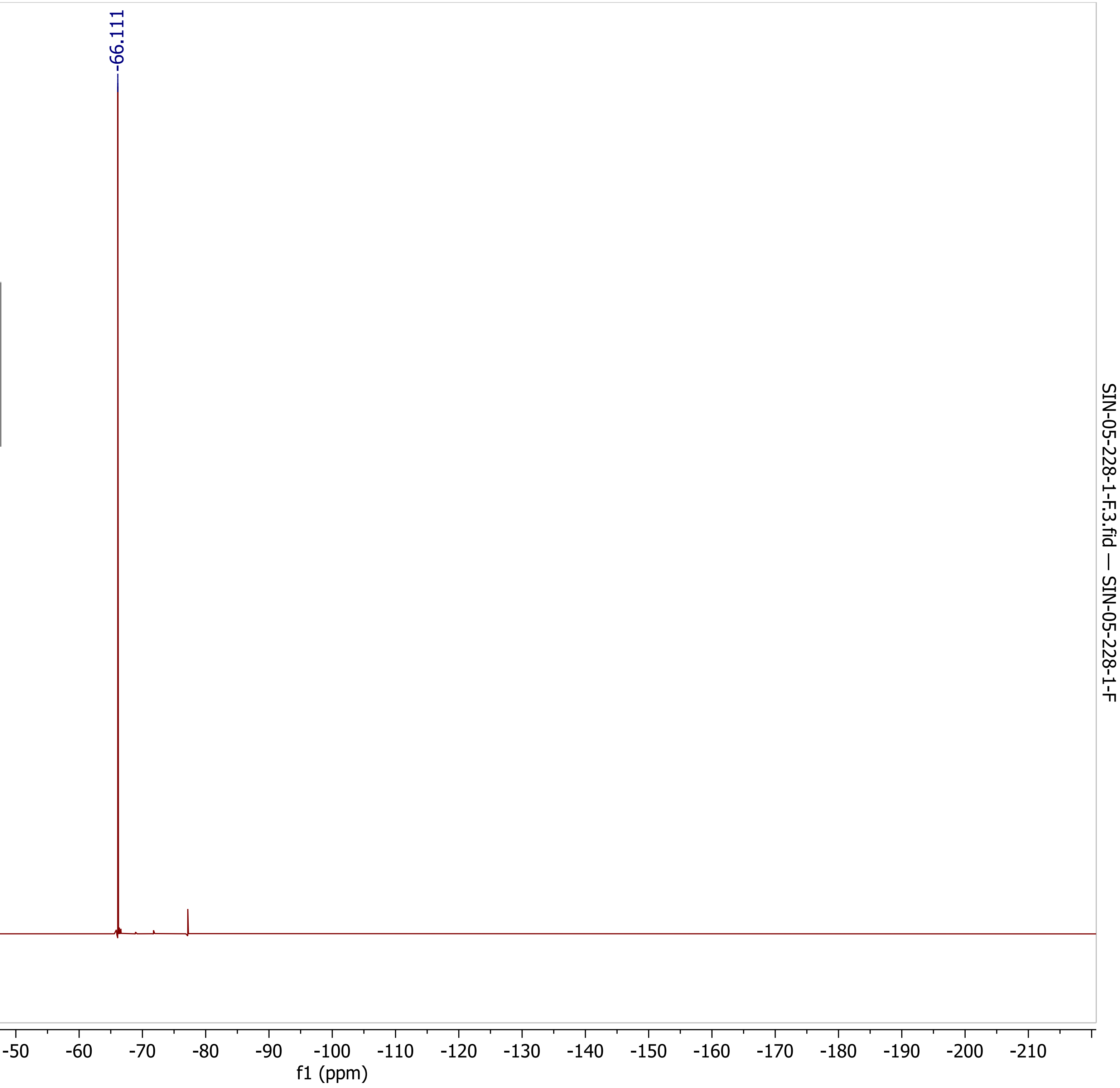

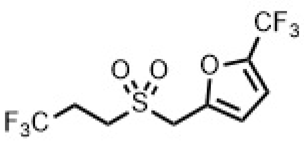

45

Parameter	Value
Solvent	CDCl3
Temperature	298.0
Acquisition Date	2024-05-02T18:58:37
Spectrometer Frequency	400.13
Nucleus	1H

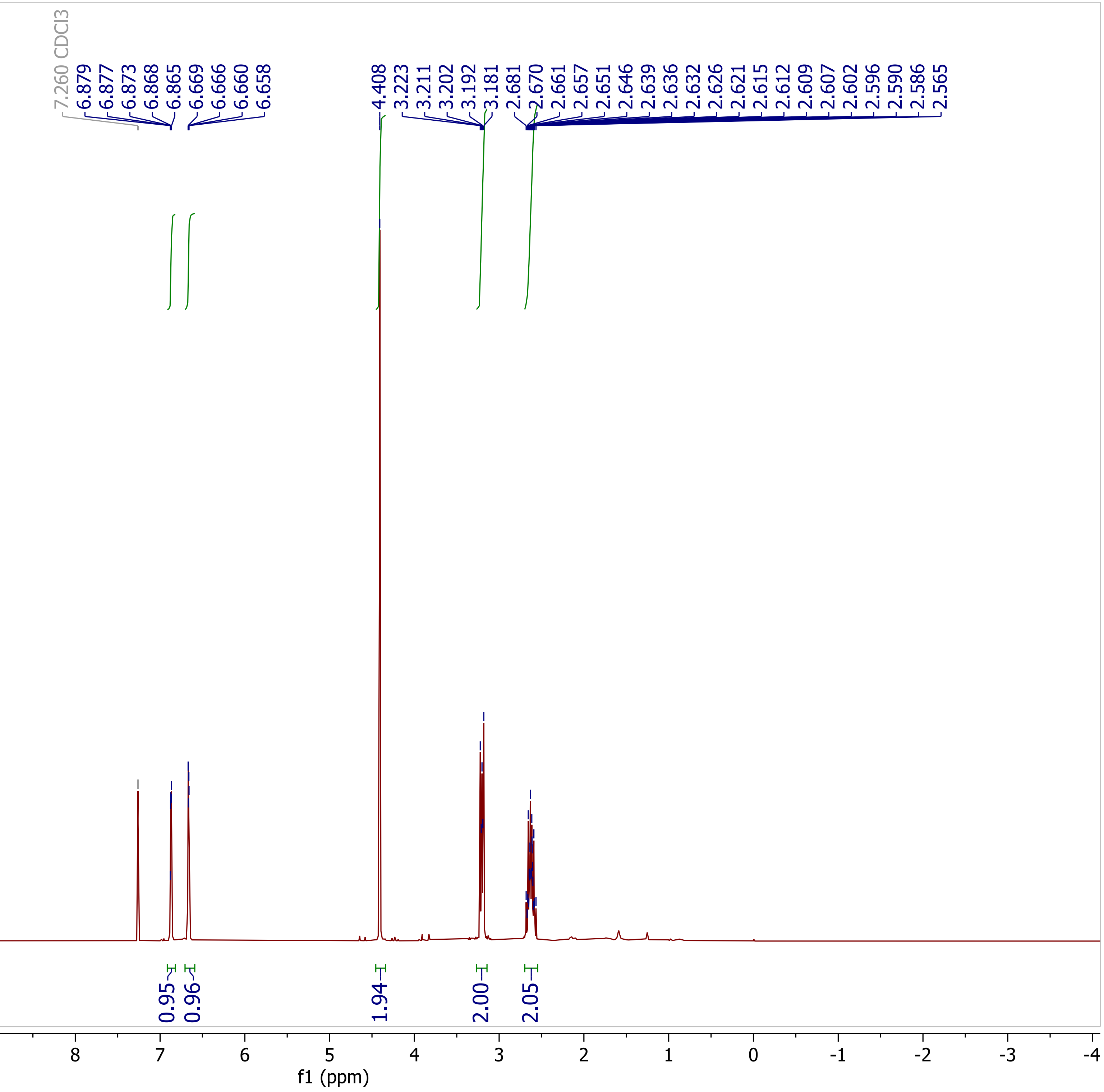

SIN-05-221-1-H.13.fid — SIN-05-221-1-H

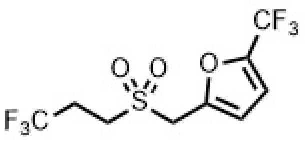

45

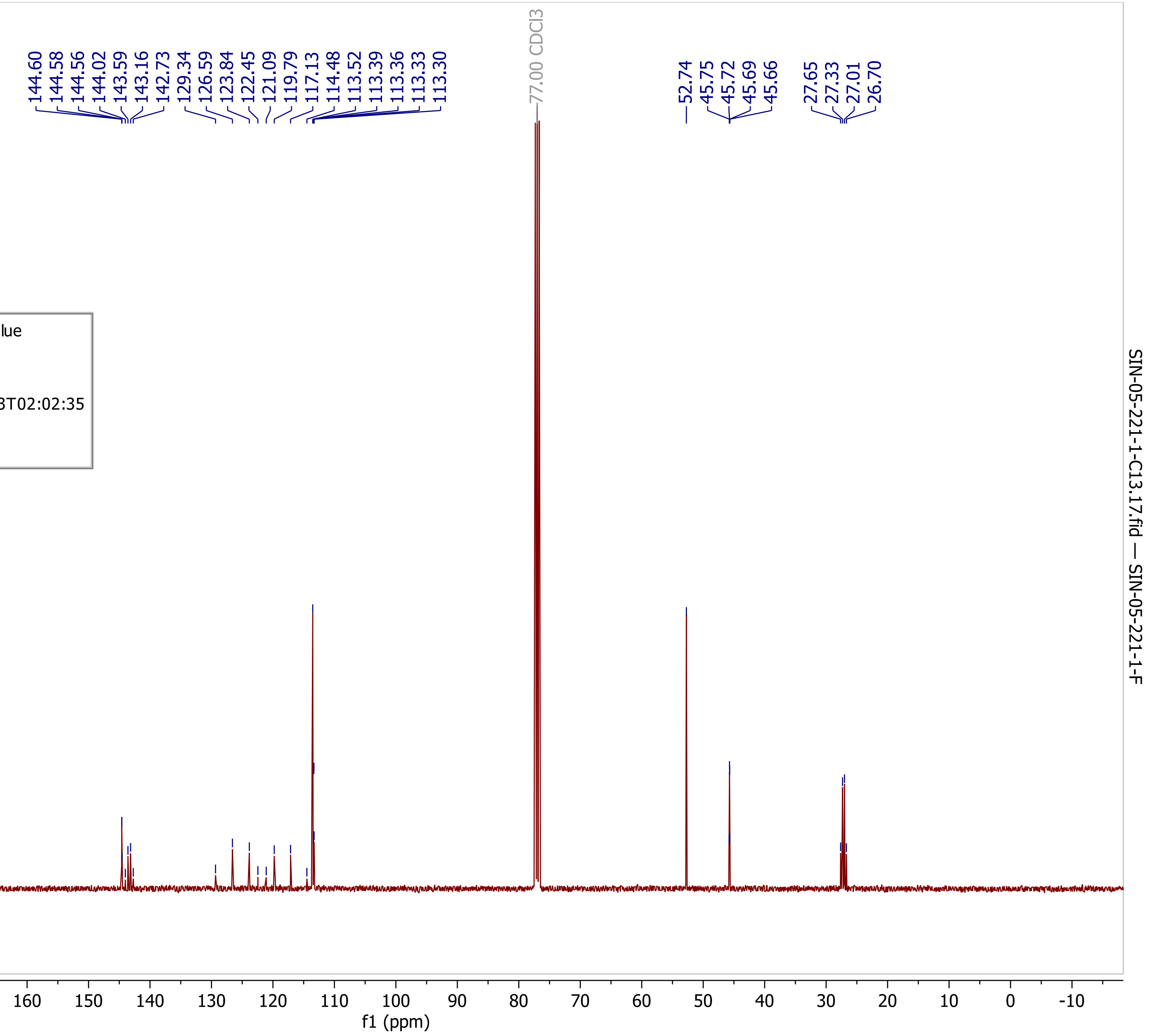

Parameter	Value
Solvent	CDCl3
Temperature	298.0
Acquisition Date	2024-05-03T02:02:35
Spectrometer Frequency	100.62
Nucleus	13C

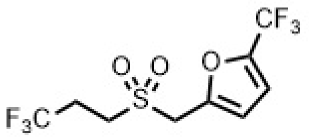

45

Parameter	Value
Solvent	CDCl3
Temperature	298.0
Acquisition Date	2024-05-03T01:02:17
Spectrometer Frequency	376.46
Nucleus	19F

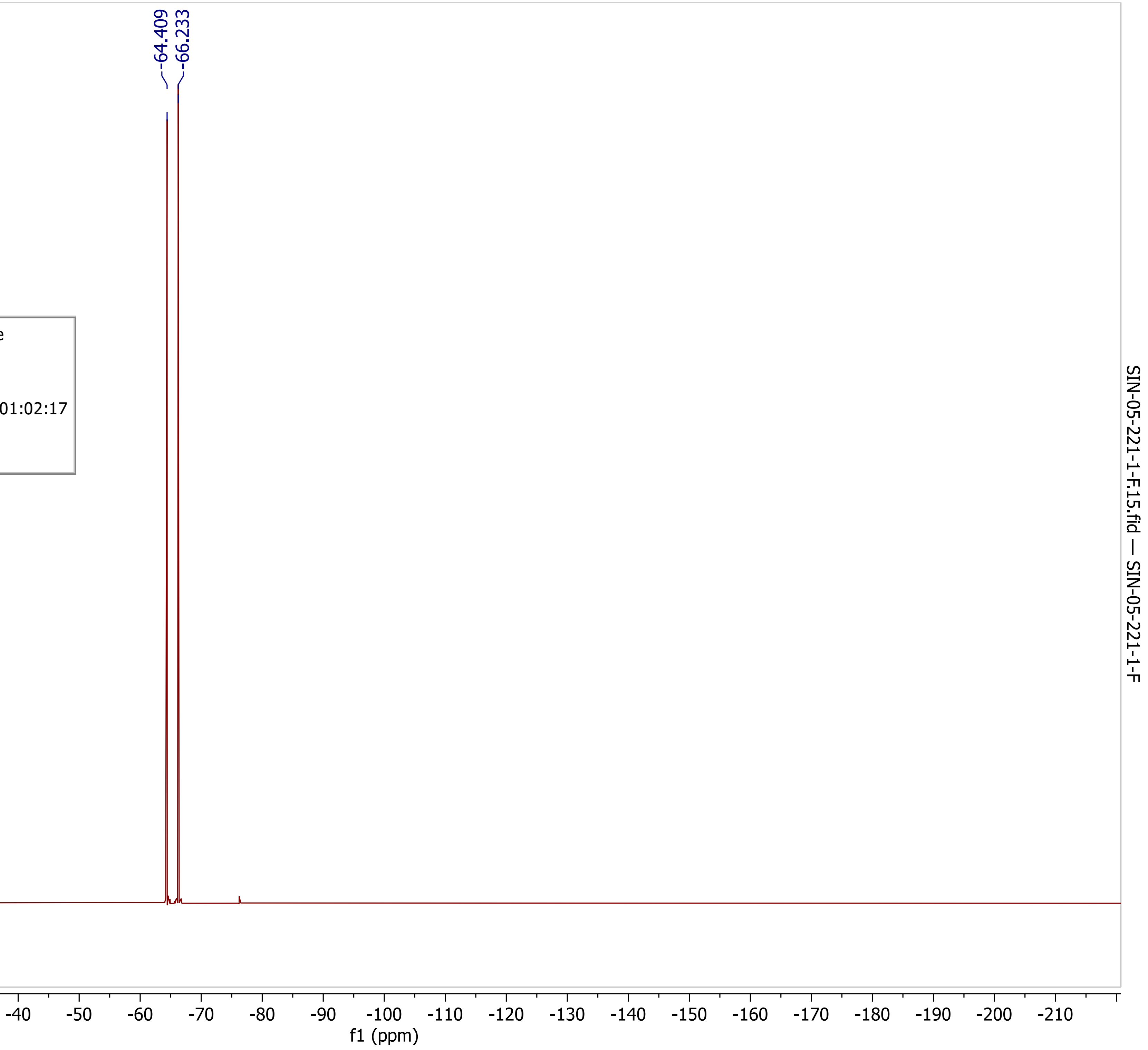

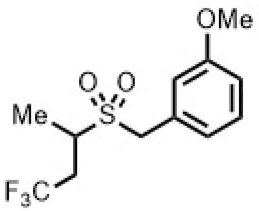

46

Parameter	Value
Solvent	CDCl3
Temperature	298.0
Acquisition Date	2024-05-23T19:34:47
Spectrometer Frequency	400.13
Nucleus	1H

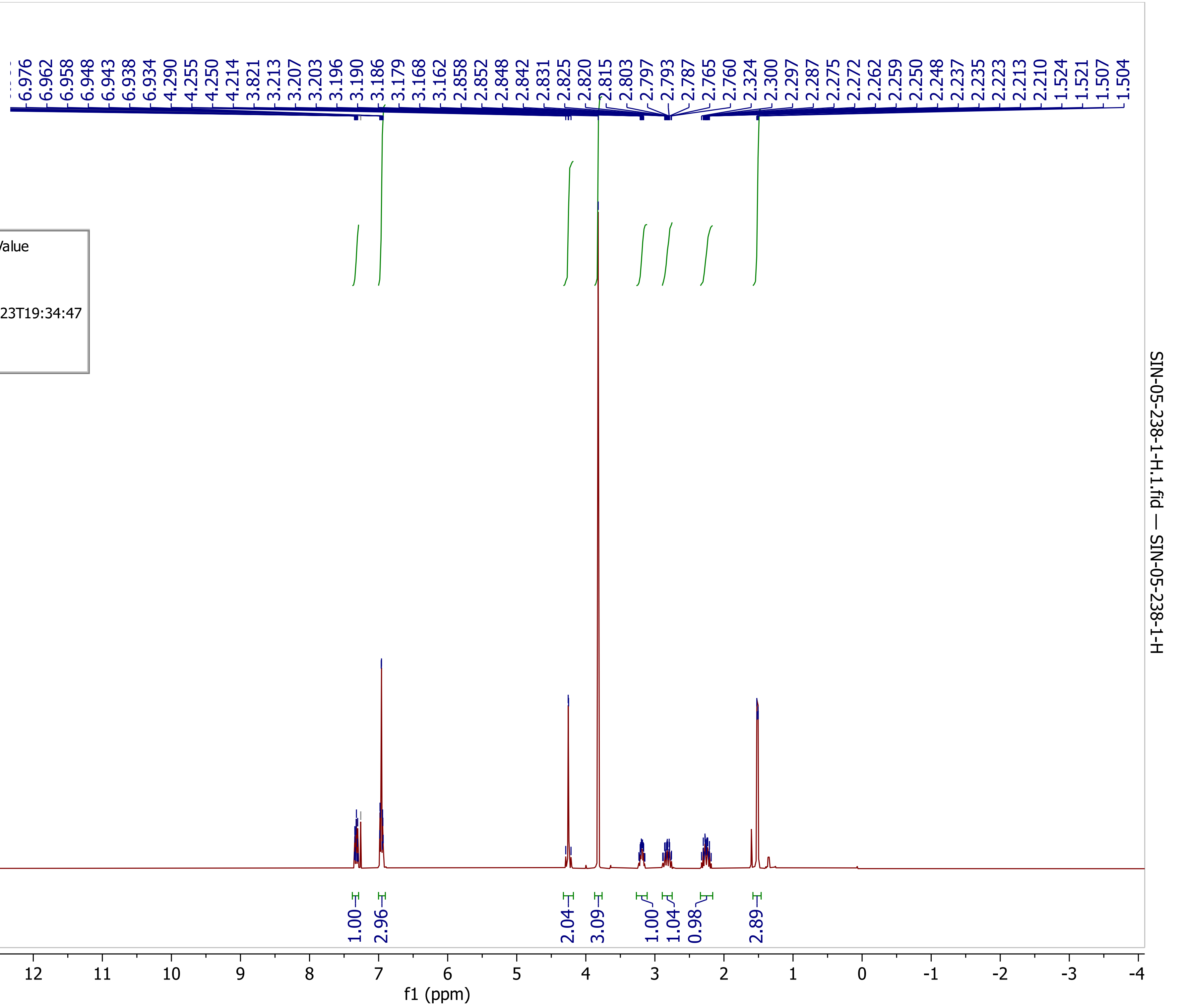

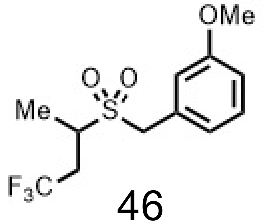

46

Parameter	Value
Solvent	CDCl3
Temperature	298.0
Acquisition Date	2024-05-24T01:27:23
Spectrometer Frequency	100.62
Nucleus	13C

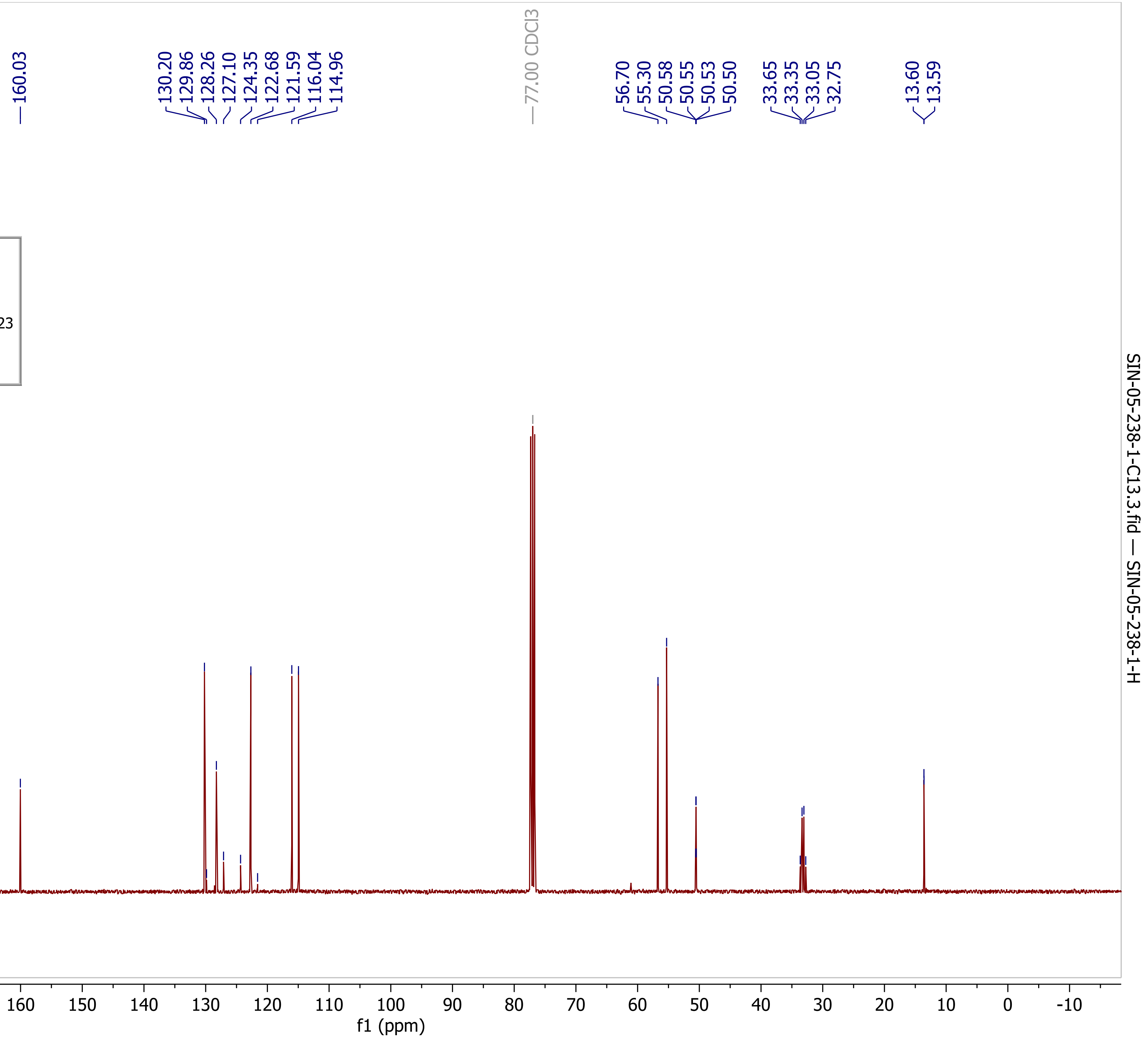

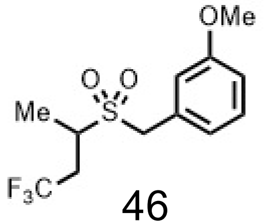

46

Parameter	Value
Solvent	CDCl3
Temperature	298.0
Acquisition Date	2024-05-18T16:10:40
Spectrometer Frequency	376.46
Nucleus	19F

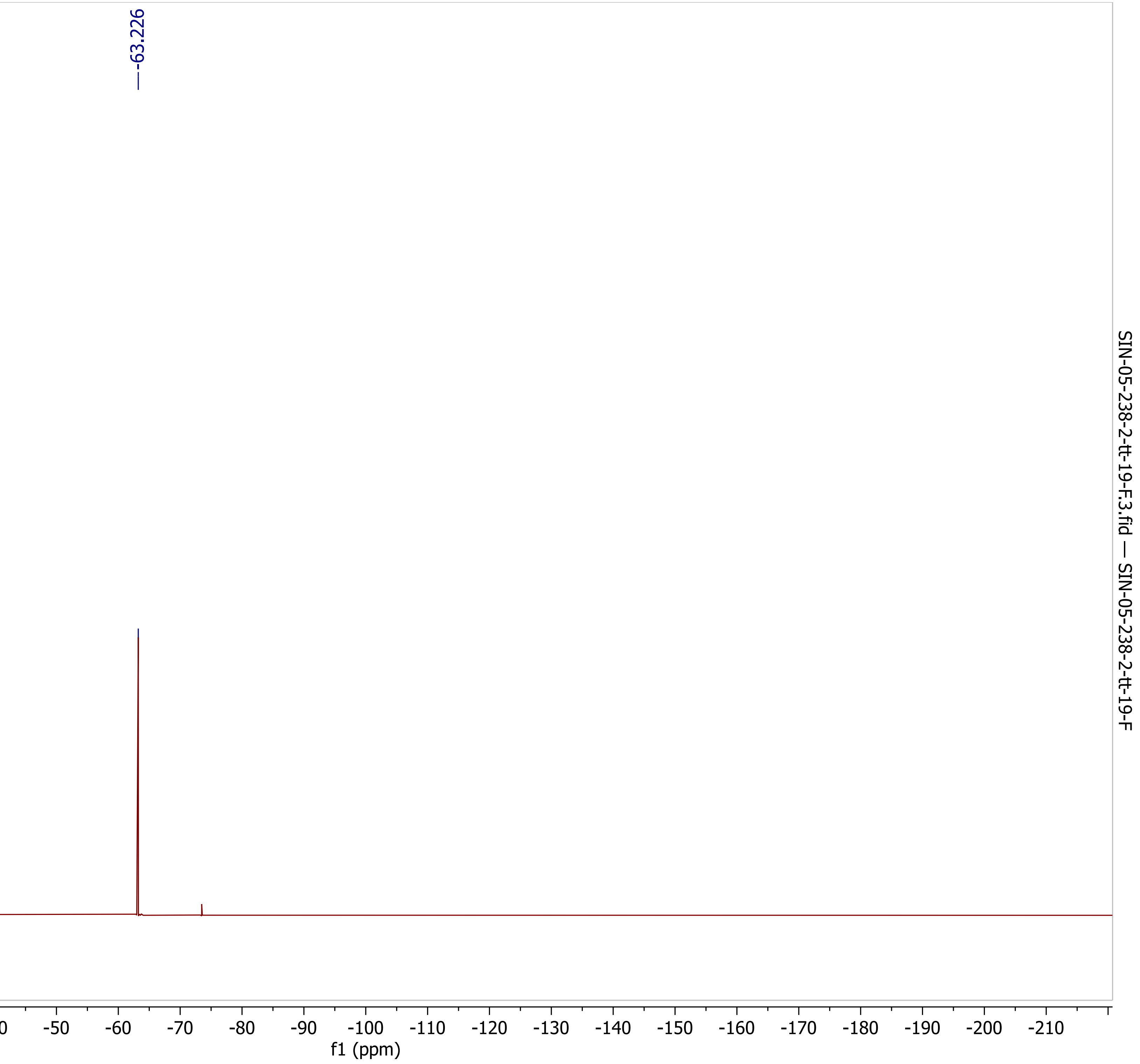

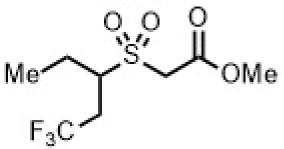

47

Parameter	Value
Solvent	CDCl3
Temperature	298.0
Acquisition Date	2024-05-11T16:32:49
Spectrometer Frequency	400.13
Nucleus	1H

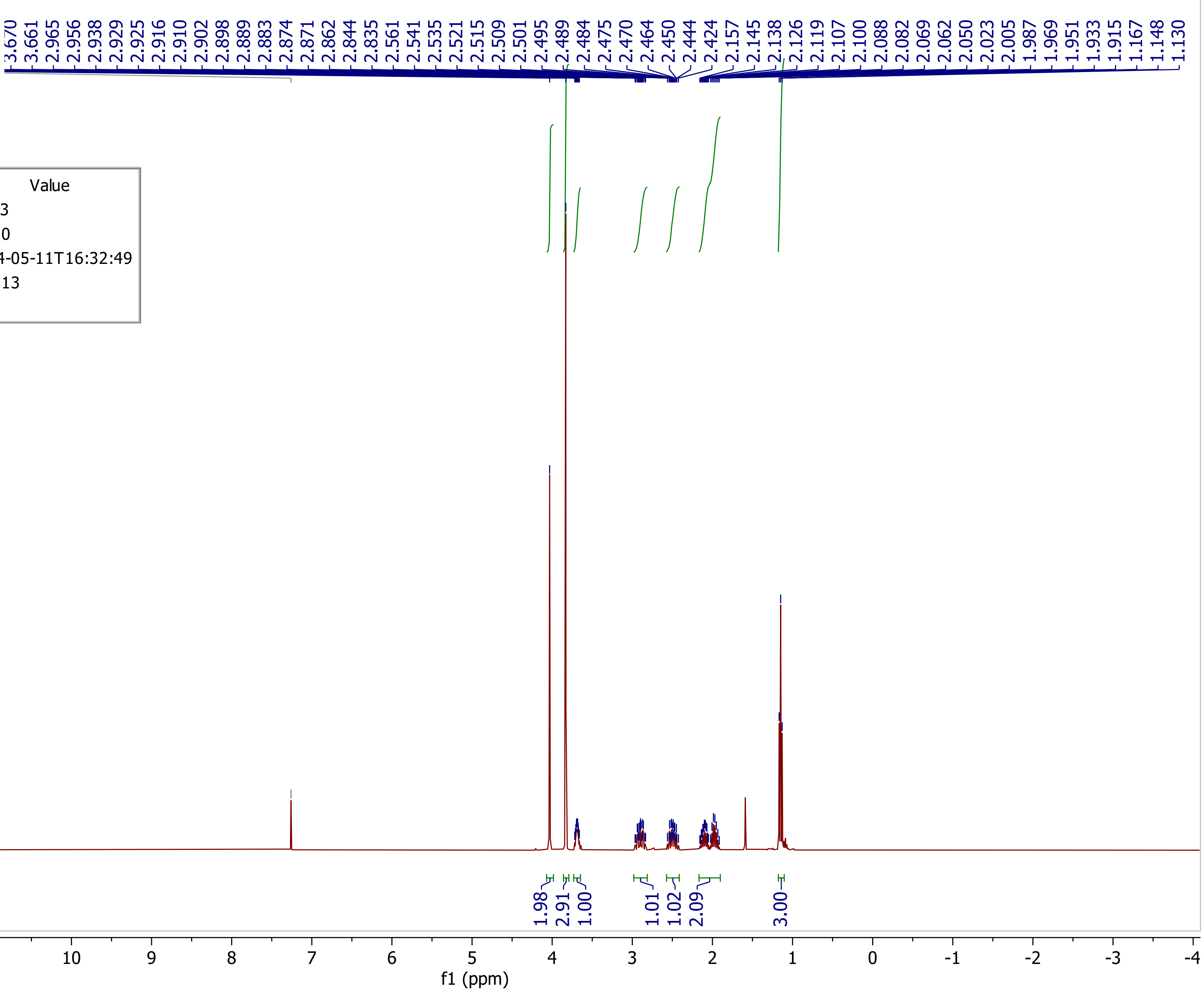

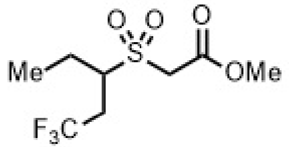

47

Parameter	Value
Solvent	CDCl3
Temperature	298.0
Acquisition Date	2024-05-11T21:32:53
Spectrometer Frequency	100.62
Nucleus	13C

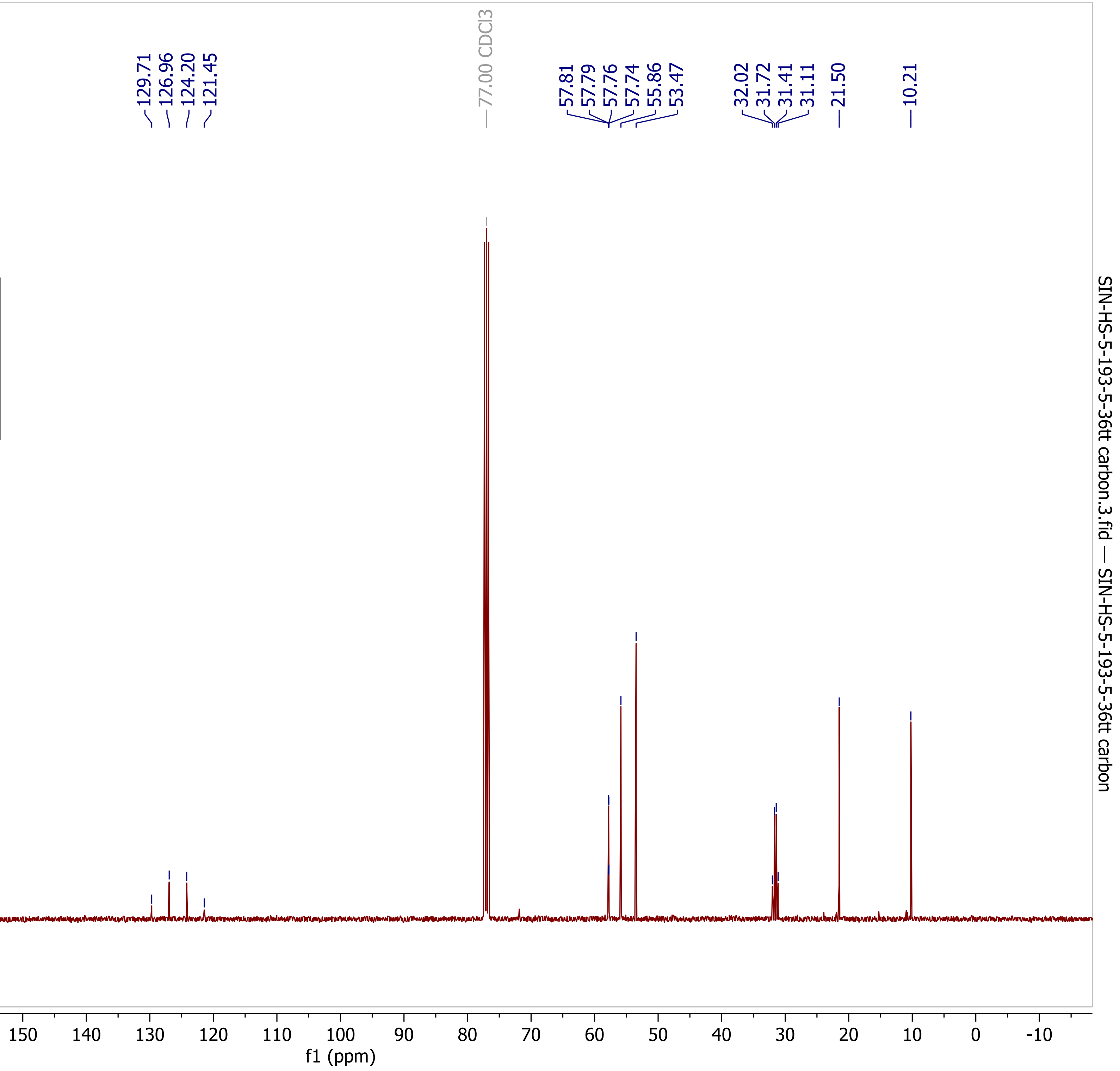

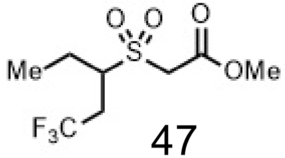

47

Parameter	Value
Solvent	CDCl3
Temperature	298.0
Acquisition Date	2024-08-06T19:23:49
Spectrometer Frequency	376.46
Nucleus	19F

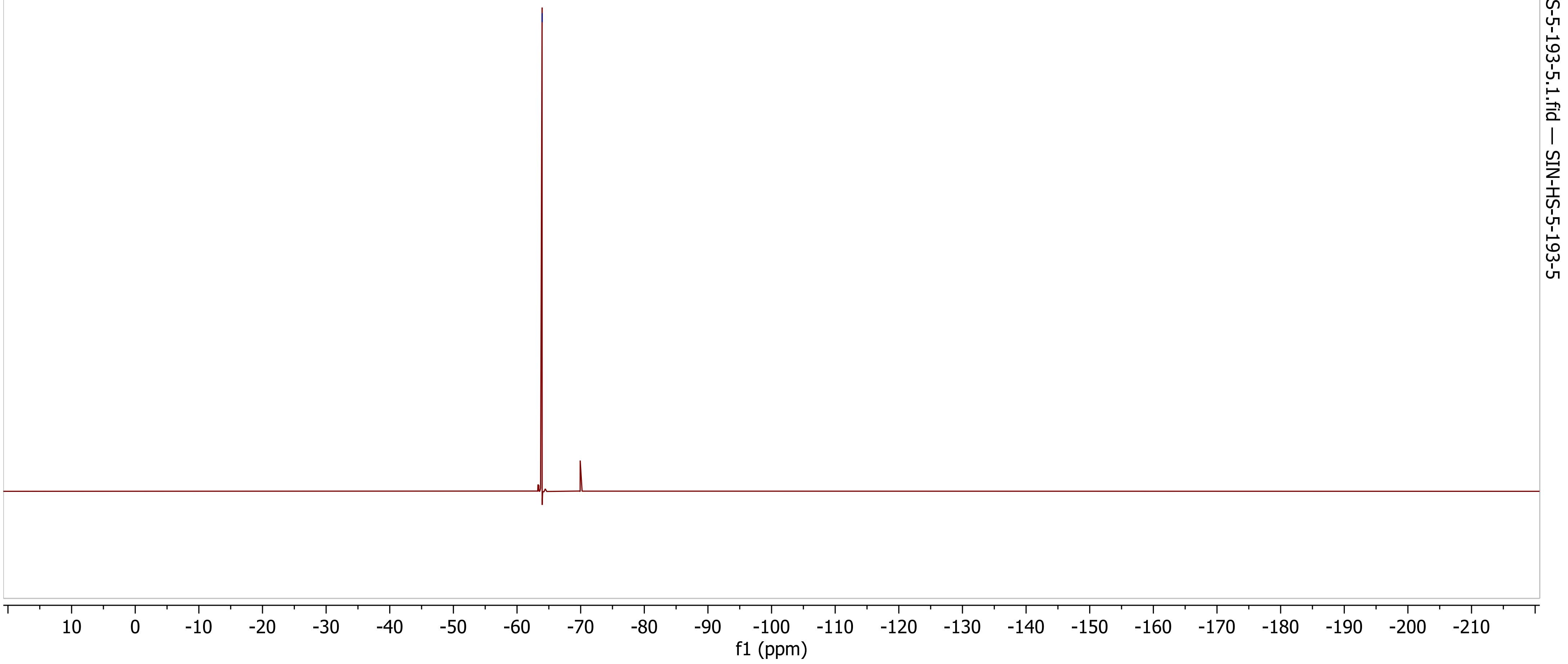

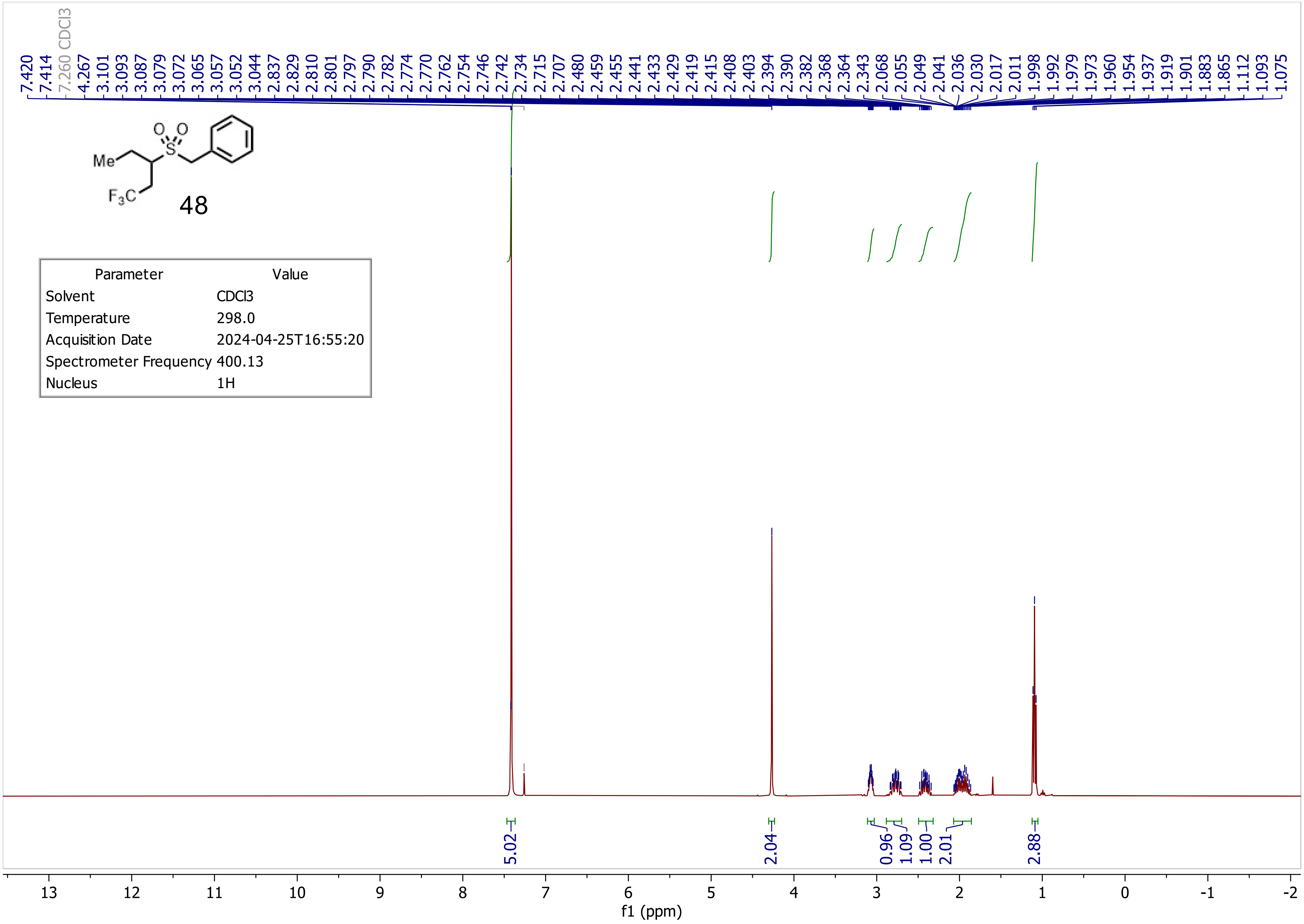

SIN-HS-5-195-1-12tt.1.fid — SIN-HS-5-195-1-12tt

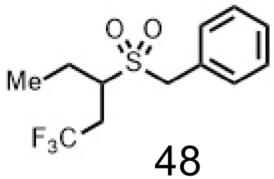

48

Parameter	Value
Solvent	CDCl3
Temperature	298.0
Acquisition Date	2024-05-10T03:35:04
Spectrometer Frequency	100.62
Nucleus	13C

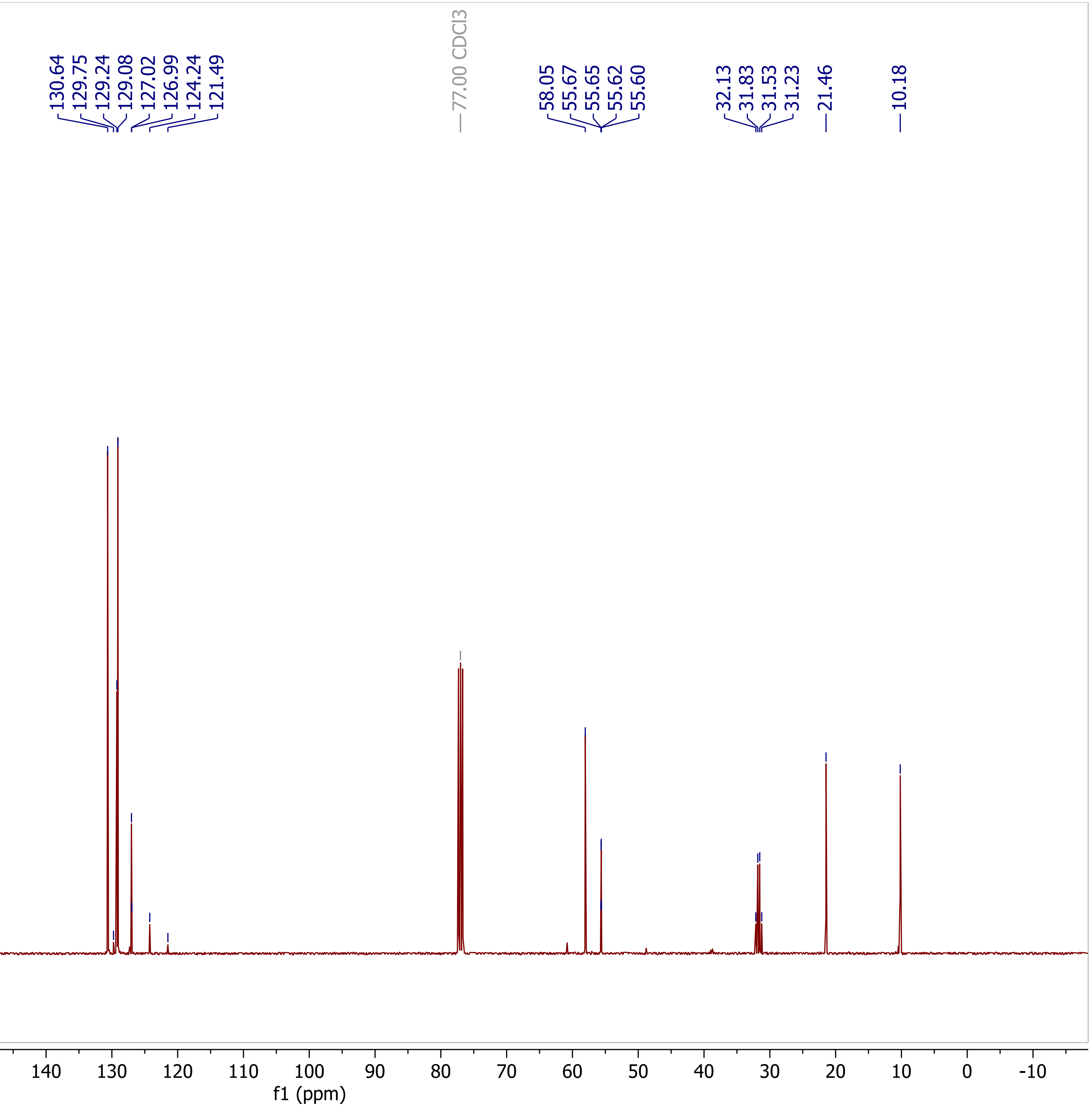

sin-HS-5-195-1-13C.1.fid — HS-5-195-13C

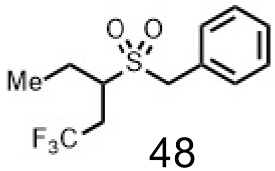

48

Parameter	Value
Solvent	CDCl3
Temperature	298.0
Acquisition Date	2024-08-06T19:09:31
Spectrometer Frequency	376.46
Nucleus	19F

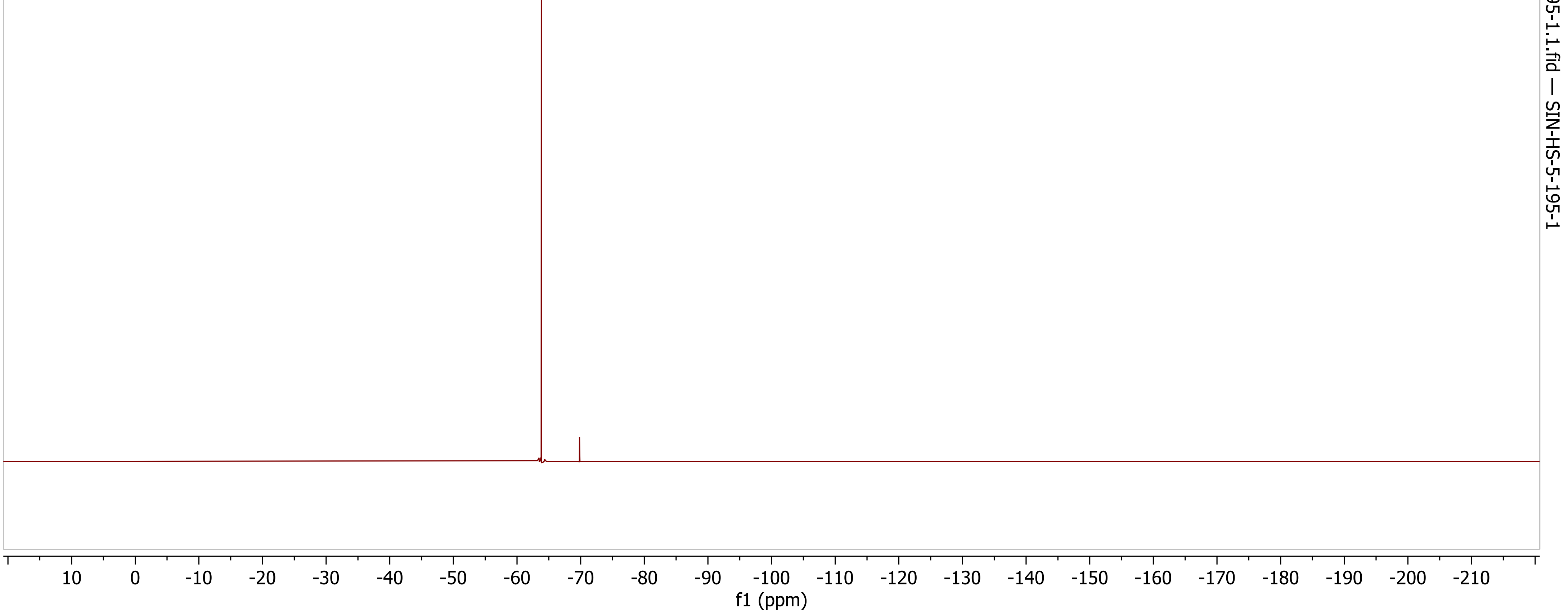

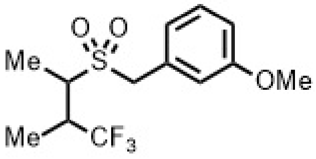

49

Parameter	Value
Solvent	CDCl3
Temperature	298.0
Acquisition Date	2024-06-21T23:36:35
Spectrometer Frequency	400.13
Nucleus	1H

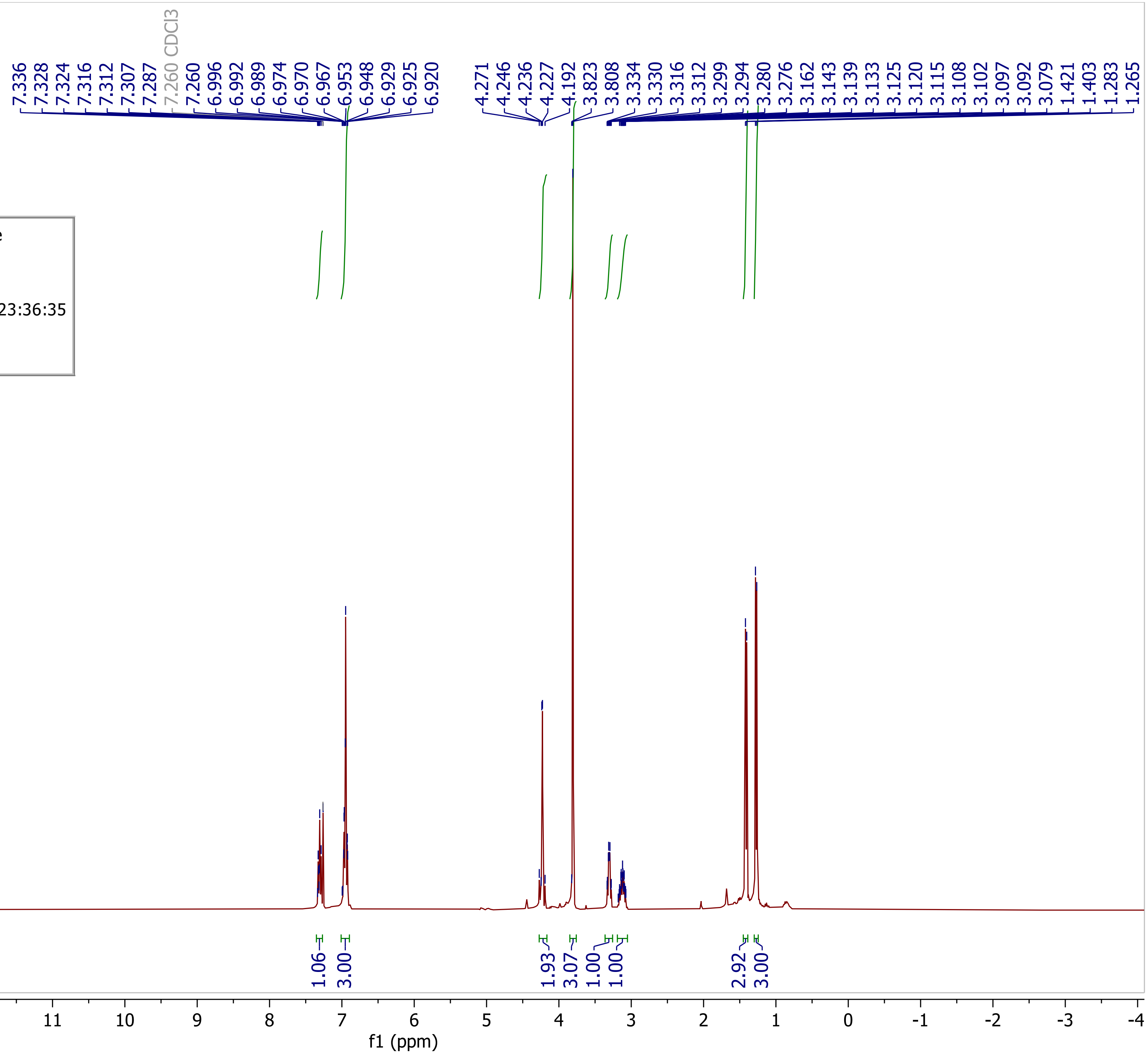

AA-03-15-1H.1.fid — AA-03-15-1H

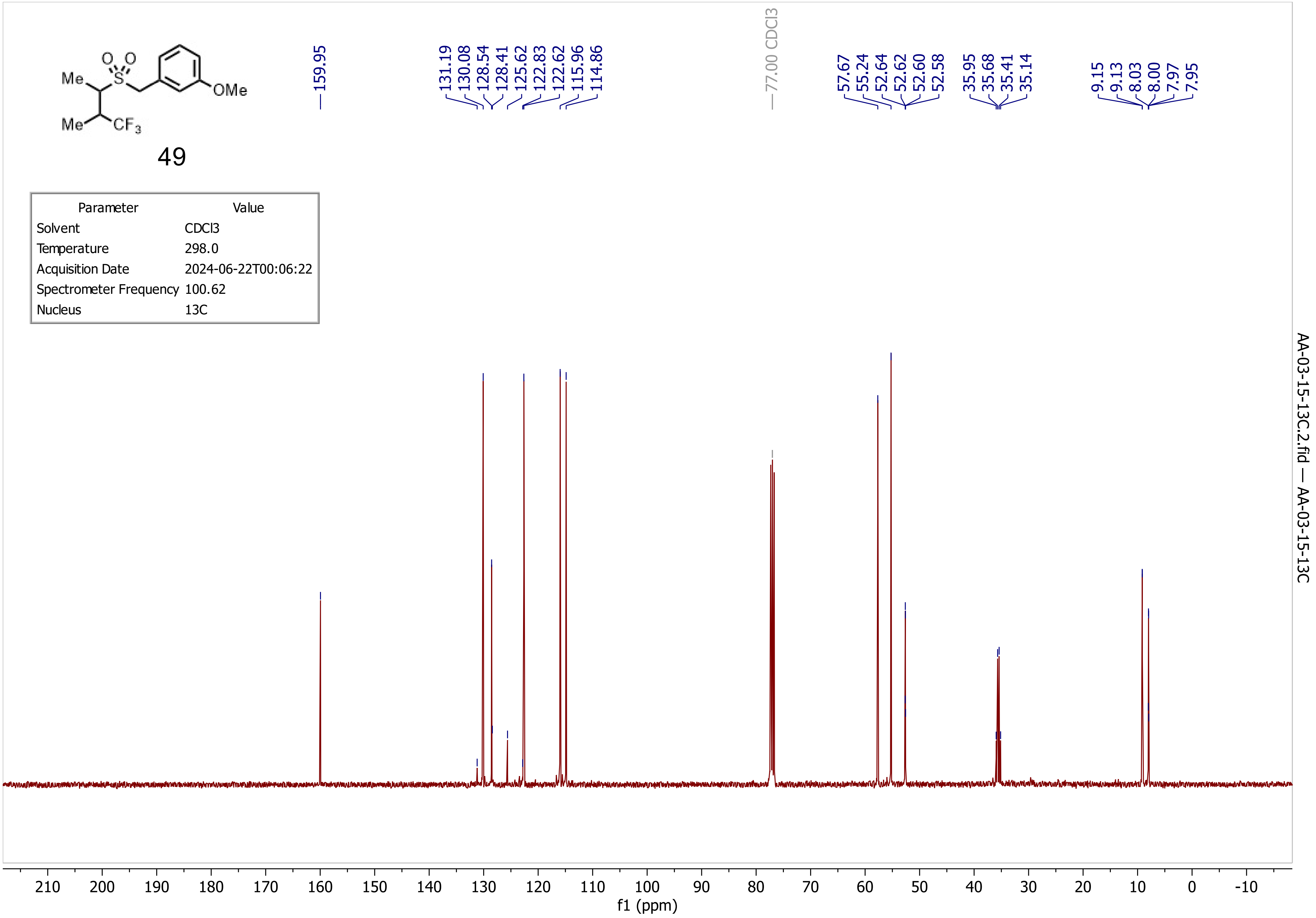

Parameter	Value
Solvent	CDCl3
Temperature	298.0
Acquisition Date	2024-06-22T00:06:22
Spectrometer Frequency	100.62
Nucleus	13C

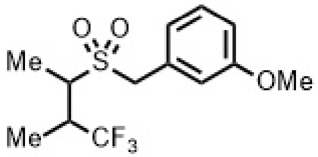

49

Parameter	Value
Solvent	CDCl3
Temperature	298.0
Acquisition Date	2024-06-22T00:10:39
Spectrometer Frequency	376.46
Nucleus	19F

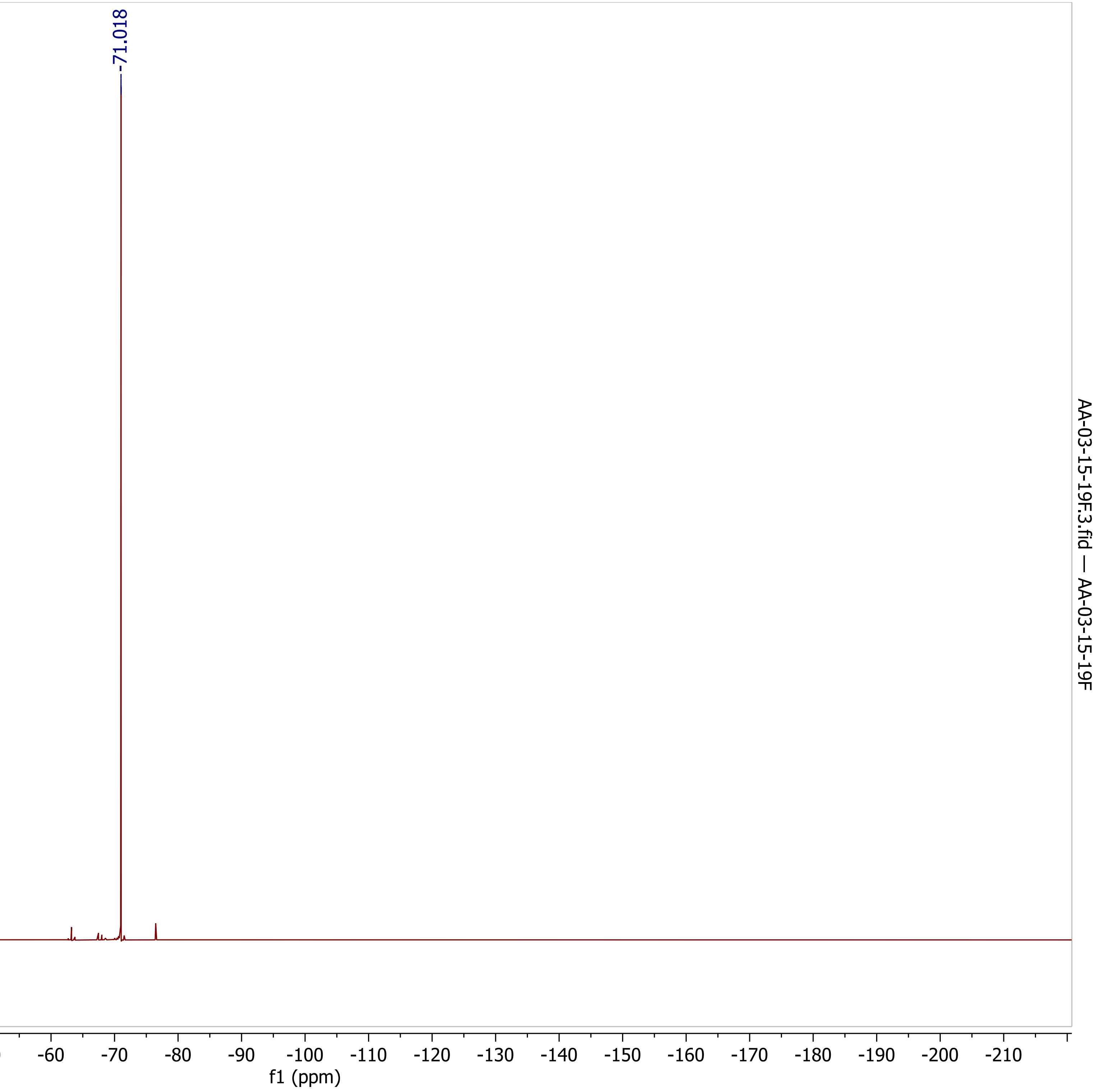

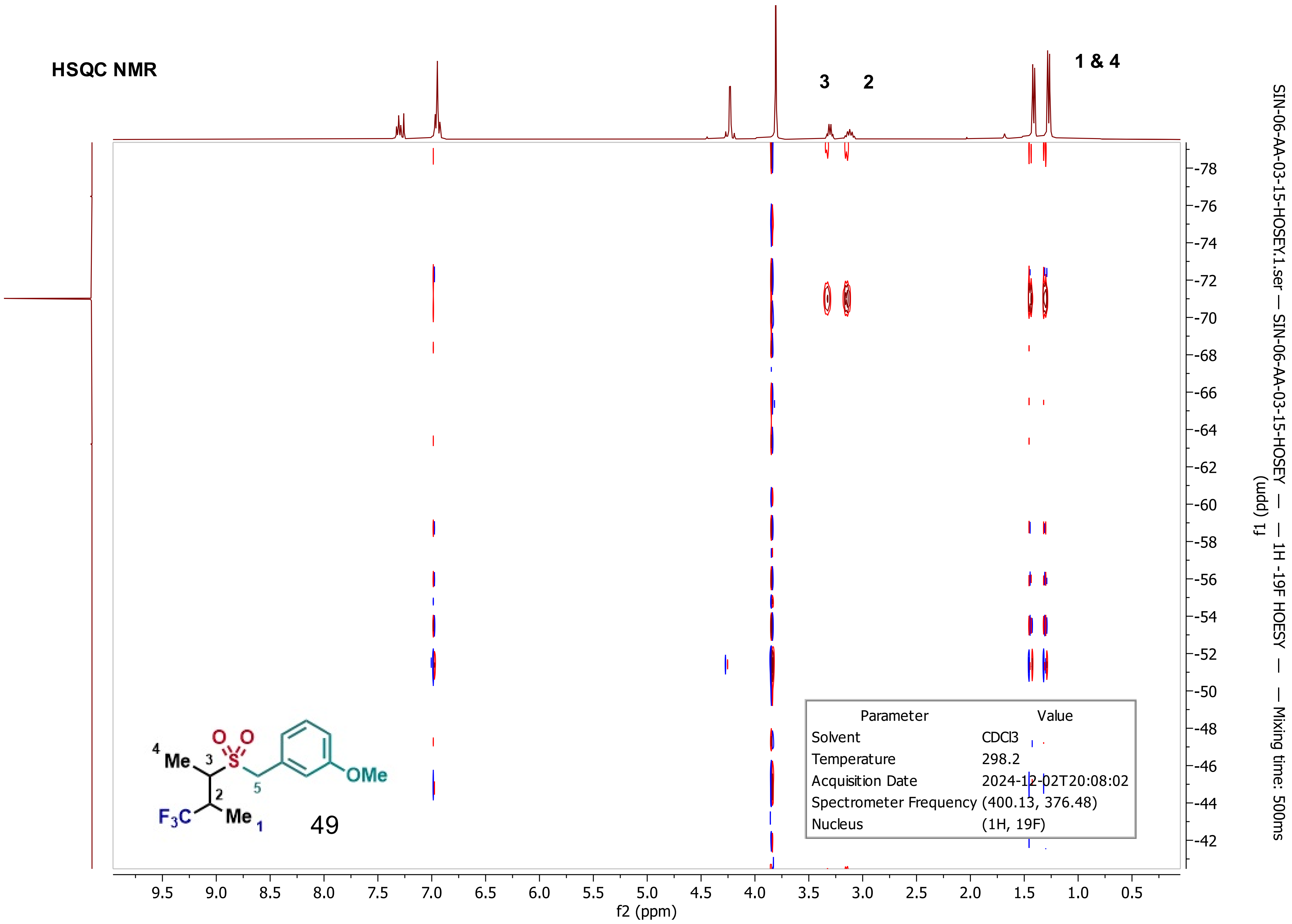
HSQC NMR
3
2
1 & 4
Me
O
O
S
3
4
2
5
F3C
Me
1
OMe
49
Parameter Value
Solvent CDCl3
Temperature 298.2
Acquisition Date 2024-12-02T20:08:02
Spectrometer Frequency (400.13, 376.48)
Nucleus (1H, 19F)
f2 (ppm)
f1 (ppm)
SIN-06-AA-03-15-HOSEY.1.ser — SIN-06-AA-03-15-HOSEY — — 1H -19F HOESY — — Mixing time: 500ms

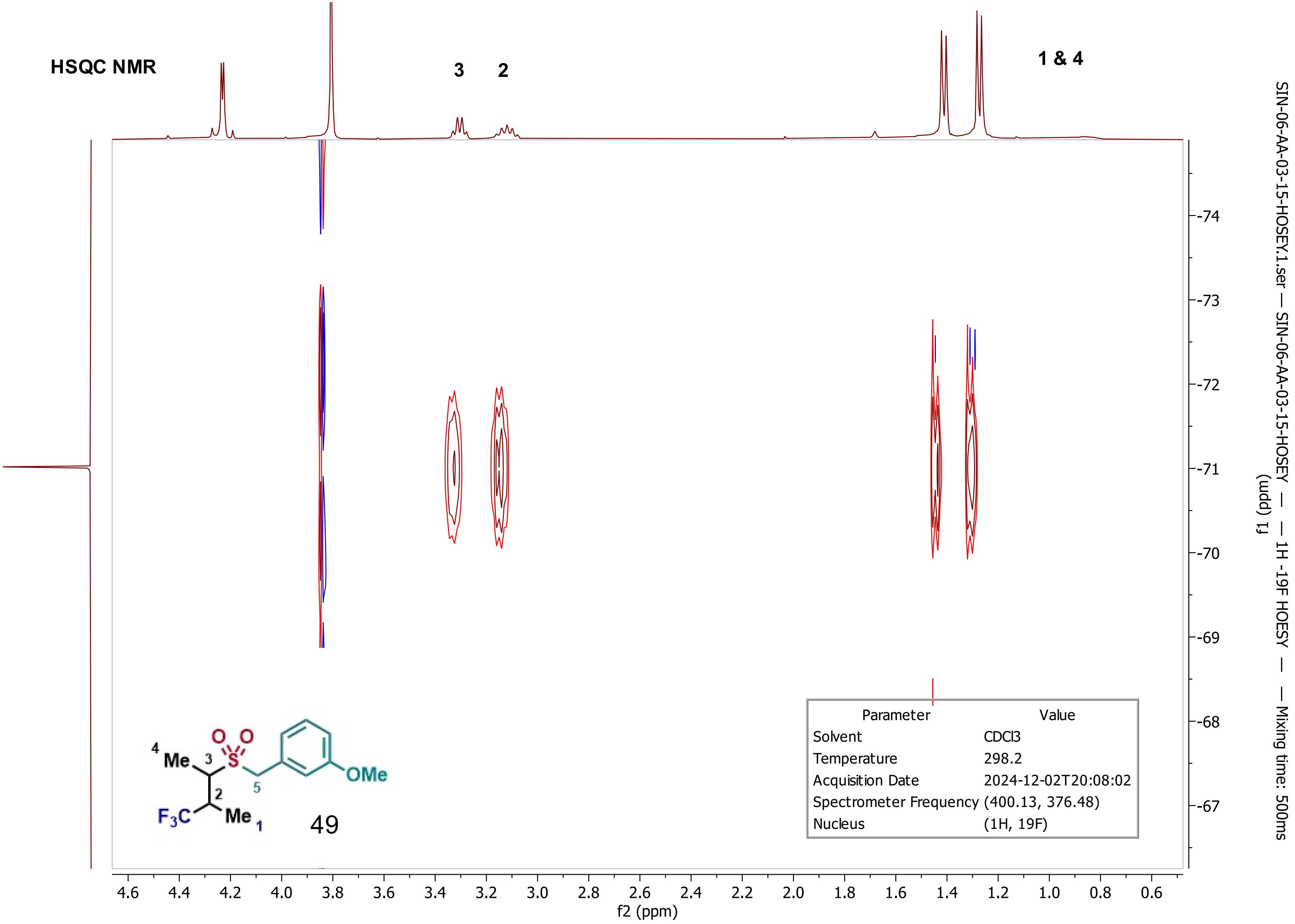

HSQC NMR
3
2
1 & 4
Me
F3C
Me
OMe
49
Parameter
Value
Solvent
CDCl3
Temperature
298.2
Acquisition Date
2024-12-02T20:08:02
Spectrometer Frequency
(400.13, 376.48)
Nucleus
(1H, 19F)
f2 (ppm)
f1 (ppm)
SIN-06-AA-03-15-HOSEY.1.ser — SIN-06-AA-03-15-HOSEY — — 1H -19F HOESY — — Mixing time: 500ms

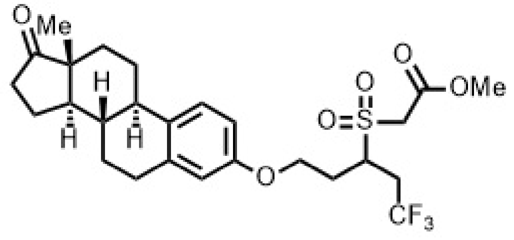

50

Parameter	Value
Solvent	CDCl3
Temperature	298.0
Acquisition Date	2024-04-15T16:36:36
Spectrometer Frequency	400.13
Nucleus	1H

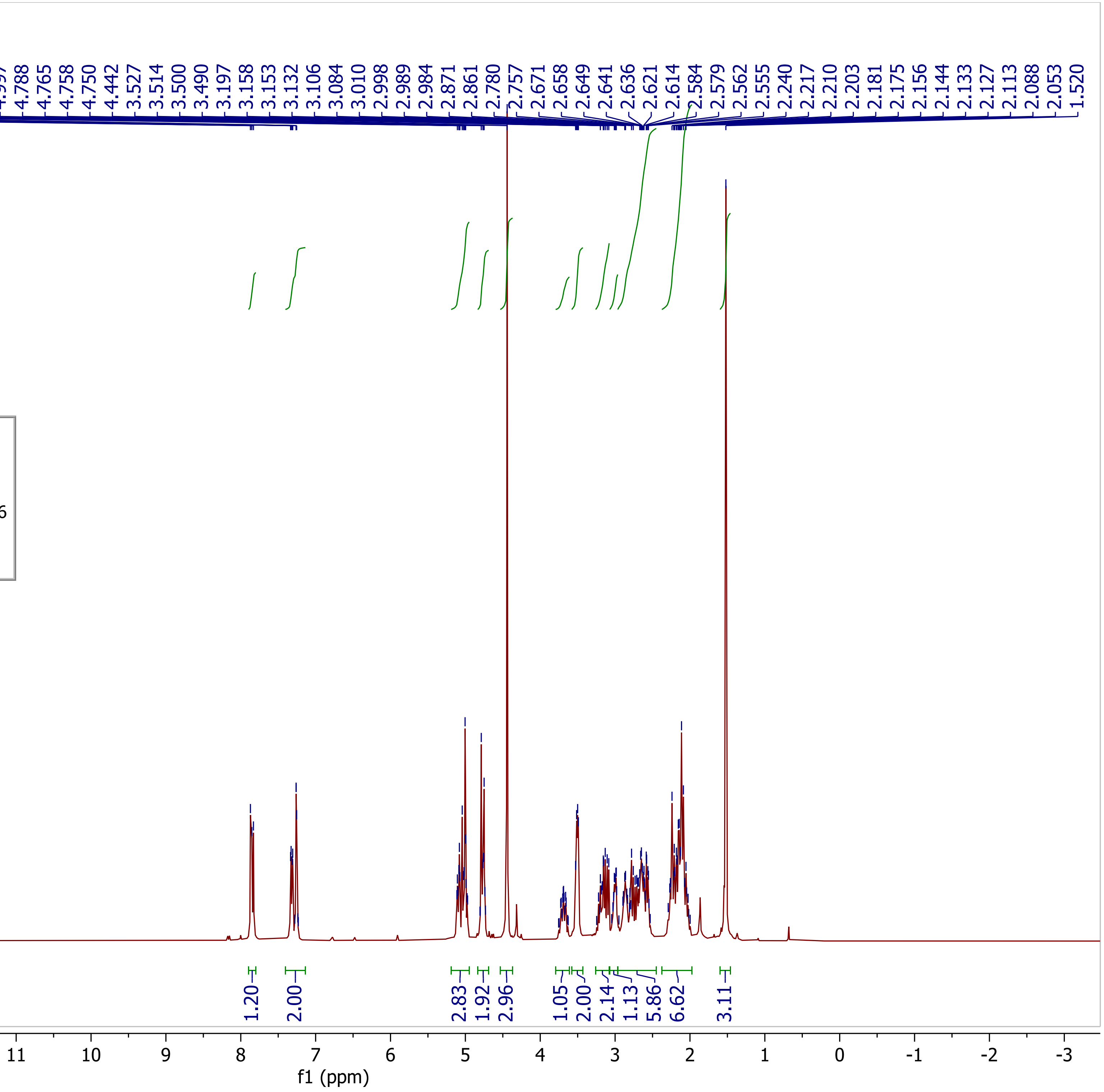

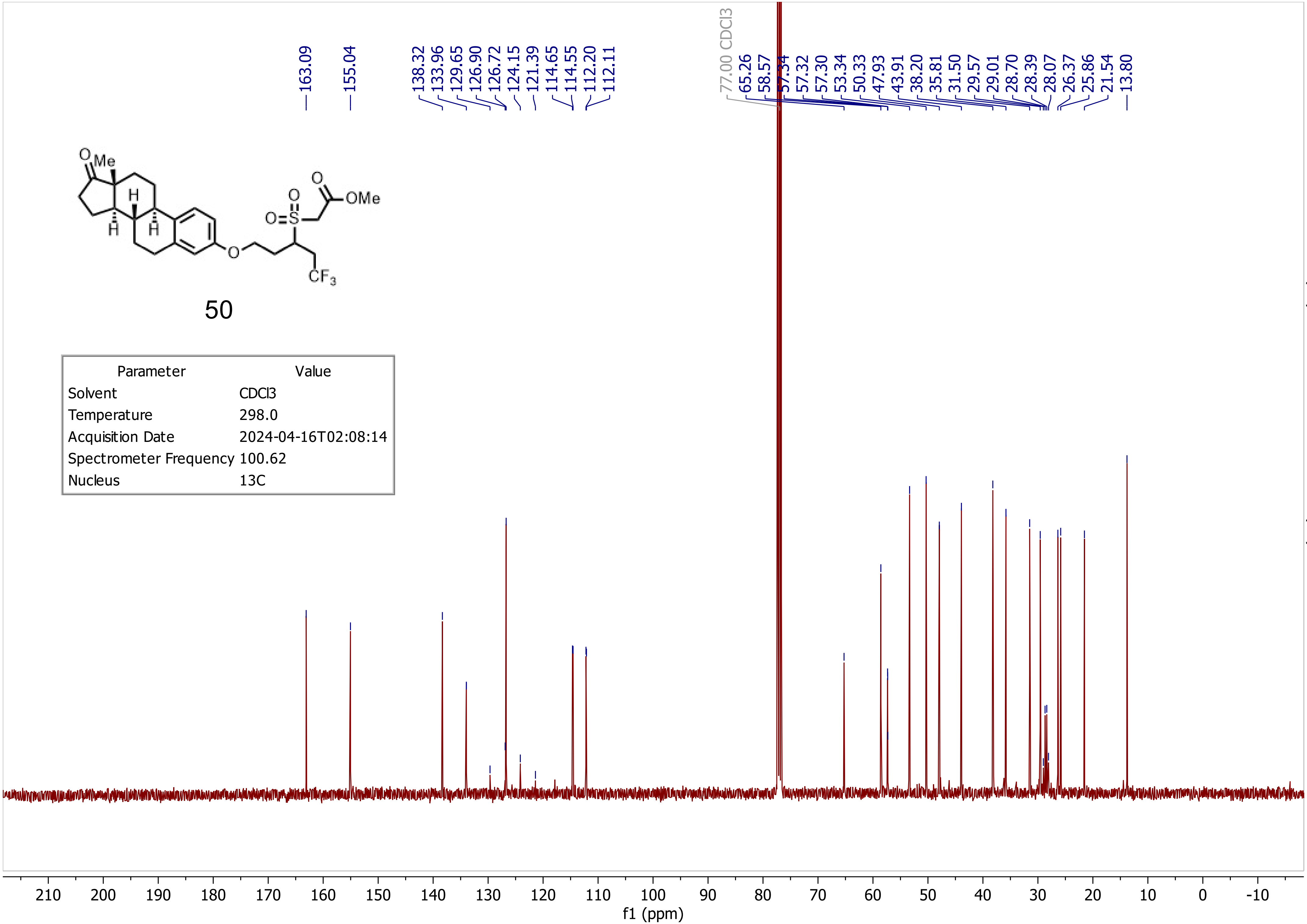

50

Parameter	Value
Solvent	CDCl3
Temperature	298.0
Acquisition Date	2024-04-16T02:08:14
Spectrometer Frequency	100.62
Nucleus	13C

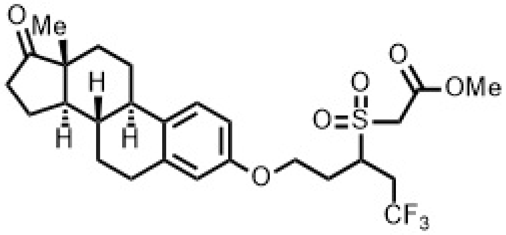

50

Parameter	Value
Solvent	CDCl3
Temperature	298.0
Acquisition Date	2024-12-03T15:24:33
Spectrometer Frequency	376.46
Nucleus	19F

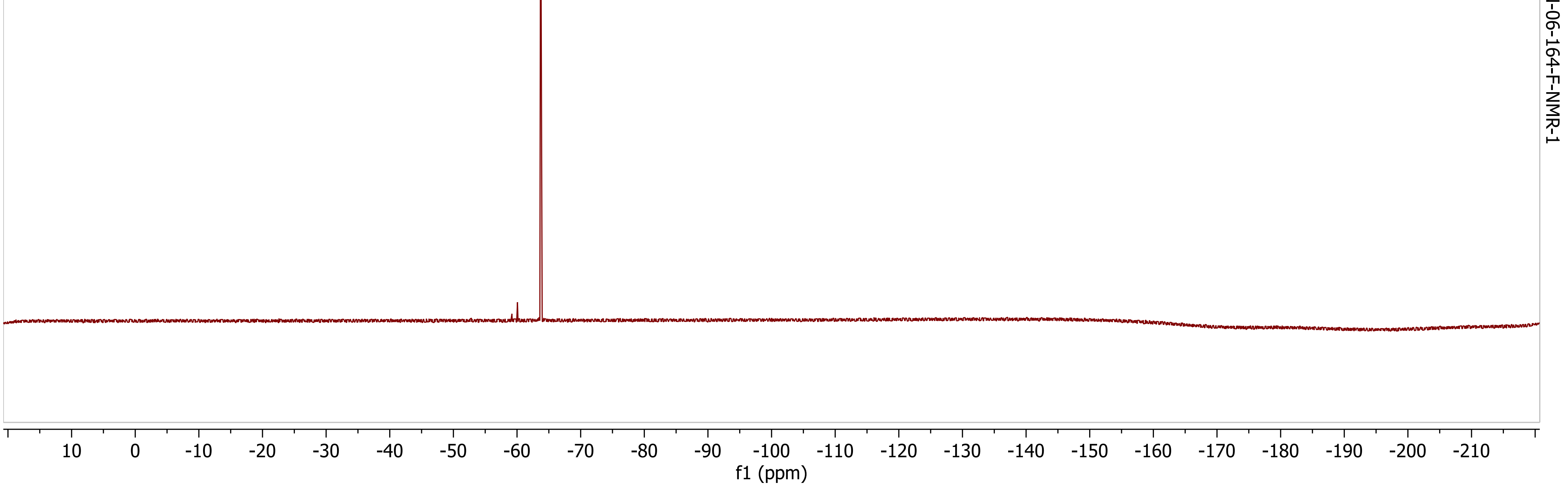

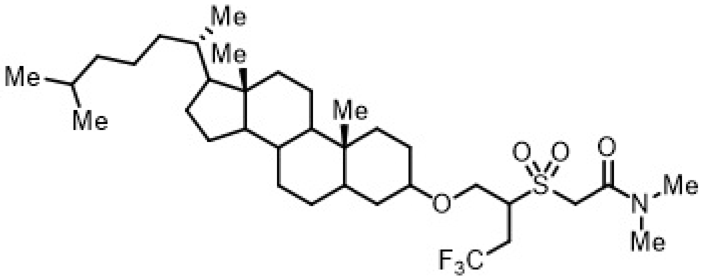

51

Parameter	Value
Solvent	CDCl3
Temperature	298.0
Acquisition Date	2024-09-08T01:59:22
Spectrometer Frequency	400.13
Nucleus	1H

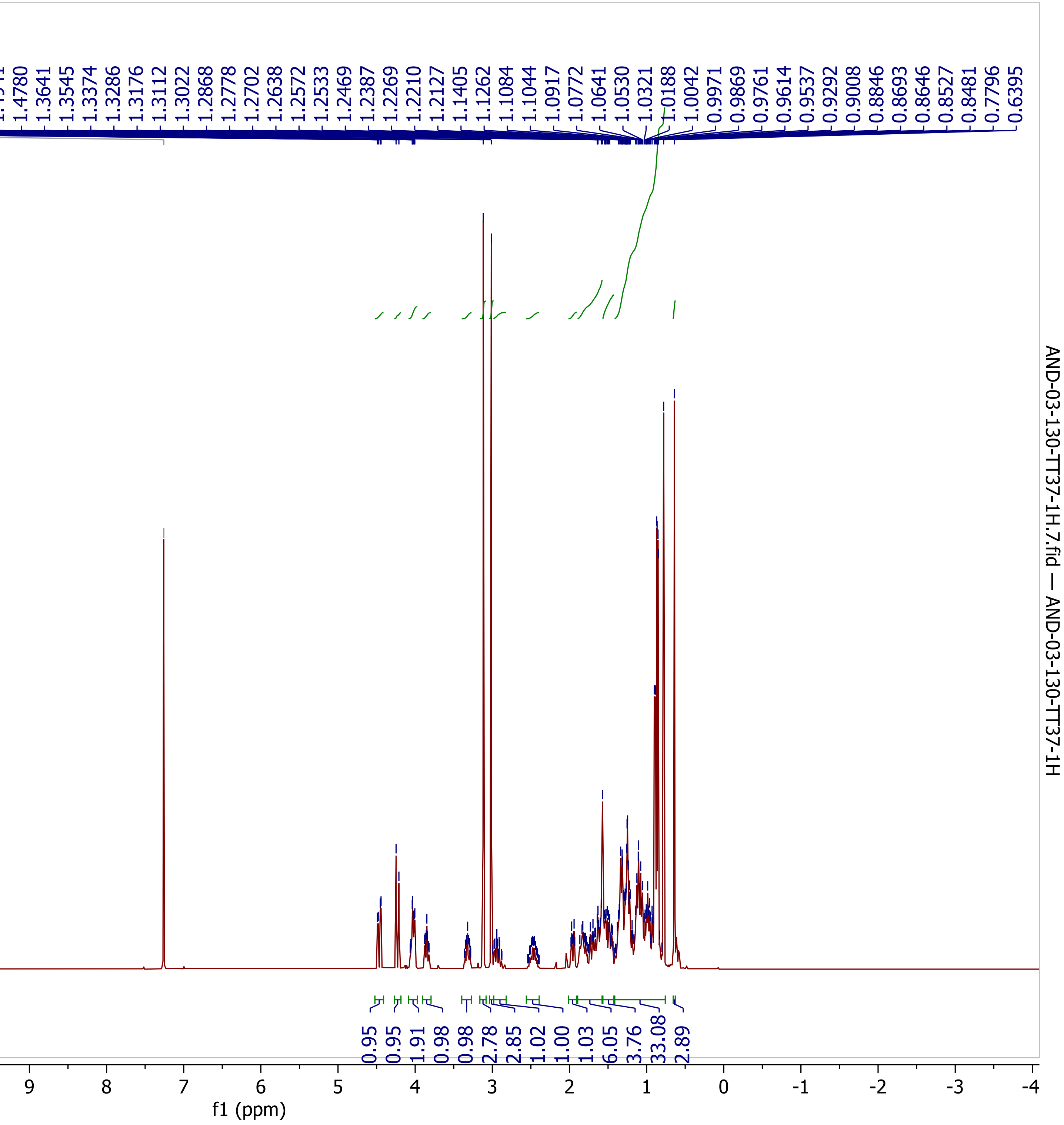

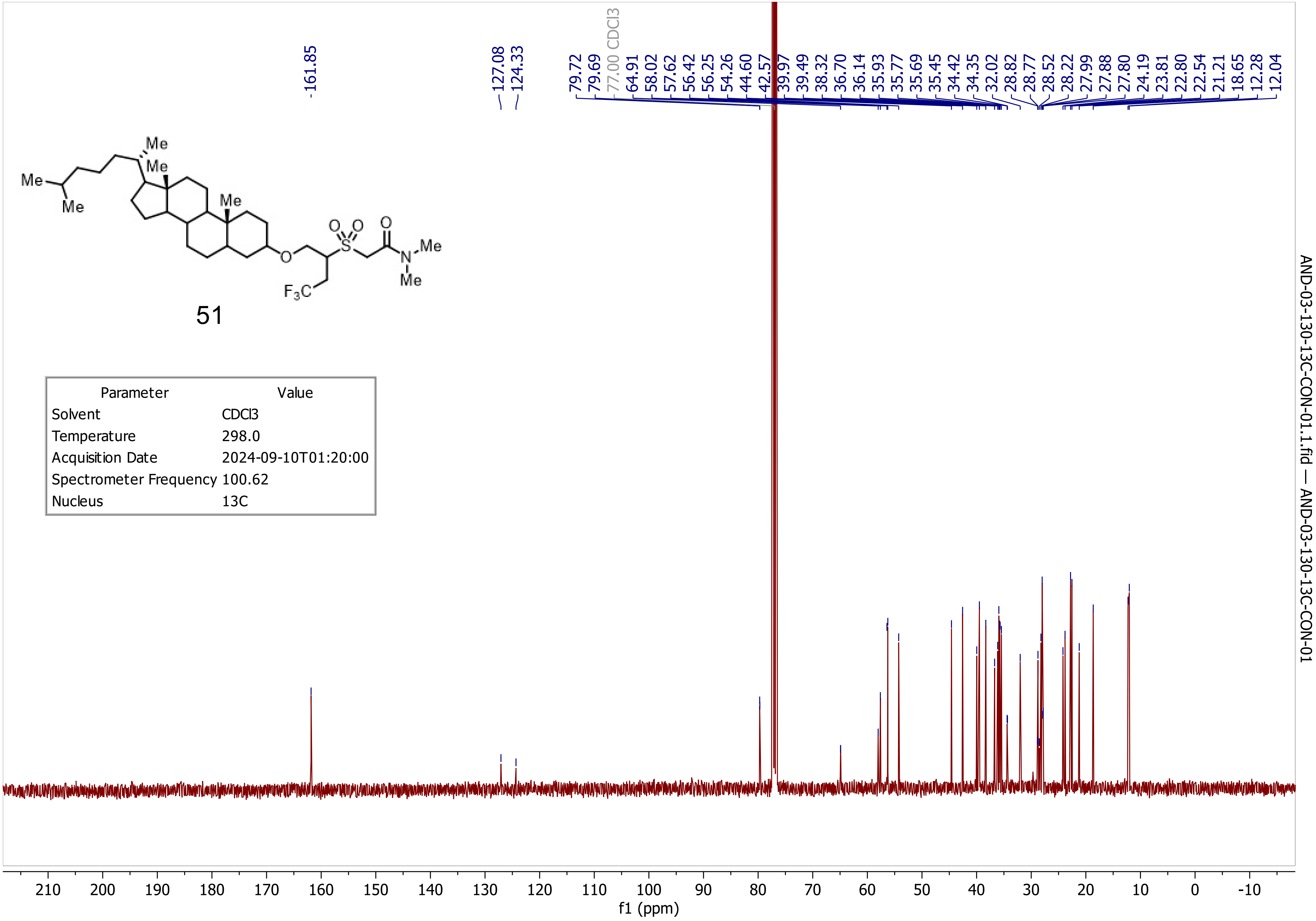

51

Parameter	Value
Solvent	CDCl3
Temperature	298.0
Acquisition Date	2024-09-10T01:20:00
Spectrometer Frequency	100.62
Nucleus	13C

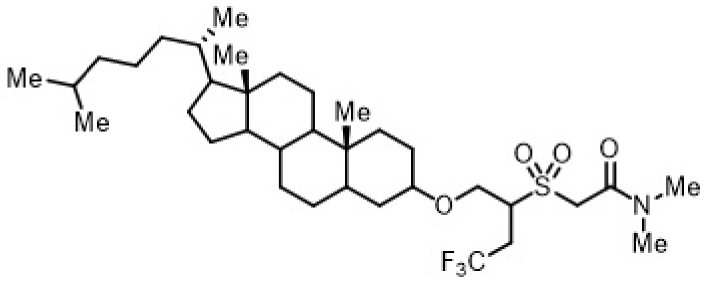

51

Parameter	Value
Solvent	CDCl3
Temperature	298.0
Acquisition Date	2024-09-08T00:55:46
Spectrometer Frequency	376.46
Nucleus	19F

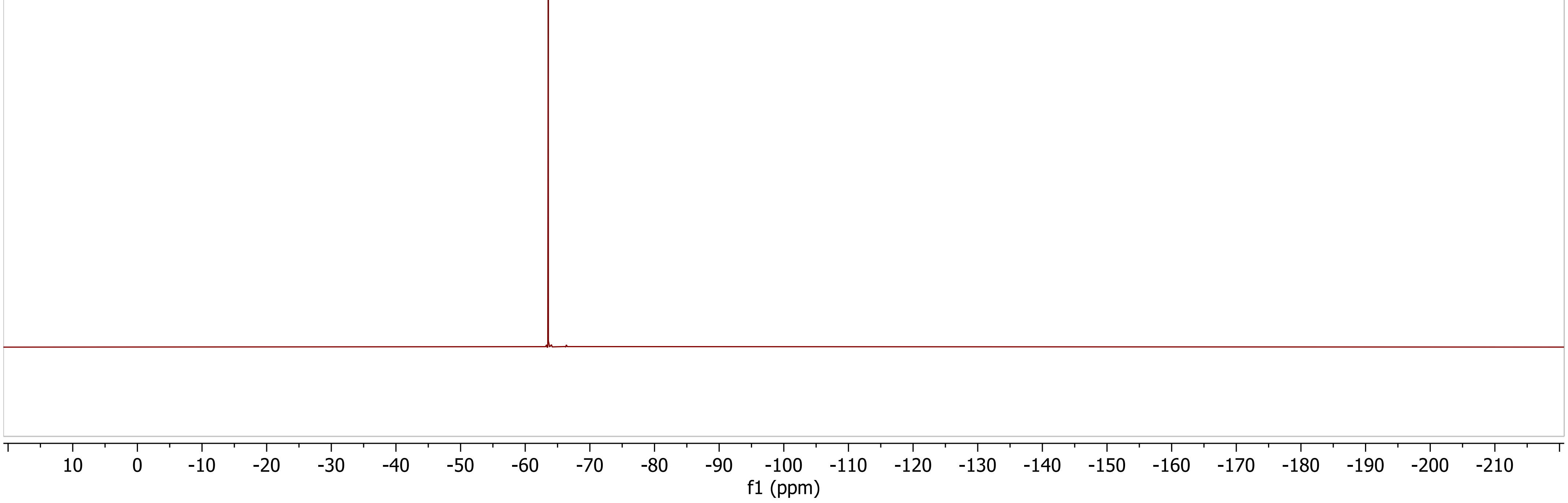

AND-03-130-TT37-19F.4.fid — AND-03-130-TT37-19F

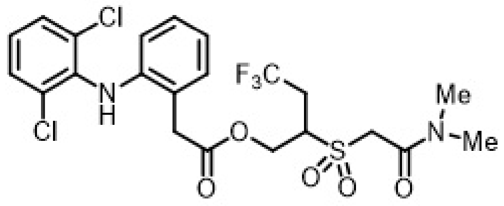

52

Parameter	Value
Solvent	CDCl3
Temperature	298.0
Acquisition Date	2024-08-25T18:03:20
Spectrometer Frequency	400.13
Nucleus	1H

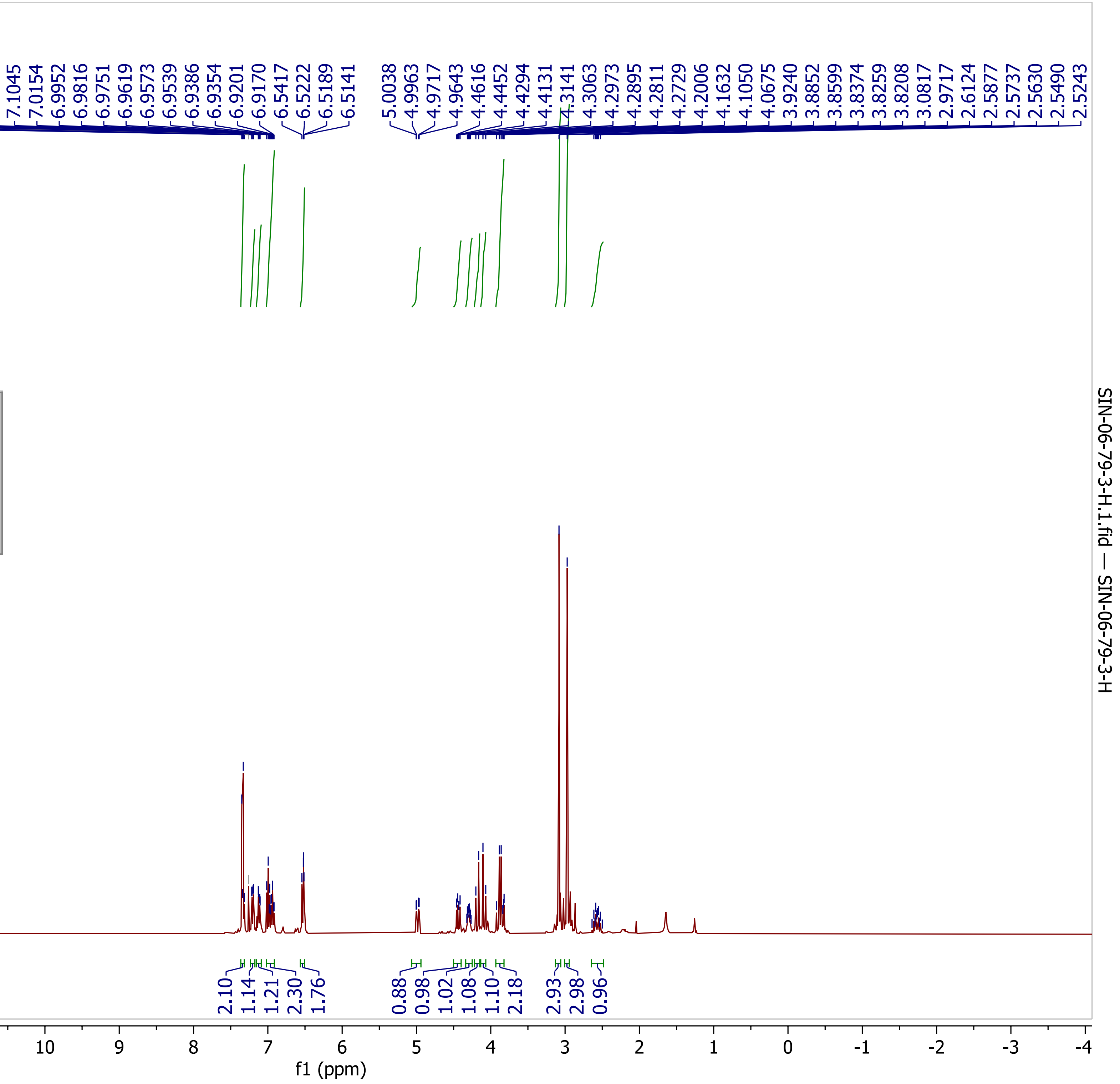

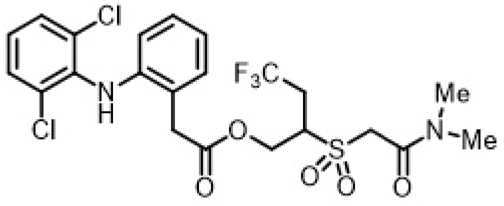

53

Parameter	Value
Solvent	CDCl3
Temperature	298.0
Acquisition Date	2024-08-26T01:06:07
Spectrometer Frequency	100.62
Nucleus	13C

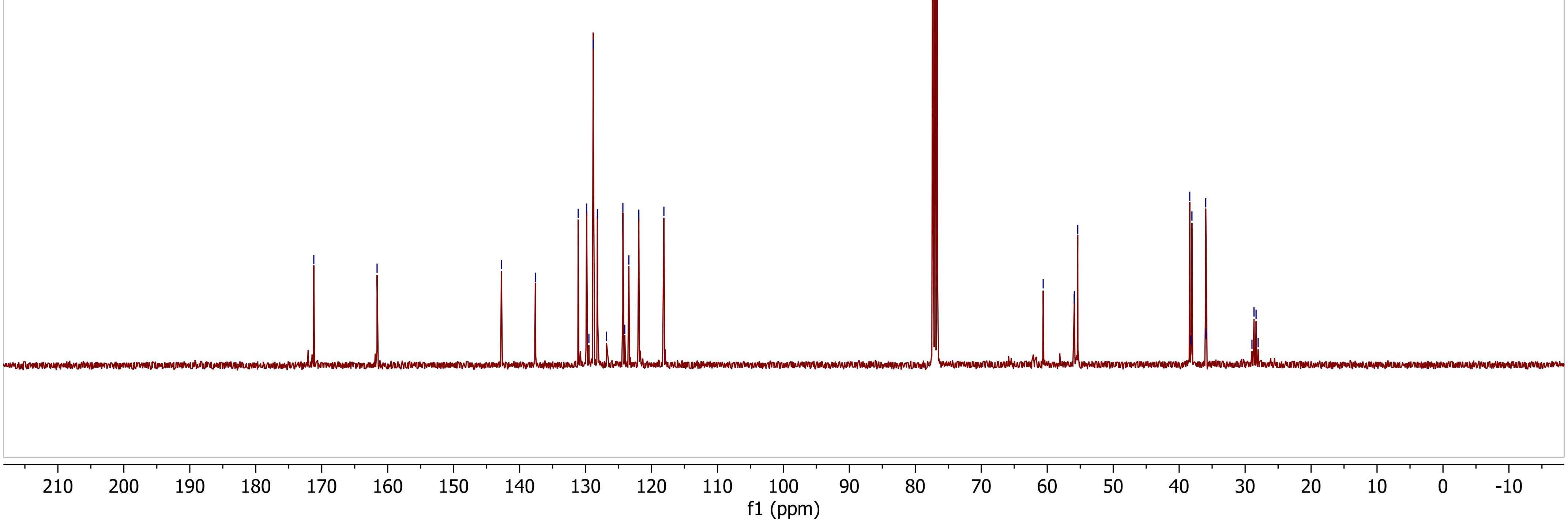

SIN-06-79-3-C13.5.fid — SIN-06-79-3-C13

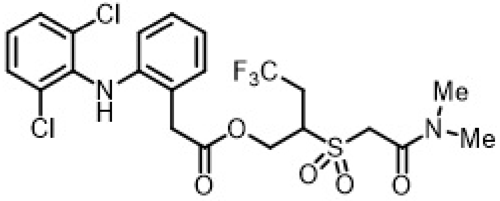

53

Parameter	Value
Solvent	CDCl3
Temperature	298.0
Acquisition Date	2024-08-26T00:05:47
Spectrometer Frequency	376.46
Nucleus	19F

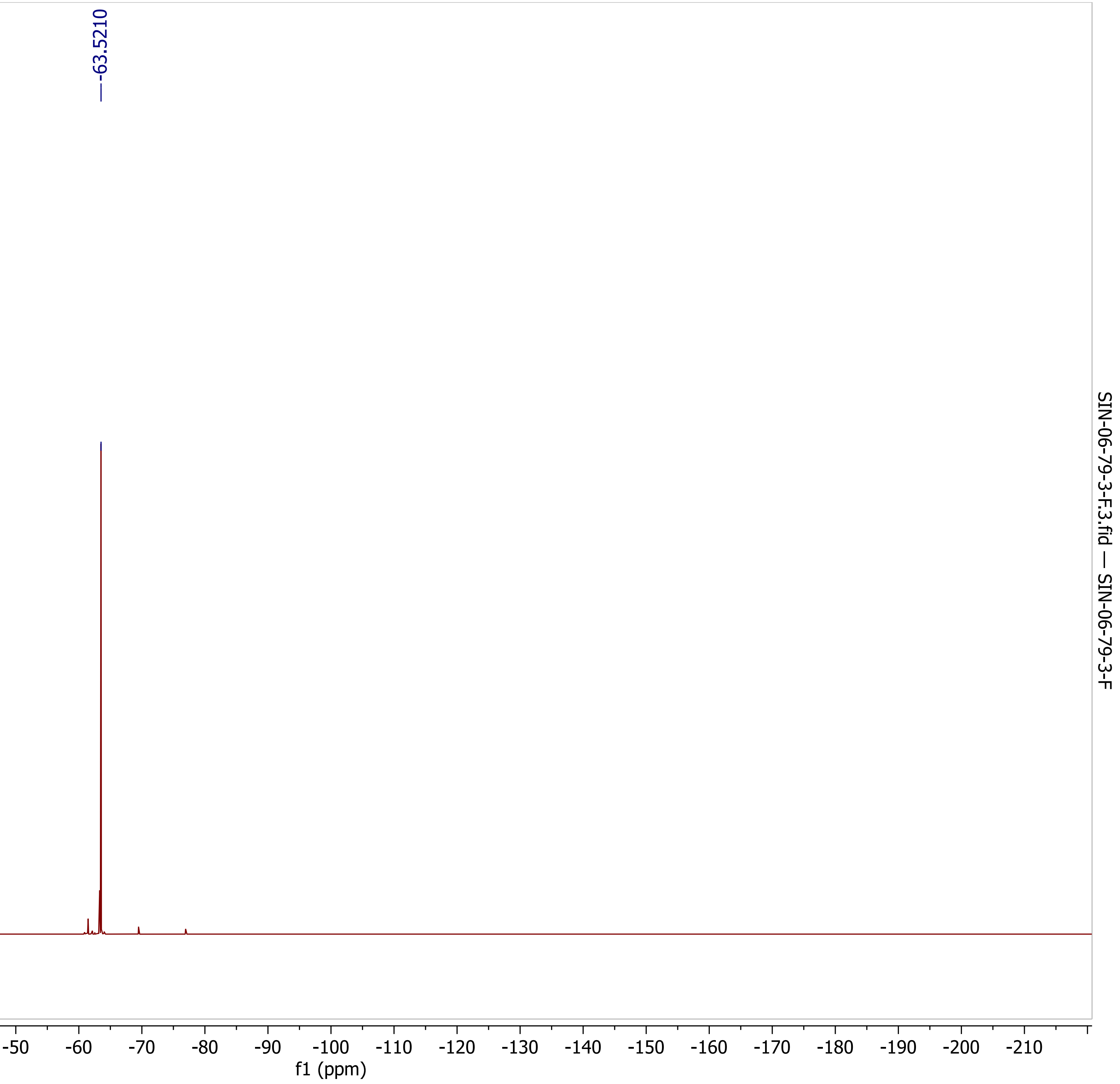

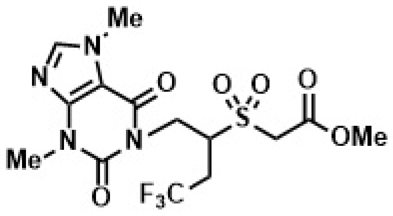

54

Parameter	Value
Solvent	CDCl3
Temperature	298.0
Acquisition Date	2024-10-19T10:54:09
Spectrometer Frequency	400.13
Nucleus	1H

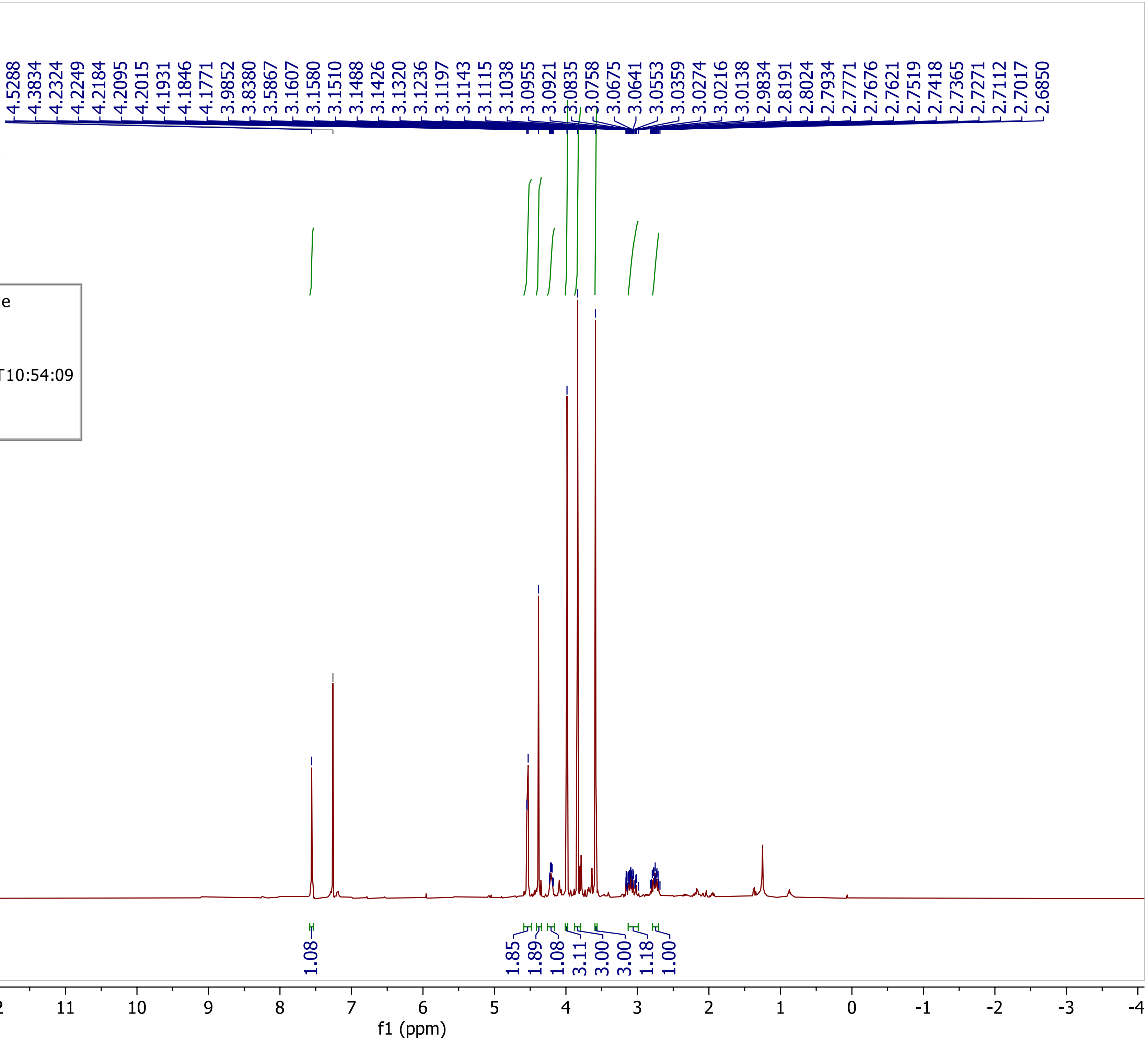

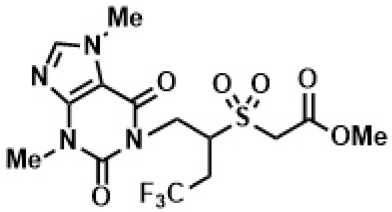

54

Parameter	Value
Solvent	CDCl3
Temperature	298.0
Acquisition Date	2024-10-19T01:56:32
Spectrometer Frequency	100.62
Nucleus	13C

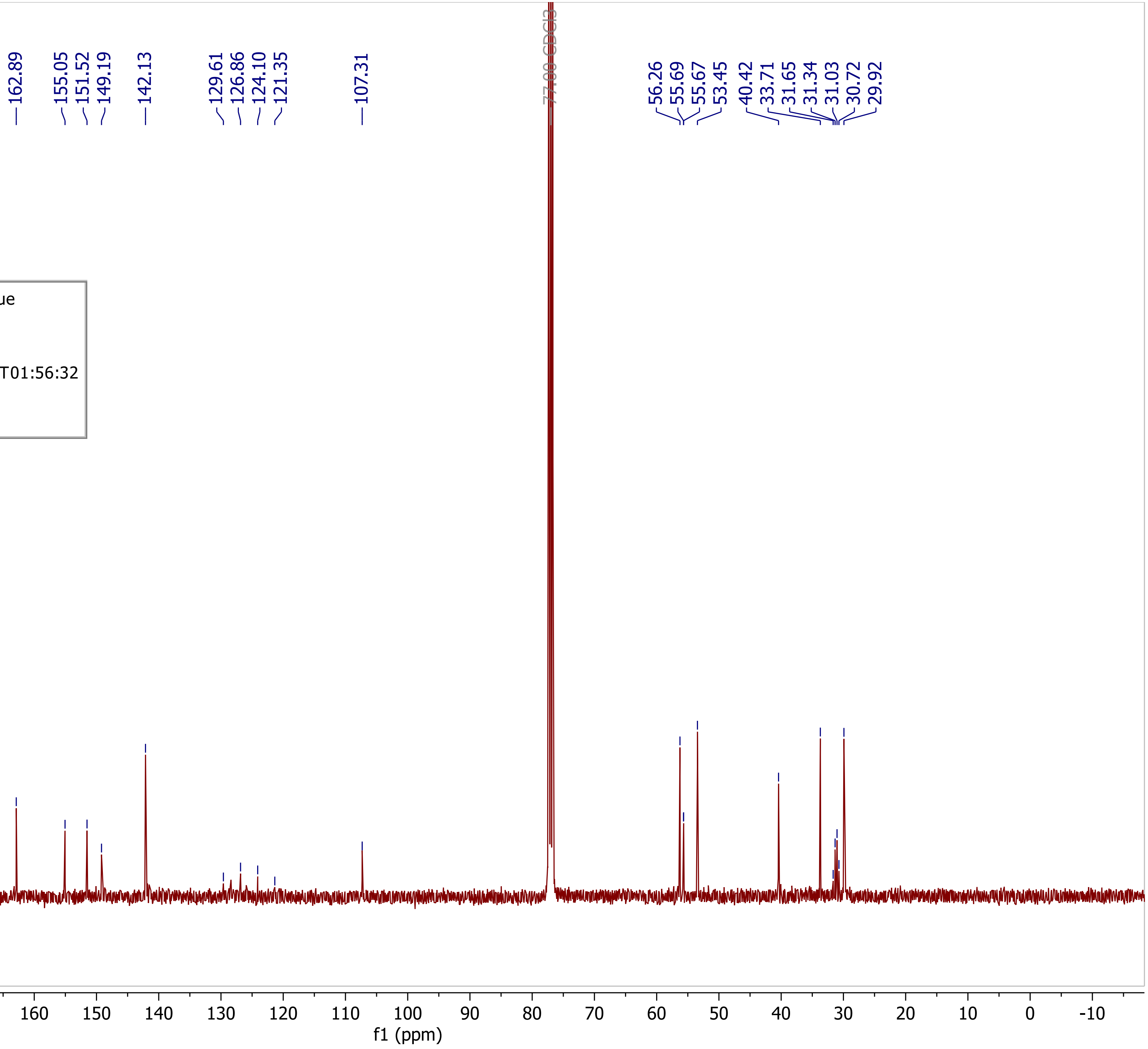

SIN-06-152-L1-1-c13-1.7.fid — SIN-06-152-L1-1-f

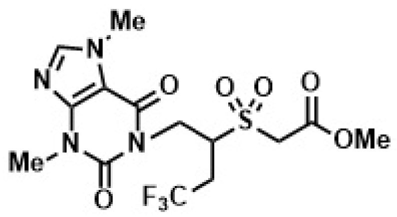

54

Parameter	Value
Solvent	CDCl3
Temperature	298.0
Acquisition Date	2024-10-18T14:40:45
Spectrometer Frequency	376.46
Nucleus	19F

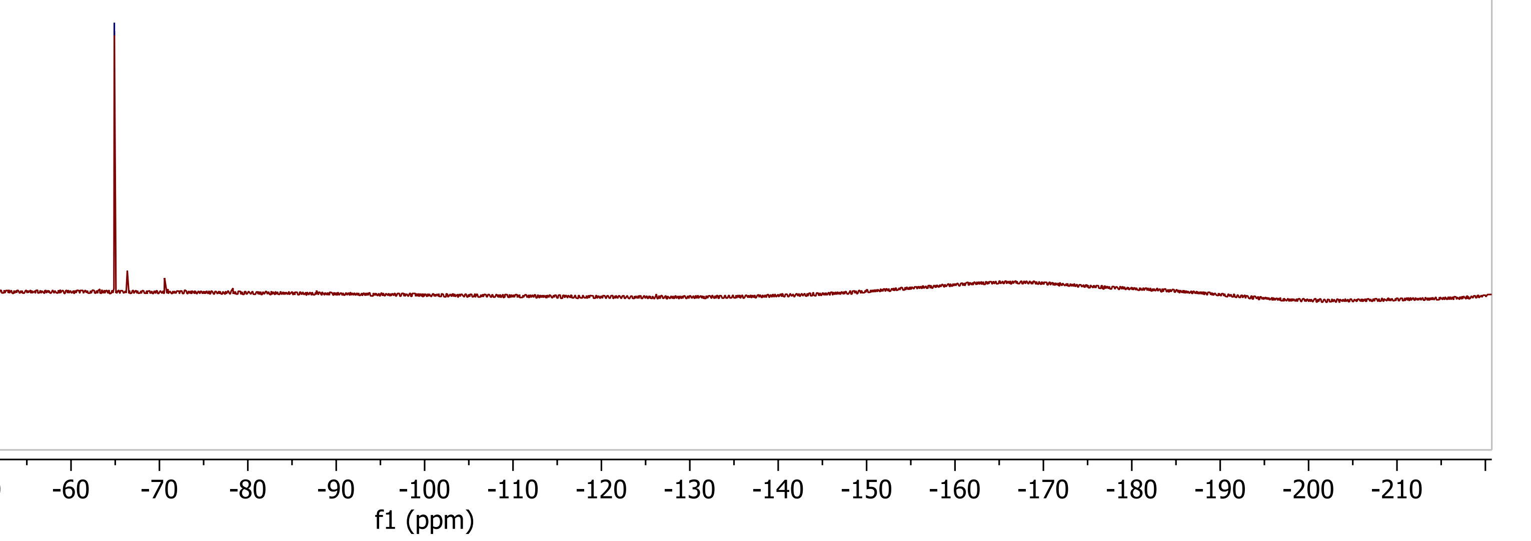

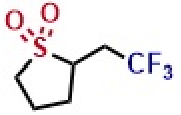

54

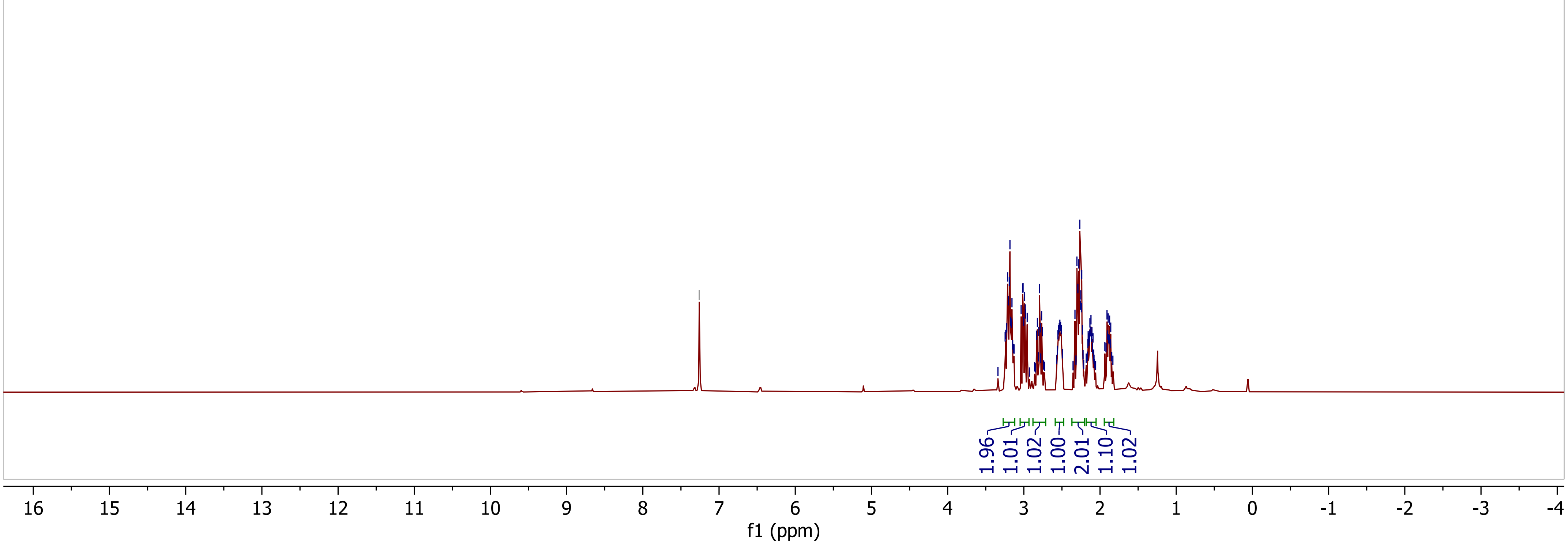

Parameter	Value
Solvent	CDCl3
Temperature	298.0
Acquisition Date	2024-01-09T18:09:21
Spectrometer Frequency	400.13
Nucleus	1H

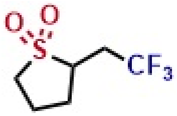

54

Parameter	Value
Solvent	CDCl3
Temperature	298.0
Acquisition Date	2024-01-09T23:58:25
Spectrometer Frequency	100.62
Nucleus	13C

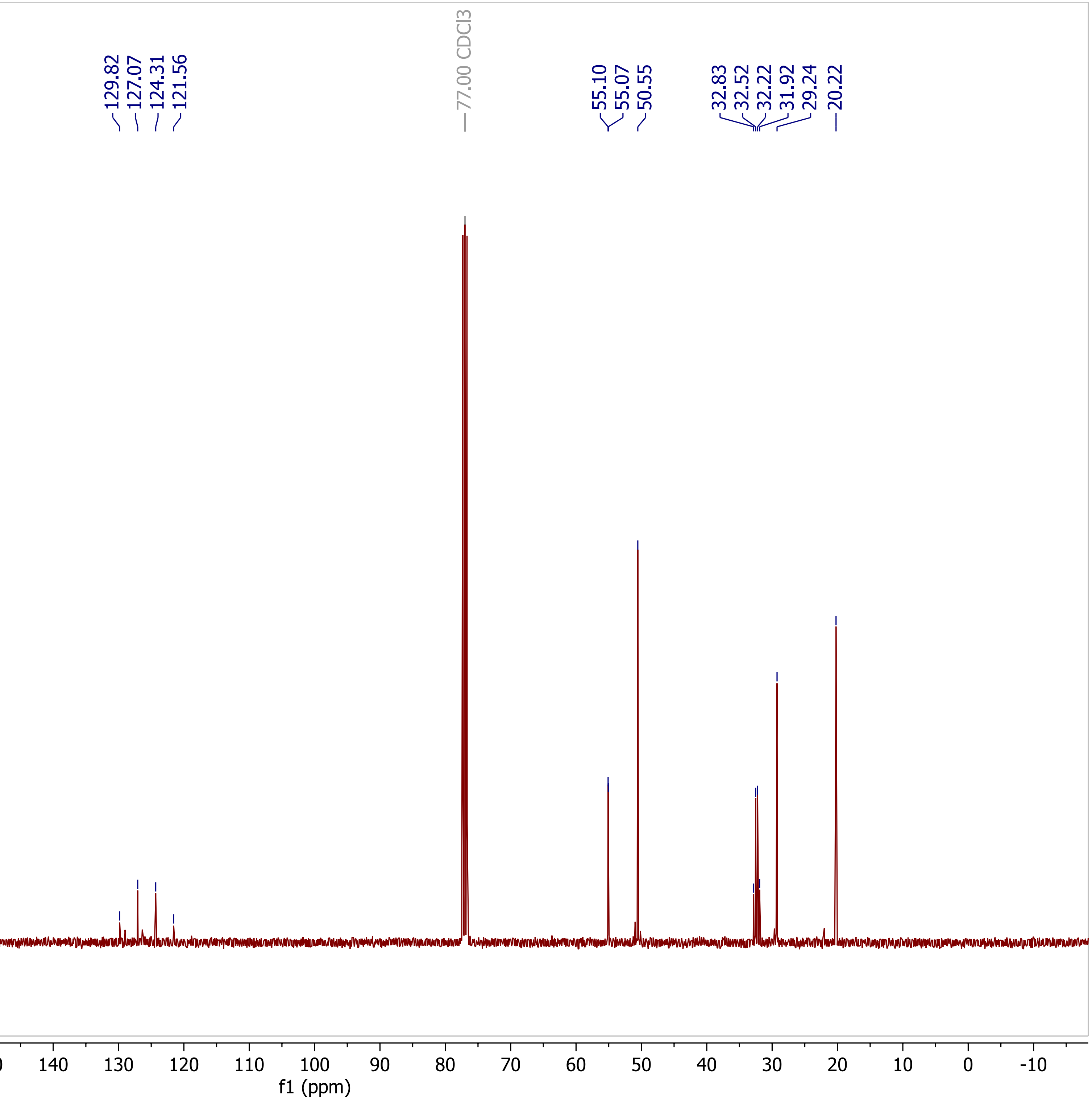

SIN-05-01-Pure-C.7.fid — SIN-05-01-Pure-C

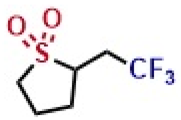

54

Parameter	Value
Solvent	CDCl3
Temperature	298.0
Acquisition Date	2024-01-09T18:13:27
Spectrometer Frequency	376.46
Nucleus	19F

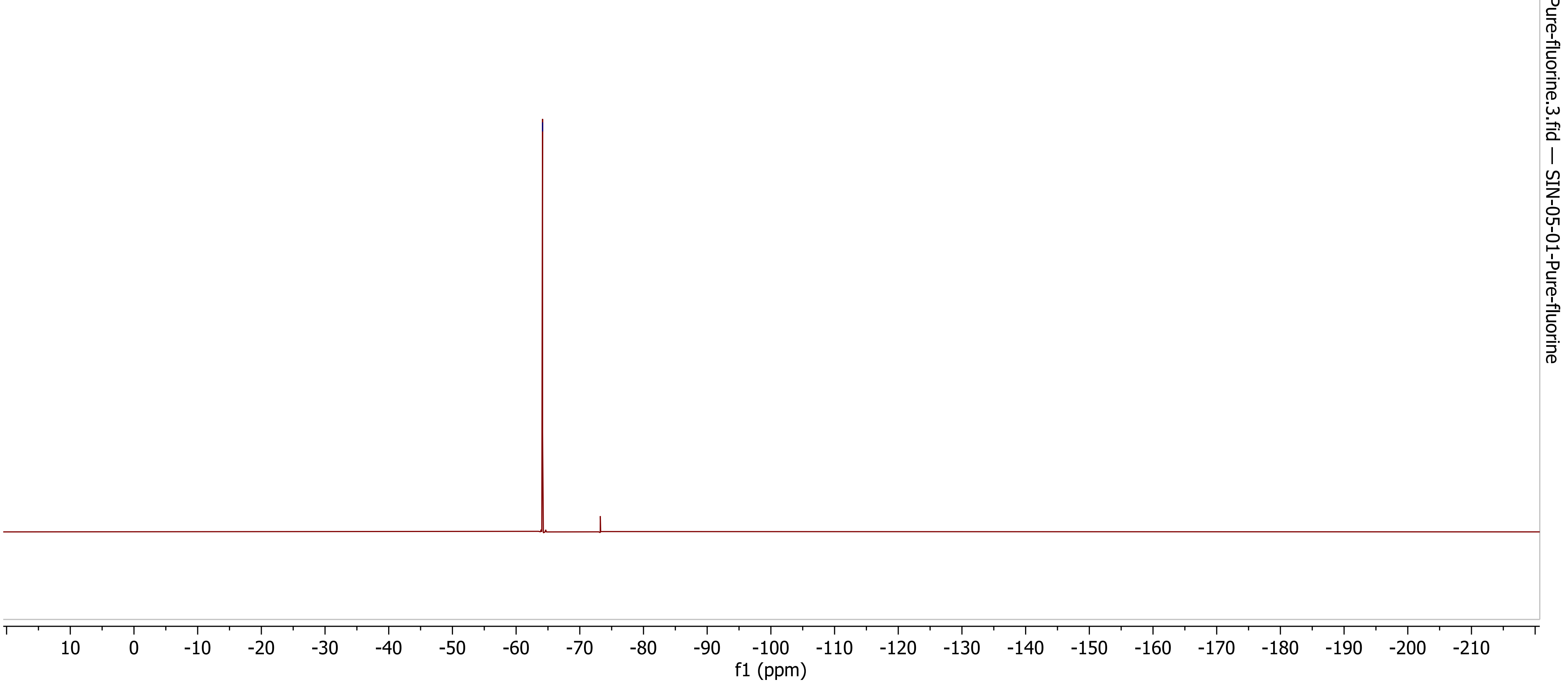

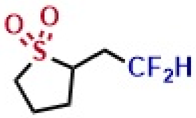

55

Parameter	Value
Solvent	CDCl3
Temperature	298.0
Acquisition Date	2024-01-19T16:03:06
Spectrometer Frequency	400.13
Nucleus	1H

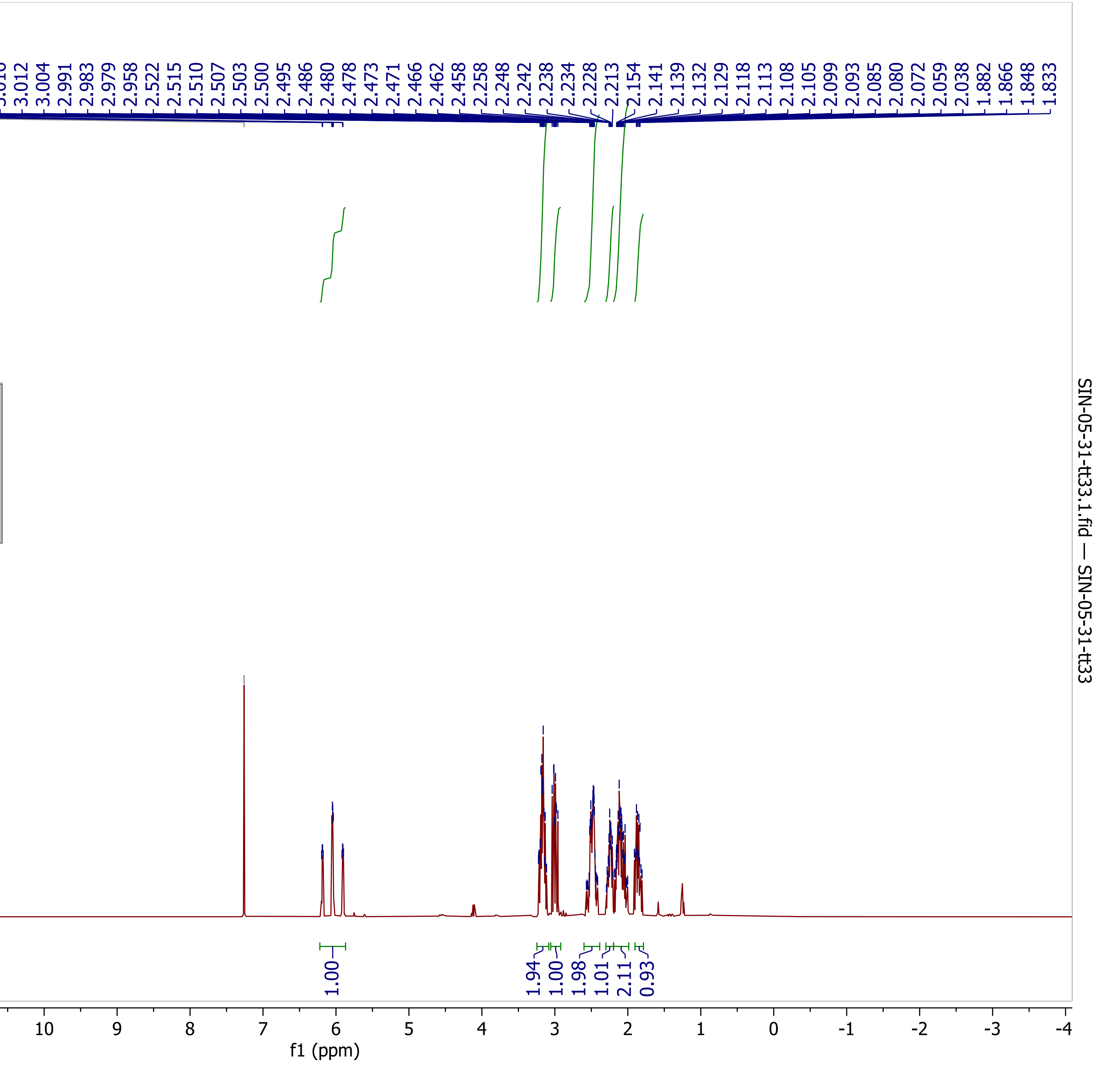

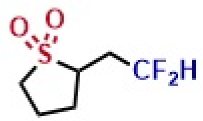

55

Parameter	Value
Solvent	CDCl3
Temperature	298.0
Acquisition Date	2024-03-10T00:19:31
Spectrometer Frequency	100.62
Nucleus	13C

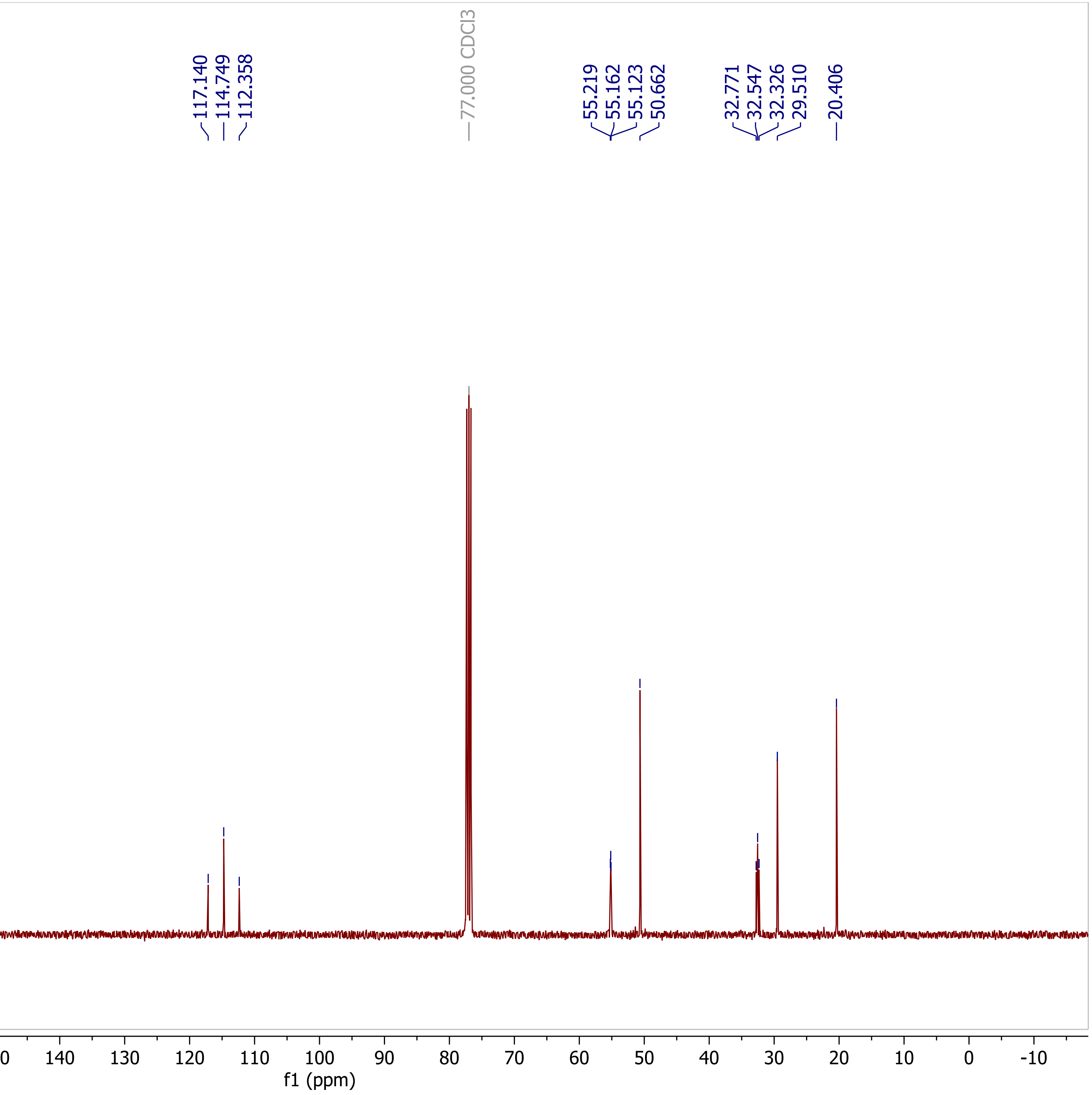

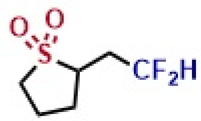

55

Parameter	Value
Solvent	CDCl3
Temperature	298.0
Acquisition Date	2024-01-19T16:05:41
Spectrometer Frequency	376.46
Nucleus	19F

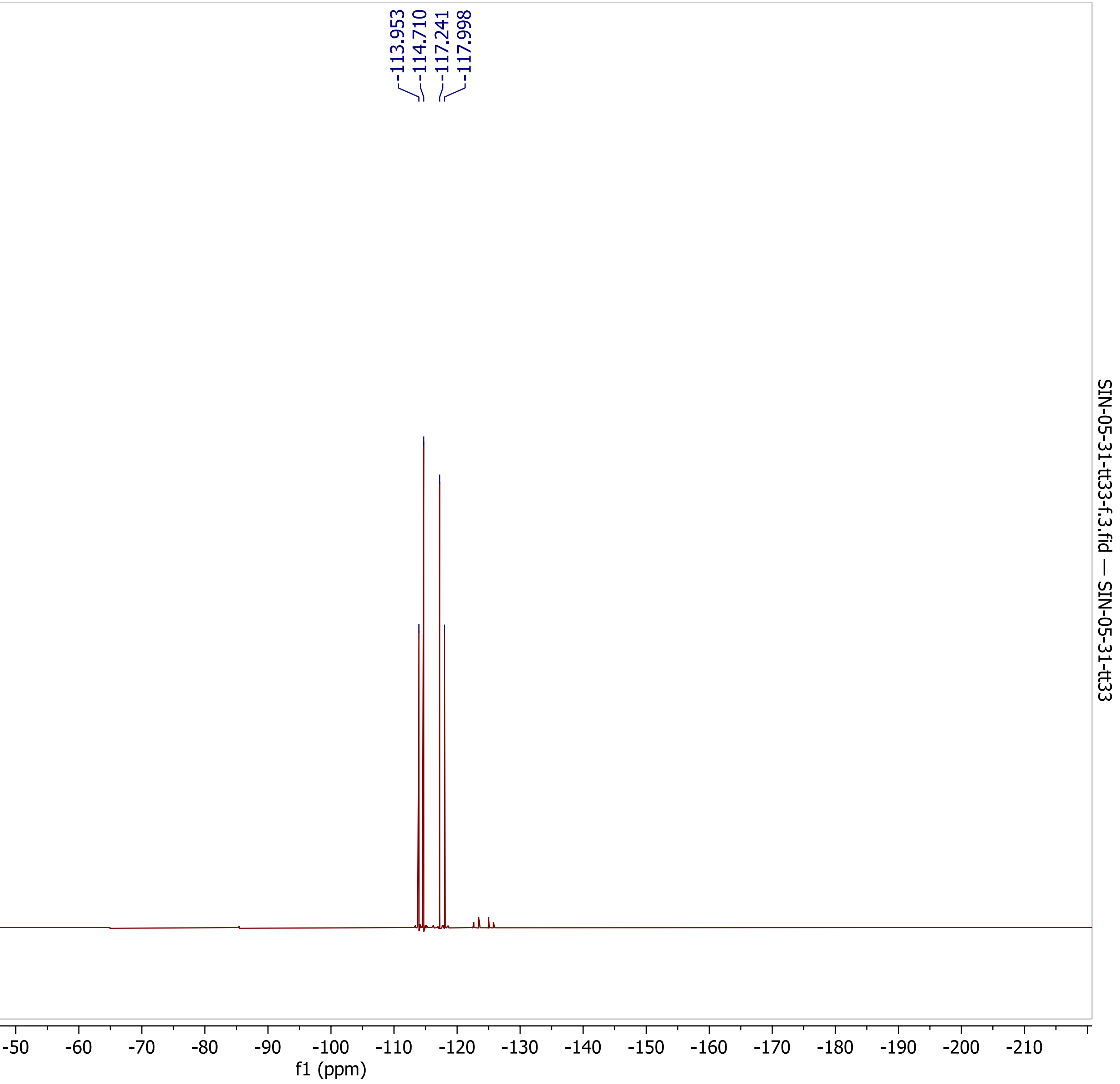

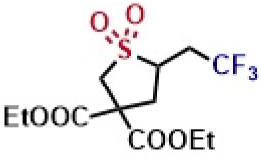

56

Parameter	Value
Solvent	CDCl3
Temperature	298.0
Acquisition Date	2024-02-28T22:12:13
Spectrometer Frequency	400.13
Nucleus	1H

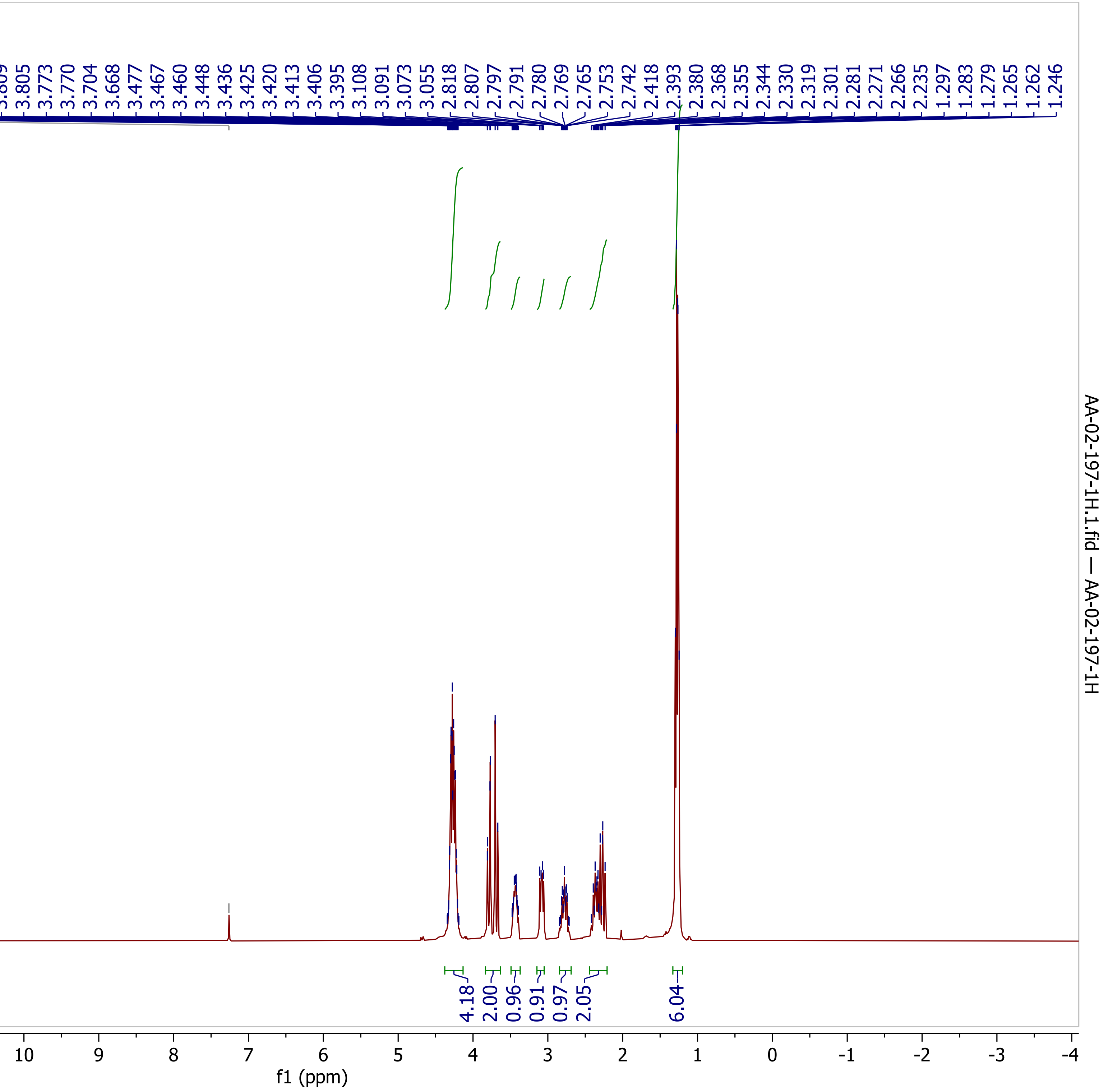

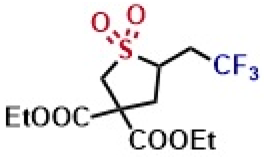

56

Parameter	Value
Solvent	CDCl3
Temperature	298.0
Acquisition Date	2024-02-28T23:23:13
Spectrometer Frequency	100.62
Nucleus	13C

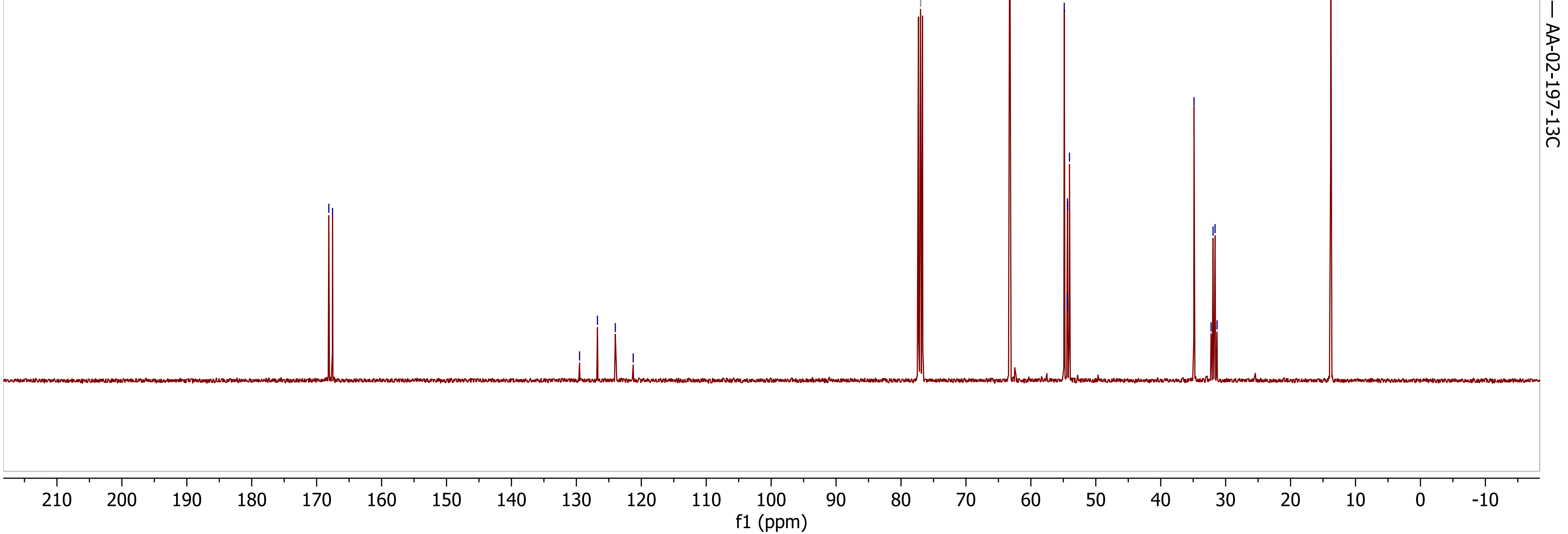

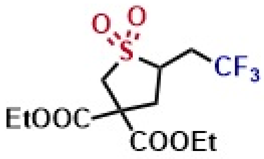

56

Parameter	Value
Solvent	CDCl3
Temperature	298.0
Acquisition Date	2024-02-28T23:27:37
Spectrometer Frequency	376.46
Nucleus	19F

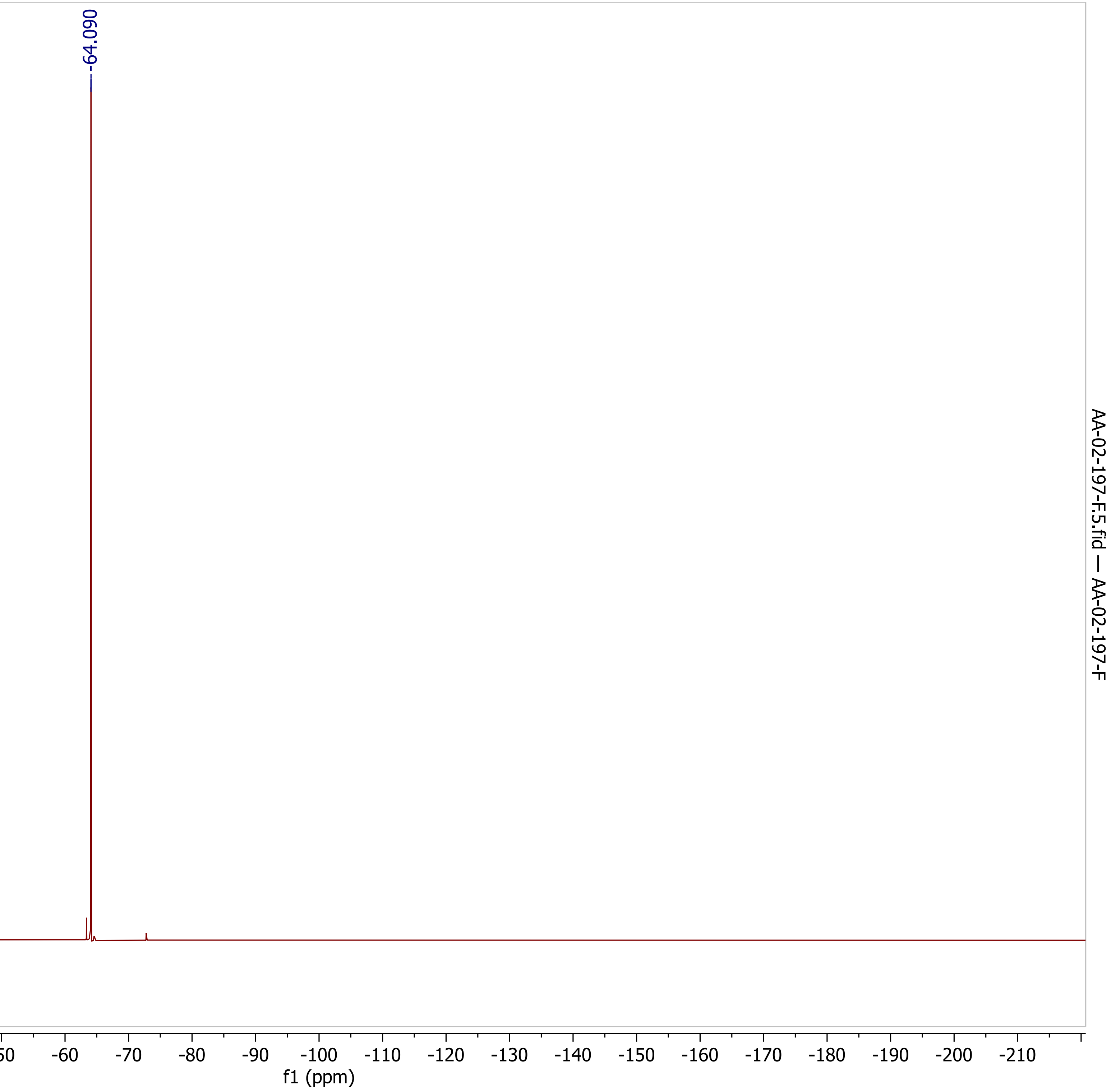

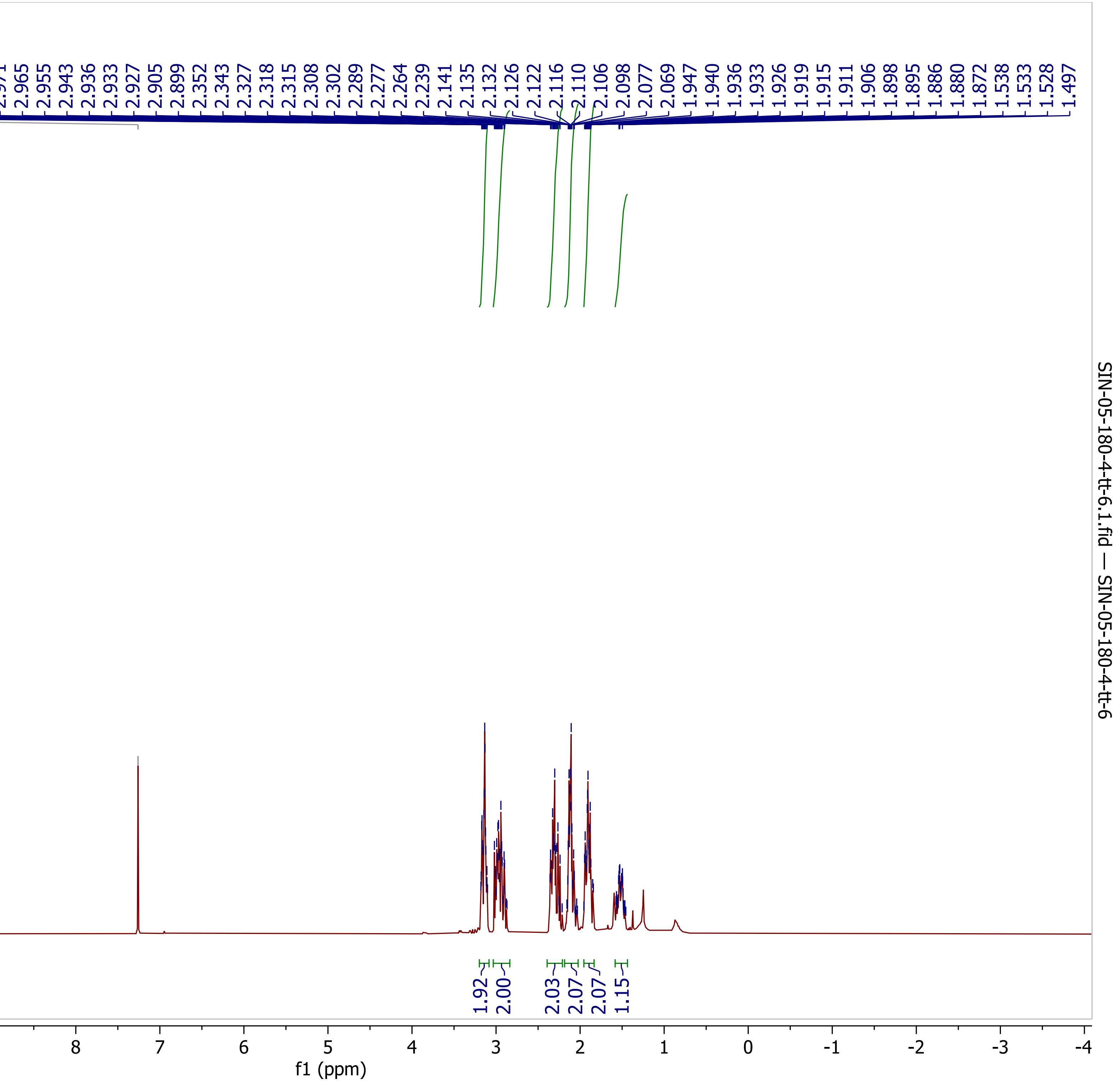

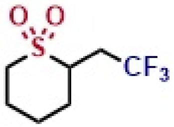

57

Parameter	Value
Solvent	CDCl3
Temperature	298.0
Acquisition Date	2024-04-24T10:10:08
Spectrometer Frequency	400.13
Nucleus	1H

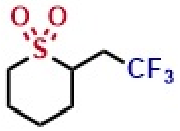

57

Parameter	Value
Solvent	CDCl3
Temperature	298.0
Acquisition Date	2024-04-27T01:43:41
Spectrometer Frequency	100.62
Nucleus	13C

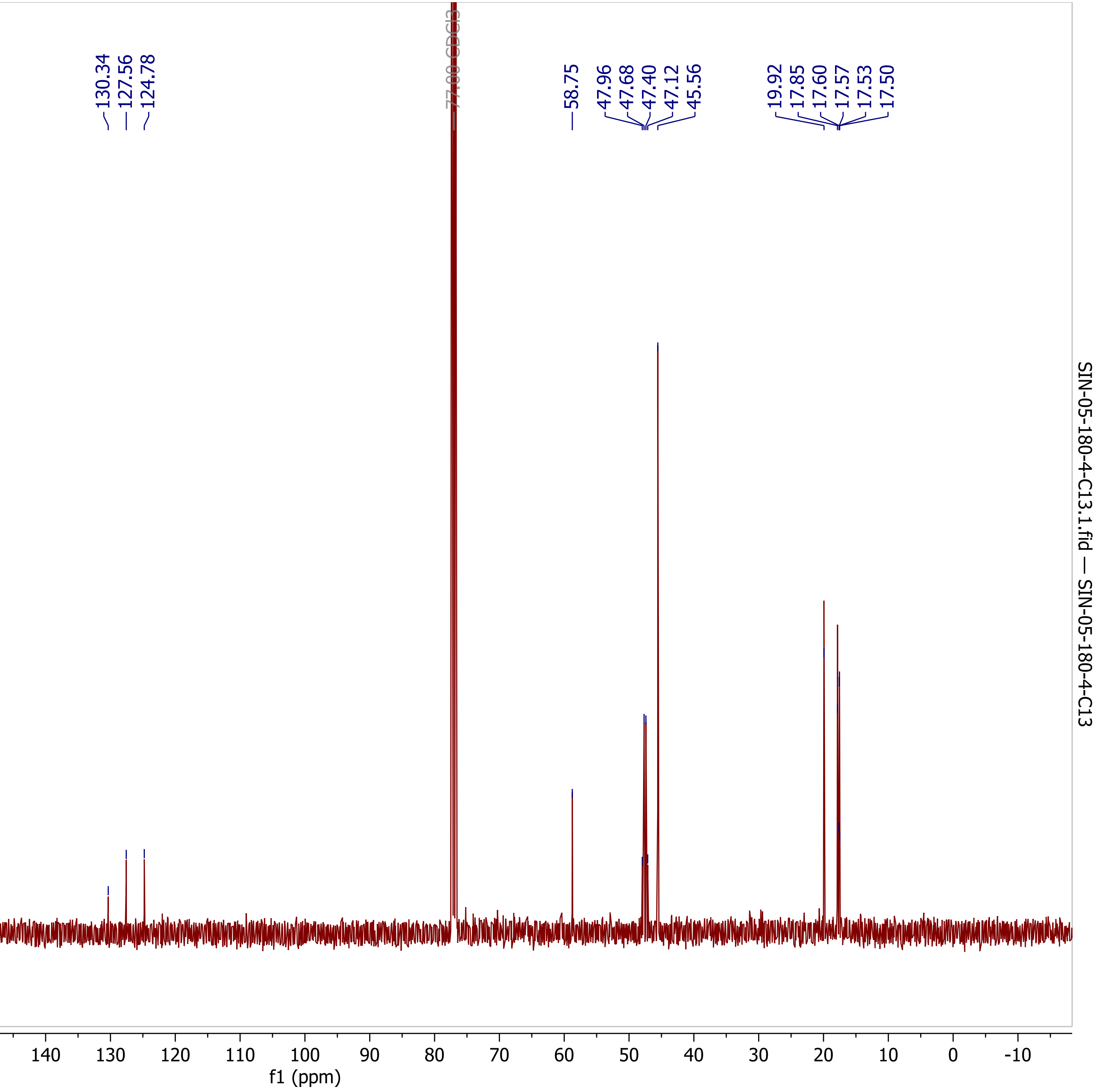

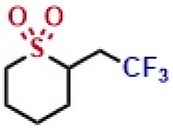

57

Parameter	Value
Solvent	CDCl3
Temperature	298.0
Acquisition Date	2024-04-25T16:10:17
Spectrometer Frequency	376.46
Nucleus	19F

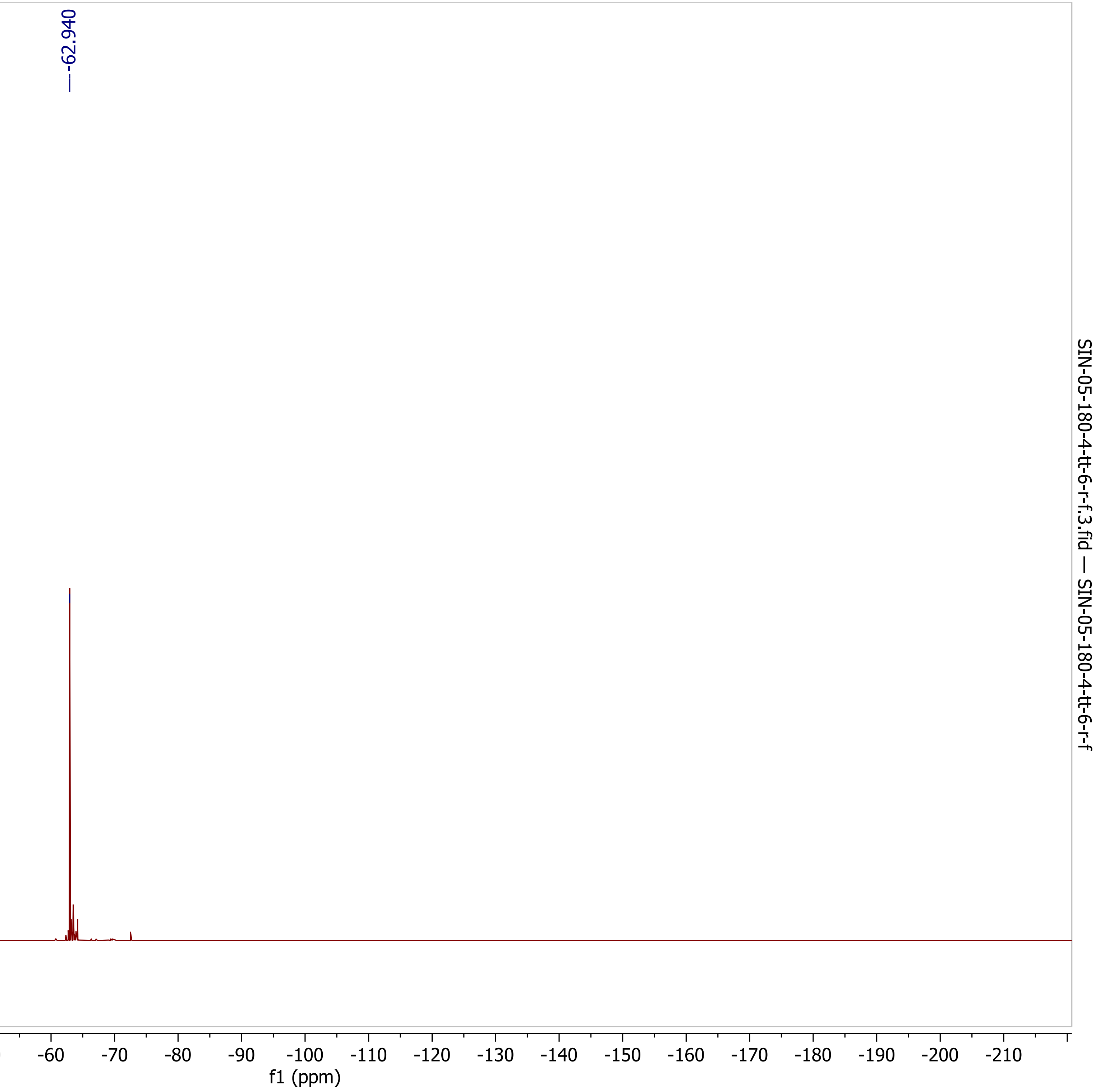

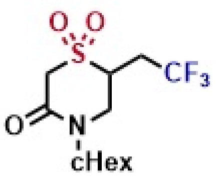

58

Parameter	Value
Solvent	CDCl3
Temperature	298.0
Acquisition Date	2024-03-19T19:17:10
Spectrometer Frequency	400.13
Nucleus	1H

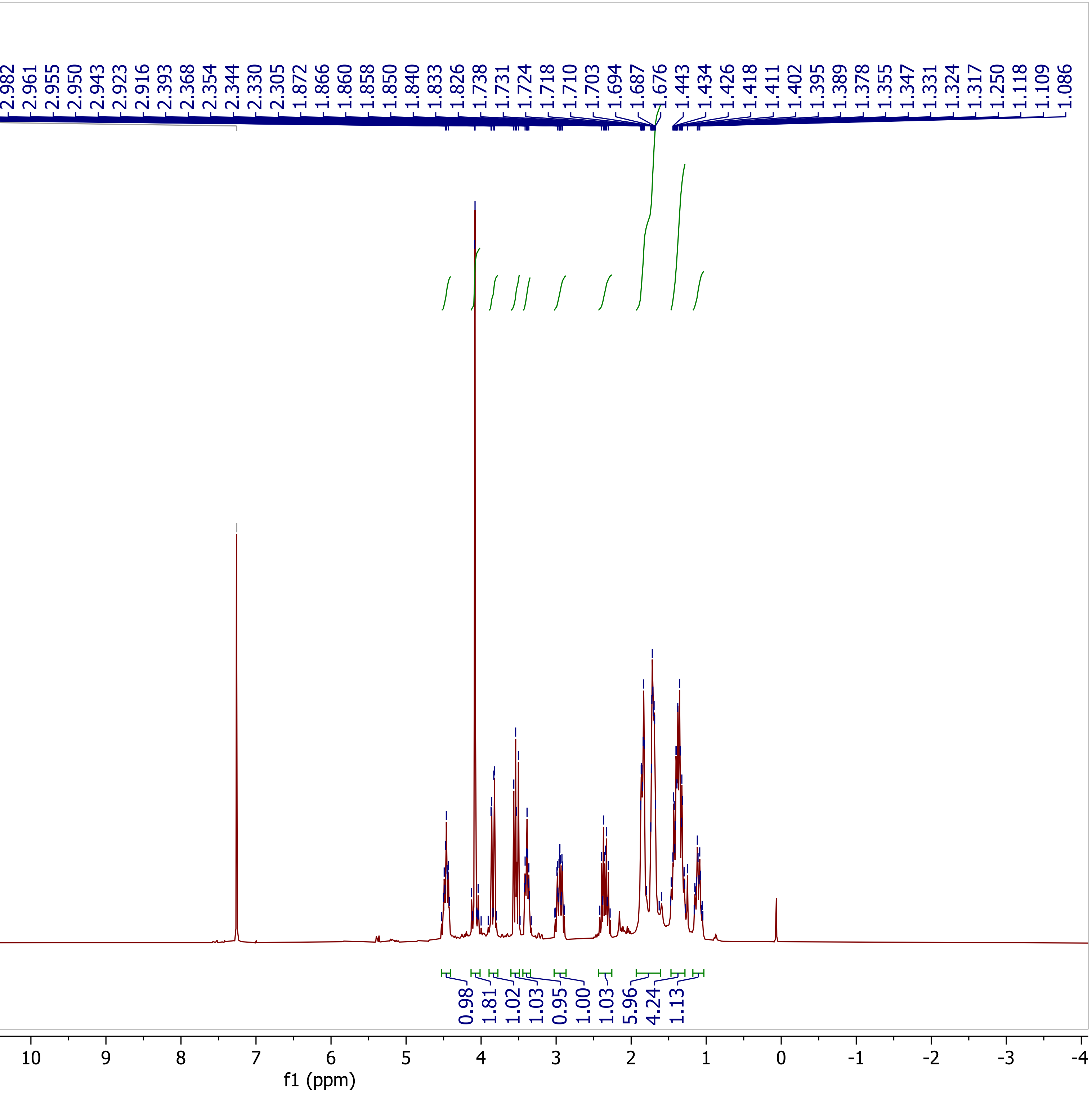

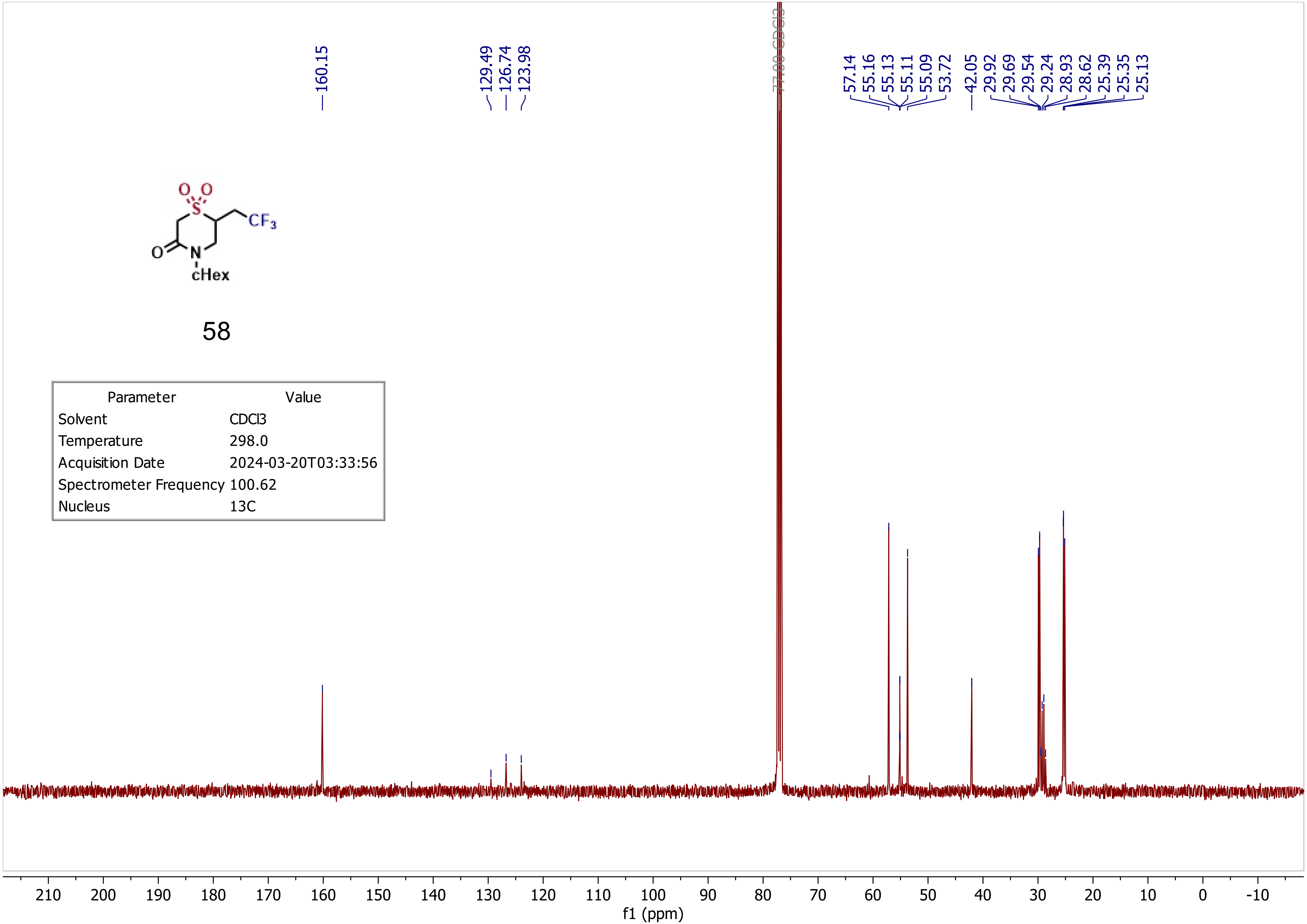

58

Parameter	Value
Solvent	CDCl3
Temperature	298.0
Acquisition Date	2024-03-20T03:33:56
Spectrometer Frequency	100.62
Nucleus	13C

SIN-05-48-1-C13.5.fid — SIN-05-48-1-C13

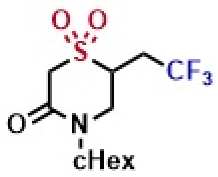

58

Parameter	Value
Solvent	CDCl3
Temperature	298.0
Acquisition Date	2024-03-20T03:35:59
Spectrometer Frequency	376.46
Nucleus	19F

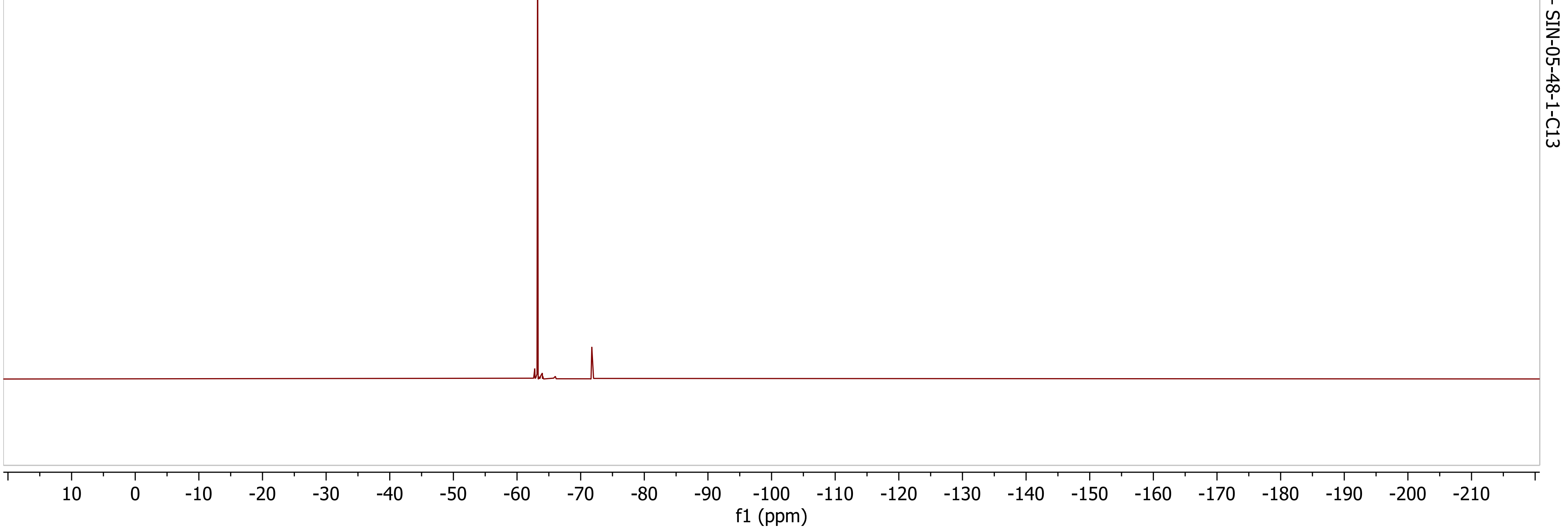

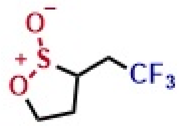

59

Parameter	Value
Solvent	CDCl3
Temperature	298.0
Acquisition Date	2024-01-16T11:48:58
Spectrometer Frequency	400.13
Nucleus	1H

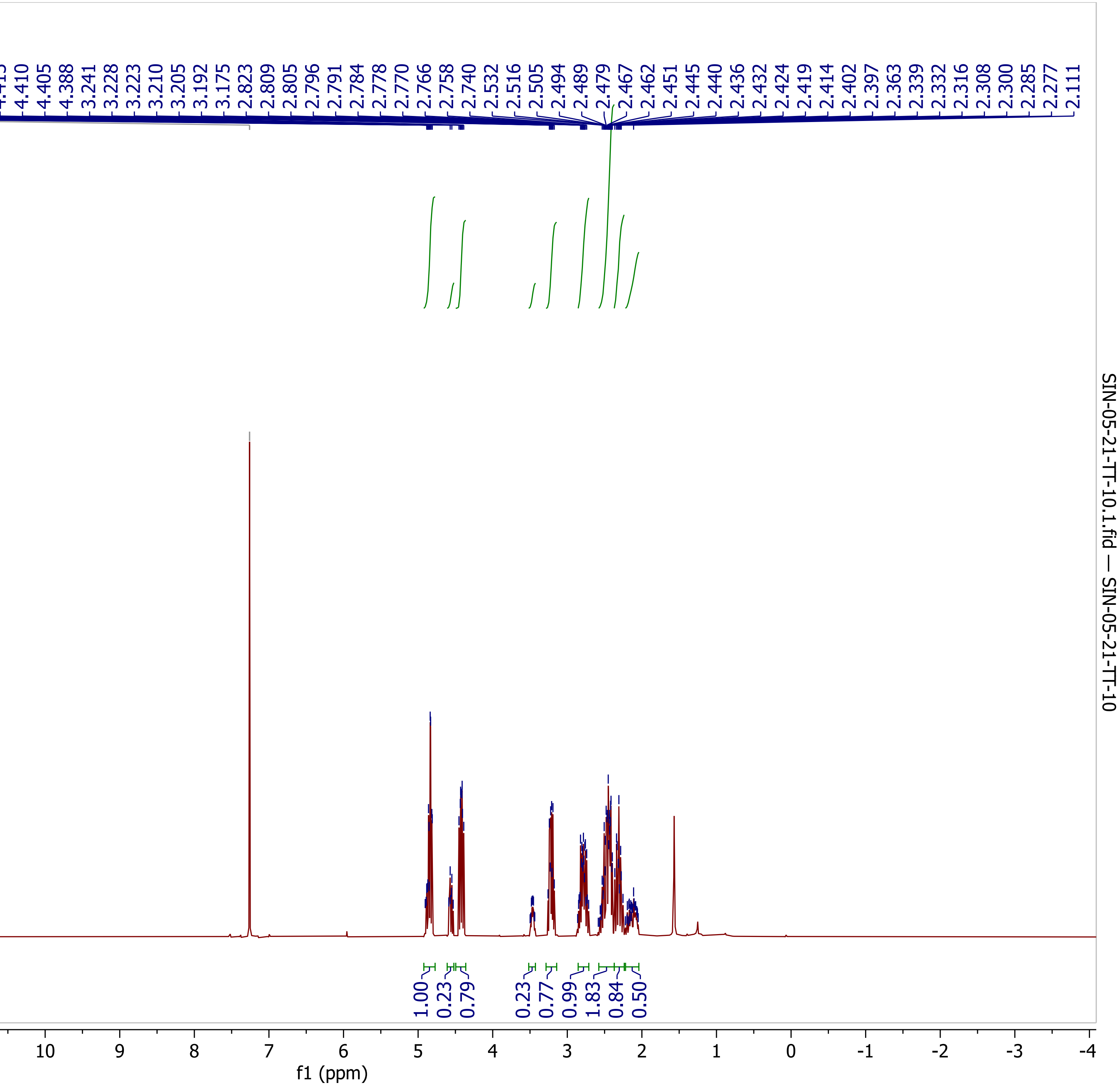

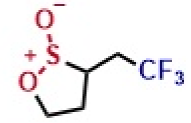

59

Parameter	Value
Solvent	CDCl3
Temperature	298.0
Acquisition Date	2024-01-20T00:08:18
Spectrometer Frequency	100.62
Nucleus	13C

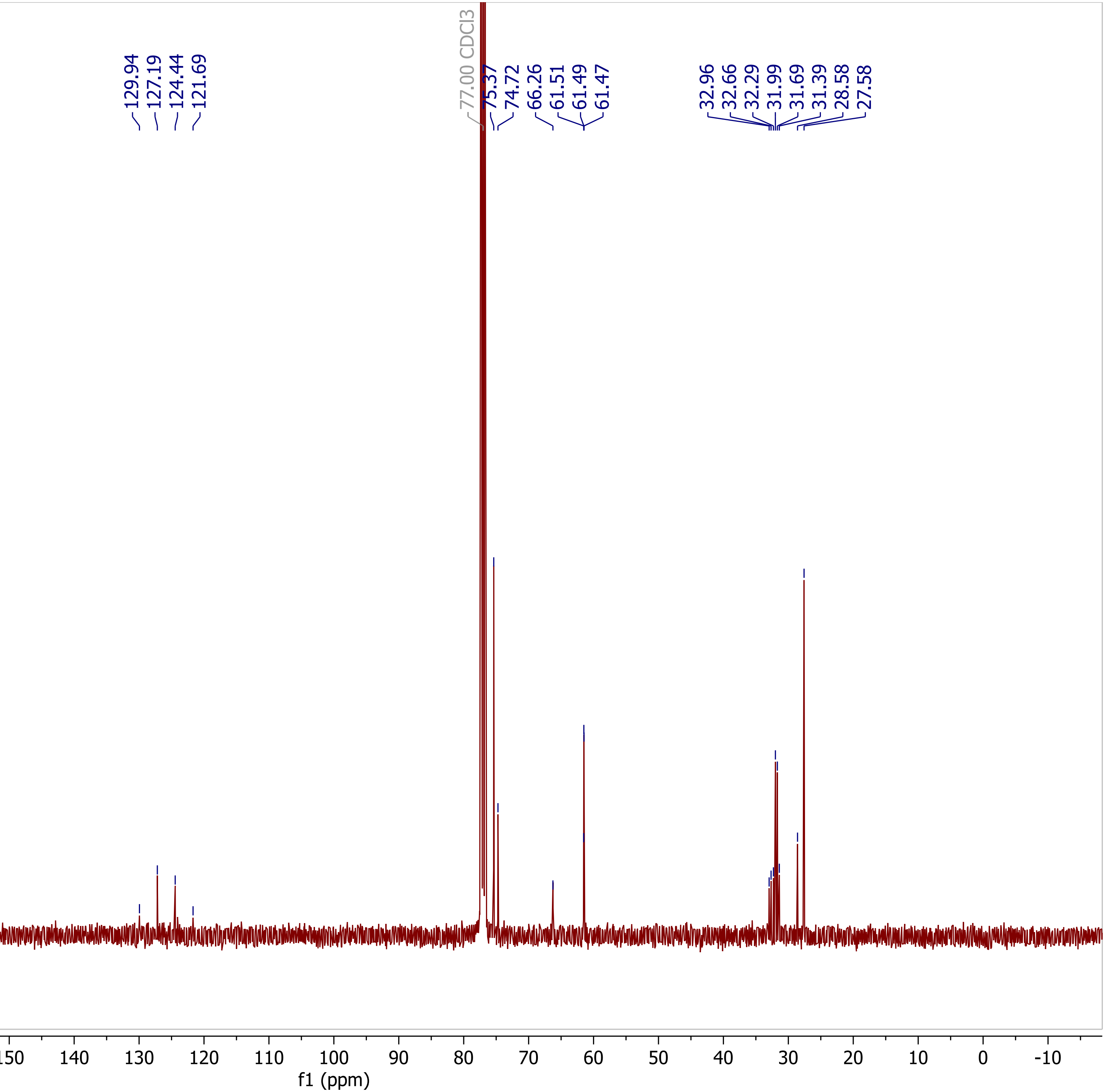

SIN-05-30-C13.1.fid — SIN-05-30-C13

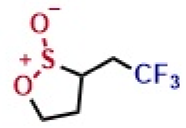

59

Parameter	Value
Solvent	CDCl3
Temperature	298.0
Acquisition Date	2024-01-20T00:12:30
Spectrometer Frequency	376.46
Nucleus	19F

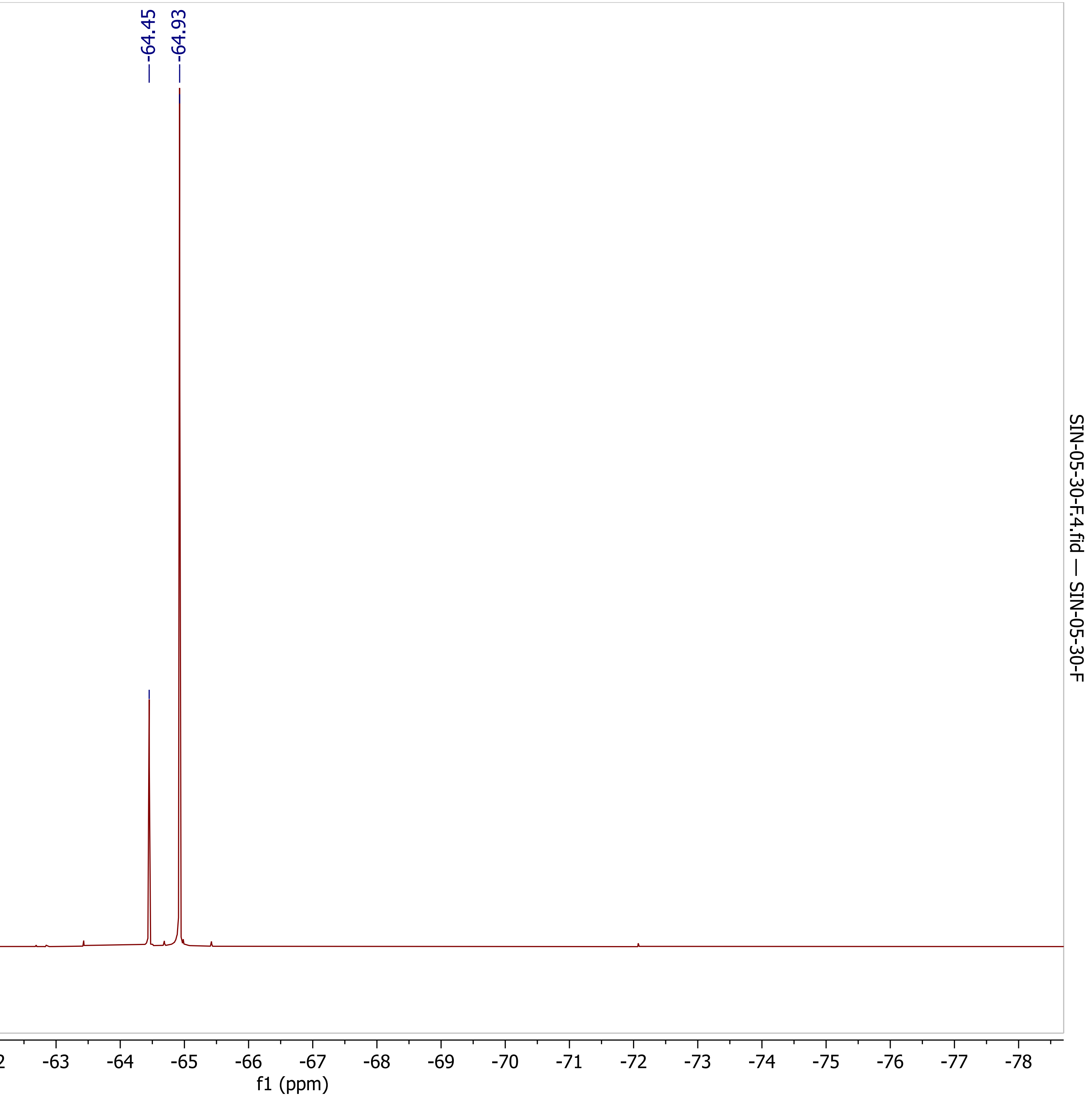

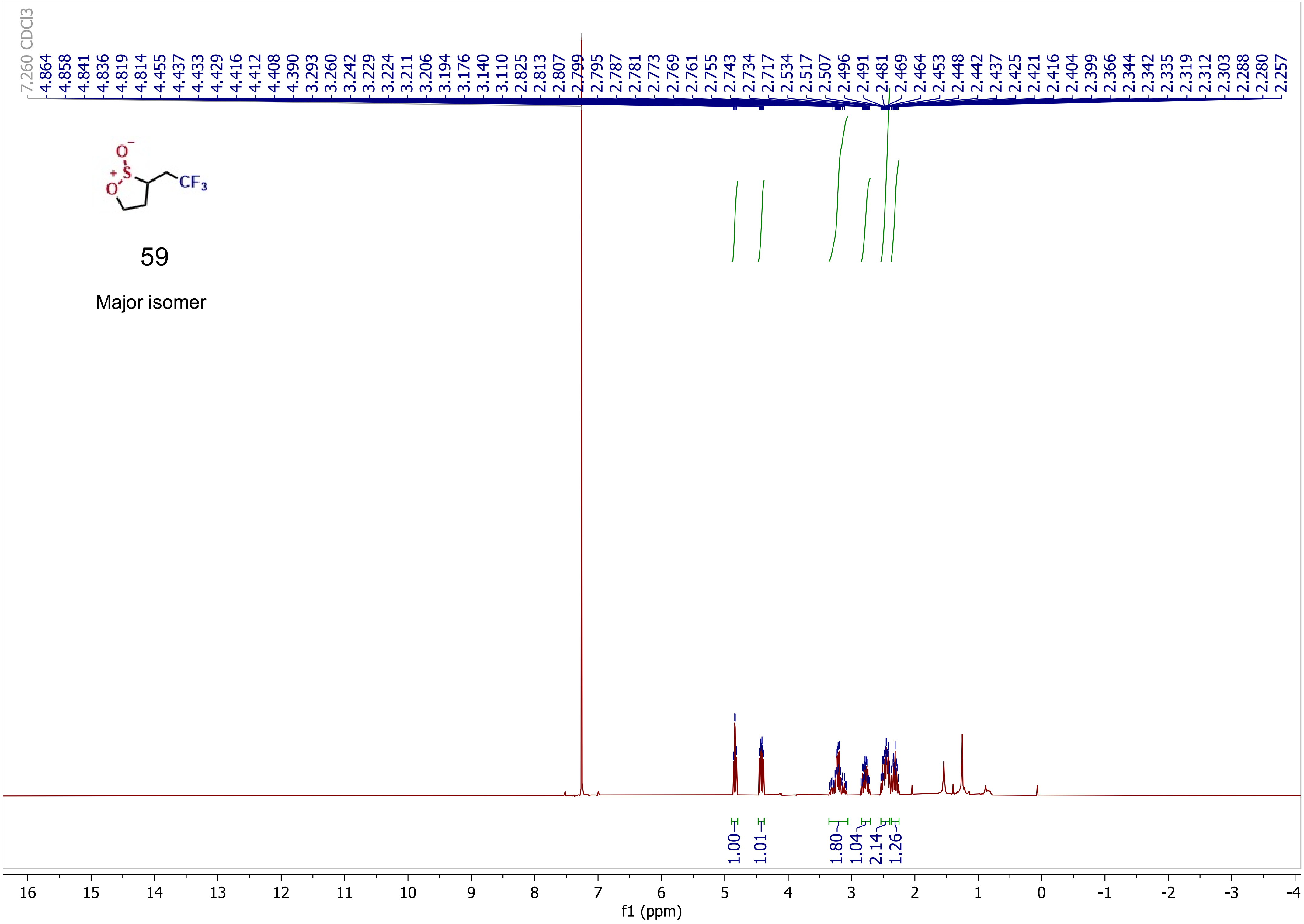

SIN-05-30-TT-30.1.fid — SIN-05-30-TT-30

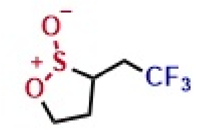

Major isomer (E)

59

Parameter	Value
Solvent	CDCl3
Temperature	298.0
Acquisition Date	2024-01-29T01:40:19
Spectrometer Frequency	100.62
Nucleus	13C

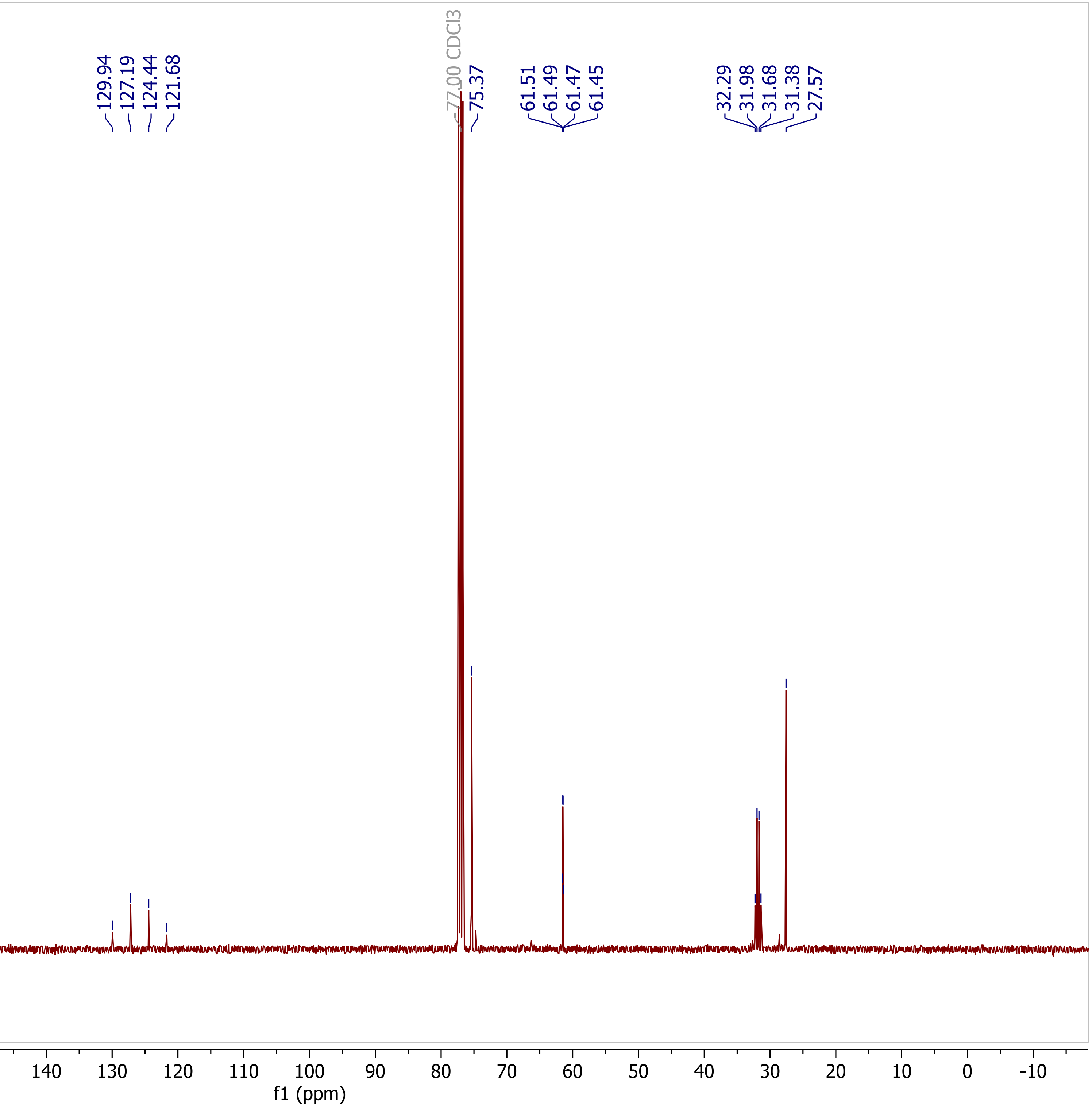

SIN-05-30-TT5-C13.3.fid — SIN-05-30-TT5-C13

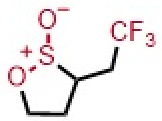

Major isomer (E)

59

Parameter	Value
Solvent	CDCl3
Temperature	298.0
Acquisition Date	2024-01-29T01:44:37
Spectrometer Frequency	376.46
Nucleus	19F

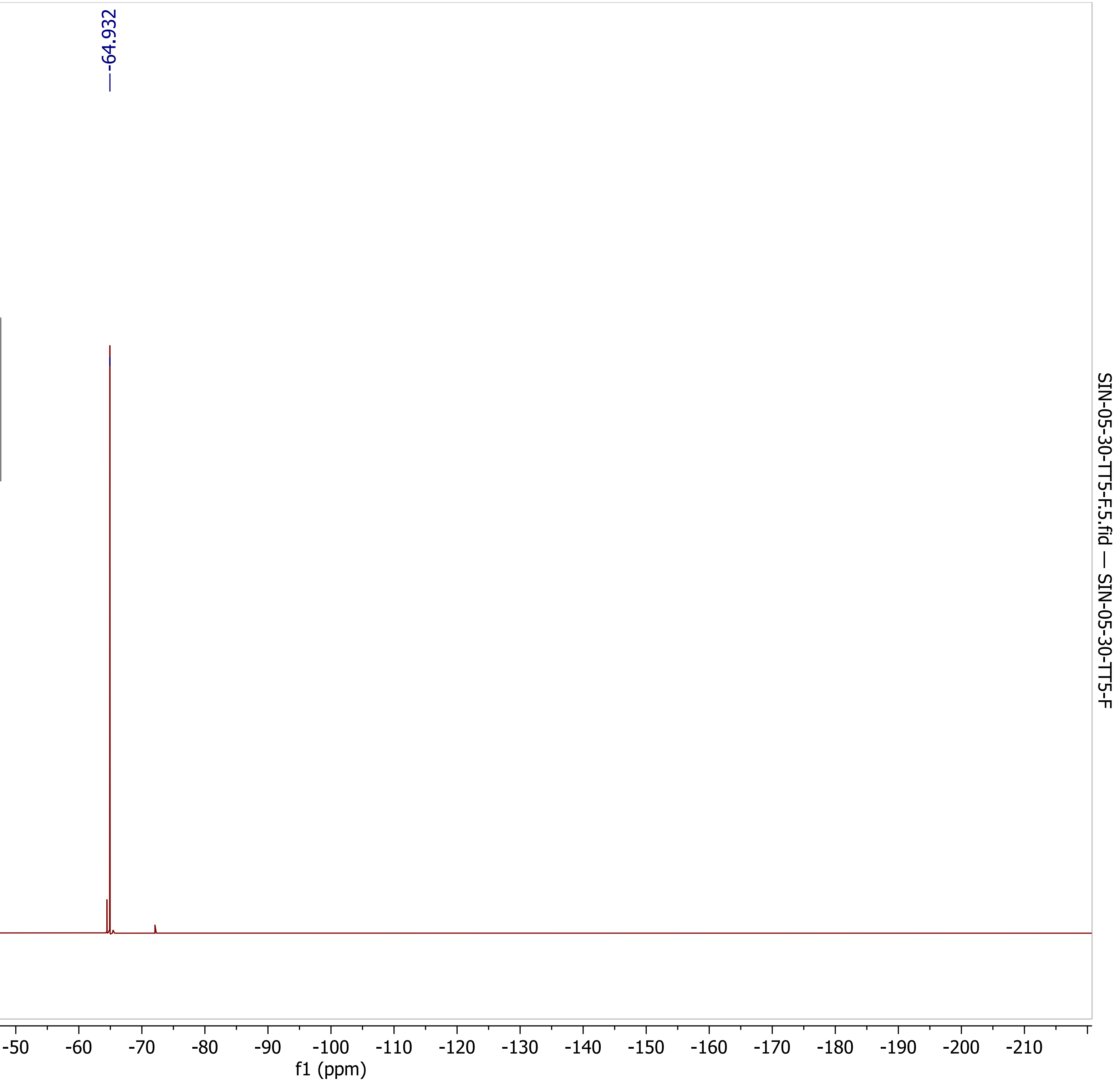

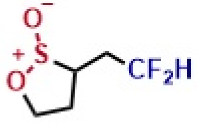

60

Parameter	Value
Solvent	CDCl3
Temperature	298.0
Acquisition Date	2024-02-06T19:22:06
Spectrometer Frequency	400.13
Nucleus	1H

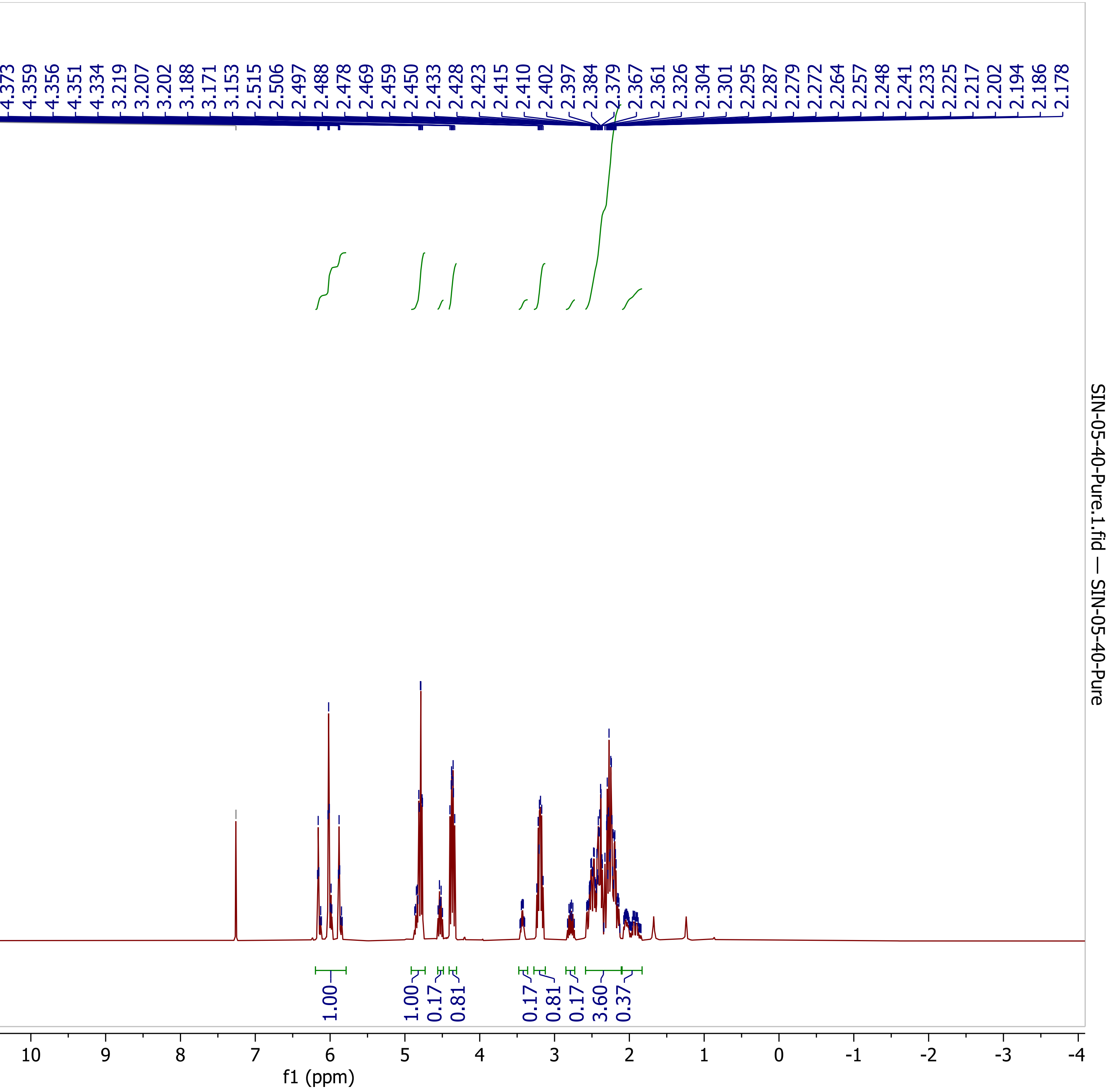

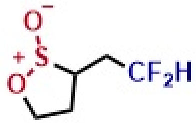

60

Parameter	Value
Solvent	CDCl3
Temperature	298.0
Acquisition Date	2024-02-07T00:04:03
Spectrometer Frequency	100.62
Nucleus	13C

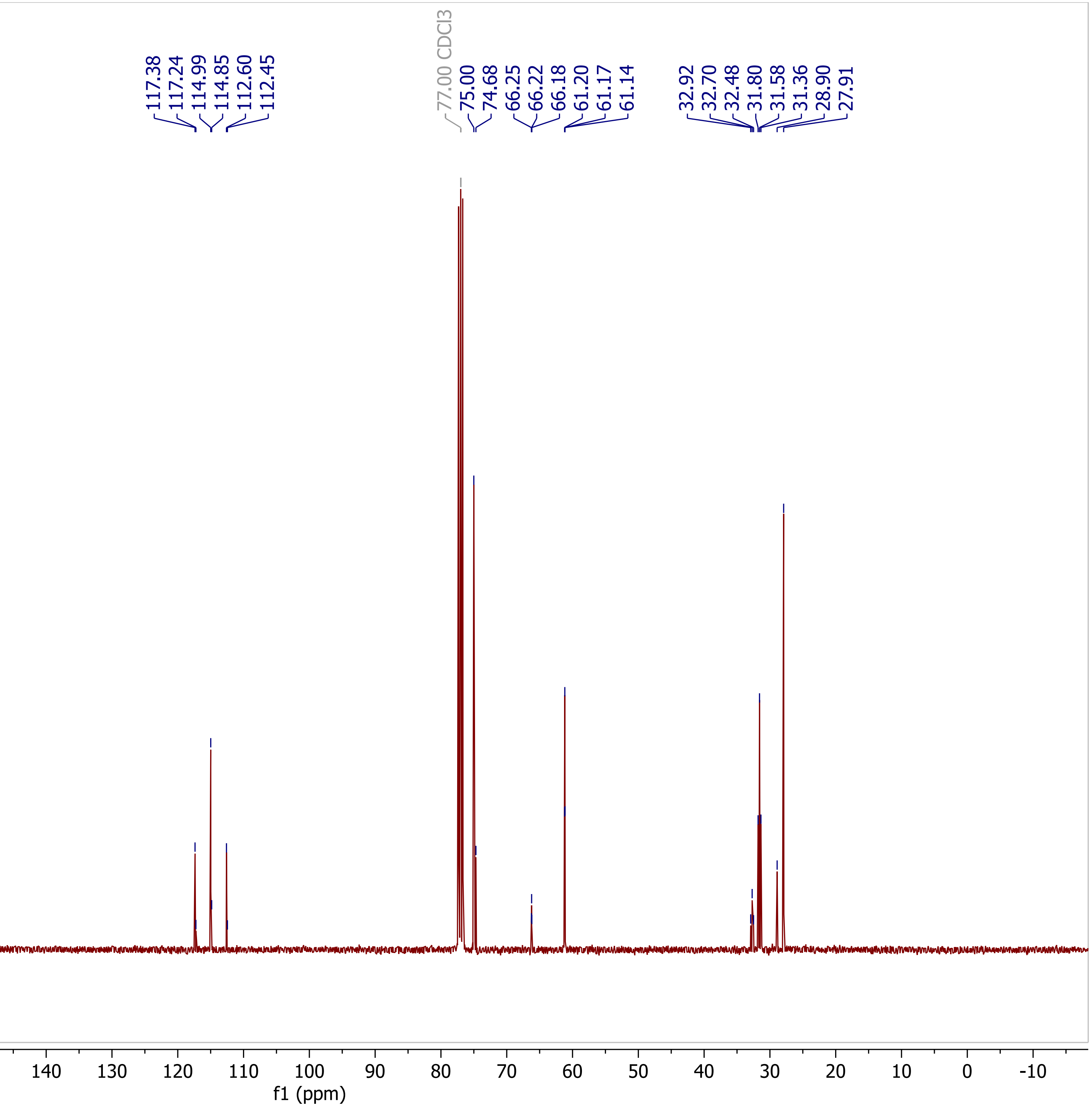

SIN-05-40-Pure-c13.3.fid — SIN-05-40-Pure

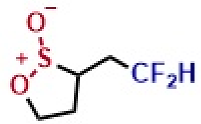

60

Parameter	Value
Solvent	CDCl3
Temperature	298.0
Acquisition Date	2024-02-07T00:08:17
Spectrometer Frequency	376.46
Nucleus	19F

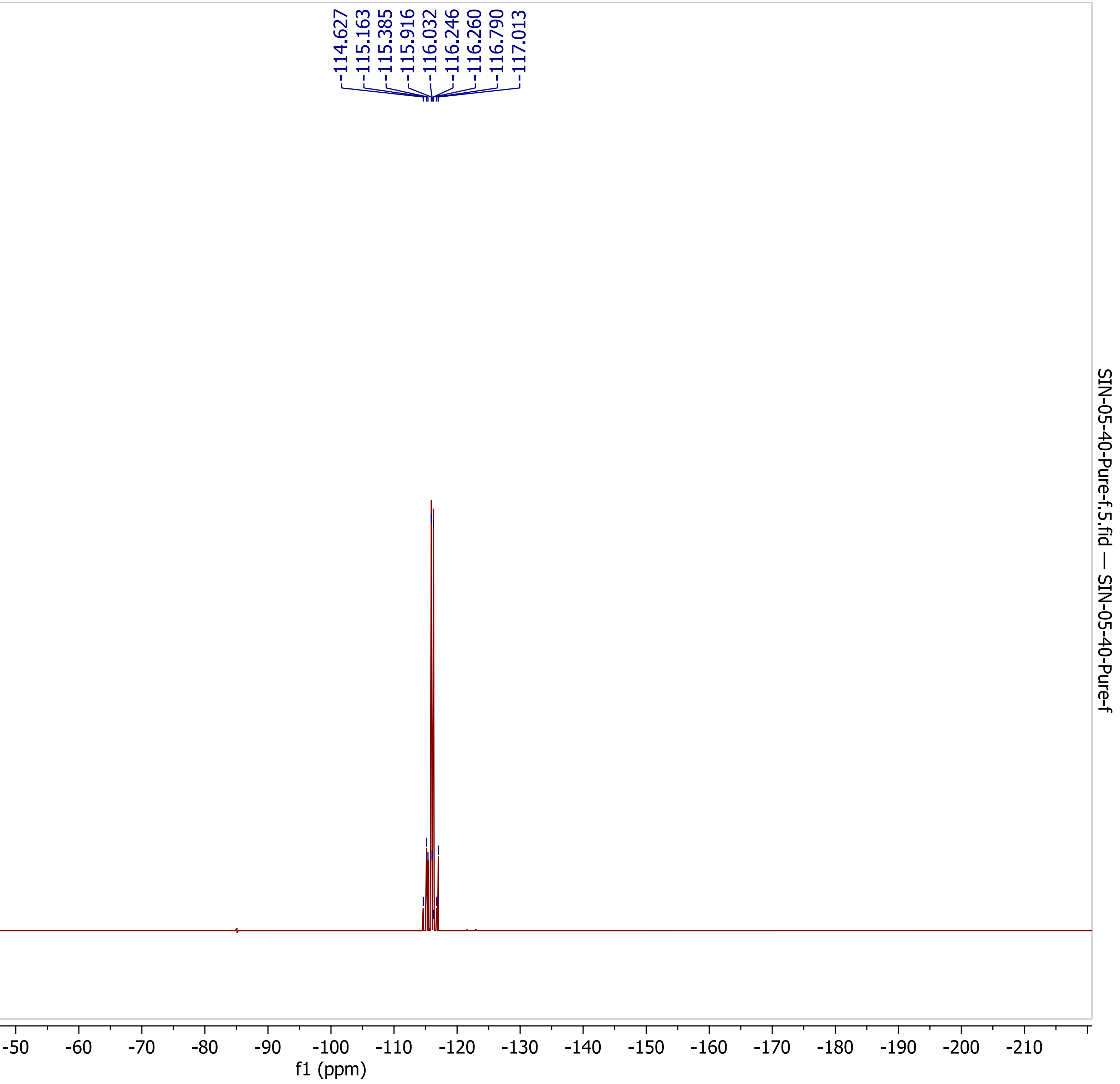

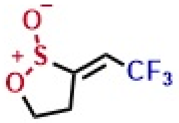

61

Parameter	Value
Solvent	CDCl3
Temperature	298.0
Acquisition Date	2024-07-17T10:14:27
Spectrometer Frequency	400.13
Nucleus	1H

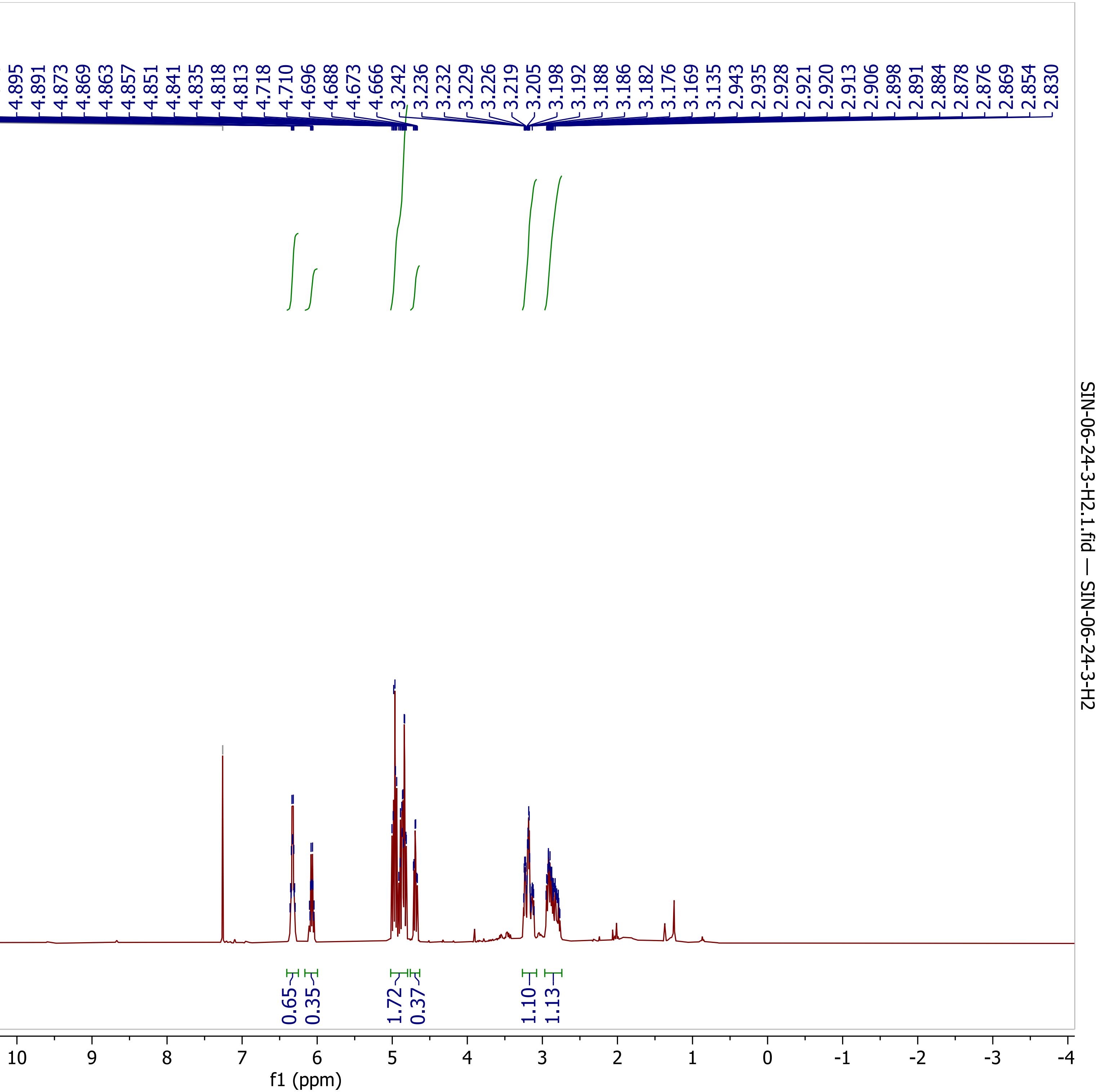

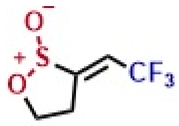

61

Parameter	Value
Solvent	CDCl3
Temperature	298.0
Acquisition Date	2024-07-17T00:03:16
Spectrometer Frequency	100.62
Nucleus	13C

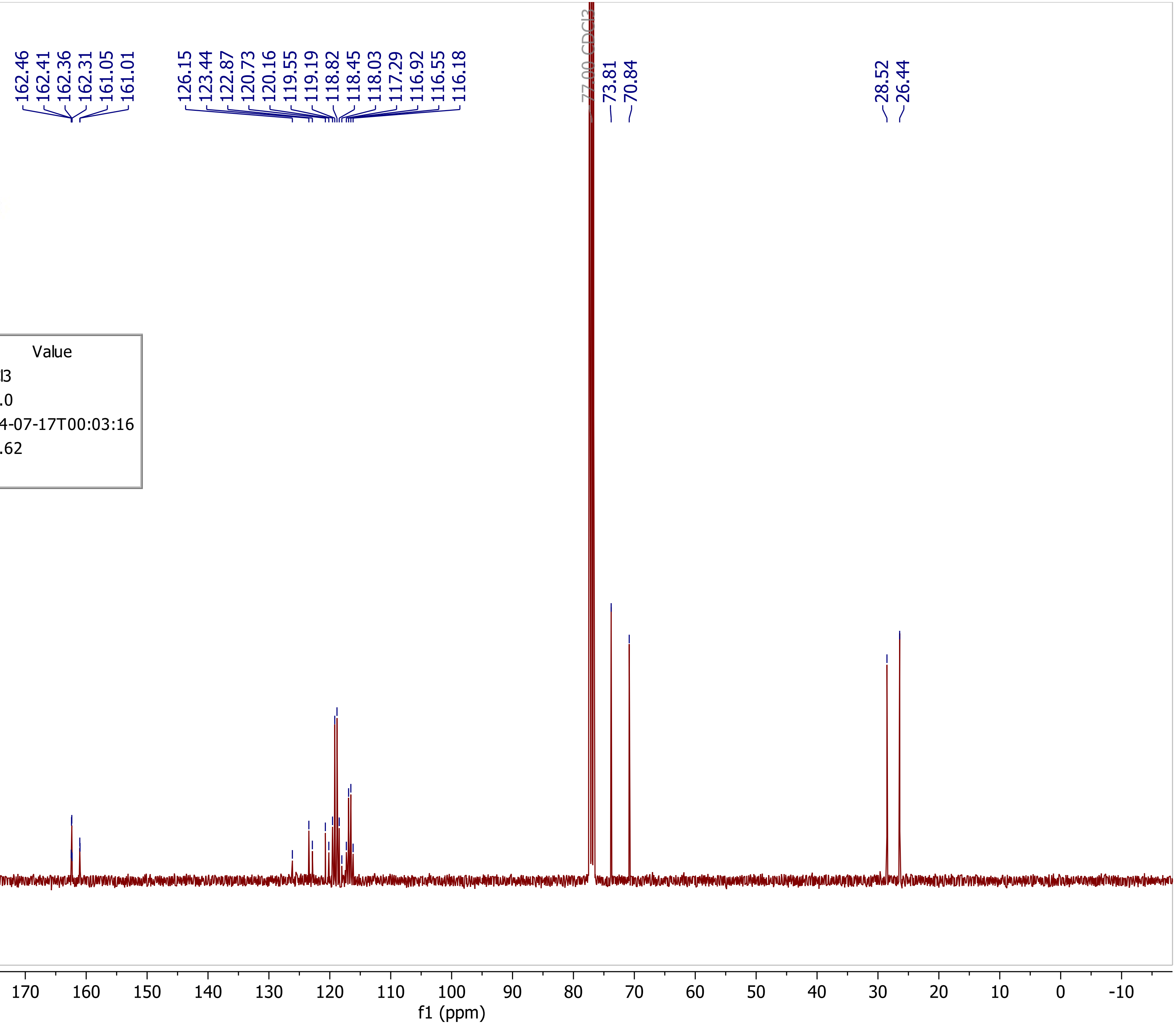

SIN-06-24-3-c13.3.fid — SIN-06-24-3-c13

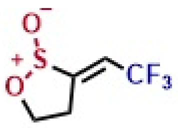

61

Parameter	Value
Solvent	CDCl3
Temperature	298.0
Acquisition Date	2024-03-17T17:30:05
Spectrometer Frequency	376.46
Nucleus	19F

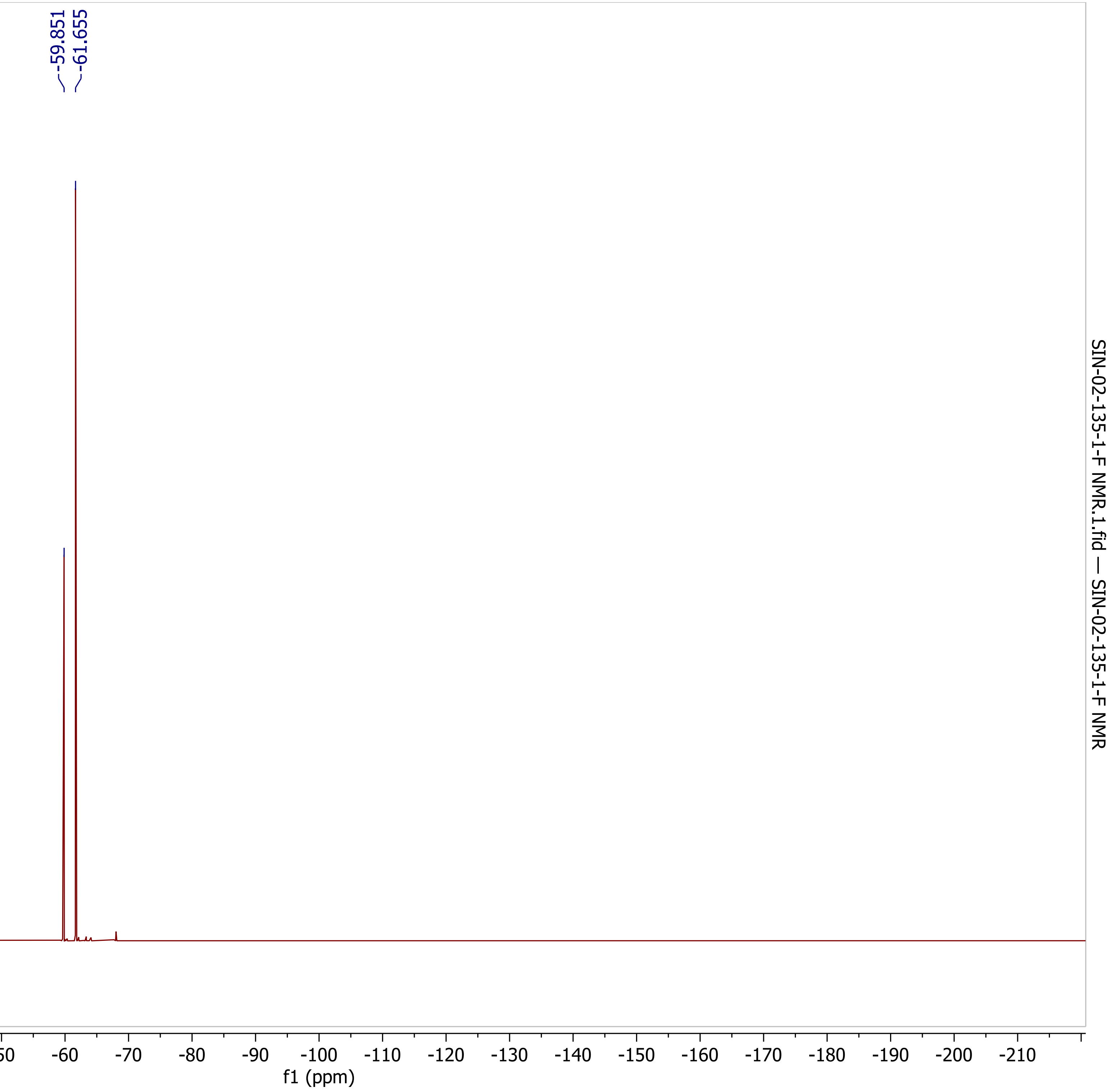

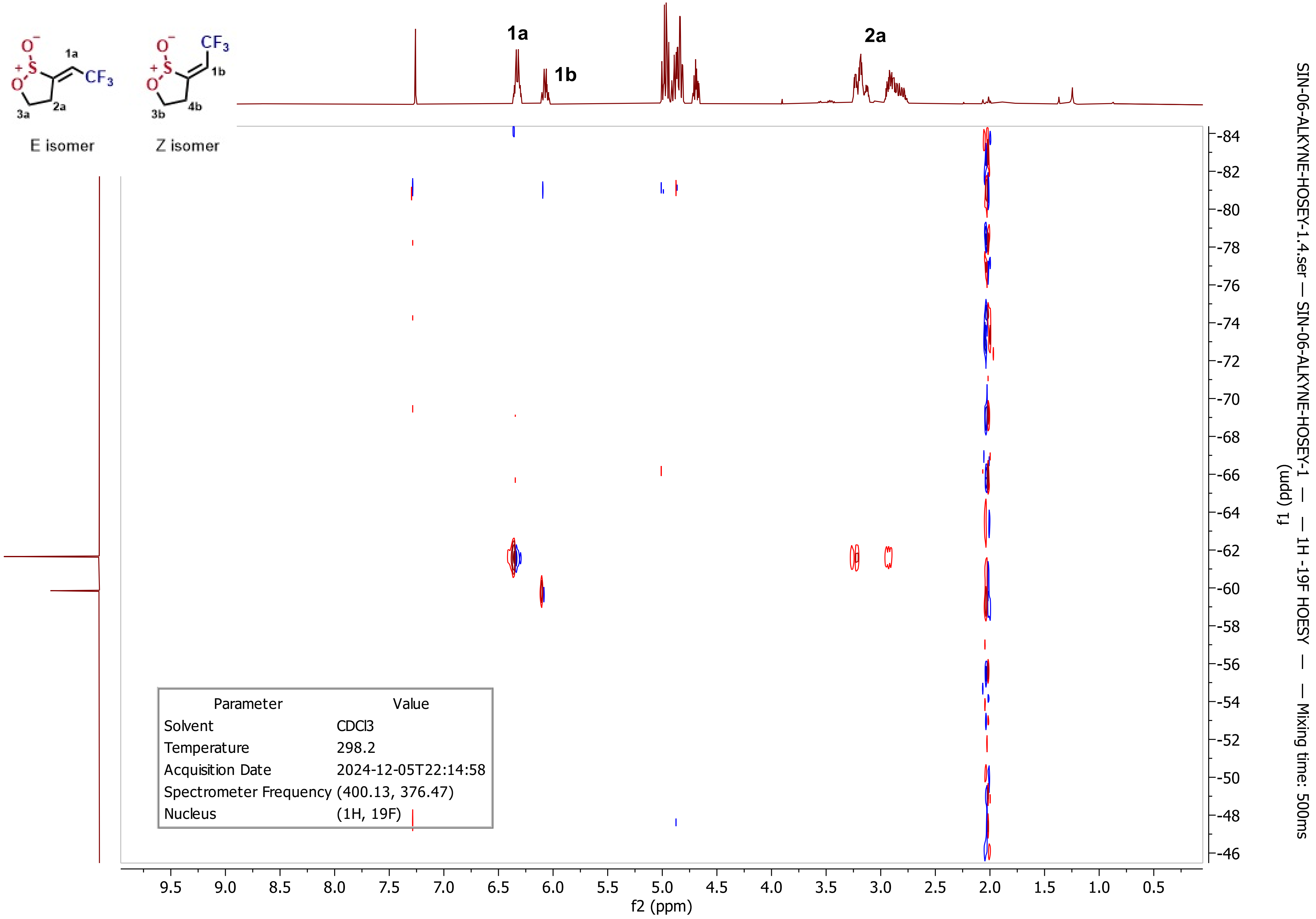

1a
1b
2a
3a
3b
4b
E isomer
Z isomer
Parameter Value
Solvent CDCl3
Temperature 298.2
Acquisition Date 2024-12-05T22:14:58
Spectrometer Frequency (400.13, 376.47)
Nucleus (1H, 19F)
f2 (ppm)
f1 (ppm)
SIN-06-ALKYNE-HOSEY-1.4.ser — SIN-06-ALKYNE-HOSEY-1 — — 1H -19F HOESY — — Mixing time: 500ms

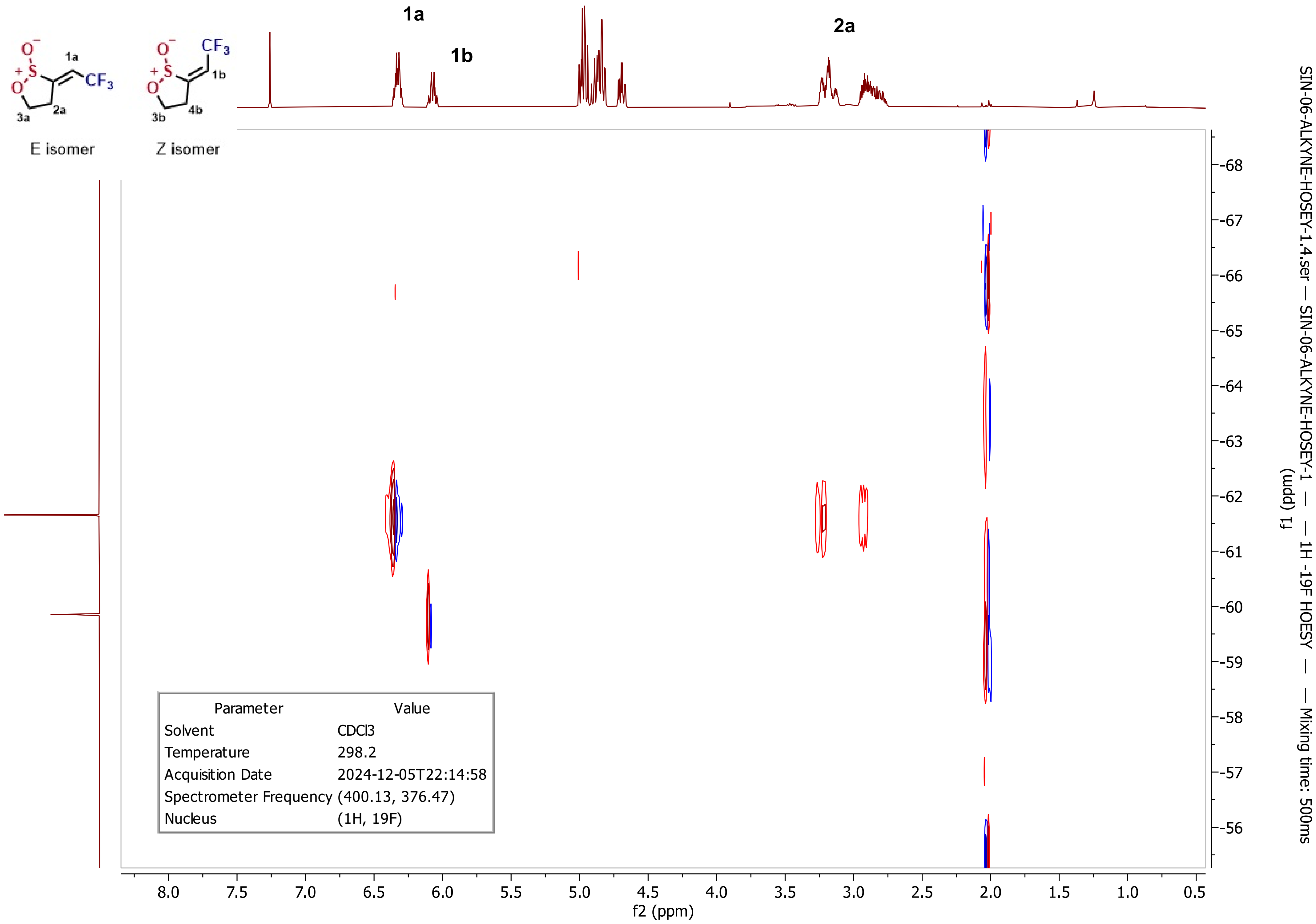

Parameter	Value
Solvent	CDCl3
Temperature	298.2
Acquisition Date	2024-12-05T22:14:58
Spectrometer Frequency	(400.13, 376.47)
Nucleus	(1H, 19F)

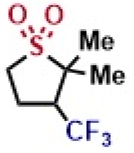

65

Parameter	Value
Solvent	CDCl3
Temperature	298.0
Acquisition Date	2024-08-24T09:47:34
Spectrometer Frequency	400.13
Nucleus	1H

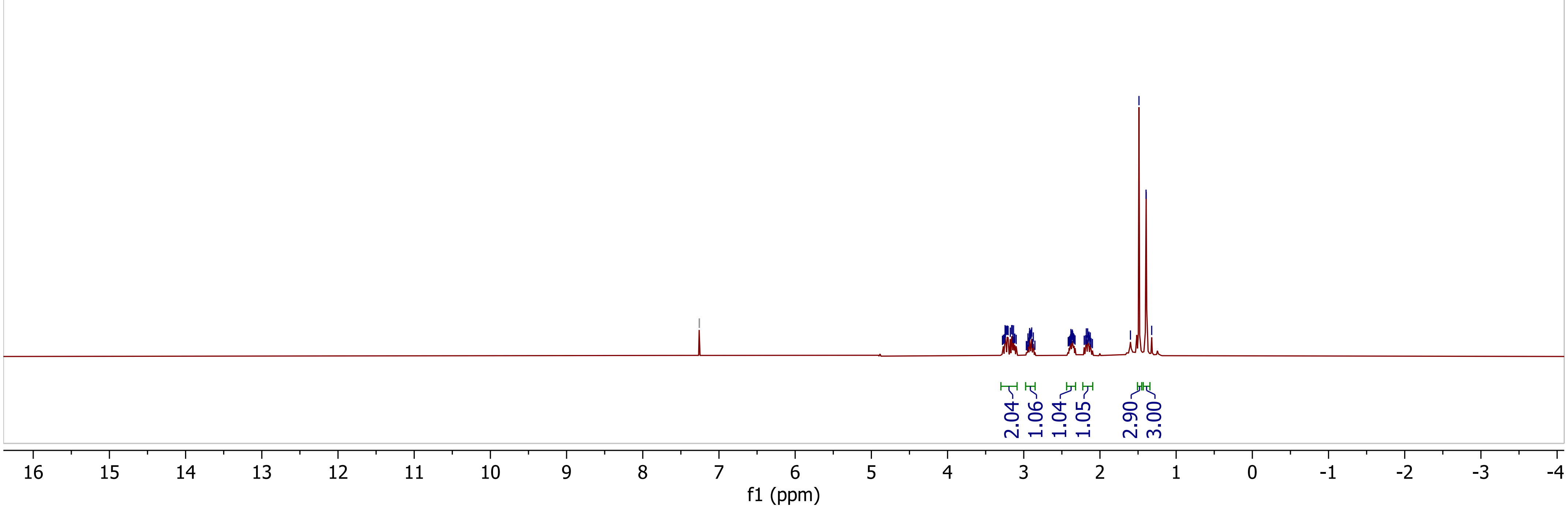

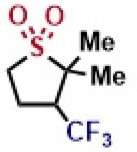

65

Parameter	Value
Solvent	CDCl3
Temperature	298.0
Acquisition Date	2024-08-20T22:39:38
Spectrometer Frequency	100.62
Nucleus	13C

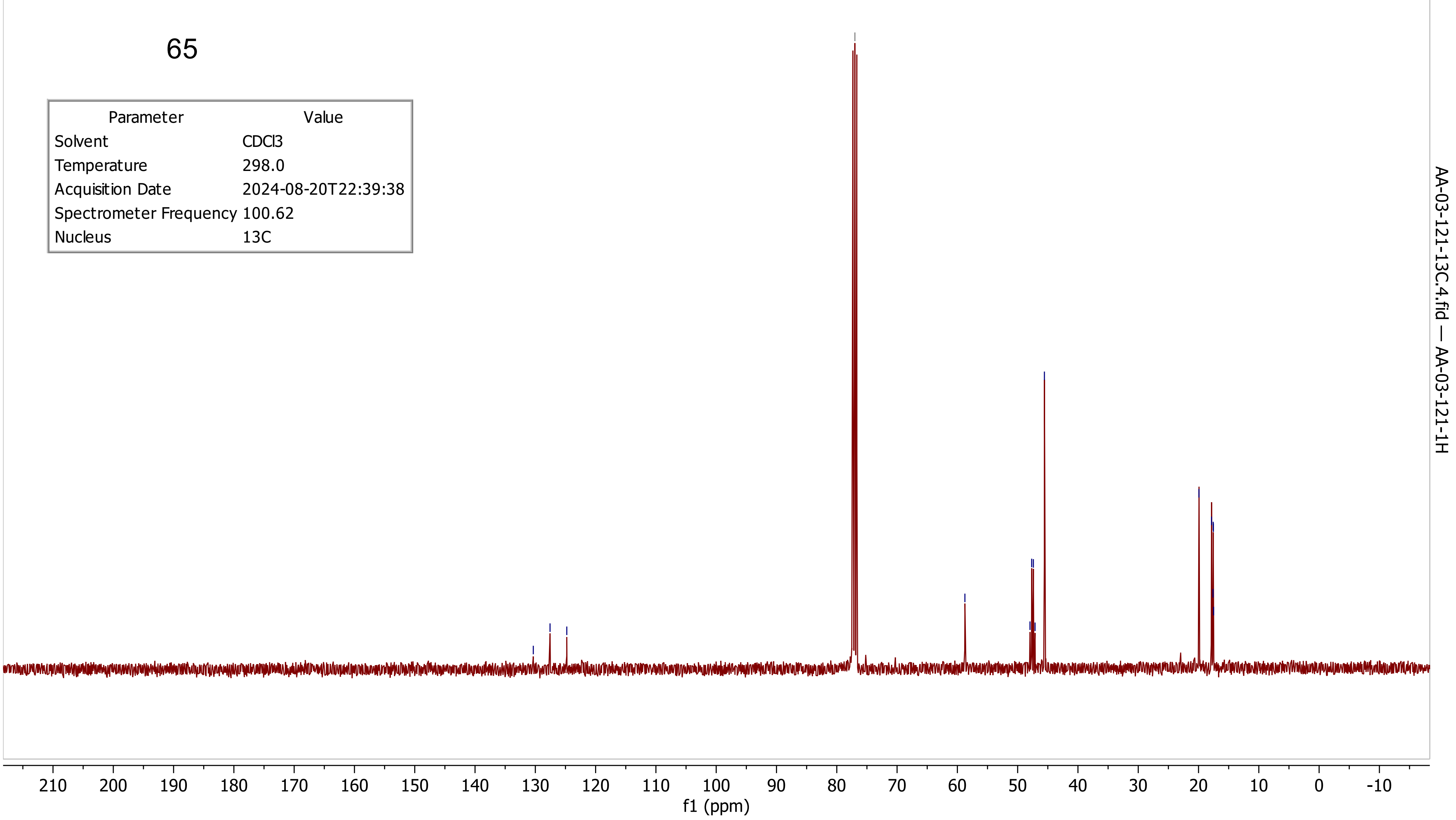

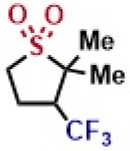

65

Parameter	Value
Solvent	CDCl3
Temperature	298.0
Acquisition Date	2024-08-20T22:44:26
Spectrometer Frequency	376.46
Nucleus	19F

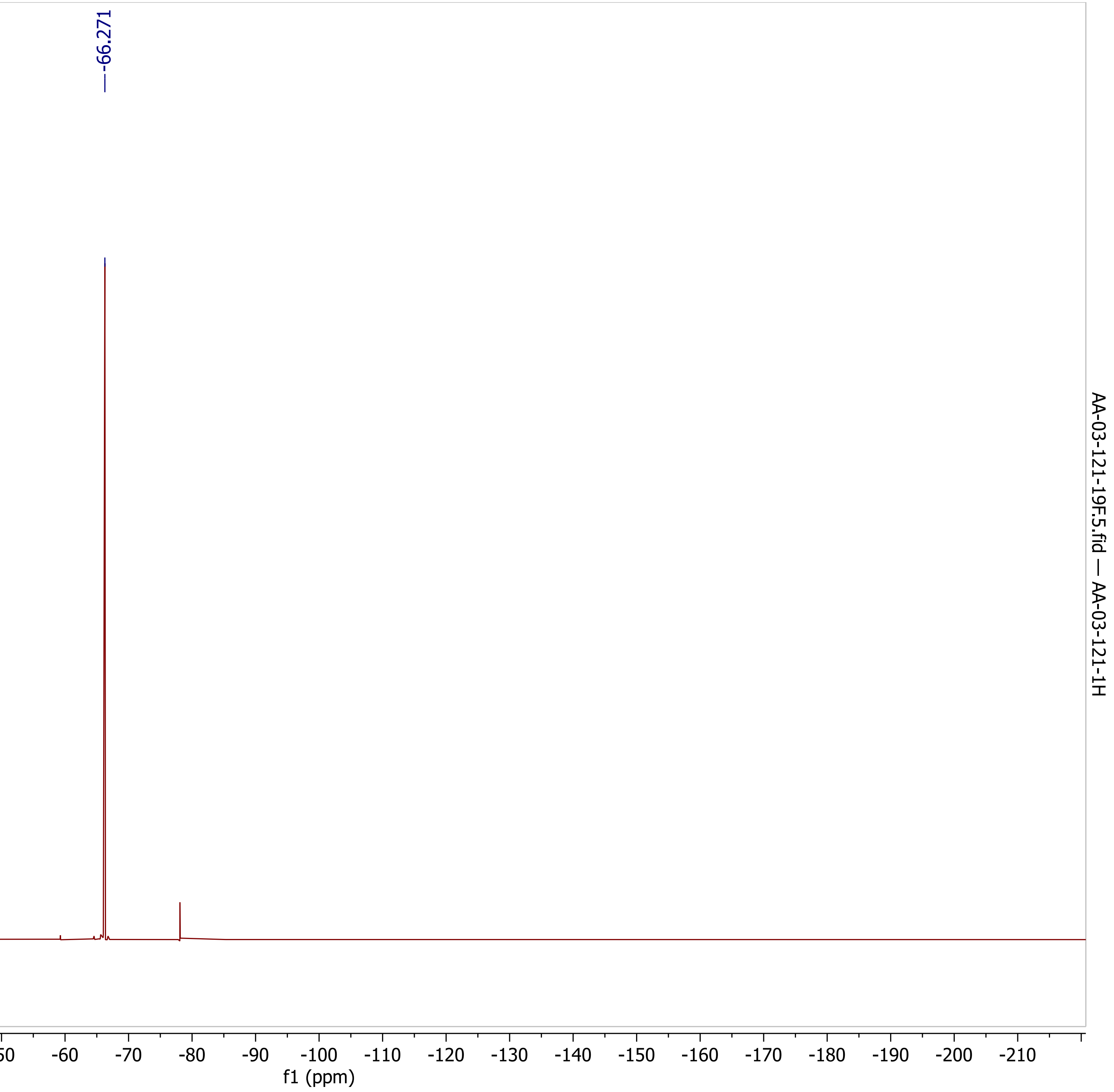

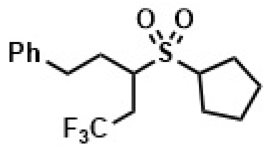

66

Parameter	Value
Solvent	CDCl3
Temperature	298.0
Acquisition Date	2024-10-08T14:35:53
Spectrometer Frequency	400.13
Nucleus	1H

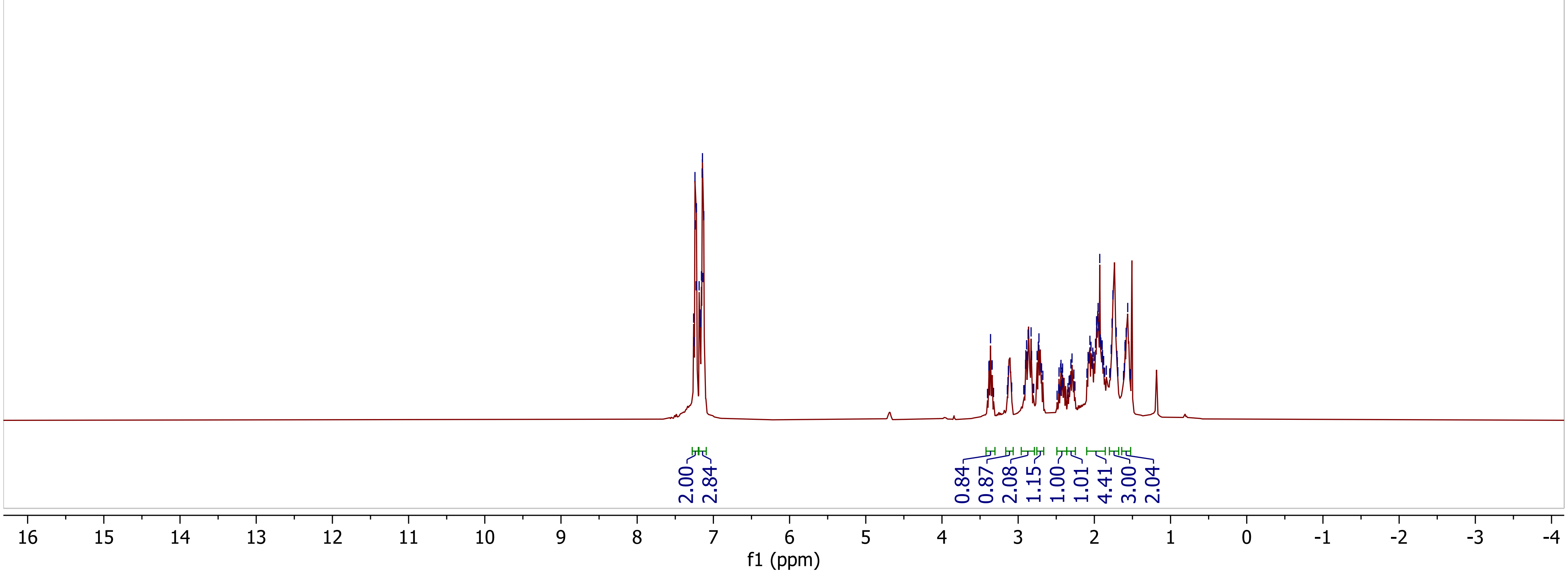

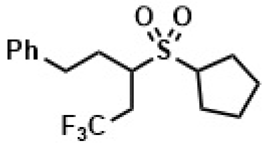

66

Parameter	Value
Solvent	CDCl3
Temperature	298.0
Acquisition Date	2024-10-09T05:14:23
Spectrometer Frequency	100.62
Nucleus	13C

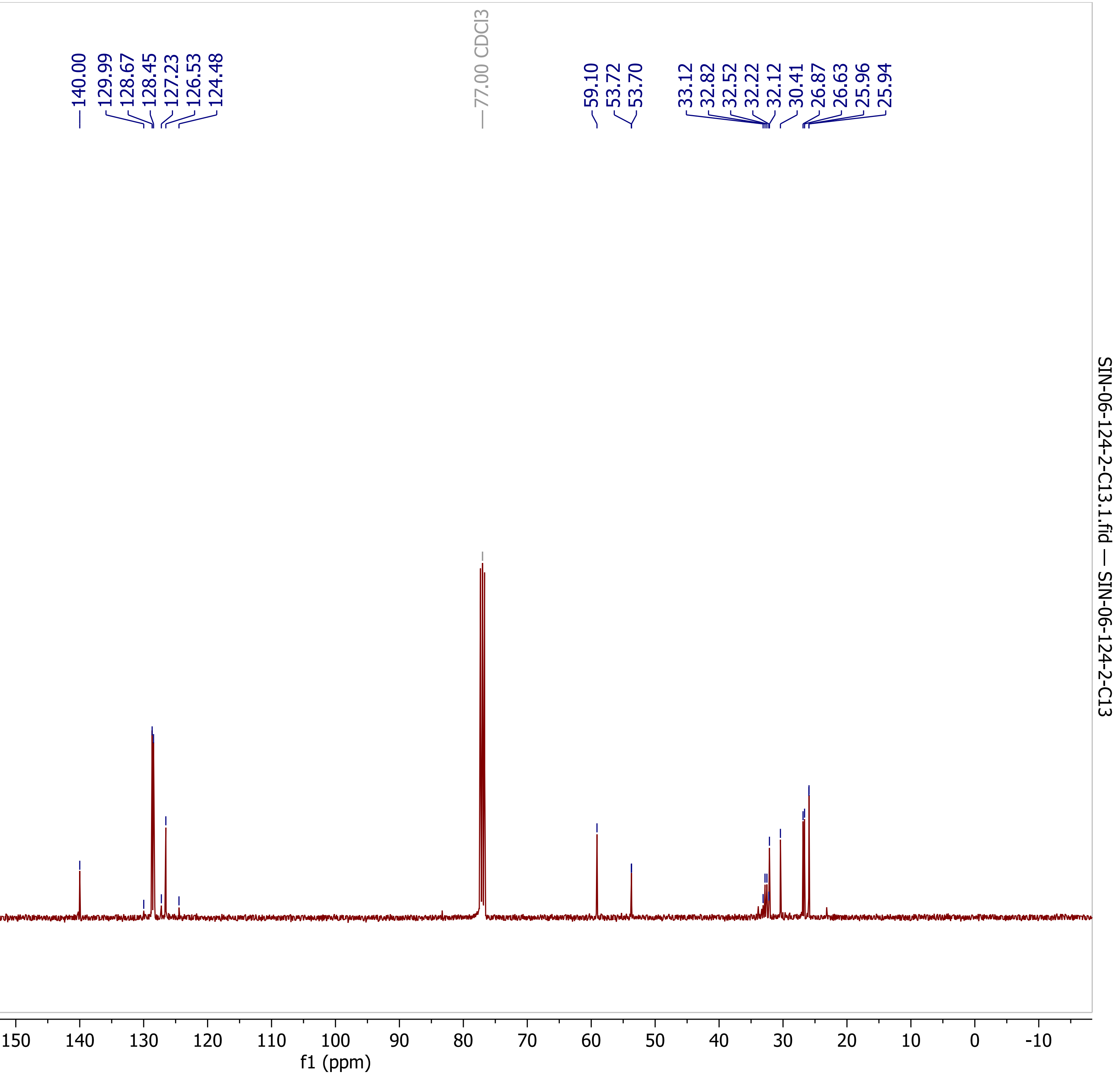

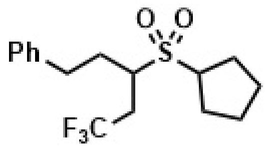

66

Parameter	Value
Solvent	CDCl3
Temperature	298.0
Acquisition Date	2024-10-07T19:16:32
Spectrometer Frequency	376.46
Nucleus	19F

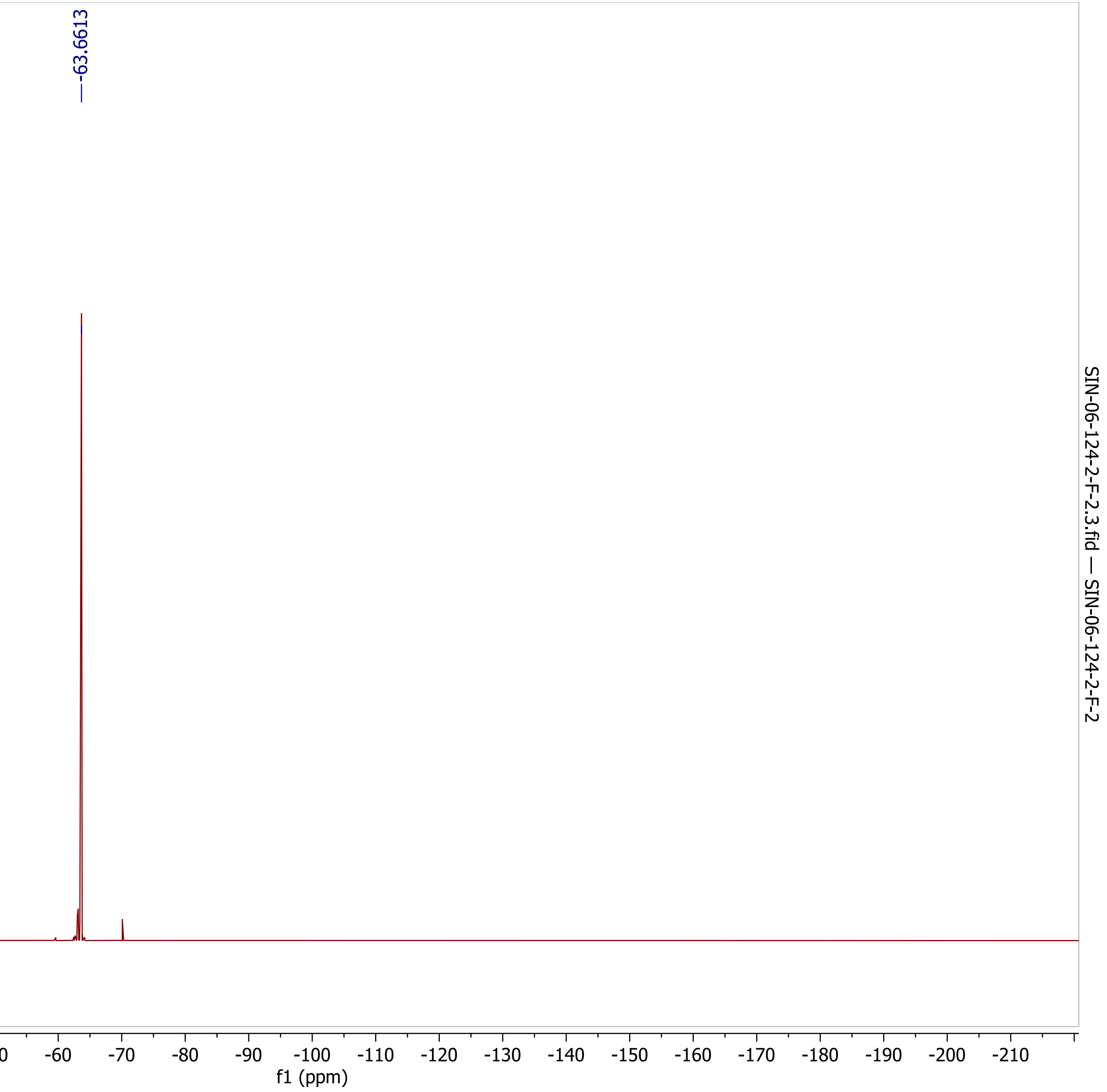

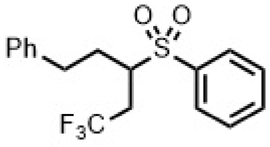

67

Parameter	Value
Solvent	CDCl3
Temperature	298.0
Acquisition Date	2024-10-26T18:23:25
Spectrometer Frequency	400.13
Nucleus	1H

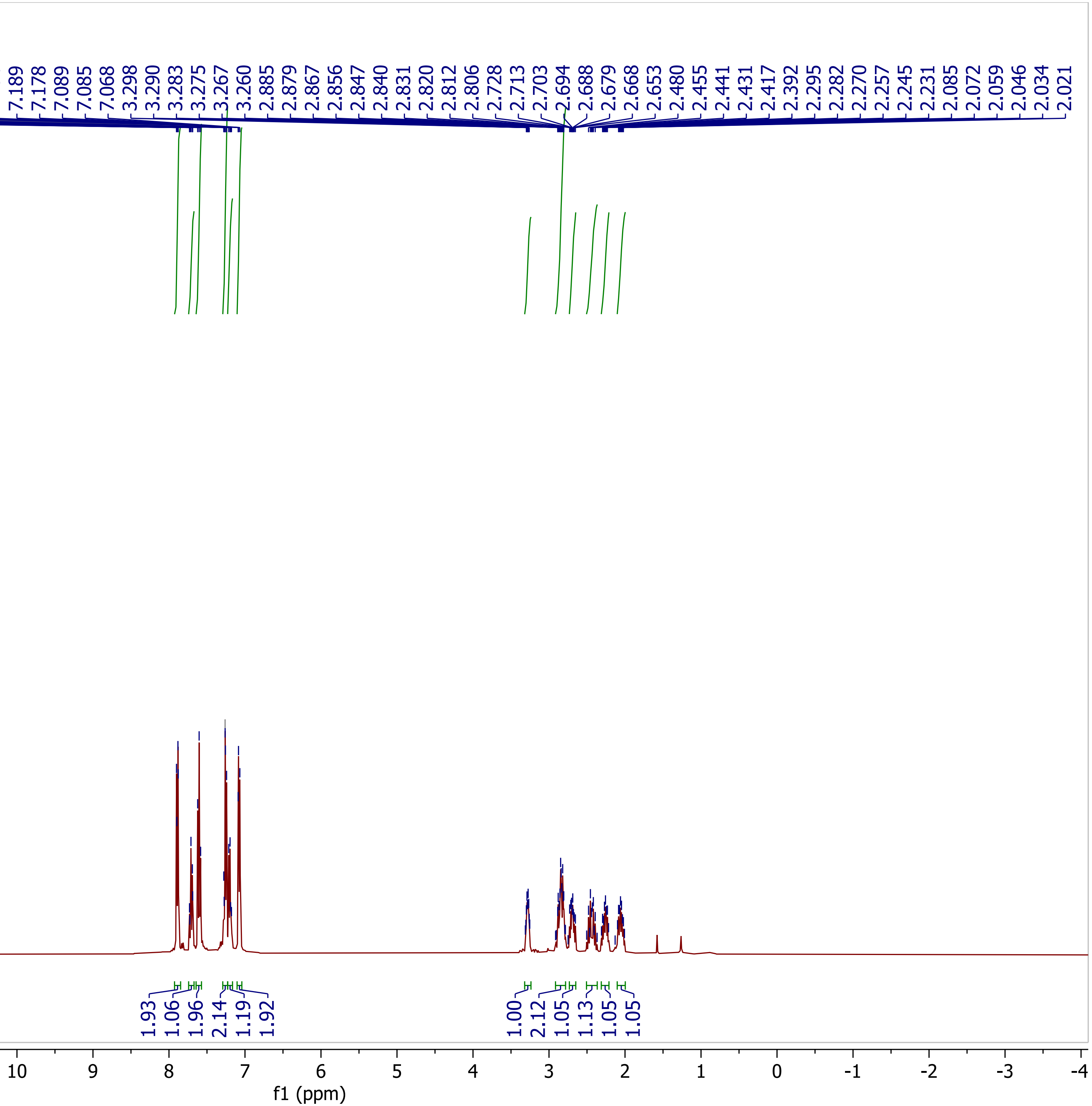

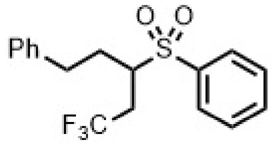

67

Parameter	Value
Solvent	CDCl3
Temperature	298.0
Acquisition Date	2024-10-27T01:11:25
Spectrometer Frequency	100.62
Nucleus	13C

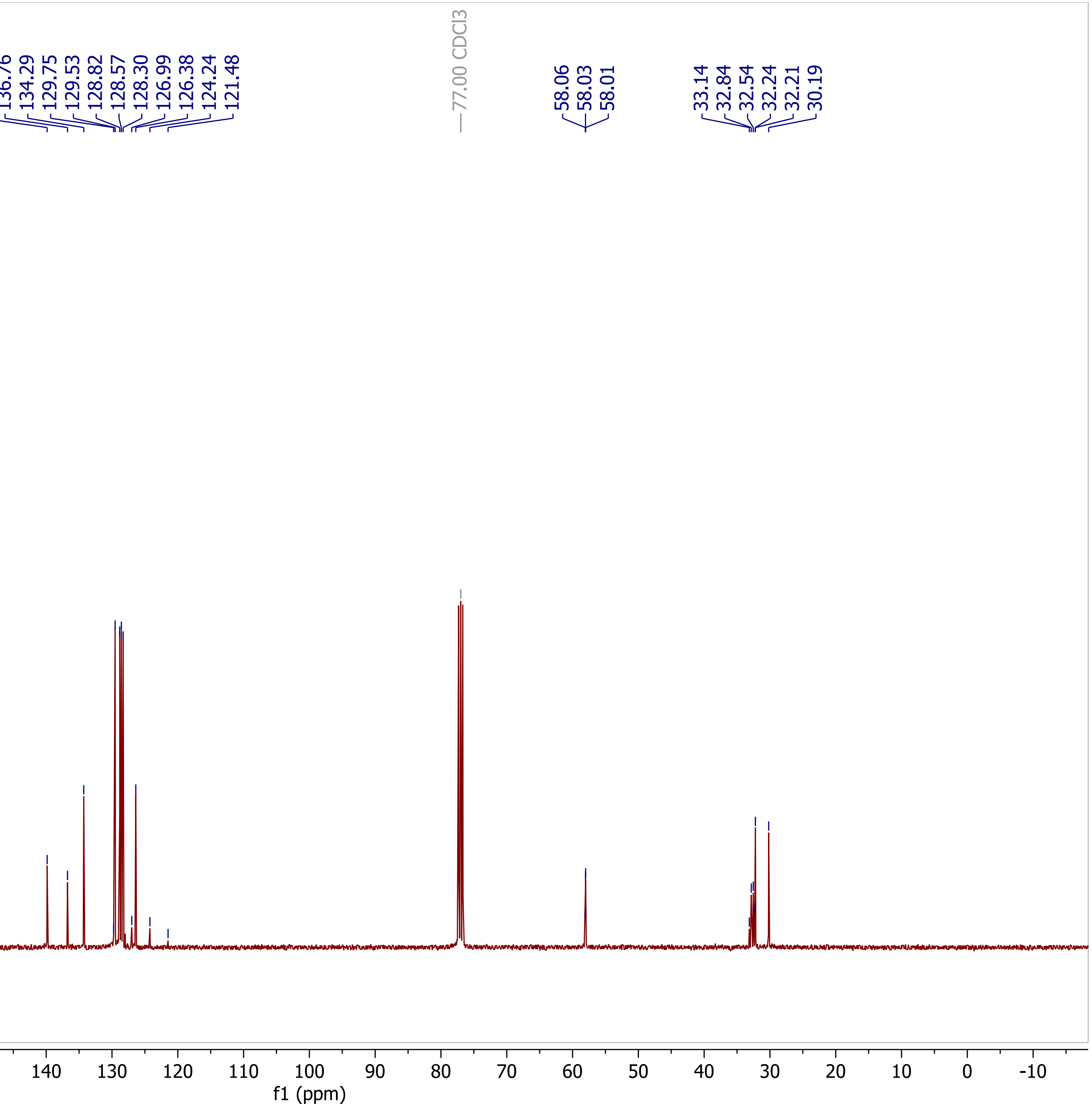

SIN-06-155-PH-c13.5.fid — SIN-06-155-PH-c13

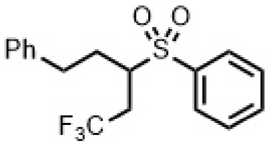

67

Parameter	Value
Solvent	CDCl3
Temperature	298.0
Acquisition Date	2024-10-26T19:29:18
Spectrometer Frequency	376.46
Nucleus	19F

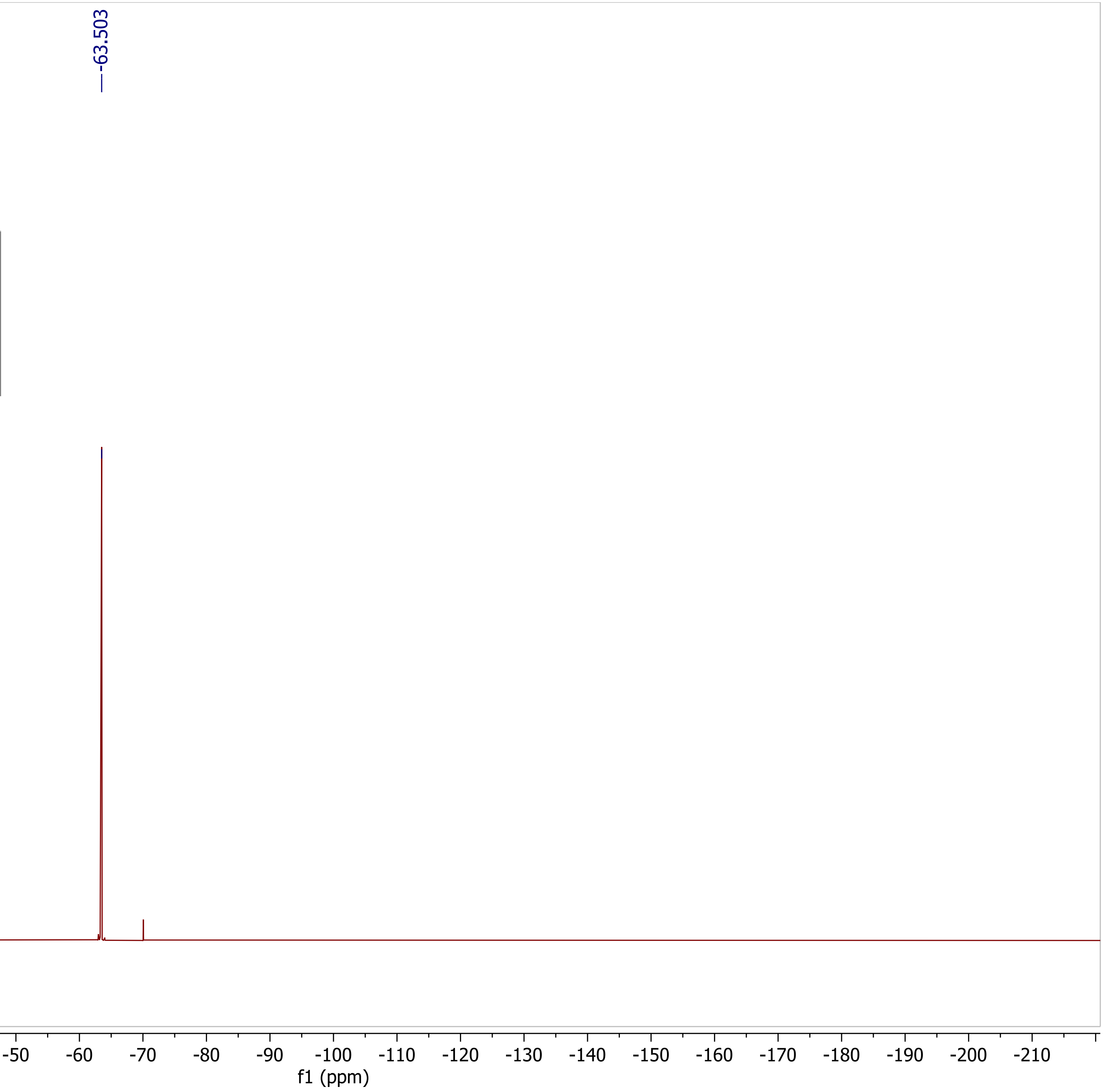

SIN-06-155-2-PH-F.3.fid — SIN-06-155-2-PH-F

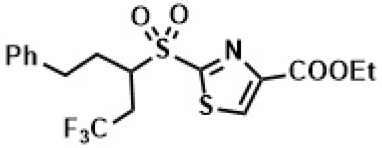

68

Parameter	Value
Solvent	CDCl3
Temperature	298.0
Acquisition Date	2024-10-07T16:03:28
Spectrometer Frequency	400.13
Nucleus	1H

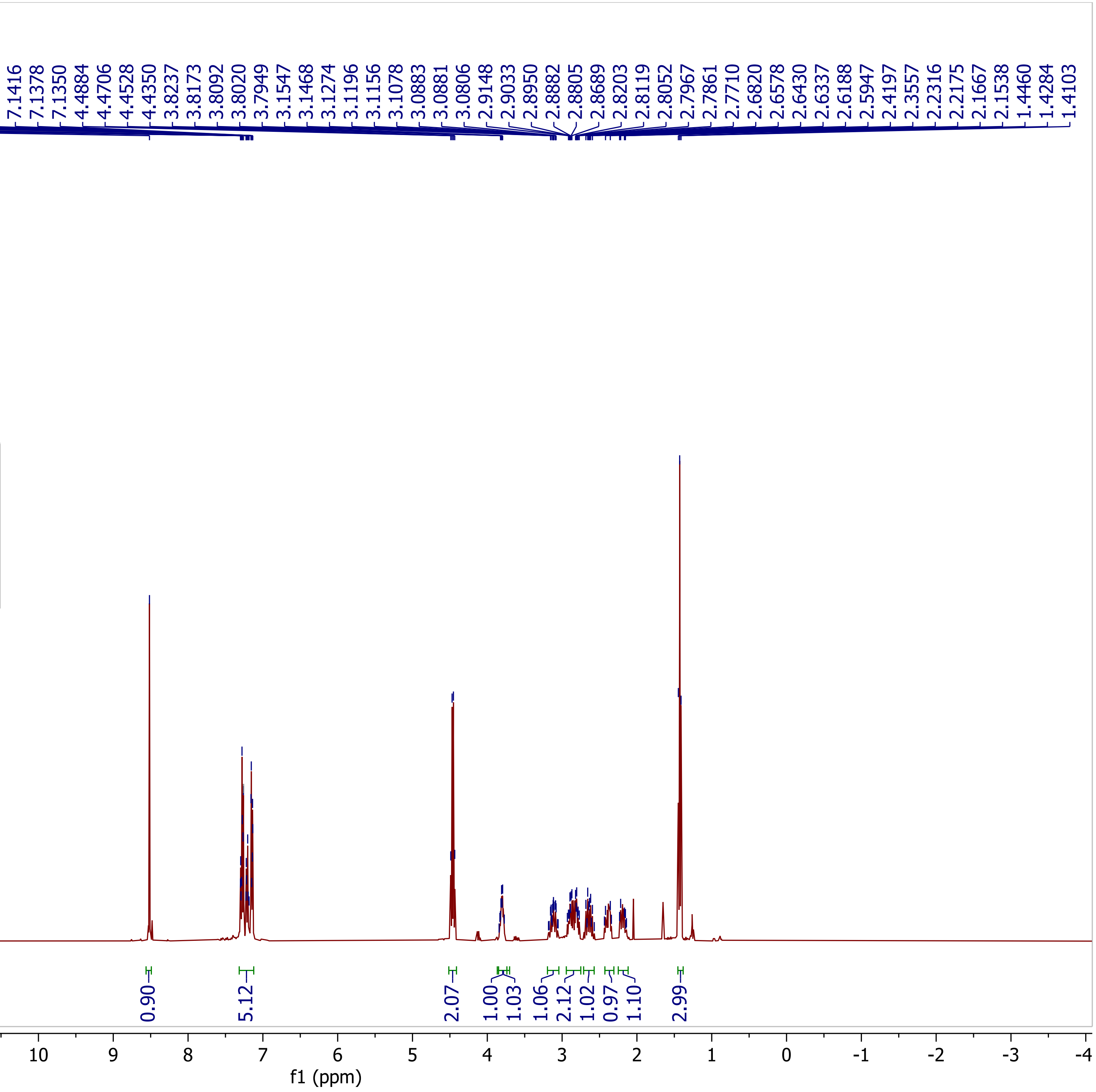

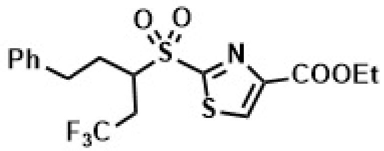

68

Parameter	Value
Solvent	CDCl3
Temperature	298.0
Acquisition Date	2024-10-08T01:41:48
Spectrometer Frequency	100.62
Nucleus	13C

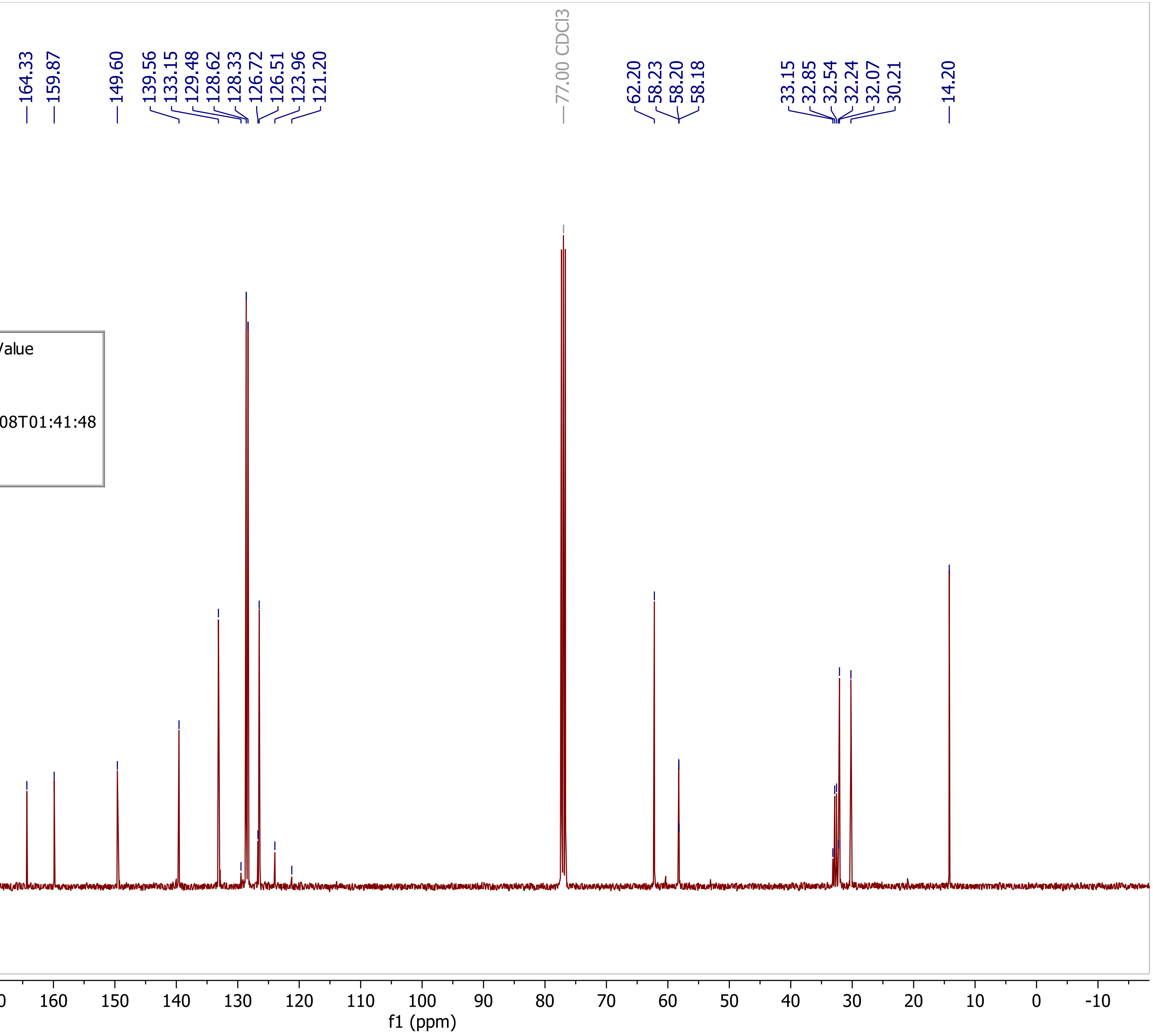

SIN-06-134-4-C13.1.fid — SIN-06-134-4-C13

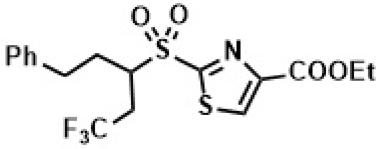

68

Parameter	Value
Solvent	CDCl3
Temperature	298.0
Acquisition Date	2024-10-08T01:46:27
Spectrometer Frequency	376.46
Nucleus	19F

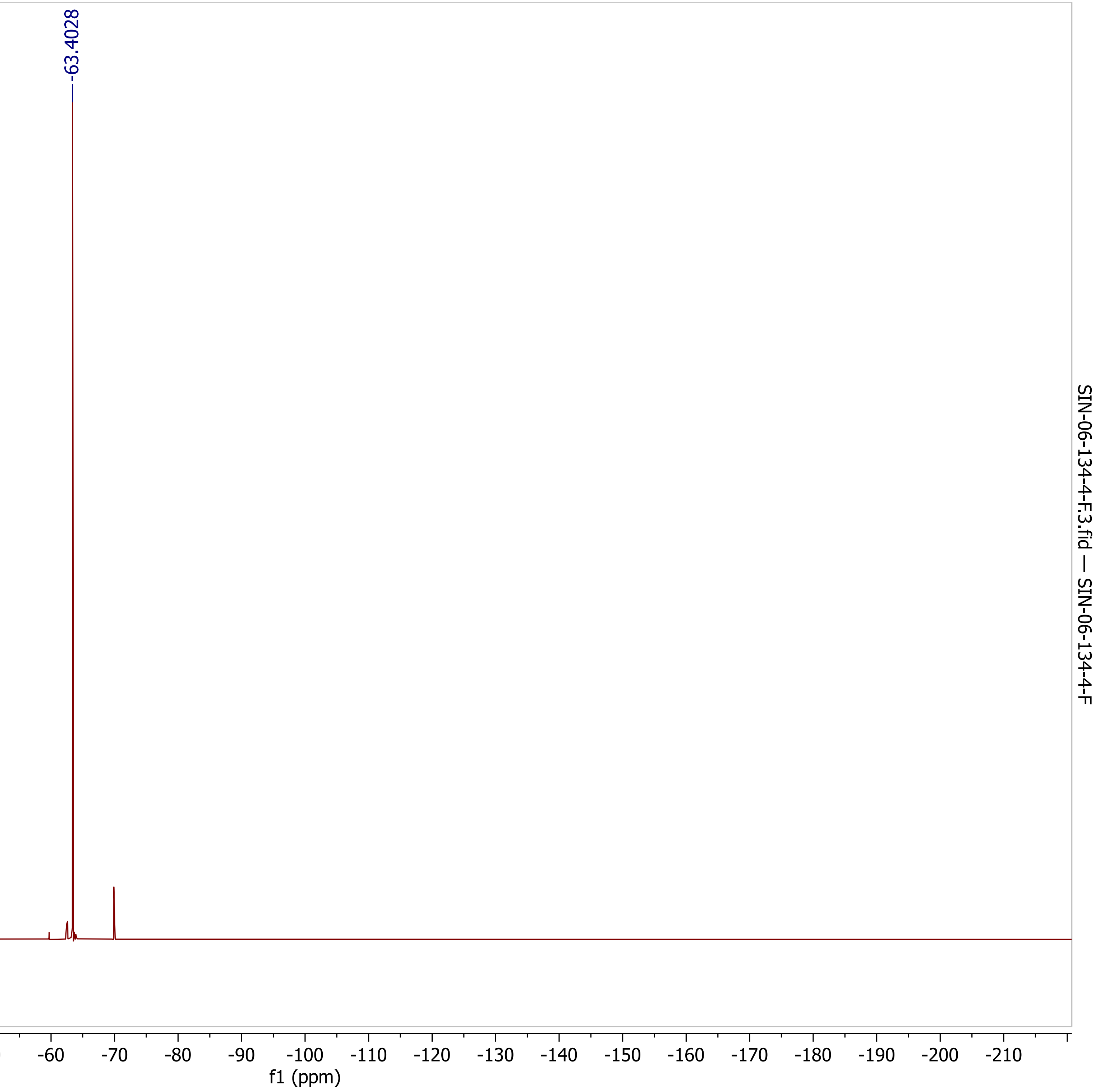

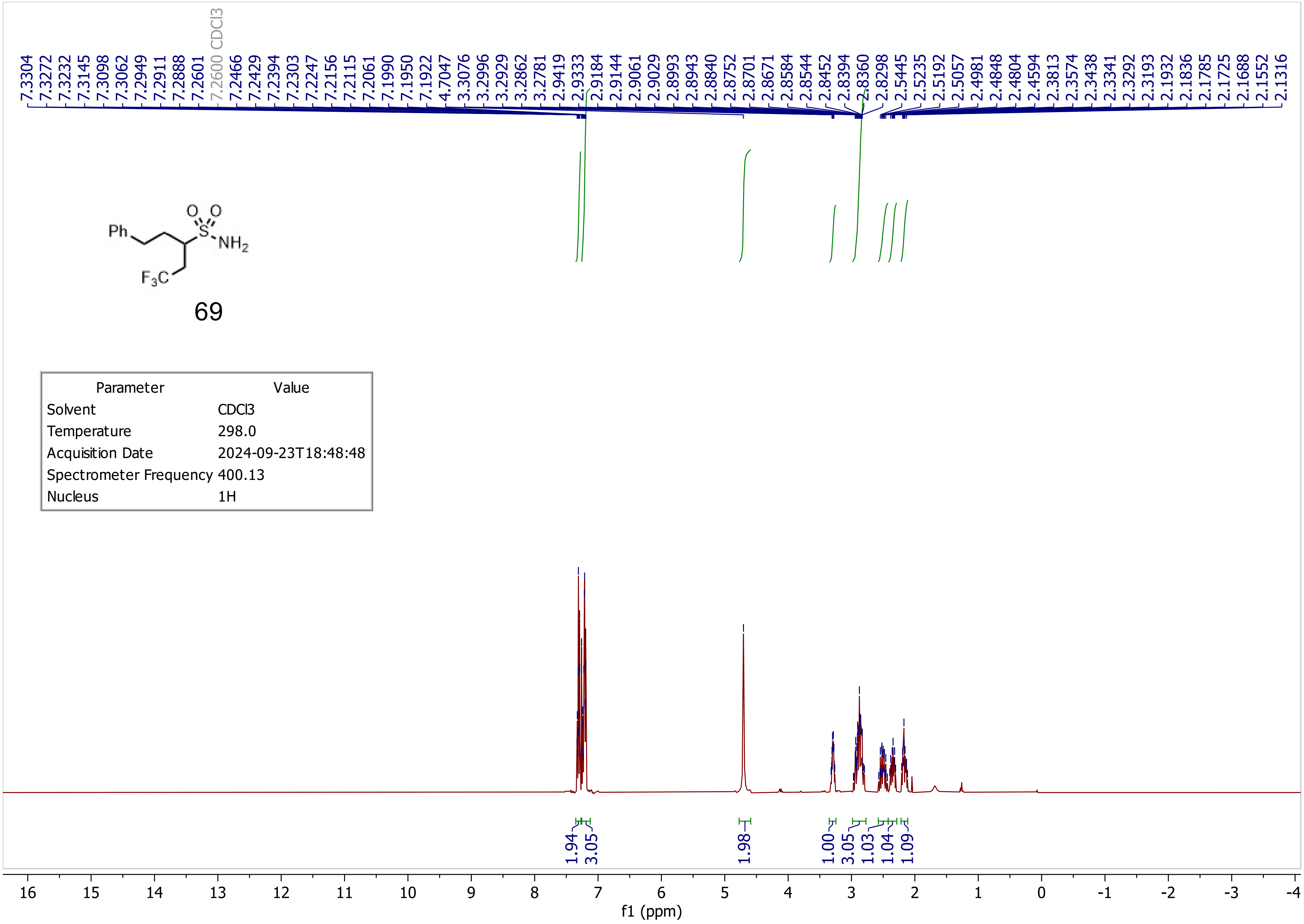

Parameter	Value
Solvent	CDCl3
Temperature	298.0
Acquisition Date	2024-09-23T18:48:48
Spectrometer Frequency	400.13
Nucleus	1H

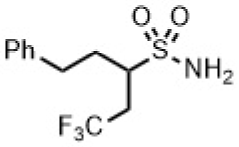

69

Parameter	Value
Solvent	CDCl3
Temperature	298.0
Acquisition Date	2024-09-24T02:59:44
Spectrometer Frequency	100.62
Nucleus	13C

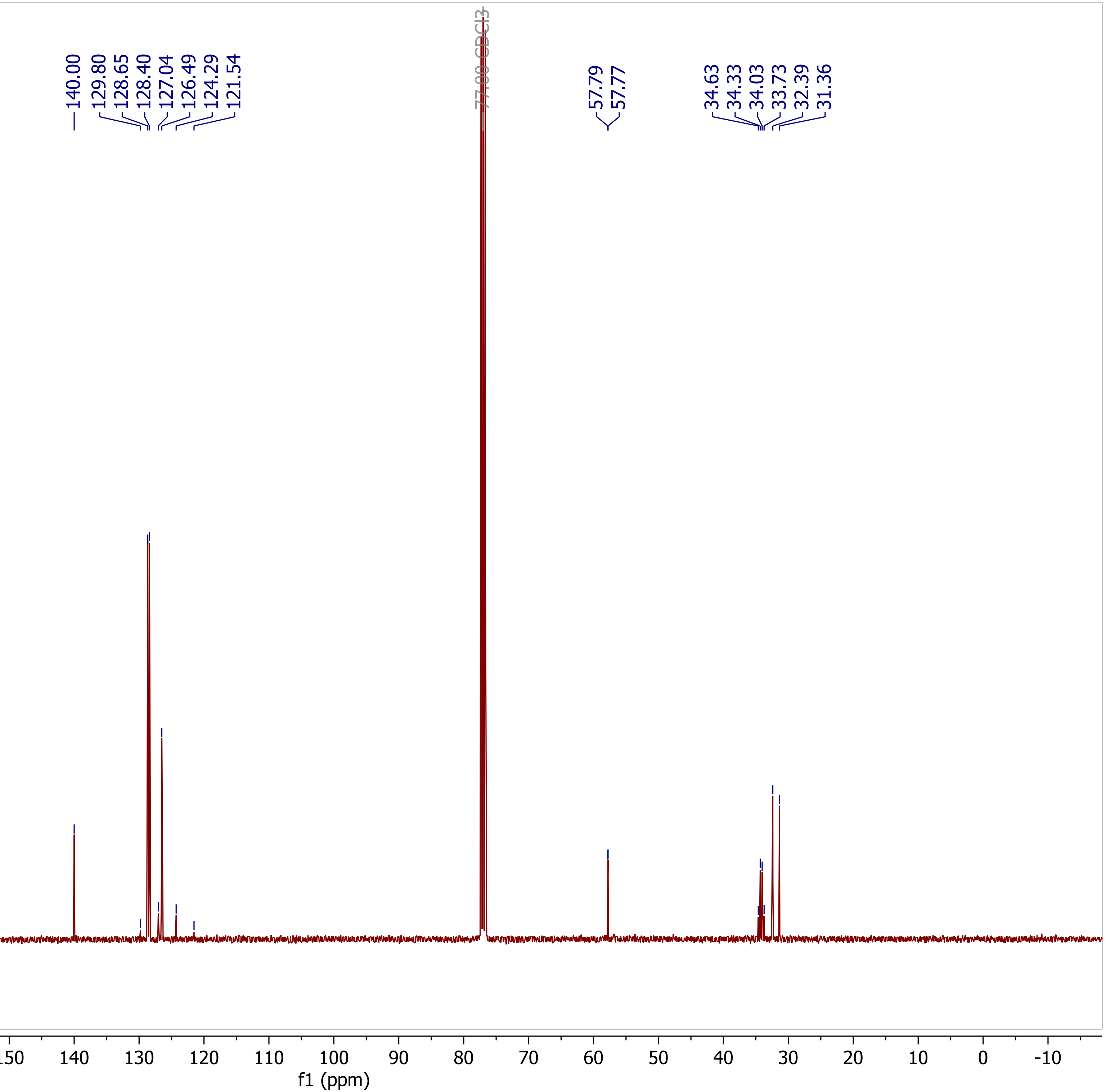

SIN-06-117-1-Pure-c13.5.fid — SIN-06-117-1-Pure-c13

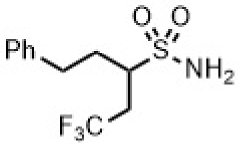

69

Parameter	Value
Solvent	CDCl3
Temperature	298.0
Acquisition Date	2024-09-23T20:28:02
Spectrometer Frequency	376.46
Nucleus	19F

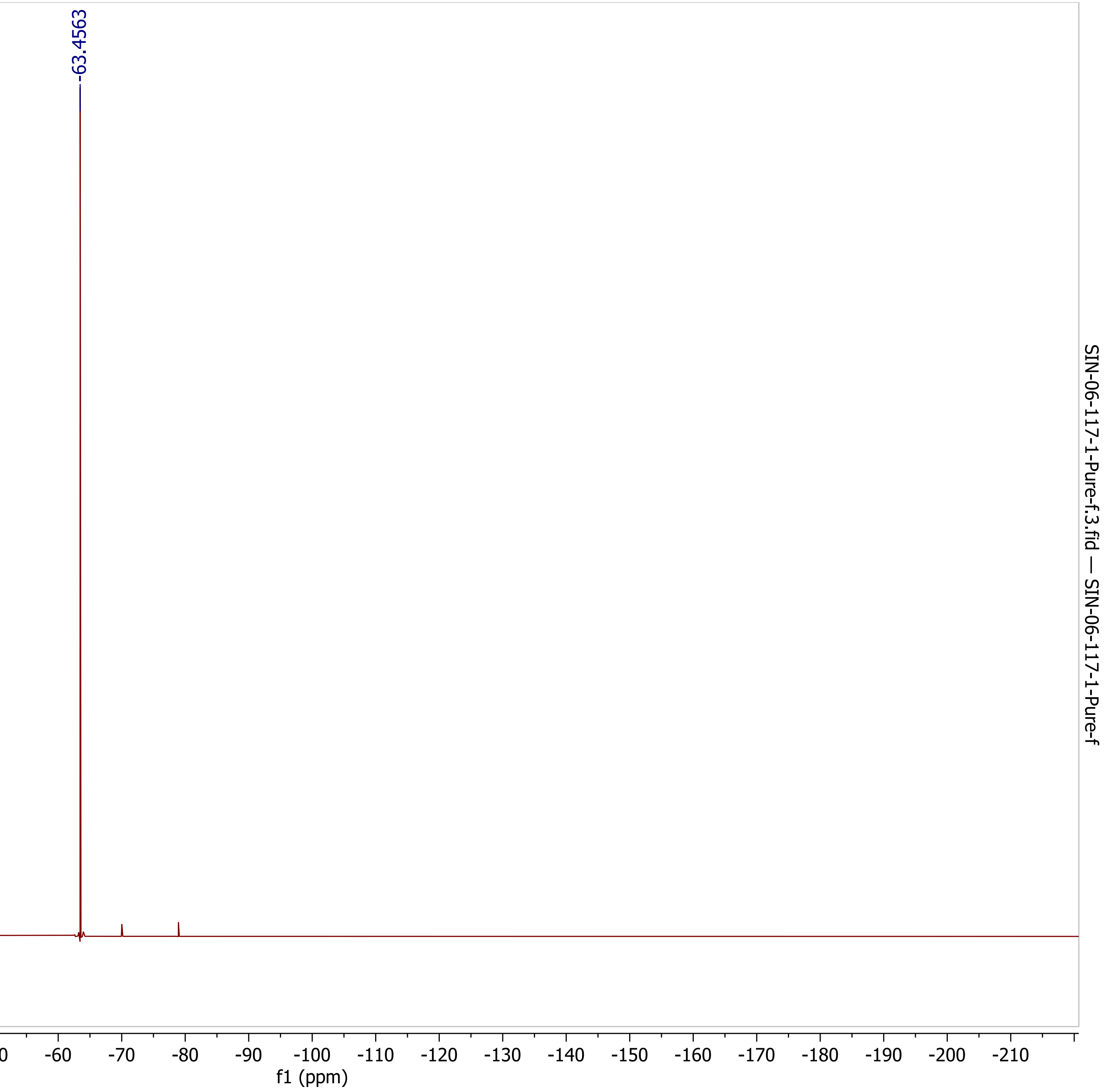

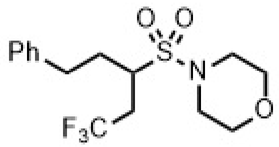

70

Parameter	Value
Solvent	CDCl3
Temperature	298.0
Acquisition Date	2024-10-15T16:16:40
Spectrometer Frequency	400.13
Nucleus	1H

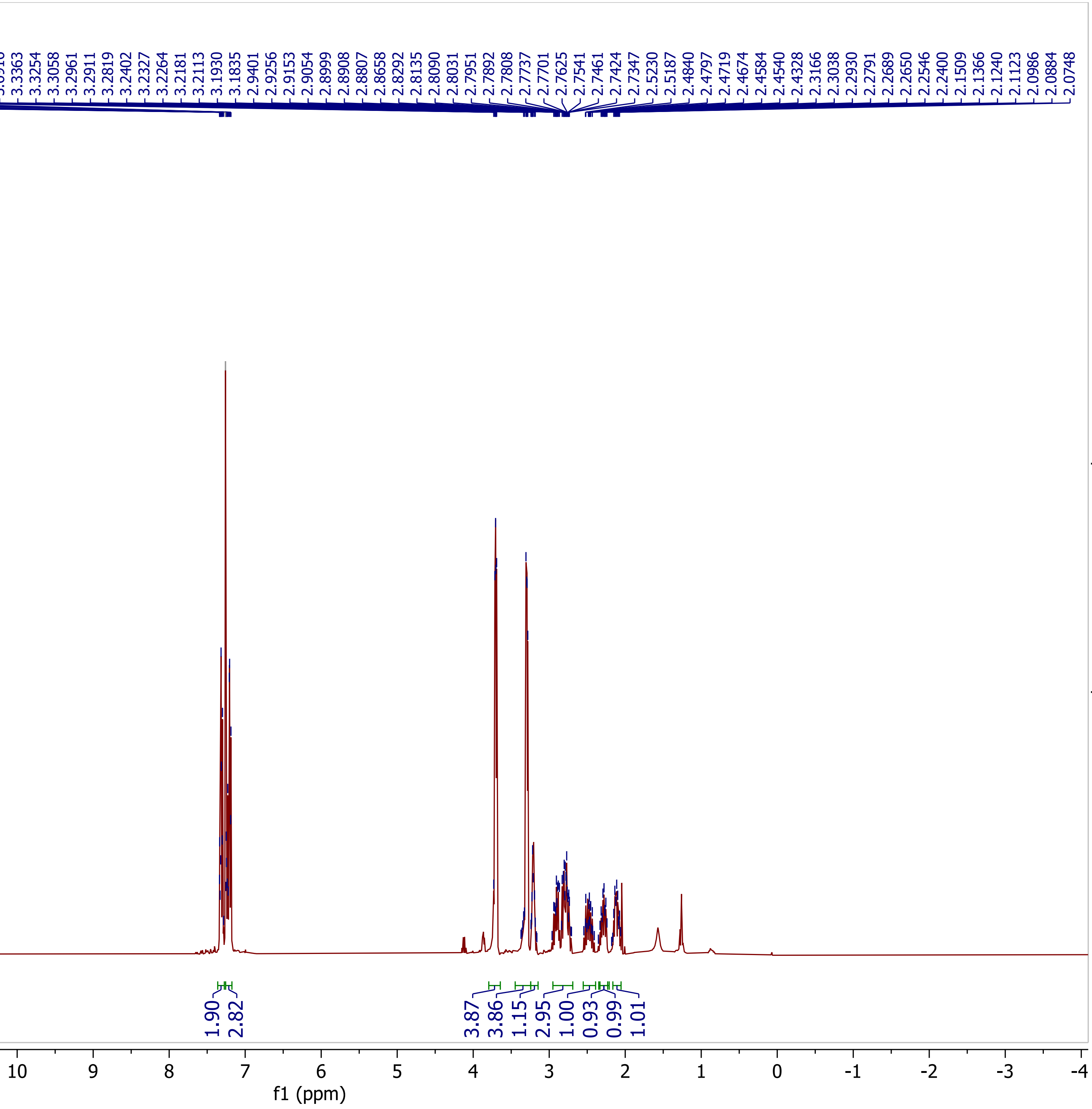

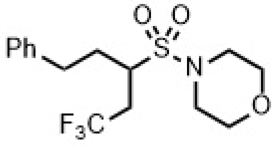

70

Parameter	Value
Solvent	CDCl3
Temperature	298.0
Acquisition Date	2024-10-15T02:11:45
Spectrometer Frequency	100.62
Nucleus	13C

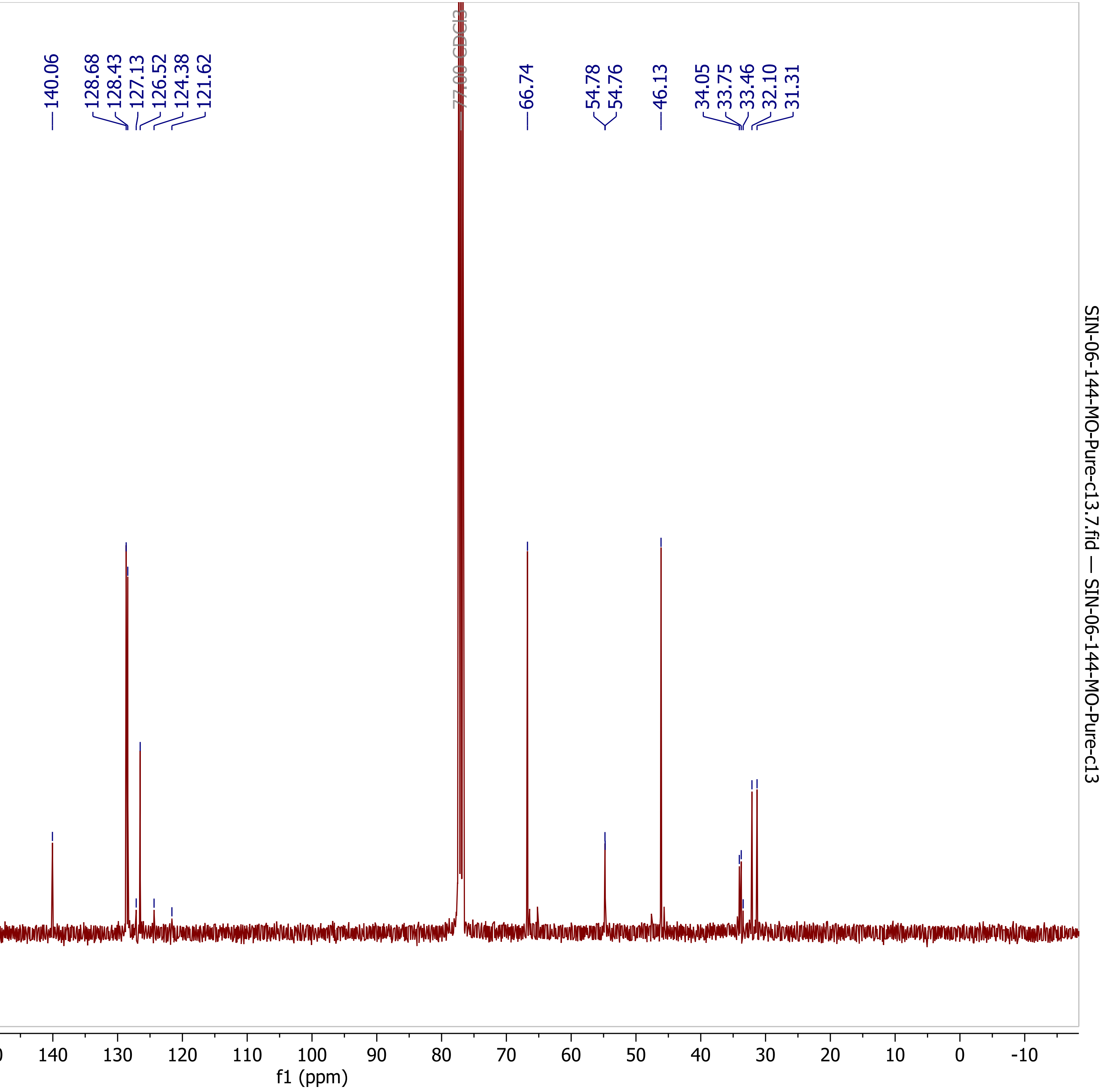

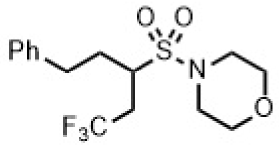

70

Parameter	Value
Solvent	CDCl3
Temperature	298.0
Acquisition Date	2024-10-14T18:11:52
Spectrometer Frequency	376.46
Nucleus	19F

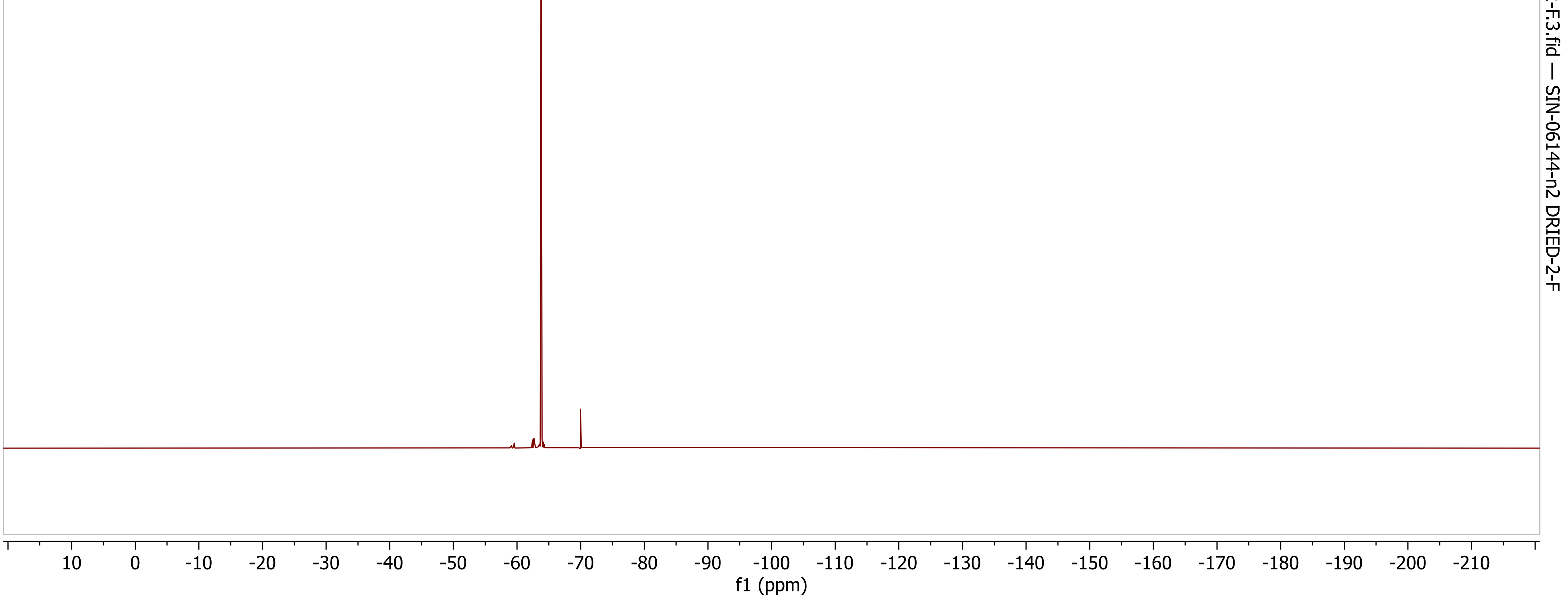

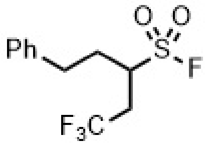

71

Parameter	Value
Solvent	CDCl3
Temperature	298.0
Acquisition Date	2024-09-23T18:43:16
Spectrometer Frequency	400.13
Nucleus	1H

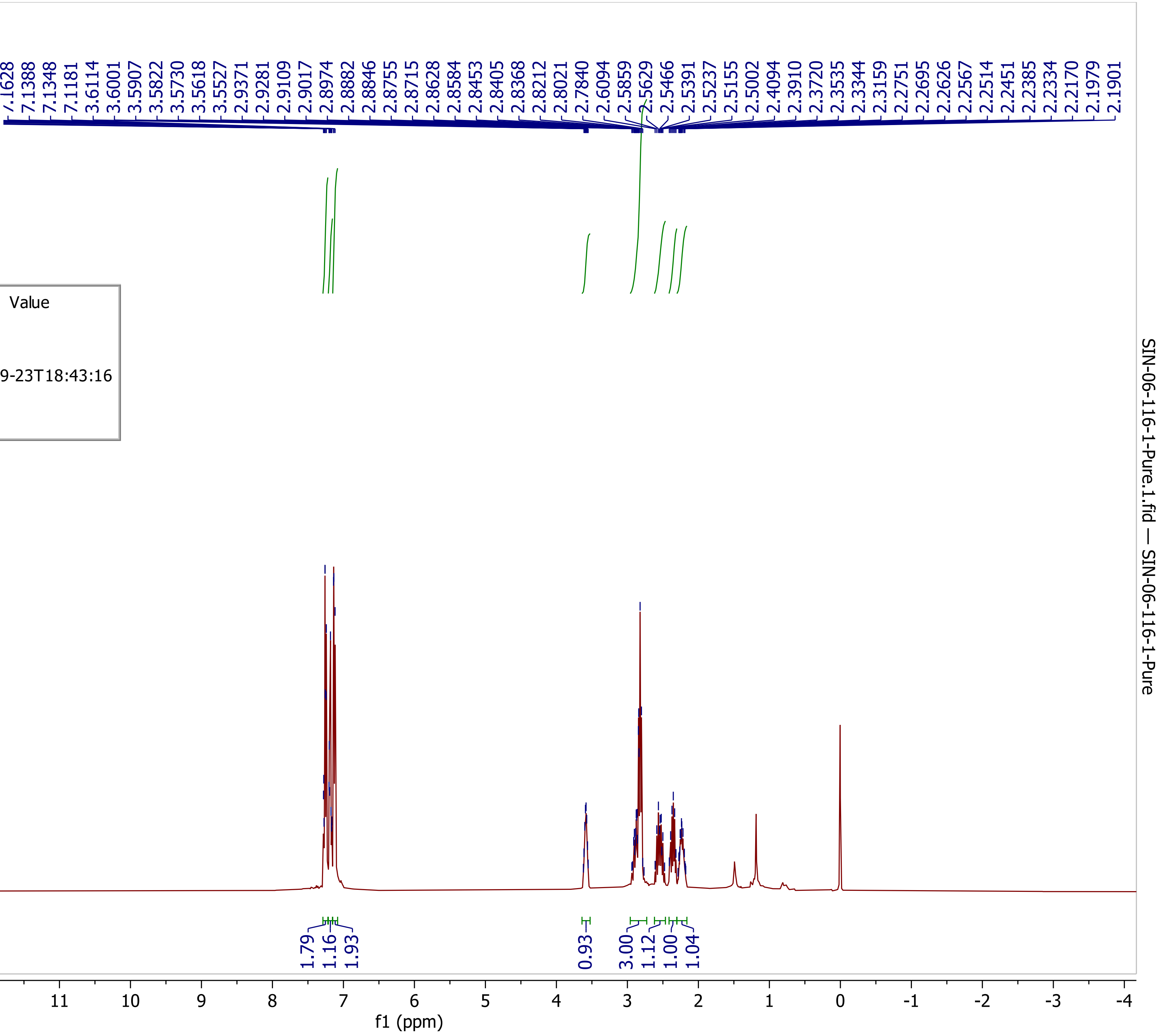

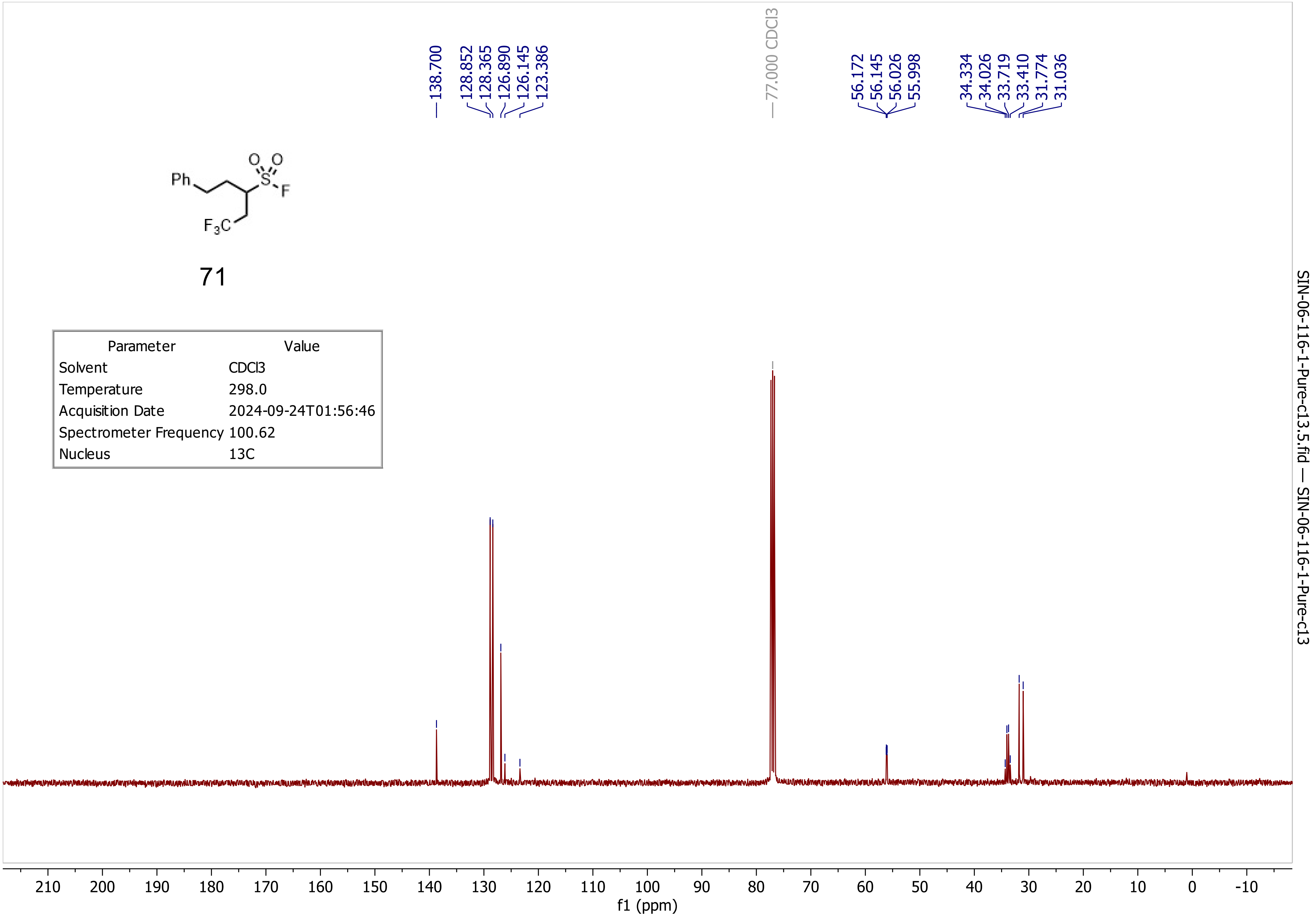

71

Parameter	Value
Solvent	CDCl3
Temperature	298.0
Acquisition Date	2024-09-24T01:56:46
Spectrometer Frequency	100.62
Nucleus	13C

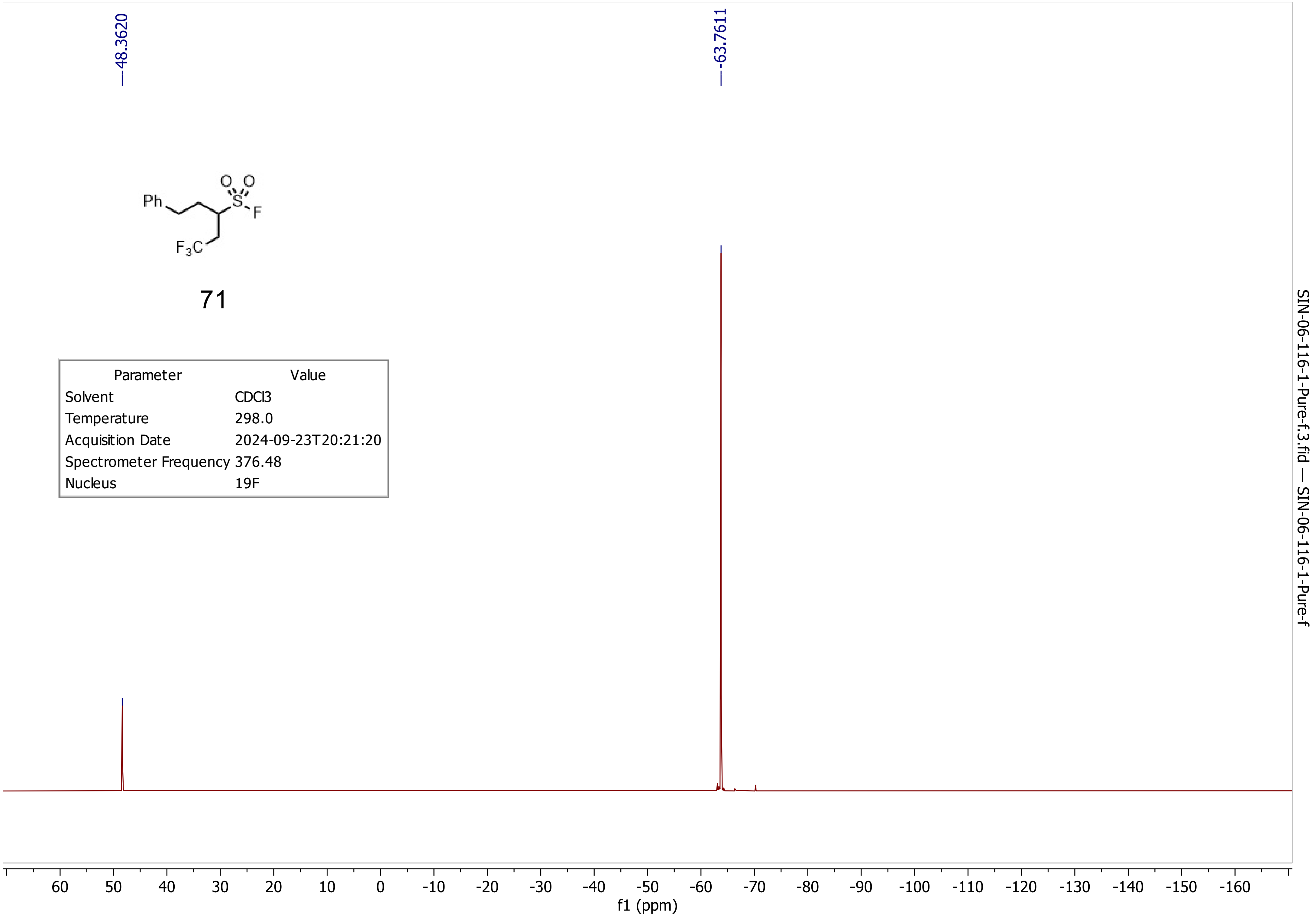

Parameter	Value
Solvent	CDCl3
Temperature	298.0
Acquisition Date	2024-09-23T20:21:20
Spectrometer Frequency	376.48
Nucleus	19F

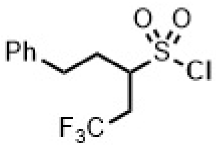

72

Parameter	Value
Solvent	CDCl3
Temperature	298.0
Acquisition Date	2024-10-26T18:43:47
Spectrometer Frequency	400.13
Nucleus	1H

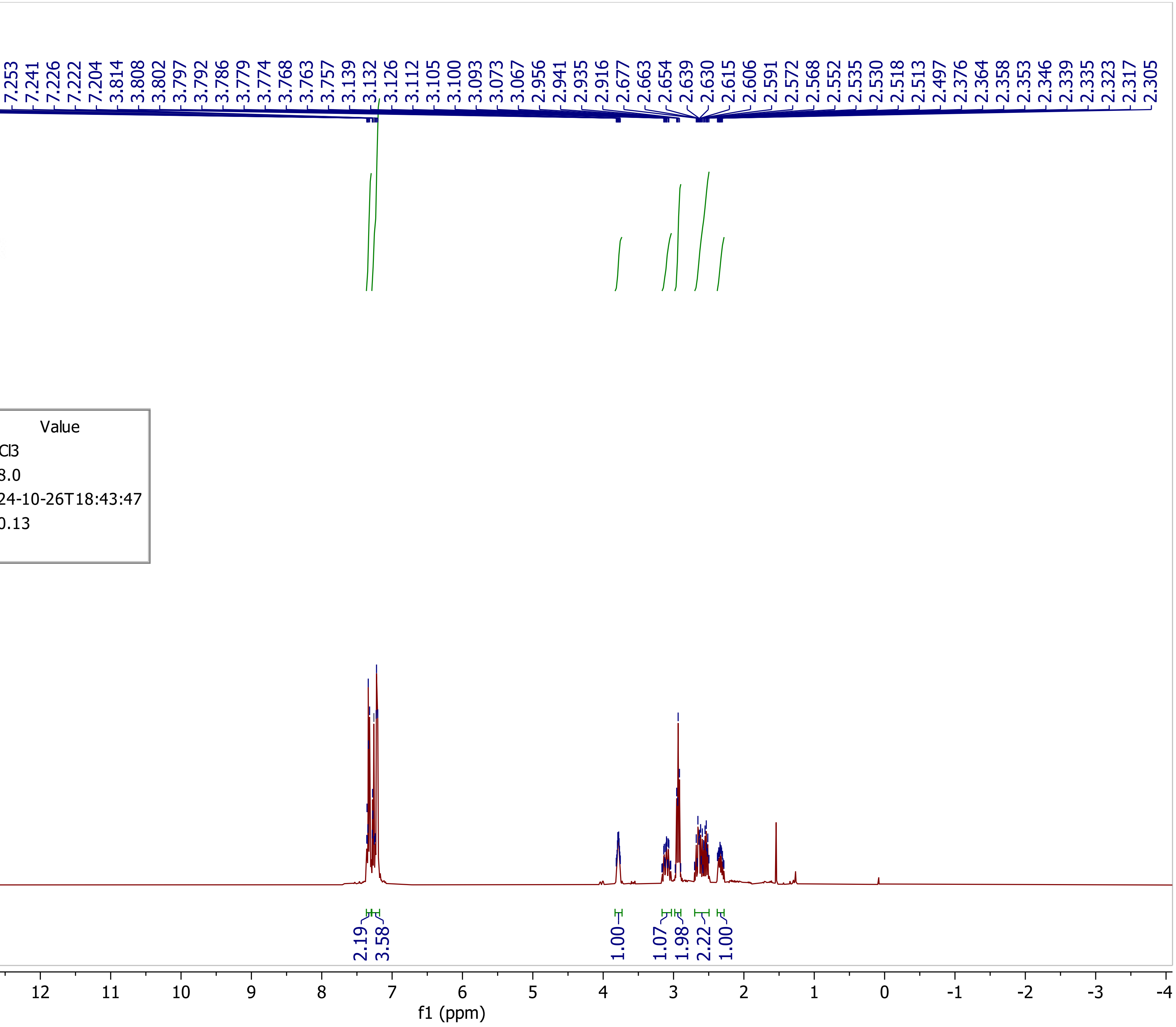

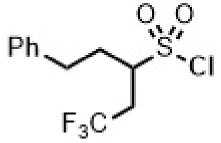

72

Parameter	Value
Solvent	CDCl3
Temperature	298.0
Acquisition Date	2024-10-26T22:18:05
Spectrometer Frequency	100.62
Nucleus	13C

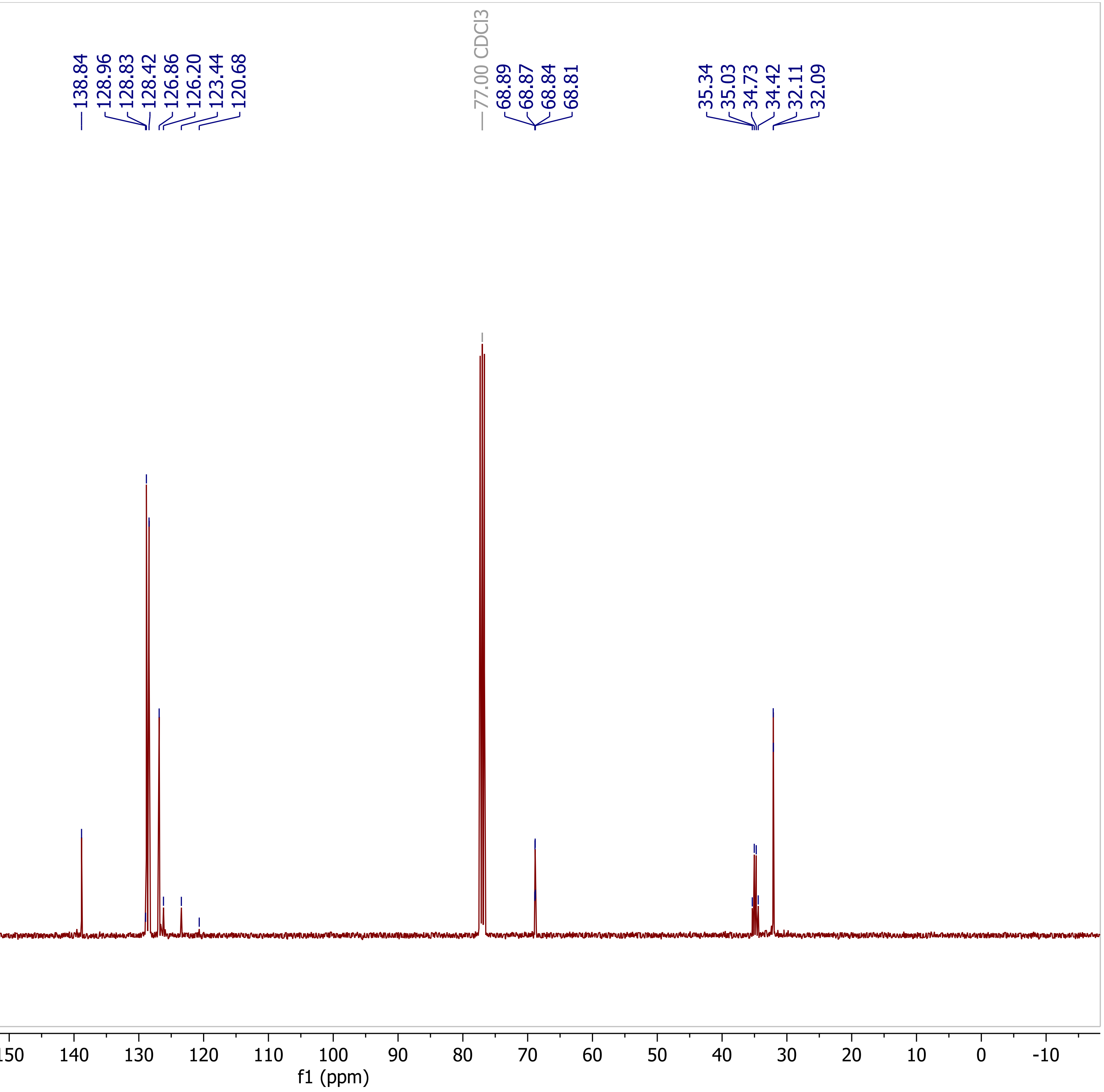

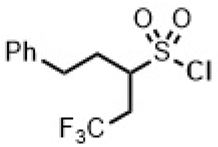

72

Parameter	Value
Solvent	CDCl3
Temperature	298.0
Acquisition Date	2024-10-26T18:48:05
Spectrometer Frequency	376.46
Nucleus	19F

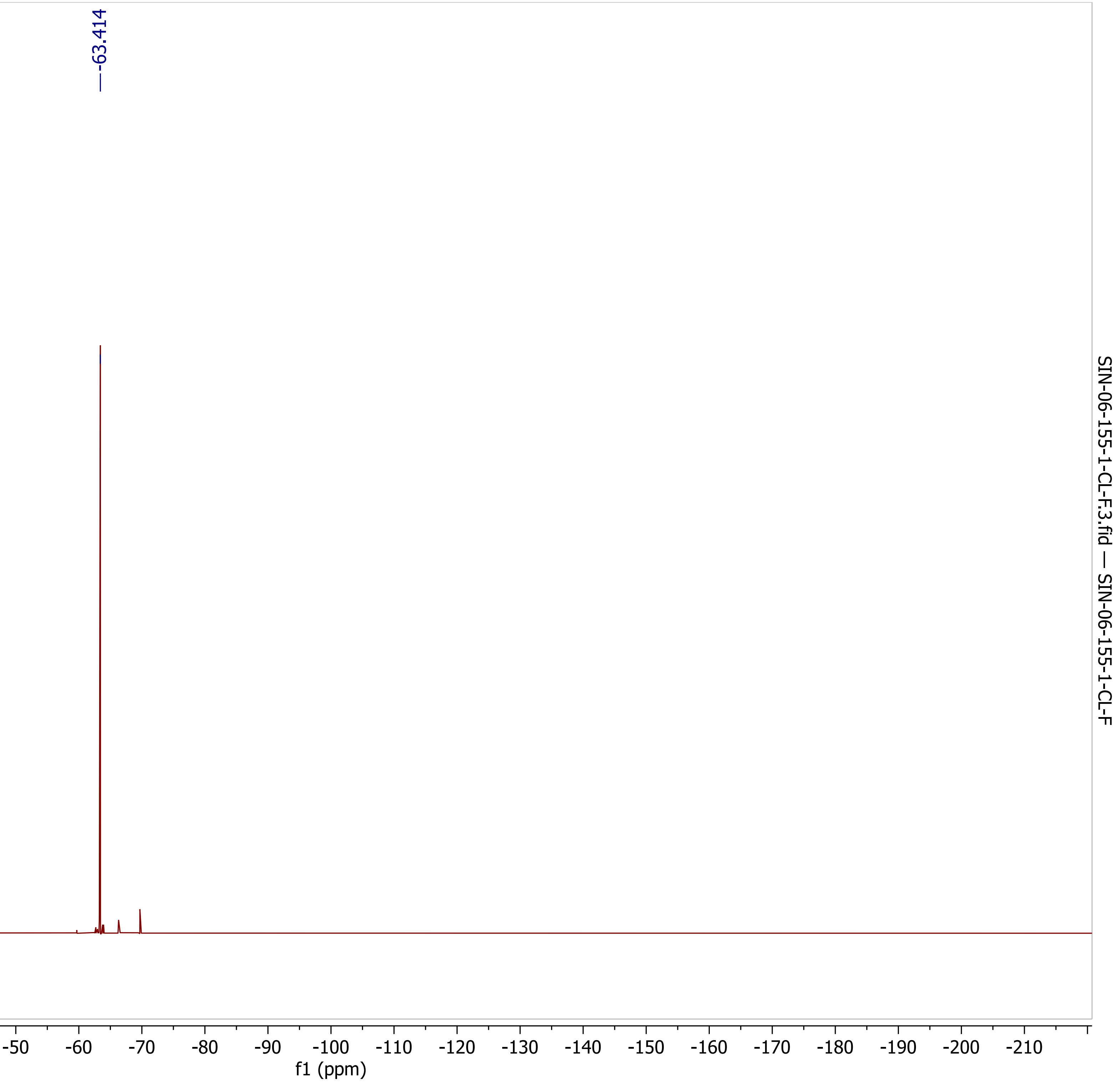

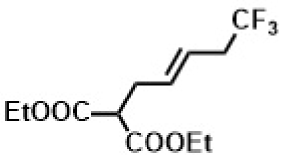

90

Parameter	Value
Solvent	CDCl3
Temperature	298.0
Acquisition Date	2024-04-11T12:42:08
Spectrometer Frequency	400.13
Nucleus	1H

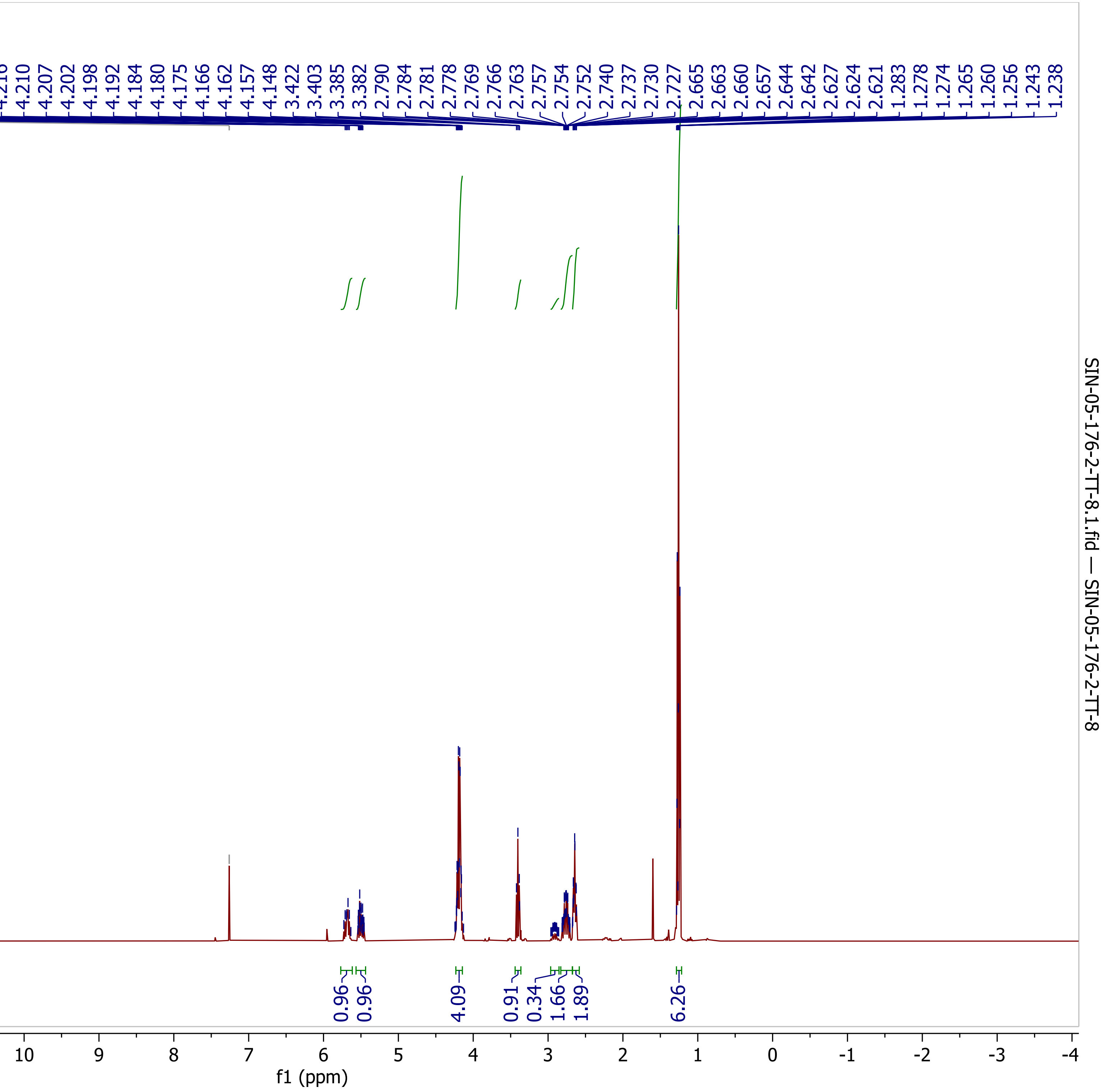

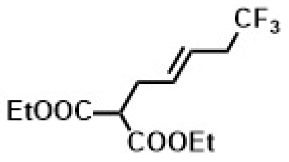

90

Parameter	Value
Solvent	CDCl3
Temperature	298.0
Acquisition Date	2024-04-11T00:40:45
Spectrometer Frequency	100.62
Nucleus	13C

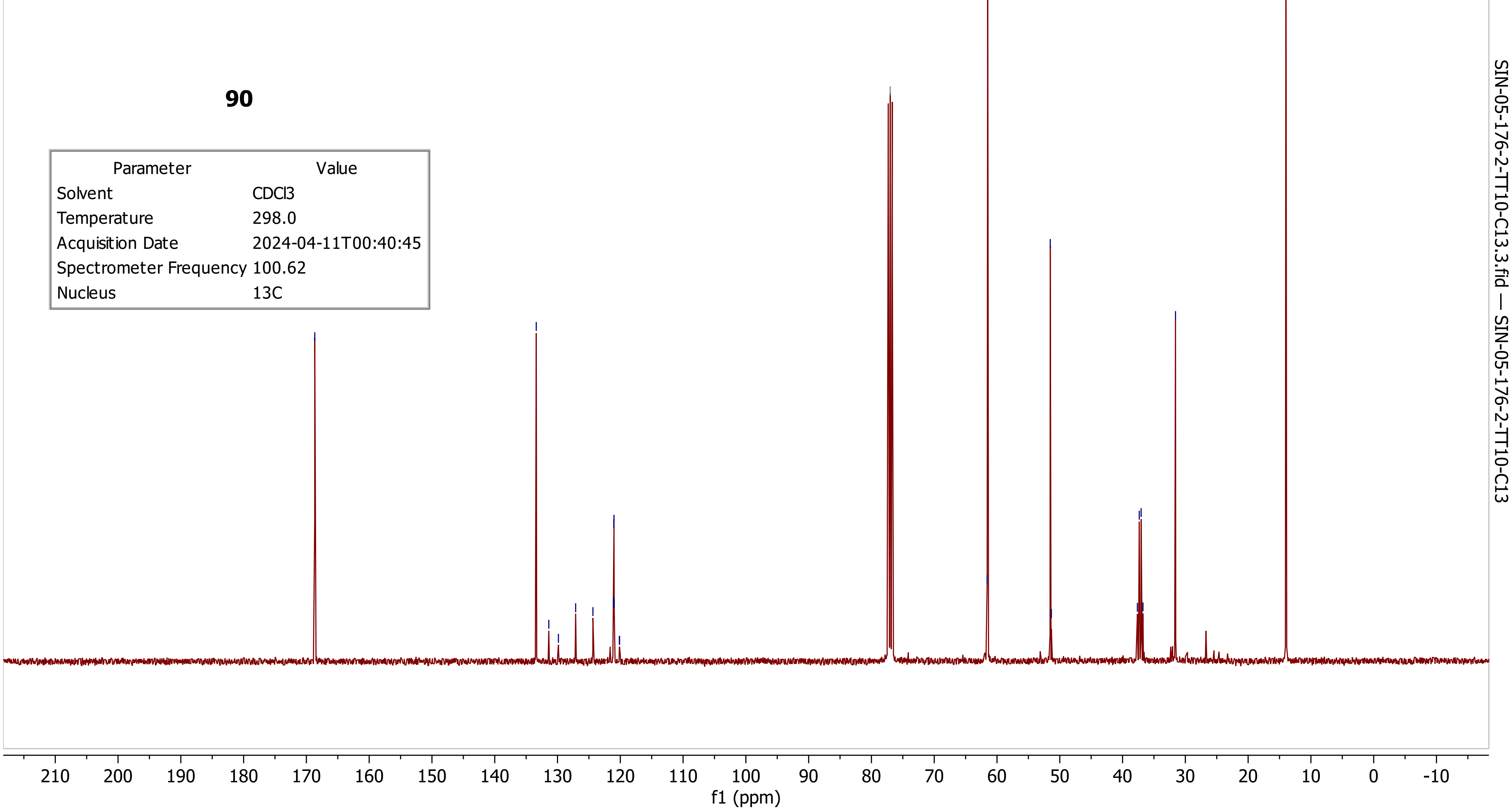

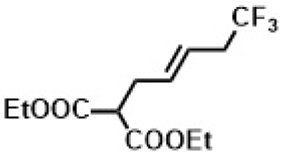

90

Parameter	Value
Solvent	CDCl3
Temperature	298.0
Acquisition Date	2024-04-11T13:55:42
Spectrometer Frequency	376.46
Nucleus	19F

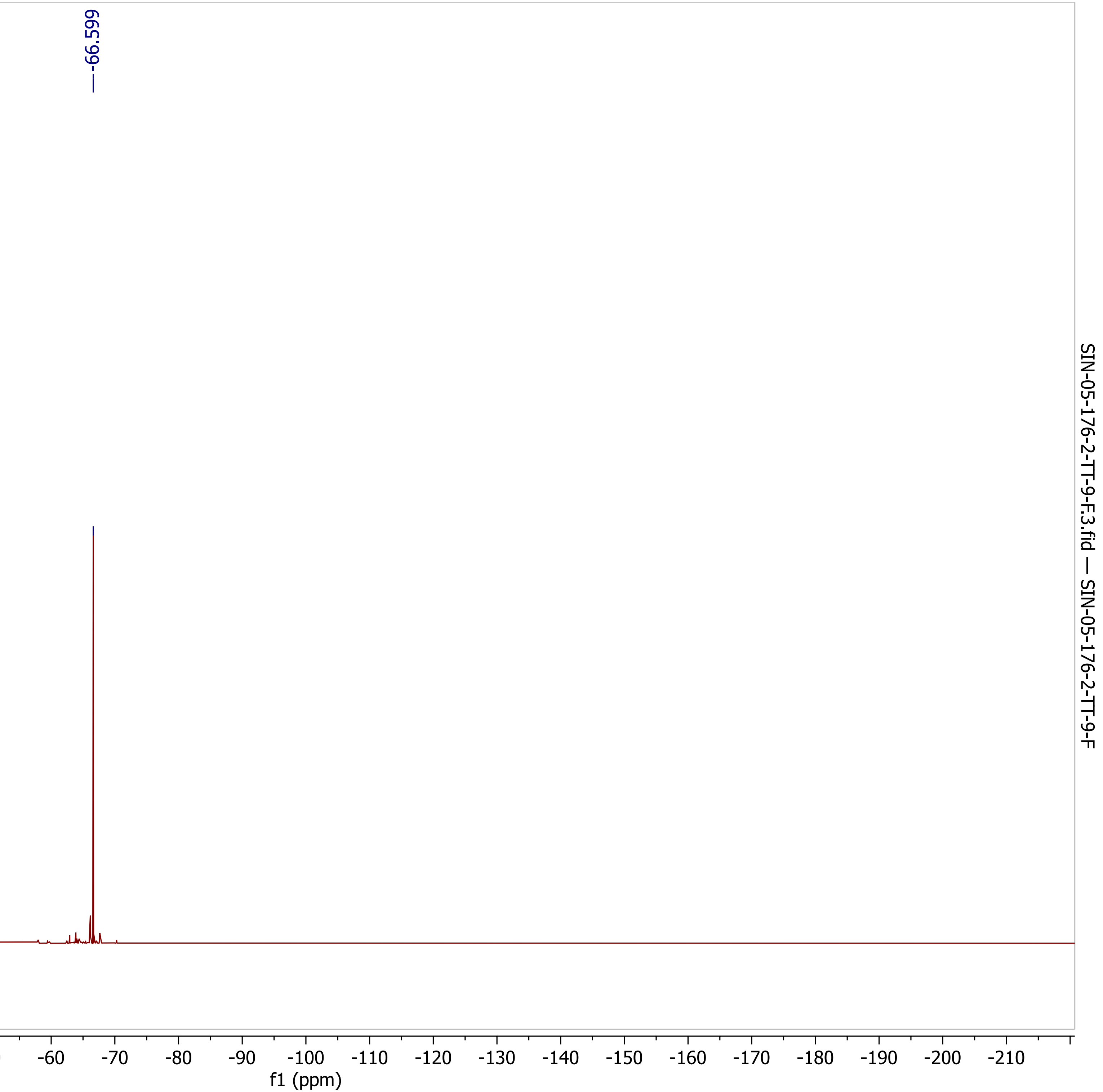

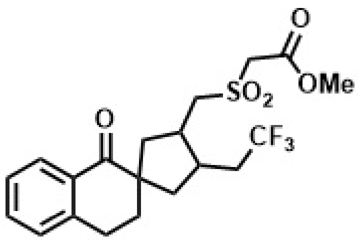

92

Parameter	Value
Solvent	CDCl3
Temperature	298.0
Acquisition Date	2024-04-25T15:27:03
Spectrometer Frequency	400.13
Nucleus	1H

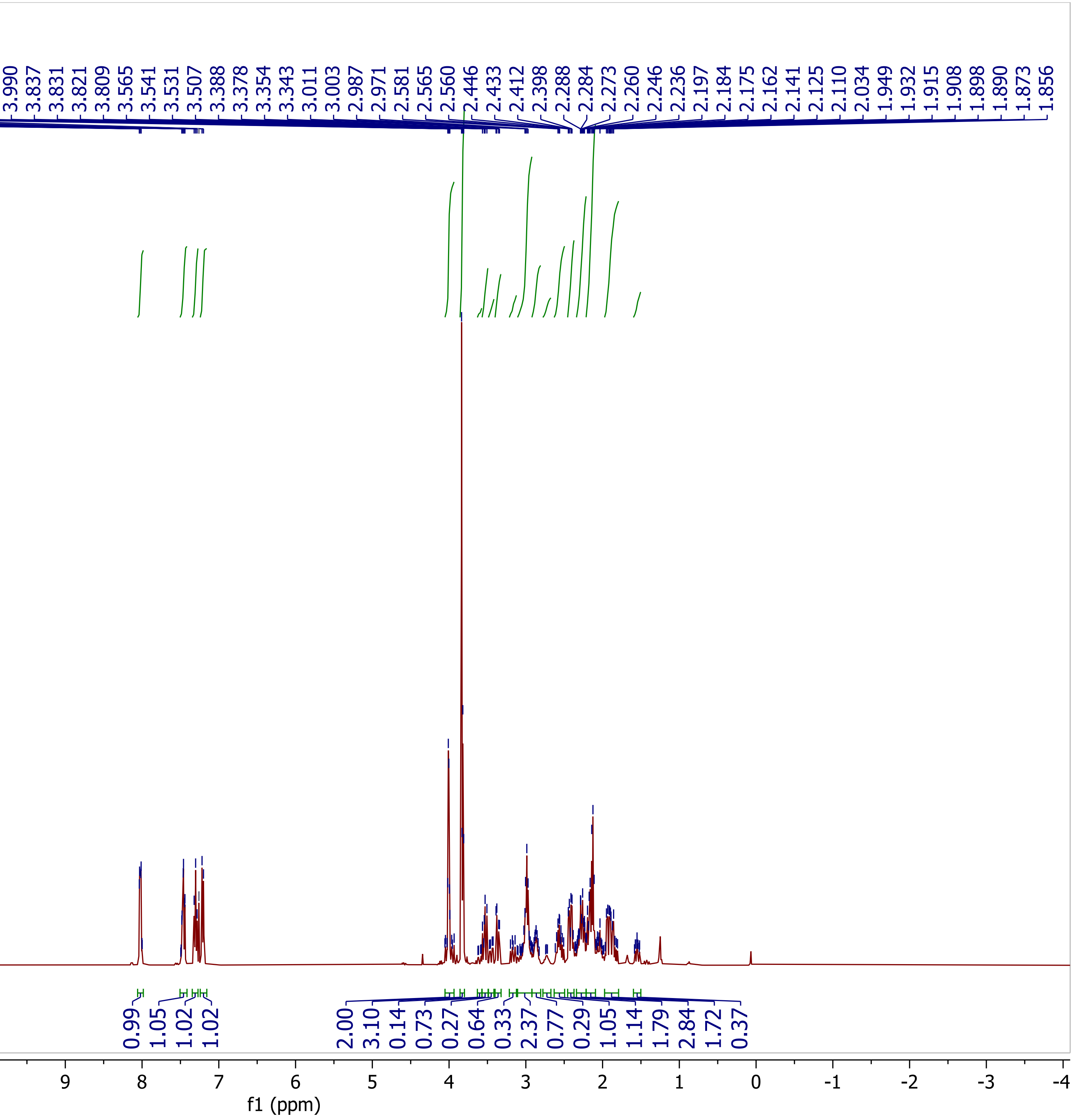

SIN-05-189-1-TT-21-retake.1.fid — SIN-05-189-1-TT-21-retake

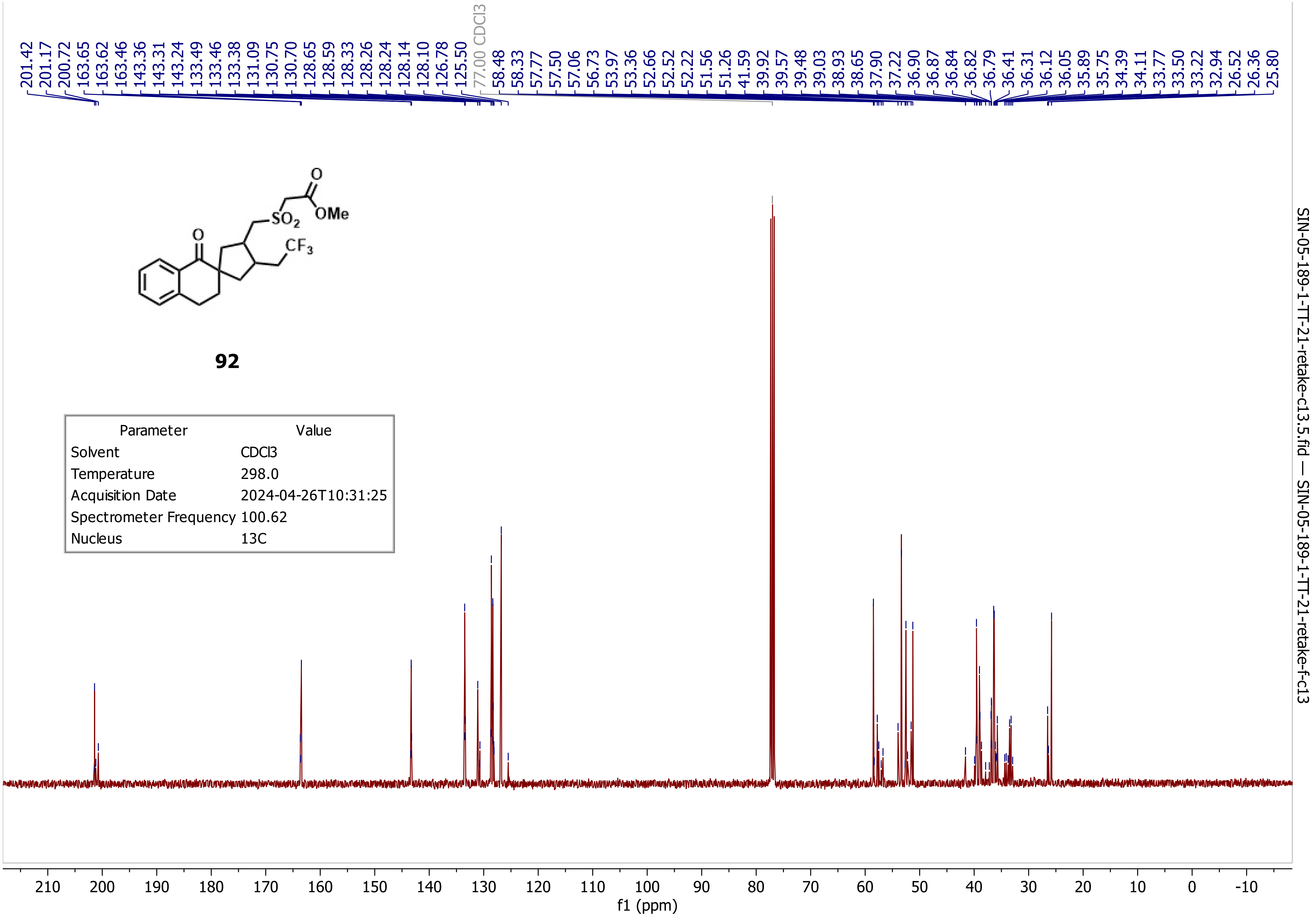

92

Parameter	Value
Solvent	CDCl3
Temperature	298.0
Acquisition Date	2024-04-26T10:31:25
Spectrometer Frequency	100.62
Nucleus	13C

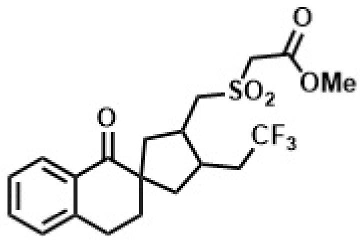

92

Parameter	Value
Solvent	CDCl3
Temperature	298.0
Acquisition Date	2024-04-25T16:03:30
Spectrometer Frequency	376.46
Nucleus	19F

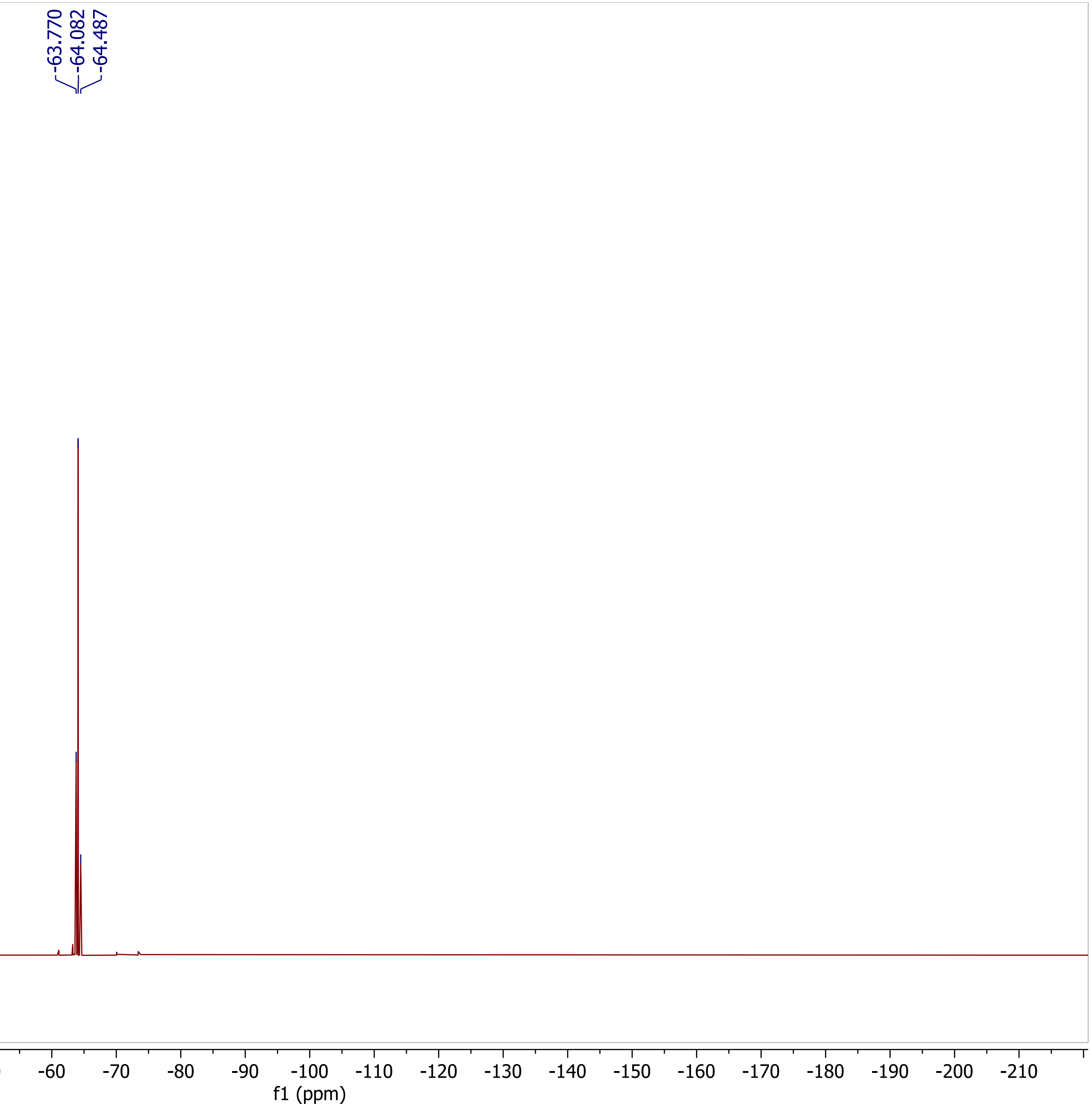

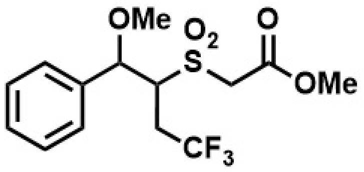

94

Parameter	Value
1 Solvent	CDCl3
2 Acquisition Date	2025-12-07T17:39:24
3 Spectrometer Frequency	400.13
4 Nucleus	1H

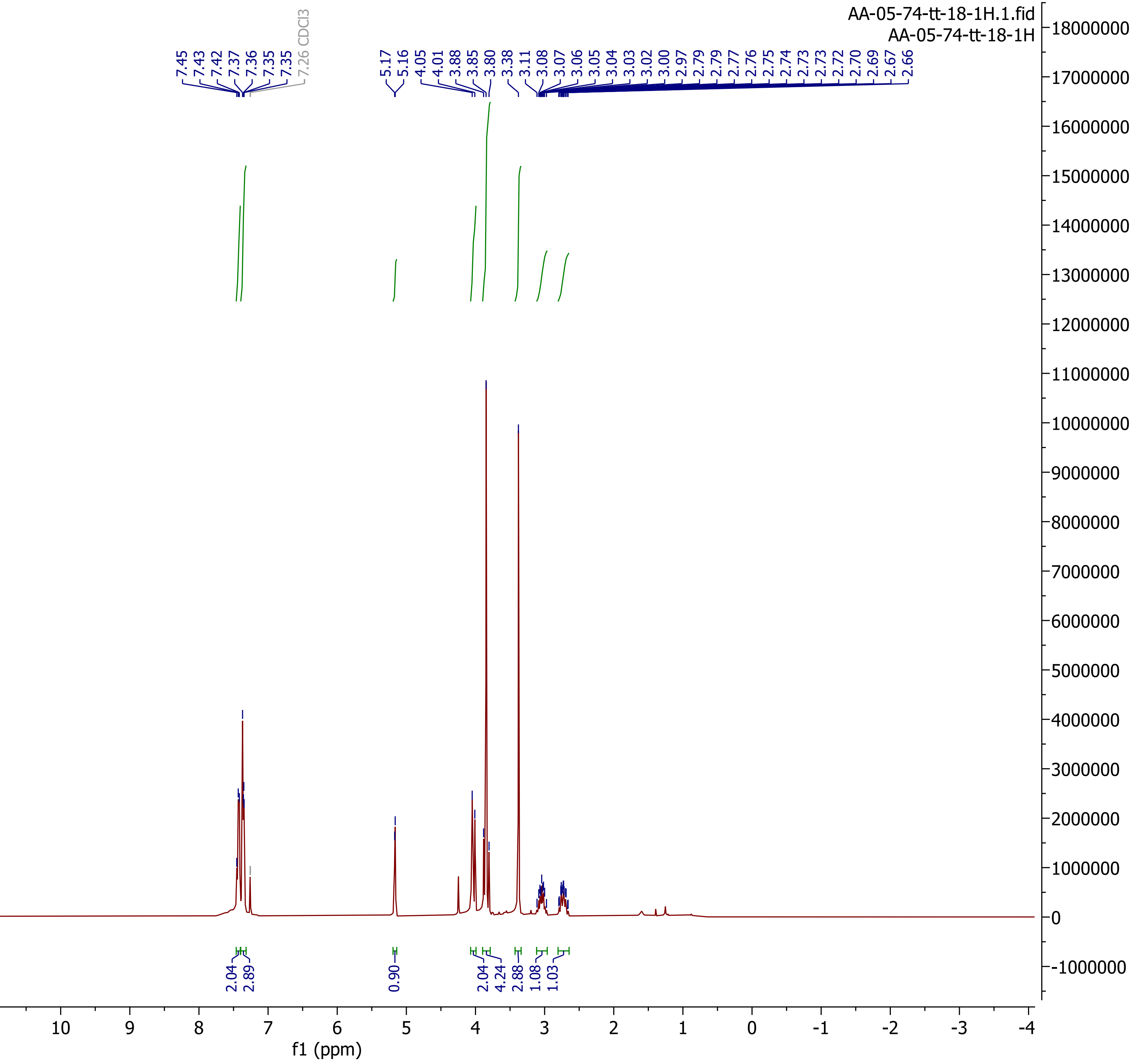

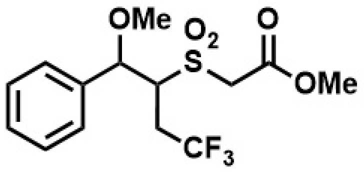

94

Parameter	Value
1 Solvent	CDCl3
2 Acquisition Date	2025-12-07T18:39:47
3 Spectrometer Frequency	100.62
4 Nucleus	13C

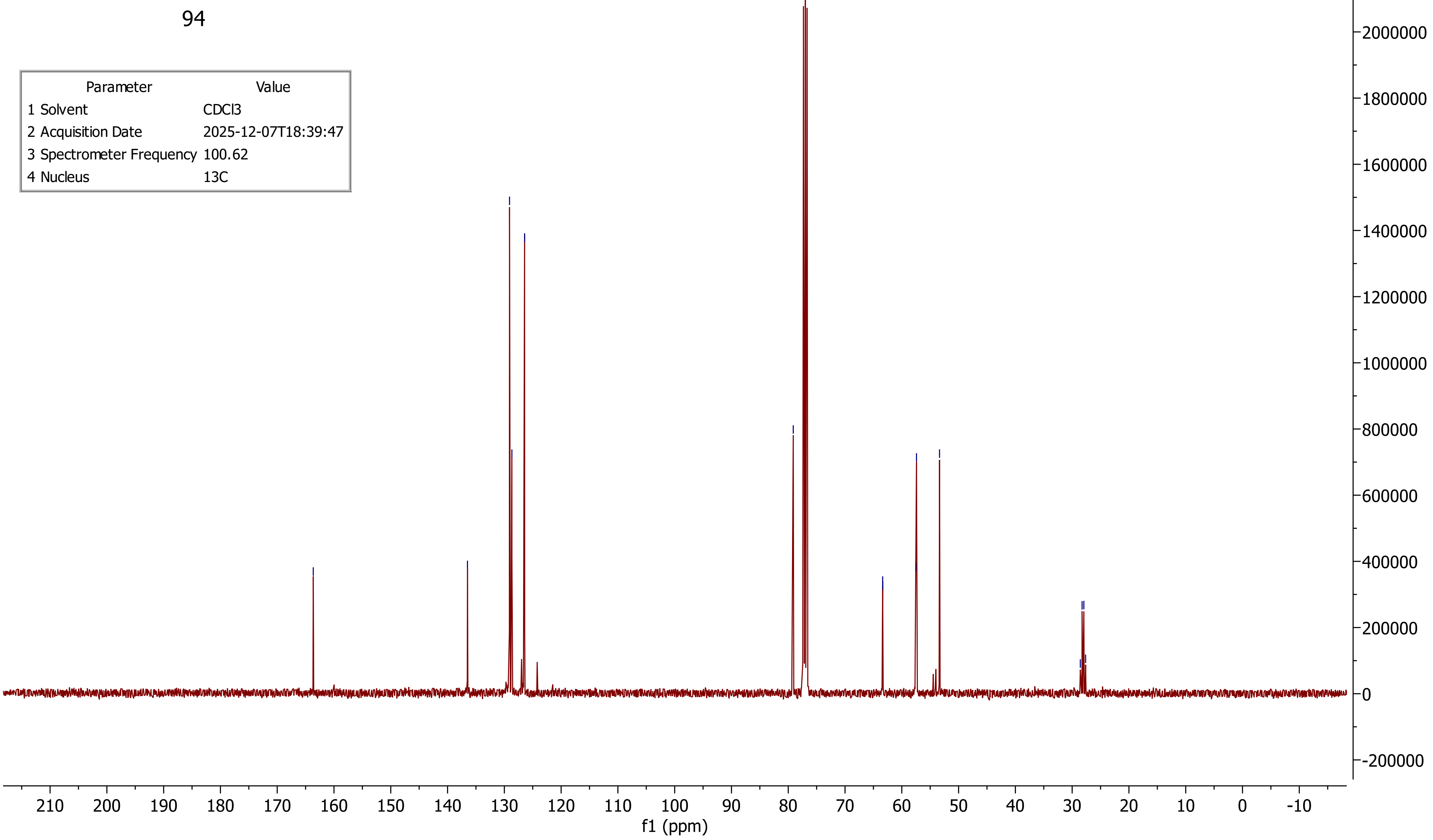

Parameter	Value
1 Solvent	CDCl3
2 Acquisition Date	2025-12-07T18:44:21
3 Spectrometer Frequency	376.46
4 Nucleus	19F

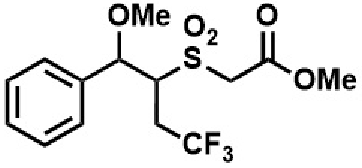

94

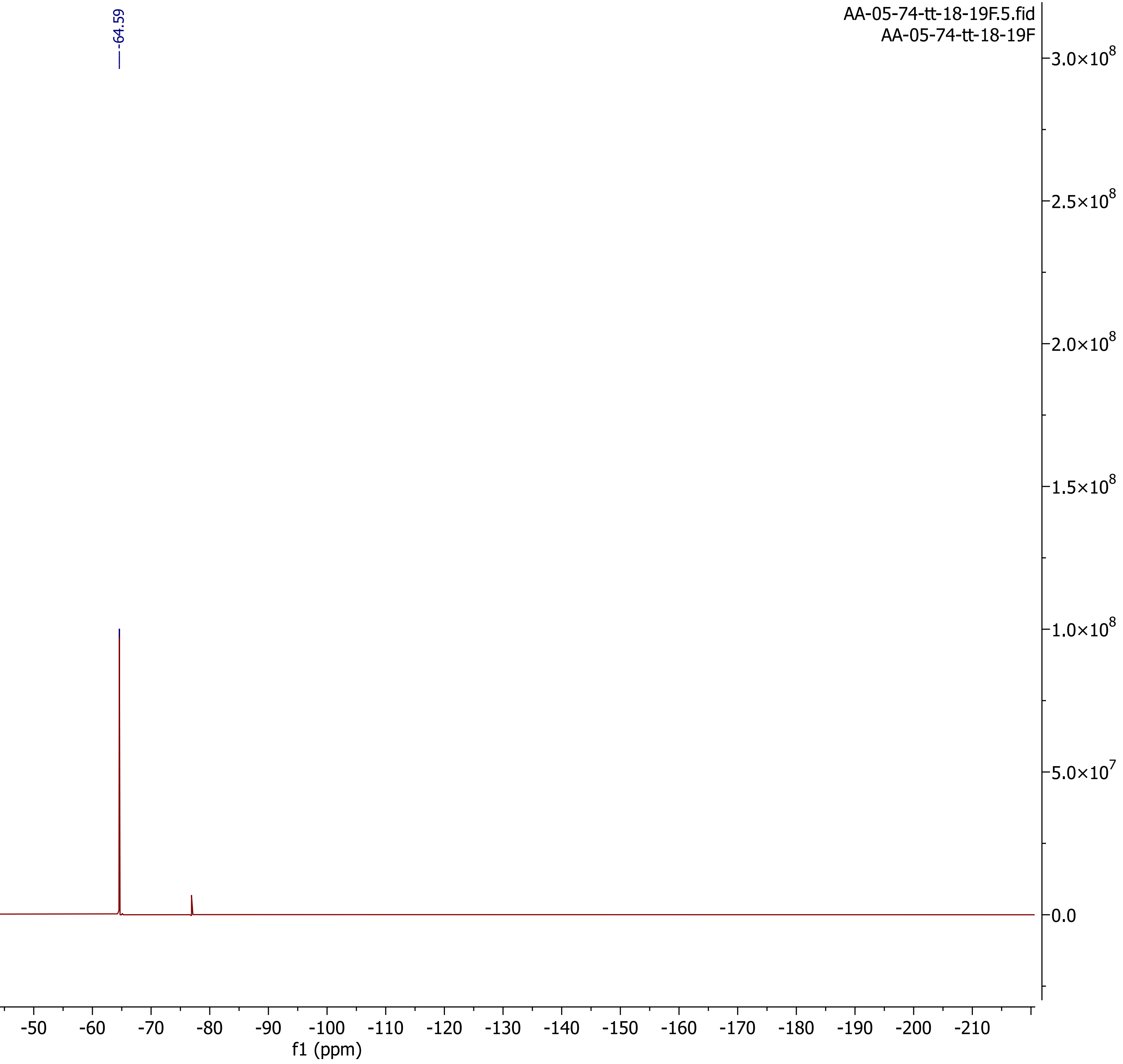

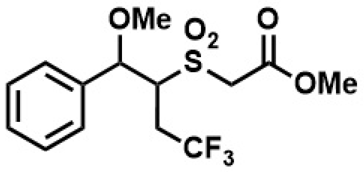

94

Parameter	Value
1 Solvent	CDCl3
2 Acquisition Date	2025-12-07T20:07:01
3 Spectrometer Frequency	400.13
4 Nucleus	1H

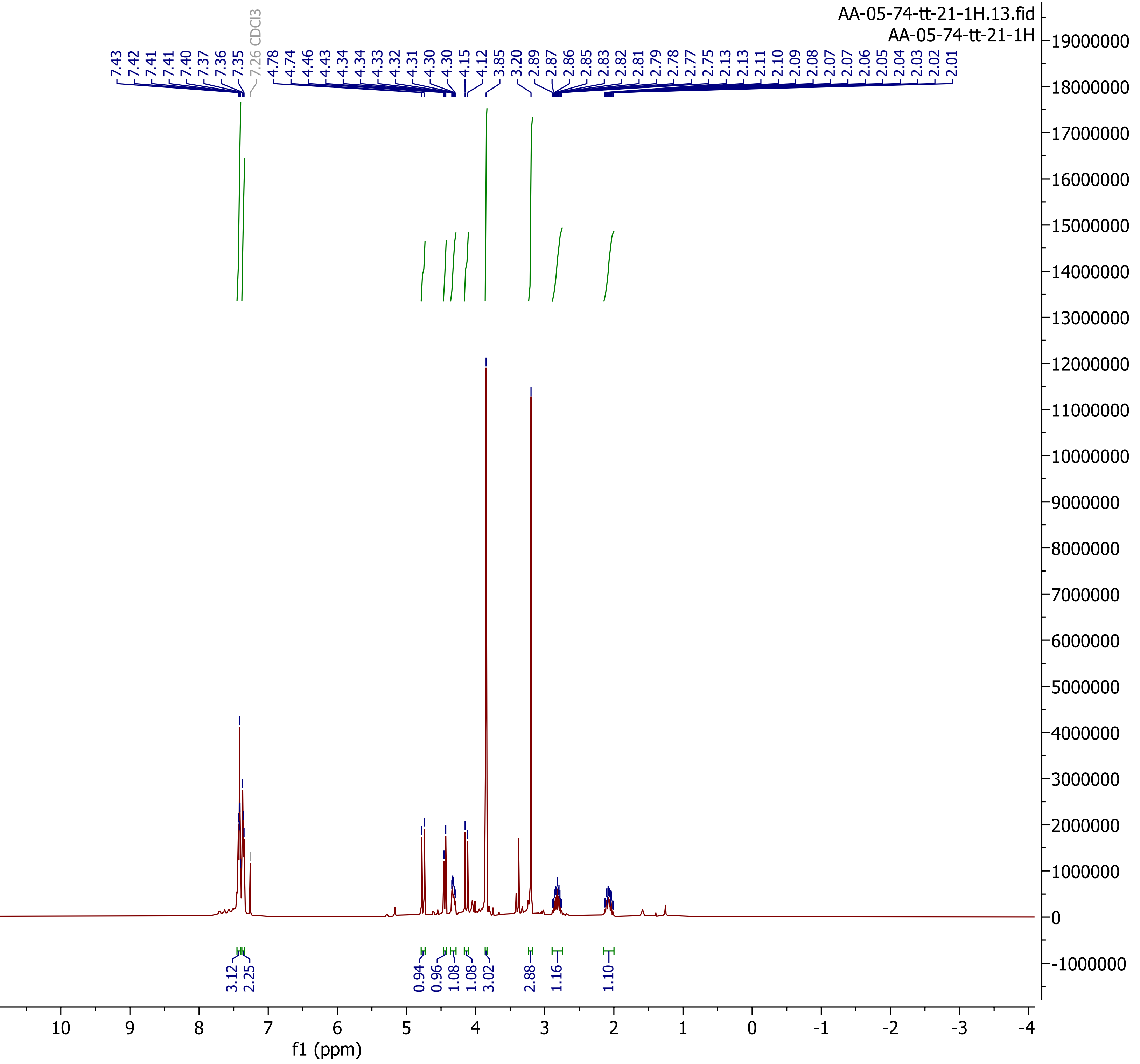

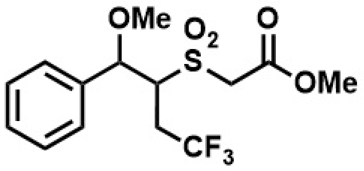

94

Parameter	Value
1 Solvent	CDCl3
2 Acquisition Date	2025-12-07T21:07:24
3 Spectrometer Frequency	100.62
4 Nucleus	13C

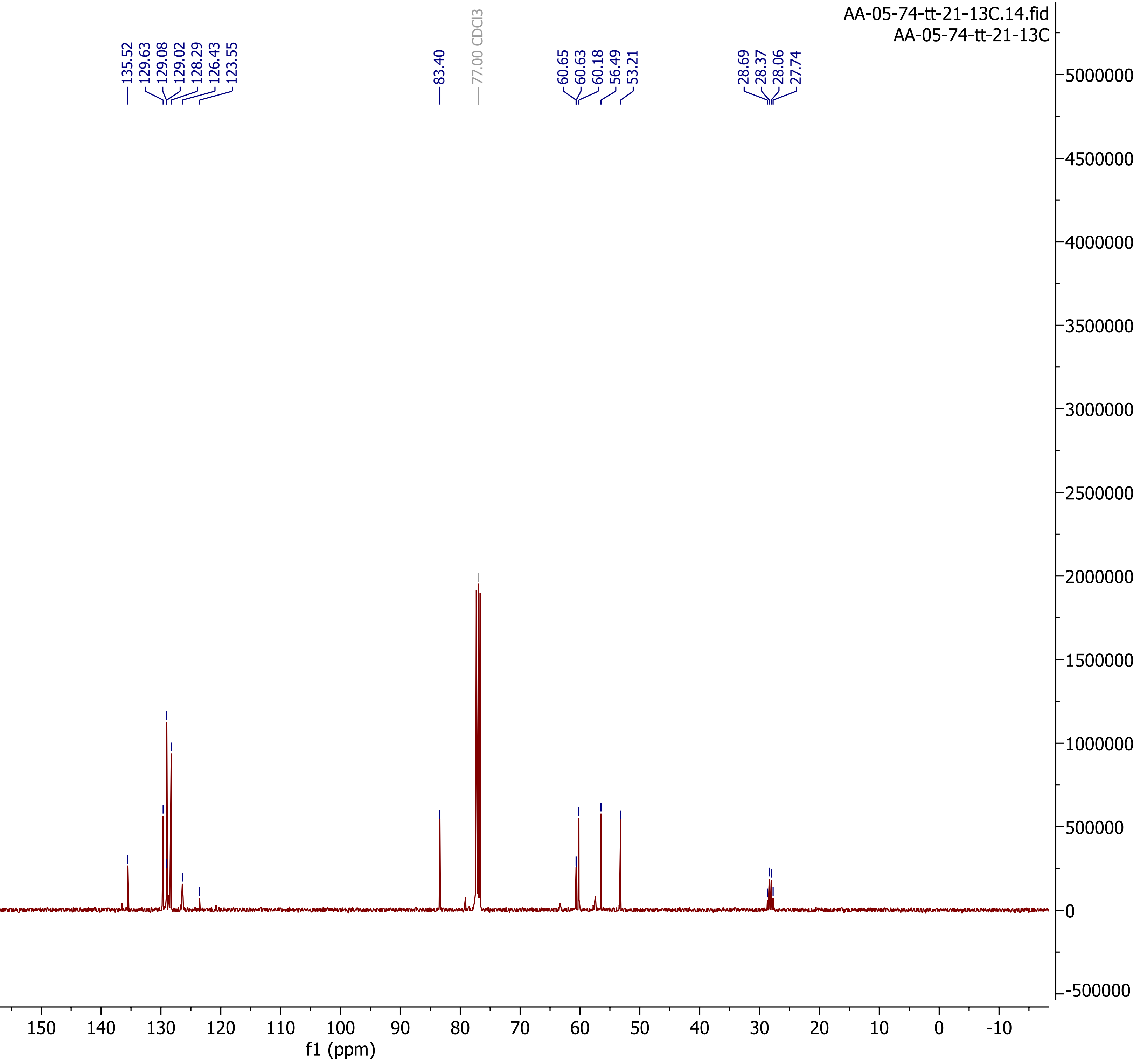

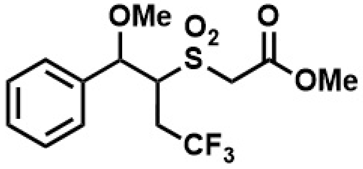

94

Parameter	Value
1 Solvent	CDCl3
2 Acquisition Date	2025-12-07T21:11:44
3 Spectrometer Frequency	376.46
4 Nucleus	19F

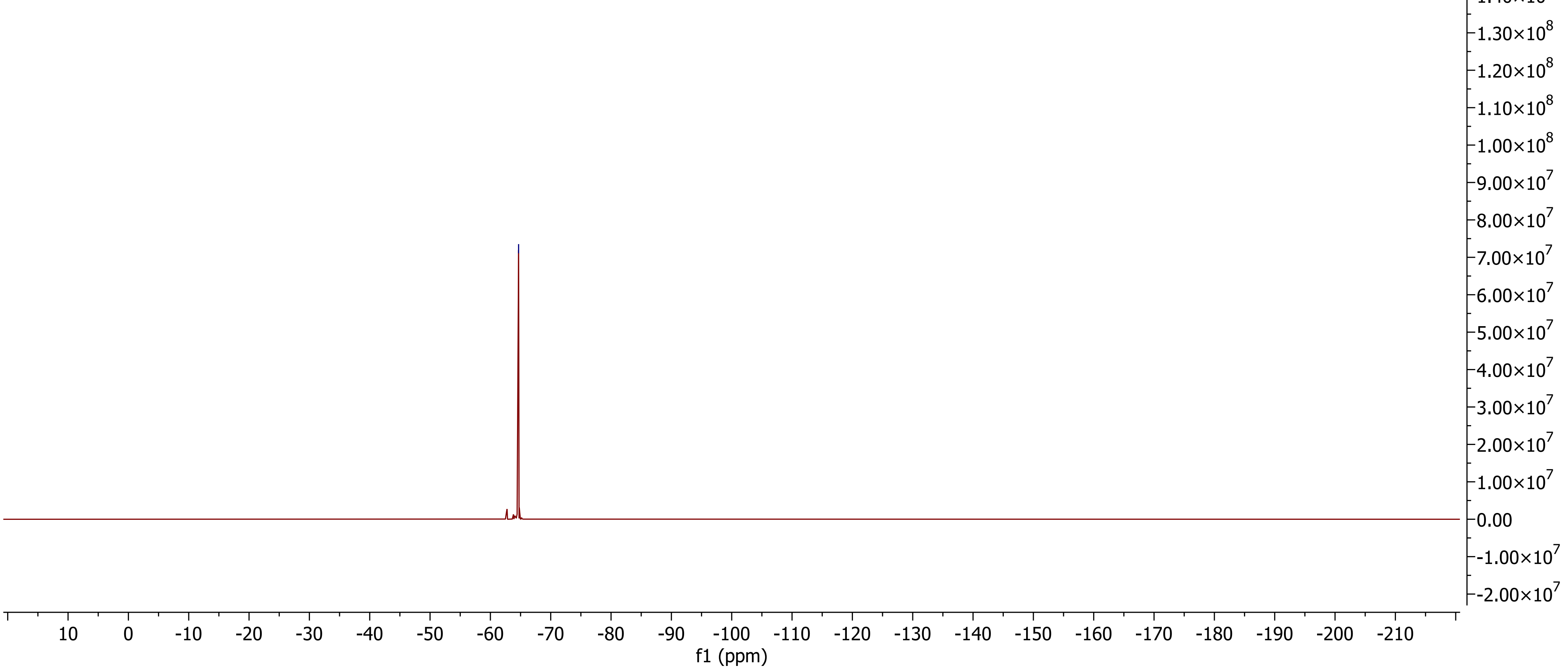

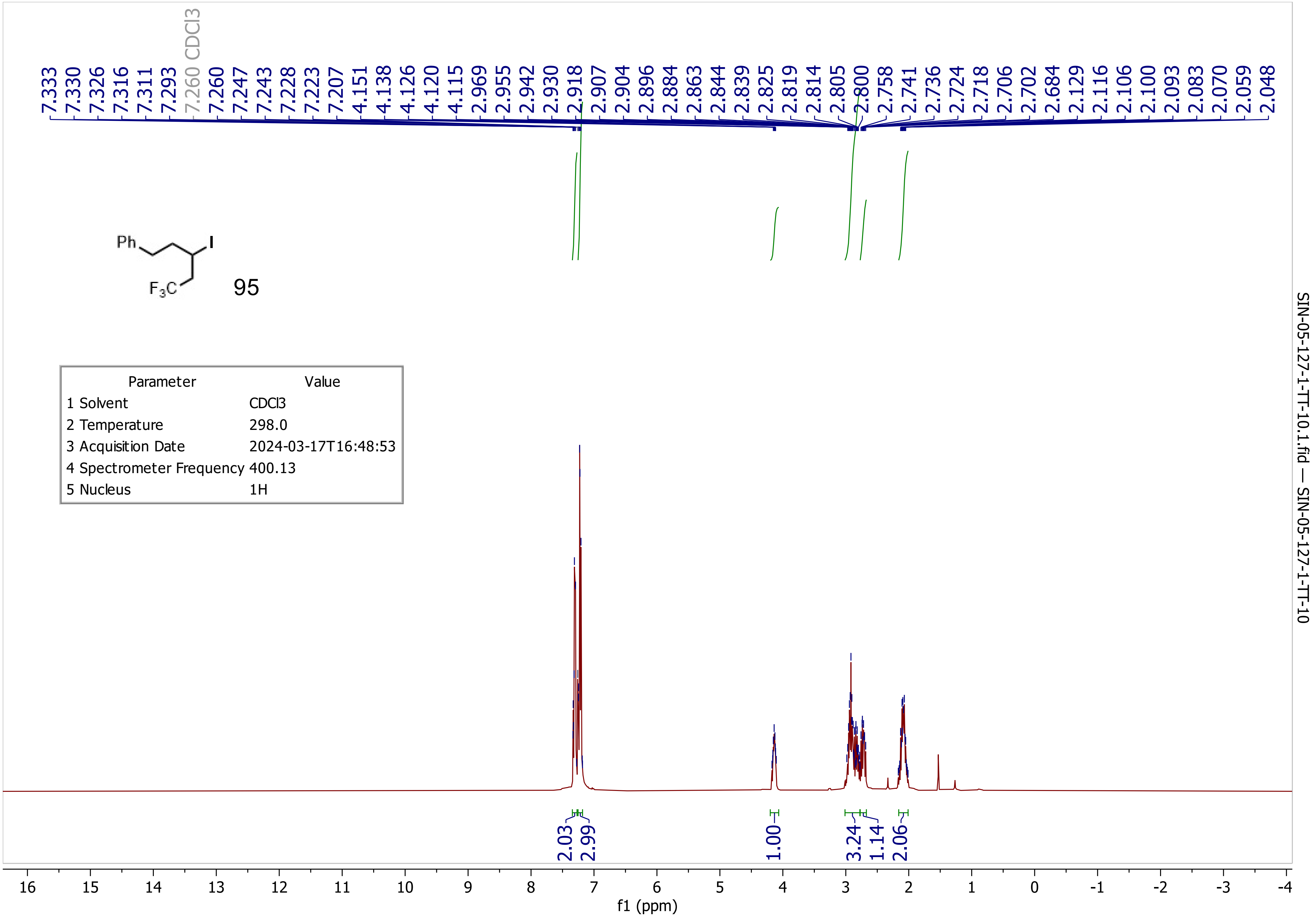
7.333
7.330
7.326
7.316
7.311
7.293
7.260 CDCl3
7.260
7.247
7.243
7.228
7.223
7.207
4.151
4.138
4.126
4.120
4.115
2.969
2.955
2.942
2.930
2.918
2.907
2.904
2.896
2.884
2.863
2.844
2.839
2.825
2.819
2.814
2.805
2.800
2.758
2.741
2.736
2.724
2.718
2.706
2.702
2.684
2.129
2.116
2.106
2.100
2.093
2.083
2.070
2.059
2.048
Ph
I
F3C
95
Parameter
Value
1 Solvent
CDCl3
2 Temperature
298.0
3 Acquisition Date
2024-03-17T16:48:53
4 Spectrometer Frequency
400.13
5 Nucleus
1H
2.03
2.99
1.00
3.24
1.14
2.06
16 15 14 13 12 11 10 9 8 7 6 5 4 3 2 1 0 -1 -2 -3 -4
f1 (ppm)
SIN-05-127-1-TT-10.1.fid — SIN-05-127-1-TT-10

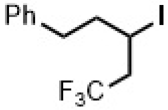

95

Parameter	Value
1 Solvent	CDCl3
2 Temperature	298.0
3 Acquisition Date	2024-03-18T01:15:15
4 Spectrometer Frequency	100.62
5 Nucleus	13C

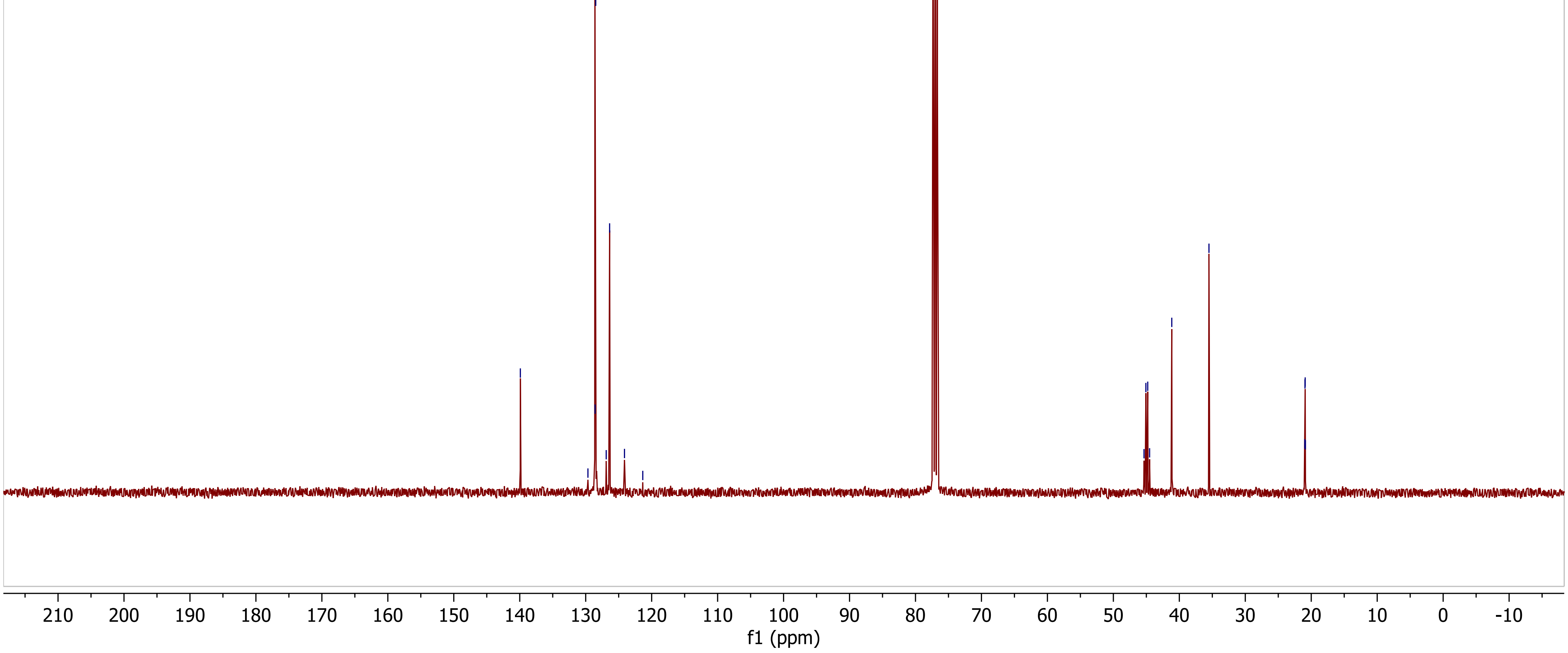

SIN-05-127-1-C13.1.fid — SIN-05-127-1-C13

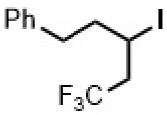

95

Parameter	Value
1 Solvent	CDCl3
2 Temperature	298.0
3 Acquisition Date	2024-03-18T01:19:31
4 Spectrometer Frequency	376.46
5 Nucleus	19F

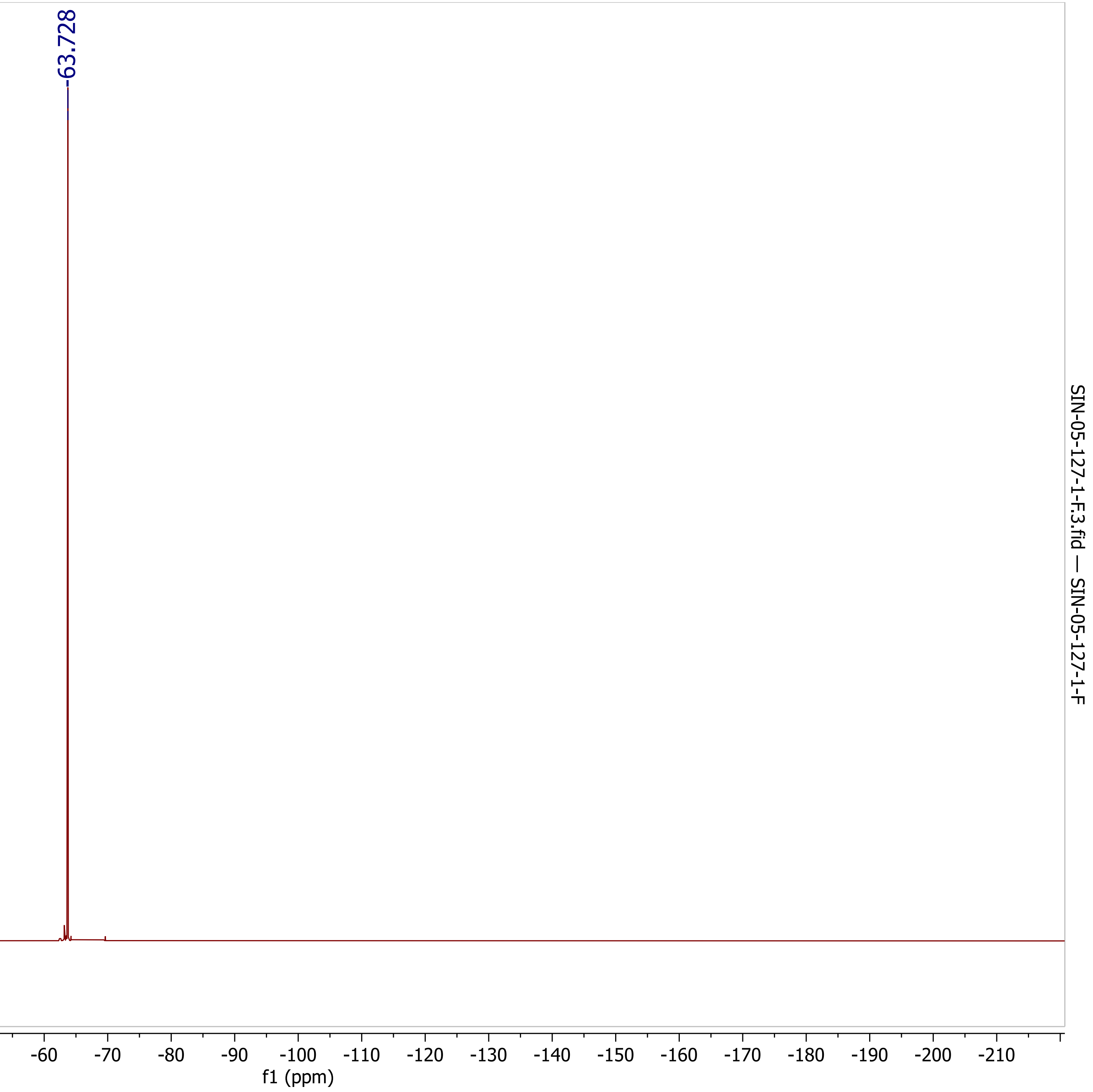

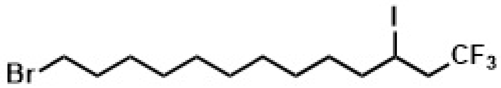

116

Parameter	Value
Solvent	CDCl3
Temperature	298.0
Spectrometer Frequency	400.13
Nucleus	1H

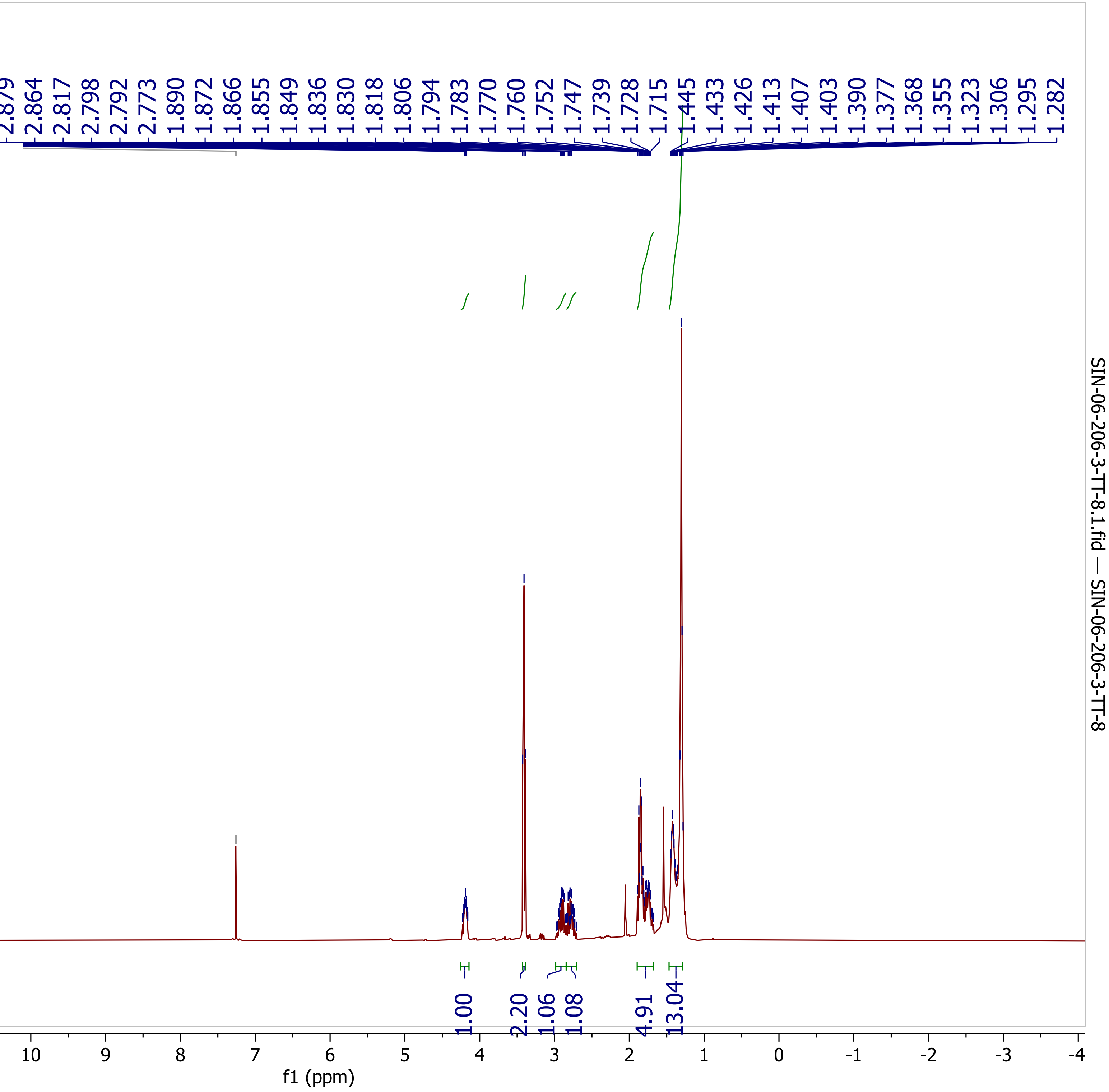

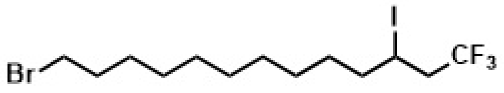

116

Parameter	Value
Solvent	CDCl3
Temperature	298.0
Spectrometer Frequency	100.62
Nucleus	13C

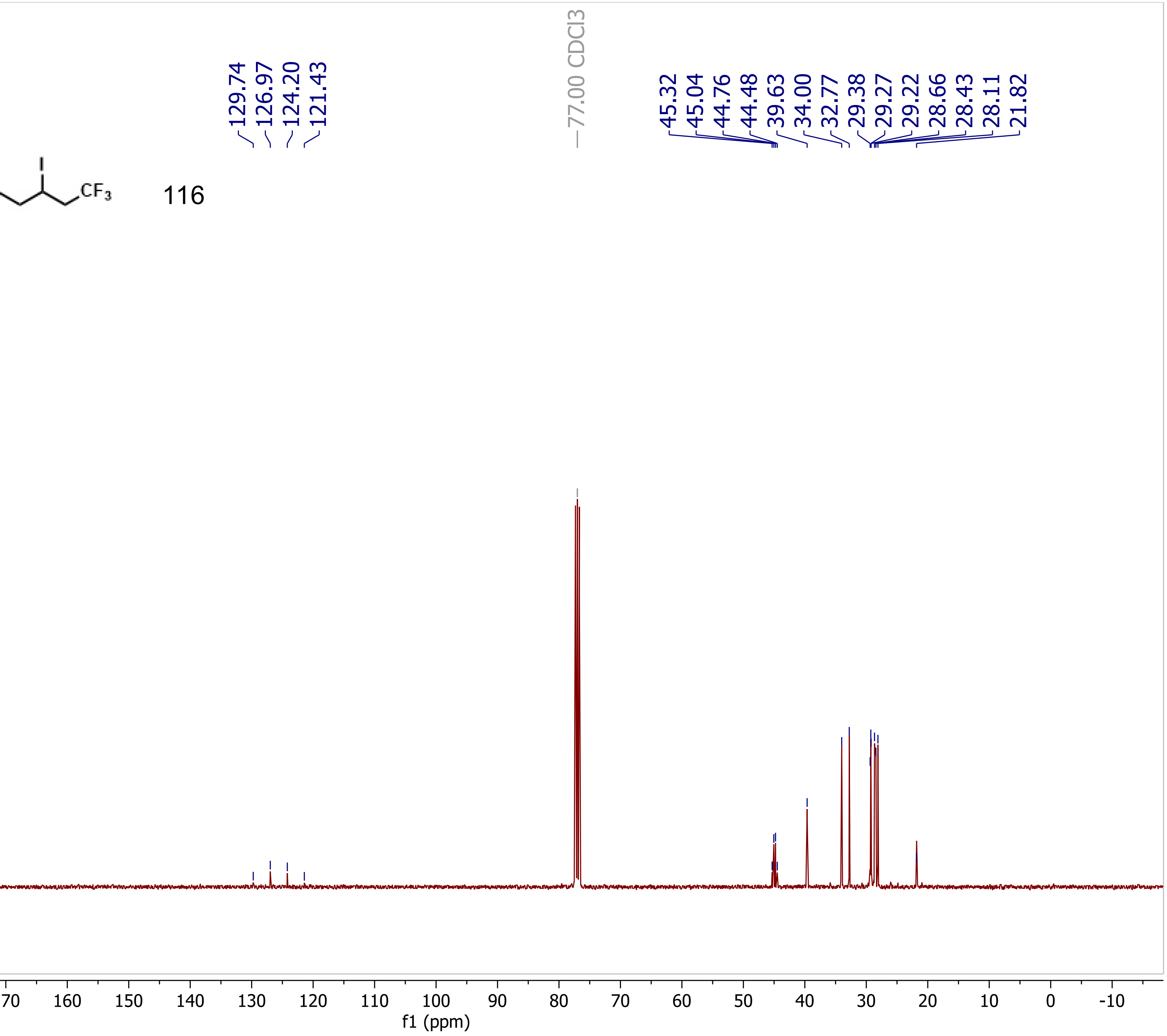

SIN-06-206-3-TT-8-C13.5.fid — SIN-06-206-3-TT-8-C13

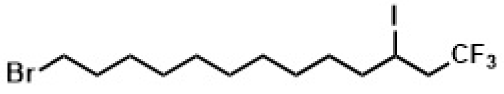

116

Parameter	Value
Solvent	CDCl3
Temperature	298.0
Spectrometer Frequency	376.46
Nucleus	19F

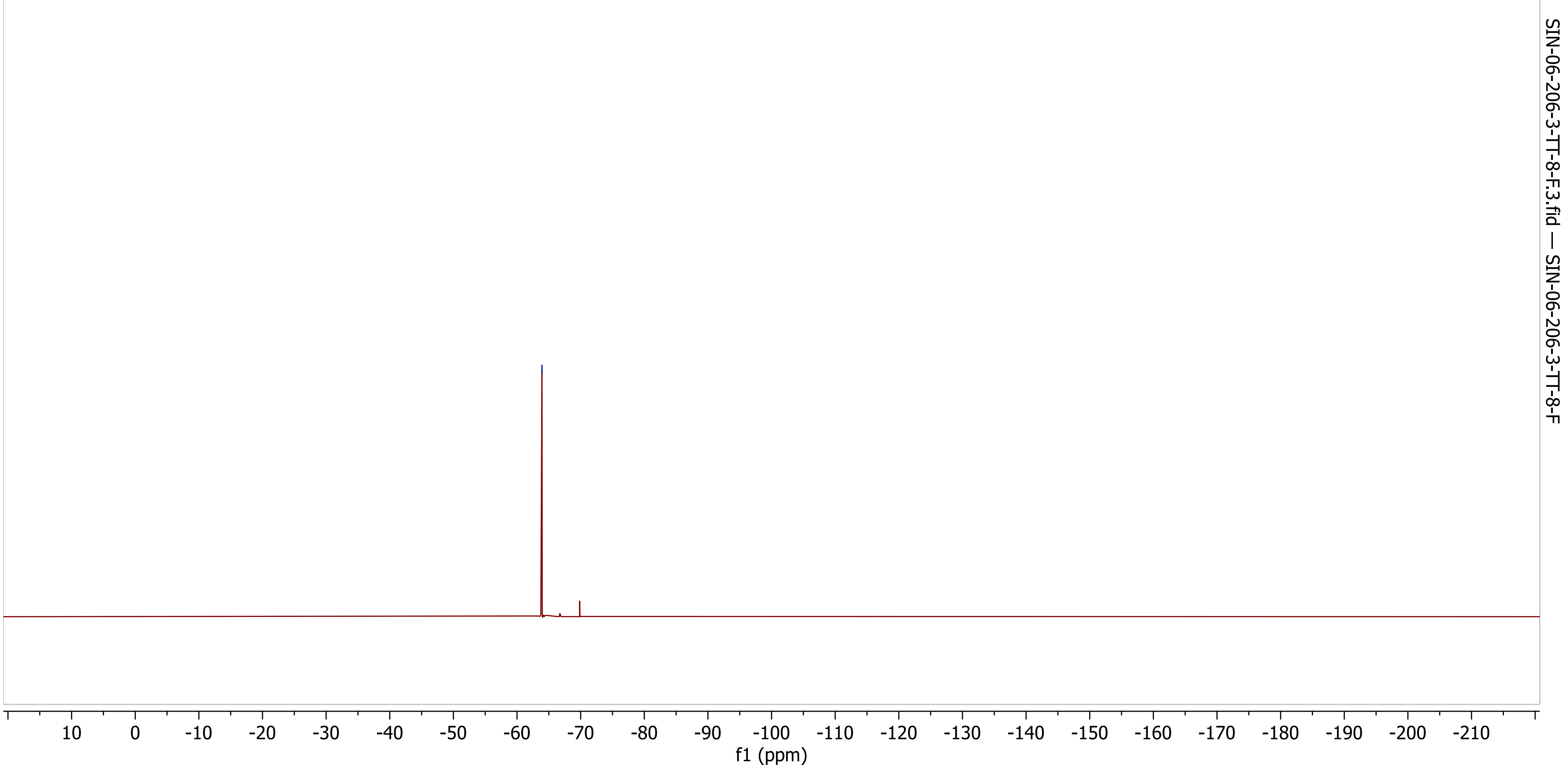

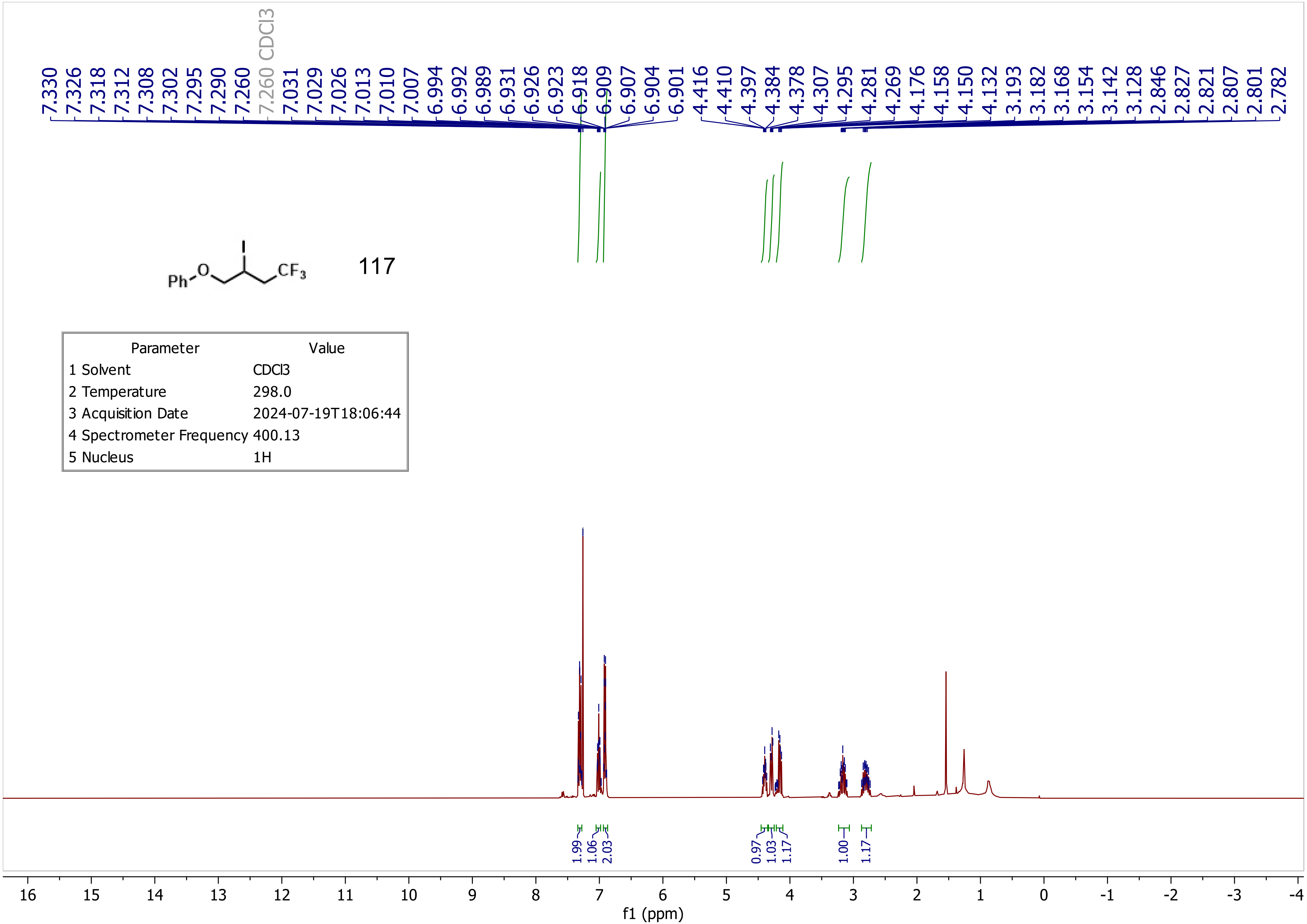

117

Parameter	Value
1 Solvent	CDCl3
2 Temperature	298.0
3 Acquisition Date	2024-07-19T18:06:44
4 Spectrometer Frequency	400.13
5 Nucleus	1H

SIN-06-31-1-TT-2.1.fid — SIN-06-31-1-TT-2

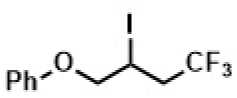

117

Parameter	Value
1 Solvent	CDCl3
2 Acquisition Date	2024-07-20T02:11:38
3 Spectrometer Frequency	100.62
4 Nucleus	(13C,)

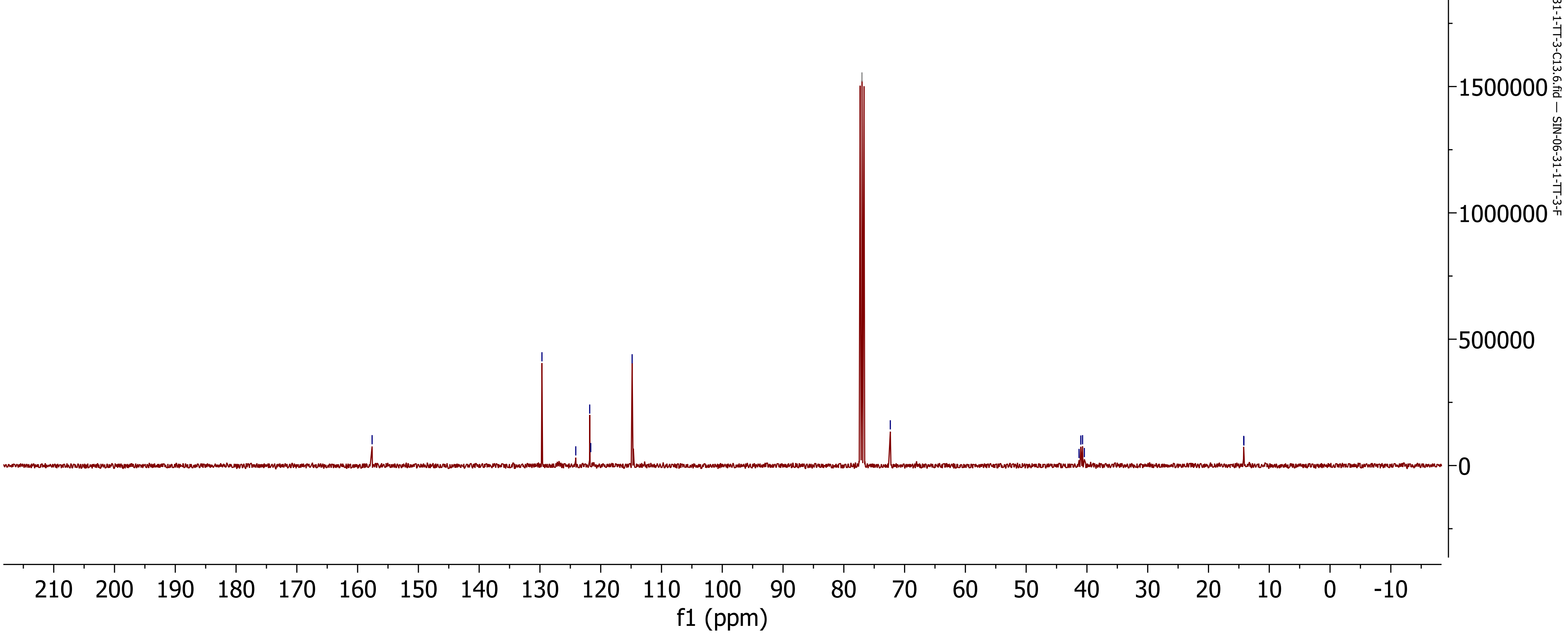

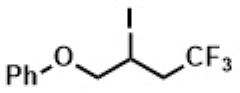

117

Parameter	Value
1 Solvent	CDCl3
2 Acquisition Date	2024-07-19T18:14:07
3 Spectrometer Frequency	376.46
4 Nucleus	(19F,)

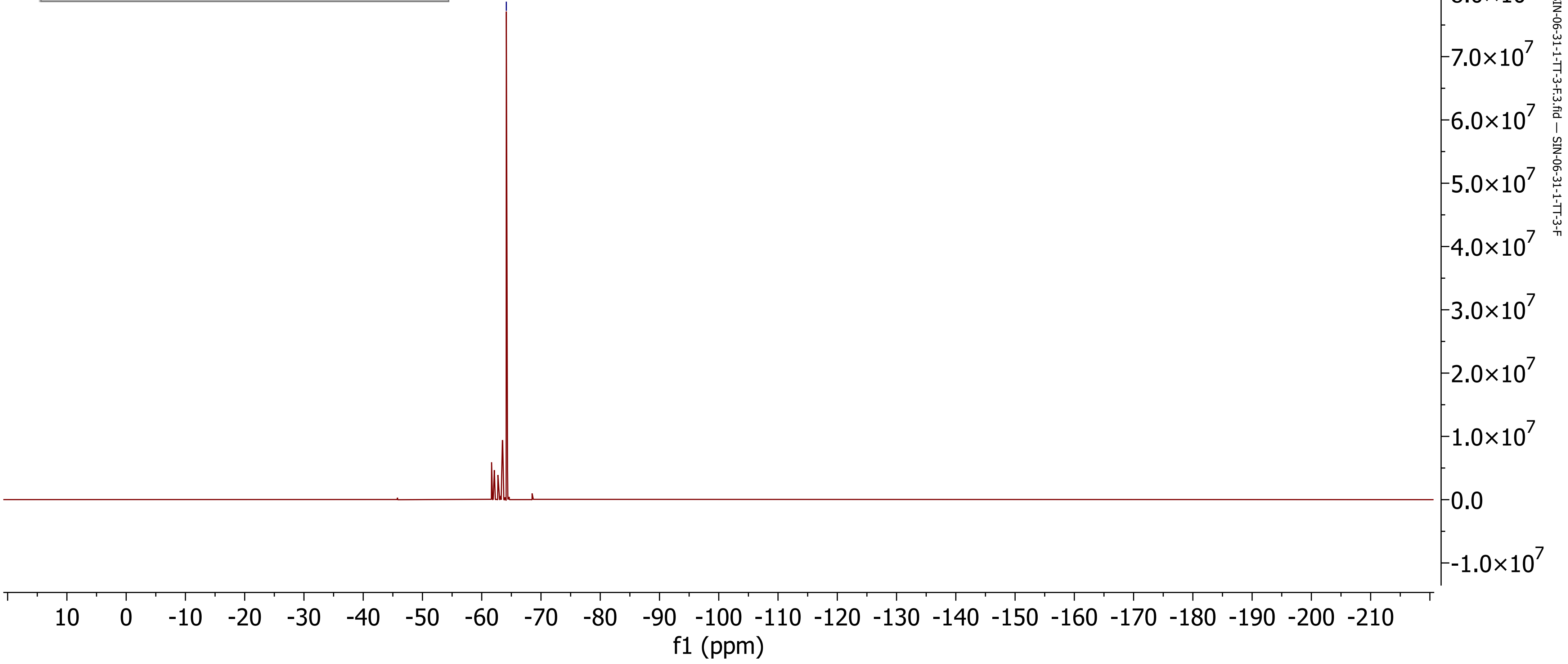

118

Parameter	Value
1 Solvent	CDCl3
2 Acquisition Date	2025-07-10T02:22:55
3 Spectrometer Frequency	400.14
4 Nucleus	(1H,)

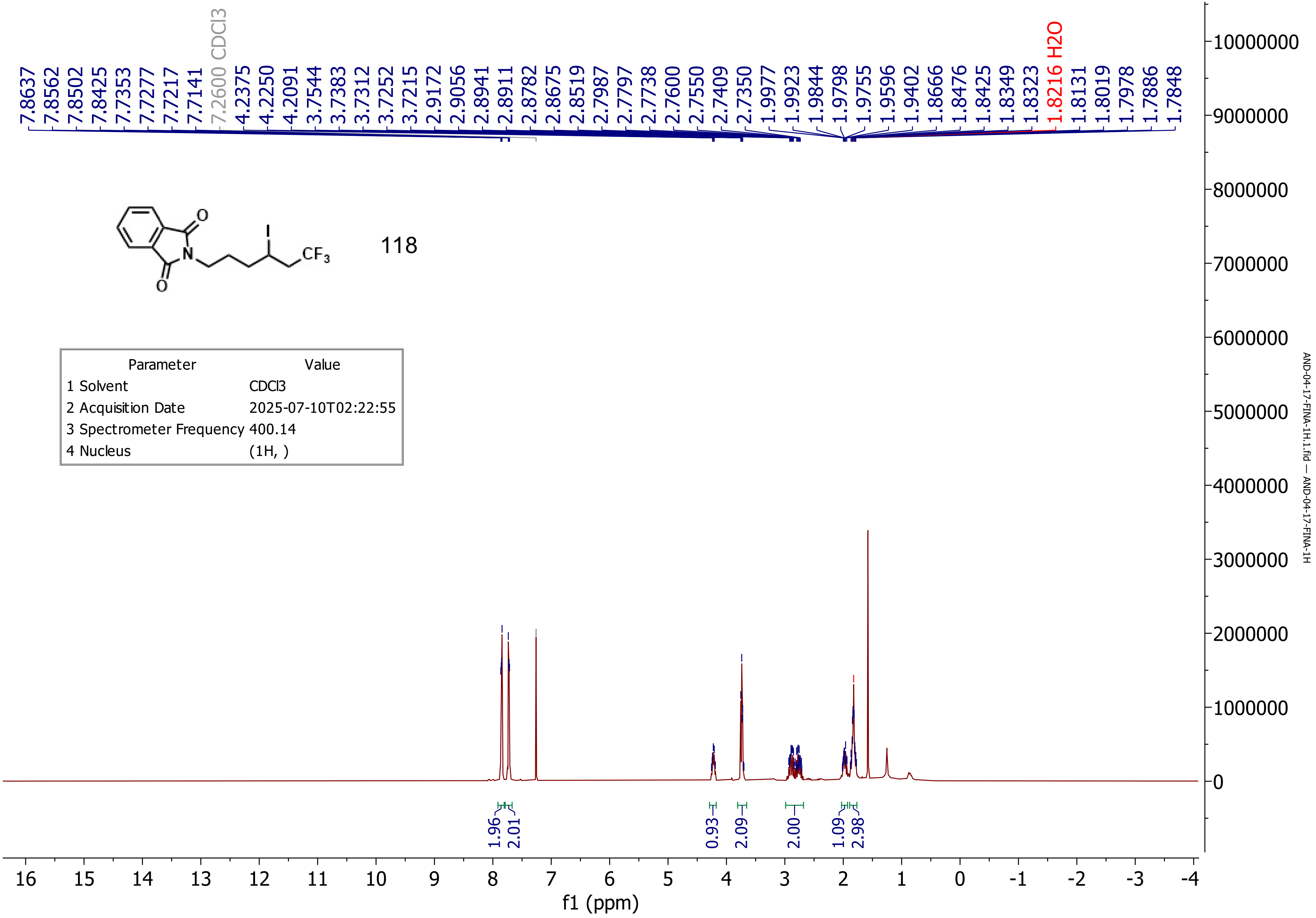

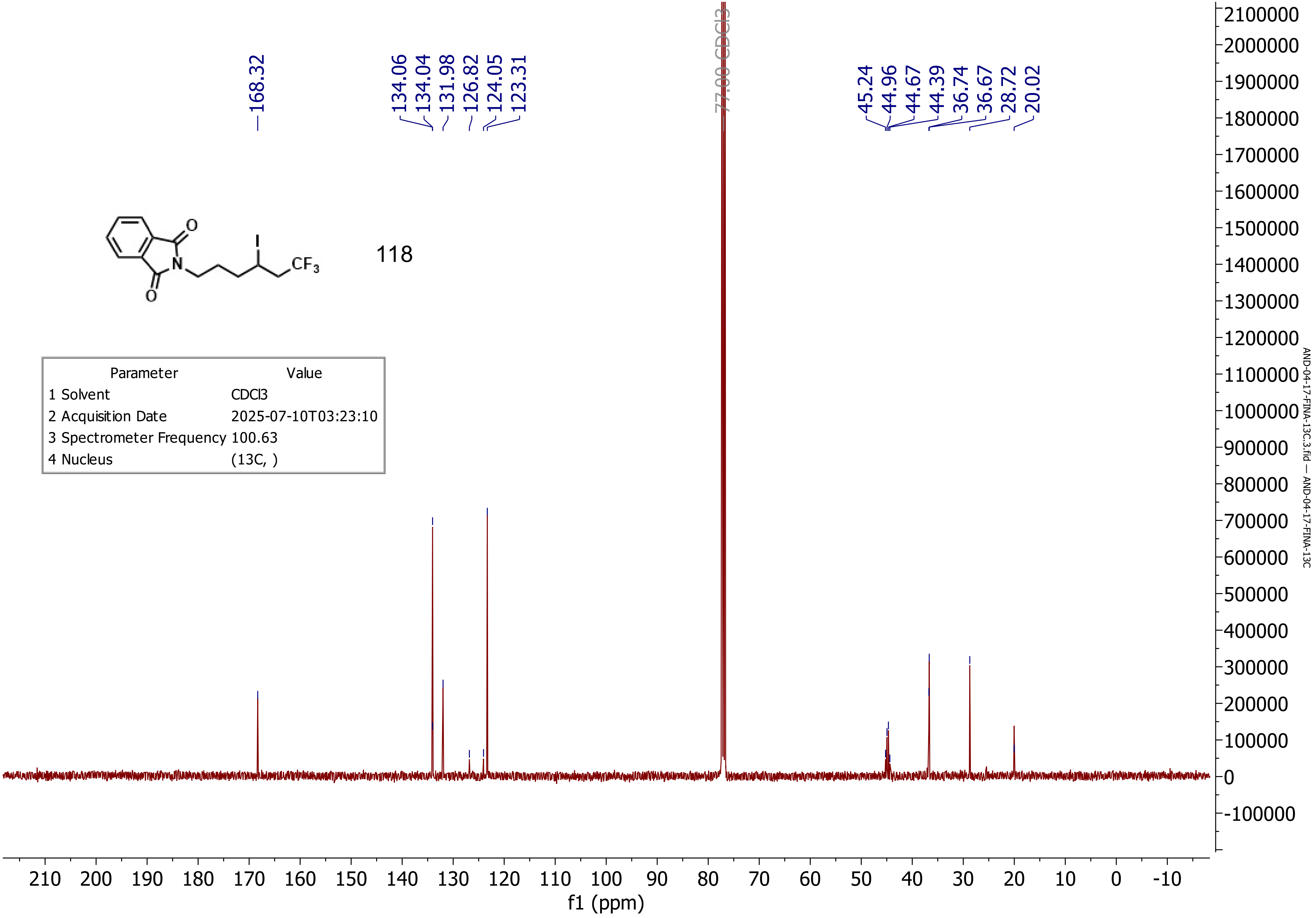

118

Parameter	Value
1 Solvent	CDCl3
2 Acquisition Date	2025-07-10T03:23:10
3 Spectrometer Frequency	100.63
4 Nucleus	(13C,)

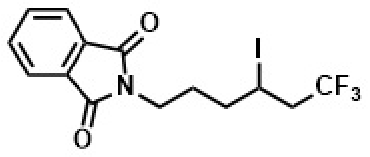

118

Parameter	Value
1 Solvent	CDCl3
2 Acquisition Date	2025-07-10T03:25:01
3 Spectrometer Frequency	376.47
4 Nucleus	(19F,)

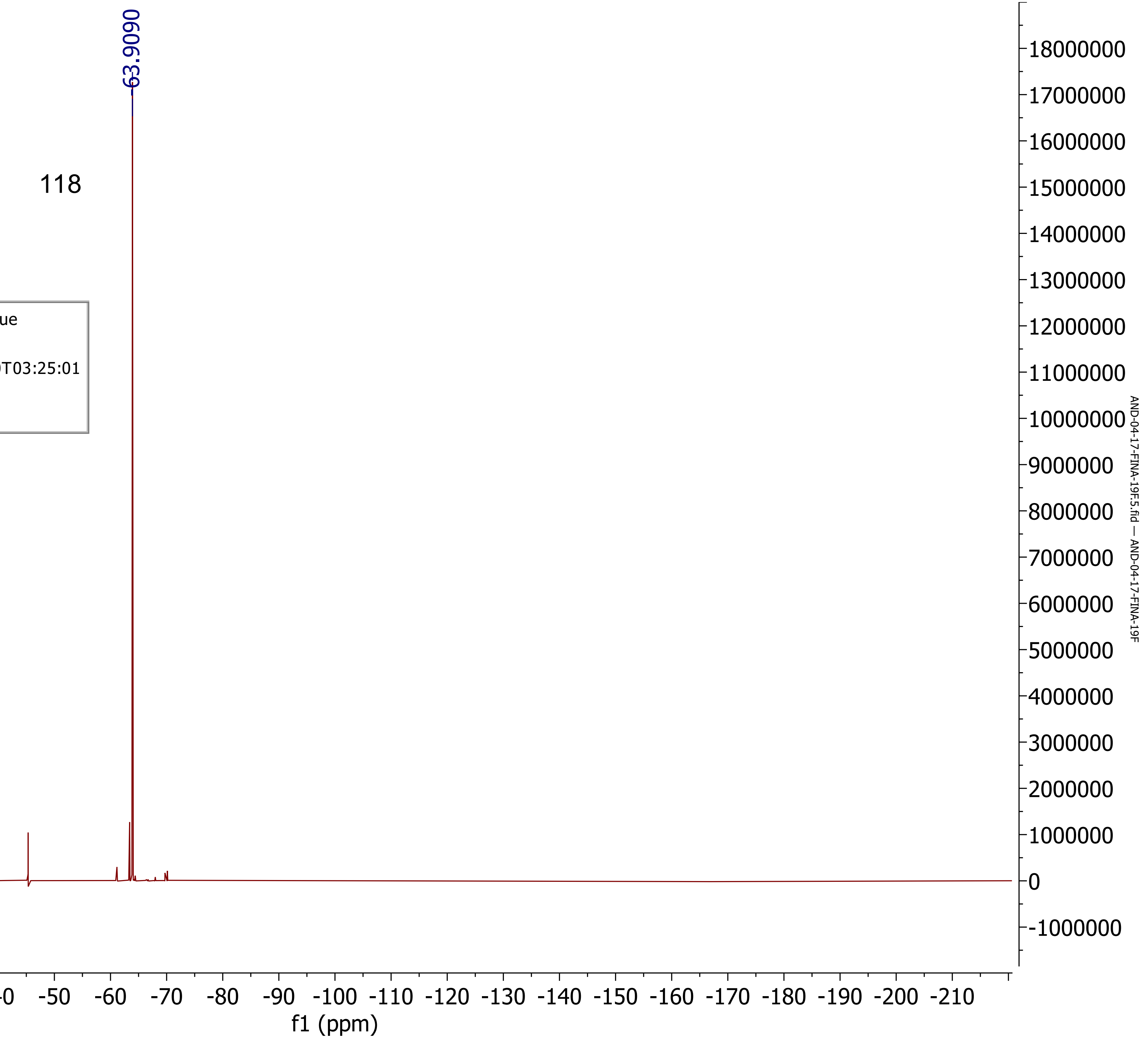

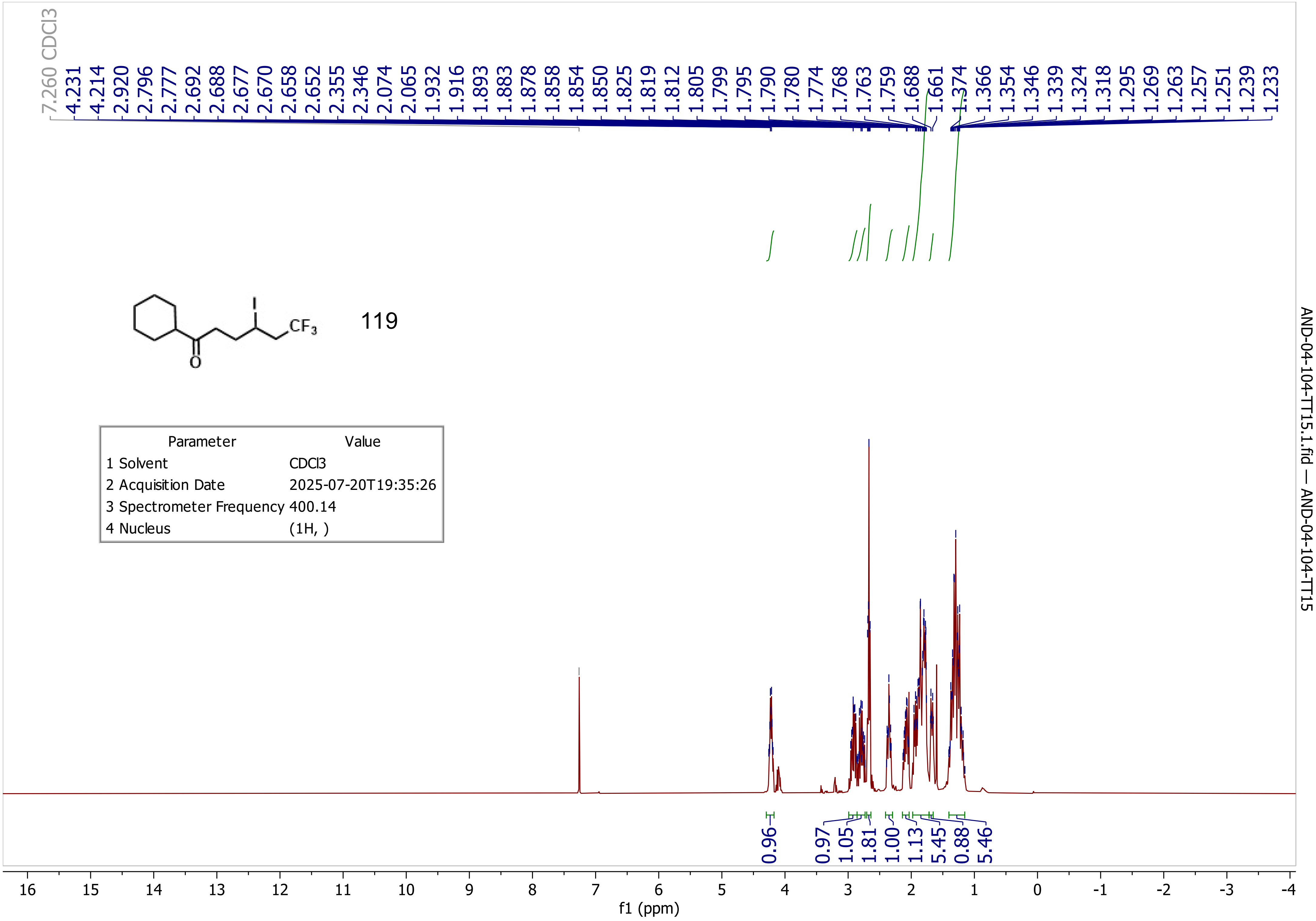

119

Parameter	Value
1 Solvent	CDCl3
2 Acquisition Date	2025-07-20T19:35:26
3 Spectrometer Frequency	400.14
4 Nucleus	(1H,)

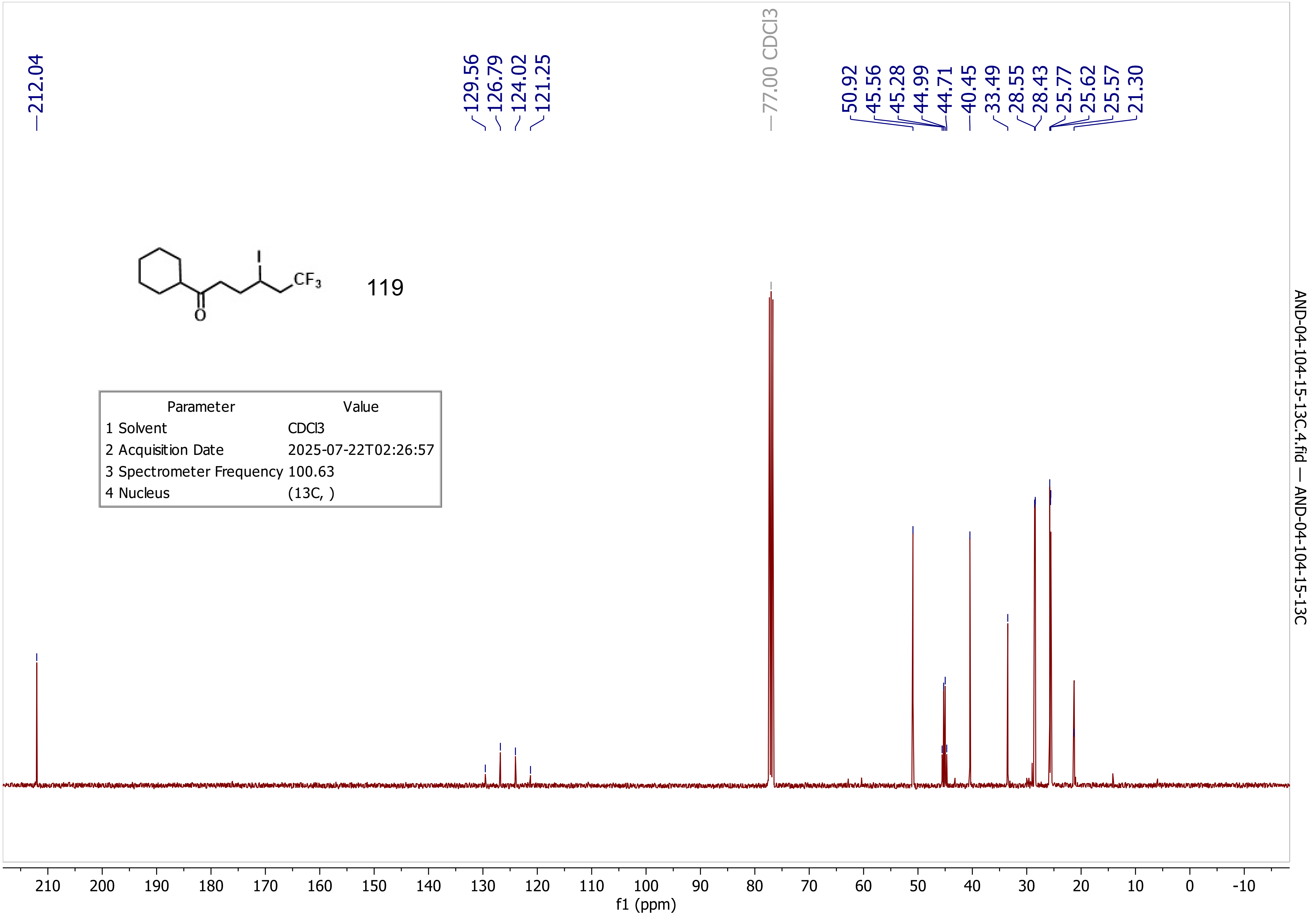

212.04
129.56
126.79
124.02
121.25
77.00 CDCl3
50.92
45.56
45.28
44.99
44.71
40.45
33.49
28.55
28.43
25.77
25.62
25.57
21.30
CF3
O
I
119
Parameter Value
1 Solvent CDCl3
2 Acquisition Date 2025-07-22T02:26:57
3 Spectrometer Frequency 100.63
4 Nucleus (13C,)
210 200 190 180 170 160 150 140 130 120 110 100 90 80 70 60 50 40 30 20 10 0 -10
f1 (ppm)
AND-04-104-15-13C.4.fid — AND-04-104-15-13C

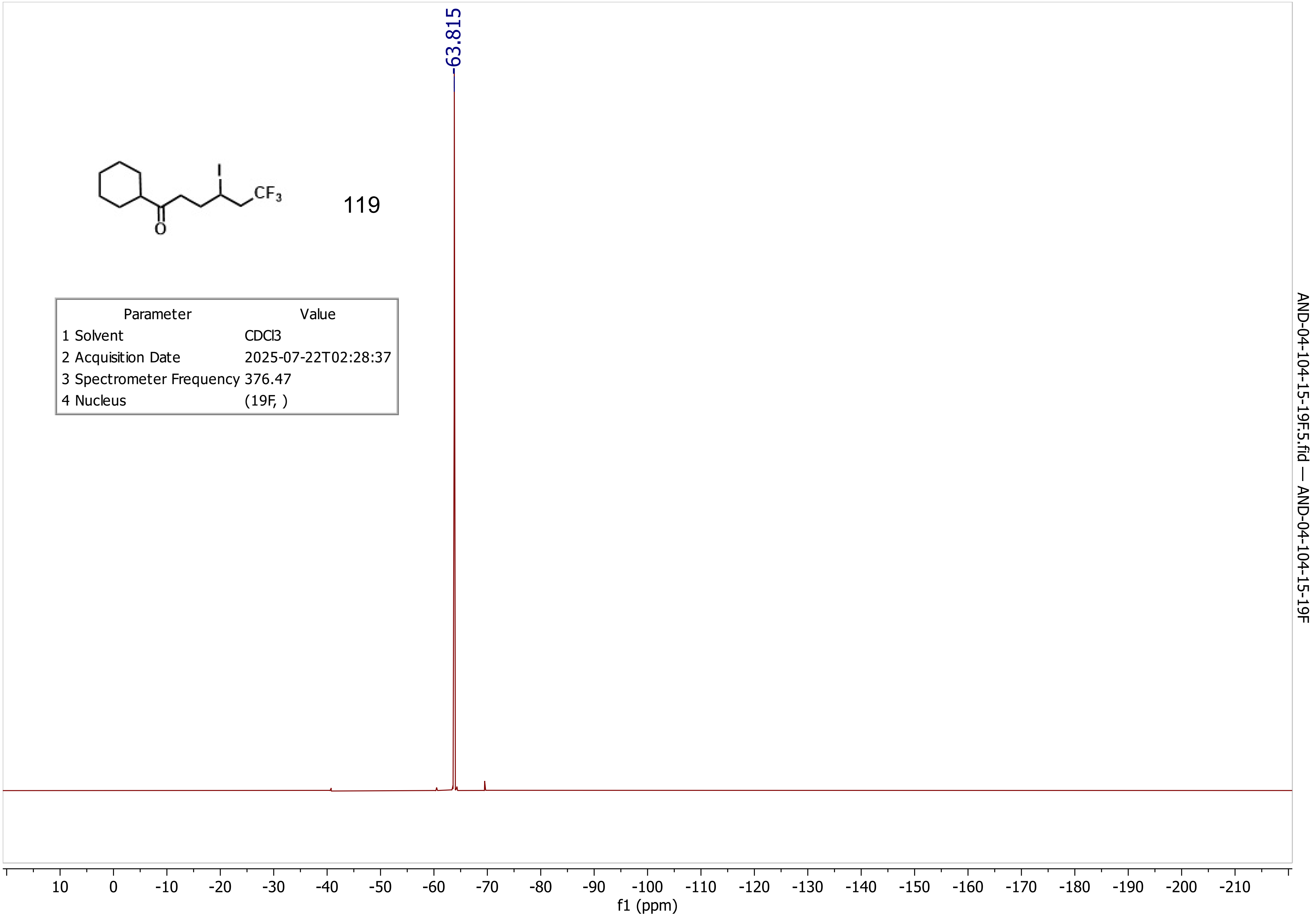

	Parameter	Value
1	Solvent	CDCl3
2	Acquisition Date	2025-07-22T02:28:37
3	Spectrometer Frequency	376.47
4	Nucleus	(19F,)

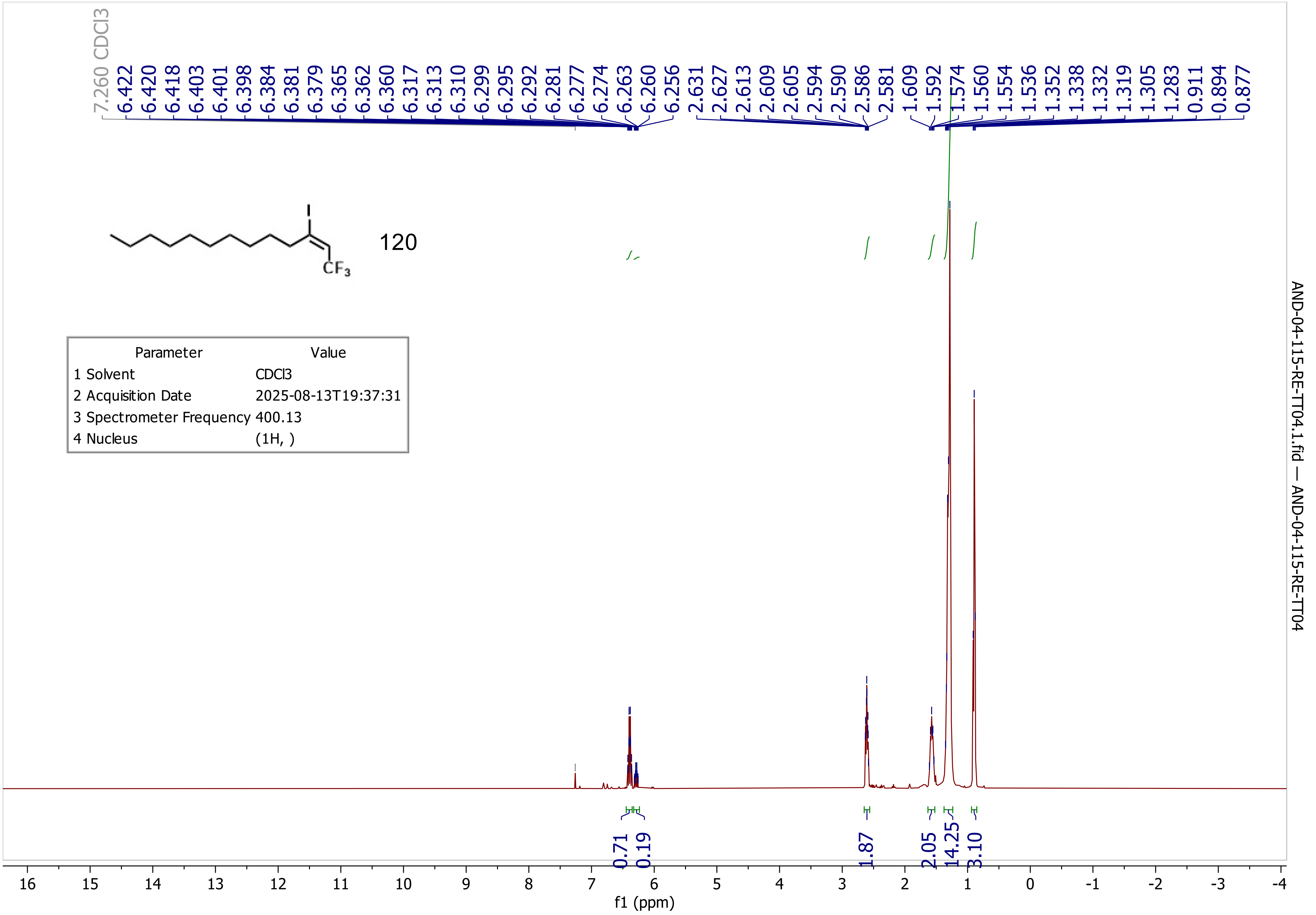

Parameter	Value
1 Solvent	CDCl3
2 Acquisition Date	2025-08-13T19:37:31
3 Spectrometer Frequency	400.13
4 Nucleus	(1H,)

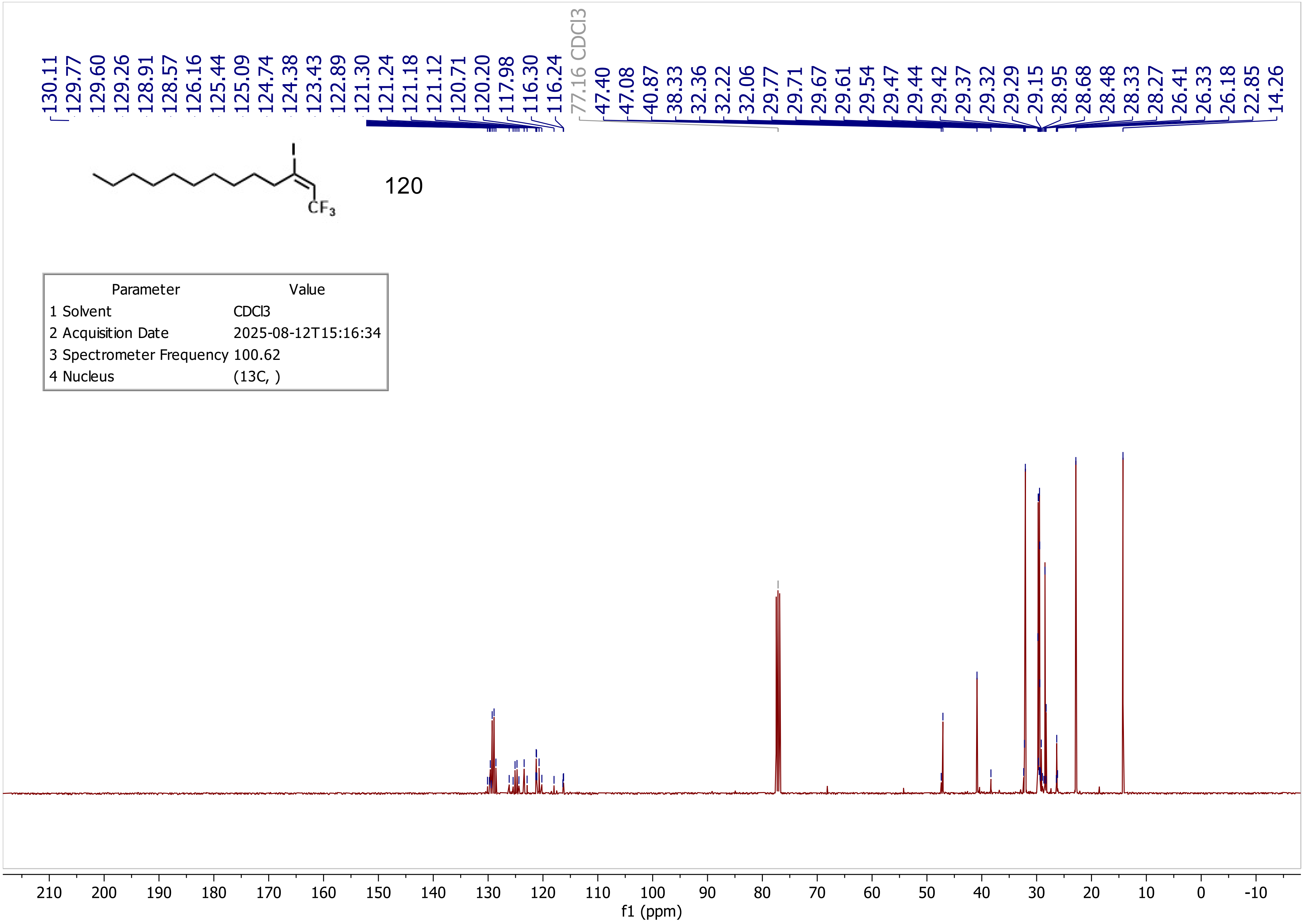

120

Parameter	Value
1 Solvent	CDCl3
2 Acquisition Date	2025-08-12T15:16:34
3 Spectrometer Frequency	100.62
4 Nucleus	(13C,)

AND-04-115-RE-PURE-13C.3.fid — AND-04-136-2ND-DMSO-13C

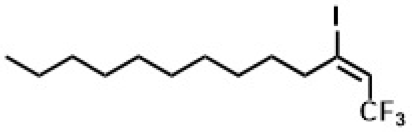

120

Parameter	Value
1 Solvent	CDCl3
2 Acquisition Date	2025-08-13T19:42:05
3 Spectrometer Frequency	376.46
4 Nucleus	(19F,)

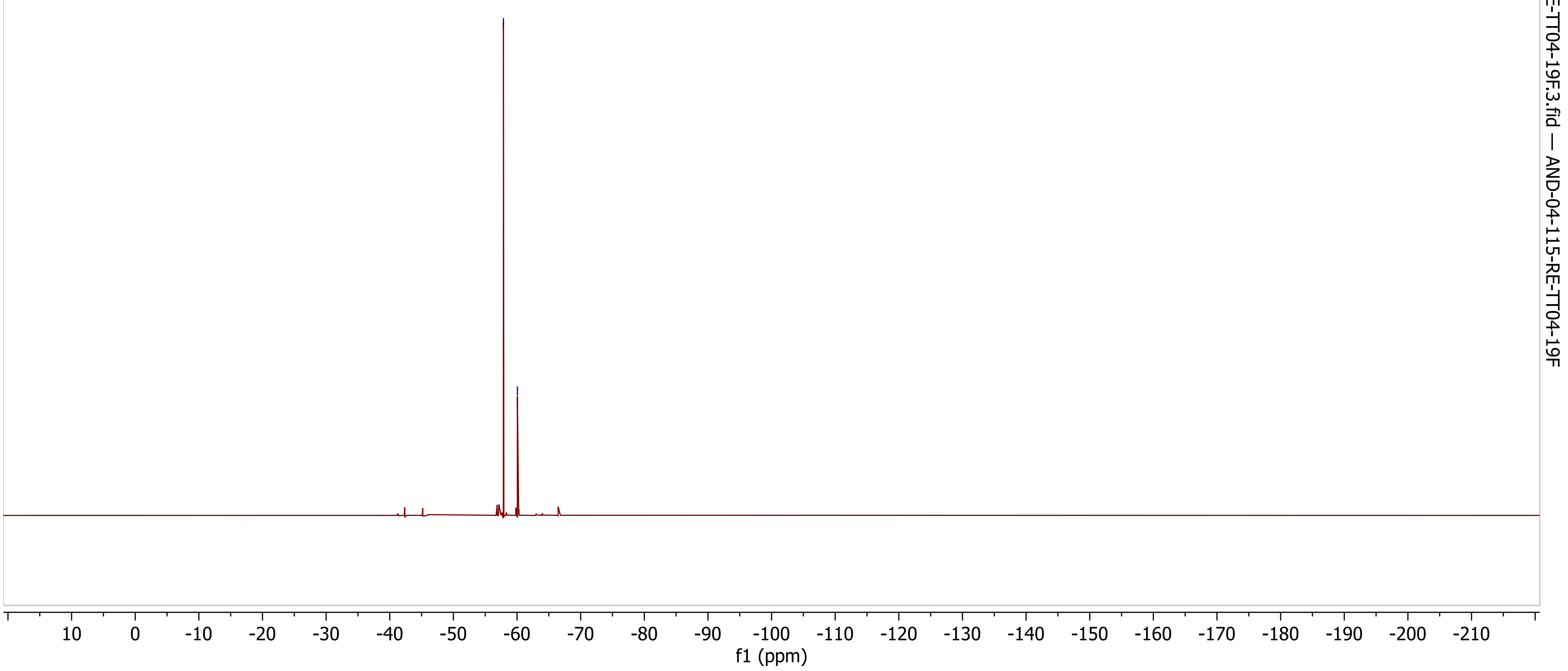

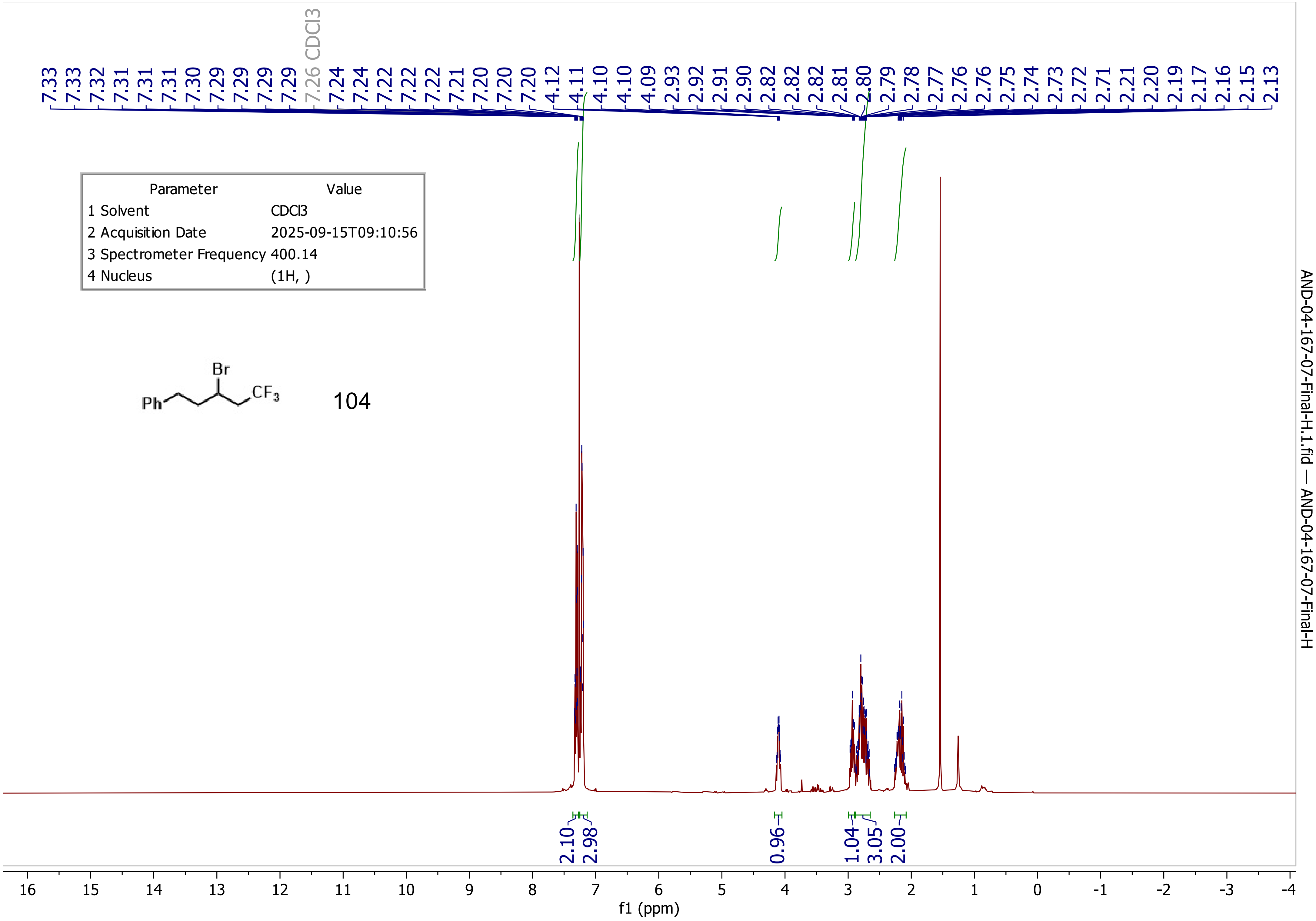

7.33 7.33 7.32 7.31 7.31 7.31 7.30 7.29 7.29 7.29 7.29 7.26 CDCl3 7.24 7.24 7.22 7.22 7.22 7.21 7.20 7.20 7.20 4.12 4.11 4.10 4.10 4.09 2.93 2.92 2.91 2.90 2.82 2.82 2.82 2.81 2.80 2.79 2.78 2.77 2.76 2.76 2.75 2.74 2.73 2.72 2.71 2.21 2.20 2.19 2.17 2.16 2.15 2.13
Parameter Value
1 Solvent CDCl3
2 Acquisition Date 2025-09-15T09:10:56
3 Spectrometer Frequency 400.14
4 Nucleus (1H,)
Br
Ph
CF3
104
2.10 2.98 0.96 1.04 3.05 2.00
16 15 14 13 12 11 10 9 8 7 6 5 4 3 2 1 0 -1 -2 -3 -4
f1 (ppm)
AND-04-167-07-Final-H.1.fid — AND-04-167-07-Final-H

Parameter	Value
1 Solvent	CDCl3
2 Acquisition Date	2025-09-15T13:30:41
3 Spectrometer Frequency	100.62
4 Nucleus	(13C,)

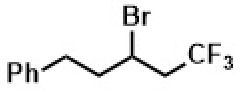

104

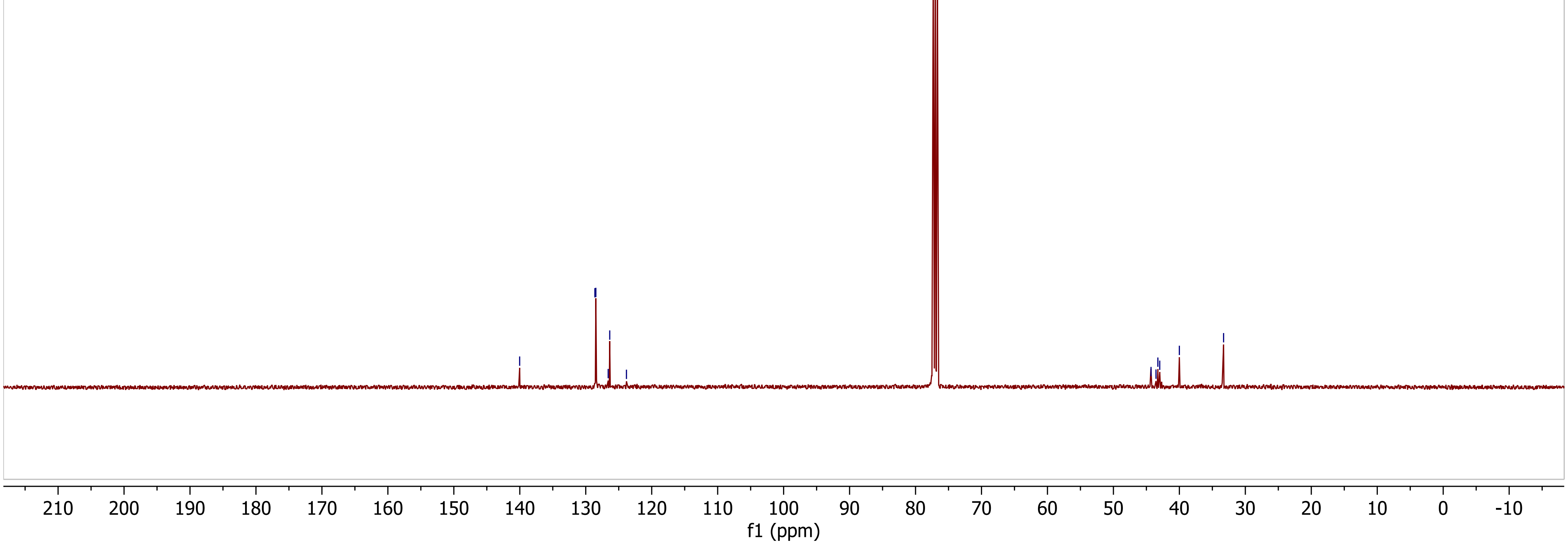

MZVXI$u7QoWJcEt5snZGiw.3.fid — AND-04-167-07-13C-FINAL-

Parameter	Value
1 Solvent	CDCl3
2 Acquisition Date	2025-09-15T09:15:45
3 Spectrometer Frequency	376.47
4 Nucleus	(19F,)

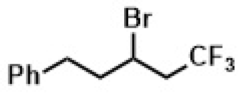

104

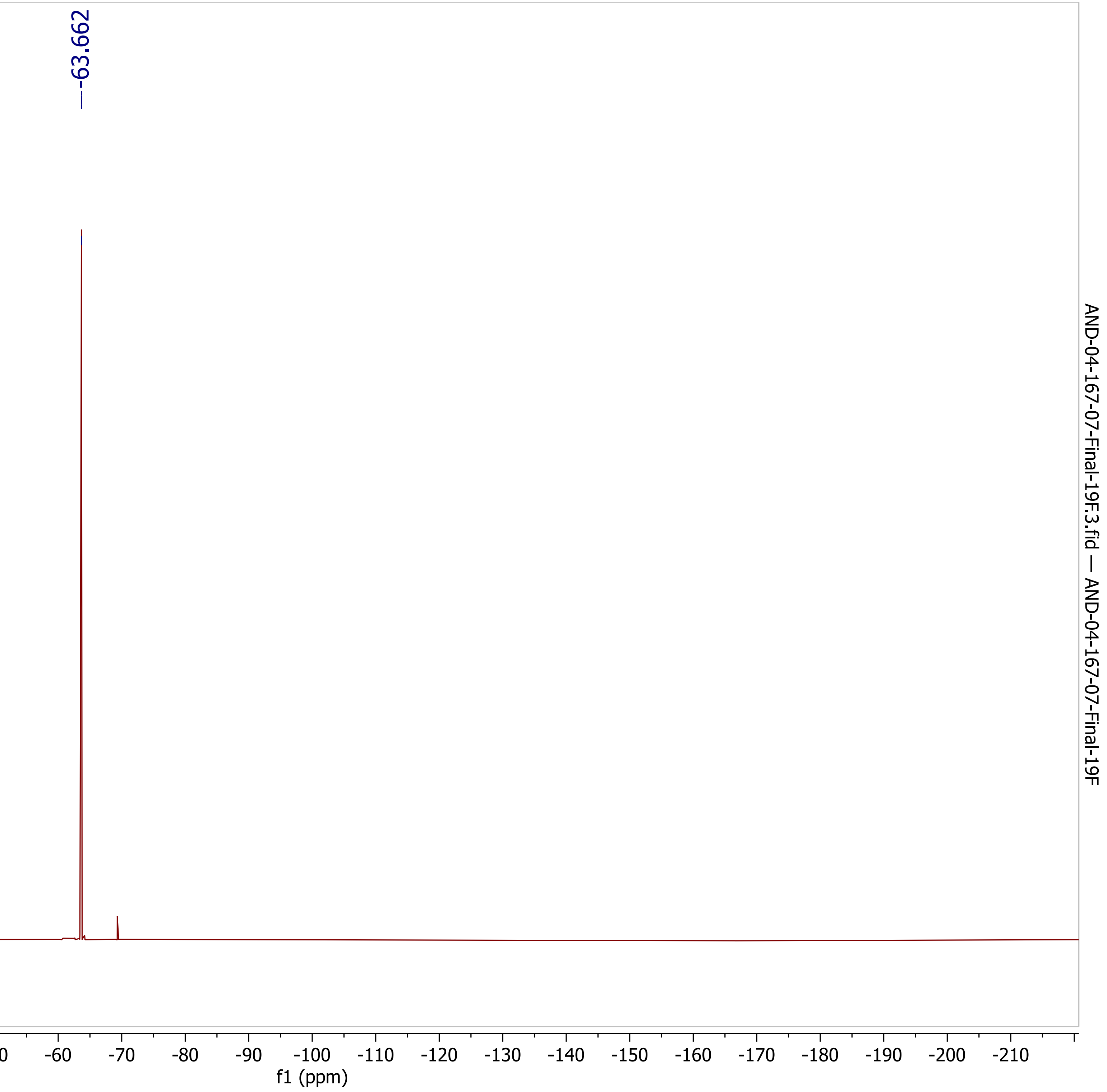

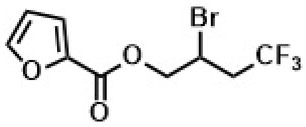

121

Parameter	Value
1 Solvent	CDCl3
2 Acquisition Date	2025-07-17T03:31:25
3 Spectrometer Frequency	400.14
4 Nucleus	(1H,)

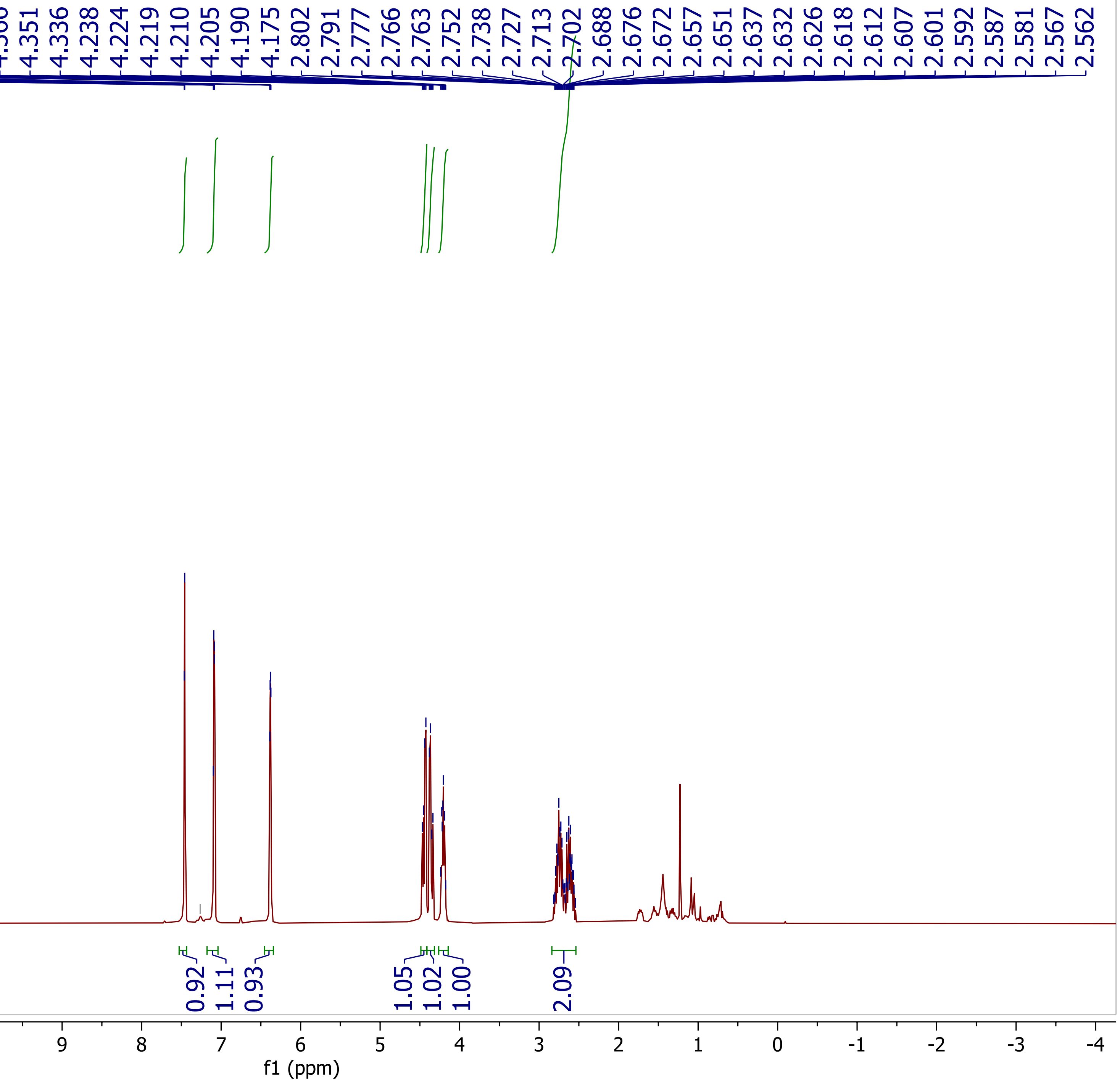

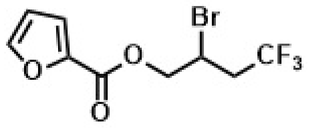

121

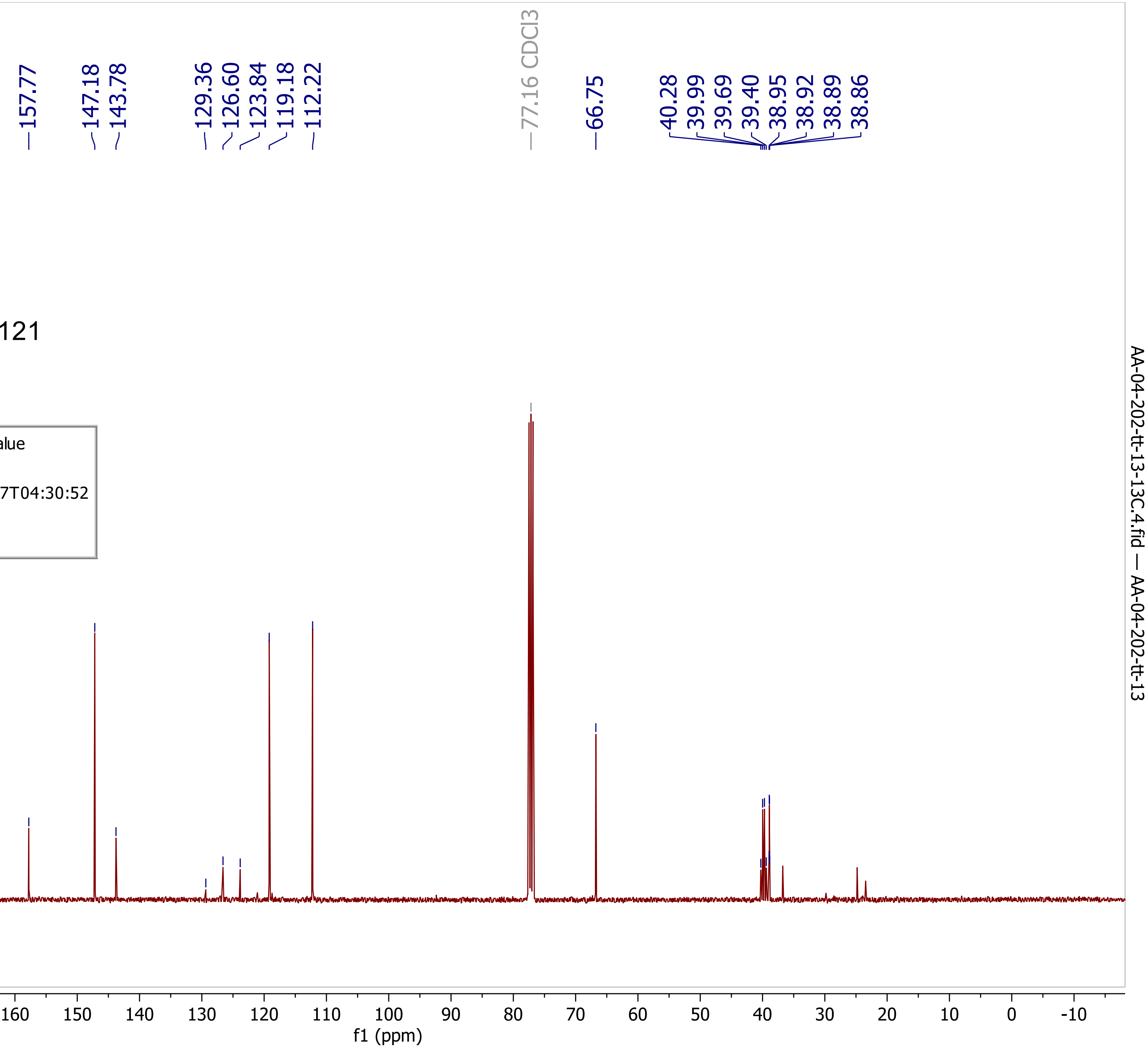

Parameter	Value
1 Solvent	CDCl3
2 Acquisition Date	2025-07-17T04:30:52
3 Spectrometer Frequency	100.63
4 Nucleus	(13C,)

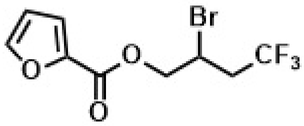

121

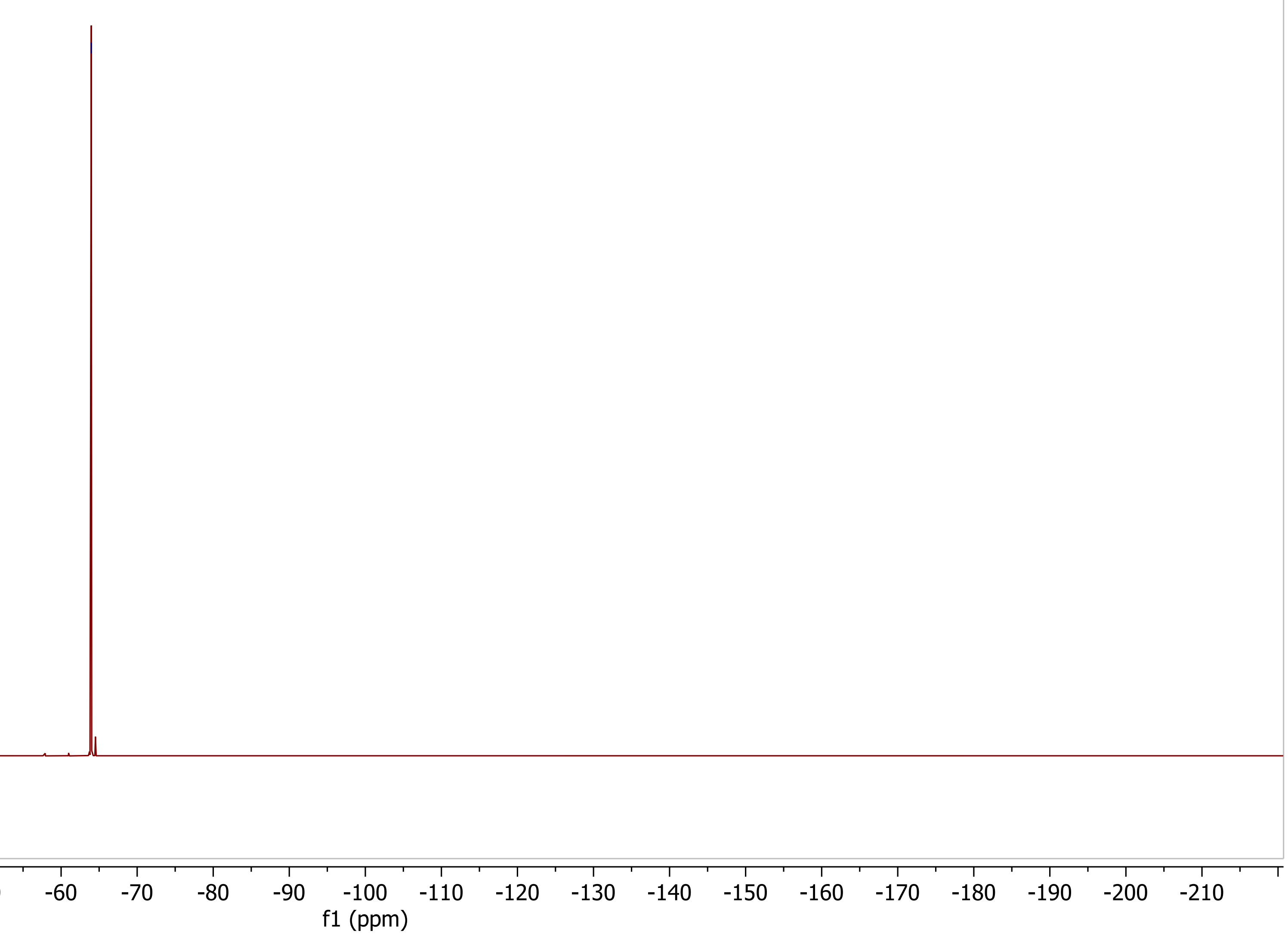

Parameter	Value
1 Solvent	CDCl3
2 Acquisition Date	2025-07-17T04:33:00
3 Spectrometer Frequency	376.47
4 Nucleus	(19F,)

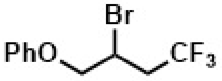

122

Parameter	Value
1 Solvent	CDCl3
2 Acquisition Date	2025-07-17T04:39:03
3 Spectrometer Frequency	400.14
4 Nucleus	(1H,)

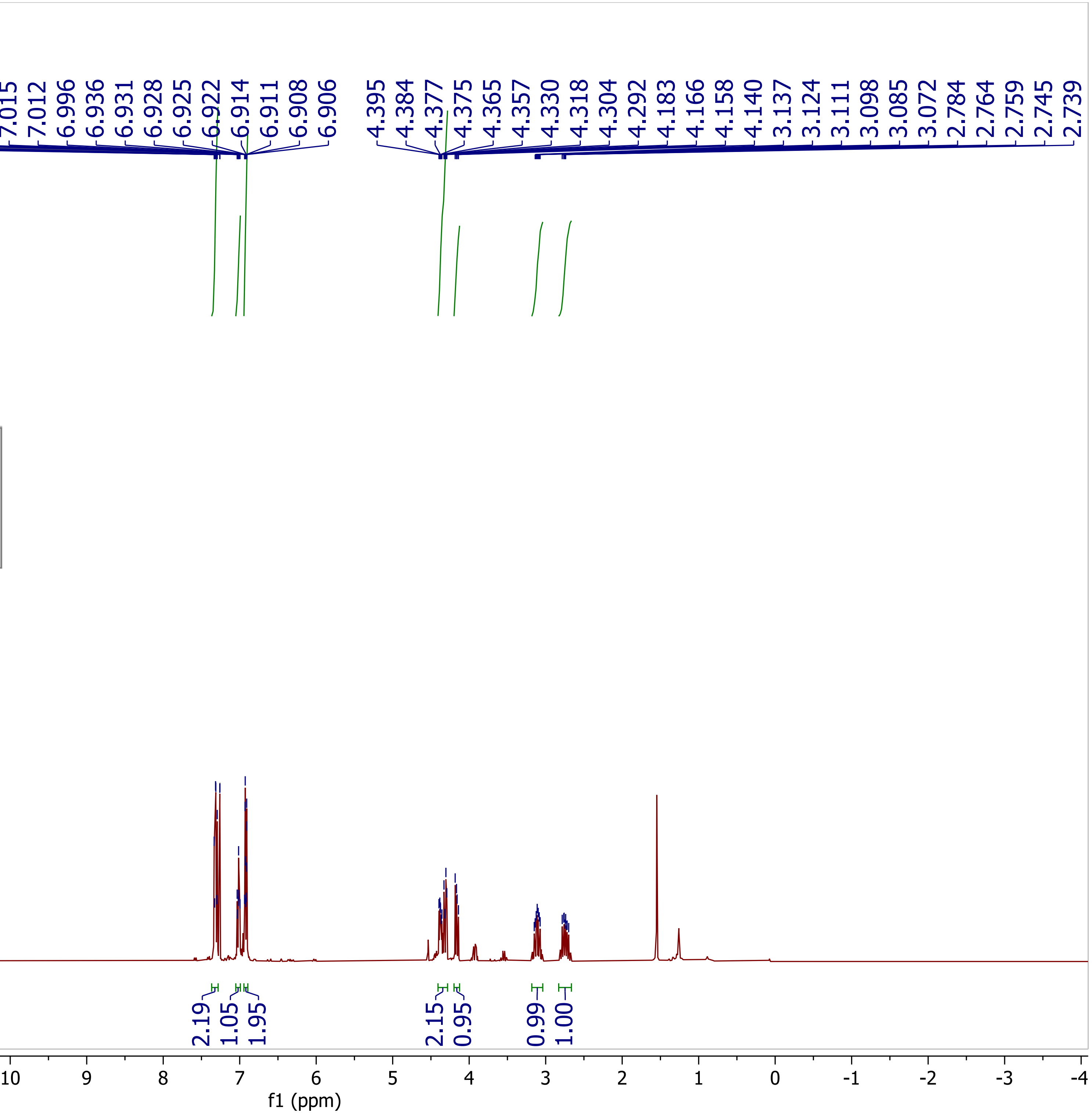

AA-04-203-tt-13-1H.8.fid — AA-04-203-tt-13

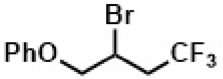

122

Parameter	Value
1 Solvent	CDCl3
2 Acquisition Date	2025-07-17T05:39:16
3 Spectrometer Frequency	100.63
4 Nucleus	(13C,)

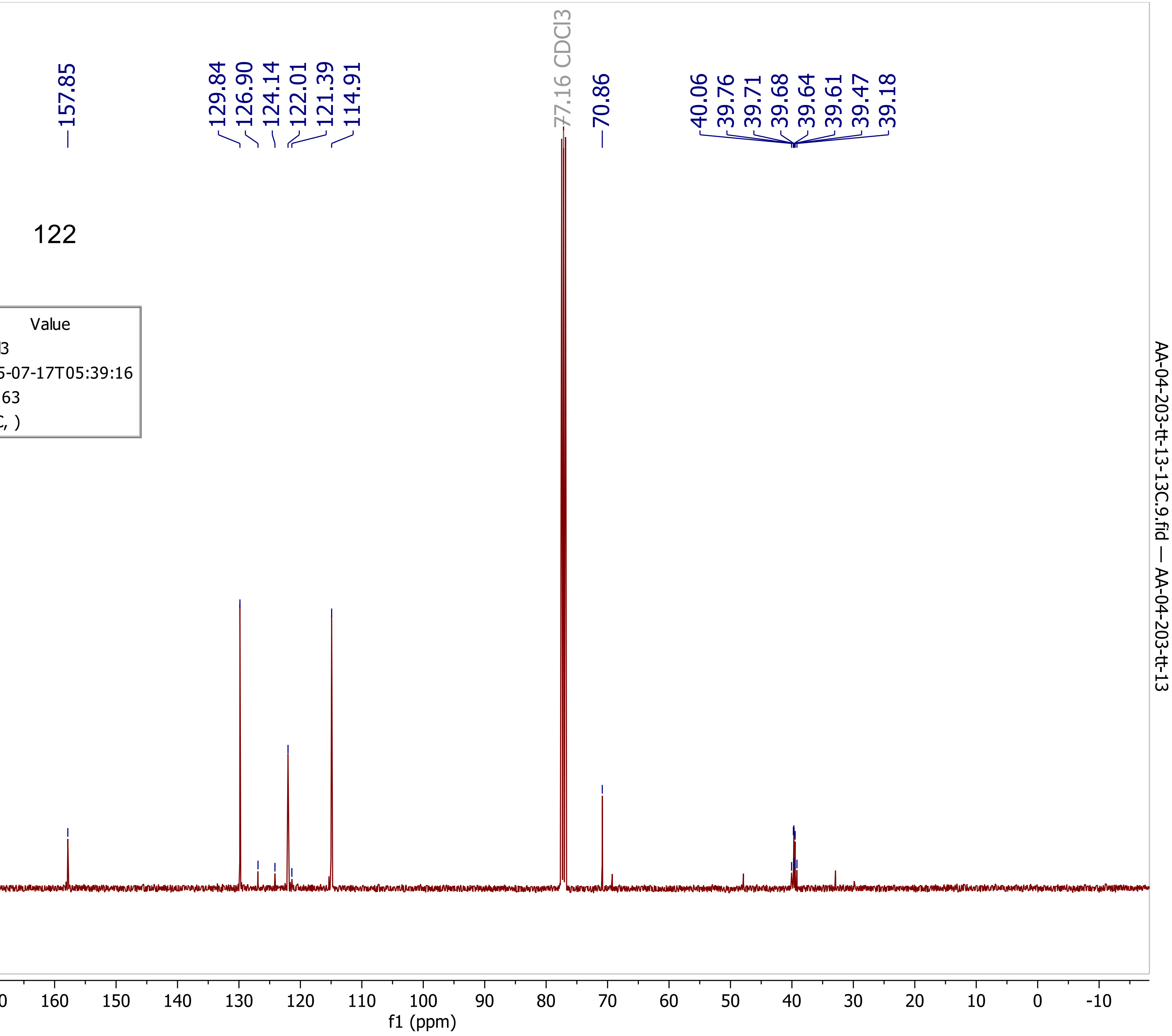

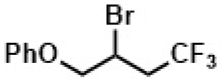

122

Parameter	Value
1 Solvent	CDCl3
2 Acquisition Date	2025-07-17T05:41:08
3 Spectrometer Frequency	376.47
4 Nucleus	(19F,)

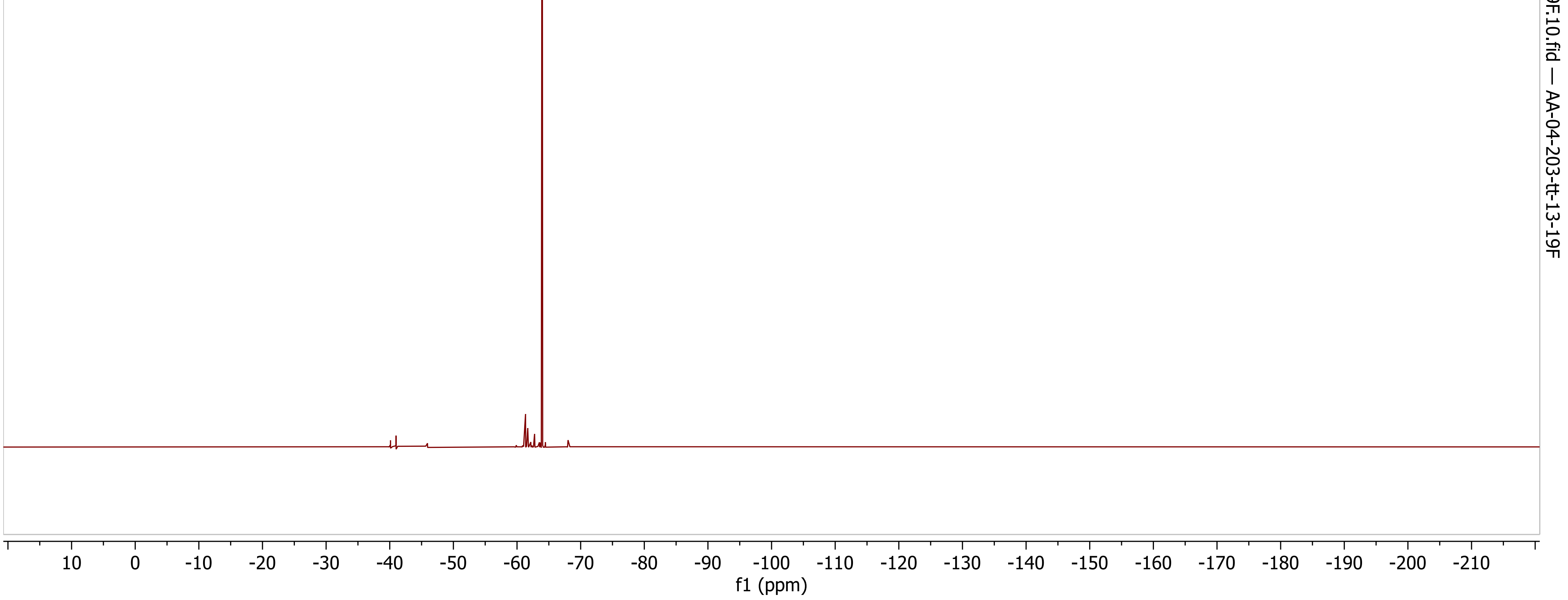

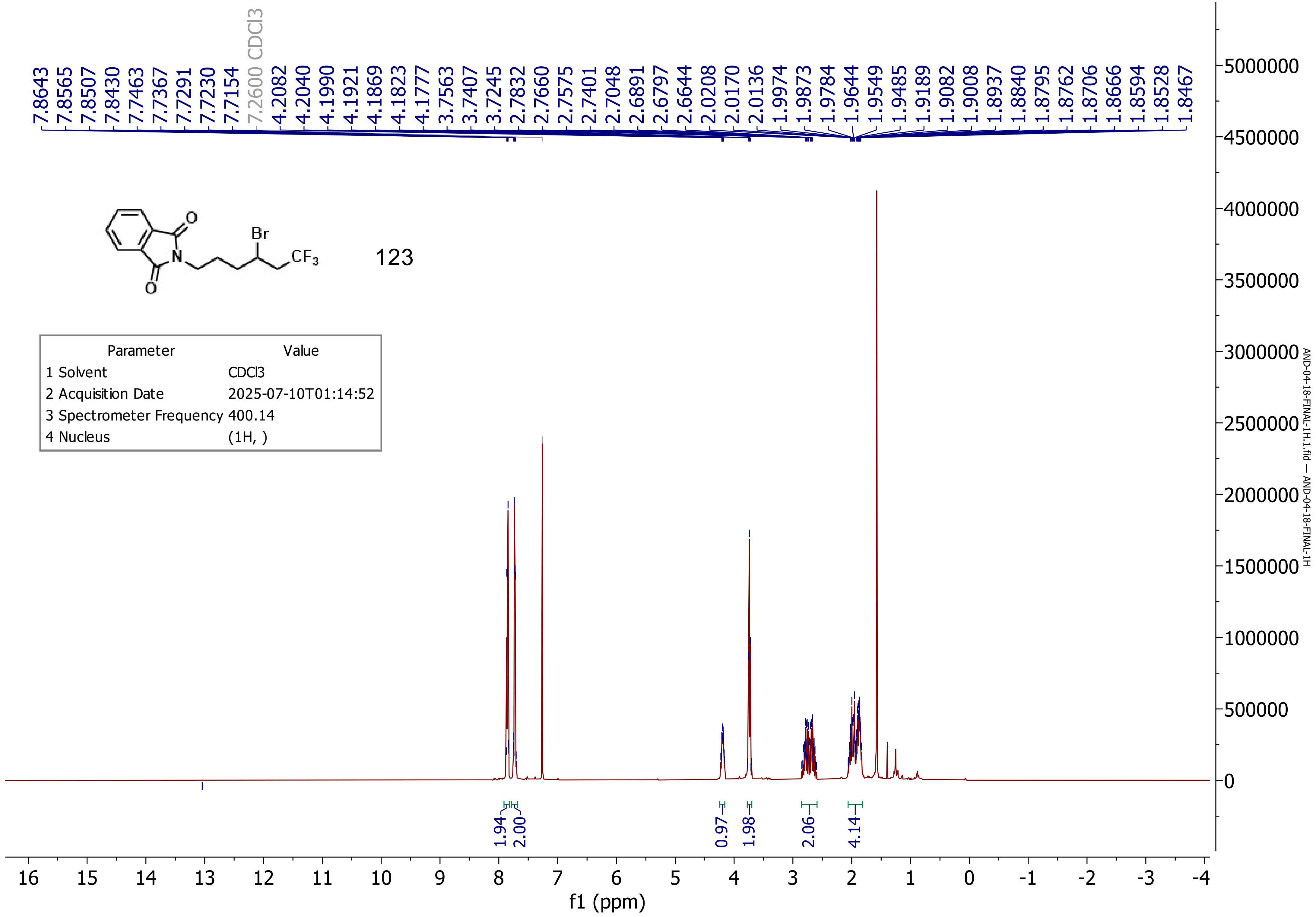

7.8643
7.8565
7.8507
7.8430
7.7463
7.7367
7.7291
7.7230
7.7154
7.2600 CDCl3
4.2082
4.2040
4.1990
4.1921
4.1869
4.1823
4.1777
3.7563
3.7407
3.7245
2.7832
2.7660
2.7575
2.7401
2.7048
2.6891
2.6797
2.6644
2.0208
2.0170
2.0136
1.9974
1.9873
1.9784
1.9644
1.9549
1.9485
1.9189
1.9082
1.9008
1.8937
1.8840
1.8795
1.8762
1.8706
1.8666
1.8594
1.8528
1.8467
Br
CF3
O
N
123
Parameter Value
1 Solvent CDCl3
2 Acquisition Date 2025-07-10T01:14:52
3 Spectrometer Frequency 400.14
4 Nucleus (1H,)
1.94
2.00
0.97
1.98
2.06
4.14
f1 (ppm)
AND-04-18-FINAL-1H.1.fid — AND-04-18-FINAL-1H

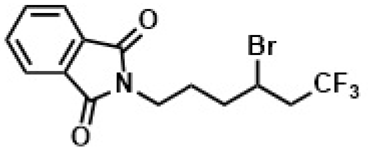

123

Parameter	Value
1 Solvent	CDCl3
2 Acquisition Date	2025-07-10T02:14:25
3 Spectrometer Frequency	100.63
4 Nucleus	(13C,)

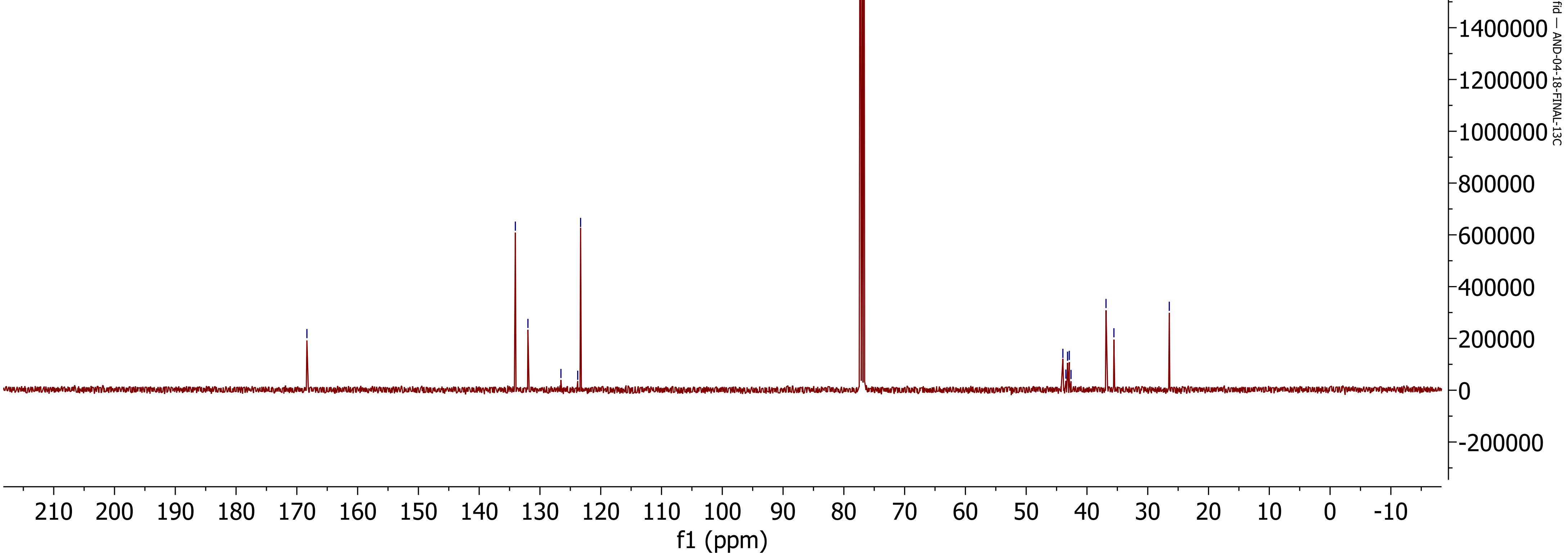

123

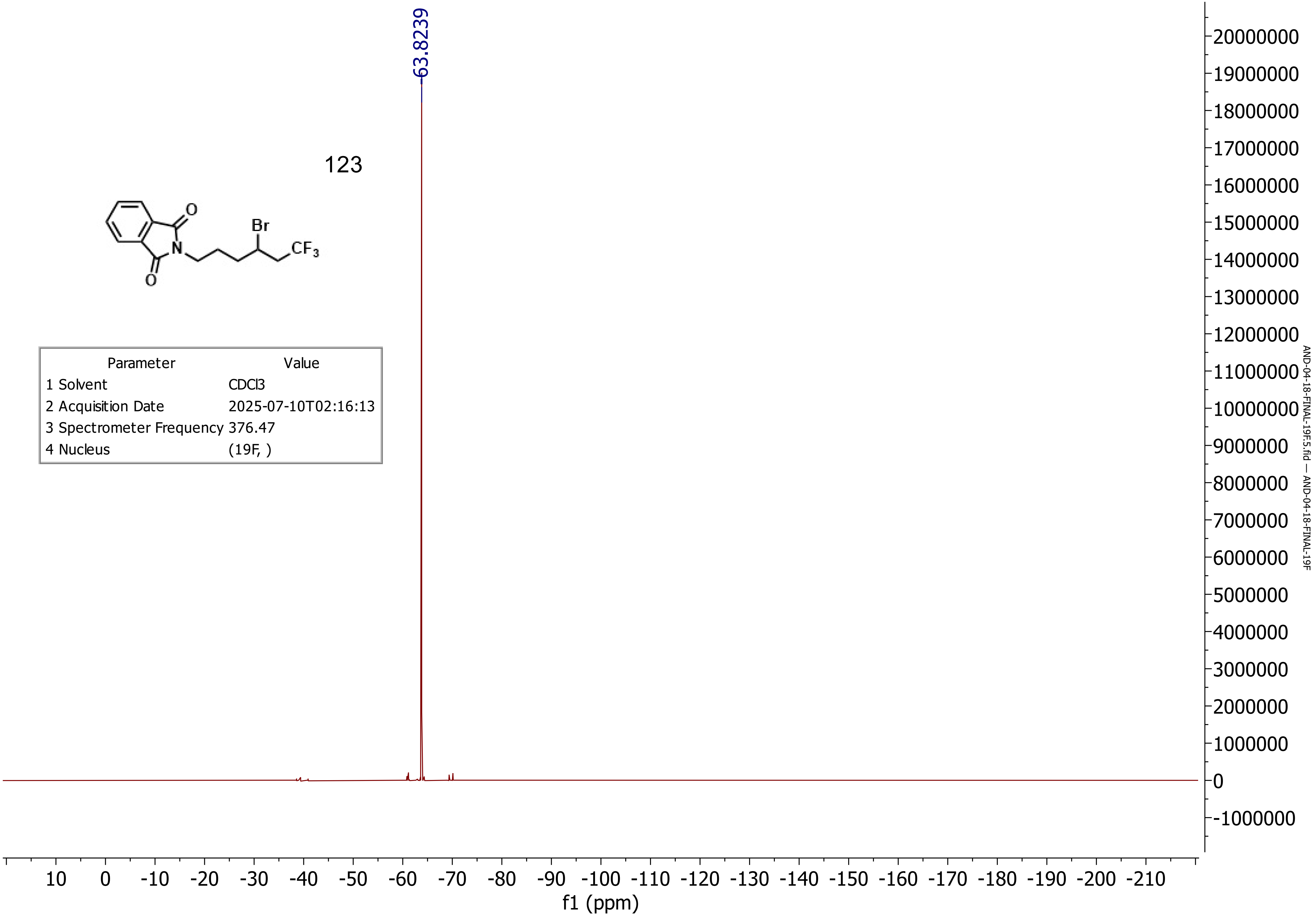

Parameter	Value
1 Solvent	CDCl3
2 Acquisition Date	2025-07-10T02:16:13
3 Spectrometer Frequency	376.47
4 Nucleus	(19F,)

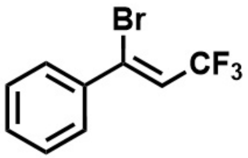
Br
CF3

124

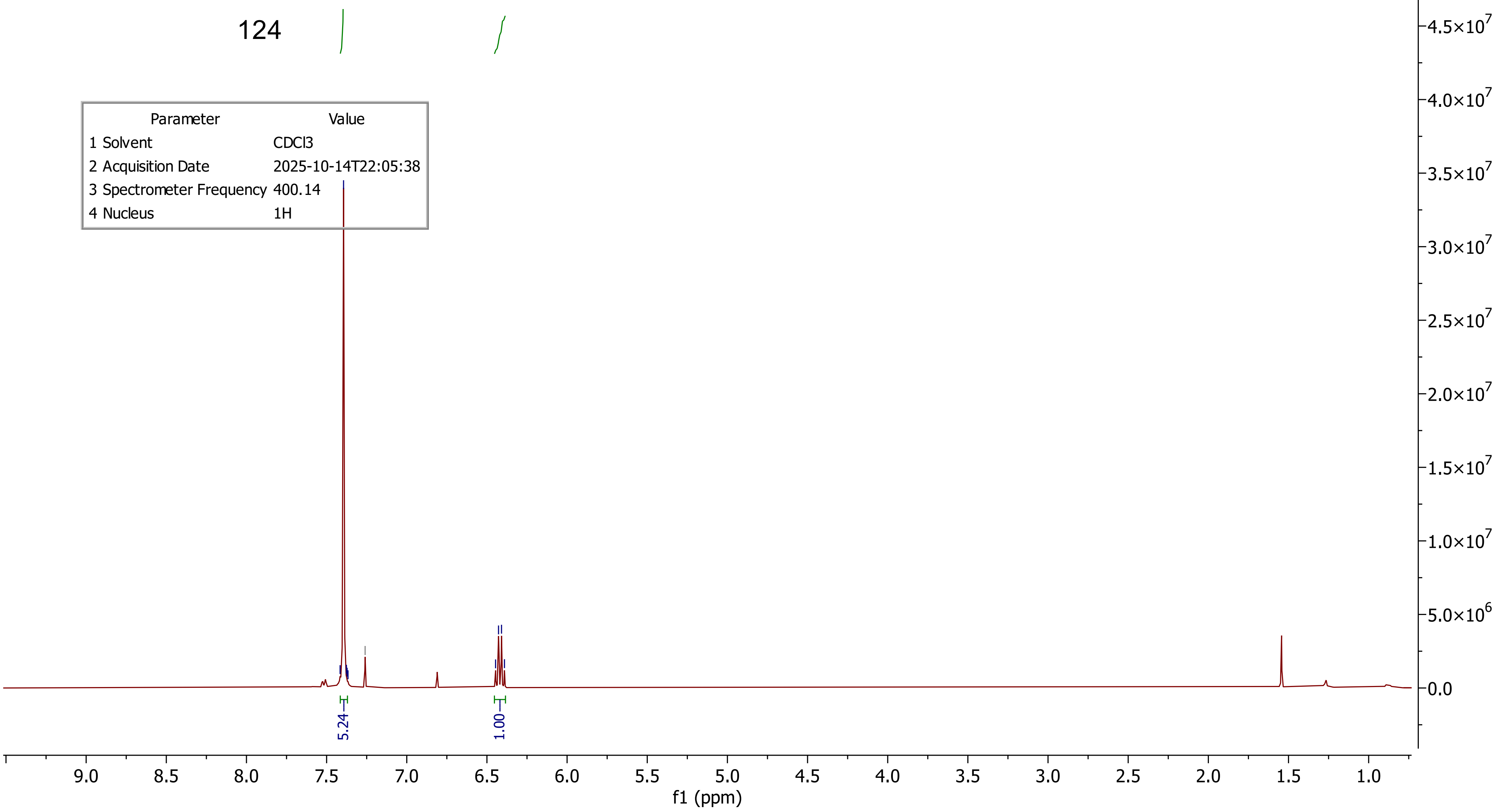
7.42
7.39
7.38
7.37
7.37
7.26 CDCl3
6.45
6.43
6.41
6.39
Parameter Value
1 Solvent CDCl3
2 Acquisition Date 2025-10-14T22:05:38
3 Spectrometer Frequency 400.14
4 Nucleus 1H
5.24
1.00
9.0
8.5
8.0
7.5
7.0
6.5
6.0
5.5
5.0
4.5
4.0
3.5
3.0
2.5
2.0
1.5
1.0
f1 (ppm)
6.0×10^7
5.5×10^7
5.0×10^7
4.5×10^7
4.0×10^7
3.5×10^7
3.0×10^7
2.5×10^7
2.0×10^7
1.5×10^7
1.0×10^7
5.0×10^6
0.0

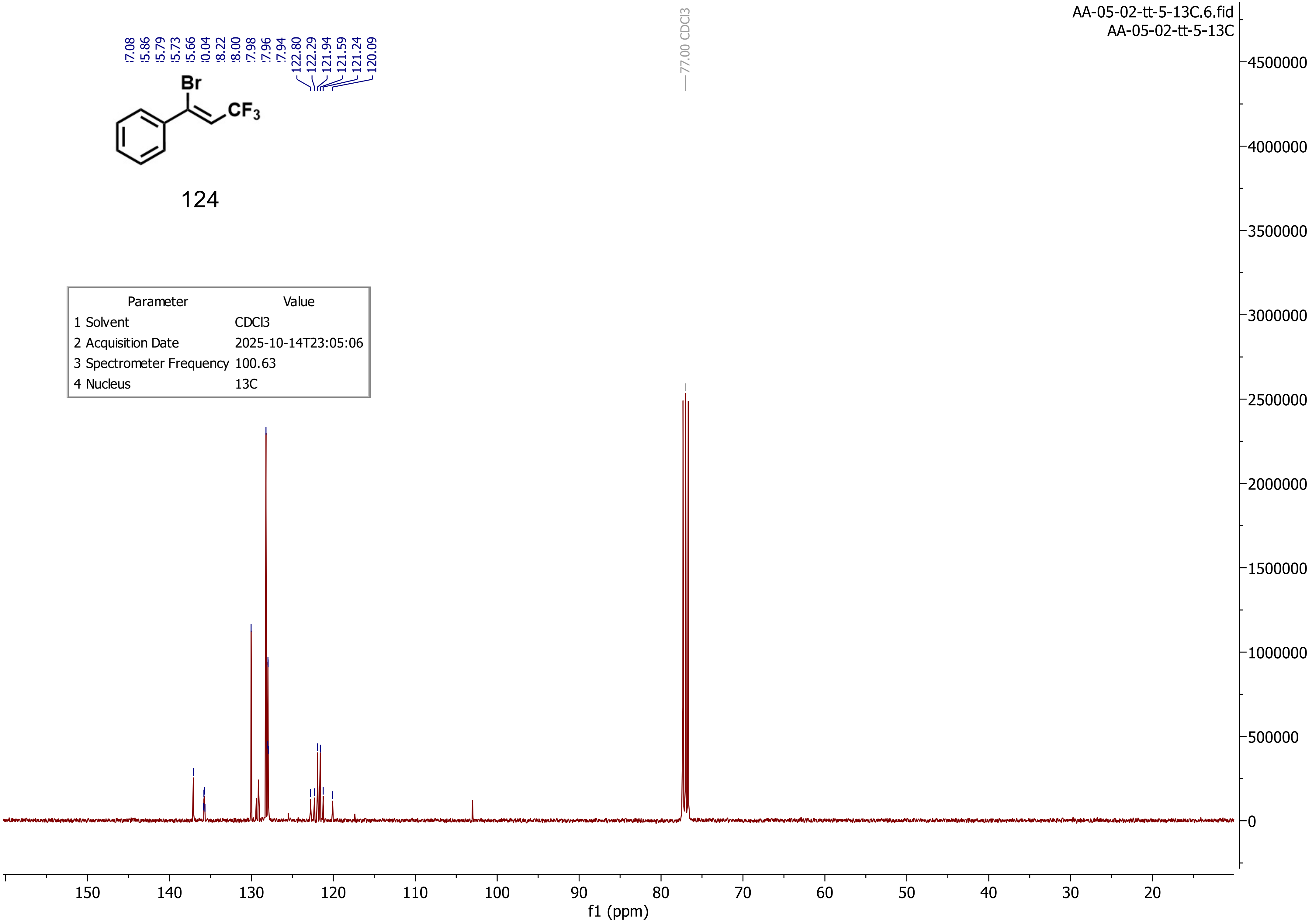

124

Parameter	Value
1 Solvent	CDCl3
2 Acquisition Date	2025-10-14T23:05:06
3 Spectrometer Frequency	100.63
4 Nucleus	13C

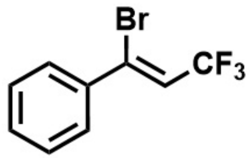

124

Parameter	Value
1 Solvent	CDCl3
2 Acquisition Date	2025-10-14T23:06:43
3 Spectrometer Frequency	376.47
4 Nucleus	19F

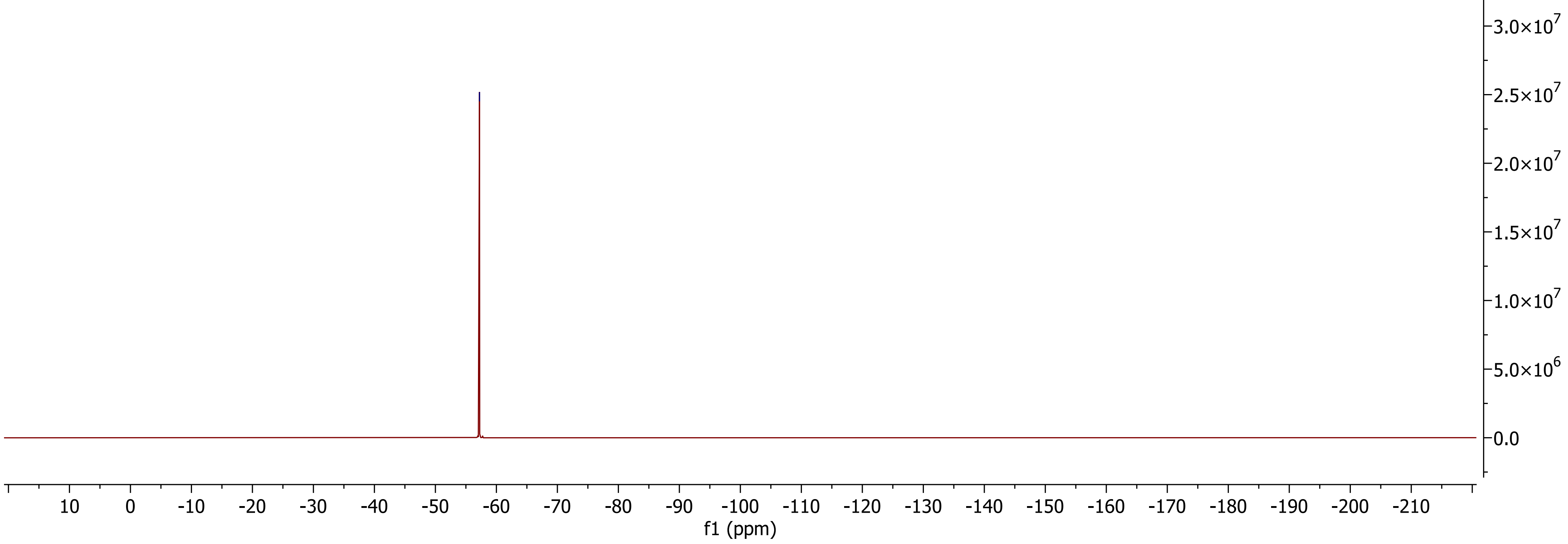

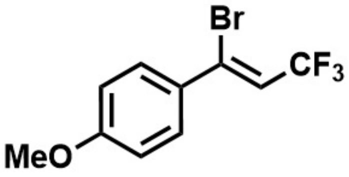

125

Parameter	Value
1 Solvent	CDCl3
2 Acquisition Date	2025-10-16T15:11:56
3 Spectrometer Frequency	400.13
4 Nucleus	1H

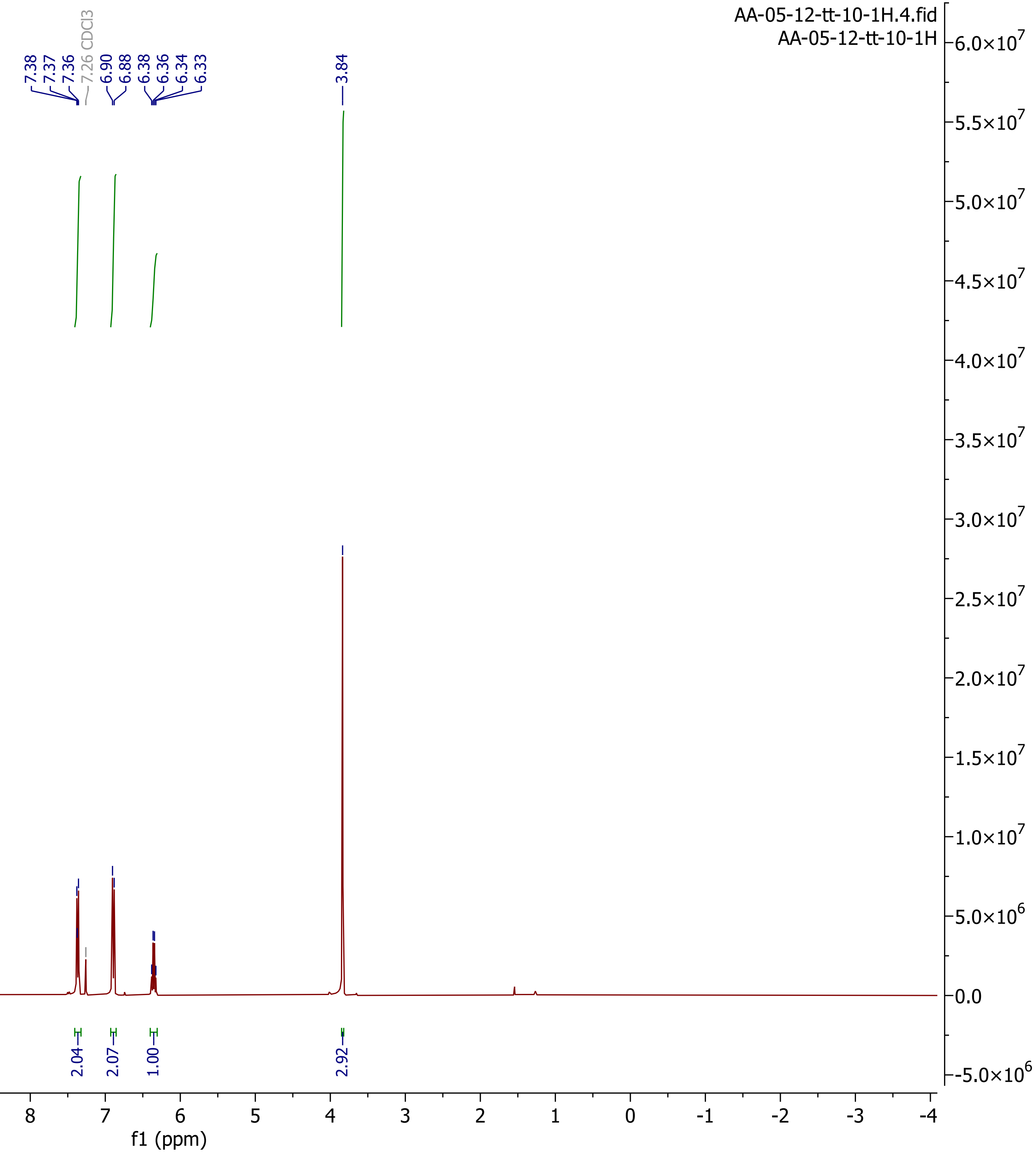

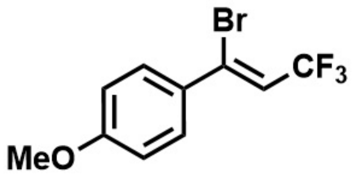

125

Parameter	Value
1 Solvent	CDCl3
2 Acquisition Date	2025-10-16T16:12:20
3 Spectrometer Frequency	100.62
4 Nucleus	13C

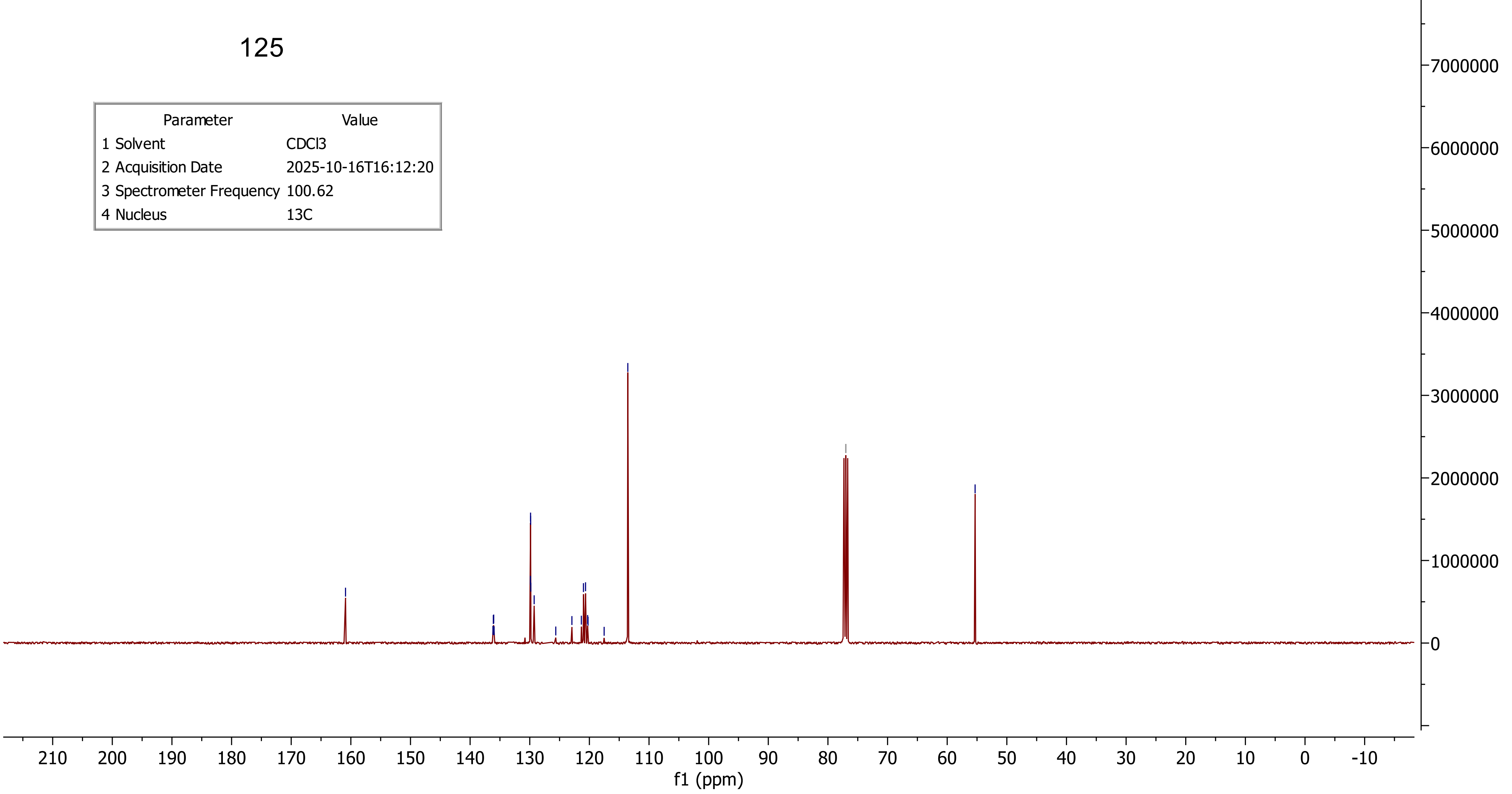

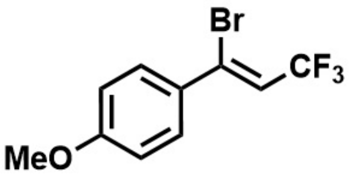

125

	Parameter	Value
1	Solvent	CDCl3
2	Acquisition Date	2025-10-16T16:16:58
3	Spectrometer Frequency	376.46
4	Nucleus	19F

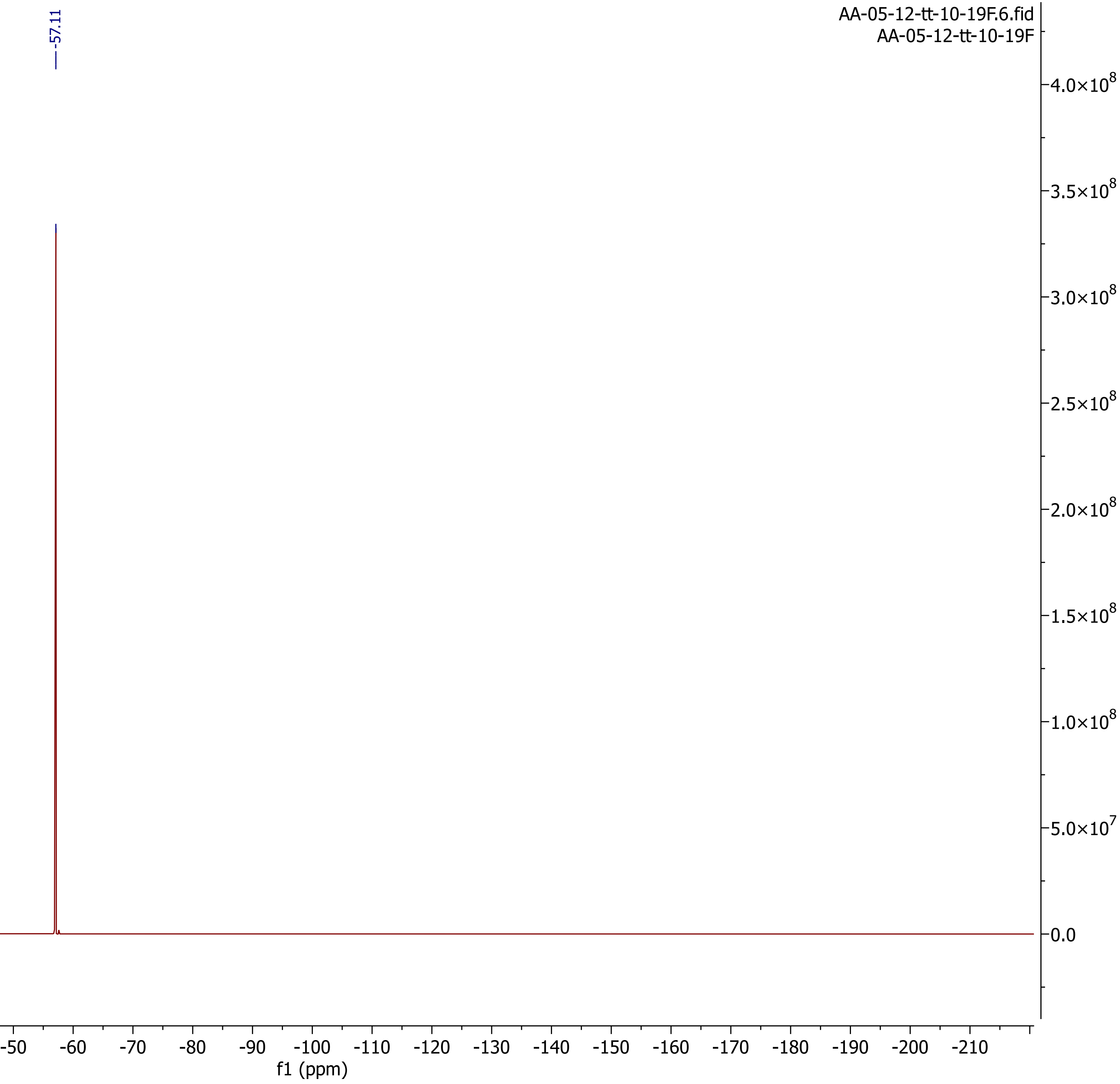

Supplement: Supplementary file 1 [file ja6c00567_si_001.pdf]
